# *O*‑Heterocycles-Fused Indoles via Tandem 1,2-Indole Migration–Hydroxycyclization Reactions in 2‑Hydroxyalkyl-3-propargylindoles

**DOI:** 10.1021/acs.orglett.6c00991

**Published:** 2026-04-06

**Authors:** Lorena Renedo, Marta Solas, Carlos Silva López, Samuel Suárez-Pantiga, Roberto Sanz

**Affiliations:** † Área de Química Orgánica, Departamento de Química, Facultad de Ciencias, 16725 Universidad de Burgos, Pza. Misael Bañuelos s/n, 09001-Burgos, Spain; ‡ Departamento de Química Orgánica, Facultad de Química, 16784 Universidade de Vigo, Campus Universitario, 36310-Vigo, Spain

## Abstract

Gold­(I)-catalyzed tandem cyclization reactions of 3-propargylindoles bearing a hydroxyalkyl substituent at C2 enable rapid access to indole-fused cyclic ethers through the vinylogous reactivity of α,β-unsaturated gold-carbene intermediates. A selective intramolecular Michael-type attack of the hydroxyl group, supported by DFT studies, affords indole-fused six-, seven-, and eight-membered *O*-heterocycles under mild conditions. A one-pot hydrogen transfer process further delivers reduced pyranoindoles, highlighting the versatility and synthetic potential of this method.

## Graphical abstract

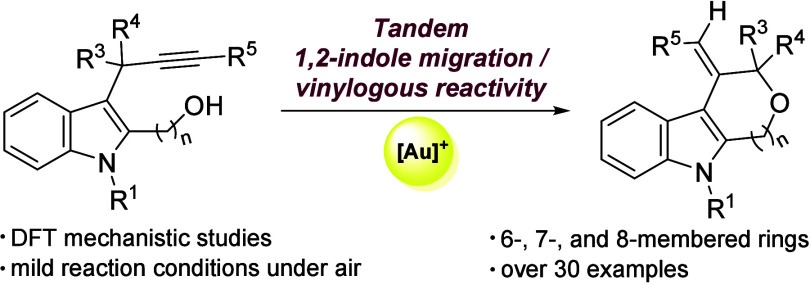

Gold catalysis has consolidated as a powerful synthetic tool, demonstrating broad compatibility with a wide range of functional groups to achieve molecular complexity.^1^ In particular, among the different reactivity patterns, gold complexes have proven to be highly effective for generating reactive metal-carbene complexes as safer alternatives to hazardous diazo compounds. These intermediates can be accessed, for example, through gold-activation of enynes^2^ or diynes,^3^ alkynes via nucleophilic addition of *N*-oxides or sulfoxides,^4^ or cycloheptatrienes.^5^ In this context, a variety of precursors have been successfully employed to generate alkenyl carbene intermediates, including propargyl esters via 1,2-acyloxy migration,^6^ cyclopropenes via ring-opening reactions,^7^ or propargyl sulfides via 1,2-sulfur migration,^8^ as well as through the decomposition of vinyl diazo compounds.^9^ Typically, these vinyl carbene intermediates undergo reactions at the carbene center,^6–9^ whereas their vinylogous reactivity at the β-position of the unsaturated gold-carbene has been comparatively less explored.^9,10^ Moreover, this reactivity mode has been most extensively studied for gold-carbene intermediates generated from vinyl diazoacetates,^9,10^ although notable alternative approaches have also emerged,^11^ revealing novel alternative reaction pathways of vinyl gold carbene species.

In this field, we have reported that readily available 3-propargylindoles^12^ undergo a gold­(I)-catalyzed 1,2-indole migration providing α,β-unsaturated gold-carbenes **A**, which can thus act as surrogates of vinyl carbenes. These highly reactive intermediates evolve through different pathways depending on the substituents at the propargylic and terminal positions of the alkyne (Scheme 1a).^13^ With aromatic groups at the propargylic position, an aura-iso-Nazarov cyclization occurs (path a). However, with aromatic-substituted alkynes (R^5^ = Ar and R^3^, R^4^ ≠ Ar), **A** evolves through an aura-Nazarov cyclization (path b). Both reactivities give rise to 3-(inden-2-yl)­indoles.^14^ However, when no aromatic groups are present at any of these positions (R^3^–R^5^), an alternative 1,2-hydride migration takes place, leading to 2-indol-3-yl-1,3-butadiene derivatives (path c).^13b^ Moreover, we have recently described inter- and intramolecular cyclopropanation reactions, the latter taking place with terminal alkynes and alkenyl substituents at C2 (path d).^15^

**Scheme 1:**
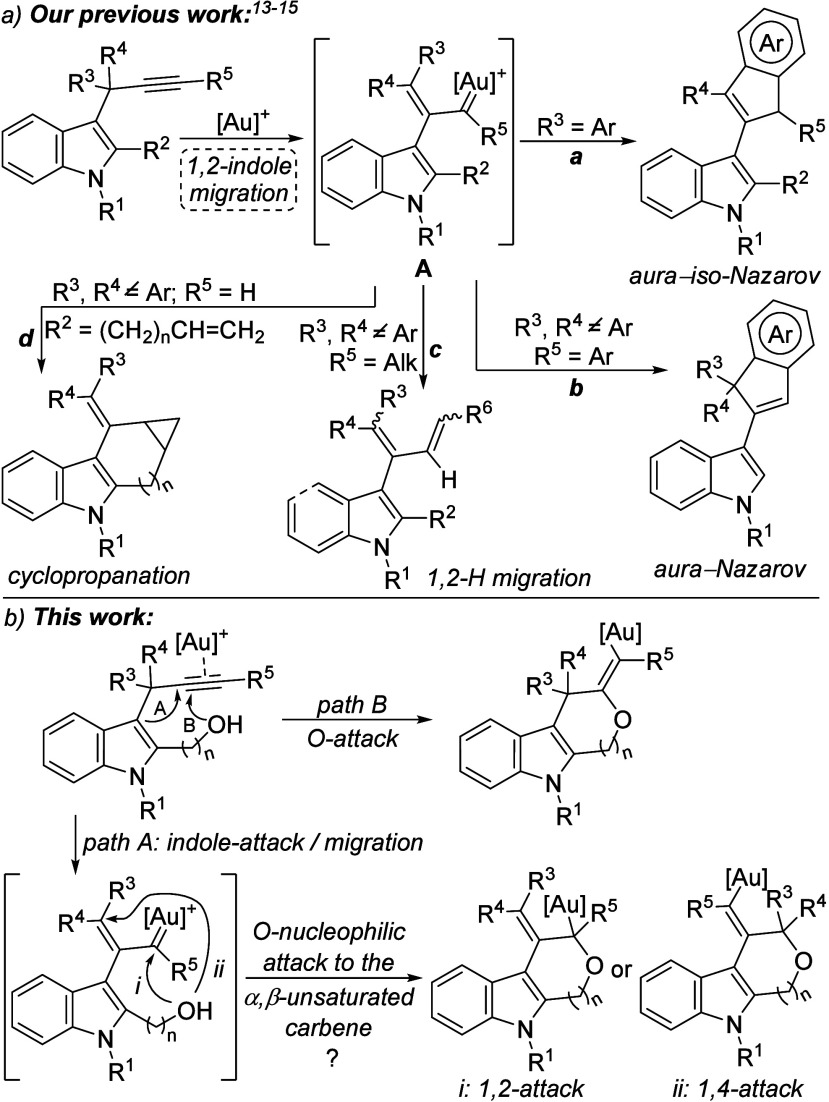
Known Reactivity of 3-Propargylindoles *via* Gold-carbene Intermediates and Proposed Work

At this point, we envisaged that highly electrophilic α,β-unsaturated gold-carbene intermediates **A** could undergo nucleophilic attack by a hydroxyl group located at C2 of the indole core, leading to interesting *O*-heterocycle-fused indole derivatives. However, at first, two different *O*-attacks, formally 1,2- and 1,4-, could occur, posing an interesting problem of site selectivity (Scheme 1b, path A). In addition, an alternative *O*-attack on the activated alkyne could compete with the indole-attack and subsequent migration (path B). We herein report our results about the gold-catalyzed reactions of 2-hydroxyalkyl-3-propargyl indoles.

2-Hydroxymethyl-3-propargylindole **1a** was selected as the model substrate and it was submitted to evolution under different gold­(I) complexes (Supporting Information (SI), Table S1). Considering our previous results,^15^ IPrAuNTf_2_ was initially selected as the catalyst that yielded selectively in high yield the tetrahydropyrano­[3,4-*b*]­indole **2a**,^16^ whereas JohnPhosAu­(MeCN)­SbF_6_ provided an even better and almost quantitative yield. The structure of **2a** shows that the hydroxyl group located at the alkyl chain of C-2 on **1a** selectively attacks the intermediate α,β-unsaturated carbene **B** in a formal 1,4-addition leading to a vinylgold **C**, which upon protodemetalation provides **2a** and recovers the catalyst (Scheme 2). Interestingly, the competitive direct attack of the hydroxy group on the activated alkyne was not observed.

**Scheme 2:**
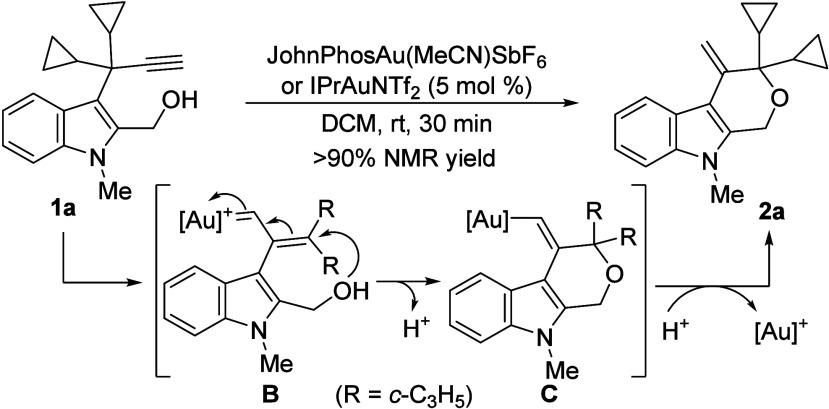
Preliminary Result with 2-Hydroxymethyl-3-propargylindole 1a: Mechanistic Proposal

After optimizing conditions, we proceeded to study the scope of the tandem process using different 2-hydroxy-functionalized 3-propargylindoles **1**, without aromatic groups at the propargylic positions and bearing a terminal alkyne (Table 1). Initially, indoles **1** were chosen to avoid the likely competitive tandem process involving 1,2-indole migration followed by (iso)­Nazarov cyclization to give indene-containing indole scaffolds. First, the reaction of model substrate **1a** could be carried out at a 2 mmol scale, showing the synthetic potential of this protocol, and leading to tetrahydropyranoindole **2a** in high yield (entry 1). Starting indoles **1b,c** with other alkyl groups at the propargylic position also gave rise to the expected derivatives **2b,c** (entries 2 and 3). Then 3-propargylindoles **1d**–**h** possessing secondary hydroxyl groups were tested, providing access to 1-substituted-4-methylenetetrahydro­pyrano­[3,4-*b*]­indoles **2d**–**h** in high yields (entries 4–8). Benzhydryl-type substrates **1i,j** also underwent this tandem process, leading to pyranoindoles **2i,j** (entries 9 and 10). Finally, N*H*-indoles **1k,l** were also evaluated, providing the corresponding N*H*-pyranoindoles **2k,l** (entries 11 and 12). Moreover, the structure of **2k** was further supported by X-ray analysis (CCDC 2533493),^17^ and it could also be prepared on a 2 mmol scale (entry 11).

**Table 1:**
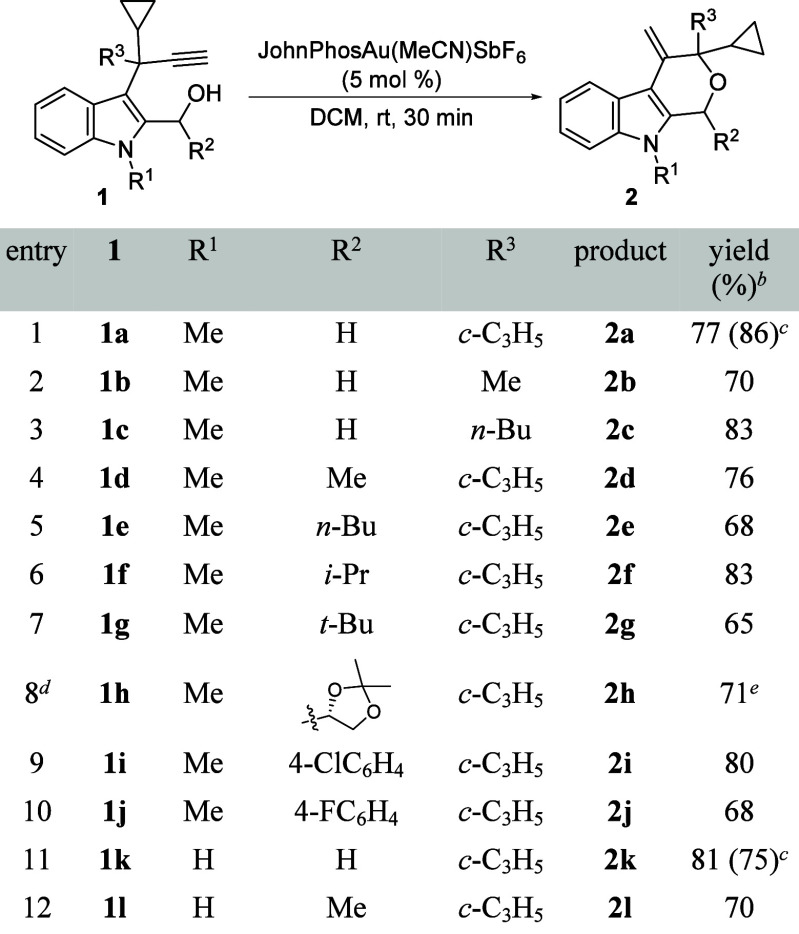
Synthesis of Tetrahydropyrano­[3,4-*b*]­indole Derivatives 2^a^

a Reaction conditions: **1** (0.5 mmol), JohnPhosAu­(MeCN)­SbF_6_ (5 mol %), in DCM (2 mL) at rt for 30 min.

b Isolated yield after column chromatography.

c Carried out with **1a** or **1k** (2 mmol) for 2 h.

d Derived from 2,3-*O*-isopropylidene-d-glyceraldehyde.

e **1h** and **2h** were a ca. 3/1 mixture of diastereoisomers.

In order to further explore the scope and potential of this procedure, we moved on to studying the possibility of synthesizing tetrahydroxepino­[3,4-*b*]­indoles **4** starting from 2-hydroxyethyl-3-propargylindole derivatives **3** (Table 2). The relatively challenging seven-membered ring could be formed onto the starting indole scaffold in good to high yields across a range of substrates.^18^ Notably, the protocol is compatible with primary alcohols (entries 1–3 and 15), as well as secondary (entries 4–13) and tertiary carbynols (entry 14), which reacted smoothly with little to no detrimental effects despite the increased steric demand (entries 4–14). Remarkably, the reaction also proceeded efficiently with N*H*-indole **3m** (entry 15). Interestingly, both enantiomers of secondary alcohol **3d** were readily prepared from (*S*)- and (*R*)-2-methyloxirane, affording the enantiomerically pure oxepinoindoles **(** ***S*** **)-4d** and **(** ***R*** **)-4d** (entries 5 and 6). Furthermore, the structure of **4a** was also confirmed by X-ray analysis (CCDC 2533495).^17^

**Table 2:**
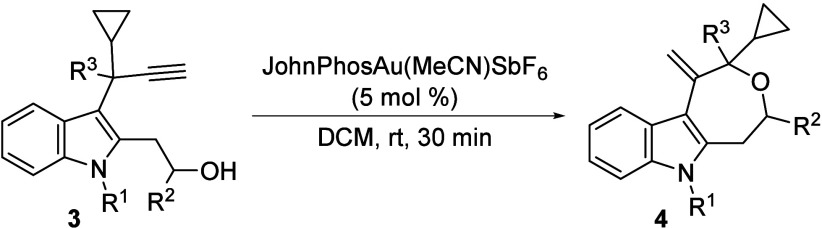
Synthesis of Tetrahydro-2*H*-oxepino­[4,5-*b*]­indole Derivatives 4^a^

entry	**3**	R^1^	R^2^	R^3^	product	yield (%)^b^
1	**3a**	Me	H	*c*-C_3_H_5_	**4a**	69
2	**3b**	Me	H	Me	**4b**	65
3	**3c**	Me	H	*n*-Bu	**4c**	89
4	**3d**	Me	Me	*c*-C_3_H_5_	**4d**	88
5	**(** ***S*** **)-3d**	Me	Me	*c*-C_3_H_5_	**(** ***S*** **)-4d**	78
6	**(** ***R*** **)-3d**	Me	Me	*c*-C_3_H_5_	**(** ***R*** **)-4d**	75
7	**3e**	Me	Me	Me	**4e**	71^c^
8	**3f**	Me	Et	*c*-C_3_H_5_	**4f**	80
9	**3g**	Me	*n*-Bu	*c*-C_3_H_5_	**4g**	79
10	**3h**	Me	*i*-Pr	*c*-C_3_H_5_	**4h**	73
11	**3i**	Me	Ph	*c*-C_3_H_5_	**4i**	76
12	**3j**	Me	CH_2_OPh	*c*-C_3_H_5_	**4j**	81
13	**3k**	Me	CH_2_O(allyl)	*c*-C_3_H_5_	**4k**	86
14	**3l**	Me	(Me)_2_	*c*-C_3_H_5_	**4l**	78
15	**3m**	H	H	*c*-C_3_H_5_	**4m**	80

a Reaction conditions: **1** (0.5 mmol), JohnPhosAu­(MeCN)­SbF_6_ (5 mol %), in DCM (2 mL) at rt for 30 min.

b Isolated yield after column chromatography.

c Obtained as a ca. 1/1 mixture of diastereoisomers.

Considering the positive results obtained with terminal 3-propargylindoles **1**, we next evaluated the reactivity of substrates bearing internal alkynes. First, as a foreseeable limitation, we confirmed that the Au-catalyzed reaction of phenyl-containing indoles **1m,n** led exclusively to the iso-Nazarov product **5**, from **1m**, and the Nazarov product **6**, from **1n**, with no traces of the corresponding products **2** (Scheme 3a). Next, methyl-substituted 3-propargylindole **1o** was subjected to the established reaction conditions and, surprisingly, the tetrahydropyrano­[3,4-*b*]­indole **2o** was selectively obtained, although competitive formation of a 3-dienylindole could have been expected.^13b^ In contrast, the reaction of the cyclopropyl-substituted alkyne **1p** afforded dienylindole **7p**, indicating that the 1,2-hydride migration is favored over intramolecular hydroxyl attack on the intermediate gold carbene.^13b^ Gratifyingly, after screening various conditions, we found that the use of IPrAuNTf_2_ in the presence of a catalytic amount of 3,5-dichloropyridine switched the reactivity toward desired pyranoindole **2p**. Disappointingly, under these conditions, the *n*-butyl-substituted substrate **1q** provided pyranoindole **2q** only as a minor product, with dienylindole **7q** being formed as the major one. Analogously, internal 2-hydroxyethyl-3-propargylindoles **3n**–**p** were examined. Similar results were observed for methyl- and cyclopropyl-substituted substrates **3n,o** compared with **1o,p** when IPrAuNTf_2_ was used in the presence of 3,5-dichloropyridine. However, with butyl-substituted **3p**, only dienylindole **8p** was obtained, highlighting the limitations of the hydroxycyclization pathway (Scheme 3b).^19^

**Scheme 3:**
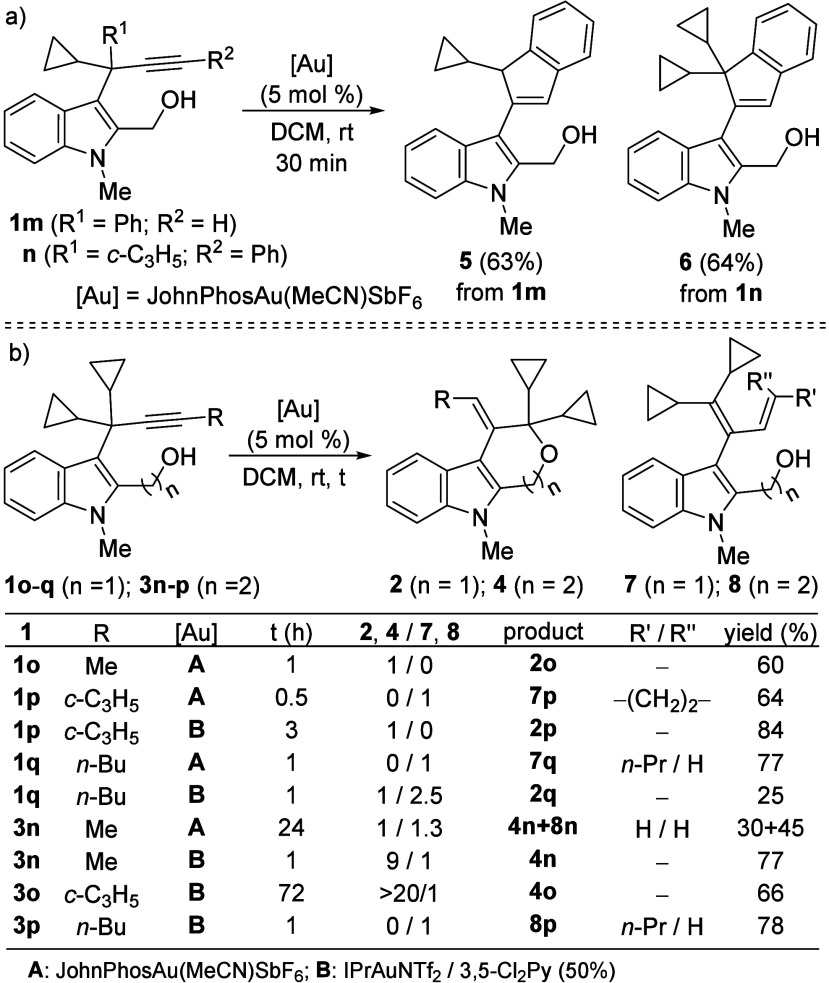
Au­(I)-Catalyzed Reactions of Aryl-Substituted Indoles 1m,n and Internal Alkynes 1o–q and 3n–p

With the auspicious results obtained above, we decided to test 3-propargylindoles **9** in which the hydroxyl group at the C2 position was lengthened even further. Under the established conditions, hexahydrooxocino­[5,4-*b*]­indoles **10**, with an eight-membered *O*-ring fused with the indole nucleus, were synthesized with moderate to good yields (Scheme 4).

**Scheme 4:**
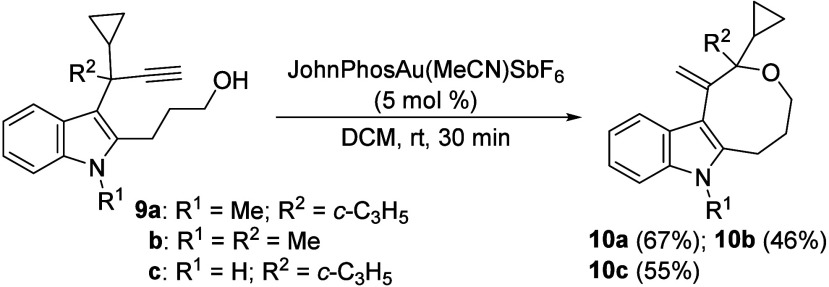
Synthesis of Hexahydrooxocino­[5,4-*b*]­indole Derivatives **10**

To rationalize the observed experimental results and to shed light onto the mechanisms that govern this reactivity, we resorted to mechanistic simulations. DFT simulations at the M06/6–31+G­(d,p) level (see SI for details) revealed an interesting mechanistic landscape with multiple exit paths in competition (Scheme 5a). From the parent indole **I** the 1,2-indole migration could compete with a direct nucleophilic attack for the oxygen atom onto the activated alkyne. This step is associated with a relatively low reaction barrier (ca. 16 kcal/mol), but the obtained adduct is unstable and reverts to the reactant barrierlessly (red arrows). The 1,2-indole migration therefore leads to a gold carbene with two key rotational isomers **III** and **III′**, each of which is ready to undergo 1,2- or 1,4-attack by the *O*-atom. The conjugated addition, however, is kinetically and thermodynamically favored, and the reaction is predicted to furnish **IV′**, as it is experimentally observed.

**Scheme 5:**
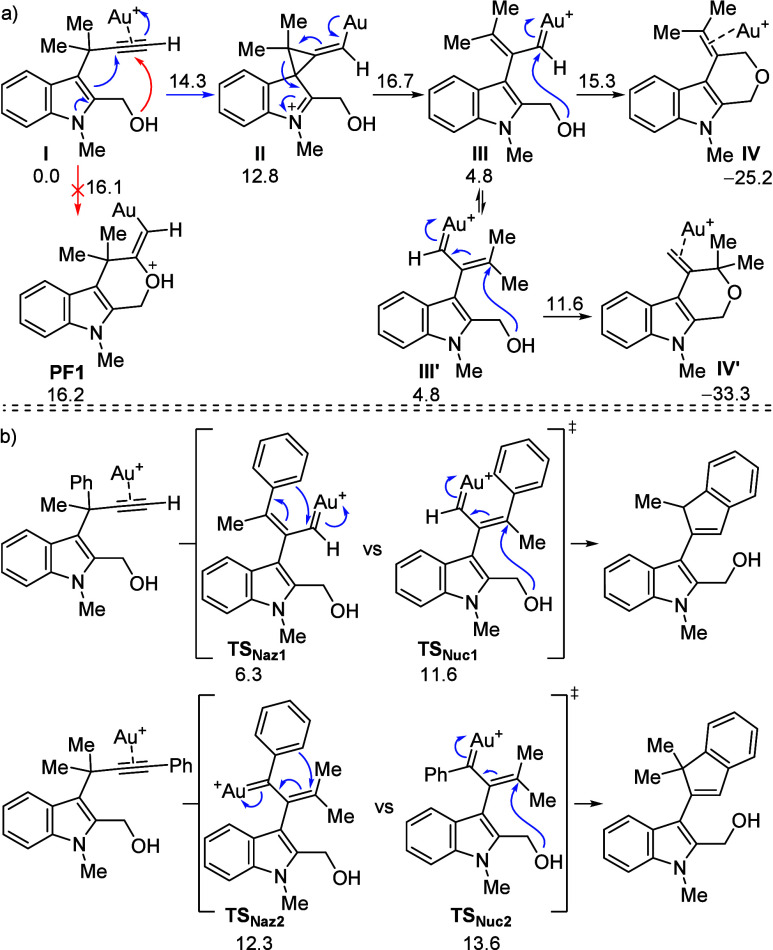
Computational Studies

When phenyl substitution appears at the alkynyl or propargyl site, a nucleophilic attack vs Nazarov-type reaction competition was observed in our experiments. This was also computationally simulated, and in both cases, the pericyclic transition state is favored over the nucleophilic alternative (**TS**_**Naz1**_ and **TS**_**Naz2**_ vs **TS**_**Nuc1**_ and **TS**_**Nuc2**_, respectively. Scheme 5b). Actually, in the case of propargylic substitution, the iso-Nazarov step is exceptionally efficient with a barrier of only 6.3 kcal/mol (**TS**_**Naz1**_). In the internal alkyne, it seems, however, that the two alternatives are closer in terms of kinetics and a substrate could perhaps be devised to steer reactivity toward the product of the *O*-attack.

Finally, we determined that the exocyclic double bond of 4-methylene­tetrahydro­pyrano­[3,4-*b*]­indoles **2** could be efficiently reduced with Hantzsch ester (HEH) without additional catalysis.^20^ Interestingly, this hydrogen transfer process was catalyzed by the same gold catalyst employed for the tandem reaction,^21^ and could be carried out in a one-pot manner to afford 4-methyl­tetrahydro­pyrano­[3,4-*b*]­indoles **11** from 3-propargylindoles **1** in high yields. From substrates bearing secondary alcohols, products **11c**–**f** were obtained as mixtures of diastereoisomers, although better stereoselectivity was observed when R^2^ is aromatic (**11e,f**). So, in our proposal, after formation of the pyranoindoles **2**, its activation could take place with the proton of the dihydropyridine or with the cationic gold, leading to an iminium intermediate **D** that was subsequently trapped with a hydride, delivering reduced pyranoindoles **11** (Scheme 6).

**Scheme 6:**
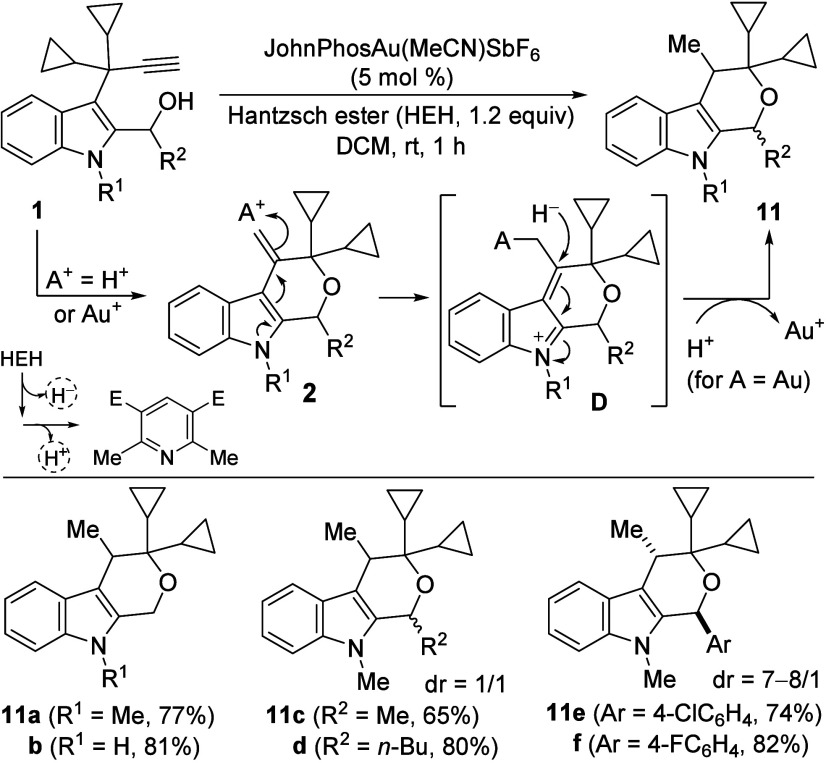
Synthesis of 4-Methyltetrahydropyrano­[3,4-*b*]­indole Derivatives **11**

In conclusion, we have developed a versatile procedure for the preparation of cyclic ethers fused to indole scaffolds by exploiting the reactivity at the vinylogous position of the alkenyl carbene intermediate generated from the initial 1,2-indole migration. The gold-catalyzed tandem reaction takes place in minutes for most precursors under mild conditions. Moreover, the method provides six-, seven-, and eight-membered ring ethers in good yields. Mechanistic investigations provided insight into the observed selectivity and opened new avenues for further exploration of this chemistry.

## Supplementary Material



## Data Availability

The data underlying this study are available in the published article, in its Supporting Information, and openly available in zenodo at 10.5281/zenodo.18832276.

## Notes

Experimental procedures, compound characterization, and NMR data, HPLC Traces, X-ray crystallographic data, computational sutdies, and copies of ^1^H and ^13^C­{^1^H} spectra (PDF)

## References

[1] Hashmi A. S. K. (2007). Gold-catalyzed
organic reactions. Chem. Rev..

[2] Luzung M. R., Markham J. P., Toste F. D. (2004). Catalytic
Isomerization
of 1,5-Enynes to Bicyclo[3.1.0]­hexenes. J. Am.
Chem. Soc..

[3] Ye L., Wang Y., Aue D. H., Zhang L. (2012). Experimental
and Computational Evidence for Gold Vinylidenes: Generation from Terminal
Alkynes via a Bifurcation Pathway and Facile C–H Insertions. J. Am. Chem. Soc..

[4] Zheng Z., Ma X., Cheng X., Zhao K., Gutman K., Li T., Zhang L. (2021). Homogeneous Gold-Catalyzed
Oxidation Reactions. Chem. Rev..

[5] Mato M., García-Morales C., Echavarren A. M. (2019). Generation
of Gold­(I) Carbenes by Retro-Buchner Reaction: From Cyclopropanes
to Natural Products Synthesis. ChemCatChem..

[6] Marion N., Nolan S. P. (2007). Propargylic esters in gold catalysis:
access to diversity. Angew. Chem., Int. Ed..

[7] Vicente R. (2021). C–C
Bond Cleavages of Cyclopropenes: Operating for Selective Ring-Opening
Reactions. Chem. Rev..

[8] Peng L., Zhang X., Zhang S., Wang J. (2007). Au-Catalyzed
Reaction of Propargylic Sulfides and Dithioacetals. J. Org. Chem..

[9] Bernardo O., García-Martínez P., Santamaría J., López L. A. (2024). Recent Advances in Gold-Catalyzed
Transformations of Vinyldiazo Reagents. Synthesis.

[10] Xu X., Zavalij P. Y., Hu W., Doyle M. P. (2013). Vinylogous Reactivity of Enol Diazoacetates with Donor-Acceptor
Substituted Hydrazones. Synthesis of Substituted Pyrazole Derivatives. J. Org. Chem..

[11] Rettenmeier E., Schuster A. M., Rudolph M., Rominger F., Gade C. A., Hashmi A. S. K. (2013). Gold Catalysis: Highly Functionalized
Cyclopentadienes Prepared by Intermolecular Cyclization of Ynamides
and Propargylic Carboxylates. Angew. Chem.,
Int. Ed..

[12] Sanz R., Miguel D., Álvarez-Gutiérrez J. M., Rodríguez F. (2008). Brønsted acid catalyzed C3-selective propargylation
and benzylation of indoles with tertiary alcohols. Synlett.

[13] Sanz R., Miguel D., Rodríguez F. (2008). Gold­(I)-catalyzed
tandem reactions initiated by 1,2-indole migrations. Angew. Chem., Int. Ed..

[14] Álvarez E., Miguel D., García García P., Fernández-Rodríguez M. A., Rodríguez F., Sanz R. (2011). Solvent- and ligand-induced switch of selectivity in gold­(I)-catalyzed
tandem reactions of 3-propargylindoles. Beilstein
J. Org. Chem..

[15] Renedo L., Solas M., Hernández-Ruiz R., Suárez-Pantiga S., Sanz R. (2025). Gold­(I)-Catalyzed Tandem
1,2-Indole Migration–Cyclopropanation
Reactions of 3-Propargylindoles with Olefins. Org. Lett..

[16] Lombardo V. M., Thomas C. D., Scheidt K. A. (2013). A Tandem Isomerization/Prins
Strategy: Iridium­(III)/Brønsted Acid Cooperative Catalysis. Angew. Chem., Int. Ed..

[17] See Supporting Information for details.

[18] Huang X., Shi Y., Wang Y., Jiao J., Tang Y., Li J., Xu S., Li Y. (2021). Synthesis
of Indole-Fused Oxepines via C-H Activation Initiated Diastereoselective
[5 + 2] Annulation of Indoles with 1,6-Enynes. Org. Lett..

[19] See Supporting Information for additional limitations regarding the substitution pattern and the nature of the nucleophilic site.

[20] **2k** was reduced to **11b** with HEH (2 equiv) in DCM (rt, 24 h).

[21] Han Z.-Y., Xiao H., Chen X.-H., Gong L.-Z. (2009). Consecutive Intramolecular
Hydroamination/Asymmetric Transfer Hydrogenation under Relay Catalysis
of an Achiral Gold Complex/Chiral Brønsted Acid Binary System. J. Am. Chem. Soc..

