# Supplementary material for: O‑Heterocycles-Fused Indoles via Tandem 1,2-Indole Migration–Hydroxycyclization Reactions in 2‑Hydroxyalkyl-3-propargylindoles
Source: Org Lett. 2026 Apr 6;28(15):4922–7. doi: 10.1021/acs.orglett.6c00991 (PMC13097240; doi:10.1021/acs.orglett.6c00991)

O-Heterocycles-fused Indoles via Tandem 1,2-Indole Migration-Hydroxycyclization Reactions in 2-Hydroxyalkyl-3-propargylindoles

Lorena Renedo,[†] Marta Solas,[†] Carlos Silva López,[‡] Samuel Suárez-Pantiga,[†] Roberto Sanz[†,*]

[†]*Área de Química Orgánica, Departamento de Química, Facultad de Ciencias, Universidad de Burgos, Pza. Misael Bañuelos s/n, 09001-Burgos, Spain*

[‡]*Departamento de Química Orgánica, Facultad de Química, Universidade de Vigo, Campus Universitario, 36310-Vigo, Spain*

Supporting Information

Table of Contents

General information S3

Synthesis and characterization data of 2-substituted indoles S1-22 S4

Procedure for the synthesis of (1-methyl-1H-indol-2-yl)methanol S1 S4

General procedure for the synthesis of 2-hydroxy-functionalized indoles S2,3,5 S4

General procedure for the synthesis of 2-hydroxy-functionalized indoles S4,6-8 S5

Procedure for the synthesis of 2-hydroxy-functionalized indol S10 S7

General procedure for the synthesis of 2-hydroxy-functionalized indoles S11-19 S7

Procedure for the synthesis of 2-hydroxy-functionalized indol S20 S10

Procedure for the synthesis of 2-hydroxy-functionalized indoles S21 S11

Procedure for the synthesis of 2-hydroxy-functionalized indoles S22 S11

Synthesis and characterization data of terminal 3-propargylindoles 1, 3, and 9 S12

General procedure for the synthesis of 3-propargylindole derivatives 1, 3, and 9 S12

Synthesis and characterization data of tetrahydropyrano[3,4-*b*]indole derivatives 2 and 1,3-dien-2-yl-indol derivatives 7 S24

Optimization studies S24

General procedure for the synthesis of tetrahydropyrano[3,4-b]indol derivatives 2a-l from 3-propargylindoles 1a-l S26

General procedure for the synthesis of tetrahydropyrano[3,4-b]indol derivatives 2o-q and 1,3-dien-2-yl-indol derivatives 7p,q S30

Synthesis and characterization data of tetrahydrooxepino[4,5-*b*]indol derivatives 4, indenylindoles 5 and 6, and 1,3-dien-2-yl-indol derivatives 8 S32

General procedure for the synthesis of tetrahydrooxepino[4,5-b]indol derivatives 4a-m S32

General procedure for the synthesis of tetrahydrooxepino[4,5-b]indoles 4n,o and 1,3-dien-2-yl-indol derivatives 8n,p S37

Reactions of phenyl-substituted 2-hydroxymethyl-3-propargylindoles 1m,n. Synthesis of 5 and 6 S39

Synthesis and characterization data of hexahydrooxocino[5,4-*b*]indole derivatives 10 S40

General procedure for the synthesis of hexahydrooxocino[5,4-b]indole derivatives 10a-c S40

Synthesis and characterization data of 4-methyltetrahydropyrano[3,4-*b*]indole derivatives 11 S41

General procedure for the synthesis of 4-methyltetrahydropyrano[3,4-b]indoles 11a-f S41

Further limitations about substrate scope S44

General procedure for the synthesis of 3-propargylindole derivatives S23,24 S44

Reactivity of 3-propargylindole S23 under gold(I)-catalysis S45

Reactivity of 3-propargylindole S24 under gold(I)-catalysis: Synthesis of 1,3-dien-2-yl-indol 8q S45

Procedure for the synthesis of 3-propargylindole derivative S26 S46

Reactivity of 3-propargylindole S26 under gold(I)-catalysis S47
Attempt of intermolecular reaction of 3-propargylindole S27 with MeOH under gold(I)-catalysis S47
HPLC Traces S48
X-Ray Crystallographic Data for 2k S50
X-Ray Crystallographic Data for 4a S51
Computational Studies S52
Cartesian coordinates S52
^{1}H and ^{13}C NMR Spectra S81

General information

General methods: All common reagents and solvents were obtained from commercial suppliers and used without any further purification. TLC was performed on aluminum-backed plates coated with silica gel 60 with F_{254} indicator; the chromatograms were visualized under ultraviolet light and/or by staining with a Ce/Mo reagent and subsequent heating. NMR spectra were measured on Varian Mercury-Plus 300 MHz, Bruker Avance 300 MHz and Bruker Avance 500 MHz spectrometers. ^{1}H NMR: splitting pattern abbreviations are: s, singlet; br s, broad singlet; d, doublet; t, triplet; q, quartet; dd, double doublet; ddd, doublets of doublets of doublets; ddt, double doublet of triplets; dt, doublet of triplets; dq, doublet of quartets; td, triplet of doublets; qd, quartet of doublets; quin, quintuplet; sext, sextet; hept, heptet; ad, apparent doublet; at, apparent triplet; aq, apparent quartet; as, apparent singlet; m, multiplet; the chemical shifts are reported in ppm using residual solvent peak as reference. ^{13}C NMR spectra were recorded at 75.4 MHz or 125.7 MHz using broadband proton decoupling and chemical shifts are reported in ppm using residual solvent peaks as reference ($CDCl_3$: δ 77.16) and the multiplicities were determined by DEPT experiments. Structural assignments were made with additional information from gCOSY and gNOESY experiments. High resolution mass spectra (HRMS) were recorded on a LC-MS instrument (1260 Infinity, Agilent) equipped with a QTOF analyzer using ESI (+). Low resolution mass spectra (LRMS) measurements were recorded on an Agilent 6890N/5973 Network GC System, equipped with a HP-5MS column. Melting points were measured on a Gallenkamp apparatus using open capillary tubes and are uncorrected. Melting points (m.p.) were measured on a Gallenkamp apparatus using open capillary tubes and are uncorrected. Enantioselectivity measurements: Agilent HPLC chromatograph equipped with Vis-UV Diode-Array detectors was used for the determination of the enantiomeric ratio; Chiralcel-OD-H and Chiralpak-AS-H were employed as chiral columns. Optical rotation values were recorded on a Carl Zeiss Kreipo 0.05 polarimeter. The specific rotation is calculated as follows:

$$\alpha_D^{25} = \left(\frac{\alpha_{obs}}{l \cdot c}\right) - \alpha_{solvent}$$

Thereby, α represents the recorded optical rotation, c the concentration of the analyte in g/mL and d the length of the cuvette in dm (0.5 dm). Usage of the sodium D line (λ = 589 nm) is indicated by D instead of the wavelength in nm. In all cases, acetone was used as the solvent (α = –0.2); the respective concentrations are specified for each compound.

The propargylic alcohols used for the synthesis of 3-propargylindoles **1**, **3**, and **9** were synthesized by adding the corresponding ketone to the organometallic species preformed from the corresponding terminal alkyne and *n*-BuLi, following a procedure described in the literature.[1]

(1*H*-Indol-2-yl)methanol (**S9**) (CAS-24621-70-3) is commercially available.

[1] Marín-Díaz. P.; Martínez-Nuñez. C.; Sanz. R.; Suárez-Pantiga. S. *Eur. J. Org. Chem.* **2024**, *27*, e202400147.

Synthesis and characterization data of 2-substituted indoles S1-22

Procedure for the synthesis of (1-methyl-1H-indol-2-yl)methanol S1

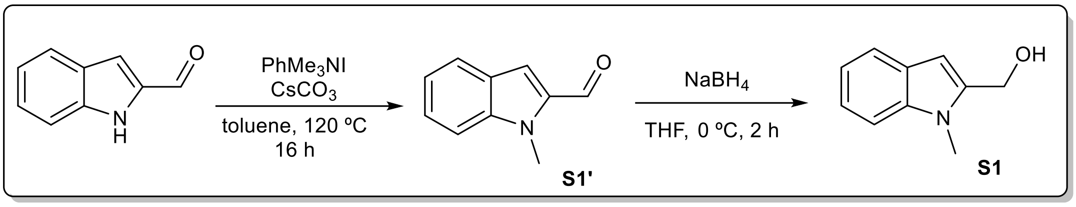

To a stirred solution of 1*H*-indole-2-carbaldehyde (0.72 g, 5 mmol) in toluene (15 mL), $PhMe_3NI$ (3.28 g, 12.5 mmol) and Cs_2CO_3 (3.25 g, 10 mmol) were placed in a Schlenk under an inert atmosphere. The reaction mixture was heated at 120 °C in an oil bath for 16 h. After complete conversion, monitored by GC-MS, the reaction was cooled to 0 °C and HCl (1 N, 5 mL) was slowly added. Then, the mixture was extracted with EtOAc (3 × 15 mL) and once with brine. The crude product **S1'** was used without further purification. The spectroscopic data of this compound match those reported in the literature.[2]

To a THF (15 mL) solution of 1-methyl-1*H*-indole-2-carbaldehyde (**S1'**) (795 mg, 5 mmol) obtained, $NaBH_4$ (0.28 g, 7.5 mmol) was slowly added with continuous stirring at 0 °C. The mixture was stirred for 2 h while it reached rt. The reaction was quenched with H_2O (10 mL) at 0 °C, and the mixture was extracted with Et_2O (3 × 15 mL). The combined organic layers were dried over anhydrous Na_2SO_4, and the solvent was removed under reduced pressure. The residue was purified by silica gel column chromatography using a 3/1 mixture of hexane and EtOAc as eluent to afford pure **S1** as a white solid (765 mg, 95%).

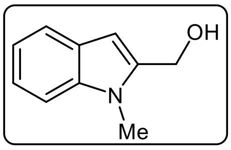

(1-Methyl-1*H*-indol-2-yl)methanol (S1): R_f = 0.25 (hexane/EtOAc, 3/1). M.p. = 108–110 °C (lit. M. p. = 108–110 °C).[3] The spectroscopic data for this compound match those reported in the literature.[3]

General procedure for the synthesis of 2-hydroxy-functionalized indoles S2,3,5

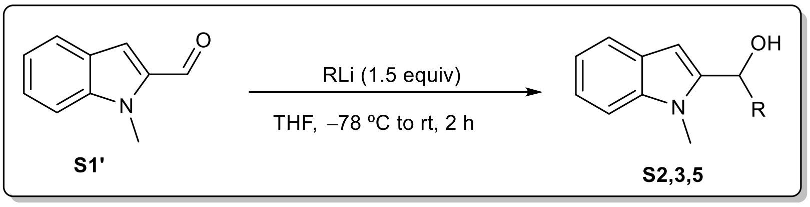

The corresponding organolithium reagent (15 mmol, 1.5 equiv) was added dropwise to a solution of 1-methyl-1*H*-indole (**S1'**, 1.59 g, 10 mmol, 1 equiv) in anhydrous THF (15 mL) at –78 °C under a nitrogen atmosphere. The mixture was allowed to warm to rt and stirred for 2 h. The reaction was quenched with aqueous NH_4Cl (10 mL), and the mixture was extracted with Et_2O (3 × 15 mL). The combined organic layers were dried over anhydrous Na_2SO_4, and the solvent was removed under reduced pressure. The residue was purified by silica gel column chromatography using mixtures of hexane/EtOAc as eluent to afford indoles **S2,3,5**.

---

[2] Templ, J.; Gjata, E.; Getzner, F.; Schnürch, M. *Org. Lett.* **2022**, *24*, 7315–7319.

[3] Lipshutz, B. H.; Hageman, M.; Fennewald, J. C.; Linstadt, R.; Slack, E.; Voigtritter, K. *Chem. Commun*. **2014**, *50*, 11378–11381.

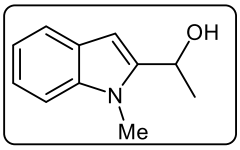

1-(1-Methyl-1*H*-indol-2-yl)ethan-1-ol (S2): Following the general procedure with MeLi (1.7 M in hexane, 8.8 mL, 15 mmol), the crude product was purified by column chromatography (hexane/EtOAc, 3/1), affording pure **S2** as a light white solid (1.57 g, 90%). R_f = 0.25 (hexane/EtOAc, 3/1). M. p. = 52–54 °C (lit. M. p. = 56.5–57.5 °C).[4] The spectroscopic data for this compound match those reported in the literature.[4]

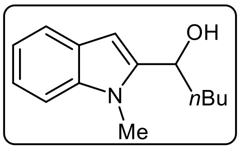

1-(1-Methyl-1*H*-indol-2-yl)pentan-1-ol (S3): Following the general procedure with *n*-BuLi (2.5 M in hexane, 6 mL, 15 mmol), the crude product was purified by column chromatography (hexane/EtOAc, 3/1), affording pure **S3** as a white solid (1.80 g, 83%). R_f = 0.21 (hexane/EtOAc, 3/1). M.p. = 93–95 °C. **^{1}H NMR** (300 MHz, $CDCl_3$): δ (ppm) = 7.62 (d, J = 7.8 Hz, 1H), 7.35 (d, J = 8.1 Hz, 1H), 7.2 (at, J = 7.4 Hz 1H), 7.14 (at, J = 7.4 Hz, 1H), 6.47 (s, 1H), 4.85 (q, J = 6.4 Hz, 1H), 3.83 (s, 3H), 2.10–1.95 (m, 2H), 1.71 (d, J = 6.0 Hz, 1H), 1.65–1.53 (m, 1H), 1.52–1.33 (m, 3H), 0.98 (t, J = 7.0 Hz, 3H). **^{13}C NMR** (75.4 MHz, $CDCl_3$): δ (ppm) = 142.2 (C), 138.1 (C), 127.3 (C), 121.9 (CH), 120.8 (CH), 119.6 (CH), 109.2 (CH), 99.0 (CH), 67.5 (CH), 36.1 (CH_2), 30.2 (CH_3), 28.4 (CH_2), 22.7 (CH_2), 14.2 (CH_3). **HRMS** (ESI-TOF) *m/z*: $[M+H]^+$ Calcd for $C_{14}H_{20}NO^+$ 218.1539; Found 218.1546.

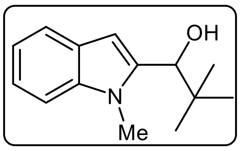

2,2-Dimethyl-1-(1-methyl-1*H*-indol-2-yl)propan-1-ol (S5): Following the general procedure with *t*-BuLi (1.7 M in pentane, 8.8 mL, 15 mmol), the crude product was purified by column chromatography (hexane/EtOAc, 4/1), affording pure **S5** as a yellow oil (1.38 g, 64%). R_f = 0.21 (hexane/EtOAc, 4/1). **^{1}H NMR** (300 MHz, $CDCl_3$): δ (ppm) = 7.74 (d, J = 7.7 Hz, 1H), 7.42 (d, J = 8.1 Hz, 1H), 7.37 (at, J = 7.4 Hz, 1H), 7.28 (at, J = 7.2 Hz, 1H), 6.59 (s, 1H), 4.71 (s, 1H), 3.76 (s, 3H), 2.37 (br s, 1H), 1.17 (s, 9H). **^{13}C NMR** (75.4 MHz, $CDCl_3$): δ (ppm) = 141.7 (C), 137.4 (C), 127.5 (C), 121.4 (CH), 120.6 (CH), 119.7 (CH), 109.4 (CH), 100.3 (CH), 74.9 (CH), 36.7 (CH_3), 30.7 (C), 26.1 (3 × CH_3). **HRMS** (ESI-TOF) *m/z*: $[M+H]^+$ Calcd for $C_{14}H_{20}NO^+$ 218.1539; Found 218.1547.

General procedure for the synthesis of 2-hydroxy-functionalized indoles S4,6-8

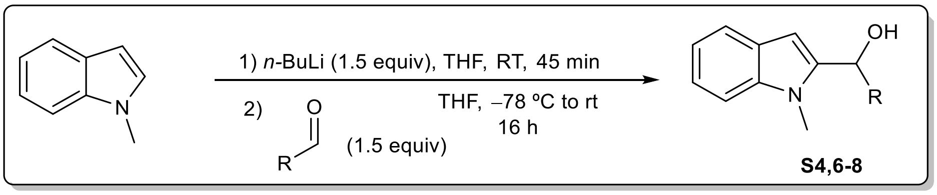

n-BuLi (2.5 M solution in hexane, 6 mL, 15 mmol, 1.5 equiv) was slowly added to a solution of 1-methyl-1*H*-indole (1.31 g, 10 mmol, 1 equiv) in anhydrous THF (15 mL) at rt under a nitrogen atmosphere. The resulting mixture was stirred at this temperature for 45 min. The corresponding aldehyde (15 mmol, 1.5 equiv) was added at –78 °C, and the reaction mixture was allowed to warm to rt and stirred overnight. The reaction was quenched with aqueous NH_4Cl (10 mL), and the mixture was extracted with Et_2O (3 × 15 mL). The combined organic layers were dried over anhydrous Na_2SO_4, and the solvent was removed under reduced pressure. The residue was purified by silica gel column chromatography using mixtures of hexane/EtOAc as eluent to afford indoles **S4,6-8**.

---

[4] Ziegler, F. E.; Spitzner, E. B.; Wilkins, C. K. *J. Org. Chem.* **1971**, *36*, 13, 1759-1764.

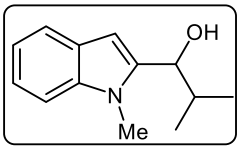

2-Methyl-1-(1-methyl-1*H*-indol-2-yl)propan-1-ol (S4): Following the general procedure with isobutyraldehyde (1.08 g, 15 mmol), the crude product was purified by column chromatography (hexane/EtOAc, 3/1), affording pure **S4** as a white solid (1.32 g, 65%). R_f = 0.28 (hexane/EtOAc, 3/1). M.p. = 84–86 °C. **^{1}H NMR** (300 MHz, $CDCl_3$): δ (ppm) = 7.65 (d, *J* = 7.8 Hz, 1H), 7.35 (d, *J* = 8.2 Hz, 1H), 7.28 (at, *J* = 8.0 Hz, 1H), 7.17 (at, *J* = 7.3 Hz, 1H), 4.48 (d, *J* = 7.1 Hz, 1H), 3.76 (s, 3H), 2.30–2.16 (m, 2H), 1.19 (d, *J* = 6.7 Hz, 3H), 0.96 (d, *J* = 6.7 Hz, 3H). **^{13}C NMR** (75.4 MHz, $CDCl_3$): δ (ppm) = 141.6 (C), 137.9 (C), 127.3 (C), 121.6 (CH), 120.6 (CH), 119.5 (CH), 109.2 (CH), 99.8 (CH), 73.3 (CH), 33.5 (CH), 30.3 (CH_3), 19.9 (CH_3), 18.8 (CH_3). **HRMS** (ESI-TOF) *m/z*: $[M+H]^+$ Calcd for $C_{13}H_{18}NO^+$ 204.1383; Found 204.1387.

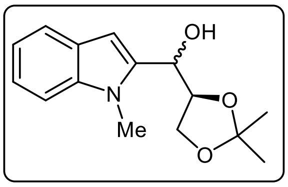

((*S*)-2,2-Dimethyl-1,3-dioxolan-4-yl)(1-methyl-1*H*-indol-2-yl)methanol (S6): Following the general procedure with (*R*)-2,2-dimethyl-1,3-dioxolane-4-carbaldehyde (1.95 g, 15 mmol), the crude product was purified by column chromatography (hexane/EtOAc, 3/1), affording pure **S6** as a yellow solid (1.72 g, 66%). R_f = 0.25 (hexane/EtOAc, 3/1). M.p. = 92–94 °C. Obtained and isolated as a ca. 2.4/1 mixture of diastereoisomers. Data for both isomers: **^{1}H NMR** (300 MHz, $CDCl_3$): δ (ppm) = 7.69 (d, *J* = 7.9 Hz, 2H), 7.41–7.31 (m, 4H), 7.22 (m, 2H), 6.47 (s, 2H), 4.90 (t, *J* = 4.8 Hz, 1H), 4.78 (t, *J* = 4.4 Hz, 1H), 4.65 (q, *J* = 6.5 Hz, 1H), 4.49 (q, *J* = 6.1 Hz, 1H), 4.27–4.15 (m, 1H), 4.14–4.04 (m, 1H), 3.81–3.77 (m, 2H), 3.79 (s, 3H), 3.72 (s, 3H), 3.30 (d, *J* = 4.8 Hz, 1H), 3.02 (d, *J* = 4.4 Hz, 1H), 1.60 (s, 6H), 1.55 (s, 6H). **^{13}C NMR** (75.4 MHz, $CDCl_3$): δ (ppm) = 138.5 (C), 137.8 (C), 137.7 (C), 137.5 (C), 127.1 (C), 127.0 (C), 122.0 (CH), 121.7 (CH), 120.74 (CH), 120.68 (CH), 119.6 (CH), 119.5 (CH), 110.0 (C), 109.4 (C), 109.2 (CH), 109.1 (CH), 100.3 (CH), 99.6 (CH), 77.6 (CH), 77.6 (CH), 68.2 (CH), 67.3 (CH), 66.5 (CH_2), 65.9 (CH_2), 30.03 (CH_3), 29.95 (CH_3), 26.7 (CH_3), 26.4 (CH_3), 25.2 (CH_3), 24.9 (CH_3). **HRMS** (ESI-TOF) *m/z*: $[M+H]^+$ Calcd for $C_{15}H_{20}NO_3^+$ 262.1438; Found 262.1439.

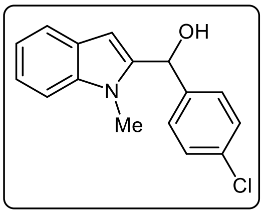

(4-Chlorophenyl)(1-methyl-1*H*-indol-2-yl)methanol (S7): Following the general procedure with 4-chlorobenzaldehyde (2.17 g, 15 mmol), the crude product was purified by column chromatography (hexane/EtOAc, 3/1), affording pure **S7** as a light yellow oil (2.27 g, 84%). R_f = 0.28 (hexane/EtOAc, 5/1). The spectroscopic data for this compound match those reported in the literature.[5]

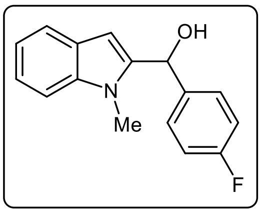

(4-Fluorophenyl)(1-methyl-1*H*-indol-2-yl)methanol (S8): Following the general procedure with 4-fluorobenzaldehyde (1.86 g, 15 mmol), the crude product was purified by column chromatography (hexane/EtOAc, 5/1), affording pure **S8** as an orange oil (2.04 g, 80%). R_f = 0.28 (hexane/EtOAc, 5/1). **^{1}H NMR** (300 MHz, $CDCl_3$): δ (ppm) = 7.66 (d, *J* = 7.8 Hz, 1H), 7.41–7.29 (m, 4H), 7.27–7.16 (m, 1H), 7.09 (at, *J* = 8.6 Hz, 2H), 6.26 (s, 1H), 5.81 (s, 1H), 3.57 (s, 3H), 3.41 (br s, 1H). **^{13}C NMR** (75.4 MHz, $CDCl_3$): δ (ppm) = 162.3 (d, *J* = 246.1 Hz, C), 141.1 (C), 138.2 (C), 137.0 (d, *J* = 2.9 Hz, C), 128.4 (d, *J* = 8.1 Hz, 2 × CH), 127.0 (C), 122.0 (CH), 120.8 (CH), 119.7 (CH), 115.2 (d, *J* = 21.5 Hz, 2 × CH), 109.2 (CH), 101.6 (CH), 69.1 (CH), 30.1 (CH_3). **HRMS** (ESI-TOF) *m/z*: $[M+H]^+$ Calcd for $C_{16}H_{15}FNO^+$ 256.1132; Found 256.1135.

[5] Shirley, D. A.; Rousell, P. A. *J. Am. Chem. Soc.* **1953**, *75*, 375–378.

Procedure for the synthesis of 2-hydroxy-functionalized indol S10

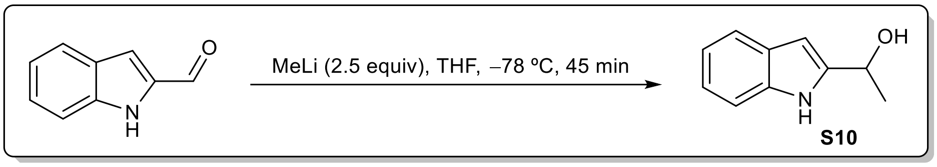

MeLi (1.6 M solution in hexane, 7.8 mL, 12.5 mmol) was slowly added to a solution of 1*H*-indole-2-carbaldehyde (725 mg, 5 mmol) in anhydrous THF (15 mL) at –78 °C under a nitrogen atmosphere. The resulting mixture was stirred at rt for 45 min. The reaction was quenched with aqueous NH_4Cl (10 mL), and the mixture was extracted with Et_2O (3 × 15 mL). The combined organic layers were dried over anhydrous Na_2SO_4, and the solvent was removed under reduced pressure. The residue was purified by silica gel column chromatography using a 3/1 mixture of hexane/EtOAc as eluent to afford pure **S10** as a yellow oil (322 mg, 40%).

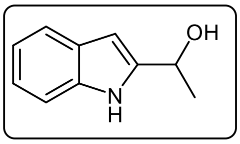

1-(1*H*-Indol-2-yl)ethan-1-ol (S10): R_f = 0.20 (hexane/EtOAc, 3/1).**^{1}H NMR** (300 MHz, $CDCl_3$): *δ* (ppm) = 8.57 (br s, 1H), 7.63 (d, *J* = 7.6 Hz, 1H), 7.31 (d, *J* = 8.0 Hz, 1H), 7.27–7.10 (m, 2H), 6.36 (s, 1H), 5.00 (q, *J* = 6.5 Hz, 1H), 2.79 (br s, 1H), 1.61 (d, *J* = 6.5 Hz, 3H). **^{13}C NMR** (75.4 MHz, $CDCl_3$): *δ* (ppm) = 142.1 (C), 136.0 (C), 128.1 (C), 122.0 (CH), 120.6 (CH), 119.9 (CH), 111.1 (CH), 98.5 (CH), 64.6 (CH), 23.1 (CH_3). **HRMS** (ESI-TOF) *m/z*: could not be recorded.

General procedure for the synthesis of 2-hydroxy-functionalized indoles S11-19

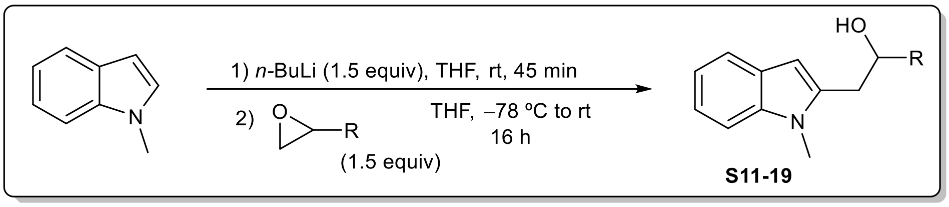

n-BuLi (2.5 M solution in hexane, 3 mL, 7.5 mmol, 1.5 equiv) was slowly added to a solution of 1-methyl-1*H*-indole (655 mg, 5 mmol, 1 equiv) in anhydrous THF (10 mL) at rt under a nitrogen atmosphere. The resulting mixture was stirred at this temperature for 45 min. The corresponding epoxide (7.5 mmol, 1.5 equiv) was added at –78 °C, and the reaction mixture was allowed to warm to rt and stirred overnight. The reaction was quenched with aqueous NH_4Cl (10 mL), and the mixture was extracted with Et_2O (3 × 15 mL). The combined organic layers were dried over anhydrous Na_2SO_4, and the solvent was removed under reduced pressure. The residue was purified by silica gel column chromatography using mixtures of hexane/EtOAc as eluents to afford indoles **S11-19**.

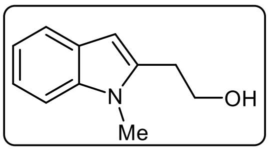

2-(1-Methyl-1*H*-indol-2-yl)ethan-1-ol (S11): Following the general procedure with oxirane (2.5 M in THF, 3 mL, 7.5 mmol), the crude product was purified by column chromatography (hexane/EtOAc, 3/1), affording pure **S11** as a light white solid (656 mg, 75%). R_f = 0.24 (hexane/EtOAc, 3/1). M.p. = 43–45 °C. **^{1}H NMR** (300 MHz, $CDCl_3$): *δ* (ppm) = 7.74 (d, *J* = 7.9 Hz, 1H), 7.44–7.33 (m, 2H), 7.33–7.25 (m, 1H), 6.44 (s, 1H), 3.95 (t, *J* = 6.6 Hz, 2H), 3.69 (s, 3H), 3.03 (t, *J* = 6.6 Hz, 2H), 2.75 (br s, 1H). **^{13}C NMR** (75.4 MHz, $CDCl_3$): *δ* (ppm) = 137.3 (C), 137.3 (C), 127.7 (C), 120.8 (CH), 119.8 (CH), 119.3 (CH), 108.9 (CH), 99.5

(CH), 61.1 (CH_2), 29.9 (CH_2), 29.3 (CH_3). **HRMS** (ESI-TOF) *m/z*: $[M+H]^+$ Calcd for $C_{11}H_{14}NO^+$ 176.1070; Found 176.1076.

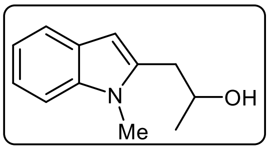

1-(1-Methyl-1*H*-indol-2-yl)propan-2-ol (S12): Following the general procedure with 2-methyloxirane (435 mg, 7.5 mmol), the crude product was purified by column chromatography (hexane/EtOAc, 3/1), affording pure **S12** as a light yellow oil (784 mg, 83%). R_f = 0.15 (hexane/EtOAc, 3/1). **^{1}H NMR** (300 MHz, $CDCl_3$): δ (ppm) = 7.65 (d, J = 7.7 Hz, 1H), 7.37 (d, J = 7.9 Hz, 1H), 7.28 (at, J = 7.5 Hz, 1H), 7.20 (at, J = 6.8 Hz, 1H), 6.42 (s, 1H), 4.22–4.05 (m, 1H), 3.73 (s, 3H), 3.07–2.81 (m, 2H), 2.18 (br s, 1H), 1.37 (d, J = 6.2 Hz, 3H). **^{13}C NMR** (75.4 MHz, $CDCl_3$): δ (ppm) = 137.6 (C), 137.4 (C), 127.8 (C), 121.1 (CH), 120.0 (CH), 119.6 (CH), 109.1 (CH), 100.6 (CH), 66.8 (CH), 36.6 (CH_2), 29.7 (CH_3), 22.9 (CH_3). **HRMS** (ESI-TOF) *m/z*: $[M+H]^+$ Calcd for $C_{12}H_{16}NO^+$ 190.1226; Found 190.1233.

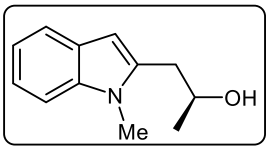

(*S*)-1-(1-Methyl-1*H*-indol-2-yl)propan-2-ol ((*S*)-S12): Following the general procedure, but using 1-methyl-1*H*-indole (131 mg, 1 mmol), *n*-BuLi (0.6 mL, 1.5 mmol) and (*S*)-2-methyloxirane (87 mg, 1.5 mmol), the crude product was purified by column chromatography (hexane/EtOAc, 3/1) affording pure **(*S*)-S12** as a light yellow oil (164 mg, 87%). R_f = 0.15 (hexane/EtOAc, 3/1). **^{1}H NMR** (300 MHz, $CDCl_3$): δ (ppm) = 7.65 (d, J = 7.7 Hz, 1H), 7.37 (d, J = 7.9 Hz, 1H), 7.28 (at, J = 7.5 Hz, 1H), 7.20 (at, J = 6.8 Hz, 1H), 6.42 (s, 1H), 4.22–4.05 (m, 1H), 3.73 (s, 3H), 3.07–2.81 (m, 2H), 2.18 (br s, 1H), 1.37 (d, J = 6.2 Hz, 3H). **^{13}C NMR** (75.4 MHz, $CDCl_3$): δ (ppm) = 137.6 (C), 137.4 (C), 127.8 (C), 121.1 (CH), 120.0 (CH), 119.6 (CH), 109.1 (CH), 100.6 (CH), 66.8 (CH), 36.6 (CH_2), 29.7 (CH_3), 22.9 (CH_3). **HRMS** (ESI-TOF) *m/z*: $[M+H]^+$ Calcd for $C_{12}H_{16}NO^+$ 190.1226; Found 190.1232. $[\alpha]_D^{25}$ = –38.9 (c 0.01534 g/mL in acetone).

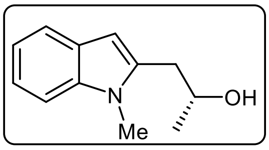

(*R*)-1-(1-Methyl-1*H*-indol-2-yl)propan-2-ol ((*R*)-S12): Following the general procedure, but using 1-methyl-1*H*-indole (131 mg, 1 mmol), *n*-BuLi (0.6 mL, 1.5 mmol) and (*R*)-2-methyloxirane (87 mg, 1.5 mmol), the crude product was purified by column chromatography (hexane/EtOAc, 3/1) affording pure **(*R*)-S12** as a light yellow oil (166 mg, 88%). R_f = 0.15 (hexane/EtOAc, 3/1). **^{1}H NMR** (300 MHz, $CDCl_3$): δ (ppm) = 7.65 (d, J = 7.7 Hz, 1H), 7.37 (d, J = 7.9 Hz, 1H), 7.28 (at, J = 7.5 Hz, 1H), 7.20 (at, J = 6.8 Hz, 1H), 6.42 (s, 1H), 4.22–4.05 (m, 1H), 3.73 (s, 3H), 3.07–2.81 (m, 2H), 2.18 (br s, 1H), 1.37 (d, J = 6.2 Hz, 3H). **^{13}C NMR** (75.4 MHz, $CDCl_3$): δ (ppm) = 137.6 (C), 137.4 (C), 127.8 (C), 121.1 (CH), 120.0 (CH), 119.6 (CH), 109.1 (CH), 100.6 (CH), 66.8 (CH), 36.6 (CH_2), 29.7 (CH_3), 22.9 (CH_3). **HRMS** (ESI-TOF) *m/z*: $[M+H]^+$ Calcd for $C_{12}H_{16}NO^+$ 190.1226; Found 190.1229. $[\alpha]_D^{25}$ = 40.2 (c 0.0057 g/mL in acetone).

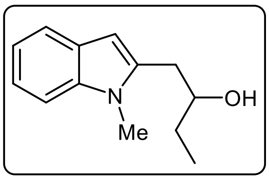

1-(1-Methyl-1*H*-indol-2-yl)butan-2-ol (S13): Following the general procedure with 2-ethyloxirane (540 mg, 7.5 mmol), the crude product was purified by column chromatography (hexane/EtOAc, 3/1), affording pure **S13** as a light yellow oil (862 mg, 85%). R_f = 0.21 (hexane/EtOAc, 3/1). **^{1}H NMR** (300 MHz, $CDCl_3$): δ (ppm) = 7.76 (d, J = 7.7 Hz, 1H), 7.46–7.36 (m, 2H), 7.36–7.28 (m, 1H), 6.50 (s, 1H), 3.99–3.82 (m, 1H), 3.73 (s, 3H), 3.03 (dd, J = 15.0, 4.4 Hz, 1H), 2.90 (dd, J = 15.0, 8.2 Hz, 1H), 2.46 (br s, 1H), 1.84–1.62 (m, 2H), 1.19 (t, J = 7.4 Hz, 3H). **^{13}C NMR** (75.4 MHz, $CDCl_3$): δ (ppm) = 137.5 (C), 137.4 (C), 127.7 (C), 120.7 (CH), 119.7 (CH), 119.3 (CH), 108.9 (CH), 100.3 (CH), 71.9 (CH), 34.3 (CH_2), 29.5 (CH_3), 29.4 (CH_2), 9.9 (CH_3). **HRMS** (ESI-TOF) *m/z*: $[M+H]^+$ Calcd for $C_{13}H_{18}NO^+$ 204.1383; Found 204.1386.

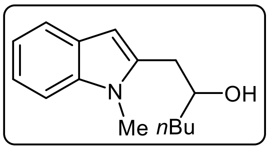

1-(1-Methyl-1*H*-indol-2-yl)hexan-2-ol (S14): Following the general procedure with 2-butyloxirane (750 mg, 7.5 mmol), the crude product was purified by column chromatography (hexane/EtOAc, 3/1), affording pure **S14** as a light orange oil (1.02 g, 88%). R_f = 0.28 (hexane/EtOAc, 3/1). **^{1}H NMR** (300 MHz, $CDCl_3$): δ (ppm) = 7.60 (d, *J* = 7.0 Hz, 1H), 7.32 (d, *J* = 8.1 Hz, 1H), 7.23 (at, *J* = 7.5 Hz, 1H), 7.13 a(t, *J* = 7.8 Hz, 1H), 6.38 (s, 1H), 3.97–3.85 (m, 1H), 3.71 (s, 3H), 3.00 (dd, *J* = 15.0, 4.0 Hz, 1H), 2.84 (dd, *J* = 15.0, 8.5 Hz, 1H), 1.95 (br s, 1H), 1.70–1.54 (m, 3H), 1.52–1.34 (m, 3H), 0.98 (t, *J* = 6.9 Hz, 3H). **^{13}C NMR** (75.4 MHz, $CDCl_3$): δ (ppm) = 137.7 (C), 137.5 (C), 127.9 (C), 121.1 (CH), 120.0 (CH), 120.0 (CH), 109.1 (CH), 100.7 (CH), 70.7 (CH), 36.7 (CH_2), 35.1 (CH_2), 29.8 (CH_3), 28.0 (CH_2), 22.8 (CH_2), 14.2 (CH_3). **HRMS** (ESI-TOF) *m/z*: $[M+H]^+$ Calcd for $C_{15}H_{22}NO^+$ 232.1696; Found 232.1701.

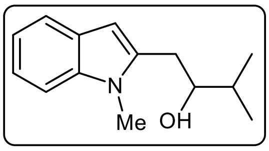

3-Methyl-1-(1-methyl-1*H*-indol-2-yl)butan-2-ol (S15): Following the general procedure with 2-isopropyloxirane (645 mg, 7.5 mmol), the crude product was purified by column chromatography (hexane/EtOAc, 3/1), affording pure **S15** as a light orange solid (835 mg, 77%). R_f = 0.26 (hexane/EtOAc, 3/1). M.p. = 45–47 °C. **^{1}H NMR** (500 MHz, $CDCl_3$): δ (ppm) = 7.73 (d, *J* = 7.8 Hz, 1H), 7.41 (d, *J* = 8.1 Hz, 1H), 7.36 (at, *J* = 7.6 Hz, 1H), 7.28 (at, *J* = 7.9 Hz, 1H), 3.73 (s, 3H), 3.75–3.69 (m, 1H), 3.05 (dd, *J* = 15.0, 3.0 Hz, 1H), 2.85 (dd, *J* = 15.0, 9.5 Hz, 1H), 2.20 (br s, 1H), 2.03–1.86 (m, *J* = 6.6, 1.3 Hz, 1H), 1.18 (d, *J* = 7.0 Hz, 6H). **^{13}C NMR** (125.7 MHz, $CDCl_3$): δ (ppm) = 137.9 (C), 137.5 (C), 127.8 (C), 120.8 (CH), 119.8 (CH), 119.4 (CH), 109.0 (CH), 100.3 (CH), 75.1 (CH), 33.1 (CH_2), 31.7 (CH_3), 29.5 (CH), 18.7 (CH_3), 17.4 (2 × CH_3). **HRMS** (ESI-TOF) *m/z*: $[M+H]^+$ Calcd for $C_{14}H_{20}NO^+$ 218.1539; Found 218.1545.

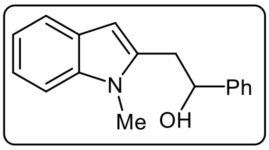

2-(1-Methyl-1*H*-indol-2-yl)-1-phenylethan-1-ol (S16): Following the general procedure with 2-pheniloxirane (900 mg, 7.5 mmol), the crude product was purified by column chromatography (hexane/EtOAc, 3/1), affording pure **S16** as a light yellow oil (840 mg, 67%). R_f = 0.25 (hexane/EtOAc, 3/1). The spectroscopic data of this compound match those reported in the literature.[6]

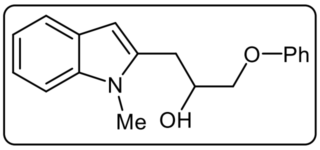

1-(1-Methyl-1*H*-indol-2-yl)-3-phenoxypropan-2-ol (S17): Following the general procedure with 2-(phenoxymethyl)oxirane (1.13 g, 7.5 mmol), the crude product was purified by column chromatography (hexane/EtOAc, 3/1) affording pure **S17** as a light orange solid (927 mg, 66%). R_f = 0.23 (hexane/EtOAc, 3/1). M.p. = 105–107 °C. **^{1}H NMR** (300 MHz, $CDCl_3$): δ (ppm) = 7.70 (d, *J* = 7.8 Hz, 1H), 7.50–7.36 (m, 3H), 7.36–7.29 (m, 1H), 7.29–7.19 (m, 1H), 7.18–7.07 (m, 2H), 7.03 (d, *J* = 8.4 Hz, 2H), 6.49 (s, 1H), 4.45–4.31 (m, 1H), 4.18–3.97 (m, 2H), 3.76 (s, 3H), 3.20 (qd, *J* = 15.1, 6.5 Hz, 2H), 2.92 (br s, 1H). **^{13}C NMR** (75.4 MHz, $CDCl_3$): δ (ppm) = 158.4 (C), 137.5 (C), 136.4 (C), 129.7 (C), 129.6 (2 × CH), 127.8 (C), 121.2 (CH), 121.1 (CH), 120.0 (CH), 119.5 (CH), 116.5 (C), 114.6 (2 × CH), 109.1 (CH), 100.8 (CH), 70.8 (CH), 69.3 (CH_2), 31.0 (CH_3), 29.6 (CH_2). **HRMS** (ESI-TOF) *m/z*: $[M+H]^+$ Calcd for $C_{18}H_{20}NO_2^+$ 282.1489; Found 282.1487.

[6] Barrios-Rivera, J.; Xu, Y.; Clarkson, G. J.; Wills. M. *Tetrahedron* **2022**, *103*, 132562.

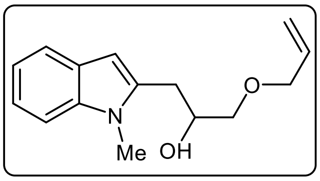

1-(Allyloxy)-3-(1-methyl-1*H*-indol-2-yl)propan-2-ol (S18): Following the general procedure with 2-((allyloxy)methyl)oxirane (855 mg, 7.5 mmol), the crude product was purified by column chromatography (hexane/EtOAc, 3/1), affording pure **S18** as a light orange oil (490 mg, 40%). R_f = 0.18 (hexane/EtOAc, 3/1). **^{1}H NMR** (300 MHz, $CDCl_3$): δ (ppm) = 7.72 (d, *J* = 7.7 Hz, 1H), 7.44–7.31 (m, 2H), 7.27 (at, *J* = 7.2 Hz, 1H), 6.48 (s, 1H), 6.17–5.99 (m, 1H), 5.45 (dd, *J* = 17.2, 1.5 Hz, 1H), 5.36 (dd, *J* = 10.4, 1.5 Hz, 1H), 4.27–4.18 (m, 1H), 4.14 (d, *J* = 5.6 Hz, 2H), 3.75 (s, 3H), 3.66–3.47 (m, 2H), 3.18–2.99 (m, 3H). **^{13}C NMR** (75.4 MHz, $CDCl_3$): δ (ppm) = 137.3 (C), 136.8 (C), 134.4 (CH), 127.7 (C), 120.7 (CH), 119.7 (CH), 119.3 (CH), 117.1 (CH_2), 108.9 (CH), 100.4 (CH), 73.3 (CH_2), 72.1 (CH_2), 69.6 (CH), 30.8 (CH_3), 29.4 (CH_2). **HRMS** (ESI-TOF) *m/z*: $[M+H]^+$ Calcd for $C_{15}H_{20}NO_2^+$ 246.1489; Found 246.1490.

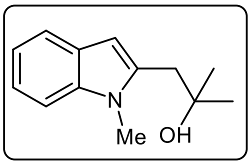

2-Methyl-1-(1-methyl-1*H*-indol-2-yl)propan-2-ol (S19): Following the general procedure with 2,2-dimethyloxirane (540 mg, 7.5 mmol), the crude product was purified by column chromatography (hexane/EtOAc, 3/1), affording pure **S19** as a light orange oil (832 mg, 82%). R_f = 0.25 (hexane/EtOAc, 3/1). **^{1}H NMR** (300 MHz, $CDCl_3$): δ (ppm) = 7.65 (d, *J* = 7.7 Hz, 1H), 7.36 (d, *J* = 8.1 Hz, 1H), 7.27 (at, *J* = 7.1 Hz, 1H), 7.18 (at, *J* = 7.3 Hz, 1H), 6.43 (s, 1H), 3.75 (s, 3H), 2.98 (s, 2H), 1.86 (br s, 1H), 1.34 (s, 6H). **^{13}C NMR** (75.4 MHz, $CDCl_3$): δ (ppm) = 137.6 (C), 137.0 (C), 127.8 (C), 121.0 (CH), 120.0 (CH), 119.6 (CH), 109.3 (CH), 101.8 (CH), 70.7 (C), 40.0 (CH_2), 30.2 (CH_3), 29.3 (2 × CH_3). **HRMS** (ESI-TOF) *m/z*: $[M+H]^+$ Calcd for $C_{13}H_{18}NO^+$ 204.1383; Found 204.1388.

Procedure for the synthesis of 2-hydroxy-functionalized indol S20

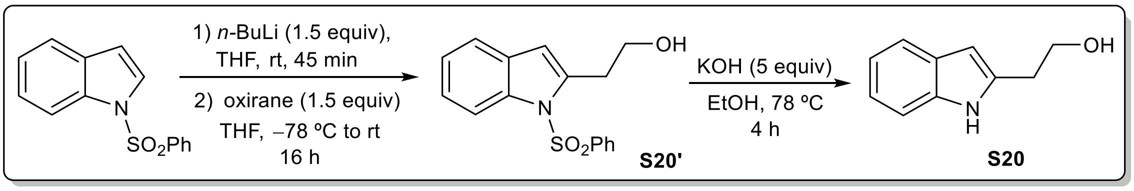

n-BuLi (2.5 M solution in hexane, 6 mL, 15 mmol, 1.5 equiv) was slowly added to a solution of 1-(phenylsulfonyl)-1*H*-indole (2.57 g, 10 mmol, 1 equiv) in anhydrous THF (15 mL) at rt under a nitrogen atmosphere. The resulting mixture was stirred at this temperature for 45 min. Oxirane (2.5 M in THF, 6 mL, 15 mmol, 1.5 equiv) was added at –78 °C, and the reaction mixture was allowed to warm to rt and stirred overnight. The reaction was quenched with aqueous NH_4Cl (10 mL), and the mixture was extracted with Et_2O (3 × 15 mL). The combined organic layers were dried over anhydrous Na_2SO_4, and the solvent was removed under reduced pressure. The residue was purified by silica gel column chromatography using a 3/1 mixture of hexane/EtOAc as eluent to afford pure **S20'** as a light yellow solid (1.89 g, 63%).

KOH (1.47 g, 30 mmol, 5 equiv) was added to a solution of 2-(1-(phenylsulfonyl)-1*H*-indol-2-yl)ethan-1-ol (**S20'**, 1.8 g, 6 mmol, 1 equiv) in EtOH (20 mL) and the mixture was stirred at reflux for 4 h. The reaction was quenched with H_2O (10 mL), and the mixture was extracted with Et_2O (3 × 15 mL). The combined organic layers were dried over anhydrous Na_2SO_4, and the solvent was removed under reduced pressure. The residue was purified by silica gel column chromatography using a 3/1 mixture of hexane and EtOAc as eluent to afford pure **S20** as a light orange solid (715 mg, 74%).

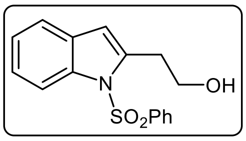

2-(1-(Phenylsulfonyl)-1*H*-indol-2-yl)ethan-1-ol (S20'): R_f = 0.26 (hexane/EtOAc, 3/1). The spectroscopic data for this compound match those reported in the literature.[7]

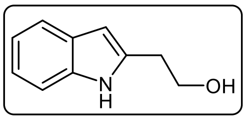

2-(1*H*-Indol-2-yl)ethan-1-ol (S20): R_f = 0.15 (hexane/EtOAc, 2/1). The spectroscopic data for this compound match those reported in the literature.[8]

Procedure for the synthesis of 2-hydroxy-functionalized indoles S21[9]

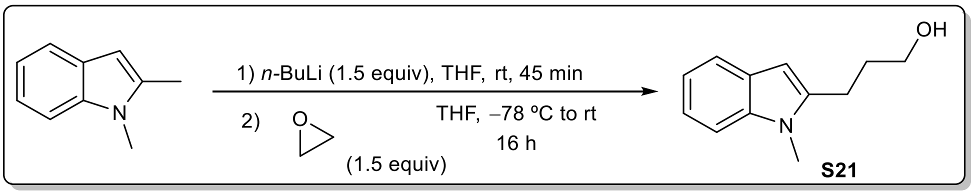

n-BuLi (2.5 M solution in hexane, 3 mL, 7.5 mmol) was slowly added to a solution of 1,2-dimethyl-1*H*-indole (725 mg, 5 mmol) in anhydrous THF (10 mL) at rt under a nitrogen atmosphere. The resulting mixture was stirred at this temperature for 45 min. Oxirane (2.5 M in THF, 3 mL, 7.5 mmol) was added at –78 °C, and the reaction mixture was allowed to warm to rt and stirred overnight. The reaction was quenched with aqueous NH_4Cl (10 mL), and the mixture was extracted with Et_2O (3 × 15 mL). The combined organic layers were dried over anhydrous Na_2SO_4, and the solvent was removed under reduced pressure. The residue was purified by silica gel column chromatography using a 2/1 mixture of hexane/EtOAc as eluent to afford pure **S21** as a yellow oil (680 mg, 72%).

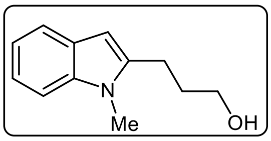

3-(1-Methyl-1*H*-indol-2-yl)propan-1-ol (S21): R_f = 0.20 (hexane/EtOAc, 2/1). The spectroscopic data for this compound match those reported in the literature.[9]

Procedure for the synthesis of 2-hydroxy-functionalized indoles S22[10]

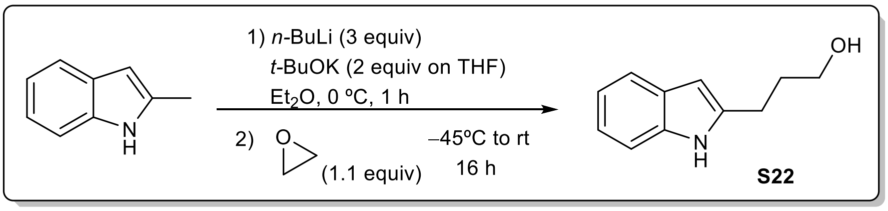

n-BuLi (2.5 M solution in hexane, 6 mL, 15 mmol) was slowly added to a solution of 2-methyl-1*H*-indole (0.65 g, 5 mmol, 1 equiv) in anhydrous Et_2O (10 mL) at 0 °C under a nitrogen atmosphere. To the resulting mixture, *t*-BuOK (1 M solution in THF, 10 mL, 10 mmol) was added, and it was stirred at this temperature for 45 min. Oxirane (2.5 M in THF, 2.2 mL, 5.5 mmol) was added at –45 °C, and the reaction mixture was allowed to warm to rt and stirred overnight. The reaction was quenched with aqueous NH_4Cl (10 mL),

[7] Bergman, J.; Pelcma, B. *Tetrahedron* **1988**, *44*, 16, 5215–5228.
[8] Sripha, K.; Zlotos, D. P.; Buller, S.; Mohr. K. *Tetrahedron* **2003**, *44*, 7183–7186.
[9] Gómez-Gil, S.; Solas, M.; Suárez-Pantiga, S.; Sanz, R. *J. Org. Chem.* **2026**, *91*, 3466–3474.
[10] Inagaki. S.; Nishizawa. Y.; Sugiura. T.; Ishihara. H. *J. Chem. Soc., Perkin Trans. 1* **1990**, 179–180.

and the mixture was extracted with Et_2O (3 × 15 mL). The combined organic layers were dried over anhydrous Na_2SO_4, and the solvent was removed under reduced pressure. The residue was purified by silica gel column chromatography using a 3/1 mixture of hexane/EtOAc as eluent to afford pure **S22** as a yellow oil (1.26 g, 72%). The spectroscopic data for this compound match those reported in the literature.[11]

Synthesis and characterization data of terminal 3-propargylindoles 1, 3, and 9

General procedure for the synthesis of 3-propargylindole derivatives 1, 3, and 9

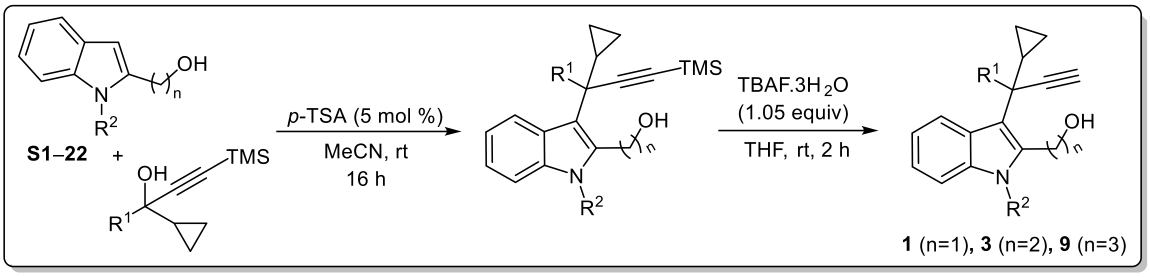

p-TSA (0.065-0.52 mmol, 0.05 equiv) was added to a mixture of the corresponding alkynol (1.3-10.4 mmol, 1.3 equiv) and indole derivative (**S1-22**, 1-8 mmol, 1 equiv) in analytical-grade MeCN (0.5 M). The reaction mixture was stirred at rt until complete consumption of the starting indole, as determined by GC–MS and/or TLC. The crude reaction mixture was neutralized by the addition of 1 M NaOH (5 mL). The mixture was extracted with Et_2O (3 × 15 mL), and the combined organic layers were dried over anhydrous Na_2SO_4. The resulting trimethylsilyl-functionalized indole intermediate was then treated with TBAF·$3H_2O$ (1.05 equiv) in THF at rt for 2 h. The reaction was quenched by the addition of water, and the mixture was extracted with Et_2O (3 × 15 mL). The combined organic layers were dried over anhydrous Na_2SO_4, and the solvent was removed under reduced pressure. The residue was purified by silica gel column chromatography using mixtures of hexane/EtOAc as eluents to afford the corresponding 3-propargylated indole derivatives **1**, **3** and **9**.

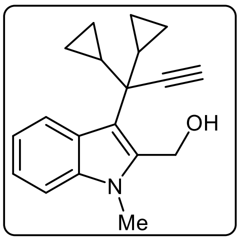

(3-(1,1-Dicyclopropylprop-2-yn-1-yl)-1-methyl-1*H*-indol-2-yl)methanol (1a): Following the general procedure with **S1** (966 mg, 6 mmol) and 1,1-dicyclopropyl-3-(trimethylsilyl)prop-2-yn-1-ol (1.62 g, 7.8 mmol), the crude product was purified by column chromatography (hexane/EtOAc, 3/1), affording pure **1a** as a brown solid (1.08 g, 65%). R_f = 0.29 (hexane/EtOAc, 3/1). M.p. = 65–67 °C. **^{1}H NMR** (300 MHz, $CDCl_3$): δ (ppm) = 8.19 (d, J = 8.2 Hz, 1H), 7.38 (d, J = 8.1 Hz, 1H), 7.36–7.24 (m, 1H), 7.24–7.09 (m, 1H), 5.18 (s, 2H), 3.84 (s, 3H), 2.40 (s, 1H), 2.17 (br s, 1H), 1.97–1.65 (m, 2H), 0.97–0.82 (m, 2H), 0.82–0.64 (m, 3H), 0.64–0.39 (m, 4H). **^{13}C NMR** (75.4 MHz, $CDCl_3$): δ (ppm) = 136.9 (C), 135.9 (C), 126.0 (C), 122.3 (CH), 121.8 (CH), 119.1 (CH), 115.4 (C), 109.4 (CH), 86.1 (C), 71.5 (CH), 54.7 (CH_2), 42.9 (C), 29.5 (CH_3), 21.2 (2 × CH), 3.7 (2 × CH_2), 2.4 (2 × CH_2). **HRMS** (ESI-TOF) *m/z*: $[M+H]^+$ Calcd for $C_{19}H_{22}NO^+$ 280.1696; Found 280.1698.

---

[11] Gao, Y.; Li, J.; Bai, S.; Tu. D.; Yang, C.; Ye, Z.; Hu, B.; Qi, X.; Jiang, C. *Org. Chem. Front.* **2020**, *7*, 1149–1157.

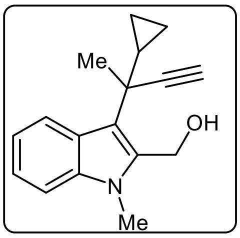

(3-(2-Cyclopropylbut-3-yn-2-yl)-1-methyl-1*H*-indol-2-yl)methanol (1b): Following the general procedure with **S1** (374 mg, 2 mmol) and 2-cyclopropyl-4-(trimethylsilyl)but-3-yn-2-ol (541 mg, 2.6 mmol), the crude product was purified by column chromatography (hexane/EtOAc, 7/1), affording pure **1b** as a yellow oil (263 mg , 52%). R_f = 0.28 (hexane/EtOAc, 3/1). **^{1}H NMR** (300 MHz, $CDCl_3$): δ (ppm) = 8.04 (d, J = 8.2 Hz, 1H), 7.36 (d, J = 8.2 Hz, 1H), 7.28 (at, J = 7.6 Hz, 1H), 7.13 (at, J = 7.5 Hz, 1H), 5.23 (d, J = 13.5 Hz, 1H), 5.11 (d, J = 13.5 Hz, 1H), 3.83 (s, 3H), 2.39 (br s, 1H), 2.06 (s, 1H), 1.97 (s, 3H), 1.75–1.55 (m, 1H), 0.81–0.72 (m, 1H), 0.69–0.51 (m, 3H).**^{13}C NMR** (75.4 MHz, $CDCl_3$): δ (ppm) = 137.1 (C), 135.2 (C), 125.5 (C), 121.9 (2 × CH), 119.2 (CH), 116.8 (C), 109.6 (CH), 89.3 (C), 70.4 (CH), 54.6 (CH_2), 37.8 (C), 31.3 (CH_3), 29.5 (CH_3), 21.7 (CH), 3.7 (CH_2), 3.3 (CH_2). **HRMS** (ESI-TOF) *m/z*: could not be recorded.

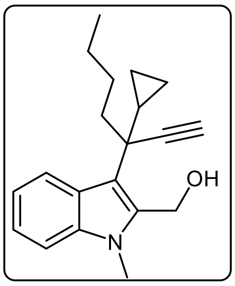

(3-(3-Cyclopropylhept-1-yn-3-yl)-1-methyl-1*H*-indol-2-yl)methanol (1c): Following the general procedure with **S1** (450 mg, 2.8 mmol) and 1,1-dicyclopropyl-3-(trimethylsilyl)prop-2-yn-1-ol (757 mg, 3.64 mmol), the crude product was purified by column chromatography (hexane/EtOAc, 3/1), affording pure **1c** as a brown oil (322 mg , 39%). R_f = 0.30 (hexane/EtOAc, 3/1). **^{1}H NMR** (300 MHz, $CDCl_3$): δ (ppm) = 7.97 (d, J = 8.2 Hz, 1H), 7.36 (d, J = 8.2 Hz, 1H), 7.27 (at, J = 7.6 Hz, 1H), 7.11 (at, J = 7.5 Hz, 1H), 5.21 (d, J = 13.6 Hz, 1H), 5.14 (d, J = 13.6 Hz, 1H), 3.83 (s, 3H), 2.50–2.38 (m, 1H), 2.40 (s, 1H), 2.15–1.97 (m, 1H), 1.72 (tt, J = 8.1, 5.3 Hz, 1H), 1.63–1.44 (m, 1H), 1.44–1.20 (m, 3H), 0.89 (t, J = 7.1 Hz, 3H), 0.83–0.74 (m, 1H), 0.69–0.57 (m, 1H), 0.57–0.40 (m, 2H).**^{13}C NMR** (75.4 MHz, $CDCl_3$): δ (ppm) = 137.1 (C), 135.9 (C), 125.6 (C), 121.9 (CH), 121.8 (CH), 119.1 (CH), 115.4 (C), 109.6 (CH), 87.7 (C), 71.5 (CH), 54.4 (CH_2), 43.2 (CH_2), 42.9 (C), 29.6 (CH_3), 27.8 (CH_2), 23.1 (CH_2), 20.3 (CH), 14.1 (CH_3), 3.6 (CH_2), 2.7 (CH_2). **HRMS** (ESI-TOF) *m/z*: $[M+H]^+$ Calcd for $C_{20}H_{26}NO^+$ 296.2009; Found 296.2010.

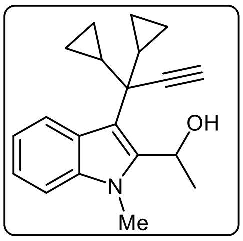

1-(3-(1,1-Dicyclopropylprop-2-yn-1-yl)-1-methyl-1*H*-indol-2-yl)ethan-1-ol (1d): Following the general procedure with **S2** (443 mg, 2.5 mmol) and 1,1-dicyclopropyl-3-(trimethylsilyl)prop-2-yn-1-ol (700 mg, 3.3 mmol), the crude product was purified by column chromatography (hexane/EtOAc, 3/1) affording pure **1d** as an orange oil (498 mg, 68%). R_f = 0.23 (hexane/EtOAc, 3/1). **^{1}H NMR** (300 MHz, $CDCl_3$): δ (ppm) = 8.26 (d, J = 8.3 Hz, 1H), 7.40 (d, J = 7.9 Hz, 1H), 7.38–7.29 (m, 1H), 7.24–7.13 (m, 1H), 6.42 (q, J = 6.8 Hz, 1H), 4.04 (s, 3H), 2.41 (br s, 1H), 2.36 (s, 1H), 1.78 (d, J = 6.8 Hz, 3H), 1.85–1.68 (m, 2H), 1.02–0.93 (m, 1H), 0.93–0.83 (m, 1H), 0.81–0.56 (m, 6H).**^{13}C NMR** (75.4 MHz, $CDCl_3$): δ (ppm) = 138.8 (C), 137.7 (C), 125.8 (C), 122.2 (CH), 121.4 (CH), 119.0 (CH), 114.2 (C), 109.0 (CH), 85.1 (C), 71.9 (CHC), 62.8 (CH), 42.7 (C), 31.7 (CH_3), 21.8 (CH_3), 21.6 (CH), 21.4 (CH), 4.1 (CH_2), 3.6 (CH_2), 2.8 (CH_2), 2.7 (CH_2). **HRMS** (ESI-TOF) *m/z*: $[M+H]^+$ Calcd for $C_{20}H_{24}NO^+$ 294.1852; Found 294.1859.

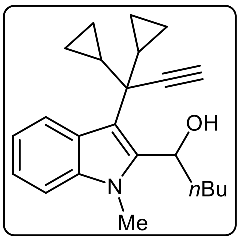

1-(3-(1,1-Dicyclopropylprop-2-yn-1-yl)-1-methyl-1*H*-indol-2-yl)pentan-1-ol (1e): Following the general procedure with **S3** (1.08 g, 5 mmol) and 1,1-dicyclopropyl-3-(trimethylsilyl)prop-2-yn-1-ol (1.35 g, 6.5 mmol), the crude product was purified by column chromatography (hexane/EtOAc, 3/1) affording pure **1e** as an orange oil (1.31 g, 78%). R_f = 0.26 (hexane/EtOAc, 3/1). **^{1}H NMR** (300 MHz, $CDCl_3$): δ (ppm) = 8.21 (d, J = 8.2 Hz, 1H), 7.38 (d, J = 8.1 Hz, 1H),

7.34–7.22 (m, 1H), 7.19–7.08 (m, 1H), 6.19 (dd, *J* = 9.9, 4.1 Hz, 1H), 4.01 (s, 3H), 2.35 (s, 1H), 2.28–2.12 (m, 2H), 2.00–1.87 (m, 1H), 1.83–1.67 (m, 3H), 1.58–1.42 (m, 3H), 1.03 (t, *J* = 7.1 Hz, 3H), 0.98–0.83 (m, 2H), 0.80–0.64 (m, 3H), 0.64–0.50 (m, 2H). **^{13}C NMR** (75.4 MHz, $CDCl_3$): *δ* (ppm) = 138.6 (C), 137.8 (C), 125.9 (C), 122.3 (CH), 121.4 (CH), 119.0 (CH), 114.9 (C), 109.1 (CH), 85.0 (C), 72.2 (CH), 67.0 (CH), 42.9 (C), 35.6 (CH_2), 32.0 (CH_3), 29.2 (CH_2), 22.8 (CH_2), 21.6 (CH), 21.5 (CH), 14.2 (CH_3), 4.1 (CH_2), 3.7 (CH_2), 2.9 (CH_2), 2.8 (CH_2). **HRMS** (ESI-TOF) *m/z*: $[M+H]^+$ Calcd for $C_{23}H_{30}NO^+$ 336.2322; Found 336.2324.

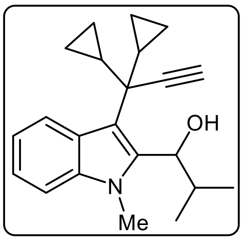

1-(3-(1,1-Dicyclopropylprop-2-yn-1-yl)-1-methyl-1*H*-indol-2-yl)-2-methylpropan-1-ol (1f): Following the general procedure with **S4** (600 mg, 3 mmol) and 1,1-dicyclopropyl-3-(trimethylsilyl)prop-2-yn-1-ol (810 g, 3.8 mmol), the crude product was purified by column chromatography (hexane/EtOAc, 5/1) affording pure **1f** as a yellow solid (618 mg, 64%). R_f = 0.33 (hexane/EtOAc, 5/1). M.p. = 83–85 °C. **^{1}H NMR** (300 MHz, $CDCl_3$): *δ* (ppm) = 8.19 (d, *J* = 7.6 Hz, 1H), 7.36 (d, *J* = 7.8 Hz, 1H), 7.34–7.26 (m, 1H), 7.20–7.10 (m, 1H), 5.90 (d, *J* = 9.9 Hz, 1H), 3.97 (s, 3H), 2.57–2.43 (m, 1H), 2.35 (s, 1H), 2.21 (br s, 1H), 1.84–1.67 (m, 2H), 1.31 (d, *J* = 6.4 Hz, 3H), 0.98–0.85 (m, 2H), 0.81 (d, *J* = 7.0 Hz, 3H), 0.77–0.63 (m, 3H), 0.62–0.44 (m, 3H). **^{13}C NMR** (75.4 MHz, $CDCl_3$): *δ* (ppm) = 137.8 (C), 137.0 (C), 125.7 (C), 122.4 (CH), 121.5 (CH), 118.8 (CH), 117.8 (C), 109.1 (CH), 84.6 (C), 73.4 (CH), 72.0 (CH), 43.1 (C), 33.1 (CH), 32.0 (CH_3), 21.6 (CH_3), 21.4 (CH_3), 21.0 (CH), 19.5 (CH), 4.1 (CH_2), 3.8 (CH_2), 3.5 (CH_2), 2.7 (CH_2). **HRMS** (ESI-TOF) *m/z*: $[M+H]^+$ Calcd for $C_{22}H_{28}NO^+$ 322.2165; Found 322.2174.

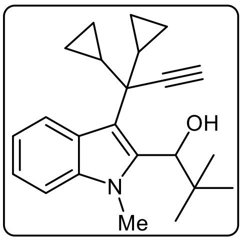

1-(3-(1,1-Dicyclopropylprop-2-yn-1-yl)-1-methyl-1*H*-indol-2-yl)-2,2-dimethylpropan-1-ol (1g): Following the general procedure with **S5** (651 mg, 3 mmol) and 1,1-dicyclopropyl-3-(trimethylsilyl)prop-2-yn-1-ol (811 mg, 3.9 mmol), the crude product was purified by column chromatography (hexane/EtOAc, 5/1) affording pure **1g** as an orange oil (372 mg, 37%). R_f = 0.33 (hexane/EtOAc, 5/1). M.p. = 76–78 °C. **^{1}H NMR** (300 MHz, $CDCl_3$): *δ* (ppm) = 8.20 (d, *J* = 8.2 Hz, 1H), 7.39 (d, *J* = 8.1 Hz, 1H), 7.37–7.29 (m, 1H), 7.21–7.13 (m, 1H), 6.48 (s, 1H), 4.06 (s, 3H), 2.44 (s, 1H), 2.12 (br s, 1H), 2.01–1.92 (m, 1H), 1.90–1.81 (m, 1H), 1.20 (s, 9H), 1.13–1.05 (m, 1H), 0.92–0.83 (m, 1H), 0.83–0.70 (m, 3H), 0.69–0.59 (m, 1H), 0.59–0.49 (m, 2H), 0.41–0.30 (m, 1H). **^{13}C NMR** (75.4 MHz, $CDCl_3$): *δ* (ppm) = 138.9 (C), 135.0 (C), 126.2 (C), 122.5 (CH), 121.5 (CH), 118.7 (CH), 118.0 (C), 109.6 (CH), 83.8 (C), 75.4 (CH), 72.9 (CH), 43.5 (C), 36.9 (C), 34.4 (CH_3), 28.0 (3 × CH_3), 21.12 (CH), 21.10 (CH), 4.3 (CH_2), 3.6 (CH_2), 3.5 (CH_2), 2.2 (CH_2). **HRMS** (ESI-TOF) *m/z*: $[M+H]^+$ Calcd for $C_{23}H_{30}NO^+$ 336.2322; Found 336.2338.

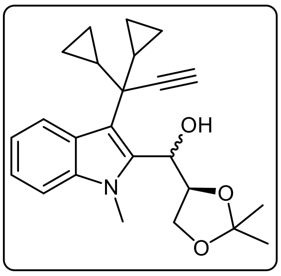

(3-(1,1-Dicyclopropylprop-2-yn-1-yl)-1-methyl-1*H*-indol-2-yl)((*S*)-2,2-dimethyl-1,3-dioxolan-4-yl)methanol (1h): Following the general procedure with **S6** (1.3 g, 5 mmol) and 1,1-dicyclopropyl-3-(trimethylsilyl)prop-2-yn-1-ol (1.35 g, 6.5 mmol), the crude product was purified by column chromatography (hexane/EtOAc, 3/1) affording pure **1h** as a yellow oil (1.14 g , 60%). R_f = 0.20 (hexane/EtOAc, 3/1). Obtained and isolated as a ca. 3/1 mixture of diasteroisomers. Data for major diasteroisomer: **^{1}H NMR** (300 MHz, $CDCl_3$): *δ* (ppm) = 8.21 (d, *J* = 8.2 Hz, 1H), 7.39–7.23 (m, 2H), 7.16 (at, *J* = 7.8 Hz, 1H), 6.34 (d, *J* = 8.2 Hz, 1H), 4.78 (dt, *J* = 8.2, 6.8 Hz, 1H), 4.00 (s, 3H), 3.93 (dd, *J* = 8.6, 6.8 Hz, 1H), 3.82 (dd, *J* = 8.6, 6.8 Hz, 1H), 2.95 (br s, 1H), 2.44 (s, 1H), 1.91–1.76 (m, 2H), 1.60 (s, 3H), 1.48 (s, 3H), 1.08–0.82 (m, 2H), 0.82–

0.38 (m, 6H). **^{13}C NMR** (75.4 MHz, $CDCl_3$): *δ* (ppm) = 138.1 (C), 133.2 (C), 125.8 (C), 122.6 (CH), 122.0 (CH), 119.1 (CH), 118.1 (C), 110.2 (CH), 109.4 (CH), 83.9 (C), 78.5 (CH), 74.1 (CH), 68.0 (CH), 66.0 (CH_2), 43.1 (C), 32.4 (CH_3), 27.3 (CH_3), 25.5 (CH_3), 21.6 (CH), 21.1 (CH), 3.9 (CH_2), 3.8 (CH_2), 3.1 (CH_2), 2.5 (CH_2). **HRMS** (ESI-TOF) *m/z*: $[M+H]^+$ Calcd for $C_{24}H_{30}NO_3^+$ 380.2220; Found 380.2221.

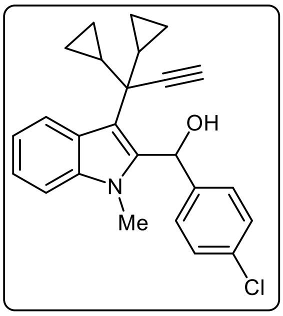

(4-Chlorophenyl)(3-(1,1-dicyclopropylprop-2-yn-1-yl)-1-methyl-1*H*-indol-2-yl)methanol (1i): Following the general procedure with **S7** (1.19 g, 4.4 mmol) and 1,1-dicyclopropyl-3-(trimethylsilyl)prop-2-yn-1-ol (1.22 g, 5.75 mmol), the crude product was purified by column chromatography (hexane/EtOAc, 3/1) affording pure **1i** as an orange oil (1.22 g , 71%). R_f = 0.28 (hexane/EtOAc, 3/1). **^{1}H NMR** (300 MHz, $CDCl_3$): *δ* (ppm) = 8.23 (d, *J* = 8.3 Hz, 1H), 7.40–7.23 (m, 7H), 7.20–7.07 (m, 1H), 3.39 (s, 3H), 2.55 (br s, 1H), 2.28 (s, 1H), 1.87–1.76 (m, 1H), 1.76–1.67 (m, 1H), 1.05–0.88 (m, 1H), 0.90–0.78 (m, 1H), 0.77–0.40 (m, 6H). **^{13}C NMR** (75.4 MHz, $CDCl_3$): *δ* (ppm) = 140.2 (C), 137.7 (C), 136.6 (C), 132.7 (C), 128.4 (2 × CH), 127.4 (2 × CH), 125.4 (C), 122.5 (CH), 122.0 (CH), 119.3 (CH), 118.3 (C), 109.3 (CH), 84.5 (C), 72.7 (CH), 66.5 (CH), 43.1 (C), 31.4 (CH_3), 21.9 (CH), 21.5 (CH), 4.4 (CH_2), 3.6 (CH_2), 3.0 (CH_2), 2.9 (CH_2). **HRMS** (ESI-TOF) *m/z*: $[M+H]^+$ Calcd for $C_{25}H_{25}NClO^+$ 390.1619; Found 390.1623.

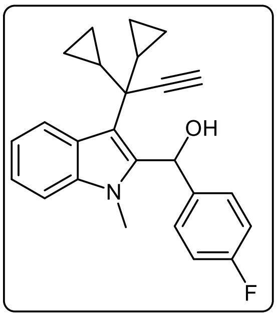

(3-(1,1-Cicyclopropylprop-2-yn-1-yl)-1-methyl-1*H*-indol-2-yl)(4-fluorophenyl)methanol (1j): Following the general procedure with **S8** (765 mg, 3 mmol) and 1,1-dicyclopropyl-3-(trimethylsilyl)prop-2-yn-1-ol (811 mg, 3.9 mmol), the crude product was purified by column chromatography (hexane/EtOAc, 5/1) affording pure **1j** as an orange oil (504 mg , 45%). R_f = 0.30 (hexane/EtOAc, 5/1). **^{1}H NMR** (300 MHz, $CDCl_3$): *δ* (ppm) = 8.33 (d, *J* = 8.2 Hz, 1H), 7.48–7.39 (m, 3H), 7.37–7.31 (m, 2H), 7.26–7.20 (m, 1H), 7.10 (at, *J* = 8.7 Hz, 2H), 3.47 (s, 3H), 2.75 (br s, 1H), 2.34 (s, 1H), 1.95–1.83 (m, 1H), 1.85–1.72 (m, 1H), 1.09–0.89 (m, 2H), 0.88–0.51 (m, 6H). **^{13}C NMR** (75.4 MHz, $CDCl_3$): *δ* (ppm) = 161.8 (C, *J* = 245.0 Hz), 137.7 (C), 137.4 (C, *J* = 3.0 Hz), 136.8 (C), 127.5 (2 × CH, *J* = 7.9 Hz), 125.4 (C), 122.5 (CH), 122.0 (CH), 119.2 (CH), 118.1 (C), 115.0 (2 × CH, *J* = 21.4 Hz), 109.3 (CH), 84.5 (C), 72.7 (CH), 66.5 (CH), 43.1 (C), 31.3 (CH_3), 21.9 (CH), 21.5 (CH), 4.3 (CH_2), 3.6 (CH_2), 3.0 (CH_2), 2.9 (CH_2). **HRMS** (ESI-TOF) *m/z*: $[M+H]^+$ Calcd for $C_{25}H_{25}NFO^+$ 374.1915; Found 374.1911.

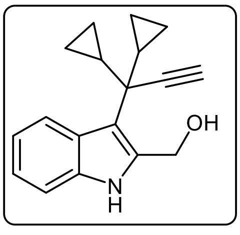

(3-(1,1-Dicyclopropylprop-2-yn-1-yl)-1*H*-indol-2-yl)methanol (1k): Following the general procedure with **S9** (1.18 g, 8 mmol) and 1,1-dicyclopropyl-3-(trimethylsilyl)prop-2-yn-1-ol (2.17 g, 10.5 mmol), the crude product was purified by column chromatography (hexane/EtOAc, 3/1) affording pure **1k** as a yellow solid (1.23 g , 58%). R_f = 0.26 (hexane/EtOAc, 3/1). M.p. = 74–76 °C. **^{1}H NMR** (300 MHz, $CDCl_3$): *δ* (ppm) = 8.66 (br s, 1H), 8.12 (d, *J* = 7.8 Hz, 1H), 7.29 (d, *J* = 7.9 Hz, 1H), 7.25–7.08 (m, 3H), 5.12 (s, 2H), 2.81 (br s, 1H), 2.30 (s, 1H), 1.80–1.55 (m, 2H), 0.88–0.69 (m, 2H), 0.70–0.57 (m, 2H), 0.57–0.30 (m, 4H). **^{13}C NMR** (75.4 MHz, $CDCl_3$): *δ* (ppm) = 135.1 (C), 133.9 (C), 127.5 (C), 122.0 (CH), 121.6 (CH), 119.3 (CH), 113.9 (C), 111.3 (CH), 84.7 (C), 72.0 (CH), 58.2 (CH_2), 42.8 (C), 20.8 (2 × CH), 3.4 (2 × CH_2), 2.4 (2 × CH_2). **HRMS** (ESI-TOF) *m/z*: $[M+H]^+$ Calcd for $C_{18}H_{20}NO^+$ 266.1539; Found 266.1547.

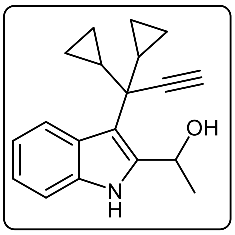

1-(3-(1,1-Dicyclopropylprop-2-yn-1-yl)-1*H*-indol-2-yl)ethan-1-ol (1l): Following the general procedure with **S10** (322 mg, 2 mmol) and 1,1-dicyclopropyl-3-(trimethylsilyl)prop-2-yn-1-ol (540 mg, 2.6 mmol), the crude product was purified by column chromatography (hexane/EtOAc, 3/1) affording pure **1l** as a brown solid (357 mg, 64%). R_f = 0.21 (hexane/EtOAc, 3/1). M. p. = 80–82 °C. ^{1}**H NMR** (300 MHz, $CDCl_3$): δ (ppm) = 8.65 (br s, 1H), 8.10 (d, J = 8.1 Hz, 1H), 7.35 (d, J = 7.9 Hz, 1H), 7.20 (at, J = 7.5 Hz, 1H), 7.11 (at, J = 7.1 Hz, 1H), 6.05 (q, J = 6.4 Hz, 1H), 2.52 (br s, 1H), 2.29 (s, 1H), 1.78–1.62 (m, 2H), 1.63 (d, J = 6.4 Hz, 3H), 0.90–0.74 (m, 2H), 0.73–0.59 (m, 2H), 0.59–0.38 (m, 4H). ^{13}**C NMR** (75.4 MHz, $CDCl_3$): δ (ppm) = 138.1 (C), 135.2 (C), 127.2 (C), 122.2 (CH), 121.7 (CH), 119.2 (CH), 113.4 (C), 111.2 (CH), 84.3 (CH), 72.3 (C), 63.3 (CH), 42.9 (C), 24.1 (CH_3), 21.1 (CH), 20.7 (CH), 3.6 (CH_2), 3.4 (CH_2), 2.6 (CH_2), 2.4 (CH_2). **HRMS** (ESI-TOF) *m/z*: $[M+H]^+$ Calcd for $C_{19}H_{22}NO^+$ 280.1696; Found 280.1695.

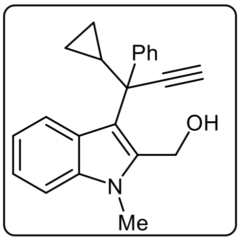

(3-(1-Cyclopropyl-1-phenylprop-2-yn-1-yl)-1-methyl-1*H*-indol-2-yl)methanol (1m): Following the general procedure with **S1** (374 mg, 2 mmol) and 1-cyclopropyl-1-phenyl-3-(trimethylsilyl)prop-2-yn-1-ol (630 mg, 2.6 mmol), the crude product was purified by column chromatography (hexane/EtOAc, 3/1) affording pure **1m** as a yellow solid (485 mg, 77%). R_f = 0.28 (hexane/EtOAc, 3/1). M.p. = 83–85 °C.^{1}**H NMR** (300 MHz, $CDCl_3$): δ (ppm) = 7.89 (d, J = 8.2 Hz, 1H), 7.65–7.58 (m, 2H), 7.44–7.24 (m, 5H), 7.17–7.01 (m, 1H), 4.85 (d, J = 13.7 Hz, 1H), 4.75 (d, J = 13.7 Hz, 1H), 3.83 (s, 3H), 2.59 (s, 1H), 2.03–1.87 (m, 1H), 1.56 (br s, 1H), 1.05–0.93 (m, 1H), 0.92–0.79 (m, 2H), 0.79–0.64 (m, 1H). ^{13}**C NMR** (75.4 MHz, $CDCl_3$): δ (ppm) = 147.7 (C), 136.9 (C), 136.2 (C), 128.3 (2 × CH), 127.1 (2 × CH), 126.8 (CH), 126.0 (C), 121.84 (CH), 121.80 (CH), 119.4 (CH), 117.5 (C), 109.5 (CH), 86.7 (C), 73.4 (CH), 54.9 (CH_2), 47.0 (C), 29.6 (CH_3), 21.9 (CH), 5.5 (CH_2), 4.2 (CH_2). **HRMS** (ESI-TOF) *m/z*: $[M+H]^+$ Calcd for $C_{22}H_{22}NO^+$ 316.1696; Found 316.1697.

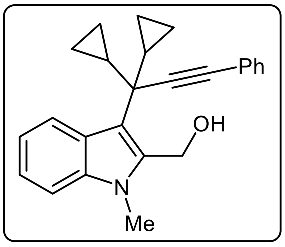

(3-(1,1-Dicyclopropyl-3-phenylprop-2-yn-1-yl)-1-methyl-1*H*-indol-2-yl)methanol (1n): Following the general procedure with **S1** (402 mg, 2.5 mmol) and 1,1-dicyclopropyl-3-phenylprop-2-yn-1-ol (689 mg, 3.25 mmol), the crude product was purified by column chromatography (hexane/EtOAc, 5/1) affording pure **1n** as a yellow solid (622 mg, 66%). R_f = 0.32 (hexane/EtOAc, 5/1). M.p. = 111–113 °C. ^{1}**H NMR** (300 MHz, $CDCl_3$): δ (ppm) = 8.26 (d, J = 8.2 Hz, 1H), 7.63–7.52 (m, 2H), 7.47–7.28 (m, 6H), 7.23–7.14 (m, 1H), 5.28 (d, J = 5.2 Hz, 2H), 3.86 (s, 3H), 2.03 (br s, 1H), 1.97–1.82 (m, 2H), 1.02–0.89 (m, 2H), 0.87–0.71 (m, 2H), 0.71–0.46 (m, 4H). ^{13}**C NMR** (75.4 MHz, $CDCl_3$): δ (ppm) = 137.1 (C), 135.8 (C), 131.6 (2 × CH), 128.5 (2 × CH), 128.2 (CH), 126.1 (C), 123.1 (C), 122.4 (CH), 121.8 (CH), 119.2 (CH), 116.0 (C), 109.5 (CH), 91.2 (C), 83.7 (C), 55.0 (CH_2), 43.6 (C), 29.5 (CH_3), 21.7 (2 × CH), 3.8 (2 × CH_2), 2.5 (2 × CH_2). **HRMS** (ESI-TOF) *m/z*: $[M+Na]^+$ Calcd for $C_{25}H_{25}NaNO^+$ 378.1828; Found 378.1839.

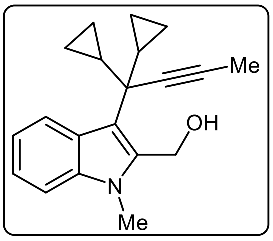

(3-(1,1-Dicyclopropylbut-2-yn-1-yl)-1-methyl-1*H*-indol-2-yl)methanol (1o): Following the general procedure with **S1** (374 mg, 2 mmol) and 1,1-dicyclopropylbut-2-yn-1-ol (390 mg, 2.6 mmol), the crude product was purified by column chromatography (hexane/EtOAc, 3/1) affording pure **1o** as a yellow solid (421 mg, 72%). R_f = 0.28 (hexane/EtOAc, 3/1). M.p. = 65–67 °C. ^{1}**H NMR**

(300 MHz, $CDCl_3$): δ (ppm) = 8.17 (d, J = 8.2 Hz, 1H), 7.37 (d, J = 8.1 Hz, 1H), 7.29 (at, J = 7.5 Hz, 1H), 7.15 (at, J = 7.5 Hz, 1H), 5.17 (s, 2H), 3.83 (s, 3H), 2.07 (br s, 1H), 1.95 (s, 3H), 1.82–1.69 (m, 2H), 0.87–0.75 (m, 2H), 0.73–0.62 (m, 2H), 0.56–0.40 (m, 4H). **^{13}C NMR** (75.4 MHz, $CDCl_3$): δ (ppm) = 134.0 (C), 135.7 (C), 126.1 (C), 122.4 (CH), 121.7 (CH), 119.0 (CH), 116.8 (C), 109.4 (CH), 80.4 (C), 78.7 (C), 54.9 (CH_2), 43.1 (C), 29.4 (CH_3), 21.6 (2 × CH), 3.6 (2 × CH_2), 3.5 (CH_3), 2.3 (2 × CH_2). **HRMS** (ESI-TOF) *m/z*: $[M+H]^+$ Calcd for $C_{20}H_{24}NO^+$ 294.1852; Found 294.1843.

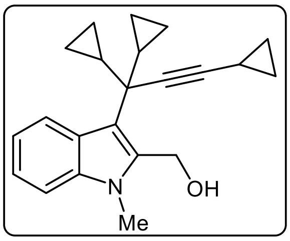

(1-Methyl-3-(1,1,3-tricyclopropylprop-2-yn-1-yl)-1*H*-indol-2-yl)methanol (1p): Following the general procedure with **S1** (805 mg, 5 mmol) and 1,1,3-tricyclopropylprop-2-yn-1-ol (1.14 g, 6.5 mmol), the crude product was purified by column chromatography (hexane/EtOAc, 3/1) affording pure **1p** as an orange oil (1.13 g, 71%). R_f = 0.34 (hexane/EtOAc, 3/1). **^{1}H NMR** (300 MHz, $CDCl_3$): δ (ppm) = 8.11 (d, J = 8.2 Hz, 1H), 7.35 (d, J = 8.2 Hz, 1H), 7.30–7.21 (m, 1H), 7.11 (at, J = 7.5 Hz, 1H), 5.14 (s, 2H), 3.81 (s, 3H), 2.00 (br s, 1H), 1.82–1.62 (m, 2H), 1.43–1.22 (m, 1H), 0.91–0.78 (m, 2H), 0.78–0.66 (m, 4H), 0.66–0.52 (m, 2H), 0.51–0.33 (m, 4H). **^{13}C NMR** (75.4 MHz, $CDCl_3$): δ (ppm) = 137.0 (C), 135.8 (C), 126.0 (C), 122.4 (CH), 121.7 (CH), 119.0 (CH), 116.8 (C), 109.5 (CH), 86.5 (C), 76.4 (C), 54.9 (CH_2), 43.1 (C), 29.5 (CH_3), 21.7 (CH), 8.1 (2 × CH), 3.5 (2 × CH_2), 2.3 (2 × CH_2), -0.5 (2 x CH_2). **HRMS** (ESI-TOF) *m/z*: $[M+H]^+$ Calcd for $C_{22}H_{26}NO^+$ 320.2009; Found 320.2010.

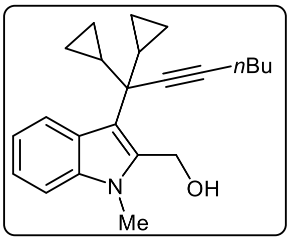

(3-(1,1-Dicyclopropylhept-2-yn-1-yl)-1-methyl-1*H*-indol-2-yl)methanol (1q): Following the general procedure with **S1** (805 mg, 5 mmol) and 1,1-dicyclopropylhept-2-yn-1-ol (1.25 g, 6.5 mmol), the crude product was purified by column chromatography (hexane/EtOAc, 3/1) affording pure **1q** as a yellow oil (722 mg, 43%). R_f = 0.36 (hexane/EtOAc, 3/1). **^{1}H NMR** (300 MHz, $CDCl_3$): δ (ppm) = 8.17 (d, J = 8.2, 1H), 7.35 (d, J = 8.2 Hz, 1H), 7.33–7.20 (m, 1H), 7.20–7.05 (m, 1H), 5.17 (s, 2H), 3.81 (s, 3H), 2.42–2.19 (m, 2H), 2.06 (br s, 1H), 1.86–1.69 (m, 2H), 1.69–1.40 (m, 4H), 1.06–0.95 (m, 3H), 0.88–0.76 (m, 2H), 0.76–0.61 (m, 2H), 0.61–0.42 (m, 4H). **^{13}C NMR** (75.4 MHz, $CDCl_3$): δ (ppm) = 137.0 (C), 135.7 (C), 126.0 (C), 122.4 (CH), 121.6 (CH), 118.9 (CH), 117.0 (C), 109.4 (CH), 83.6 (C), 80.9 (C), 54.8 (CH_2), 43.2 (C), 31.1 (CH_2), 29.4 (CH_3), 22.2 (CH_2), 21.7 (2 × CH), 18.5 (CH_2), 13.7 (CH_3), 3.5 (2 × CH_2), 2.3 (2 × CH_2). One aromatic C was not observed due to overlapping. **HRMS** (ESI-TOF) *m/z*: $[M+H]^+$ Calcd for $C_{23}H_{30}NO^+$ 336.2322; Found 336.2317.

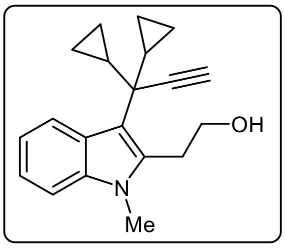

2-(3-(1,1-Dicyclopropylprop-2-yn-1-yl)-1-methyl-1*H*-indol-2-yl)ethan-1-ol (3a): Following the general procedure with **S10** (580 mg, 3.3 mmol) and 1,1-dicyclopropyl-3-(trimethylsilyl)prop-2-yn-1-ol (892 mg, 4.3 mmol), the crude product was purified by column chromatography (hexane/EtOAc, 2/1) affording pure **3a** as an orange solid (802 mg, 83%). R_f = 0.25 (hexane/EtOAc, 2/1). M.p. = 56–58 °C. **^{1}H NMR** (300 MHz, $CDCl_3$): δ (ppm) = 8.19 (d, J = 8.1 Hz, 1H), 7.36 (d, J = 8.1 Hz, 1H), 7.33–7.21 (m, 1H), 7.21–7.13 (m, 1H), 3.94 (t, J = 7.3 Hz, 2H), 3.76 (s, 3H), 3.55 (t, J = 7.3 Hz, 2H), 2.39 (s, 1H), 2.19 (br s, 1H), 1.93–1.72 (m, 2H), 1.00–0.84 (m, 2H), 0.78–0.44 (m, 6H). **^{13}C NMR** (75.4 MHz, $CDCl_3$): δ (ppm) = 136.9 (C), 133.2 (C), 126.5 (C), 121.7 (CH), 120.9 (CH), 118.8 (CH), 114.5 (C), 109.1 (CH), 84.1 (C), 72.7 (CH), 63.0 (CH_2), 43.1 (C), 29.7 (CH_3), 28.7 (CH_2), 20.9 (2 × CH), 3.5 (2 × CH_2), 2.5 (2 × CH_2). **HRMS** (ESI-TOF) *m/z*: $[M+H]^+$ Calcd for $C_{20}H_{24}NO^+$ 294.1852; Found 294.1863.

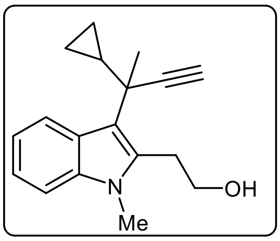

2-(3-(2-Cyclopropylbut-3-yn-2-yl)-1-methyl-1*H*-indol-2-yl)ethan-1-ol (3b): Following the general procedure with **S10** (175 mg, 1 mmol) and 2-cyclopropyl-4-(trimethylsilyl)but-3-yn-2-ol (236 mg, 1.3 mmol), the crude product was purified by column chromatography (hexane/EtOAc, 2/1) affording pure **3b** as a yellow oil (190 mg, 71%). R_f = 0.20 (hexane/EtOAc, 2/1). **^{1}H NMR** (300 MHz, $CDCl_3$): δ (ppm) = 8.03 (d, J = 8.1 Hz, 1H), 7.32 (d, J = 8.1 Hz, 1H), 7.23 (at, J = 7.1 Hz, 1H), 7.12 (at, J = 7.0 Hz, 1H), 3.92 (t, J = 7.2 Hz, 2H), 3.74 (s, 3H), 3.63–3.43 (m, 2H), 2.35 (s, 1H), 1.93 (s, 3H), 1.83 (br s, 1H), 1.75–1.62 (m, 1H), 0.85–0.75 (m, 1H), 0.75–0.50 (m, 3H). **^{13}C NMR** (75.4 MHz, $CDCl_3$): δ (ppm) = 137.1 (C), 132.6 (C), 126.1 (C), 121.4 (CH), 121.1 (CH), 118.9 (CH), 115.4 (C), 109.2 (CH), 87.9 (C), 71.3 (CH), 63.0 (CH_2), 38.0 (C), 31.1 (CH_3), 29.8 (CH_3), 28.6 (CH_2), 21.1 (CH), 4.0 (CH_2), 3.1 (CH_2). **HRMS** (ESI-TOF) *m/z*: $[M+H]^+$ Calcd for $C_{18}H_{22}NO^+$ 268.1696; Found 268.1704.

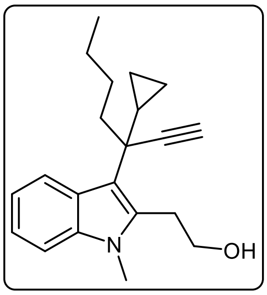

2-(3-(3-Cyclopropylhept-1-yn-3-yl)-1-methyl-1*H*-indol-2-yl)ethan-1-ol (3c): Following the general procedure with **S10** (525 mg, 3 mmol) and 3-cyclopropyl-1-(trimethylsilyl)hept-1-yn-3-ol (873 mg, 3.9 mmol), the crude product was purified by column chromatography (hexane/EtOAc, 3/1) affording pure **3c** as an orange oil (556 mg, 60%). R_f = 0.25 (hexane/EtOAc, 3/1). **^{1}H NMR** (300 MHz, $CDCl_3$): δ (ppm) = 7.94 (d, J = 8.1 Hz, 1H), 7.32 (d, J = 8.1 Hz, 1H), 7.22 (at, J = 7.6 Hz, 1H), 7.09 (at, J = 7.0 Hz, 1H), 3.93 (t, J = 7.2 Hz, 2H), 3.74 (s, 3H), 3.64–3.44 (m, 2H), 2.54–2.40 (m, 1H), 2.34 (s, 1H), 2.10–1.96 (m, 1H), 1.82–1.63 (m, 2H), 1.61–1.44 (m, 1H), 1.40–1.17 (m, 3H), 0.87 (t, J = 7.2 Hz, 3H), 0.93–0.78 (m, 1H), 0.68–0.47 (m, 2H), 0.47–0.40 (m, 1H). **^{13}C NMR** (75.4 MHz, $CDCl_3$): δ (ppm) = 137.1 (C), 133.5 (C), 126.1 (C), 121.2 (CH), 121.0 (CH), 118.8 (CH), 113.7 (C), 109.3 (CH), 86.7 (C), 72.4 (CH), 63.4 (CH_2), 43.1 (C), 42.9 (CH_2), 29.8 (CH_3), 28.4 (CH_2), 27.8 (CH_2), 23.1 (CH_2), 20.0 (CH), 14.1 (CH_3), 3.3 (CH_2), 2.8 (CH_2). **HRMS** (ESI-TOF) *m/z*: $[M+H]^+$ Calcd for $C_{21}H_{28}NO^+$ 310.2165; Found 310.2169.

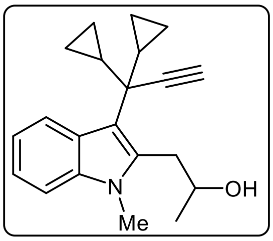

1-(3-(1,1-Dicyclopropylprop-2-yn-1-yl)-1-methyl-1*H*-indol-2-yl)propan-2-ol (3d): Following the general procedure with **S11** (820 mg, 4.3 mmol) and 1,1-dicyclopropyl-3-(trimethylsilyl)prop-2-yn-1-ol (1.20 g, 5.6 mmol), the crude product was purified by column chromatography (hexane/EtOAc, 3/1) affording pure **3d** as an orange oil (818 mg, 62%). R_f = 0.23 (hexane/EtOAc, 3/1). M.p. = 76–78 °C. **^{1}H NMR** (500 MHz, $CDCl_3$): δ (ppm) = 8.16 (d, J = 8.0 Hz, 1H), 7.36 (d, J = 8.2 Hz, 1H), 7.27 (at, J = 8.1 Hz, 1H), 7.15 (at, J = 6.9 Hz, 1H), 4.27–4.15 (m, 1H), 3.76 (s, 3H), 3.49 (dd, J = 15.0, 4.0 Hz, 1H), 3.32 (dd, J = 15.0, 9.2 Hz, 1H), 2.36 (s, 1H), 2.04 (br s, 1H), 1.93–1.73 (m, 2H), 1.35 (d, J = 6.2 Hz, 3H), 0.96–0.83 (m, 2H), 0.76–0.53 (m, 4H), 0.53–0.40 (m, 2H). **^{13}C NMR** (125.7 MHz, $CDCl_3$): δ (ppm) = 137.1 (C), 133.8 (C), 126.4 (C), 121.8 (CH), 120.9 (CH), 118.7 (CH), 115.0 (C), 109.3 (CH), 84.1 (C), 73.5 (CH), 69.3 (CH), 43.3 (C), 34.3 (CH_2), 30.3 (CH_3), 23.2 (CH_3), 20.9 (CH), 20.7 (CH), 3.5 (CH_2), 3.5 (CH_2), 2.5 (CH_2), 2.4 (CH_2). **HRMS** (ESI-TOF) *m/z*: $[M+H]^+$ Calcd for $C_{21}H_{26}NO^+$ 308.2009; Found 308.2017.

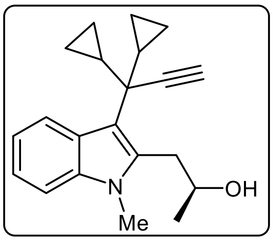

(*S*)-1-(3-(1,1-dicyclopropylprop-2-yn-1-yl)-1-methyl-1*H*-indol-2-yl)propan-2-ol ((*S*)-3d): Following the general procedure with **(*S*)-S11** (190 mg, 1 mmol) and 1,1-dicyclopropyl-3-(trimethylsilyl)prop-2-yn-1-ol (270 mg, 1.3 mmol), the crude product was purified by column chromatography (hexane/EtOAc, 3/1) affording pure **(*S*)-3d** as an orange oil (203 mg, 75%). **HRMS** (ESI-TOF) *m/z*: calcd for $C_{21}H_{26}NO^+$ $[M+H]^+$ 308.2009; found 308.2017. **Column:** Chiralpak OD-H; **Flow:** 0.5 mL/min; **Eluent:** hex/*i*-PrOH 95/5; 100% ee; $[\alpha]_D^{25}$ = –31.3 (c 0.016 g/mL in acetone).

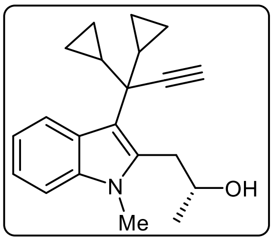

(*R*)-1-(3-(1,1-Dicyclopropylprop-2-yn-1-yl)-1-methyl-1*H*-indol-2-yl)propan-2-ol ((*R*)-3d): Following the general procedure with **(*R*)-S11** (190 mg, 1 mmol) and 1,1-dicyclopropyl-3-(trimethylsilyl)prop-2-yn-1-ol (270 g, 1.3 mmol), the crude product was purified by column chromatography (hexane/EtOAc, 3/1) affording pure **(*R*)-3d** as an orange oil (224 mg, 73%). **HRMS** (ESI-TOF) *m/z*: calcd for $C_{21}H_{26}NO^+$ $[M+H]^+$ 308.2009; found 308.2012. **Column:** Chiralpak OD-H; **Flow:** 0.5 mL/min; **Eluent:** hex/*i*-PrOH 95/5; 100% ee; $[\alpha]_D^{25}$ = 33.5 (c 0.003 g/mL in acetone).

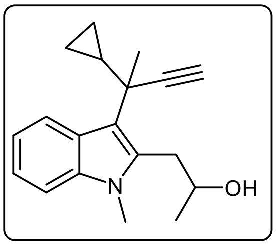

1-(3-(2-Cyclopropylbut-3-yn-2-yl)-1-methyl-1*H*-indol-2-yl)propan-2-ol (3e): Following the general procedure with **S11** (400 mg, 2.1 mmol) and 2-cyclopropyl-4-(trimethylsilyl)but-3-yn-2-ol (500 mg, 2.75 mmol), the crude product was purified by column chromatography (hexane/EtOAc, 3/1) affording pure **3e** as a yellow solid (442 mg, 75%). R_f = 0.23 (hexane/EtOAc, 3/1). M.p. = 75–77 °C. Obtained and isolated as a c.a 1/1 mixture of diasteroisomers. Data for both isomers: **^{1}H NMR** (300 MHz, $CDCl_3$): δ (ppm) = 8.10 (d, *J* = 7.9 Hz, 2H), 7.38 (d, *J* = 8.2 Hz, 2H), 7.30 (at, *J* = 7.1 Hz, 2H), 7.20 (at, *J* = 7.4 Hz, 2H), 4.29–4.13 (m, 2H), 3.76 (s, 3H), 3.75 (s, 3H), 3.56 (dd, *J* = 14.9, 4.2 Hz, 1H), 3.50–3.39 (m, 2H), 3.31 (dd, *J* = 14.9, 9.0 Hz, 1H), 2.42 (s, 1H), 2.39 (s, 1H), 2.13 (br s, 2H), 2.01 (s, 6H), 1.91–1.69 (m, 2H), 1.39 (s, 3H), 1.37 (s, 3H), 0.98–0.84 (m, 2H), 0.83–0.65 (m, 4H), 0.65–0.51 (m, 2H). **^{13}C NMR** (75.4 MHz, $CDCl_3$): δ (ppm) = 137.14 (C), 137.10 (C), 133.3 (C), 133.1 (C), 126.1 (C), 126.0 (C), 121.4 (CH), 121.3 (CH), 121.00 (CH), 120.95 (CH), 118.69 (CH), 118.69 (CH), 115.4 (C), 115.0 (C), 109.31 (CH), 109.27 (CH), 87.8 (C), 87.7 (C), 72.0 (CH), 71.8 (CH), 69.3 (CH), 68.8 (CH), 37.94 (C), 37.92 (C), 34.2 (CH_2), 34.1 (CH_2), 31.1 (CH_3), 30.9 (CH_3), 30.2 (CH_3), 30.1 (CH_3), 23.2 (CH_3), 23.1 (CH_3), 21.0 (CH), 20.8 (CH), 3.99 (CH_2), 3.97 (CH_2), 3.14 (CH_2), 3.09 (CH_2). **HRMS** (ESI-TOF) *m/z*: $[M+H]^+$ Calcd for $C_{19}H_{24}NO^+$ 282.1852; Found 282.1861.

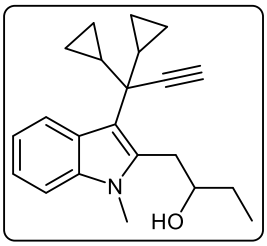

1-(3-(1,1-Dicyclopropylprop-2-yn-1-yl)-1-methyl-1*H*-indol-2-yl)butan-2-ol (3f): Following the general procedure with **S12** (900 mg, 4.4 mmol) and 1,1-dicyclopropyl-3-(trimethylsilyl)prop-2-yn-1-ol (1.22 g, 5.8 mmol), the crude product was purified by column chromatography (hexane/EtOAc, 3/1) affording pure **3f** as an orange oil (1.16 g, 82%). R_f = 0.21 (hexane/EtOAc, 3/1). **^{1}H NMR** (300 MHz, $CDCl_3$): δ (ppm) = 8.23 (d, *J* = 8.1 Hz, 1H), 7.41 (d, *J* = 8.1 Hz, 1H), 7.33 (at, *J* = 7.5 Hz, 1H), 7.22 (at, *J* = 7.5 Hz, 1H), 4.08–3.96 (m, 1H), 3.80 (s, 3H), 3.55 (dd, *J* = 14.9, 3.7 Hz, 1H), 3.41 (dd, *J* = 14.9, 9.6 Hz, 1H), 2.41 (s, 1H), 2.10 (br s, 1H), 1.96–1.80 (m, 2H), 1.80–1.64 (m, 2H), 1.15 (t, *J* = 7.4 Hz, 3H), 1.03–0.84 (m, 2H), 0.82–0.62 (m, 4H), 0.60–0.44 (m, 2H). **^{13}C NMR** (75.4 MHz, $CDCl_3$): δ (ppm) = 137.1 (C), 133.7 (C), 126.4 (C), 121.7 (CH), 120.9 (CH), 118.6 (CH), 115.0 (C), 109.2 (CH), 84.1 (C), 74.1 (CH), 73.5 (CH), 43.2 (C), 32.4 (CH_2), 30.21 (CH_3), 30.20 (CH_2), 20.9 (CH), 20.7 (CH),

10.2 (CH_3), 3.6 (CH_2), 3.4 (CH_2), 2.5 (CH_2), 2.4 (CH_2). **HRMS** (ESI-TOF) *m/z*: $[M+H]^+$ Calcd for $C_{22}H_{28}NO^+$ 322.2165; Found 322.2165.

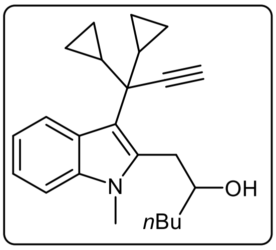

1-(3-(1,1-Dicyclopropylprop-2-yn-1-yl)-1-methyl-1*H*-indol-2-yl)hexan-2-ol (3g): Following the general procedure with **S13** (1.05 g, 4.5 mmol) and 1,1-dicyclopropyl-3-(trimethylsilyl)prop-2-yn-1-ol (1.25 g, 5.9 mmol), the crude product was purified by column chromatography (hexane/EtOAc, 5/1) affording pure **3g** as an orange oil (1.33 g, 85%). R_f = 0.32 (hexane/EtOAc, 5/1). **^{1}H NMR** (300 MHz, $CDCl_3$): δ (ppm) = 8.24 (d, J = 8.1 Hz, 1H), 7.40 (d, J = 8.1 Hz, 1H), 7.37–7.28 (m, 1H), 7.27–7.19 (m, 1H), 4.14–4.02 (m, 1H), 3.78 (s, 3H), 3.56 (dd, J = 14.9, 3.7 Hz, 1H), 3.39 (dd, J = 14.9, 9.5 Hz, 1H), 2.41 (br s, 1H), 2.17 (s, 1H), 1.99–1.83 (m, 2H), 1.79–1.66 (m, 2H), 1.59–1.44 (m, 3H), 1.18–1.06 (m, 3H), 1.05–0.89 (m, 2H), 0.81–0.59 (m, 4H), 0.59–0.40 (m, 2H). **^{13}C NMR** (75.4 MHz, $CDCl_3$): δ (ppm) = 137.2 (C), 133.9 (C), 126.6 (C), 121.9 (CH), 121.0 (CH), 118.8 (CH), 115.0 (C), 109.4 (CH), 84.2 (C), 73.6 (CH), 73.0 (CH), 43.4 (C), 37.3 (CH_2), 33.1 (CH_2), 30.4 (CH_3), 28.2 (CH_2), 23.0 (CH_2), 21.0 (CH), 20.9 (CH), 14.3 (CH_3), 3.7 (CH_2), 3.6 (CH_2), 2.7 (CH_2), 2.6 (CH_2). **HRMS** (ESI-TOF) *m/z*: $[M+H]^+$ Calcd for $C_{24}H_{32}NO^+$ 350.2478; Found 350.2487.

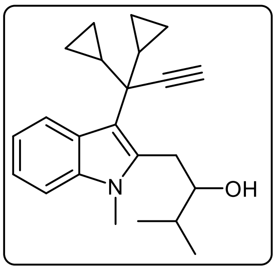

1-(3-(1,1-Dicyclopropylprop-2-yn-1-yl)-1-methyl-1*H*-indol-2-yl)-3-methylbutan-2-ol (3h): Following the general procedure with **S14** (750 mg, 3.45 mmol) and 1,1-dicyclopropyl-3-(trimethylsilyl)prop-2-yn-1-ol (1 g, 4.5 mmol), the crude product was purified by column chromatography (hexane/EtOAc, 5/1) affording pure **3h** as an orange oil (1.01 g, 87%). R_f = 0.34 (hexane/EtOAc, 5/1). **^{1}H NMR** (500 MHz, $CDCl_3$): δ (ppm) = 8.12 (d, J = 8.2 Hz, 1H), 7.33 (d, J = 8.2 Hz, 1H), 7.26–7.17 (m, 1H), 7.17–7.03 (m, 1H), 3.82–3.76 (m, 1H), 3.74 (s, 3H), 3.49–3.33 (m, 2H), 2.33 (s, 1H), 1.97–1.87 (m, 1H), 1.87–1.78 (m, 1H), 1.78–1.70 (m, 2H), 1.09 (t, J = 7.3 Hz, 6H), 0.97–0.73 (m, 2H), 0.70–0.51 (m, 4H), 0.46–0.31 (m, 2H). **^{13}C NMR** (125.7 MHz, $CDCl_3$): δ (ppm) = 137.3 (C), 133.7 (C), 126.6 (C), 121.9 (CH), 121.0 (CH), 118.7 (CH), 115.6 (C), 109.3 (CH), 84.4 (C), 76.7 (CH), 73.9 (CH), 43.3 (C), 34.3 (CH), 30.4 (CH_3), 29.7 (CH_2), 21.1 (CH), 20.8 (CH), 18.9 (CH_3), 17.6 (CH_3), 3.8 (CH_2), 3.4 (CH_2), 2.6 (CH_2), 2.5 (CH_2). **HRMS** (ESI-TOF) *m/z*: $[M+H]^+$ Calcd for $C_{23}H_{30}NO^+$ 336.2322; Found 336.2330.

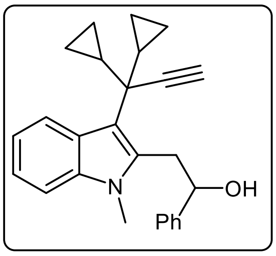

2-(3-(1,1-Dicyclopropylprop-2-yn-1-yl)-1-methyl-1*H*-indol-2-yl)-1-phenylethan-1-ol (3i): Following the general procedure with **S15** (800 mg, 3.2 mmol) and 1,1-dicyclopropyl-3-(trimethylsilyl)prop-2-yn-1-ol (880 mg, 4.15 mmol), the crude product was purified by column chromatography (hexane/EtOAc, 3/1) affording pure **3i** as an orange solid (1.04 g, 88%). R_f = 0.31 (hexane/EtOAc, 3/1). M.p. = 100–102 °C. **^{1}H NMR** (300 MHz, $CDCl_3$): δ (ppm) = 8.10 (d, J = 8.1 Hz, 1H), 7.45–7.25 (m, 6H), 7.23–7.15 (m, 1H), 7.13–7.02 (m, 1H), 5.42–4.98 (m, 1H), 3.77 (dd, J = 14.9, 8.9 Hz, 1H), 3.57 (dd, J = 14.9, 8.9 Hz, 1H), 3.53 (s, 3H), 2.33 (s, 1H), 2.14 (br s, 1H), 1.91–1.71 (m, 2H), 0.94–0.77 (m, 2H), 0.68–0.48 (m, 4H), 0.47–0.31 (m, 2H). **^{13}C NMR** (75.4 MHz, $CDCl_3$): δ (ppm) = 144.2 (C), 137.1 (C), 132.7 (C), 128.5 (2 × CH), 127.6 (C), 126.4 (CH), 125.8 (2 × CH), 121.9 (CH), 121.0 (CH), 118.8 (CH), 115.3 (C), 109.5 (CH), 83.9 (C), 75.2 (CH), 73.9 (CH), 43.5 (C), 34.9 (CH_2), 29.9 (CH_3), 21.1 (CH), 20.8 (CH), 3.7 (CH_2), 3.6 (CH_2), 2.6 (CH_2), 2.2 (CH_2). **HRMS** (ESI-TOF) *m/z*: $[M+H]^+$ Calcd for $C_{26}H_{28}NO^+$ 370.2165; Found 370.2174.

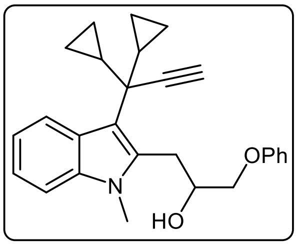

1-(3-(1,1-Dicyclopropylprop-2-yn-1-yl)-1-methyl-1*H*-indol-2-yl)-3-phenoxypropan-2-ol (3j): Following the general procedure with **S16** (850 mg, 3 mmol) and 1,1-dicyclopropyl-3-(trimethylsilyl)prop-2-yn-1-ol (830 mg, 3.9 mmol), the crude product was purified by column chromatography (hexane/EtOAc, 3/1) affording pure **3j** as an orange oil (933 mg, 78%). R_f = 0.20 (hexane/EtOAc, 3/1). **^{1}H NMR** (300 MHz, $CDCl_3$): δ (ppm) = 8.16 (d, *J* = 8.1 Hz, 1H), 7.41–7.32 (m, 3H), 7.27 (at, *J* = 7.5 Hz, 1H), 7.15 (at, *J* = 8.0 Hz, 1H), 7.06 (d, *J* = 7.4 Hz, 1H), 7.04–6.94 (m, 2H), 4.54–4.37 (m, 1H), 4.09 (t, *J* = 4.7 Hz, 2H), 3.80 (s, 3H), 3.74 (dd, *J* = 15.1, 4.9 Hz, 1H), 3.54 (dd, *J* = 15.1, 8.8 Hz, 1H), 2.56 (d, *J* = 4.4 Hz, 1H), 2.38 (s, 1H), 1.92–1.78 (m, 2H), 0.99–0.80 (m, 2H), 0.74–0.51 (m, 4H), 0.51–0.41 (m, 2H). **^{13}C NMR** (75.4 MHz, $CDCl_3$): δ (ppm) = 158.6 (C), 137.2 (C), 132.7 (C), 129.7 (2 × CH), 126.5 (C), 121.9 (CH), 121.3 (CH), 121.1 (CH), 118.8 (CH), 115.3 (C), 114.6 (2 × CH), 109.4 (CH), 84.2 (C), 73.8 (CH), 71.4 (CH), 71.3 (CH_2), 43.4 (C), 30.3 (CH_3), 29.0 (CH_2), 20.9 (2 × CH), 3.6 (2 × CH_2), 2.6 (CH_2), 2.5 (CH_2). **HRMS** (ESI-TOF) *m/z*: $[M+H]^+$ Calcd for $C_{27}H_{30}NO_2^+$ 400.2271; Found 400.2267.

1-(Allyloxy)-3-(3-(1,1-dicyclopropylprop-2-yn-1-yl)-1-methyl-1*H*-indol-2-yl)propan-2-ol (3k): Following the general procedure with **S17** (360 mg, 1.5 mmol) and 1,1-dicyclopropyl-3-(trimethylsilyl)prop-2-yn-1-ol (400 mg, 1.9 mmol), the crude product was purified by column chromatography (hexane/EtOAc, 3/1) affording pure **3k** as a yellow oil (360 mg, 66%). R_f = 0.15 (hexane/EtOAc, 3/1). **^{1}H NMR** (300 MHz, $CDCl_3$): δ (ppm) = 8.16 (d, *J* = 8.1 Hz, 1H), 7.36 (d, *J* = 8.1 Hz, 1H), 7.26 (at, *J* = 7.5 Hz, 1H), 7.15 (at, *J* = 7.5 Hz, 1H), 6.08–5.91 (m, 1H), 5.37 (d, *J* = 17.2 Hz, 1H), 5.28 (d, *J* = 10.4 Hz, 1H), 4.31–4.22 (m, 1H), 4.11 (d, *J* = 5.6 Hz, 2H), 3.79 (s, 3H), 3.68–3.48 (m, 3H), 3.39 (dd, *J* = 15.0, 8.7 Hz, 1H), 2.60 (br s, 1H), 2.38 (s, 1H), 1.92–1.77 (m, 2H), 1.01–0.84 (m, 2H), 0.78–0.53 (m, 4H), 0.53–0.38 (m, 2H). **^{13}C NMR** (75.4 MHz, $CDCl_3$): δ (ppm) = 137.1 (C), 134.6 (CH), 133.1 (C), 126.5 (C), 121.8 (CH), 120.9 (CH), 118.7 (CH), 117.3 (CH_2), 114.9 (C), 109.3 (CH), 84.0 (C), 73.9 (CH_2), 73.6 (CH), 72.4 (CH_2), 71.8 (CH), 43.3 (C), 30.2 (CH_3), 28.9 (CH_2), 20.8 (2 × CH), 3.5 (2 × CH_2), 2.5 (CH_2), 2.4 (CH_2). **HRMS** (ESI-TOF) *m/z*: $[M+H]^+$ Calcd for $C_{24}H_{30}NO_2^+$ 364.2271; Found 364.2271.

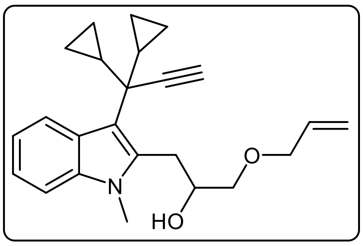

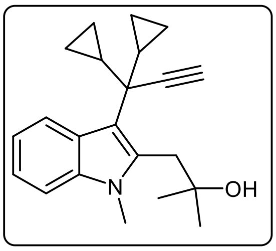

1-(3-(1,1-Dicyclopropylprop-2-yn-1-yl)-1-methyl-1*H*-indol-2-yl)-2-methylpropan-2-ol (3l): Following the general procedure with **S18** (910 mg, 4.5 mmol) and 1,1-dicyclopropyl-3-(trimethylsilyl)prop-2-yn-1-ol (1.23 g, 5.8 mmol), the crude product was purified by column chromatography (hexane/EtOAc, 3/1) affording pure **3l** as a yellow oil (940 mg, 65%). R_f = 0.31 (hexane/EtOAc, 3/1). **^{1}H NMR** (300 MHz, $CDCl_3$): δ (ppm) = 8.11 (d, *J* = 8.1 Hz, 1H), 7.34 (d, *J* = 8.1 Hz, 1H), 7.23 (at, *J* = 7.5 Hz, 1H), 7.12 (at, *J* = 7.5 Hz, 1H), 3.75 (s, 3H), 3.56 (br s, 1H), 2.36 (s, 1H), 1.98–1.74 (m, 2H), 1.34 (s, 6H), 0.99–0.77 (m, 2H), 0.73–0.46 (m, 4H), 0.46–0.20 (m, 2H). **^{13}C NMR** (75.4 MHz, $CDCl_3$): δ (ppm) = 137.8 (C), 132.6 (C), 126.6 (C), 121.9 (CH), 121.1 (CH), 118.6 (CH), 117.2 (C), 109.5 (CH), 84.2 (C), 74.5 (CH), 71.2 (C), 43.4 (C), 36.4 (CH_2), 31.1 (CH_3), 30.3 (2 x CH_3), 20.5 (2 × CH), 3.6 (2 × CH_2), 2.5 (2 × CH_2). **HRMS** (ESI-TOF) *m/z*: $[M+H]^+$ Calcd for $C_{22}H_{28}NO^+$ 322.2165; Found 322.2174.

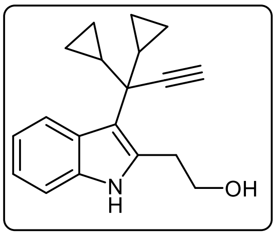

2-(3-(1,1-Dicyclopropylprop-2-yn-1-yl)-1*H*-indol-2-yl)ethan-1-ol (3m): Following the general procedure with **S19** (317 mg, 2 mmol) and 1,1-dicyclopropyl-3-(trimethylsilyl)prop-2-yn-1-ol (550 mg, 2.6 mmol), the crude product was purified by column chromatography (hexane/EtOAc, 2/1) affording pure **3m** as an orange oil (345 mg, 62%). R_f = 0.23 (hexane/EtOAc, 2/1). **^{1}H NMR** (300 MHz, $CDCl_3$): δ (ppm) = 8.69 (br s, 1H), 8.12 (d, *J* = 7.9 Hz, 1H), 7.30 (d, *J* = 8.0 Hz, 1H), 7.23–6.97 (m, 2H), 3.92 (t, *J* = 5.9 Hz, 2H), 3.34 (t, *J* = 5.9 Hz, 2H), 2.53 (br s, 1H), 2.27 (s, 1H), 1.72–1.54 (m, 2H), 0.87–0.72 (m, 2H), 0.72–0.23 (m, 6H). **^{13}C NMR** (75.4 MHz, $CDCl_3$): δ (ppm) = 135.3 (C), 133.7 (C), 127.3 (C), 121.7 (CH), 120.9 (CH), 118.9 (CH), 113.6 (C), 110.7 (CH), 84.4 (C), 72.4 (CH), 63.0 (CH_2), 42.7 (C), 30.8 (CH_2), 20.9 (2 × CH), 3.4 (2 × CH_2), 2.4 (2 × CH_2). **HRMS** (ESI-TOF) *m/z*: $[M+H]^+$ Calcd for $C_{19}H_{22}NO^+$ 280.1696; Found 280.1704.

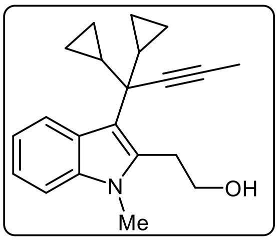

2-(3-(1,1-Dicyclopropylbut-2-yn-1-yl)-1-methyl-1*H*-indol-2-yl)ethan-1-ol (3n): Following the general procedure with **S10** (600 mg, 3.4 mmol) and 1,1-dicyclopropylbut-2-yn-1-ol (663 mg, 4.4 mmol), the crude product was purified by column chromatography (hexane/EtOAc, 2/1) affording pure **3n** as a yellow oil (856 mg, 82%). R_f = 0.25 (hexane/EtOAc, 2/1). **^{1}H NMR** (300 MHz, $CDCl_3$): δ (ppm) = 8.18 (d, *J* = 7.8 Hz, 1H), 7.35 (d, *J* = 8.1 Hz, 1H), 7.32–7.23 (m, 1H), 7.21–7.12 (m, 1H), 3.93 (t, *J* = 7.2 Hz, 2H), 3.73 (s, 3H), 3.54 (t, *J* = 7.2 Hz, 2H), 2.24 (br s, 1H), 1.95 (s, 3H), 1.87–1.69 (m, 2H), 0.93–0.81 (m, 2H), 0.72–0.62 (m, 2H), 0.62–0.50 (m, 2H), 0.51–0.39 (m, 2H). **^{13}C NMR** (75.4 MHz, $CDCl_3$): δ (ppm) = 136.9 (C), 133.0 (C), 126.5 (C), 121.7 (CH), 120.7 (CH), 118.6 (CH), 115.7 (C), 109.0 (CH), 79.8 (C), 78.8 (C), 63.0 (CH_2), 43.3 (C), 29.6 (CH_3), 28.6 (CH_2), 21.2 (2 × CH), 3.4 (2 × CH_2), 3.3 (CH_3), 2.3 (2 × CH_2). **HRMS** (ESI-TOF) *m/z*: $[M+H]^+$ Calcd for $C_{21}H_{26}NO^+$ 308.2009; Found 308.2016.

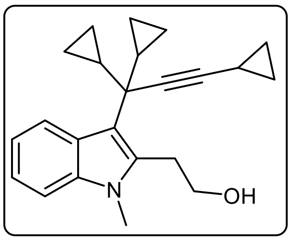

2-(1-Methyl-3-(1,1,3-tricyclopropylprop-2-yn-1-yl)-1*H*-indol-2-yl)ethan-1-ol (3o): Following the general procedure with **S10** (525 mg, 3 mmol) and 1,1,3-tricyclopropylprop-2-yn-1-ol (686 mg, 3.9 mmol), the crude product was purified by column chromatography (hexane/EtOAc, 3/1) affording pure **3o** as an orange oil (669 mg, 67%). R_f = 0.22 (hexane/EtOAc, 3/1). **^{1}H NMR** (300 MHz, $CDCl_3$): δ (ppm) = 8.21 (d, *J* = 8.1 Hz, 1H), 7.39 (d, *J* = 8.1 Hz, 1H), 7.32 (at, *J* = 7.4 Hz, 1H), 7.21 (at, *J* = 7.4 Hz, 1H), 3.98 (t, *J* = 7.2 Hz, 2H), 3.77 (s, 3H), 3.60 (t, *J* = 7.2 Hz, 2H), 2.45 (br s, 1H), 1.87 (dq, *J* = 8.1, 5.4 Hz, 2H), 1.46–1.35 (m, 1H), 0.99–0.79 (m, 6H), 0.78–0.64 (m, 2H), 0.64–0.53 (m, 2H), 0.53–0.43 (m, 2H). **^{13}C NMR** (75.4 MHz, $CDCl_3$): δ (ppm) = 136.9 (C), 132.9 (C), 126.4 (C), 121.7 (CH), 120.7 (CH), 118.5 (CH), 115.7 (C), 109.0 (CH), 87.5 (C), 74.6 (C), 63.0 (CH_2), 43.2 (C), 29.5 (CH_3), 28.5 (CH_2), 21.2 (2 × CH), 7.8 (2 × CH_2), 3.2 (2 × CH_2), 2.2 (2 × CH_2), –0.5 (CH). **HRMS** (ESI-TOF) *m/z*: $[M+H]^+$ Calcd for $C_{23}H_{28}NO^+$ 334.2165; Found 334.2161.

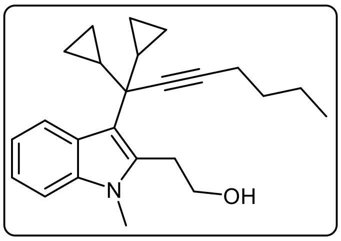

2-(3-(1,1-Dicyclopropylhept-2-yn-1-yl)-1-methyl-1*H*-indol-2-yl)ethan-1-ol (3p): Following the general procedure with **S10** (525 mg, 3 mmol) and 1,1-dicyclopropylhept-2-yn-1-ol (748 mg, 3.9 mmol), the crude product was purified by column chromatography (hexane/EtOAc, 3/1) affording pure **3p** as an orange oil (774 mg,74%). R_f = 0.18 (hexane/EtOAc, 3/1). **^{1}H NMR** (300 MHz, $CDCl_3$): δ (ppm) = 8.13 (d, *J* = 8.1 Hz, 1H), 7.32 (d, *J* = 8.1 Hz, 1H), 7.22 (at, *J* = 7.3 Hz, 1H), 7.11 (at, *J* = 7.2 Hz, 1H), 3.93 (t, *J* = 7.1 Hz,

2H), 3.74 (s, 3H), 3.55 (t, *J* = 7.1 Hz, 2H), 2.29 (t, *J* = 6.8 Hz, 2H), 1.84–1.71 (m, 3H), 1.64–1.41 (m, 4H), 1.00 (t, *J* = 7.1 Hz, 3H), 0.82 (td, *J* = 9.5, 5.2 Hz, 2H), 0.66–0.57 (m, 2H), 0.57–0.46 (m, 2H), 0.45–0.30 (m, 2H). **^{13}C NMR** (75.4 MHz, $CDCl_3$): δ (ppm) = 136.9 (C), 132.9 (C), 126.5 (C), 121.7 (CH), 120.7 (CH), 118.5 (CH), 115.9 (C), 109.0 (CH), 84.7 (C), 79.2 (C), 63.0 (CH_2), 43.3 (C), 31.0 (CH_2), 29.5 (CH_3), 28.5 (CH_2), 22.1 (CH_2), 21.2 (2 × CH), 18.4 (CH_2), 13.6 (CH_3), 3.2 (2 × CH_2), 2.2 (2 × CH_2). **HRMS** (ESI-TOF) *m/z*: $[M+H]^+$ Calcd for $C_{24}H_{32}NO^+$ 350.2478; Found 350.2478.

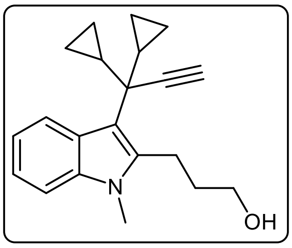

3-(3-(1,1-Dicyclopropylprop-2-yn-1-yl)-1-methyl-1*H*-indol-2-yl)propan-1-ol (9a): Following the general procedure with **S20** (370 mg, 2 mmol) and 1,1-dicyclopropyl-3-(trimethylsilyl)prop-2-yn-1-ol (540 mg, 2.6 mmol), the crude product was purified by column chromatography (hexane/EtOAc, 2/1) affording pure **9a** as an orange oil (246 mg, 40%). R_f = 0.14 (hexane/EtOAc, 2/1). **^{1}H NMR** (300 MHz, $CDCl_3$): δ (ppm) = 8.12 (d, *J* = 8.0 Hz, 1H), 7.30 (d, *J* = 7.6 Hz, 1H), 7.21 (at, *J* = 7.5 Hz, 1H), 7.10 (at, *J* = 7.5 Hz, 1H), 3.78 (t, *J* = 6.3 Hz, 2H), 3.71 (s, 3H), 3.36–3.19 (m, 2H), 2.30 (s, 1H), 2.01–1.88 (m, 2H), 1.85 (s, 1H), 1.77–1.65 (m, 2H), 0.93–0.78 (m, 2H), 0.70–0.38 (m, 6H). **^{13}C NMR** (75.4 MHz, $CDCl_3$): δ (ppm) = 136.9 (C), 136.8 (C), 126.7 (C), 121.7 (CH), 120.6 (CH), 118.7 (CH), 113.0 (C), 108.9 (CH), 84.4 (C), 72.2 (CH), 62.7 (CH_2), 43.0 (C), 33.7 (CH_3), 29.5 (CH_2), 22.2 (2 × CH), 20.9 (CH_2), 3.6 (2 × CH_2), 2.5 (2 × CH_2).**HRMS** (ESI-TOF) *m/z*: $[M+H]^+$ Calcd for $C_{21}H_{26}NO^+$ 308.2009; Found 308.2017.

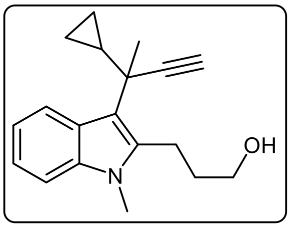

3-(3-(2-Cyclopropylbut-3-yn-2-yl)-1-methyl-1*H*-indol-2-yl)propan-1-ol (9b): Following the general procedure with **S20** (360 mg, 3.7 mmol) and 1,1-dicyclopropyl-3-(trimethylsilyl)prop-2-yn-1-ol (870 mg, 4.8 mmol), the crude product was purified by column chromatography (hexane/EtOAc, 3/1) affording pure **9b** as a yellow oil (478 mg, 46%). R_f = 0.10 (hexane/EtOAc, 3/1). **^{1}H NMR** (300 MHz, $CDCl_3$): δ (ppm) = 8.07 (d, *J* = 8.1 Hz, 1H), 7.33 (d, *J* = 8.0 Hz, 1H), 7.24 (at, *J* = 7.0 Hz, 1H), 7.14 (at, *J* = 7.0 Hz, 1H), 3.81 (t, *J* = 6.2 Hz, 2H), 3.73 (s, 3H), 3.41–3.22 (m, 2H), 2.35 (s, 1H), 1.95 (s, 3H), 2.03–1.84 (m, 3H), 1.76–1.62 (m, 1H), 0.87–0.77 (m, 1H), 0.78–0.70 (m, 1H), 0.69–0.53 (m, 2H). **^{13}C NMR** (75.4 MHz, $CDCl_3$): δ (ppm) = 136.9 (C), 136.3 (C), 126.2 (C), 121.3 (CH), 120.7 (CH), 118.8 (CH), 113.8 (C), 109.0 (CH), 88.0 (C), 70.9 (CH), 62.6 (CH_2), 37.8 (C), 33.6 (CH_2), 31.0 (CH_3), 29.4 (CH_3), 21.9 (CH_2), 21.1 (CH), 4.0 (CH_2), 3.1 (CH_2). **HRMS** (ESI-TOF) *m/z*: $[M+H]^+$ Calcd for $C_{19}H_{24}NO^+$ 282.1852; Found 282.1853.

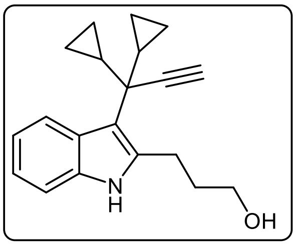

3-(3-(1,1-Dicyclopropylprop-2-yn-1-yl)-1*H*-indol-2-yl)propan-1-ol (9c): Following the general procedure with **S21** (250 mg, 1.5 mmol) and 1,1-dicyclopropyl-3-(trimethylsilyl)prop-2-yn-1-ol (406 mg, 1.95 mmol), the crude product was purified by column chromatography (hexane/EtOAc, 2/1) affording pure **9c** as a yellow oil (312 mg, 71%). R_f = 0.20 (hexane/EtOAc, 2/1). **^{1}H NMR** (300 MHz, $CDCl_3$): δ (ppm) = 8.53 (br s, 1H), 8.12 (d, *J* = 7.9 Hz, 1H), 7.29 (d, *J* = 7.3 Hz, 1H), 7.24–7.02 (m, 2H), 3.75 (t, *J* = 6.1 Hz, 2H), 3.25 (t, *J* = 7.5 Hz, 2H), 2.44 (br s, 1H), 2.29 (s, 1H), 2.06–1.90 (m, 2H), 1.78–1.55 (m, 2H), 0.92–0.73 (m, 2H), 0.73–0.52 (m, 4H), 0.52–0.39 (m, 2H). **^{13}C NMR** (75.4 MHz, $CDCl_3$): δ (ppm) = 135.3 (C), 135.2 (C), 127.6 (C), 121.7 (CH), 120.8 (CH), 118.8 (CH), 113.2 (C), 110.5 (CH), 84.3 (C), 72.1 (CH), 62.2 (CH_2), 42.8 (C), 33.2 (CH_2), 24.3 (CH_2), 20.8 (2 × CH), 3.4 (2 × CH_2), 2.4 (2 × CH_2). **HRMS** (ESI-TOF) *m/z*: $[M+H]^+$ Calcd for $C_{20}H_{24}NO^+$ 294.1852; Found 294.1860.

Synthesis and characterization data of tetrahydropyrano[3,4-*b*]indole derivatives 2 and 1,3-dien-2-yl-indol derivatives 7

Optimization studies

As already mentioned in the main manuscript, a study was conducted to optimize the reaction conditions for the tandem 1,2-indole migration–hydroxycyclization sequence. Herein, we present the table summarizing the detailed optimization process for the reaction of (3-(1,1-dicyclopropylprop-2-yn-1-yl)-1-methyl-1*H*-indol-2-yl)methanol (**1a**). The study focused primarily on evaluating the role of the gold(I) catalyst, as well as the presence of silver salts, on the efficiency and selectivity of the reaction (Table S1). All the tested gold catalysts and silver salts are commercially available and were purchased from Sigma-Aldrich and BLDpharm: IPrAuNTf$_2$ (CAS 951776-24-2), Ph$_3$PAuNTf$_2$ (CAS 1246810-76-3), XPhosAuNTf$_2$ (CAS 934506-10-2), BrettPhosAuNTf$_2$ (CAS 1296269-97-0), SPhosAuNTf$_2$ (CAS 1121960-90-4), JohnPhosAu(MeCN)SbF$_6$ (CAS 866641-66-9), JohnPhosAuCl (CAS 854045-93-5).

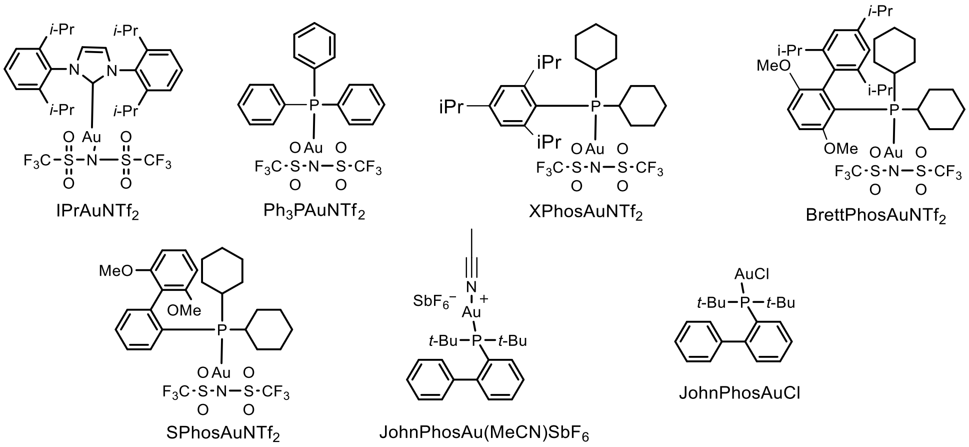

Table S1: Optimization of the conditions for the Au-catalyzed tandem 1,2-indole migration–hydroxycyclization of **1a**[a]

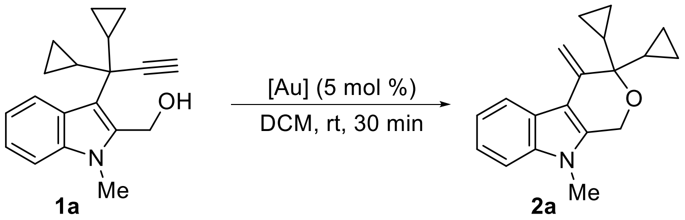

entry	Catalyst [Au]	conversion (%)[b]	yield (%)[b]
**1**	IPrAuNTf$_2$	100	90
**2**	Ph$_3$PAuNTf$_2$	70	47
**3**	XPhosAuNTf$_2$	100	85
**4**	BrettPhosAuNTf$_2$	100	79
**5**	SPhosAuNTf$_2$	100	65
**6**	JohnPhosAu(MeCN)SbF$_6$	100	97
**7**	JohnPhosAuCl/AgNTf$_2$	100	64
**8**	JohnPhosAuCl/AgSbF$_6$	100	54
**9**	JohnPhosAuCl/AgBF$_4$	100	61

[a]Reaction conditions: **1a** (0.15 mmol), gold catalyst (5 mol %), in DCM (1 mL) at rt for 30 min. [b]Determined by ^1H NMR analysis using CH_2Br_2 as internal standard.

When a gold catalyst bearing an NHC ligand was employed, the desired product **2a** was obtained in 90% yield (entry 1). Although this initial result demonstrated high efficiency, additional catalysts were also evaluated. In particular, catalysts featuring phosphine ligands were examined (entries 2–6). When $Ph_3PAuNTf_2$ was used as the catalyst (entry 2), the reaction reached only 70% conversion under the established conditions, affording **2a** in 47% yield. In contrast, the remaining catalysts (entries 3–5) provided complete conversion of **1a** and good yields in all cases. Notably, the use of JohnPhosAu(MeCN)SbF_6 as the catalyst (entry 6) led to a further improvement, delivering **2a** in 97% yield.

Next, we investigated the influence of silver salts and counterions on the hydroxycyclization reactivity. Treatment of **1a** with catalytic systems generated *in situ* from JohnPhosAuCl and various silver salts (entries 7–9) resulted in decreased yields of **2a**, indicating a detrimental effect of silver salts on the reaction outcome.

Based on these results, JohnPhosAu(MeCN)SbF_6 in DCM was identified as the optimal catalyst for this transformation, providing the highest yields and shortest reaction times in the absence of silver additives.

In a similar manner, a brief study was conducted using (3-(1,1-dicyclopropylprop-2-yn-1-yl)-1*H*-indol-2-yl)methanol (**1k**) as a model substrate to evaluate the reaction conditions for the tandem 1,2-indole migration/hydroxycyclization sequence. This study likewise focused on assessing the influence of the gold(I) catalyst and the presence of silver salts on the efficiency and selectivity of the process (Table S2).

Table S2: Optimization of the conditions for the Au-catalyzed 1,2-indole migration–hydroxycyclization of **1k**[a]

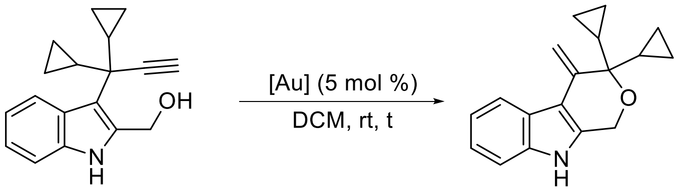

1k **2k**

entry	Catalyst [Au]	t (h)	conversion (%)[b]	yield (%)[b]
**1**	$IPrAuNTf_2$	4	35	30
**2**	$Ph_3PAuNTf_2$	4	100	45
**3**	$XPhosAuNTf_2$	4	100	43
**4**	$BrettPhosAuNTf_2$	0.5	100	70
**5**	$SPhosAuNTf_2$	0.5	58	50
**6**	JohnPhosAu(MeCN)SbF_6	0.5	100	85
**7**	JohnPhosAuCl/$AgNTf_2$	0.5	52	28
**8**	JohnPhosAuCl/$AgSbF_6$	0.5	49	37
**9**	JohnPhosAuCl/NaBARF	0.5	38	26

[a]Reaction conditions: **1k** (0.15 mmol), gold catalyst (5 mol %), in DCM (1 mL) at rt for 30 min. [b]Determined by ^1H NMR analysis using CH_2Br_2 as internal standard.

When a gold catalyst bearing NHC ligands was employed, the desired product **2k** was obtained in 30% yield; however, the reaction did not reach completion, and unreacted starting material remained even

after extending the reaction time to 4 h (entry 1). Therefore, additional catalysts featuring phosphine ligands were evaluated to further examine their reactivity (entries 2–6). In most cases, full conversion was achieved, affording the desired product **2k** in moderate yields (entries 2,3) to good yields (entries 4 and 6). However, when SPhosAuNTf$_2$ was used as the catalyst (entry 5), complete consumption of the starting material was not observed. Notably, the best results were obtained with JohnPhosAu(MeCN)SbF$_6$ (entry 6), which provided improved yields of **2k** and shorter reaction times.

Next, we investigated the influence of silver salts and counterions on the hydroxycyclization reativity. Treatment of **1k** for 30 min with catalytic systems generated *in situ* from JohnPhosAuCl and various silver salts (entries 7–9) resulted in decreased conversion and lower yields of **2k**, indicating a detrimental effect of silver additives on the reaction outcome.

Based on these results, JohnPhosAu(MeCN)SbF$_6$ in DCM was selected as the optimal catalyst for both substrates, as it provided the highest yields and shorter reaction times for these hydroxycyclization provesses.

General procedure for the synthesis of tetrahydropyrano[3,4-b]indol derivatives 2a-l from 3-propargylindoles 1a-l

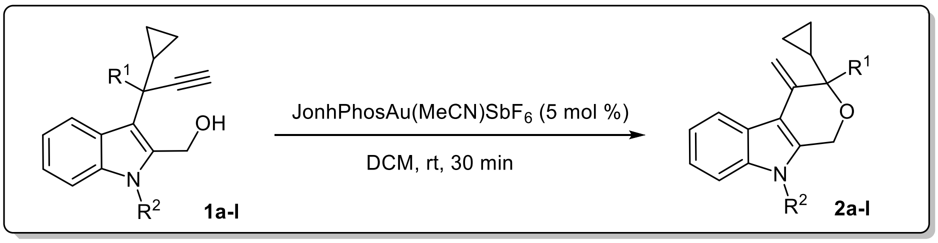

JonhPhosAu(MeCN)SbF$_6$ (19.5 mg, 0.025 mmol, 0.05 equiv) was dissolved in DCM (1 mL), and the resulting solution was stirred at rt for 5 min. A solution of the corresponding 3-propargylindole **1a-l** (0.5 mmol, 1 equiv) in DCM (1 mL) was then added. The reaction mixture was stirred at rt for 30 min (until complete consumption of the starting material, as determined by GC–MS or TLC). The mixture was filtered through a short pad of silica gel and celite using a 2/1 (hexane/EtOAc) mixture as eluent. The solvents were removed under reduced pressure, and the crude product was purified by flash column chromatography using mixtures of hexane/EtOAc as eluent, affording the corresponding tetrahydropyrano[3,4-*b*]indol derivatives **2a-l**.

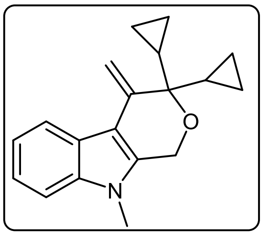

3,3-Dicyclopropyl-9-methyl-4-methylene-1,3,4,9-tetrahydropyrano[3,4-*b*]indole (2a): Following the general procedure with **1a** (140 mg, 0.5 mmol), the crude product was purified by column chromatography (hexane/EtOAc, 5/1) affording pure **2a** as a yellow solid (107 mg, 77%). R_f = 0.23 (hexane/EtOAc, 5/1). M.p. = 67–69 °C. **^{1}H NMR** (300 MHz, CDCl$_3$): δ (ppm) = 7.91 (d, J = 7.0 Hz, 1H), 7.36–7.31 (m, 1H), 7.31–7.20 (m, 2H), 5.69 (s, 1H), 5.23 (s, 1H), 5.06 (s, 2H), 3.60 (s, 3H), 1.28–1.16 (m, 2H), 0.60–0.42 (m, 8H). **^{13}C NMR** (75.4 MHz, CDCl$_3$): δ (ppm) = 140.4 (C), 137.8 (C), 136.0 (C), 124.6 (C), 121.5 (CH), 120.4 (CH), 120.3 (CH), 109.1 (CH), 108.3 (C), 104.9 (CH$_2$), 78.9 (C), 58.0 (CH$_2$), 29.5 (CH$_3$), 16.6 (2 × CH), 2.3 (2 × CH$_2$), -0.1 (2 × CH$_2$). **HRMS** (ESI-TOF) *m/z*: [M+H]$^+$ Calcd for $C_{19}H_{22}NO^+$ 280.1696; Found 280.1696. **2a** could also be obtained in a 2-mmol scale as a yellow solid (480 mg, 86%), starting from **1a** (560 mg, 2 mmol).

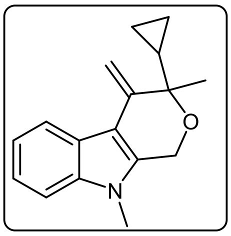

3-Cyclopropyl-3,9-dimethyl-4-methylene-1,3,4,9-tetrahydropyrano[3,4-*b*]indole (2b): Following the general procedure with **1b** (127 mg, 0.5 mmol), the crude product was purified by column chromatography (hexane/EtOAc, 5/1) affording pure **2b** as an orange solid (89 mg, 70%). R_f = 0.24 (hexane/EtOAc, 5/1). M.p. = 60–62 °C. **^{1}H NMR** (300 MHz, $CDCl_3$): δ (ppm) = 7.89 (d, J = 7.2 Hz, 1H), 7.34 (d, J = 7.5 Hz, 1H), 7.31–7.18 (m, 2H), 5.58 (s, 1H), 5.19 (d, J = 15.3 Hz, 1H), 5.04 (s, 1H), 4.89 (d, J = 15.3 Hz, 1H), 3.63 (s, 3H), 1.47 (s, 3H), 1.33–1.15 (m, 1H), 0.54–0.33 (m, 3H), 0.33–0.25 (m, 1H). **^{13}C NMR** (75.4 MHz, $CDCl_3$): δ (ppm) = 142.1 (C), 137.9 (C), 135.7 (C), 124.9 (C), 121.6 (CH), 120.4 (CH), 120.4 (CH), 109.1 (CH), 107.9 (C), 103.6 (CH_2), 78.3 (C), 58.1 (CH_2), 29.6 (CH_3), 23.8 (CH), 17.8 (CH_3), 3.3 (CH_2), -0.2 (CH_2). **HRMS** (ESI-TOF) *m/z*: $[M+H]^+$ Calcd for $C_{17}H_{20}NO^+$ 254.1539; Found 254.1542.

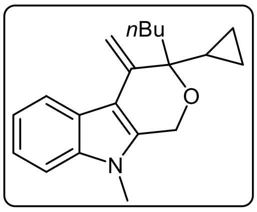

3-Butyl-3-cyclopropyl-9-methyl-4-methylene-1,3,4,9-tetrahydropyrano[3,4-*b*]indole (2c): Following the general procedure with **1c** (148 mg, 0.5 mmol), the crude product was purified by column chromatography (hexane/EtOAc, 5/1) affording pure **2c** as a white oil (122 mg, 83%). R_f = 0.32 (hexane/EtOAc, 5/1). **^{1}H NMR** (300 MHz, $CDCl_3$): δ (ppm) = 7.91 (d, J = 7.7 Hz, 1H), 7.34 (d, J = 7.1 Hz, 1H), 7.31–7.19 (m, 2H), 5.66 (s, 1H), 5.20 (d, J = 15.2 Hz, 1H), 4.91 (s, 1H), 4.88 (d, J = 15.2 Hz, 1H), 3.62 (s, 3H), 2.06–1.81 (m, 2H), 1.69–1.51 (m, 2H), 1.49–1.31 (m, 2H), 1.25–1.10 (m, 1H), 0.97 (t, J = 7.2 Hz, 3H), 0.47–0.24 (m, 4H). **^{13}C NMR** (75.4 MHz, $CDCl_3$): δ (ppm) = 139.3 (C), 137.8 (C), 136.1 (C), 124.9 (C), 121.6 (CH), 120.5 (CH), 120.4 (CH), 109.1 (CH), 108.2 (C), 103.9 (CH_2), 80.0 (C), 57.8 (CH_2), 38.6 (CH_2), 29.5 (CH_3), 25.9 (CH_2), 23.5 (CH_2), 16.8 (CH), 14.3 (CH_3), 2.8 (CH_2), -0.8 (CH_2). **HRMS** (ESI-TOF) *m/z*: $[M+H]^+$ Calcd for $C_{20}H_{26}NO^+$ 296.2009; Found 296.2009.

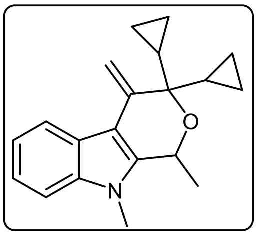

3,3-Dicyclopropyl-1,9-dimethyl-4-methylene-1,3,4,9-tetrahydropyrano[3,4-*b*]indole (2d): Following the general procedure with **1d** (147 mg, 0.5 mmol), the crude product was purified by column chromatography (hexane/EtOAc, 10/1) affording pure **2d** as a yellow oil (111 mg, 76%). R_f = 0.30 (hexane/EtOAc, 10/1). **^{1}H NMR** (500 MHz, $CDCl_3$): δ (ppm) = 7.93 (d, J = 7.8 Hz, 1H), 7.35 (d, J = 8.1 Hz, 1H), 7.32–7.26 (m, 1H), 7.26–7.21 (m, 1H), 5.68 (s, 1H), 5.32 (q, J = 6.4 Hz, 1H), 5.24 (s, 1H), 3.73 (s, 3H), 1.68 (d, J = 6.4 Hz, 3H), 1.40–1.30 (m, 1H), 1.11–1.03 (m, 1H), 0.89–0.78 (m, 1H), 0.73–0.59 (m, 1H), 0.59–0.46 (m, 2H), 0.46–0.33 (m, 2H), 0.33–0.23 (m, 1H), 0.13–0.02 (m, 1H). **^{13}C NMR** (125.7 MHz, $CDCl_3$): δ (ppm) = 141.3 (C), 139.7 (C), 138.3 (C), 124.4 (C), 121.7 (CH), 120.4 (CH), 120.3 (CH), 109.1 (CH), 108.6 (C), 104.7 (CH_2), 77.7 (C), 64.1 (CH), 31.3 (CH_3), 21.6 (CH_3), 18.3 (CH), 16.3 (CH), 2.7 (CH_2), 2.4 (CH_2), 0.6 (CH_2), 0.5 (CH_2). **HRMS** (ESI-TOF) *m/z*: $[M+H]^+$ Calcd for $C_{20}H_{24}NO^+$ 294.1852; Found 294.1860.

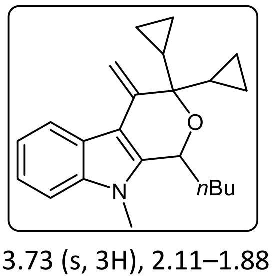

1-Butyl-3,3-dicyclopropyl-9-methyl-4-methylene-1,3,4,9-tetrahydropyrano[3,4-*b*]indole (2e): Following the general procedure with **1e** (167 mg, 0.5 mmol), the crude product was purified by column chromatography (hexane/EtOAc, 5/1) affording pure **2e** as an orange oil (114 mg, 68%). R_f = 0.25 (hexane/EtOAc, 5/1). **^{1}H NMR** (300 MHz, $CDCl_3$): δ (ppm) = 7.93 (d, J = 7.8 Hz, 1H), 7.36 (d, J = 7.6 Hz, 1H), 7.33–7.18 (m, 2H), 5.67 (s, 1H), 5.26–5.19 (m, 2H), 3.73 (s, 3H), 2.11–1.88 (m, 2H), 1.62–1.24 (m, 5H), 1.21–1.04 (m, 1H), 0.97 (t, J = 6.8 Hz, 3H), 0.90–0.78 (m, 1H), 0.71–0.57 (m, 1H), 0.58–0.45 (m, 2H), 0.45–0.35 (m, 2H), 0.35–0.21 (m, 1H), 0.21–0.02 (m, 1H). **^{13}C NMR** (75.4 MHz, $CDCl_3$): δ (ppm) = 141.6 (C), 138.5 (C), 138.3 (C), 124.5 (C), 121.5 (CH), 120.3 (CH),

120.2 (CH), 109.5 (C), 109.0 (CH), 104.3 (CH_2), 76.7 (C), 67.5 (CH), 34.6 (CH_2), 31.3 (CH_3), 26.9 (CH_2), 22.7 (CH_2), 17.8 (CH), 16.9 (CH), 14.2 (CH_3), 2.9 (CH_2), 1.9 (CH_2), 0.6 (CH_2), 0.5 (CH_2). **HRMS** (ESI-TOF) *m/z*: $[M+H]^+$ Calcd for $C_{23}H_{30}NO^+$ 336.2322; Found 336.2323.

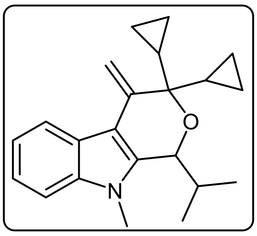

3,3-Dicyclopropyl-1-isopropyl-9-methyl-4-methylene-1,3,4,9-tetrahydropyrano[3,4-*b*]indole (2f): Following the general procedure with **1f** (161 mg, 0.5 mmol), the crude product was purified by column chromatography (hexane/EtOAc, 10/1) affording pure **2f** as an orange oil (133 mg, 83%). R_f = 0.36 (hexane/EtOAc, 10/1). **^{1}H NMR** (300 MHz, $CDCl_3$): δ (ppm) = 7.91 (d, *J* = 8.1 Hz, 1H), 7.34 (d, *J* = 7.6 Hz, 1H), 7.30–7.26 (m, 1H), 7.25–7.18 (m, 1H), 5.63 (s, 1H), 5.19 (s, 1H), 5.09 (d, *J* = 2.5 Hz, 1H), 3.73 (s, 3H), 2.59–2.45 (m, 1H), 1.43–1.31 (m, 1H), 1.20 (d, *J* = 6.8 Hz, 3H), 1.14–1.03 (m, 1H), 0.95–0.87 (m, 1H), 0.70 (d, *J* = 6.8 Hz, 3H), 0.66–0.57 (m, 1H), 0.55–0.43 (m, 2H), 0.43–0.32 (m, 2H), 0.28–0.18 (m, 1H), 0.06– -0.06 (m, 1H). **^{13}C NMR** (75.4 MHz, $CDCl_3$): δ (ppm) = 142.0 (C), 138.5 (C), 138.1 (C), 124.7 (C), 121.6 (CH), 120.3 (CH), 120.2 (CH), 110.5 (C), 109.0 (CH), 104.2 (CH_2), 75.9 (C), 71.3 (CH), 32.1 (CH), 31.6 (CH_3), 19.6 (CH), 17.7 (CH), 17.2 (CH_3), 15.4 (CH_3), 3.3 (CH_2), 1.4 (CH_2), 0.7 (CH_2), 0.2 (CH_2). **HRMS** (ESI-TOF) *m/z*: $[M+H]^+$ Calcd for $C_{22}H_{28}NO^+$ 322.2165; Found 322.2172.

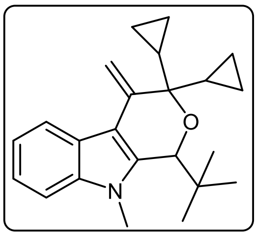

1-(Tert-butyl)-3,3-dicyclopropyl-9-methyl-4-methylene-1,3,4,9-tetrahydropyrano[3,4-*b*]indole (2g): Following the general procedure with **1g** (165 mg, 0.5 mmol), the crude product was purified by column chromatography (hexane/EtOAc, 10/1) affording pure **2g** as a yellow oil (108 mg, 65%). R_f = 0.33 (hexane/EtOAc, 15/1). **^{1}H NMR** (500 MHz, $CDCl_3$): δ (ppm) = 7.90 (d, *J* = 7.7 Hz, 1H), 7.38 (d, *J* = 8.1 Hz, 1H), 7.34–7.29 (m, 1H), 7.28–7.22 (m, 1H), 5.54 (s, 1H), 5.38 (s, 1H), 4.77 (s, 1H), 3.69 (s, 3H), 1.34–1.26 (m, 1H), 1.14–1.08 (m, 1H), 0.98 (s, 9H), 0.95–0.83 (m, 1H), 0.76–0.69 (m, 1H), 0.61–0.50 (m, 2H), 0.29–0.17 (m, 2H), 0.10–0.03 (m, 1H), -0.08– -0.16 (m, 1H). **^{13}C NMR** (125.7 MHz, $CDCl_3$): δ (ppm) = 143.0 (C), 139.7 (C), 137.3 (C) 125.2 (C), 121.8 (CH), 120.3 (CH), 119.5 (CH), 112.2 (C), 109.7 (CH), 104.9 (CH_2), 76.8 (C), 76.2 (CH), 39.7 (C), 32.9 (CH_3), 26.6 (3 × CH_3), 19.4 (CH), 17.5 (CH), 2.3 (CH_2), 2.0 (CH_2), 1.7 (CH_2), 0.5 (CH_2). **HRMS** (ESI-TOF) *m/z*: $[M+H]^+$ Calcd for $C_{23}H_{30}NO^+$ 336.2322; Found 336.2332.

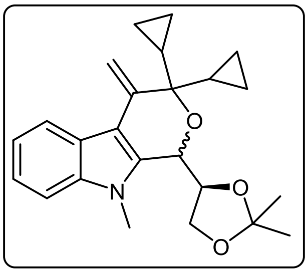

3,3-Dicyclopropyl-1-((*S*)-2,2-dimethyl-1,3-dioxolan-4-yl)-9-methyl-4-methylene-1,3,4,9-tetrahydropyrano[3,4-*b*]indole (2h): Following the general procedure with **1h** (189 mg, 0.5 mmol), the crude product was purified by column chromatography (hexane/EtOAc, 10/1) affording pure **2h** as a yellow oil (135 mg, 71%). R_f = 0.25 (hexane/EtOAc, 10/1). Obtained and isolated as a ca. 3/1 mixture of diasteroisomers. Data for major diasteroisomers: **^{1}H NMR** (300 MHz, $(CD_3)_2CO$): δ (ppm) = 7.86 (d, *J* = 7.7 Hz, 1H), 7.45 (d, *J* = 8.0 Hz, 1H), 7.30–7.11 (m, 2H), 5.61 (s, 1H), 5.45 (d, *J* = 2.8 Hz, 1H), 5.21 (s, 1H), 4.97 (td, *J* = 6.8, 2.8 Hz, 1H), 4.12–3.98 (m, 2H), 3.87 (s, 3H), 1.45 (s, 3H), 1.24 (s, 3H), 1.14–1.01 (m, 1H), 0.93–0.82 (m, 1H), 0.66–0.55 (m, 1H), 0.54–0.28 (m, 5H), 0.26–0.14 (m, 1H), 0.01– -0.11 (m, 1H). **^{13}C NMR** (75.4 MHz, $(CD_3)_2CO$): δ (ppm) = 141.6 (C), 138.6 (C), 135.6 (C), 124.4 (C), 121.6 (CH), 120.0 (CH), 119.7 (CH), 109.7 (C), 109.5 (CH), 109.1 (C), 104.0 (CH_2), 76.9 (C), 76.5 (CH), 67.5 (CH), 64.7 (CH_2), 31.3 (CH_3), 25.5 (CH_3), 24.7 (CH_3), 17.6 (CH), 16.8 (CH), 2.5 (CH_2), 1.3 (CH_2), 0.1 (CH_2), 0.0 (CH_2). **HRMS** (ESI-TOF) *m/z*: $[M+H]^+$ Calcd for $C_{24}H_{30}NO_3^+$ 380.2220; Found 380.2222.

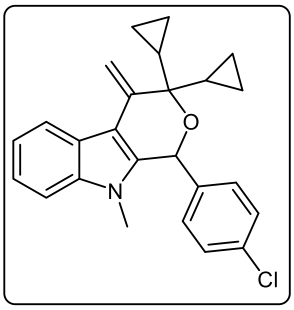

1-(4-Chlorophenyl)-3,3-dicyclopropyl-9-methyl-4-methylene-1,3,4,9-tetrahydropyrano[3,4-*b*]indole (2i): Following the general procedure with **1i** (195 mg, 0.5 mmol), the crude product was purified by column chromatography (hexane/EtOAc, 10/1) affording pure **2i** as a yellow oil (151 mg, 80%). R_f = 0.29 (hexane/EtOAc, 10/1). **^{1}H NMR** (300 MHz, $CDCl_3$): δ (ppm) = 7.93 (d, *J* = 8.5 Hz, 1H), 7.37–7.29 (m, 3H), 7.29–7.16 (m, 4H), 6.06 (s, 1H), 5.72 (s, 1H), 5.25 (s, 1H), 3.15 (s, 3H), 1.37–1.22 (m, 1H), 1.20–1.04 (m, 1H), 0.94–0.83 (m, 1H), 0.76–0.60 (m, 1H), 0.52–0.27 (m, 5H), 0.17–0.02 (m, 1H). **^{13}C NMR** (75.4 MHz, $CDCl_3$): δ (ppm) = 141.0 (C), 139.3 (C), 138.3 (C), 136.8 (C), 134.4 (C), 130.6 (2 × CH), 129.0 (2 × CH), 124.2 (C), 122.0 (CH), 120.7 (CH), 120.5 (CH), 110.2 (C), 109.2 (CH), 105.4 (CH_2), 79.0 (C), 71.0 (CH), 31.0 (CH_3), 18.4 (CH), 16.1 (CH), 2.8 (CH_2), 2.5 (CH_2), 0.6 (2 × CH_2). **HRMS** (ESI-TOF) *m/z*: $[M+H]^+$ Calcd for $C_{25}H_{25}ClNO^+$ 390.1619; Found 390.1624.

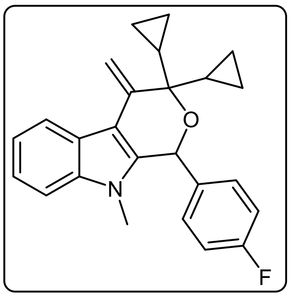

3,3-Dicyclopropyl-1-(4-fluorophenyl)-9-methyl-4-methylene-1,3,4,9-tetrahydropyrano[3,4-*b*]indole (2j): Following the general procedure with **1j** (136 mg, 0.5 mmol), the crude product was purified by column chromatography (hexane/EtOAc, 5/1) affording pure **2j** as an orange oil (126 mg, 68%). R_f = 0.31 (hexane/EtOAc, 5/1). **^{1}H NMR** (300 MHz, $(CD_3)_2CO$): δ (ppm) = 7.94 (d, *J* = 8.3 Hz, 1H), 7.46–7.30 (m, 3H), 7.27–7.09 (m, 4H), 6.27 (s, 1H), 5.73 (s, 1H), 5.26 (s, 1H), 3.20 (s, 3H), 1.41–1.32 (m, 1H), 1.25–1.13 (m, 1H), 0.75–0.60 (m, 1H), 0.49–0.25 (m, 6H), 0.16– -0.03 (m, 1H). **^{13}C NMR** (75.4 MHz, $(CD_3)_2CO$): δ (ppm) = 162.7 (C, d, *J* = 245.3 Hz), 141.4 (C), 138.4 (C), 137.3 (C), 137.10 (C, d, *J* = 3.2 Hz), 131.1 (2 × CH, d, *J* = 8.4 Hz), 124.1 (C), 121.8 (CH), 120.3 (CH), 120.1 (CH), 115.3 (2 × CH, d, *J* = 21.6 Hz), 109.7 (C), 109.4 (CH), 104.4 (CH_2), 78.3 (C), 70.6 (CH), 30.3 (CH_3), 17.9 (CH), 16.1 (CH), 2.4 (CH_2), 1.7 (CH_2), 0.1 (CH_2), 0.0 (CH_2). **HRMS** (ESI-TOF) *m/z*: $[M+H]^+$ Calcd for $C_{25}H_{25}NFO^+$ 374.1915; Found 374.1913.

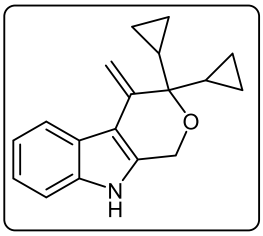

3,3-Dicyclopropyl-4-methylene-1,3,4,9-tetrahydropyrano[3,4-*b*]indole (2k): Following the general procedure with **1k** (132 mg, 0.5 mmol), the crude product was purified by column chromatography (hexane/EtOAc, 3/1) affording pure **2k** as a white solid (107 mg, 81%). R_f = 0.25 (hexane/EtOAc, 3/1). M.p. = 109–111 °C. **^{1}H NMR** (300 MHz, $CDCl_3$): δ (ppm) = 7.91 (br s, 1H), 7.90–7.84 (m, 1H), 7.34–7.27 (m, 1H), 7.25–7.13 (m, 2H), 5.69 (s, 1H), 5.25 (s, 1H), 4.97 (s, 2H), 1.24–1.11 (m, 2H), 0.55–0.37 (m, 8H). **^{13}C NMR** (75.4 MHz, $CDCl_3$): δ (ppm) = 140.4 (C), 136.6 (C), 134.7 (C), 124.9 (C), 122.1 (CH), 120.7 (CH), 120.5 (CH), 111.3 (CH), 109.6 (C), 106.0 (CH_2), 79.3 (C), 58.8 (CH_2), 16.6 (2 × CH), 2.4 (2 × CH_2), -0.1 (2 × CH_2). **HRMS** (ESI-TOF) *m/z*: $[M+H]^+$ Calcd for $C_{18}H_{20}NO^+$ 266.1539; Found 266.1542. **2k** could also be obtained in a 2-mmol scale as a white solid (397 mg, 75%), starting from **1k** (528 mg, 2 mmol).

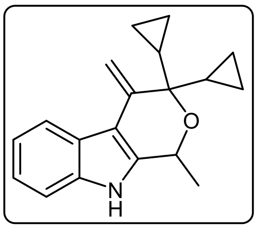

3,3-Dicyclopropyl-1-methyl-4-methylene-1,3,4,9-tetrahydropyrano[3,4-*b*]indole (2l): Following the general procedure with **1l** (140 mg, 0.5 mmol), the crude product was purified by column chromatography (hexane/EtOAc, 3/1) affording pure **2l** as a brown oil (98 mg, 70%). R_f = 0.23 (hexane/EtOAc, 3/1). **^{1}H NMR** (300 MHz, $(CD_3)_2CO$): δ (ppm) = 10.26 (br s, 1H), 7.83 (d, *J* = 9.1 Hz, 1H), 7.43–7.33 (m, 1H), 7.18–7.05 (m, 3H), 5.61 (s, 1H), 5.36 (q, *J* = 6.5 Hz, 1H), 5.14

(s, 1H), 1.53 (d, J = 6.5 Hz, 3H), 1.48–1.38 (m, 1H), 1.13–1.01 (m, 1H), 0.91–0.80 (m, 1H), 0.59–0.44 (m, 2H), 0.43–0.24 (m, 4H), 0.15–0.01 (m, 1H). **^{13}C NMR** (75.4 MHz, $(CD_3)_2CO$): δ (ppm) = 141.6 (C), 139.3 (C), 137.1 (C), 124.8 (C), 121.4 (CH), 120.0 (CH), 119.9 (CH), 111.4 (CH), 108.4 (C), 103.6 (CH_2), 77.7 (C), 63.5 (CH), 19.3 (CH_3), 18.5 (CH), 15.8 (CH), 2.1 (CH_2), 1.5 (CH_2), -0.2 (CH_2), -0.7 (CH_2). **HRMS** (ESI-TOF) *m/z*: $[M+H]^+$ Calcd for $C_{19}H_{22}NO^+$ 280.1696; Found 280.1693.

***General procedure for the synthesis of tetrahydropyrano[3,4-b]indol 2o-q and 1,3-dien-2-yl-indol* derivatives 7p,q**

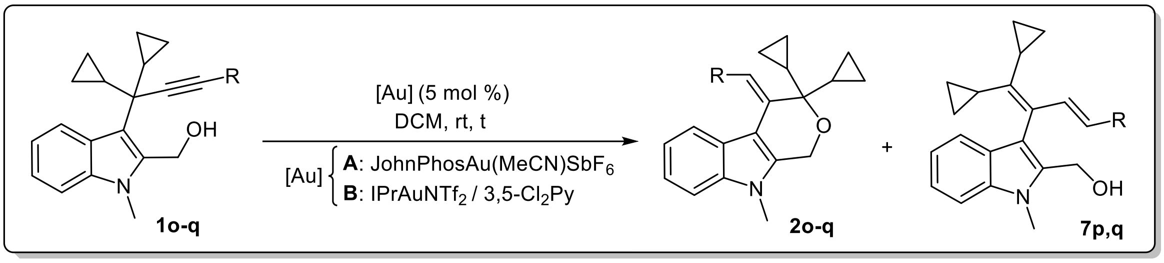

The corresponding catalytic system: **A** [JohnPhosAu(MeCN)SbF_6 (19 mg, 0.025 mmol, 0.05 equiv); **B** [IPrAuNTf_2 (21 mg, 0.025 mmol, 0.05 equiv) and 3,5-dichloropyridine (0.25 mmol, 36 mg, 0.5 equiv) was dissolved in DCM (1 mL), and the resulting solution was stirred at rt for 5 min. A solution of the corresponding 3-propargyl indole **1o-q** (0.5 mmol, 1 equiv) in DCM (1 mL) was then added. The reaction mixture was stirred at rt for 30 min (until complete consumption of the starting material, as determined by GC–MS or TLC). The mixture was filtered through a short pad of silica gel and celite using a 2/1 (hexane/EtOAc) mixture as eluent. The solvents were removed under reduced pressure, and the crude product was purified by flash column chromatography using mixtures of hexane/EtOAc as eluents, affording the corresponding tetrahydropyrano[3,4-*b*]indoles **2o-q** or 1,3-dien-2-yl-indol derivatives **7p,q**.

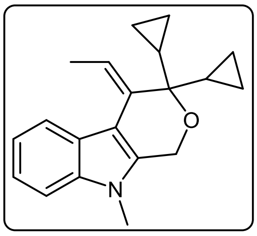

(*E*)-3,3-Dicyclopropyl-4-ethylidene-9-methyl-1,3,4,9-tetrahydropyrano[3,4-*b*]indole (2o): Following the general procedure using catalytic system A with **1o** (146 mg, 0.5 mmol) for 1 h, the crude product was purified by column chromatography (hexane/EtOAc, 4/1) affording pure **2o** as a yellow solid (88 mg , 60%). R_f = 0.34 (hexane/EtOAc, 4/1). M.p. = 100–102 °C. Obtained and isolated as a >20/1 mixture of *E/Z* isomers. **^{1}H NMR** (300 MHz, $CDCl_3$): δ (ppm) = 7.68 (d, J = 7.8 Hz, 1H), 7.33 (d, J = 7.7 Hz, 1H), 7.28–7.21 (m, 1H), 7.21–7.14 (m, 1H), 5.96 (q, J = 7.2 Hz, 1H), 5.04 (s, 2H), 3.64 (s, 3H), 2.14 (d, J = 7.2 Hz, 3H), 1.13–0.99 (m, 2H), 0.54–0.28 (m, 8H). **^{13}C NMR** (75.4 MHz, $CDCl_3$): δ (ppm) = 137.8 (C), 134.9 (C), 132.7 (C), 125.6 (C), 121.8 (CH), 121.1 (CH), 119.2 (CH), 118.4 (CH), 109.0 (C), 108.8 (CH), 78.2 (C), 59.2 (CH_2), 29.5 (CH_3), 17.5 (CH_3), 16.7 (2 × CH), 2.5 (2 × CH_2), 0.8 (2 x CH_2). **HRMS** (ESI-TOF) *m/z*: $[M+H]^+$ Calcd for $C_{20}H_{24}NO^+$ 294.1852; Found 294.1860.

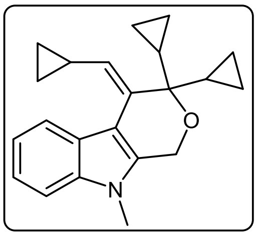

(*E*)-3,3-Dicyclopropyl-4-(cyclopropylmethylene)-9-methyl-1,3,4,9-tetrahydropyrano[3,4-*b*]indole (2p): Following the general procedure using catalytic system B with **1p** (160 mg, 0.5 mmol) for 3 h, the crude product was purified by column chromatography (hexane/EtOAc, 4/1) affording pure **2p** as a yellow oil (134 mg, 84%). R_f = 0.20 (hexane/EtOAc, 5/1). Obtained and isolated as a >20/1 mixture of *E/Z* isomers. **^{1}H NMR** (300 MHz, $CDCl_3$): δ (ppm) = 8.00 (d, *J* = 7.9 Hz, 1H), 7.32 (d, *J* = 7.9 Hz, 1H), 7.26–7.18 (m, 1H), 7.18–7.11 (m, 1H), 5.20 (d, *J* = 10.0 Hz, 1H), 5.04 (s, 2H), 3.64 (s, 3H), 2.28–1.93 (m, 1H), 1.18–0.91 (m, 4H), 0.63–0.52 (m, 2H), 0.49–0.25 (m, 8H). **^{13}C NMR** (75.4 MHz, $CDCl_3$): δ (ppm) = 137.9 (C), 134.7 (C), 130.1 (C), 128.7 (CH), 125.7 (C), 121.6 (CH), 121.1 (CH), 119.2 (CH), 109.3 (C), 108.8 (CH), 77.9 (C), 59.2 (CH_2), 29.5 (CH_3), 16.7 (2 × CH), 13.9 (CH), 8.7 (2 × CH_2), 2.5 (2 x CH_2), 0.8 (2 × CH_2). **HRMS** (ESI-TOF) *m/z*: $[M+H]^+$ Calcd for $C_{22}H_{26}NO^+$ 320.2009; Found 320.2010.

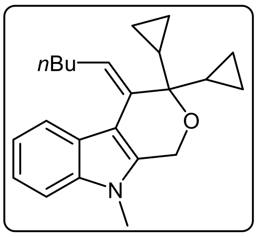

(*E*)-3,3-Dicyclopropyl-9-methyl-4-pentylidene-1,3,4,9-tetrahydropyrano[3,4-*b*]indole (2q): Following the general procedure using catalytic system B with **1q** (168 mg, 0.5 mmol) for 1 h, a ca. 2.5/1 mixture of **7q**/**2q** was obtained. The crude product was purified by column chromatography (hexane/EtOAc, 5/1) affording pure **2q** as a yellow oil (42 mg, 25%). R_f = 0.30 (hexane/EtOAc, 5/1). Obtained and isolated as a >20/1 mixture of *E/Z* isomers. **^{1}H NMR** (300 MHz, $CDCl_3$): δ (ppm) = 7.64 (d, *J* = 7.8 Hz, 1H), 7.32 (d, *J* = 7.9 Hz, 1H), 7.27–7.19 (m, 1H), 7.19–7.12 (m, 1H), 5.80 (t, *J* = 7.2 Hz, 1H), 5.02 (s, 2H), 3.63 (s, 3H), 2.65–2.47 (m, 2H), 1.58–1.46 (m, 2H), 1.46–1.31 (m, 2H), 1.14–0.99 (m, 2H), 0.92 (t, *J* = 7.2 Hz, 3H), 0.51–0.26 (m, 8H). **^{13}C NMR** (75.4 MHz, $CDCl_3$): δ (ppm) = 137.8 (C), 135.0 (C), 131.0 (C), 125.7 (C), 125.0 (CH), 121.5 (CH), 121.1 (CH), 119.2 (CH), 109.3 (C), 108.8 (CH), 78.2 (C), 59.2 (CH_2), 33.1 (CH_2), 31.0 (CH_2), 29.5 (CH_3), 22.6 (CH_2), 16.8 (2 × CH), 14.3 (CH_3), 2.6 (2 × CH_2), 0.9 (2 × CH_2). **HRMS** (ESI-TOF) *m/z*: $[M+H]^+$ Calcd for $C_{23}H_{30}NO^+$ 336.2322; Found 336.2322.

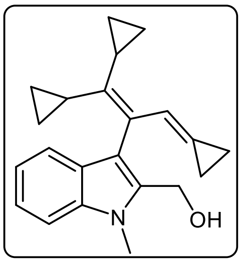

(3-(1,1-Dicyclopropyl-3-cyclopropylideneprop-1-en-2-yl)-1-methyl-1*H*-indol-2-yl)methanol (7p): Following the general procedure using catalytic system A with **1p** (160 mg, 0.5 mmol) for 30 min, the crude product was purified by column chromatography (hexane/EtOAc, 3/1) affording pure **7p** as a yellow solid (102 mg, 64%). R_f = 0.33 (hexane/EtOAc, 3/1). M.p. = 65–67 °C. **^{1}H NMR** (500 MHz, $(CD_3)_2CO$): δ (ppm) = 7.36 (d, *J* = 8.2 Hz, 1H), 7.32 (d, *J* = 7.9 Hz, 1H), 7.14 (at, *J* = 7.6 Hz, 1H), 6.99 (at, *J* = 7.4 Hz, 1H), 5.67 (s, 1H), 4.71 (d, *J* = 12.8, Hz, 1H), 4.69 (d, *J* = 12.8, Hz, 1H), 3.93 (t, *J* = 5.1 Hz, 1H), 3.88 (s, 3H), 2.84–2.71 (m, 2H), 2.38–2.28 (m, 2H), 1.93–1.81 (m, 1H), 1.23–1.11 (m, 1H), 0.88–0.71 (m, 4H), 0.36–0.11 (m, 4H). **^{13}C NMR** (125.7 MHz, $(CD_3)_2CO$): δ (ppm) = 148.6 (C), 142.0 (C), 137.3 (C), 136.3 (C), 130.7 (CH), 127.7 (C), 127.6 (C), 121.2 (CH), 119.9 (CH), 118.7 (CH), 113.4 (C), 108.9 (CH), 54.6 (CH_2), 33.1 (CH_2), 29.6 (CH_3), 26.5 (CH_2), 14.8 (CH), 13.9 (CH), 7.1 (CH_2), 6.8 (CH_2), 6.4 (CH_2), 5.8 (CH_2). **HRMS** (ESI-TOF) *m/z*: $[M+H]^+$ Calcd for $C_{22}H_{26}NO^+$ 320.2009; Found 320.2010.

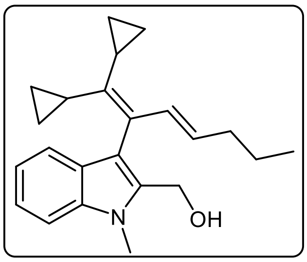

(*E*)-(3-(1,1-Dicyclopropylhepta-1,3-dien-2-yl)-1-methyl-1*H*-indol-2-yl)methanol (7q): Following the general procedure using catalytic system A with **1q** (168 mg, 0.5 mmol) for 1 h, the crude product was purified by column chromatography (hexane/EtOAc, 3/1) affording pure **7q** as a yellow oil (123 mg, 77%). R_f = 0.35 (hexane/EtOAc, 3/1). Obtained and isolated as a >20/1 mixture of *E/Z* isomers. **^{1}H NMR** (300 MHz, $CDCl_3$): δ (ppm) = 7.42–7.34 (m, 2H), 7.29–7.21 (m, 1H), 7.14–7.04 (m, 2H), 5.22–5.04 (m, 1H), 4.77–4.58 (m, 2H), 3.89 (s, 3H), 2.05 (q, *J* = 7.0 Hz, 2H), 1.61 (br s, 1H), 1.48–1.20 (m, 4H), 0.91–0.81 (m, 4H), 0.77–0.65 (m, 2H), 0.65–0.52 (m, 2H), 0.42–0.27 (m, 2H). **^{13}C NMR** (75.4 MHz, $CDCl_3$): δ (ppm) = 140.0 (C), 137.5 (C), 135.6 (C), 131.8 (CH), 131.4 (CH), 130.2 (C), 127.5 (C), 121.9 (CH), 120.9 (CH), 119.1 (CH), 114.7 (C), 109.0 (CH), 55.7 (CH_2), 35.3 (CH_2), 30.0 (CH_3), 22.8 (CH_2), 16.7 (CH), 13.9 (CH), 11.3 (CH_3), 6.5 (CH_2), 6.4 (CH_2), 6.3 (CH_2), 6.1 (CH_2). **HRMS** (ESI-TOF) *m/z*: $[M+H]^+$ Calcd for $C_{22}H_{28}NO^+$ 322.2165; Found 322.2168.

Synthesis and characterization data of tetrahydrooxepino[4,5-*b*]indol derivatives 4 and 1,3-dien-2-yl-indol derivatives 8

General procedure for the synthesis of tetrahydrooxepino[4,5-b]indol derivatives 4a-m

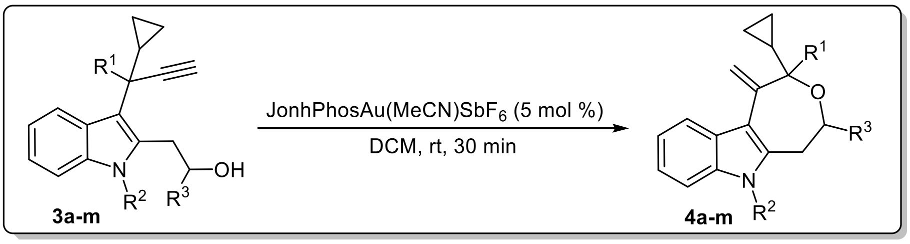

JonhPhosAu(MeCN)SbF_6 (19.5 mg, 0.025 mmol, 0.05 equiv) was dissolved in DCM (1 mL), and the resulting solution was stirred at rt for 5 min. A solution of the corresponding 3-propargyl indole **3a-m** (0.5 mmol, 1 equiv) in DCM (1 mL) was then added. The reaction mixture was stirred at rt for 30 min (until complete consumption of the starting material, as determined by GC–MS or TLC). The mixture was filtered through a short pad of silica gel and celite using a 2/1 (hexane/EtOAc) mixture as eluent. The solvents were removed under reduced pressure, and the crude product was purified by flash column chromatography using mixtures of hexane/EtOAc as eluents, affording the corresponding tetrahydrooxepino[4,5-*b*]indoles **4a-m**.

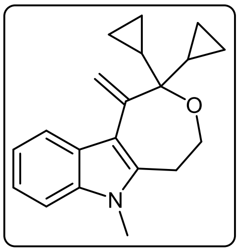

2,2-Dicyclopropyl-6-methyl-1-methylene-1,4,5,6-tetrahydro-2*H*-oxepino[4,5-*b*]indole (4a): Following the general procedure with **3a** (146 mg, 0.5 mmol), the crude product was purified by column chromatography (hexane/EtOAc, 5/1) affording pure **4a** as a white solid (101 mg, 69%). R_f = 0.35 (hexane/EtOAc, 3/1). M.p. = 143–145 °C. **^{1}H NMR** (300 MHz, $CDCl_3$): δ (ppm) = 7.75 (d, *J* = 7.8 Hz, 1H), 7.32 (d, *J* = 7.7 Hz, 1H), 7.26 (at, *J* = 7.3 Hz, 1H), 7.19 (at, *J* = 7.3 Hz, 1H), 5.80 (s, 1H), 5.57 (s, 1H), 4.18 (t, *J* = 5.3 Hz, 2H), 3.67 (s, 3H), 3.04 (t, *J* = 5.3 Hz, 2H), 1.20–0.93 (m, 2H), 0.68–0.48 (m, 4H), 0.48–0.23 (m, 4H). **^{13}C NMR** (75.4 MHz, $CDCl_3$): δ (ppm) = 145.6 (C), 137.2 (C), 135.0 (C), 127.2 (C), 121.0 (CH), 119.4 (CH), 119.1 (CH), 115.8 (CH_2), 113.2 (C), 108.6 (CH), 86.0

(C), 61.5 (CH_2), 30.9 (CH_2), 29.6 (CH_3), 18.0 (2 × CH), 1.7 (2 × CH_2), 0.8 (2 × CH_2). **HRMS** (ESI-TOF) *m/z*: $[M+H]^+$ Calcd for $C_{20}H_{24}NO^+$ 294.1852; Found 294.1859.

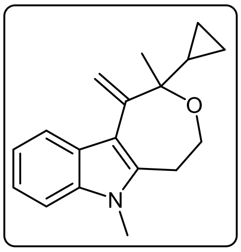

2-Cyclopropyl-2,6-dimethyl-1-methylene-1,4,5,6-tetrahydro-2*H*-oxepino[4,5-*b*]indole (4b): Following the general procedure with **3b** (133 mg, 0.5 mmol), the crude product was purified by column chromatography (hexane/EtOAc, 3/1) affording pure **4b** as a yellow oil (87 mg, 65%). R_f = 0.25 (hexane/EtOAc, 3/1). **^{1}H NMR** (300 MHz, $CDCl_3$): δ (ppm) = 7.72 (d, *J* = 7.2 Hz, 1H), 7.30 (d, *J* = 7.4 Hz, 1H), 7.27–7.20 (m, 1H), 7.20–7.10 (m, 1H), 5.51 (d, *J* = 1.4 Hz, 1H), 5.45 (d, *J* = 1.4 Hz, 1H), 4.22–4.05 (m, 2H), 3.65 (s, 3H), 3.20–2.92 (m, 2H), 1.34 (s, 3H), 1.29–1.14 (m, 1H), 0.58–0.34 (m, 4H). **^{13}C NMR** (75.4 MHz, $CDCl_3$): δ (ppm) = 146.4 (C), 137.1 (C), 135.3 (C), 127.4 (C), 121.0 (CH), 119.5 (CH), 119.0 (CH), 114.7 (CH_2), 112.3 (C), 108.6 (CH), 85.6 (C), 60.8 (CH_2), 30.7 (CH_2), 29.6 (CH_3), 24.1 (CH_3), 20.4 (CH), 2.1 (CH_2), 1.4 (CH_2). **HRMS** (ESI-TOF) *m/z*: $[M+H]^+$ Calcd for $C_{18}H_{22}NO^+$ 268.1696; Found 268.1701.

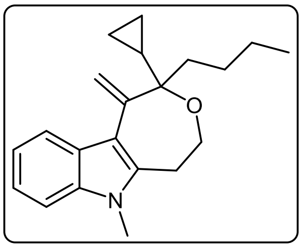

2-Butyl-2-cyclopropyl-6-methyl-1-methylene-1,4,5,6-tetrahydro-2H-oxepino[4,5-*b*]indole (4c): Following the general procedure with **3c** (154 mg, 0.5 mmol), the crude product was purified by column chromatography (hexane/EtOAc, 5/1) affording pure **4c** as a purple solid (138 mg, 89%). R_f = 0.30 (hexane/EtOAc, 5/1). M.p. = 80–82 °C. **^{1}H NMR** (300 MHz, $CDCl_3$): δ (ppm) = 7.76 (d, *J* = 7.6 Hz, 1H), 7.33 (d, *J* = 7.4 Hz, 1H), 7.27 (at, *J* = 7.3 Hz, 1H), 7.21 (at, *J* = 7.3 Hz, 1H), 5.66 (s, 1H), 5.60 (s, 1H), 4.21 (dt, *J* = 12.2, 4.8 Hz, 1H), 4.04 (dt, *J* = 12.2, 4.8 Hz, 1H), 3.66 (s, 3H), 3.12–2.88 (m, 2H), 1.81–1.62 (m, 2H), 1.58–1.41 (m, 2H), 1.39–1.23 (m, 3H), 0.93 (t, *J* = 7.3 Hz, 3H), 0.68–0.36 (m, 4H). **^{13}C NMR** (75.4 MHz, $CDCl_3$): δ (ppm) = 145.0 (C), 137.2 (C), 135.1 (C), 127.3 (C), 121.0 (CH), 119.4 (CH), 119.0 (CH), 115.9 (CH_2), 112.8 (C), 108.6 (CH), 86.6 (C), 61.7 (CH_2), 40.1 (CH_2), 31.0 (CH_2), 29.5 (CH_3), 26.6 (CH_2), 23.3 (CH_2), 17.4 (CH), 14.2 (CH_3), 1.2 (CH_2), 0.9 (CH_2). **HRMS** (ESI-TOF) *m/z*: $[M+H]^+$ Calcd for $C_{21}H_{28}NO^+$ 310.2165; Found 310.2167.

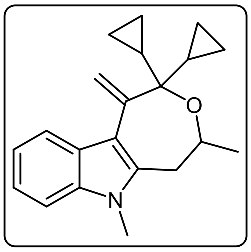

2,2-Dicyclopropyl-4,6-dimethyl-1-methylene-1,4,5,6-tetrahydro-2*H*-oxepino[4,5-*b*]indole (4d): Following the general procedure with **3d** (154 mg, 0.5 mmol), the crude product was purified by column chromatography (hexane/EtOAc, 10/1) affording pure **4d** as a white solid (135 mg, 88%). R_f = 0.27 (hexane/EtOAc, 10/1). M.p. = 170–172 °C. **^{1}H NMR** (500 MHz, $CDCl_3$): δ (ppm) = 7.71 (d, *J* = 7.8 Hz, 1H), 7.29 (d, *J* = 8.6 Hz, 1H), 7.26–7.19 (m, 1H), 7.19–7.13 (m, 1H), 5.72 (d, *J* = 1.8 Hz, 1H), 5.52 (d, *J* = 1.7 Hz, 1H), 4.55–4.41 (m, 1H), 3.65 (s, 3H), 2.93 (d, *J* = 7.1 Hz, 2H), 1.41 (d, *J* = 6.3 Hz, 3H), 1.39–1.34 (m, 1H), 0.98–0.83 (m, 1H), 0.79–0.74 (m, 1H), 0.74–0.67 (m, 1H), 0.66–0.59 (m, 1H), 0.59–0.51 (m, 1H), 0.46–0.37 (m, 1H), 0.25–0.14 (m, 2H), 0.14–0.05 (m, 1H). **^{13}C NMR** (125.7 MHz, $CDCl_3$): δ (ppm) = 146.0 (C), 137.1 (C), 135.2 (C), 127.1 (C), 121.0 (CH), 119.4 (CH), 119.1 (CH), 115.3 (CH_2), 113.0 (C), 108.6 (CH), 85.5 (C), 67.0 (CH), 37.6 (CH_2), 29.6 (CH_3), 22.7 (CH_3), 18.1 (CH), 17.9 (CH), 3.6 (CH_2), 1.6 (CH_2), 0.5 (CH_2), 0.3 (CH_2). **HRMS** (ESI-TOF) *m/z*: $[M+H]^+$ Calcd for $C_{21}H_{26}NO^+$ 308.2009; Found 308.2015.

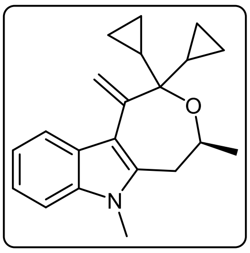

(*S*)-2,2-Dicyclopropyl-4,6-dimethyl-1-methylene-1,4,5,6-tetrahydro-2*H*-oxepino[4,5-*b*]indole ((*S*)-4d): Following the general procedure with **(*S*)-3d** (154 mg, 0.5 mmol), the crude product was purified by column chromatography (hexane/EtOAc, 10/1) affording pure **(*S*)-4d** as a white solid (120 mg,78%). **HRMS** (ESI-TOF) *m/z*: calcd for $C_{21}H_{26}NO^+$ $[M+H]^+$ 308.2009; found 308.2017. **Column:** Chiralpak OD-H; **Flow:** 0.5 mL/min; **Eluent:** hex/*i*-PrOH 95/5; 100% ee; $[\alpha]_D^{25}$ = –96.1 (c 0.01038 g/mL in acetone).

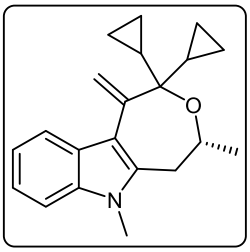

(*R*)-2,2-Dicyclopropyl-4,6-dimethyl-1-methylene-1,4,5,6-tetrahydro-2*H*-oxepino[4,5-*b*]indole ((*R*)-4d): Following the general procedure with **(*R*)-3d** (154 mg, 0.5 mmol), the crude product was purified by column chromatography (hexane/EtOAc, 10/1) affording pure **(*R*)-4d** as a white solid (115 mg, 75%). **HRMS** (ESI-TOF) *m/z*: calcd for $C_{21}H_{26}NO^+$ $[M+H]^+$ 308.2009; found 308.2012. **Column:** Chiralpak OD-H; **Flow:** 0.5 mL/min; **Eluent:** hex/*i*-PrOH 95/5; 100% ee; $[\alpha]_D^{25}$ = 94.2 (c 0.00478 g/mL in acetone).

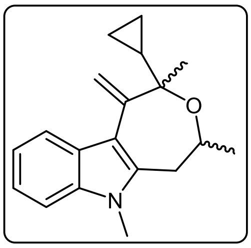

2-Cyclopropyl-2,4,6-trimethyl-1-methylene-1,4,5,6-tetrahydro-2*H*-oxepino[4,5-*b*]indole (4e): Following the general procedure with **3e** (142 mg, 0.5 mmol), the crude product was purified by column chromatography (hexane/EtOAc, 10/1) affording pure **4e** as a yellow solid (99 mg , 71%). R_f = 0.29 (hexane/EtOAc, 10/1). M.p. = 110–112 °C. Obtained as a ca. 1/1 mixture of diasteroisomers and isolated as a ca. 1.3/1. Data for both diasteroisomers: **^{1}H NMR** (300 MHz, $CDCl_3$): *δ* (ppm) = 7.80–7.69 (m, 2H), 7.36–7.28 (m, 2H), 7.28–7.23 (m, H), 7.23–7.15 (m, 2H), 5.84 (s, 1H), 5.47 (s, 1H), 5.43 (s, 1H), 5.26 (s, 1H), 4.51–4.33 (m, 2H), 3.66 (s, 3H), 3.65 (s, 3H), 3.05–2.85 (m, 4H), 1.56 (s, 3H), 1.44 (t, *J* = 5.9 Hz, 6H), 1.15–1.02 (m, 2H), 1.06 (s, 3H), 0.79–0.65 (m, 2H), 0.64–0.52 (m, 2H), 0.50–0.31 (m, 2H), 0.30–0.16 (m, 2H). **^{13}C NMR** (75.4 MHz, $CDCl_3$): *δ* (ppm) = 148.0 (C), 146.1 (C), 137.0 (2 × C), 135.5 (C), 135.4 (C), 127.7 (C), 127.2 (C), 121.0 (CH), 120.9 (CH), 119.43 (CH), 119.41 (CH), 119.1 (CH), 119.0 (CH), 114.1 (CH_2), 113.7 (CH_2), 112.3 (C), 112.0 (C), 108.61 (CH), 108.60 (CH), 85.2 (C), 84.0 (C), 67.2 (CH), 66.4 (CH), 37.8 (CH_2), 37.3 (CH_2), 29.6 (2 × CH_3), 25.3 (CH_3), 23.4 (CH_3), 23.2 (CH), 22.9 (CH), 21.7 (CH_3), 18.4 (CH_3), 3.3 (CH_2), 1.7 (2 × CH_2), 0.8 (CH_2). **HRMS** (ESI-TOF) *m/z*: $[M+H]^+$ Calcd for $C_{19}H_{24}NO^+$ 282.1852; Found 282.1860.

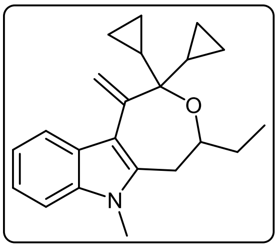

2,2-Dicyclopropyl-4-ethyl-6-methyl-1-methylene-1,4,5,6-tetrahydro-2*H*-oxepino[4,5-*b*]indole (4f): Following the general procedure with **3f** (161 mg, 0.5 mmol), the crude product was purified by column chromatography (hexane/EtOAc, 5/1) affording pure **4f** as a yellow solid (128 mg, 80%). R_f = 0.29 (hexane/EtOAc, 5/1). M.p. = 161–163 °C. **^{1}H NMR** (300 MHz, $CDCl_3$): *δ* (ppm) = 7.73 (d, *J* = 7.5 Hz, 1H), 7.31 (d, *J* = 7.6 Hz, 1H), 7.28–7.23 (m, 1H), 7.23–7.14 (m, 1H), 5.80 (d, *J* = 1.7 Hz, 1H), 5.54 (d, *J* = 1.7 Hz, 1H), 4.34–4.16 (m, 1H), 3.66 (s, 3H), 3.01 (dd, *J* = 16.7, 2.8 Hz, 1H), 2.89 (dd, *J* = 16.7, 10.6 Hz, 1H), 1.91–1.60 (m, 2H), 1.53–1.36 (m, 1H), 1.13 (t, *J* = 7.5 Hz, 3H), 1.03–0.88 (m, 1H), 0.88–0.72 (m, 2H), 0.72–0.64 (m, 1H), 0.65–0.47 (m, 1H), 0.37–0.27 (m, 1H), 0.27–0.16 (m, 1H), 0.16–0.02 (m, 2H). **^{13}C NMR** (75.4 MHz, $CDCl_3$): *δ* (ppm) = 146.2 (C), 137.1 (C), 135.2 (C), 127.1 (C), 120.9 (CH), 119.4 (CH), 119.1 (CH), 115.4 (CH_2), 112.9 (C), 108.5 (CH), 85.3 (C), 71.6 (CH), 35.4 (CH_2), 30.2 (CH_2), 29.5 (CH_3), 19.2 (CH), 16.4 (CH), 10.9 (CH_3), 4.6

(CH_2), 1.1 (2 × CH_2), -0.1 (CH_2). **HRMS** (ESI-TOF) *m/z*: $[M+H]^+$ Calcd for $C_{22}H_{28}NO^+$ 322.2165; Found 322.2165.

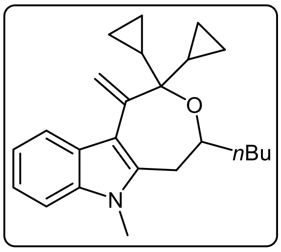

4-Butyl-2,2-dicyclopropyl-6-methyl-1-methylene-1,4,5,6-tetrahydro-2*H*-oxepino[4,5-*b*]indole (4g): Following the general procedure with **3g** (175 mg, 0.5 mmol), the crude product was purified by column chromatography (hexane/EtOAc, 10/1) affording pure **4g** as a white solid (137 mg, 79%). R_f = 0.28 (hexane/EtOAc, 10/1). M.p. = 103–105 °C. **^{1}H NMR** (300 MHz, $CDCl_3$): δ (ppm) = 7.73 (d, J = 7.3 Hz, 1H), 7.29 (d, J = 7.3 Hz, 1H), 7.26–7.20 (m, 1H), 7.20–7.11 (m, 1H), 5.79 (d, J = 1.8 Hz, 1H), 5.54 (d, J = 1.8 Hz, 1H), 4.36–4.24 (m, 1H), 3.63 (s, 3H), 3.05–2.81 (m, 2H), 1.89–1.75 (m, 1H), 1.75–1.58 (m, 2H), 1.53–1.33 (m, 4H), 1.02 (t, J = 7.1 Hz, 3H), 0.99–0.89 (m, 1H), 0.87–0.75 (m, 2H), 0.61–0.50 (m, 1H), 0.3–0.27 (m, 1H), 0.27–0.01 (m, 2H). **^{13}C NMR** (75.4 MHz, $CDCl_3$): δ (ppm) = 146.2 (C), 137.1 (C), 135.2 (C), 127.0 (C), 120.9 (CH), 119.4 (CH), 119.1 (CH), 115.4 (CH_2), 112.9 (C), 108.5 (CH), 85.4 (C), 70.3 (CH), 37.1 (CH_2), 35.7 (CH_2), 29.5 (CH_3), 28.4 (CH_2), 23.0 (CH_2), 19.2 (CH), 16.4 (CH), 14.3 (CH_3), 4.6 (CH_2), 1.2 (2 × CH_2), -0.1 (CH_2). **HRMS** (ESI-TOF) *m/z*: $[M+H]^+$ Calcd for $C_{24}H_{32}NO^+$ 350.2478; Found 350.2486.

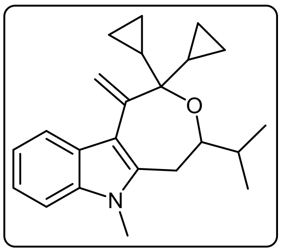

2,2-Dicyclopropyl-4-isopropyl-6-methyl-1-methylene-1,4,5,6-tetrahydro-2*H*-oxepino[4,5-*b*]indole (4h): Following the general procedure with **3h** (168 mg, 0.5 mmol), the crude product was purified by column chromatography (hexane/EtOAc, 10/1) affording pure **4h** as a white solid (122 mg, 73%). R_f = 0.30 (hexane/EtOAc, 10/1). M. p. = 143–145 °C. **^{1}H NMR** (300 MHz, $CDCl_3$): δ (ppm) = 7.73 (d, J = 7.8 Hz, 1H), 7.29 (d, J = 7.4 Hz, 1H), 7.27–7.19 (m, 1H), 7.19–7.13 (m, 1H), 5.82 (d, J = 1.9 Hz, 1H), 5.51 (d, J = 1.9 Hz, 1H), 4.20–4.02 (m, 1H), 3.68–3.62 (m, 3H), 3.00–2.84 (m, 2H), 2.07–1.89 (m, 1H), 1.51–1.38 (m, 1H), 1.11 (t, J = 6.4 Hz, 6H), 0.99–0.88 (m, 1H), 0.88–0.79 (m, 2H), 0.67–0.56 (m, 1H), 0.56–0.47 (m, 1H), 0.34–0.22 (m, 1H), 0.21–0.12 (m, 1H), 0.08– -0.05 (m, 2H). **^{13}C NMR** (75.4 MHz, $CDCl_3$): δ (ppm) = 146.9 (C), 137.2 (C), 135.3 (C), 127.2 (C), 120.9 (CH), 119.3 (CH), 119.2 (CH), 114.9 (CH_2), 112.8 (C), 108.5 (CH), 84.6 (C), 74.3 (CH), 34.1 (CH), 32.1 (CH_2), 29.6 (CH_3), 19.7 (CH_3), 19.4 (CH_3), 18.9 (CH), 15.8 (CH), 5.1 (CH_2), 1.6 (CH_2), 0.2 (CH_2), -0.4 (CH_2). **HRMS** (ESI-TOF) *m/z*: $[M+H]^+$ Calcd for $C_{23}H_{30}NO^+$ 336.2322; Found 336.2327.

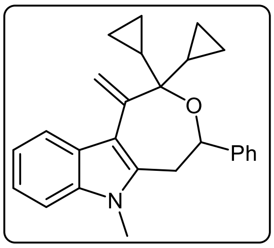

2,2-Dicyclopropyl-6-methyl-1-methylene-4-phenyl-1,4,5,6-tetrahydro-2*H*-oxepino[4,5-*b*]indole (4i): Following the general procedure with **3i** (185 mg, 0.5 mmol), the crude product was purified by column chromatography (hexane/EtOAc, 10/1) affording pure **4i** as a white solid (140 mg, 76%). R_f = 0.33 (hexane/EtOAc, 10/1). M.p. = 145–147 °C. **^{1}H NMR** (500 MHz, $CDCl_3$): δ (ppm) = 7.75 (d, J = 7.3 Hz, 1H), 7.51 (d, J = 7.0 Hz, 2H), 7.44–7.37 (m, 2H), 7.36–7.27 (m, 2H), 7.26–7.19 (m, 1H), 7.19–7.11 (m, 1H), 5.78 (d, J = 1.7 Hz, 1H), 5.57 (d, J = 1.7 Hz, 1H), 5.39 (dd, J = 10.7, 3.0 Hz, 1H), 3.62 (s, 3H), 3.21 (dd, J = 16.8, 3.0 Hz, 1H), 3.10 (dd, J = 16.8, 10.7 Hz, 1H), 1.44–1.27 (m, 1H), 0.95–0.74 (m, 2H), 0.70–0.55 (m, 1H), 0.55–0.43 (m, 1H), 0.41–0.24 (m, 3H), 0.20–0.04 (m, 2H). **^{13}C NMR** (125.7 MHz, $CDCl_3$): δ (ppm) = 146.4 (C), 143.8 (C), 137.1 (C), 134.8 (C), 128.3 (2 × CH), 127.19 (C), 127.17 (CH), 125.6 (2 × CH), 121.2 (CH), 119.5 (CH), 119.3 (CH), 115.3 (CH_2), 113.2 (C), 108.7 (CH), 85.9 (C), 72.3 (C), 39.1 (CH_2), 29.6 (CH_3), 18.4 (CH), 18.1 (CH), 3.6

(CH_2), 0.8 (2 × CH_2), 0.3 (CH_2). **HRMS** (ESI-TOF) *m/z*: $[M+H]^+$ Calcd for $C_{26}H_{28}NO^+$ 370.2165; Found 370.2169.

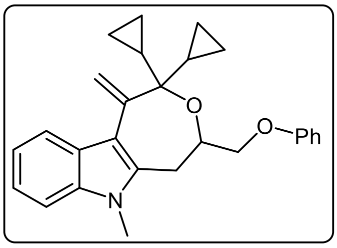

2,2-Dicyclopropyl-6-methyl-1-methylene-4-(phenoxymethyl)-1,4,5,6-tetrahydro-2*H*-oxepino[4,5-*b*]indole (4j): Following the general procedure with **3j** (200 mg, 0.5 mmol), the crude product was purified by column chromatography (hexane/EtOAc, 5/1) affording pure **4j** as a yellow solid (161 mg, 81%). R_f = 0.18 (hexane/EtOAc, 5/1). M.p. = 170–172 °C. **^{1}H NMR** (300 MHz, $CDCl_3$): δ (ppm) = 7.73 (d, *J* = 7.7 Hz, 1H), 7.44–7.30 (m, 3H), 7.29–7.14 (m, 2H), 7.09–6.96 (m, 3H), 5.79 (d, *J* = 1.4 Hz, 1H), 5.58 (d, *J* = 1.4 Hz, 1H), 4.84–4.67 (m, 1H), 4.30 (dd, *J* = 9.3, 5.1 Hz, 1H), 4.08 (dd, *J* = 9.3, 7.5 Hz, 1H), 3.69 (s, 3H), 3.28 (dd, *J* = 16.7, 2.4 Hz, 1H), 3.04 (dd, *J* = 16.7, 11.2 Hz, 1H), 1.57–1.42 (m, 1H), 1.04–0.90 (m, 1H), 0.8–0.65 (m, 3H), 0.65–0.50 (m, 1H), 0.49–0.30 (m, 1H), 0.30–0.17 (m, 2H), 0.16–0.05 (m, 1H). **^{13}C NMR** (75.4 MHz, $CDCl_3$): δ (ppm) = 158.8 (C), 145.5 (C), 137.2 (C), 134.5 (C), 129.6 (2 × CH), 127.1 (C), 121.1 (CH), 121.0 (CH), 119.5 (CH), 119.2 (CH), 116.3 (CH_2), 114.6 (2 × CH), 112.9 (C), 108.7 (CH), 86.5 (C), 71.0 (CH_2), 68.9 (CH), 33.3 (CH_2), 29.7 (CH_3), 18.8 (CH), 16.6 (CH), 4.4 (CH_2), 1.4 (CH_2), 0.8 (CH_2), 0.0 (CH_2). **HRMS** (ESI-TOF) *m/z*: $[M+H]^+$ Calcd for $C_{27}H_{30}NO_2^+$ 400.2271; Found 400.2267.

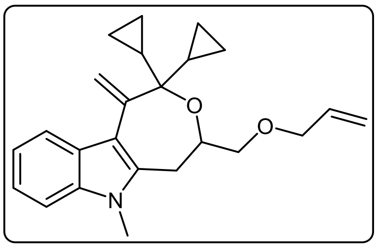

4-((Allyloxy)methyl)-2,2-dicyclopropyl-6-methyl-1-methylene-1,4,5,6-tetrahydro-2*H*-oxepino[4,5-*b*]indole (4k): Following the general procedure with **3k** (180 mg, 0.5 mmol), the crude product was purified by column chromatography (hexane/EtOAc, 5/1) affording pure **4k** as a yellow solid (156 mg, 86%). R_f = 0.21 (hexane/EtOAc, 5/1). M.p. = 108–110 °C. **^{1}H NMR** (300 MHz, $CDCl_3$): δ (ppm) = 7.73 (d, *J* = 7.5 Hz, 1H), 7.31 (d, *J* = 7.7 Hz, 1H), 7.29–7.21 (m, 1H), 7.21–7.12 (m, 1H), 6.14–5.90 (m, 1H), 5.77 (d, *J* = 1.6 Hz, 1H), 5.56 (d, *J* = 1.6 Hz, 1H), 5.39 (dd, *J* = 17.2, 1.5 Hz, 1H), 5.28 (dd, *J* = 10.4, 1.5 Hz, 1H), 4.63–4.44 (m, 1H), 4.15 (d, *J* = 5.5 Hz, 2H), 3.79 (dd, *J* = 9.7, 4.8 Hz, 1H), 3.68 (s, 3H), 3.60 (dd, *J* = 9.7, 7.3 Hz, 1H), 3.16 (dd, *J* = 16.8, 2.5 Hz, 1H), 2.97 (dd, *J* = 16.8, 11.1 Hz, 1H), 1.55–1.37 (m, 1H), 1.04–0.88 (m, 1H), 0.88–0.64 (m, 3H), 0.62–0.48 (m, 1H), 0.47–0.31 (m, 1H), 0.30–0.02 (m, 3H). **^{13}C NMR** (75.4 MHz, $CDCl_3$): δ (ppm) = 145.7 (C), 137.1 (C), 134.91 (C), 134.87 (CH), 127.1 (C), 121.0 (CH), 119.4 (CH), 119.1 (CH), 117.0 (CH_2), 115.8 (CH_2), 112.8 (C), 108.6 (CH), 86.0 (C), 73.6 (CH_2), 72.5 (CH_2), 69.4 (CH), 33.2 (CH_2), 29.6 (CH_3), 18.6 (CH), 16.9 (CH), 4.1 (CH_2), 1.3 (CH_2), 0.7 (CH_2), 0.0 (CH_2). **HRMS** (ESI-TOF) *m/z*: $[M+H]^+$ Calcd for $C_{24}H_{30}NO_2^+$ 364.2271; Found 364.2270.

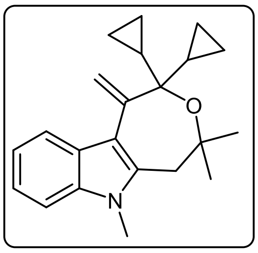

2,2-Dicyclopropyl-4,4,6-trimethyl-1-methylene-1,4,5,6-tetrahydro-2*H*-oxepino[4,5-*b*]indole (4l): Following the general procedure with **3l** (160 mg, 0.5 mmol), the crude product was purified by column chromatography (hexane/EtOAc, 10/1) affording pure **4l** as a white solid (125 mg, 78%). R_f = 0.34 (hexane/EtOAc, 10/1). M. p. = 110–112 °C. **^{1}H NMR** (500 MHz, $CDCl_3$): δ (ppm) = 7.88 (d, *J* = 7.9 Hz, 1H), 7.34 (d, *J* = 8.1 Hz, 1H), 7.29–7.24 (m, 1H), 7.24–7.16 (m, 1H), 5.85 (s, 1H), 5.59 (s, 1H), 3.69 (s, 3H), 3.07 (s, 2H), 1.53 (s, 3H), 1.52 (s, 3H), 1.10–0.99 (m, 2H), 0.73–0.49 (m, 4H), 0.39–0.17 (m, 4H). **^{13}C NMR** (125.7 MHz, $CDCl_3$): δ (ppm) = 148.1 (C), 137.3 (C), 134.6 (C), 127.1 (C), 121.1 (CH), 119.7 (CH), 119.5 (CH), 113.3 (C), 112.4 (CH_2), 108.7

(CH), 80.5 (C), 74.0 (C), 41.5 (CH_2), 30.5 (2 × CH_3), 29.6 (CH_3), 20.1 (2 × CH), 2.2 (2 × CH_2), 1.1 (2 × CH_2). **HRMS** (ESI-TOF) *m/z*: $[M+H]^+$ Calcd for $C_{22}H_{28}NO^+$ 322.2165; Found 322.2172.

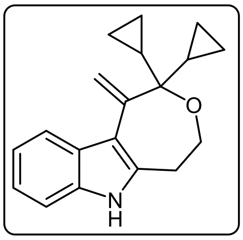

2,2-Dicyclopropyl-1-methylene-1,4,5,6-tetrahydro-2*H*-oxepino[4,5-*b*]indole (4m): Following the general procedure with **3m** (140 mg, 0.5 mmol), the crude product was purified by column chromatography (hexane/EtOAc, 5/1) affording pure **4m** as a white solid (111 mg, 80%). R_f = 0.25 (hexane/EtOAc, 5/1). M.p. = 180–182 °C. **^{1}H NMR** (500 MHz, $CDCl_3$): δ (ppm) = 7.92 (br s, 1H), 7.77–7.61 (m, 1H), 7.35–7.23 (m, 1H), 7.23–7.08 (m, 2H), 5.77 (d, *J* = 1.6 Hz, 1H), 5.54 (d, *J* = 1.6 Hz, 1H), 4.11 (t, *J* = 5.4 Hz, 2H), 3.04 (t, *J* = 5.4 Hz, 2H), 1.11–1.02 (m, 2H), 0.56–0.43 (m, 4H), 0.43–0.29 (m, 4H). **^{13}C NMR** (125.7 MHz, $CDCl_3$): δ (ppm) = 145.5 (C), 136.4 (C), 133.5 (C), 128.1 (C), 121.6 (CH), 119.8 (CH), 119.2 (CH), 116.0 (CH_2), 114.1 (C), 110.3 (CH), 85.9 (C), 61.7 (CH_2), 31.5 (CH_2), 18.1 (2 × CH), 1.8 (2 × CH_2), 0.8 (2 × CH_2). **HRMS** (ESI-TOF) *m/z*: $[M+H]^+$ Calcd for $C_{19}H_{22}NO^+$ 280.1696; Found 280.1703.

General procedure for the synthesis of tetrahydrooxepino[4,5-b]indoles 4n,o, indenylindoles 5 and 6, and 1,3-dien-2-yl-indol derivatives 8n,p

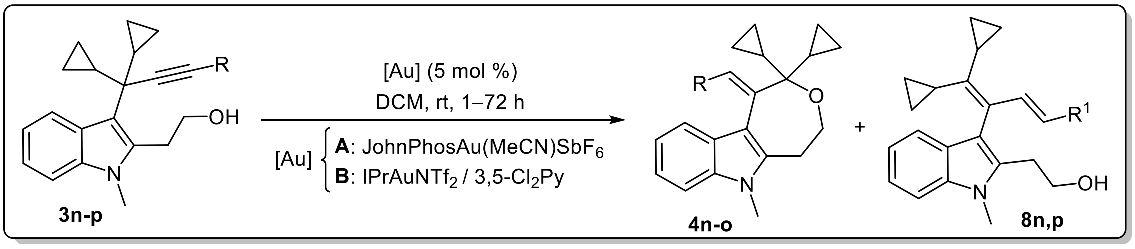

The corresponding catalytic system: **A** [JohnPhosAu(MeCN)SbF_6 (19 mg, 0.025 mmol, 0.05 equiv); **B** [IPrAuNTf_2 (21 mg, 0.025 mmol, 0.05 equiv) and 3,5-dichloropyridine (0.25 mmol, 36 mg, 0.5 equiv) was dissolved in DCM (1 mL), and the resulting solution was stirred at rt for 5 min. A solution of the corresponding 3-propargyl indole **3n-p** (0.5 mmol, 1 equiv) in DCM (1 mL) was then added. The reaction mixture was stirred at rt for 1–72 h (until complete consumption of the starting material, as determined by GC–MS or TLC). The mixture was filtered through a short pad of silica gel and celite using a 2/1 (hexane/EtOAc) mixture as eluent. The solvents were removed under reduced pressure, and the crude product was purified by flash column chromatography using mixtures of hexane/EtOAc as eluents, affording the corresponding tetrahydrooxepino[4,5-*b*]indoles **4n-o** and 1,3-dien-2-yl-indol derivatives **8n,p**.

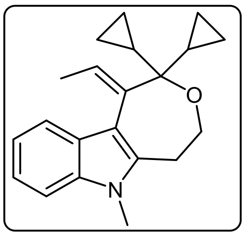

(*E*)-2,2-Dicyclopropyl-1-ethylidene-6-methyl-1,4,5,6-tetrahydro-2*H*-oxepino[4,5-*b*]indole (4n): Following the general procedure using catalytic system B with **3n** (154 mg, 0.5 mmol) for 1 h a ca. 9/1 mixture of **4n**/**8n** was obtained. The crude product was purified by column chromatography (hexane/EtOAc, 3/1) affording pure **4n** as a white solid (118 mg, 77%). R_f = 0.36 (hexane/EtOAc, 3/1). M.p. = 80–82 °C. Obtained and isolated as a >20/1 mixture of *E*/*Z* isomers. **^{1}H NMR** (500 MHz, $CDCl_3$): δ (ppm) = 7.34–7.30 (m, 2H), 7.24–7.18 (m, 1H), 7.18–7.11 (m, 1H), 6.27 (q, *J* = 6.9 Hz, 1H), 4.34 (td, *J* = 12.4, 2.5 Hz, 1H), 3.95 (dd, *J* = 12.4, 5.5 Hz, 1H), 3.69 (s, 3H), 3.20–3.06 (m, 1H), 2.93–2.80 (m, 1H), 1.71 (d, *J* = 6.9 Hz, 3H), 1.20–1.06 (m, 1H), 0.82–0.70 (m, 2H),

0.65–0.47 (m, 3H), 0.36–0.28 (m, 1H), 0.24–0.14 (m, 1H), 0.13– -0.01 (m, 2H). **^{13}C NMR** (125.7 MHz, $CDCl_3$): *δ* (ppm) = 137.1 (C), 136.9 (C), 135.3 (C), 127.6 (C), 125.1 (CH), 120.5 (CH), 120.1 (CH), 118.9 (CH), 110.8 (C), 108.6 (CH), 85.5 (C), 61.1 (CH_2), 30.9 (CH_2), 29.6 (CH_3), 19.5 (CH_3), 16.3 (CH), 16.1 (CH), 2.3 (CH_2), 1.2 (CH_2), 0.6 (CH_2), 0.3 (CH_2). **HRMS** (ESI-TOF) *m/z*: $[M+H]^+$ Calcd for $C_{21}H_{26}NO^+$ 308.2009; Found 308.2016.

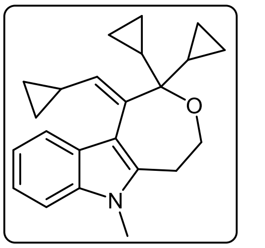

(*E*)-2,2-Dicyclopropyl-1-(cyclopropylmethylene)-6-methyl-1,4,5,6-tetrahydro-2*H*-oxepino[4,5-*b*]indole (4o): Following the general procedure using catalytic system B with **3o** (166 mg, 0.5 mmol) for 72 h, the crude product was purified by column chromatography (hexane/EtOAc, 5/1) affording pure **4o** as a yellow oil (109 mg, 66%). R_f = 0.33 (hexane/EtOAc, 5/1). Obtained and isolated as a >20/1 mixture of *E*/*Z* isomers. **^{1}H NMR** (300 MHz, $CDCl_3$): *δ* (ppm) = 7.66 (d, *J* = 7.4 Hz, 1H), 7.33 (d, *J* = 7.7 Hz, 1H), 7.23 (at, *J* = 7.3 Hz, 1H), 7.17 (at, *J* = 7.4 Hz, 1H), 5.52 (d, *J* = 10.1 Hz, 1H), 4.43 (td, *J* = 12.3, 2.5 Hz, 1H), 4.05–3.93 (m, 1H), 3.70 (s, 3H), 3.16 (dt, *J* = 16.7, 12.3, 1H), 2.92 (d, *J* = 16.7 Hz, 1H), 1.59–1.40 (m, 1H), 1.19–1.07 (m, 1H), 1.00–0.84 (m, 1H), 0.84–0.73 (m, 2H), 0.72–0.43 (m, 6H), 0.43–0.30 (m, 1H), 0.29–0.15 (m, 1H), 0.15–0.02 (m, 2H). **^{13}C NMR** (75.4 MHz, $CDCl_3$): *δ* (ppm) = 136.8 (C), 135.4 (C), 135.2 (CH), 134.1 (C), 127.8 (C), 120.44 (CH), 120.39 (CH), 118.8 (CH), 111.2 (C), 108.4 (CH), 85.4 (C), 61.0 (CH_2), 30.9 (CH_2), 29.6 (CH_3), 19.4 (CH), 16.4 (CH), 13.0 (CH), 8.4 (CH_2), 7.0 (CH_2), 2.4 (CH_2), 1.2 (CH_2), 0.6 (CH_2), 0.3 (CH_2). **HRMS** (ESI-TOF) *m/z*: $[M+H]^+$ Calcd for $C_{23}H_{28}NO^+$ 334.2165; Found 334.2166.

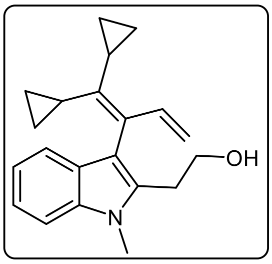

2-(3-(1,1-Dicyclopropylbuta-1,3-dien-2-yl)-1-methyl-1*H*-indol-2-yl)ethan-1-ol (8n): Following the general procedure using catalytic system A with **3n** (154 mg, 0.5 mmol) for 24 h, a ca. 1.3/1 mixture of **8n**/**4n** was obtained. The crude product was purified by column chromatography (hexane/EtOAc, 3/1) affording pure **8n** as a white oil (69 mg, 45%). R_f = 0.25 (hexane/EtOAc, 3/1). **^{1}H NMR** (300 MHz, $CDCl_3$): *δ* (ppm) = 7.52–7.40 (m, 1H), 7.36–7.29 (m, 2H), 7.24–7.16 (m, 1H), 7.11–7.03 (m, 1H), 4.96 (dd, *J* = 10.7, 2.2 Hz, 1H), 4.61 (dd, *J* = 17.2, 2.2 Hz, 1H), 3.84–3.73 (m, 2H), 3.76 (s, 3H), 3.01–2.90 (m, 2H), 1.71 (br s, 1H), 1.43–1.28 (m, 2H), 0.93–0.81 (m, 2H), 0.81–0.72 (m, 1H), 0.72–0.57 (m, 2H), 0.48–0.31 (m, 1H). **^{13}C NMR** (75.4 MHz, $CDCl_3$): *δ* (ppm) = 143.1 (C), 137.1 (C), 136.8 (CH), 134.3 (C), 130.8 (C), 128.1 (C), 121.0 (CH), 119.7 (CH), 119.1 (CH), 113.8 (CH_2), 112.6 (C), 108.8 (CH), 61.8 (CH_2), 30.0 (CH_3), 28.8 (CH_2), 16.8 (CH), 10.7 (CH), 6.5 (CH_2), 6.3 (CH_2), 6.2 (CH_2), 5.8 (CH_2). **HRMS** (ESI-TOF) *m/z*: $[M+H]^+$ Calcd for $C_{21}H_{26}NO^+$ 308.2009; Found 308.2014.

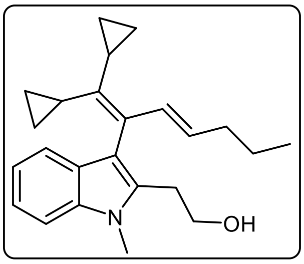

(*E*)-2-(3-(1,1-Dicyclopropylhepta-1,3-dien-2-yl)-1-methyl-1*H*-indol-2-yl)ethan-1-ol (8p): Following the general procedure using catalytic system B with **4p** (175 mg, 0.5 mmol) for 1 h, the crude product was purified by column chromatography (hexane/EtOAc, 5/1) affording pure **8p** as a yellow oil (136 mg, 78%). R_f = 0.30 (hexane/EtOAc, 5/1). Obtained and isolated as a >20/1 mixture of *E*/Z isomers. **^{1}H NMR** (300 MHz, $CDCl_3$): *δ* (ppm) = 7.40–7.33 (m, 2H), 7.28–7.20 (m, 1H), 7.15 (d, *J* = 13.9 Hz, 1H), 7.12–7.07 (m, 1H), 5.16 (dt, *J* = 13.9, 7.1 Hz, 1H), 3.86–3.77 (m, 2H), 3.80 (s, 3H), 3.08–2.92 (m, 2H), 2.16–2.03 (m, 2H), 1.87 (br s, 1H), 1.47–1.25 (m, 5H), 0.95–0.83 (m, 1H), 0.90 (t, *J* = 7.3 Hz, 3H), 0.79–0.70 (m, 1H), 0.70–0.58 (m, 3H), 0.47–0.26 (m, 2H). **^{13}C NMR** (75.4 MHz, $CDCl_3$): *δ* (ppm) = 139.8 (C), 137.0 (C), 134.1 (C), 131.2

(CH), 130.7 (CH), 130.4 (C), 128.1 (C), 120.9 (CH), 119.9 (CH), 119.0 (CH), 113.7 (C), 108.7 (CH), 61.8 (CH_2), 35.2 (CH_2), 29.9 (CH_3), 28.8 (CH_2), 22.8 (CH_2), 16.5 (CH), 13.8 (CH), 10.7 (CH_3), 6.5 (CH_2), 6.3 (CH_2), 6.0 (CH_2), 5.5 (CH_2). **HRMS** (ESI-TOF) *m/z*: $[M+H]^+$ Calcd for $C_{24}H_{32}NO^+$ 350.2478; Found 350.2479.

Reactions of phenyl-substituted 2-hydroxymethyl-3-propargylindoles 1m,n. Synthesis of 5 and 6

Once the reactivity of various terminal 3-propargy-2-hydroxyfunctionalized indoles had been established, our next objective was to extend this methodology to substrates **1m,n**, which bear an aromatic substituent either at the propargylic position (**1m**, R^1 = Ph, R^2 = H) or on the alkyne (**1n**, R^1 = *c*-C_3H_5, R^2 = Ph). When these 3-propargylindoles **1m,n** were subjected to the previously optimized reaction conditions, the only products obtained were the corresponding 3-inden-2-yl indoles **5** and **6**, respectively, arising from a tandem 1,2-indole migration/(iso)aura-Nazarov cyclization. This reactivity is consistent with our previous report on analogous substrates lacking hydroxyl groups.[12] These results demonstrate that the tandem 1,2-indole migration/hydroxycyclization sequence requires the absence of Ar groups at both the propargylic position and the alkyne terminus in order to proceed efficiently.

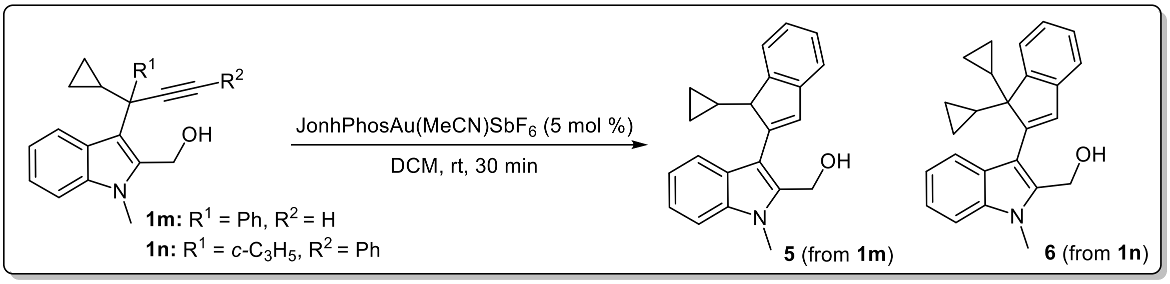

JonhPhosAu(MeCN)SbF_6 (19.5 mg, 0.025 mmol, 0.05 equiv) was dissolved in DCM (1 mL), and the resulting solution was stirred at rt for 5 min. A solution of the corresponding 3-propargyl indole **1m,n** (0.5 mmol, 1 equiv) in DCM (1 mL) was then added. The reaction mixture was stirred at rt for 30 min (until complete consumption of the starting material, as determined by GC–MS or TLC). The mixture was filtered through a short pad of silica gel and celite using a 2/1 (hexane/EtOAc) mixture as eluent. The solvents were removed under reduced pressure, and the crude product was purified by flash column chromatography using mixtures of hexane/EtOAc as eluents, affording the corresponding indenylindoles **5** and **6**.

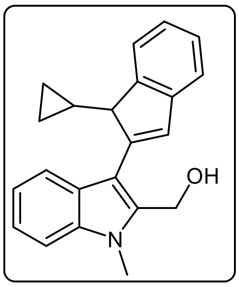

(3-(1-Cyclopropyl-1*H*-inden-2-yl)-1-methyl-1*H*-indol-2-yl)methanol (5): Following the procedure with **1m** (157 mg, 0.5 mmol), the crude product was purified by column chromatography (hexane/EtOAc, 3/1) affording pure **5** as a brown solid (100 mg, 63%). R_f = 0.18 (hexane/EtOAc, 3/1). M.p. = 125–127 °C. **^{1}H NMR** (300 MHz, $CDCl_3$): δ (ppm) = 7.74 (d, *J* = 7.9 Hz, 1H), 7.64 (d, *J* = 7.2 Hz, 1H), 7.49–7.33 (m, 4H), 7.33–7.26 (m, 1H), 7.25–7.16 (m, 1H), 6.83 (s, 1H), 5.02 (d, *J* = 12.8 Hz, 1H), 4.87 (d, *J* = 12.8 Hz, 1H), 3.91 (s, 3H), 3.79 (d, *J* = 8.3 Hz, 1H), 1.85 (br s, 1H), 0.84–0.65 (m, 1H), 0.45–0.15 (m, 4H). **^{13}C NMR** (75.4 MHz, $CDCl_3$): δ (ppm) = 146.9 (C), 146.1 (C), 144.6 (C), 137.4 (C), 135.7 (C), 128.9 (CH), 127.3 (C), 127.0 (CH), 124.3 (CH), 123.7 (CH), 122.7 (CH), 120.7

[12] Sanz, R.; Miguel, D.; Gohain, M.; García-García, P.; Fernández-Rodríguez, M. A.; González-Pérez, A.; Nieto-Faza, O.; De Lera, A. R. *Chem. Eur. J.* **2010**, *16*, 9818–9828.

(CH), 120.4 (CH), 120.0 (CH), 112.7 (C), 109.4 (CH), 55.3 (CH_2), 54.7 (CH), 30.1 (CH_3), 13.3 (CH), 3.0 (CH_2), 1.7 (CH_2). **HRMS** (ESI-TOF) *m/z*: $[M+H]^+$ Calcd for $C_{22}H_{22}NO^+$ 316.1696; Found 316.1702.

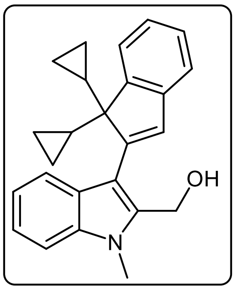

(3-(1,1-Dicyclopropyl-1*H*-inden-2-yl)-1-methyl-1*H*-indol-2-yl)methanol (6): Following the procedure with **1n** (177 mg, 0.5 mmol), the crude product was purified by column chromatography (hexane/EtOAc, 3/1) affording pure **6** as a white solid (114 mg, 64%). R_f = 0.27 (hexane/EtOAc, 3/1). M.p. = 131–133 °C. **^{1}H NMR** (300 MHz, $CDCl_3$): δ (ppm) = 7.73 (d, *J* = 8.0 Hz, 1H), 7.47–7.39 (m, 2H), 7.39–7.29 (m, 3H), 7.27–7.10 (m, 2H), 6.68 (s, 1H), 4.89 (s, 2H), 3.94 (s, 3H), 1.70 (s, 1H), 1.11–0.83 (m, 2H), 0.74–0.56 (m, 2H), 0.56–0.40 (m, 2H), 0.40–0.26 (m, 2H), 0.16–-0.05 (m, 2H). **^{13}C NMR** (75.4 MHz, $CDCl_3$): δ (ppm) = 152.1 (C), 147.2 (C), 144.3 (C), 137.4 (C), 136.3 (C), 130.8 (CH), 128.2 (C), 127.2 (CH), 124.0 (CH), 123.8 (CH), 122.2 (CH), 121.5 (CH), 120.8 (CH), 119.3 (CH), 111.5 (C), 109.0 (CH), 57.4 (C), 55.7 (CH_2), 30.2 (CH_3), 14.6 (2 × CH), 2.8 (2 × CH_2), –0.6 (2 × CH_2). **HRMS** (ESI-TOF) *m/z*: $[M+H]^+$ Calcd for $C_{25}H_{26}NO^+$ 356.2009; Found 356.2002.

Synthesis and characterization data of hexahydrooxocino[5,4-*b*]indole derivatives 10

General procedure for the synthesis of hexahydrooxocino[5,4-b]indole derivatives 10a-c

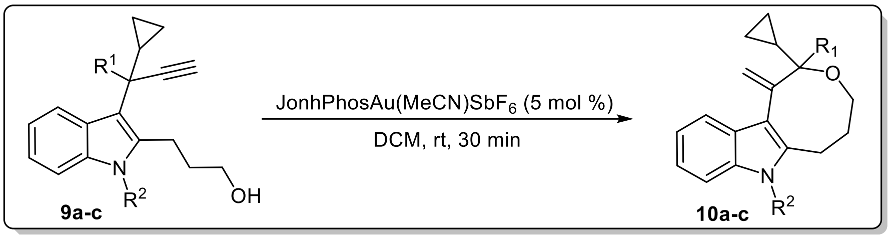

JonhPhosAu(MeCN)SbF_6 (19.5 mg, 0.025 mmol, 0.05 equiv) was dissolved in DCM (1 mL), and the resulting solution was stirred at rt for 5 min. A solution of the corresponding 3-propargylindole **9** (0.5 mmol, 1 equiv) in DCM (1 mL) was then added. The reaction mixture was stirred at rt for 30 min (until complete consumption of the starting material, as determined by GC–MS or TLC). The mixture was filtered through a short pad of silica gel and celite using a 2/1 (hexane/EtOAc) mixture as eluent. The solvents were removed under reduced pressure, and the crude product was purified by flash column chromatography using mixtures of hexane/EtOAc as eluents, affording the corresponding hexahydrooxocino[5,4-*b*]indoles **10a-c**.

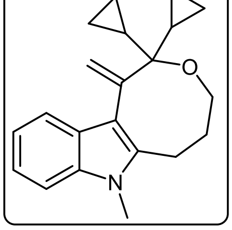

2,2-Dicyclopropyl-7-methyl-1-methylene-1,2,4,5,6,7-hexahydrooxocino[5,4-*b*]indole (10a): Following the general procedure with **9a** (154 mg, 0.5 mmol), the crude product was purified by column chromatography (hexane/EtOAc, 5/1) affording pure **10a** as a yellow solid (102 mg, 67%). R_f = 0.32 (hexane/EtOAc, 5/1). M. p. = 102–104 °C. **^{1}H NMR** (300 MHz, $CDCl_3$): δ (ppm) = 7.53 (d, *J* = 7.7 Hz, 1H), 7.27 (d, *J* = 9.1 Hz, 1H), 7.17 (at, *J* = 7.5 Hz, 1H), 7.09 (at, *J* = 7.5 Hz, 1H), 5.81 (d, *J* = 2.0 Hz, 1H), 5.26 (d, *J* = 2.0 Hz, 1H), 4.17–4.04 (m, 2H), 3.69 (s, 3H), 3.01 (dd, *J* = 6.3, 5.0 Hz, 2H), 2.30–2.15 (m, 2H), 1.12–0.89 (m, 2H), 0.78–0.57 (m, 2H), 0.54–0.36 (m, 2H), 0.36–0.25 (m, 2H), 0.25–0.10 (m, 2H). **^{13}C NMR** (75.4 MHz, $CDCl_3$): δ (ppm) = 151.7 (C), 136.9 (C), 136.5 (C), 128.9

(C), 120.5 (CH), 118.9 (CH), 118.9 (CH), 114.9 (CH_2), 112.0 (C), 108.1 (CH), 77.5 (C), 65.0 (CH_2), 29.4 (CH_3), 26.4 (CH_2), 25.1 (CH_2), 17.2 (2 × CH), 2.6 (2 × CH_2), 0.1 (2 × CH_2). **HRMS** (ESI-TOF) *m/z*: $[M+H]^+$ Calcd for $C_{21}H_{26}NO^+$ 308.2009; Found 308.2016.

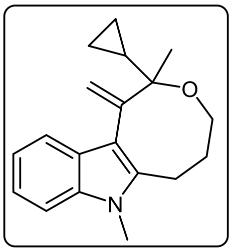

2-Cyclopropyl-2,7-dimethyl-1-methylene-1,2,4,5,6,7-hexahydrooxocino[5,4-*b*]indole (10b): Following the general procedure with **9b** (140 mg, 0.5 mmol), the crude product was purified by column chromatography (hexane/EtOAc, 10/1) affording pure **10b** as a yellow solid (64 mg, 46%). R_f = 0.18 (hexane/EtOAc, 10/1). M.p. = 114–116 °C. **^{1}H NMR** (500 MHz, DMSO-d_6, 100 °C): δ (ppm) = 7.36 (d, J = 7.9 Hz, 1H), 7.32 (d, J = 8.1 Hz, 1H), 7.05 (at, J = 7.5 Hz, 1H), 6.96 (at, J = 7.4 Hz, 1H), 5.54 (d, J = 0.8 Hz, 1H), 5.03 (d, J = 0.8 Hz, 1H), 3.92–3.84 (m, 1H), 3.84–3.78 (m, 1H), 3.67 (s, 3H), 2.97–2.87 (m, 2H), 2.22–2.07 (m, 1H), 2.07–1.93 (m, 1H), 1.10 (s, 3H), 1.09–1.01 (m, 1H), 0.45–0.28 (m, 2H), 0.28–0.12 (m, 2H). **^{13}C NMR** (125.7 MHz, DMSO-d_6, 100 °C): δ (ppm) = 151.2 (C), 136.8 (C), 135.7 (C), 128.3 (C), 119.5 (CH), 118.1 (CH), 117.2 (CH), 112.6 (CH_2), 110.3 (C), 108.0 (CH), 76.5 (C), 63.6 (CH_2), 28.5 (CH_3), 24.61 (CH_2), 24.58 (CH_2), 20.4 (CH_3), 19.2 (CH), 1.8 (CH_2), 0.3 (CH_2). **HRMS** (ESI-TOF) *m/z*: $[M+H]^+$ Calcd for $C_{19}H_{24}NO^+$ 282.1852; Found 282.1853.

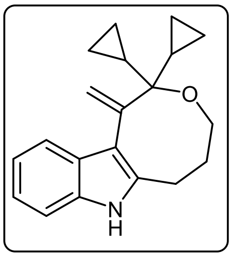

2,2-Dicyclopropyl-1-methylene-1,2,4,5,6,7-hexahydrooxocino[5,4-*b*]indole (10c): Following the general procedure with **9c** (147 mg, 0.5 mmol), the crude product was purified by column chromatography (hexane/EtOAc, 3/1) affording pure **10c** as a yellow oil (80 mg, 55%). R_f = 0.32 (hexane/EtOAc, 3/1). **^{1}H NMR** (300 MHz, $CDCl_3$): δ (ppm) = 7.79 (br s, 1H), 7.51 (d, J = 7.1 Hz, 1H), 7.26 (dd, J = 7.2, 6.1 Hz, 1H), 7.18–6.98 (m, 2H), 5.82 (d, J = 1.9 Hz, 1H), 5.28 (d, J = 1.9 Hz, 1H), 4.09–4.02 (m, 2H), 2.99 (dd, J = 6.8, 4.6 Hz, 2H), 2.15–2.05 (m, 2H), 1.13–0.91 (m, 2H), 0.75–0.52 (m, 2H), 0.52–0.37 (m, 2H), 0.37–0.24 (m, 2H), 0.26– -0.02 (m, 2H). **^{13}C NMR** (75.4 MHz, $CDCl_3$): δ (ppm) = 151.0 (C), 135.6 (C), 135.1 (C), 130.1 (C), 120.9 (CH), 119.3 (CH), 118.9 (CH), 115.4 (CH_2), 112.8 (C), 109.8 (CH), 77.8 (C), 64.7 (CH_2), 27.2 (CH_2), 25.8 (CH_2), 17.1 (2 × CH), 2.5 (2 × CH_2), 0.2 (2 × CH_2). **HRMS** (ESI-TOF) *m/z*: $[M+H]^+$ Calcd for $C_{20}H_{24}NO^+$ 294.1852; Found 294.1859.

Synthesis and characterization data of 4-methyltetrahydropyrano[3,4-*b*]indole derivatives 11

General procedure for the synthesis of 4-methyltetrahydropyrano[3,4-b]indole derivatives 11a-f

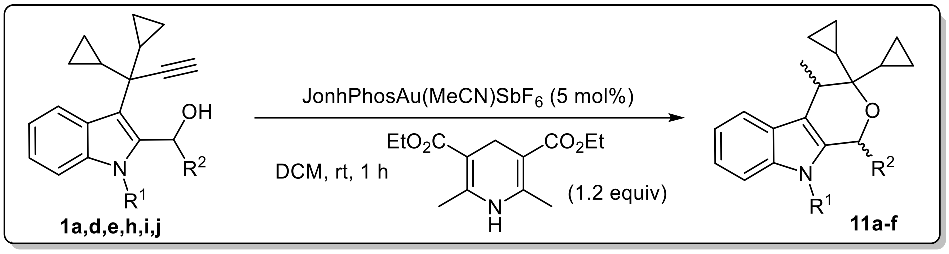

JonhPhosAu(MeCN)SbF_6 (19.5 mg, 0.025 mmol, 0.05 equiv) was dissolved in DCM (1 mL), and the resulting solution was stirred at rt for 5 min. A solution of the corresponding 3-propargyl indole **1** (0.5 mmol, 1 equiv) and Hantzsch ester (0.6 mmol, 152 mg, 1.2 equiv) in DCM (1 mL) was then added. The reaction mixture was stirred at rt for 1 h (until complete consumption of the starting material, as

determined by GC–MS or TLC). The mixture was filtered through a short pad of silica gel and celite using a 2/1 (hexane/EtOAc) mixture as eluent. The solvents were removed under reduced pressure, and the crude product was purified by flash column chromatography using mixtures of hexane/EtOAc as eluents, affording the corresponding 4-methyltetrahydropyrano[3,4-*b*]indole derivatives **11a-f**.

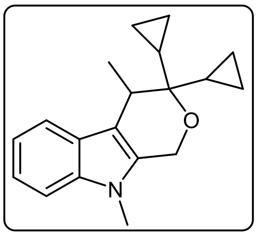

3,3-Dicyclopropyl-4,9-dimethyl-1,3,4,9-tetrahydropyrano[3,4-*b*]indole (11a): Following the general procedure with **1a** (140 mg, 0.5 mmol), the crude product was purified by column chromatography (hexane/EtOAc, 5/1) affording pure **11a** as a yellow solid (108 mg, 77%). R_f = 0.28 (hexane/EtOAc, 5/1). M. p. = 102–104 °C **^{1}H NMR** (300 MHz, $CDCl_3$): δ (ppm) = 7.74 (d, *J* = 7.8 Hz, 1H), 7.39 (d, *J* = 7.9 Hz, 1H), 7.36–7.27 (m, 1H), 7.27–7.16 (m, 1H), 4.97 (s, 2H), 3.65 (s, 3H), 3.19 (q, *J* = 7.0 Hz, 1H), 1.60 (d, *J* = 7.0 Hz, 3H), 1.23–1.10 (m, 1H), 1.05–0.93 (m, 1H), 0.8–0.72 (m, 2H), 0.72–0.58 (m, 2H), 0.58–0.47 (m, 2H), 0.47–0.38 (m, 2H). **^{13}C NMR** (75.4 MHz, $CDCl_3$): δ (ppm) = 137.4 (C), 132.6 (C), 126.9 (C), 120.7 (CH), 118.8 (CH), 118.7 (CH), 112.0 (C), 108.8 (CH), 74.9 (C), 57.8 (CH_2), 36.7 (CH), 29.4 (CH_3), 16.9 (CH_3), 15.2 (CH), 14.5 (CH), 2.0 (CH_2), 1.2 (CH_2), 0.3 (CH_2), 0.0 (CH_2). **HRMS** (ESI-TOF) *m/z*: $[M+H]^+$ calcd for $C_{19}H_{24}NO^+$ 282.1852; Found 282.1852.

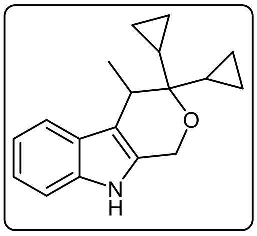

3,3-Dicyclopropyl-4-methyl-1,3,4,9-tetrahydropyrano[3,4-*b*]indole (11b): Following the general procedure with **1j** (130 mg, 0.5 mmol), the crude product was purified by column chromatography (hexane/EtOAc, 5/1) affording pure **11b** as a yellow solid (108 mg, 81%). R_f = 0.23 (hexane/EtOAc, 5/1). M. p. = 96–98 °C. **^{1}H NMR** (300 MHz, $CDCl_3$): δ (ppm) = 7.71–7.63 (m, 1H), 7.58 (br s, 1H), 7.32–7.26 (m, 1H), 7.25–7.14 (m, 2H), 4.90–4.68 (m, 2H), 3.11 (q, *J* = 7.0 Hz, 1H), 1.54 (d, *J* = 7.0 Hz, 3H), 1.17–1.01 (m, 1H), 1.01–0.82 (m, 1H), 0.81–0.65 (m, 2H), 0.64–0.51 (m, 2H), 0.51–0.42 (m, 2H), 0.42–0.28 (m, 2H). **^{13}C NMR** (75.4 MHz, $CDCl_3$): δ (ppm) = 136.5 (C), 131.3 (C), 127.2 (C), 121.3 (CH), 119.4 (CH), 118.7 (CH), 113.2 (C), 111.1 (CH), 75.2 (C), 58.3 (CH_2), 36.5 (CH), 16.7 (CH), 15.2 (CH), 14.7 (CH_3), 2.0 (CH_2), 1.3 (CH_2), 0.3 (CH_2), 0.0 (CH_2). **HRMS** (ESI-TOF) *m/z*: $[M+H]^+$ Calcd for $C_{18}H_{22}NO^+$ 268.1696; Found 268.1695.

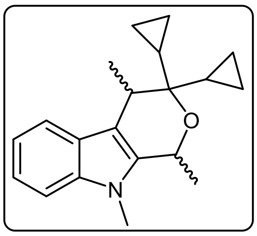

3,3-Dicyclopropyl-1,4,9-trimethyl-1,3,4,9-tetrahydropyrano[3,4-*b*]indole (11c): Following the general procedure with **1d** (147 mg, 0.5 mmol), the crude product was purified by column chromatography (hexane/EtOAc, 15/1) affording pure **11c** as a white solid (95 mg, 65%). R_f = 0.31 (hexane/EtOAc, 15/1). M.p. = 76–78 °C. Obtained and isolated as a ca. 1/1 mixture of diasteroisomers. Data for both diasteroisomers: **^{1}H NMR** (500 MHz, $CDCl_3$): δ (ppm) = 7.85 (d, *J* = 8.0 Hz, 1H), 7.66 (d, *J* = 7.8 Hz, 1H), 7.41–7.35 (m, 2H), 7.34–7.26 (m, 2H), 7.26–7.15 (m, 2H), 5.47–4.91 (m, 2H), 3.76 (s, 3H), 3.75 (s, 3H), 3.43 (qd, *J* = 7.1, 1.8 Hz, 1H), 3.04 (qd, *J* = 6.9, 1.1 Hz, 1H), 1.69 (d, *J* = 7.2 Hz, 3H), 1.65 (d, *J* = 6.3 Hz, 3H), 1.61 (d, *J* = 6.4 Hz, 3H), 1.51 (d, *J* = 7.0 Hz, 3H), 1.23–1.10 (m, 2H), 1.08–0.99 (m, 1H), 0.87–0.73 (m, 4H), 0.73–0.55 (m, 4H), 0.55–0.45 (m, 2H), 0.47–0.36 (m, 5H), 0.35–0.24 (m, 2H). **^{13}C NMR** (125.7 MHz, $CDCl_3$): δ (ppm) = 137.9 (C), 137.7 (C), 137.2 (C), 136.7 (C), 126.8 (C), 126.6 (C), 121.1 (CH), 120.7 (CH), 120.2 (CH), 118.9 (CH), 118.9 (CH), 118.1 (CH), 113.6 (C), 111.1 (C), 108.93 (CH), 108.87 (CH), 75.0 (C), 74.2 (C), 63.8 (CH), 63.3 (CH), 39.5 (CH), 36.0 (CH), 31.1 (CH_3), 31.0 (CH_3), 21.6 (CH_3), 21.5 (CH_3), 18.4 (CH), 16.9 (CH), 15.7 (CH), 15.4 (CH), 13.8 (CH_3), 12.6 (CH_3), 3.5 (CH_2),

2.6 (CH_2), 1.7 (CH_2), 1.4 (CH_2), 0.3 (CH_2), 0.1 (CH_2), -0.6 (CH_2), -1.9 (CH_2). **HRMS** (ESI-TOF) *m/z*: $[M+H]^+$ Calcd for $C_{20}H_{26}NO^+$ 296.2009; Found 296.2009.

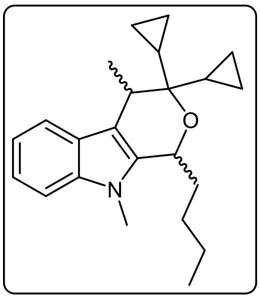

1-Butyl-3,3-dicyclopropyl-4,9-dimethyl-1,3,4,9-tetrahydropyrano[3,4-*b*]indole (11d): Following the general procedure with **1e** (165 mg, 0.5 mmol), the crude product was purified by column chromatography (hexane/EtOAc, 10/1) affording pure **11d** as an orange oil (135 mg, 80%). R_f = 0.35 (hexane/EtOAc, 10/1). Obtained and isolated as a ca. 1.5/1 mixture of diasteroisomers. Data for both diasteroisomers: **^{1}H NMR** (500 MHz, $CDCl_3$): δ (ppm) = 7.83 (d, *J* = 7.9 Hz, 1H), 7.63 (d, *J* = 7.7 Hz, 1H), 7.36 (d, *J* = 8.1 Hz, 2H), 7.32–7.24 (m, 2H), 7.23–7.12 (m, 2H), 5.13–5.05 (m, 2H), 3.74 (s, 3H), 3.73 (s, 3H), 3.41 (q, *J* = 7.1 Hz, 1H), 3.02 (q, *J* = 6.0 Hz, 1H), 2.14–1.81 (m, 4H), 1.66 (d, *J* = 7.1 Hz, 3H), 1.47 (d, *J* = 6.0 Hz, 3H), 1.60–1.27 (m, 9H), 1.19–1.04 (m, 2H), 1.04–0.91 (m, 7H), 0.86–0.70 (m, 3H), 0.68–0.45 (m, 6H), 0.45–0.31 (m, 5H), 0.31–0.22 (m, 2H). **^{13}C NMR** (125.7 MHz, $CDCl_3$): δ (ppm) = 138.0 (C), 137.8 (C), 136.1 (C), 135.4 (C), 126.9 (C), 126.7 (C), 121.0 (CH), 120.5 (CH), 120.2 (CH), 118.87 (CH), 118.85 (CH), 118.0 (CH), 114.5 (C), 111.8 (C), 108.92 (CH), 108.86 (CH), 74.7 (C), 73.8 (C), 67.3 (CH), 66.7 (CH), 39.9 (CH), 36.2 (CH), 34.7 (CH_2), 34.6 (CH_2), 31.1 (2 × CH_3), 27.0 (CH_2), 26.9 (CH_2), 22.83 (CH_2), 22.76 (CH_2), 18.4 (CH), 17.0 (CH), 15.8 (CH), 15.2 (CH), 14.3 (2 × CH_3), 13.8 (CH_3), 12.4 (CH_3), 3.6 (CH_2), 2.6 (CH_2), 1.7 (CH_2), 1.3 (CH_2), 0.3 (CH_2), 0.1 (CH_2), -0.7 (CH_2), -2.1 (CH_2). **HRMS** (ESI-TOF) *m/z*: $[M+H]^+$ Calcd for $C_{23}H_{32}NO^+$ 338.2478; Found 338.2476.

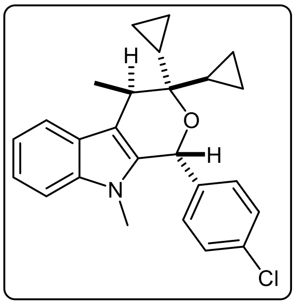

(1*R,4*R**)-1-(4-chlorophenyl)-3,3-dicyclopropyl-4,9-dimethyl-1,3,4,9-tetrahydropyrano[3,4-*b*]indole (11e):** Following the general procedure with **1h** (195 mg, 0.5 mmol), the crude product was purified by column chromatography (hexane/EtOAc, 40/1) affording pure **11e** as a yellow oil (145 mg, 74%). R_f = 0.18 (hexane/EtOAc, 40/1). Obtained and isolated as a ca. 8/1 mixture of diasteroisomers. Data for the major diasteroisomer: **^{1}H NMR** (500 MHz, $CDCl_3$): δ (ppm) = 7.77 (d, *J* = 7.8 Hz, 1H), 7.49–7.40 (m, 4H), 7.39–7.31 (m, 2H), 7.31–7.22 (m, 1H), 6.01 (s, 1H), 3.28 (s, 3H), 3.18 (q, *J* = 6.9 Hz, 1H), 1.67 (d, *J* = 7.0 Hz, 3H), 1.40–1.32 (m, 1H), 1.09–0.99 (m, 1H), 0.87–0.79 (m, 1H), 0.79–0.72 (m, 1H), 0.70–0.60 (m, 2H), 0.61–0.50 (m, 2H), 0.49–0.35 (m, 2H). **^{13}C NMR** (125.7 MHz, $CDCl_3$): δ (ppm) = 139.5 (C), 137.7 (C), 134.2 (C), 133.6 (C), 130.6 (2 × CH), 128.9 (2 × CH), 126.3 (C), 121.5 (CH), 119.0 (CH), 118.3 (CH), 115.1 (C), 109.0 (CH), 75.6 (C), 71.0 (CH), 35.8 (CH), 30.7 (CH_3), 18.8 (CH_3), 16.9 (CH), 16.0 (CH), 2.8 (CH_2), 1.7 (CH_2), 0.4 (CH_2), -0.6 (CH_2). **HRMS** (ESI-TOF) *m/z*: $[M–H]^+$ Calcd for $C_{25}H_{25}NClO^+$ 390.1619; Found 390.1620.

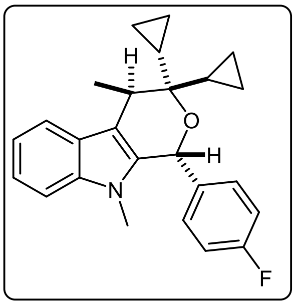

(1*R,4*R**)-1-(4-Fluorophenyl)-3,3-dicyclopropyl-4,9-dimethyl-1,3,4,9-tetrahydropyrano[3,4-*b*]indole (11f):** Following the general procedure with **1i** (136 mg, 0.5 mmol), the crude product was purified by column chromatography (hexane/EtOAc, 10/1) affording pure **11f** as a yellow oil (153 mg, 82%). R_f = 0.29 (hexane/EtOAc, 10/1). Obtained and isolated as a ca. 7/1 mixture of diasteroisomers. Data for the major diasteroisomer: **^{1}H NMR** (500 MHz, $CDCl_3$): δ (ppm) = 7.66 (d, *J* = 7.6 Hz, 1H), 7.40–7.31 (m, 2H), 7.30–7.22 (m, 2H), 7.22–7.14 (m, 1H), 7.07 (t, *J* = 8.7 Hz, 2H), 5.91 (s, 1H), 3.19 (s, 3H), 3.11–3.01 (m, 1H), 1.56 (d, *J* = 6.9 Hz, 3H), 1.29–1.18 (m, 1H), 0.78–0.68 (m, 1H), 0.68–0.58

(m, 1H), 0.58–0.42 (m, 4H), 0.40–0.24 (m, 3H). **^{13}C NMR** (125.7 MHz, $CDCl_3$): *δ* (ppm) = 162.8 (C, d, *J* = 246.7 Hz), 137.7 (C), 136.8 (C, d, *J* = 3.2 Hz), 134.0 (C), 130.9 (CH, d, *J* = 8.2 Hz), 126.3 (C), 121.4 (CH), 119.0 (CH), 118.3 (CH), 115.6 (CH, d, *J* = 21.5 Hz), 115.1 (C), 109.0 (CH), 75.6 (C), 71.0 (CH), 35.8 (CH), 30.7 (CH_3), 18.8 (CH_3), 16.9 (CH), 16.1 (CH), 2.7 (CH_2), 1.7 (CH_2), 0.4 (CH_2), -0.6 (CH_2). **HRMS** (ESI-TOF) *m/z*: $[M+H]^+$ Calcd for $C_{25}H_{27}NFO^+$ 376.2071; Found 376.2061.

Further limitations about substrate scope

General procedure for the synthesis of 3-propargylindole derivatives S23,24

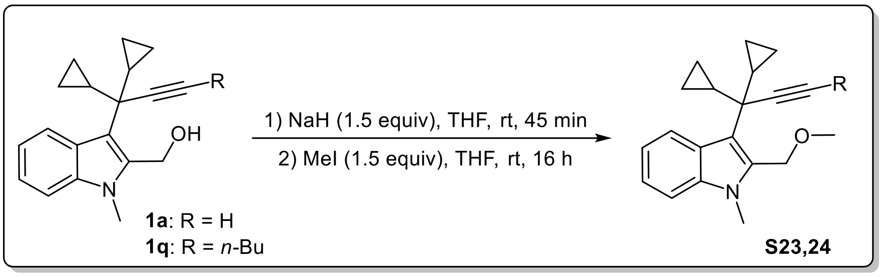

NaH (54 mg, 2.25 mmol, 1.5 equiv) was added to the corresponding 3-propargilindol (1.5 mmol, 1 equiv) dissolved in THF (5 mL). The reaction mixture was stirred at rt for 45 min. Then, MeI (320 mg, 2.25 mmol, 1.5 equiv) was added to the mixture and it was stirred at rt for 16 h. The reaction was quenched by the addition of water, and the mixture was extracted with Et_2O (3 × 15 mL). The combined organic layers were dried over anhydrous Na_2SO_4, and the solvent was removed under reduced pressure. The residue was purified by silica gel column chromatography using mixtures of hexane/EtOAc as eluents to afford the corresponding 3-propargylindole derivatives **S23,24**.

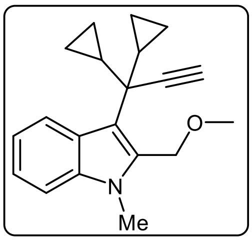

3-(1,1-Dicyclopropylprop-2-yn-1-yl)-2-(methoxymethyl)-1-methyl-1*H*-indole (S23): Following the general procedure with **1a** (418 mg, 1.5 mmol) the crude product was purified by column chromatography (hexane/EtOAc, 5/1) affording pure **S23** as a yellow oil (263 mg, 60%). R_f = 0.32 (hexane/EtOAc, 5/1). **^{1}H NMR** (500 MHz, $CDCl_3$): *δ* (ppm) = 8.29 (d, *J* = 8.3 Hz, 1H), 7.40 (d, *J* = 8.3 Hz, 1H), 7.33 (at, *J* = 7.6 Hz, 1H), 7.19 (at, *J* = 8.1 Hz, 1H), 5.13 (s, 2H), 3.84 (s, 3H), 3.55 (s, 3H), 2.41 (s, 1H), 1.84–1.73 (m, 2H), 1.02–0.85 (m, 2H), 0.75–0.71 (m, 2H), 0.71–0.59 (m, 2H), 0.63–0.51 (m, 2H). **^{13}C NMR** (125.7 MHz, $CDCl_3$): *δ* (ppm) = 137.3 (C),132.8 (C), 126.3 (C), 122.6 (CH), 121.8 (CH), 119.0 (CH), 116.7 (C), 109.4 (CH), 84.8 (C), 72.6 (C), 64.3 (CH_2), 57.9 (CH_3), 43.1 (C), 29.9 (CH_3), 21.3 (2 x CH), 3.8 (2 × CH_2), 2.8 (2 × CH_2). **HRMS** (ESI-TOF) *m/z*: $[M+H]^+$ Calcd for $C_{20}H_{24}NO^+$ 294.1852; Found 294.1857.

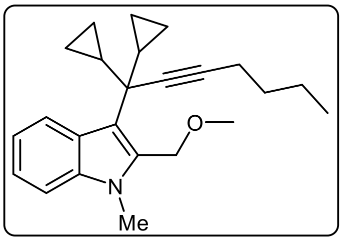

3-(1,1-Dicyclopropylhept-2-yn-1-yl)-2-(methoxymethyl)-1-methyl-1*H*-indole (S24): Following the general procedure with **1q** (503 mg, 1.5 mmol) the crude product was purified by column chromatography (hexane/EtOAc, 5/1) affording pure **S24** as a yellow oil (371 mg, 71%). R_f = 0.36 (hexane/EtOAc, 5/1). **^{1}H NMR** (300 MHz, $CDCl_3$): *δ* (ppm) = 8.19 (d, *J* = 8.2 Hz, 1H), 7.33 (d, *J* = 8.2 Hz, 1H), 7.24 (at, *J* = 7.5 Hz, 1H), 7.09 (at, *J* = 7.5 Hz, 1H), 5.09 (s, 2H), 3.79 (s, 3H), 3.46 (s, 3H), 2.28 (t, *J* = 6.9 Hz, 2H), 1.81-1.64 (m, 2H), 1.64–1.40 (m, 4H), 0.98 (t, *J* = 7.1 Hz, 3H), 0.88–0.71 (m, 2H), 0.67–0.47 (m, 4H), 0.47–0.35 (m, 2H). **^{13}C NMR**

(75.4 MHz, $CDCl_3$): δ (ppm) = 137.3 (C), 132.4 (C), 126.3 (C), 122.7 (CH), 121.6 (CH), 118.6 (CH), 118.3 (C), 109.2 (CH), 84.6 (C), 79.9 (C), 64.2 (CH_2), 57.6 (CH_3), 43.3 (C), 31.2 (CH_2), 29.9 (CH_3), 22.2 (CH_2), 21.6 (2 x CH), 18.6 (CH_2), 13.8 (CH_3), 3.5 (2 × CH_2), 2.5 (2 × CH_2). **HRMS** (ESI-TOF) *m/z*: $[M+H]^+$ Calcd for $C_{24}H_{32}NO^+$ 350.2478; Found 350.2469.

Reactivity of 3-propargylindole S23 under gold(I)-catalysis

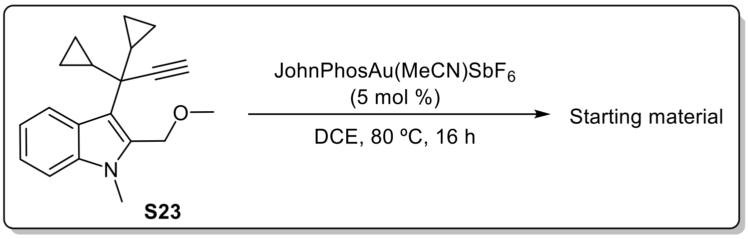

JohnPhosAu(MeCN)SbF_6 (19.5 mg, 0.025 mmol) was dissolved in DCM (1 mL), and the resulting solution was stirred at rt for 5 min. A solution of 3-propargylindole **S23** (146 mg, 0.5 mmol) in DCM (1 mL) was then added. The reaction was stirred at rt for 24 h, but no progress was observed. The reaction conditions were forced by heating at 80 °C, but after 16 h at this temperature only starting material **S23** was recovered.

Reactivity of 3-propargylindole S24 under gold(I)-catalysis: Synthesis of 1,3-dien-2-yl-indol 8q

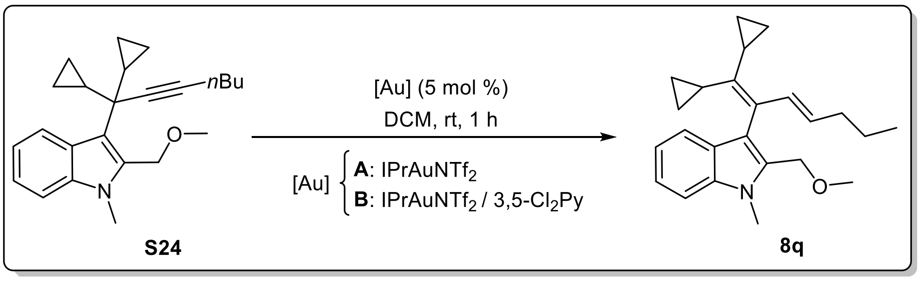

The corresponding catalytic system: **A** IPrAuNTf_2 (21 mg, 0.025 mmol); **B** [IPrAuNTf_2 (21 mg, 0.025 mmol) and 3,5-dichloropyridine (0.25 mmol, 36 mg)] was dissolved in DCM (1 mL), and the resulting solution was stirred at rt for 5 min. A solution of 3-propargyl indole **S24** (175 mg, 0.5 mmol) in DCM (1 mL) was then added. The reaction mixture was stirred at rt for 1 h (until complete consumption of the starting material, as determined by GC–MS or TLC). The mixture was filtered through a short pad of silica gel and celite using a 2/1 (hexane/EtOAc) mixture as eluent. The solvents were removed under reduced pressure, and the crude product was purified by flash column chromatography using a 10/1 mixture of hexane and EtOAc as eluent to afford pure 1,3-dien-2-yl-indol derivative **8q** as a yellow oil (158 mg, 91%, using IPrAuNTf_2 as catalyst). With both catalytic systems the diene **8q** was exclusively obtained.

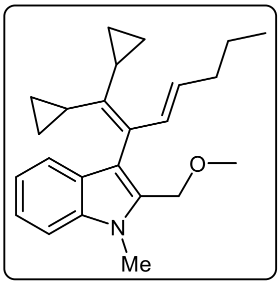

(*E*)-3-(1,1-Dicyclopropylhepta-1,3-dien-2-yl)-2-(methoxymethyl)-1-methyl-1*H*-indole (8q): R_f = 0.37 (hexane/EtOAc, 5/1). Obtained and isolated as a 12/1 mixture of *E*/*Z* isomers. **^{1}H NMR** (300 MHz, $CDCl_3$): δ (ppm) = 7.41 (d, *J* = 7.8 Hz, 1H), 7.36 (d, *J* = 8.1 Hz, 1H), 7.25 (at, *J* = 7.6 Hz, 1H), 7.13–7.02 (m, 2H), 5.22–5.08 (m, 1H), 4.50 (q, *J* = 12.6 Hz, 2H), 3.86 (s, 3H), 3.27 (s, 3H), 2.06 (q, *J* = 7.2 Hz, 2H), 1.50–1.38 (m, 1H), 1.38–1.21 (m, 3H), 0.87 (t, *J* = 7.3 Hz, 5H), 0.72–0.64 (m, 1H), 0.64–0.51 (m, 3H), 0.45–0.25 (m, 2H). **^{13}C NMR** (75.4 MHz,

CDCl$_3$): δ (ppm) = 139.9 (C), 137.5 (C), 133.0 (C), 132.0 (CH), 130.6 (CH), 130.2 (C), 127.5 (C), 121.7 (CH), 120.9 (CH), 118.9 (CH), 115.8 (C), 108.8 (CH), 64.8 (CH$_2$), 57.5 (CH), 35.4 (CH$_2$), 30.2 (CH$_3$), 22.8 (CH$_2$), 16.6 (CH), 13.8 (CH), 11.3 (CH$_3$), 6.6 (CH$_2$), 6.3 (CH$_2$), 6.2 (CH$_2$), 5.7 (CH$_2$). **HRMS** (ESI-TOF) *m/z*: $[M+H]^+$ Calcd for $C_{24}H_{32}NO^+$ 350.2478; Found 350.2468.

Procedure for the synthesis of 3-propargylindole derivative S26

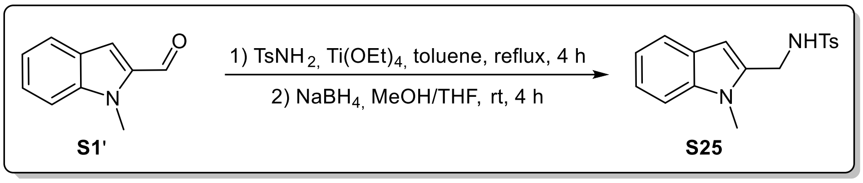

To a solution of the aldehyde **S1'** (477 mg, 3 mmol) in toluene (12 mL, 0.25 M) was added tosyl amide (769 mg, 4.5 mmol) and Ti(OEt)$_4$ (1.36 g, 6 mmol). The mixture was refluxed for 4 h before it was cooled to rt. After removal of the solvent, the residue was dissolved in MeOH/THF (1/1 v/v, 0.2 M). Then, NaBH$_4$ (456 mg, 12 mmol) was slowly added at 0 °C. After further stirring for 4 h at rt, the reaction was quenched by the addition of water, and the mixture was extracted with Et$_2$O (3 × 15 mL). The combined organic layers were dried over anhydrous Na$_2$SO$_4$, and the solvent was removed under reduced pressure. The residue was purified by silica gel column chromatography using a 3/1 (hexane/EtOAc) mixture as eluent to afford pure **S25** as a red solid (706 mg, 75%). M. p. = 147–149 °C (lit. M. p. = 151.4–152.1 °C).[13] The spectroscopic data for this compound match those reported in the literature.[13]

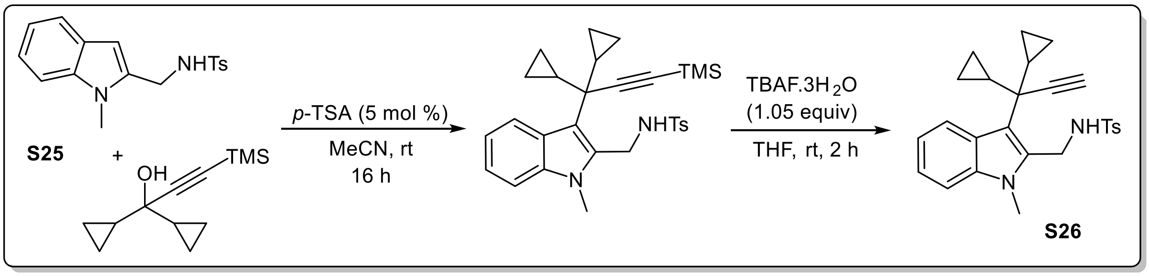

p-TSA (5 mol%) was added to a mixture of the 1,1-dicyclopropyl-3-(trimethylsilyl)prop-2-yn-1-ol (594 mg, 2.8 mmol) and indole derivative **S25** (706 mg, 2.2 mmol) in analytical-grade MeCN (4.4 mL, 0.5 M). The reaction mixture was stirred at rt until complete consumption of the starting indole, as determined by GC–MS and/or TLC. The crude reaction mixture was neutralized by the addition of 1 M NaOH (5 mL). The mixture was extracted with Et$_2$O (3 × 15 mL), and the combined organic layers were dried over anhydrous Na$_2$SO$_4$. The resulting trimethylsilyl-functionalized indole intermediate was then treated with TBAF·3H$_2$O (727 mg, 2.31 mmol) in THF (5 mL) at rt for 2 h. The reaction was quenched by the addition of water, and the mixture was extracted with Et$_2$O (3 × 15 mL). The combined organic layers were dried over anhydrous Na$_2$SO$_4$, and the solvent was removed under reduced pressure. The residue was purified by silica gel column chromatography using a 5/1 (hexane/EtOAc) mixture as eluent to afford the 3-propargylindole derivative **S26**.

[13] Bandini, M.; Gualandi, A.; Monari, M.; Romaniello, A.; Savoia, D.; Tragni, M. *J. Organomet. Chem*. **2011**, *696*, 338–347

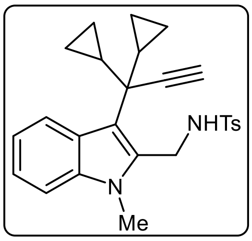

***N*-((3-(1,1-Dicyclopropylprop-2-yn-1-yl)-1-methyl-1*H*-indol-2-yl)methyl)-4-methylbenzenesulfonamide (S26)**: Yellow solid (570 mg, 60%). R_f = 0.32 (hexane/EtOAc, 5/1). M. p. = 120–122 °C. **^{1}H NMR** (500 MHz, $CDCl_3$): δ (ppm) = 8.07 (d, *J* = 8.2 Hz, 1H), 7.84 (d, *J* = 7.7 Hz, 2H), 7.38–7.30 (m, 3H), 7.26 (at, *J* = 7.6 Hz, 1H), 7.10 (at, *J* = 7.6 Hz, 1H), 4.67 (br s, 1H), 4.67 (d, *J* = 3.2 Hz, 2H), 3.77 (s, 3H), 2.48 (s, 3H), 1.71 (s, 1H), 1.65–1.54 (m, 2H), 0.71–0.64 (m, 2H), 0.62–0.52 (m, 2H), 0.46–0.26 (m, 4H). **^{13}C NMR** (125.7 MHz, $CDCl_3$): δ (ppm) = 143.9 (C), 137.1 (C), 136.5 (C), 130.1 (C), 129.9 (2 × CH), 127.3 (2 × CH), 125.7 (C), 122.1 (CH), 122.1 (CH), 119.2 (CH), 116.6 (C), 109.5 (CH), 84.6 (CH), 71.9 (C), 42.8 (C), 38.1 (CH_2), 29.5 (CH_3), 21.6 (CH_3), 21.1 (CH), 3.6 (2 × CH_2), 2.4 (2 × CH_2). **HRMS** (ESI-TOF) *m/z*: $[M+H]^+$ Calcd for $C_{26}H_{29}N_2O_2S^+$ 433.1944; Found 433.1947.

Reactivity of 3-propargylindole S26 under gold(I)-catalysis

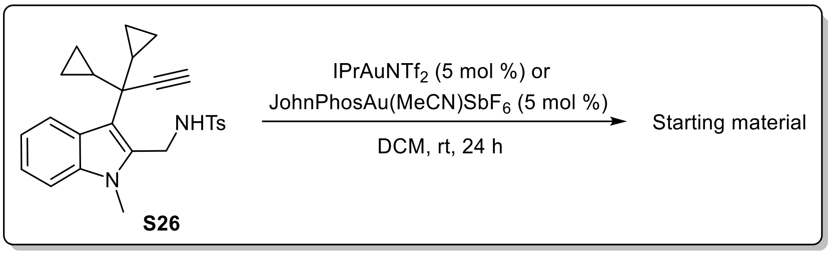

$IPrAuNTf_2$ (21 mg, 0.025 mmol, 5 mol%) or JohnPhosAu(MeCN)SbF_6 (19.5 mg, 0.025 mmol, 5 mol%) was dissolved in DCM (1 mL), and the resulting solution was stirred at rt for 5 min. A solution of 3-propargylindole **S26** (216 mg, 0.5 mmol) in DCM (1 mL) was then added. After stirring at rt for 24 h, only starting material **S26** was recovered with both catalytic systems.

Attempt of intermolecular reaction of 3-propargylindole S27 with MeOH under gold(I)-catalysis

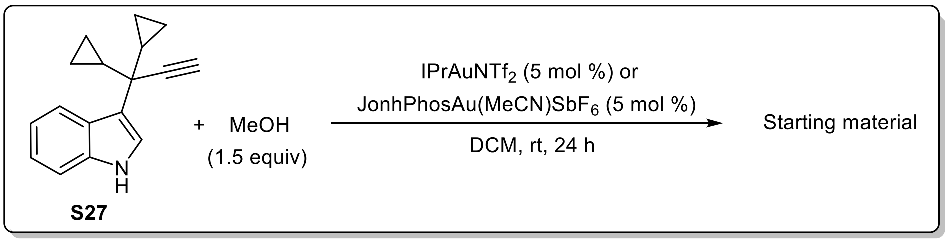

$IPrAuNTf_2$ (21 mg, 0.025 mmol) or JonhPhosAu(MeCN)SbF_6 (19.5 mg, 0.025 mmol) was dissolved in DCM (1 mL), and the resulting solution was stirred at rt for 5 min. A solution of 3-propargyl indole **S27**[14] (117 mg, 0.5 mmol) in DCM (1 mL) and MeOH (24 mg, 0.75 mmol) was then added. The reaction was stirred at rt for 24 h. However, only starting material **S27** was recovered in both cases.

[14] Renedo, L.; Álvarez, E.; Solas, M.; Suárez-Pantiga, S.; Fernández-Rodríguez, M. A.; Sanz, R. *Adv. Synth. Catal.* **2024**, *366*, 2079–2089.

HPLC Traces

Column: Chiralpak OD-H; Eluent hex/*i*-PrOH 95/5; Flow 0.5 mL/min

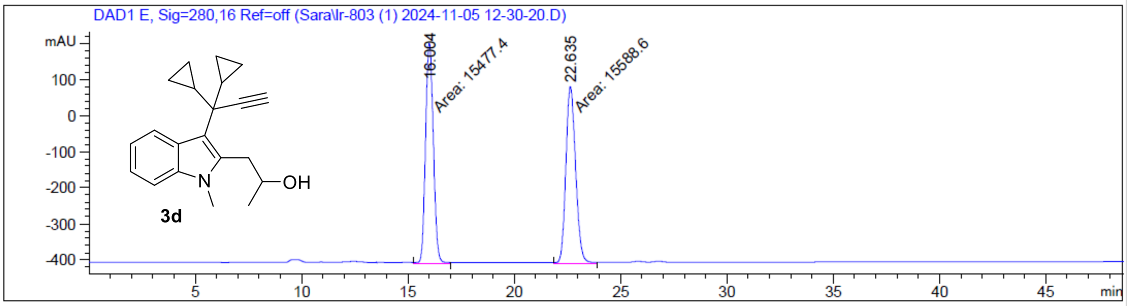

Peak #	RetTime [min]	Type	Width [min]	Area [mAU*s]	Height [mAU]	Area %
1	16.004	MM	0.4229	1.54774e4	610.01544	49.8212
2	22.635	MM	0.5309	1.55886e4	489.41818	50.1788

Totals : 3.10660e4 1099.43362

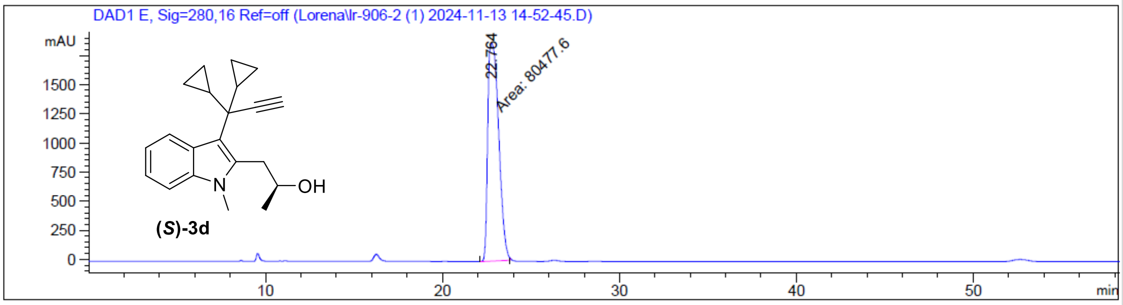

Peak #	RetTime [min]	Type	Width [min]	Area [mAU*s]	Height [mAU]	Area %
1	22.764	MM	0.7175	8.04776e4	1869.49854	100.0000

Totals : 8.04776e4 1869.49854

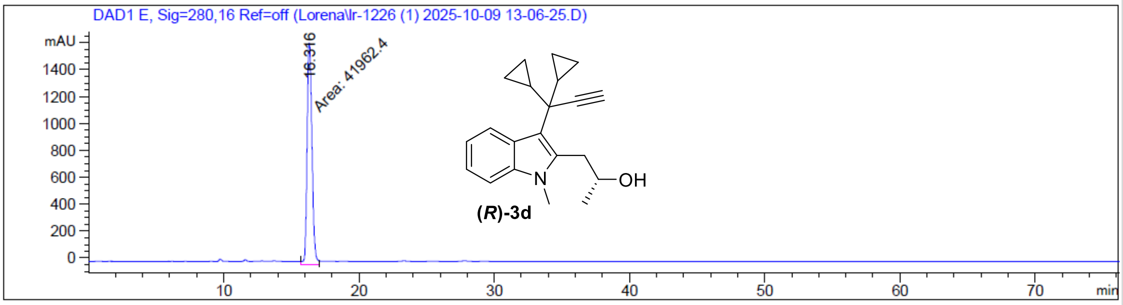

Peak #	RetTime [min]	Type	Width [min]	Area [mAU*s]	Height [mAU]	Area %
1	16.316	MM	0.4242	4.19624e4	1648.50903	100.0000

Totals : 4.19624e4 1648.50903

Column: Chiralpak OD-H; Eluent hex/*i*-PrOH 95/5; Flow 0.5 mL/min

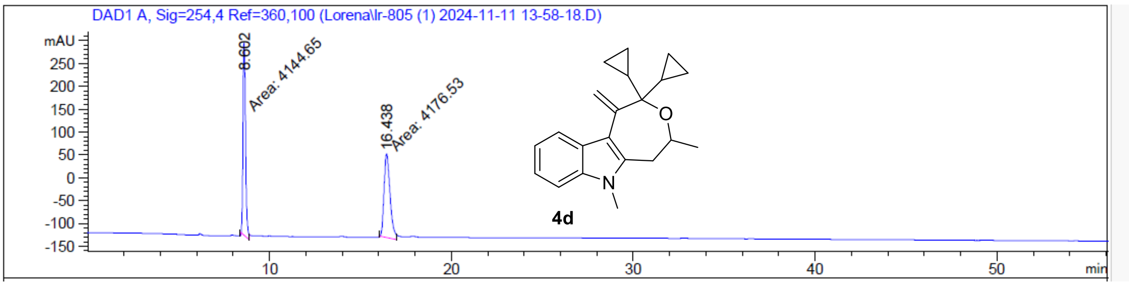

Peak #	RetTime [min]	Type	Width [min]	Area [mAU*s]	Height [mAU]	Area %
1	8.602	MM	0.1634	4144.64551	422.87900	49.8084
2	16.438	MM	0.3813	4176.52930	182.56386	50.1916

Totals : 8321.17480 605.44286

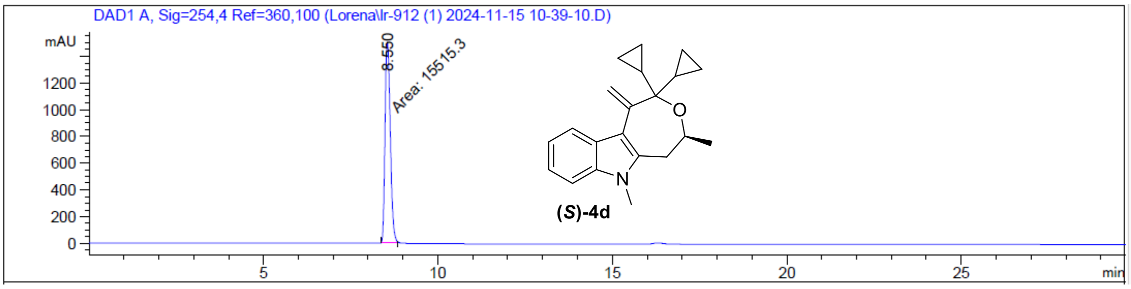

Peak #	RetTime [min]	Type	Width [min]	Area [mAU*s]	Height [mAU]	Area %
1	8.550	MM	0.1726	1.55153e4	1498.10938	100.0000

Totals : 1.55153e4 1498.10938

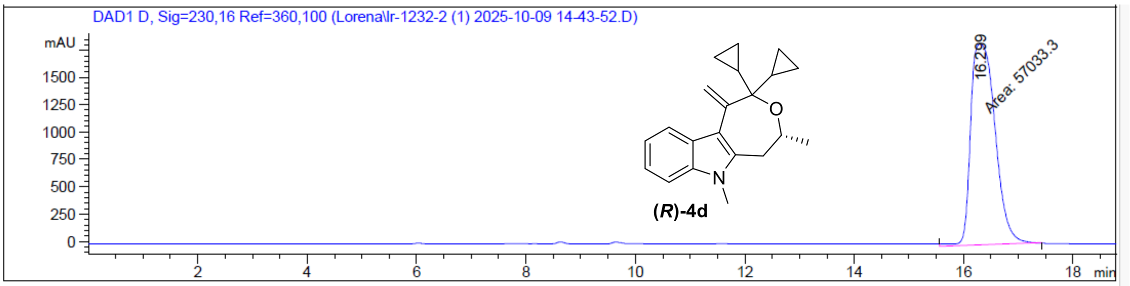

Peak #	RetTime [min]	Type	Width [min]	Area [mAU*s]	Height [mAU]	Area %
1	16.299	MM	0.5176	5.70333e4	1836.54114	100.0000

Totals : 5.70333e4 1836.54114

X-Ray Crystallographic Data for 2k

A single crystal of CCDC 2533493 (**2k**) suitable for X-Ray crystallography was obtained by crystallization from a hexane/CH_2Cl_2 solution. The crystal was kept at 299.0 K during data collection on a Bruker D8 VENTURE PhotonIII area-detector diffractometer and the data were corrected for absorption effects using the Multi-Scan method (SADABS). The structure was solved with ShelXT[15a] using direct methods, and refined with the ShelXL[15b] package using Least Squares minimization within the OLEX2 suite.[16]

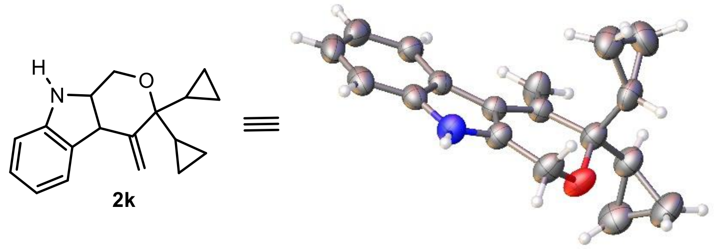

Figure S1. *X-ray structure of **2k**, ellipsoids at 50%*

**Table S3: Crystal data and structure refinement for 2k (CCDC 2533493)**	
Identification code	**2k**
Empirical formula	$C_{18}H_{19}NO$
Formula weight	265.34
Temperature/K	299.0
Crystal system	orthorhombic
Space group	$P2_12_12_1$
a/Å	6.2856(3)
b/Å	7.8235(4)
c/Å	29.3805(11)
α/°	90
β/°	90
γ/°	90
Volume/Å^3	1444.80(11)
Z	4
ρ_{calc}g/cm^3	1.220
μ/mm^{-1}	0.075
F(000)	568.0
Crystal size/mm^3	0.5 × 0.4 × 0.4
Radiation	MoKα (λ = 0.71073)
2Θ range for data collection/°	5.388 to 72.848
Index ranges	-10 ≤ h ≤ 9, -7 ≤ k ≤ 13, -48 ≤ l ≤ 47
Reflections collected	25611
Independent reflections	6662 [R_{int} = 0.0763, R_{sigma} = 0.1059]
Data/restraints/parameters	6662/0/190
Goodness-of-fit on F^2	1.041
Final R indexes [I>=2σ (I)]	R_1 = 0.0749, wR_2 = 0.1114
Final R indexes [all data]	R_1 = 0.1928, wR_2 = 0.1458
Largest diff. peak/hole / e Å^{-3}	0.14/-0.18

[15] (a) Sheldrick, G. M. *Acta Cryst*. **2015**, *A71*, 3-8. (b) Sheldrick, G. M.; *Acta Cryst*. **2015**, *C71*, 3-8.
[16] Dolomanov, O. V.; Bourhis, L. J.; Gildea, R. J.; Howard, J. A. K.; Puschmann, H. *J. Appl. Crystallogr*. **2009**, *42*, 339–341.

X-Ray Crystallographic Data for 4a

A single crystal of CCDC 2533495 (**4a**) suitable for X-Ray crystallography was obtained by crystallization from a hexane/CH_2Cl_2 solution. The crystal was kept at 298.0 K during data collection on a Bruker APEX-II CCD diffractometer and the data were corrected for absorption effects using the Multi-Scan method (SADABS). The structure was solved with the ShelXT[15a] using direct methods and refined with the ShelXL[15b] refinement package using Least Squares minimization within the OLEX2 suite.[16]

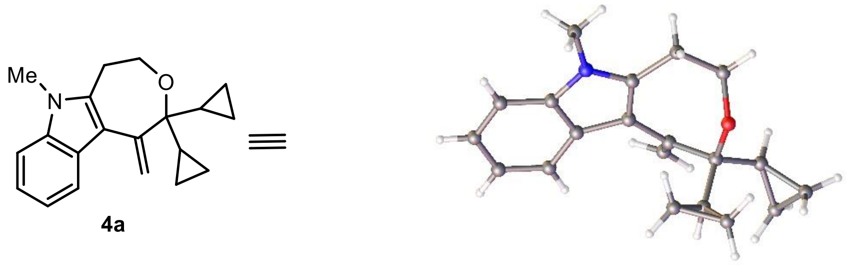

Figure S2. *X-ray structure of **4a**, ellipsoids at 50%*

Table S5: Crystal data and structure refinement for 4a (CCDC 2533495)

Identification code	**4a**
Empirical formula	$C_{20}H_{23}NO$
Formula weight	293.39
Temperature/K	298
Crystal system	monoclinic
Space group	C2/c
a/Å	20.6550(3)
b/Å	11.0668(13)
c/Å	13.8977(16)
α/°	90
β/°	90.189(7)
γ/°	90
Volume/Å^3	3176.8(7)
Z	8
ρ_{calc}g/cm^3	1.227
μ/mm^{-1}	0.514
F(000)	624.0
Crystal size/mm^3	0.4 × 0.3 × 0.08
Radiation	CuKα (λ = 1.54178)
2Θ range for data collection/°	9.066 to 146.226
Index ranges	-25 ≤ h ≤ 25, -13 ≤ k ≤ 13, -17 ≤ l ≤ 17
Reflections collected	40776
Independent reflections	3118 [R_{int} = 0.0764, R_{sigma} = 0.0296]
Data/restraints/parameters	3118/0/208
Goodness-of-fit on F^2	1.034
Final R indexes [I>=2σ (I)]	R_1 = 0.0522, wR_2 = 0.1436
Final R indexes [all data]	R_1 = 0.0559, wR_2 = 0.1497
Largest diff. peak/hole / e Å^{-3}	0.23/-0.21

Computational Studies

Methodology:

All the calculations in this work have been carried out with the M06[17] density functional together with the triple-z quality basis set Def2TZVP.[18] This functional together with a flexible basis set has been shown to provide good results in gold catalysis.[19] Dichloromethane solvation has been introduced in the simulations through the polarizable continuum method (PCM).[20] The default pruned grid with 99 radial shells and 590 angular points has been used for numerical integration of the functional in most cases. For some difficult transition states a finer grid with 175 radial shells and 974 angular points for first-row atoms and 250 radial shells and 974 angular points for atoms in the second and later rows was employed. All optimized structures were verified as minima or transition states of the potential energy surface through the analysis of the hessian matrix.

Cartesian coordinates

Scheme 5a

I

SCF Energy: -1308.20319825

ZPE-corrected Energy: -1307.80454

ΔU: -1307.77804

ΔH: -1307.77710

ΔG: -1307.86079

Num. Imaginary Frequencies: 0

C	1.635119	-1.014368	0.057482
C	2.332413	0.107723	0.628504
C	2.620129	0.982819	-0.443624
N	2.134807	0.432547	-1.596620
C	1.534637	-0.765706	-1.294314
C	2.739254	0.508507	1.910651
C	3.382279	1.715581	2.077679
C	3.648095	2.559376	0.994454
C	3.270684	2.201165	-0.279976
C	0.870953	-1.543526	-2.370229
H	2.560156	-0.108019	2.779373
H	3.690948	2.015812	3.072153
H	4.156457	3.501694	1.159944
H	3.465903	2.849713	-1.126073

[17] Zhao, Y., Truhlar, D.G. The M06 suite of density functionals for main group thermochemistry, thermochemical kinetics, noncovalent interactions, excited states, and transition elements: two new functionals and systematic testing of four M06-class functionals and 12 other functionals. *Theor Chem Account* **2008**, *120*, 215–241.

[18] Weigend, F.; Ahlrichs, R. Balanced basis sets of split valence, triple zeta valence and quadruple zeta valence quality for H to Rn: Design and assessment of accuracy, *Phys. Chem. Chem. Phys.* **2005**, *7*, 3297-305

[19] Nieto Faza, O.; Rodríguez, R.; Silva López, C. Performance of density functional theory on homogeneous gold catalysis. *Theor. Chem. Acc*. **2010**. *128*. 647-661.

[20] Cossi, M.; Barone, V.; Cammi, R.; Tomasi, J. Ab initio study of solvated molecules: A new implementation of the polarizable continuum model. *Chem. Phys. Lett.* **1996**, *255*, 327–335.

C	2.210468	1.077691	-2.884658
H	3.227355	1.428896	-3.065631
H	1.529087	1.932009	-2.936069
H	1.941903	0.379469	-3.672170
C	-0.319683	-2.460418	0.388247
C	1.095520	-2.256864	0.749916
C	1.126524	-2.161334	2.278892
H	0.598107	-1.277426	2.640590
H	2.158555	-2.134225	2.628997
H	0.650333	-3.045157	2.705968
C	-1.485489	-2.729831	0.151554
H	-2.440709	-3.159806	-0.063021
Au	-1.522892	-0.447653	0.232727
P	-1.884142	1.829678	0.158903
C	-0.333700	2.725000	0.389634
C	-3.026817	2.493650	1.385809
C	-2.504965	2.392516	-1.439417
C	1.888821	-3.507906	0.344333
H	2.927658	-3.370438	0.650123
H	1.492866	-4.394811	0.843718
H	1.870726	-3.676346	-0.731933
H	-3.109895	3.575408	1.260877
H	-4.008602	2.038037	1.259286
H	-2.659863	2.270335	2.387278
H	-0.515292	3.801486	0.356555
H	0.122629	2.455425	1.343247
H	0.357469	2.447810	-0.409007
H	-2.579867	3.482329	-1.442901
H	-1.818414	2.069592	-2.223723
H	-3.486642	1.960128	-1.631343
H	1.573373	-1.710203	-3.198539
H	0.585472	-2.530709	-1.996647
O	-0.263996	-0.820750	-2.812177
H	-0.656245	-1.287838	-3.555902

TSII-II

SCF Energy: -1308.17670179

ZPE-corrected Energy: -1307.77926

ΔU: -1307.75276

ΔH: -1307.75182

ΔG: -1307.83798

Num. Imaginary Frequencies: 1

Imaginary Frequency: -279.0738

C	-2.494940	-0.101383	0.725611

C	-3.473841	-0.992734	0.123395
C	-4.490917	-0.169325	-0.378314
N	-4.162228	1.151526	-0.113315
C	-2.986600	1.206675	0.530314
C	-3.615080	-2.371502	-0.032603
C	-4.731796	-2.865384	-0.676792
C	-5.723371	-2.019185	-1.171235
C	-5.615670	-0.651952	-1.027071
C	-2.353199	2.529808	0.791360
H	-2.871740	-3.064222	0.332551
H	-4.837340	-3.936302	-0.801522
H	-6.585899	-2.438851	-1.674081
H	-6.378022	0.015133	-1.409769
C	-4.998245	2.261619	-0.521966
H	-6.029428	2.053138	-0.237653
H	-4.944259	2.401384	-1.602418
H	-4.674075	3.175197	-0.035214
C	-0.749225	-0.002910	0.326275
C	-1.243789	-0.480195	1.561290
C	-1.032217	-1.968558	1.774438
H	-0.061303	-2.097407	2.257748
H	-1.007446	-2.521498	0.837761
H	-1.799085	-2.384977	2.430007
C	-0.038091	0.327969	-0.686304
H	-0.447647	0.694920	-1.619895
Au	2.025665	0.074614	-0.389941
P	4.318273	-0.217739	-0.079712
C	4.879518	-0.009435	1.628230
C	5.351195	0.929435	-1.023656
C	4.932604	-1.853493	-0.550828
C	-1.005032	0.261769	2.861756
H	-1.625021	-0.181703	3.643013
H	0.042083	0.139609	3.148299
H	-1.222329	1.324295	2.821008
H	6.406662	0.729498	-0.825357
H	5.154025	0.809662	-2.088971
H	5.117500	1.955048	-0.737703
H	5.960878	-0.152497	1.684784
H	4.629234	0.992405	1.977304
H	4.384205	-0.738182	2.269994
H	6.009800	-1.911687	-0.379655
H	4.430512	-2.619098	0.040799
H	4.723793	-2.033424	-1.605519
H	-2.995771	3.110401	1.470116
H	-1.391621	2.396263	1.287215
O	-2.191239	3.171643	-0.451313
H	-1.791668	4.033333	-0.301502

II

SCF Energy: -1308.17902591

ZPE-corrected Energy: -1307.78088

ΔU: -1307.75396

ΔH: -1307.75302

ΔG: -1307.84037

Num. Imaginary Frequencies: 0

C	-2.351806	0.056946	0.344216
C	-3.289416	-1.026332	0.031631
C	-4.523483	-0.427255	-0.209731
N	-4.369908	0.964825	-0.095586
C	-3.114916	1.264012	0.180255
C	-3.177645	-2.399400	-0.121965
C	-4.305409	-3.124574	-0.466470
C	-5.533891	-2.503390	-0.669587
C	-5.660699	-1.131291	-0.553973
C	-2.620620	2.669230	0.193465
H	-2.226256	-2.902639	-0.009466
H	-4.226268	-4.197373	-0.593295
H	-6.396976	-3.097974	-0.941594
H	-6.605077	-0.636958	-0.744041
C	-5.490027	1.874119	-0.269600
H	-6.335507	1.487123	0.297417
H	-5.758882	1.937384	-1.324100
H	-5.229701	2.862494	0.090823
C	-0.829213	0.027656	0.143989
C	-1.208695	-0.059596	1.506301
C	-1.164188	-1.386550	2.215417
H	-0.233910	-1.419954	2.788482
H	-1.157129	-2.233956	1.536119
H	-1.994625	-1.494209	2.916710
C	0.062373	0.069783	-0.819278
H	-0.315606	0.157448	-1.836518
Au	2.097624	-0.024517	-0.413254
P	4.393131	-0.120797	0.049982
C	4.871212	0.527458	1.672968
C	5.435912	0.804953	-1.106622
C	5.103094	-1.787628	0.028245
C	-1.037800	1.093737	2.461152
H	-1.870235	1.151448	3.166121
H	-0.128427	0.912024	3.039341
H	-0.914963	2.055447	1.970846
H	6.487560	0.704852	-0.828390
H	5.291484	0.421563	-2.116915

H	5.156297	1.858602	-1.090722
H	5.952984	0.452549	1.804710
H	4.569364	1.571806	1.754651
H	4.372027	-0.042234	2.457064
H	6.171271	-1.745066	0.253116
H	4.601801	-2.409478	0.770234
H	4.958561	-2.234312	-0.955658
H	-2.946944	3.157432	1.125678
H	-1.527643	2.648326	0.195970
O	-3.112428	3.318829	-0.947782
H	-2.802361	4.228801	-0.940390

TSII-III

SCF Energy: -1308.17152683

ZPE-corrected Energy: -1307.77491

ΔU: -1307.74833

ΔH: -1307.74739

ΔG: -1307.83417

Num. Imaginary Frequencies: 1

Imaginary Frequency: -106.3430

C	-2.381558	0.073569	-0.020460
C	-3.321999	-1.007111	-0.129668
C	-4.597665	-0.427450	-0.149640
N	-4.450561	0.947987	-0.041250
C	-3.137127	1.250731	0.025094
C	-3.192676	-2.385408	-0.259022
C	-4.339340	-3.145855	-0.386094
C	-5.601486	-2.549985	-0.395469
C	-5.751718	-1.179498	-0.284274
C	-2.653365	2.653695	0.058646
H	-2.211826	-2.848777	-0.278512
H	-4.260443	-4.221068	-0.491265
H	-6.481531	-3.172175	-0.503935
H	-6.732156	-0.719998	-0.314413
C	-5.568896	1.865509	-0.016660
H	-6.320548	1.496611	0.681580
H	-6.015544	1.953496	-1.008453
H	-5.237446	2.848297	0.303523
C	-0.906818	-0.008884	0.062494
C	-0.898060	0.055696	1.499211
C	-1.171705	-1.129718	2.326162
H	-0.178222	-1.539882	2.561925
H	-1.725508	-1.909966	1.809940
H	-1.645523	-0.879307	3.276786

C	0.060540	-0.017668	-0.848947
H	-0.296948	-0.020714	-1.879365
Au	2.083345	-0.070614	-0.411466
P	4.381046	-0.117281	0.074723
C	5.286219	1.377949	-0.403095
C	5.298745	-1.452259	-0.736410
C	4.789773	-0.324700	1.828051
C	-0.504128	1.281623	2.218656
H	0.296707	1.000041	2.913261
H	-0.145538	2.069255	1.560418
H	-1.315300	1.652237	2.852689
H	6.354083	-1.404748	-0.458536
H	4.888608	-2.416228	-0.434347
H	5.205006	-1.357209	-1.818348
H	6.343322	1.276811	-0.147039
H	5.188986	1.536656	-1.477282
H	4.869462	2.241666	0.115593
H	5.873815	-0.341388	1.961925
H	4.368029	0.498829	2.404806
H	4.367235	-1.260436	2.194851
H	-2.966717	3.134773	0.998128
H	-1.558714	2.639522	0.046847
O	-3.170301	3.326965	-1.063463
H	-2.899297	4.248345	-1.020819

III

SCF Energy: -1308.19079884

ZPE-corrected Energy: -1307.79346

ΔU: -1307.76632

ΔH: -1307.76537

ΔG: -1307.85317

Num. Imaginary Frequencies: 0

C	2.440998	0.078052	0.037926
C	3.474604	-0.856632	-0.278985
C	4.618795	-0.099813	-0.600374
N	4.297068	1.228035	-0.490438
C	2.982616	1.331247	-0.098459
C	3.540941	-2.249682	-0.332392
C	4.733073	-2.845952	-0.683481
C	5.862391	-2.076458	-0.990176
C	5.822557	-0.697956	-0.955336
C	2.357376	2.639905	0.196095
H	2.667735	-2.853823	-0.105561
H	4.801486	-3.926557	-0.726354

H	6.785857	-2.574659	-1.260817
H	6.699739	-0.107010	-1.191993
C	5.231030	2.306605	-0.697514
H	5.766682	2.160990	-1.636973
H	5.957254	2.360775	0.117079
H	4.700471	3.253987	-0.748379
C	1.062931	-0.258644	0.451528
C	0.877860	-0.851251	1.712858
C	1.991543	-0.877252	2.674566
H	2.637551	-0.003488	2.577299
H	2.619165	-1.749545	2.445143
H	1.636701	-0.999023	3.697540
C	-0.008835	0.083580	-0.355871
H	0.343806	0.609850	-1.248338
Au	-2.065474	0.003306	-0.261612
P	-4.418839	0.002284	-0.258342
C	-5.154555	1.081220	0.994828
C	-5.161960	-1.617825	0.055759
C	-5.173038	0.544089	-1.810998
C	-0.359400	-1.492792	2.176406
H	-0.101898	-2.418161	2.700306
H	-0.820442	-0.849522	2.935639
H	-1.084492	-1.694193	1.393159
H	-6.251289	-1.539441	0.045636
H	-4.844008	-2.322579	-0.712711
H	-4.835630	-1.988835	1.027506
H	-6.244201	1.038210	0.932521
H	-4.836311	0.761416	1.987199
H	-4.824265	2.107651	0.835154
H	-6.261636	0.514031	-1.727622
H	-4.855827	1.561813	-2.038581
H	-4.854793	-0.111011	-2.621933
H	2.511349	3.338441	-0.637673
H	1.274127	2.496229	0.296646
O	2.920996	3.145589	1.390899
H	2.586875	4.035459	1.536185

III’

SCF Energy: -1308.19067336

ZPE-corrected Energy: -1307.79322

ΔU: -1307.76613

ΔH: -1307.76519

ΔG: -1307.85313

Num. Imaginary Frequencies: 0

C	2.474753	-0.232891	0.206479
C	3.101982	1.049281	0.255111
C	4.417863	0.874239	-0.216685
N	4.587636	-0.451653	-0.524428
C	3.408324	-1.115059	-0.276468
C	2.679513	2.316879	0.656360
C	3.563539	3.370863	0.564171
C	4.864226	3.180266	0.082402
C	5.309938	1.935529	-0.313878
C	3.256334	-2.562491	-0.548101
H	1.672854	2.468148	1.033551
H	3.251674	4.363113	0.868174
H	5.535378	4.028707	0.019079
H	6.316877	1.797261	-0.689885
C	5.800008	-1.010758	-1.069900
H	6.662496	-0.599641	-0.543943
H	5.896918	-0.789454	-2.135475
H	5.802866	-2.090369	-0.942103
C	1.071680	-0.526412	0.559536
C	0.791086	-1.307921	1.689609
C	1.856193	-1.631647	2.655386
H	2.695382	-0.939492	2.610461
H	2.243976	-2.632716	2.423090
H	1.449367	-1.697774	3.666432
C	0.063833	0.075469	-0.177729
H	0.494034	0.757868	-0.917897
Au	-1.994941	0.114161	-0.184593
P	-4.341426	0.263576	-0.275790
C	-5.158648	0.192910	1.337196
C	-5.128260	-1.056305	-1.231593
C	-4.965704	1.787775	-1.023307
C	-0.526937	-1.870478	2.025047
H	-0.394960	-2.867162	2.453665
H	-0.971685	-1.262058	2.821697
H	-1.222125	-1.907621	1.190422
H	-6.211802	-0.918913	-1.233665
H	-4.759756	-1.037292	-2.257328
H	-4.887415	-2.023668	-0.790468
H	-6.240971	0.269641	1.211808
H	-4.918199	-0.749290	1.829958
H	-4.809037	1.014663	1.962339
H	-6.057769	1.776944	-1.034262
H	-4.618697	2.647444	-0.449986
H	-4.594185	1.874859	-2.044308
H	3.994481	-3.135622	0.032986
H	2.263851	-2.878013	-0.200040
O	3.410846	-2.794147	-1.931571
H	3.449647	-3.742710	-2.082876

TSIII-IV

SCF Energy: -1308.17896610

ZPE-corrected Energy: -1307.77957

ΔU: -1307.75388

ΔH: -1307.75293

ΔG: -1307.83636

Num. Imaginary Frequencies: 1

Imaginary Frequency: -235.5512

C	-2.531278	0.039466	0.219487
C	-3.792798	0.555285	-0.233315
C	-4.702096	-0.522472	-0.218628
N	-4.033604	-1.646904	0.203076
C	-2.732915	-1.296336	0.443479
C	-4.233055	1.793953	-0.702702
C	-5.545541	1.932379	-1.102545
C	-6.437170	0.855706	-1.048163
C	-6.027324	-0.387027	-0.610670
C	-1.693911	-2.285334	0.812190
H	-3.551857	2.634732	-0.763829
H	-5.894493	2.890551	-1.469283
H	-7.463285	0.997149	-1.366240
H	-6.711352	-1.227538	-0.587657
C	-4.628721	-2.954282	0.326438
H	-4.902054	-3.356160	-0.652073
H	-5.525661	-2.906426	0.946521
H	-3.930104	-3.641268	0.799088
C	-1.193348	0.652575	0.309000
C	-0.963621	1.855032	0.887136
C	-2.039627	2.619594	1.580859
H	-2.947058	2.043577	1.745528
H	-2.297637	3.527083	1.026336
H	-1.658396	2.960690	2.548434
C	-0.144970	-0.244018	-0.204769
H	-0.492170	-0.778826	-1.091203
Au	1.955466	-0.115498	-0.180826
P	4.295905	-0.055253	-0.225090
C	5.065133	0.297384	1.375247
C	4.981647	1.202361	-1.331647
C	5.081478	-1.593985	-0.764033
C	0.340986	2.573224	0.926329
H	0.182289	3.621489	0.656305
H	0.734051	2.579897	1.948431
H	1.101288	2.165285	0.264386

H	6.073246	1.170911	-1.305999
H	4.638712	1.020085	-2.350200
H	4.640931	2.189882	-1.019791
H	6.152383	0.319323	1.273435
H	4.717620	1.262994	1.743239
H	4.785845	-0.472348	2.095005
H	6.168030	-1.482654	-0.755813
H	4.796686	-2.405810	-0.094702
H	4.750942	-1.840472	-1.773205
H	-1.860309	-2.750975	1.787335
H	-1.615748	-3.086837	0.072125
O	-0.397968	-1.649587	0.854617
H	-0.210532	-1.317193	1.744827

TSIII'-IV'

SCF Energy: -1308.18483881

ZPE-corrected Energy: -1307.78537

ΔU: -1307.75999

ΔH: -1307.75904

ΔG: -1307.84226

Num. Imaginary Frequencies: 1

Imaginary Frequency: -233.9791

C	5.503461	1.867703	-0.030845
C	4.559159	0.852090	-0.097962
C	3.179864	1.082515	0.080719
C	2.753398	2.381459	0.363831
C	3.687212	3.394149	0.437007
C	5.047735	3.142679	0.233975
N	4.751703	-0.491995	-0.312107
C	3.528892	-1.104579	-0.271786
C	2.530040	-0.193793	-0.040409
C	1.104953	-0.534247	0.045833
C	0.111735	0.320015	-0.280274
Au	-1.956312	0.278469	-0.162393
P	-4.295377	0.321182	0.005658
C	-4.980811	1.812312	0.771801
C	6.040559	-1.104138	-0.520136
C	3.357290	-2.567332	-0.431312
O	1.960557	-2.887719	-0.527033
C	0.832969	-1.883821	0.570634
C	-0.425276	-2.596618	0.234408
C	1.395327	-2.241724	1.899879
C	-4.977872	-1.028380	1.004190
C	-5.191316	0.193082	-1.563891

H	1.705890	2.597328	0.538318
H	3.362653	4.404036	0.658484
H	5.755867	3.960634	0.293764
H	6.558779	1.666864	-0.174617
H	6.694173	-0.923938	0.336060
H	6.519909	-0.701748	-1.415062
H	5.933094	-2.179397	-0.643493
H	2.341030	-1.749476	2.115073
H	1.486439	-3.321394	2.023357
H	0.663512	-1.889793	2.634125
H	0.500738	1.284140	-0.610827
H	-0.324395	-3.661527	0.443962
H	-1.217535	-2.205052	0.879092
H	-0.733152	-2.439877	-0.798688
H	-6.268708	0.214094	-1.384537
H	-4.917024	1.025827	-2.211751
H	-4.926721	-0.739168	-2.063269
H	-6.066744	-0.955774	1.049474
H	-4.697563	-1.986222	0.564500
H	-4.570839	-0.976513	2.014502
H	-6.069231	1.739569	0.827756
H	-4.574121	1.927883	1.776619
H	-4.706914	2.687311	0.182135
H	3.851201	-2.962164	-1.323159
H	3.738330	-3.129608	0.426932
H	1.650332	-2.705354	-1.427548

IV

SCF Energy: -1308.24716905

ZPE-corrected Energy: -1307.84492

ΔU: -1307.81956

ΔH: -1307.81862

ΔG: -1307.90098

Num. Imaginary Frequencies: 0

C	-1.775067	-0.473854	0.153796
C	-1.868862	0.903805	0.565200
C	-3.066917	1.399056	0.007761
N	-3.666740	0.388256	-0.710628
C	-2.880286	-0.716891	-0.627547
C	-1.057062	1.794011	1.273329
C	-1.463784	3.103872	1.430270
C	-2.672215	3.557020	0.896263
C	-3.488181	2.709764	0.174146
C	-3.143017	-1.998818	-1.326003

H	-0.111096	1.472801	1.698007
H	-0.835907	3.793101	1.982524
H	-2.968796	4.588443	1.043953
H	-4.419324	3.059224	-0.256330
C	-4.920257	0.518395	-1.412376
H	-4.859882	1.313595	-2.158205
H	-5.729969	0.751133	-0.717882
H	-5.158907	-0.412185	-1.921722
C	-0.769785	-1.531158	0.286782
C	-0.027453	-1.824935	1.416662
C	-0.254958	-1.137732	2.727901
H	-1.012786	-0.360220	2.684620
H	0.669140	-0.714026	3.129711
H	-0.584805	-1.894914	3.446763
C	-0.890007	-2.496212	-0.877589
H	-0.628738	-1.995366	-1.821311
Au	1.293126	-0.412000	0.064902
P	2.914098	1.040019	-0.740349
C	3.918396	1.767057	0.571464
C	2.164628	2.445965	-1.588646
C	4.090925	0.333641	-1.910400
C	0.806789	-3.066342	1.555677
H	0.210544	-3.843550	2.045913
H	1.183783	-3.467688	0.617528
H	1.662603	-2.869481	2.204782
H	2.940150	3.147611	-1.903189
H	1.613342	2.097383	-2.461907
H	1.470517	2.946794	-0.912100
H	4.629504	2.474065	0.138462
H	3.271517	2.289434	1.276943
H	4.460986	0.984777	1.101763
H	4.795417	1.104831	-2.229481
H	4.636901	-0.481828	-1.436535
H	3.559602	-0.054416	-2.779003
H	-4.137750	-2.392289	-1.097976
H	-3.096932	-1.844145	-2.417494
O	-2.215808	-2.976687	-0.929709
H	-0.251244	-3.369549	-0.767925

IV’

SCF Energy: -1308.25957665

ZPE-corrected Energy: -1307.85797

ΔU: -1307.83302

ΔH: -1307.83207

ΔG: -1307.91379

Num. Imaginary Frequencies: 0

C	3.869900	2.627737	0.338268
C	3.324005	1.361359	0.242768
C	2.202510	1.066032	-0.553215
C	1.603964	2.103591	-1.263608
C	2.140434	3.375511	-1.172459
C	3.260979	3.636065	-0.386459
N	3.737956	0.193268	0.874831
C	2.917459	-0.802958	0.509094
C	1.942266	-0.341459	-0.363455
C	0.944008	-1.215523	-0.855018
C	0.051324	-0.868011	-1.862530
Au	-1.496623	-0.061630	-0.507313
P	-3.175788	0.774908	0.855933
C	-3.329700	2.574709	0.863103
C	4.885410	0.109514	1.748274
C	3.016677	-2.218858	0.921146
O	2.247792	-3.018490	0.067504
C	0.905579	-2.592927	-0.177124
C	0.337159	-3.675136	-1.067431
C	0.122358	-2.537527	1.131524
C	-2.922686	0.322638	2.587804
C	-4.831636	0.172524	0.458390
H	0.731438	1.935058	-1.882450
H	1.681728	4.185604	-1.726625
H	3.659456	4.642164	-0.339435
H	4.737543	2.824618	0.956457
H	4.760546	0.782534	2.598048
H	5.793568	0.385857	1.210241
H	4.993233	-0.904983	2.123720
H	-0.942884	-2.405059	0.931195
H	0.439503	-1.717482	1.779222
H	0.258731	-3.482232	1.662945
H	0.290957	-0.003518	-2.474056
H	0.425848	-4.631709	-0.551220
H	-0.718259	-3.502170	-1.279494
H	0.885503	-3.735795	-2.008509
H	-5.554674	0.584134	1.166066
H	-5.100610	0.478471	-0.552589
H	-4.849596	-0.916027	0.513225
H	-3.730900	0.723805	3.203213
H	-2.901402	-0.763726	2.680339
H	-1.969235	0.722767	2.934150
H	-4.116316	2.875457	1.558978
H	-2.384491	3.023625	1.167933
H	-3.578790	2.925513	-0.138262
H	4.045715	-2.581986	0.851739

H	2.711405	-2.332173	1.974129
H	-0.486070	-1.656194	-2.378498

TS1-PF1

SCF Energy: -1308.17717074

ZPE-corrected Energy: -1307.77807

ΔU: -1307.75264

ΔH: -1307.75170

ΔG: -1307.83517

Num. Imaginary Frequencies: 1

Imaginary Frequency: -306.3481

C	-2.695978	-0.088833	0.347048
C	-3.651732	0.971130	0.185040
C	-3.737994	2.309236	0.584895
C	-4.854240	3.045971	0.250988
C	-5.905272	2.482161	-0.478616
C	-5.861916	1.163342	-0.877534
C	-4.737289	0.423717	-0.534589
N	-4.470675	-0.898176	-0.784942
C	-3.252135	-1.193349	-0.233360
C	-2.695142	-2.563032	-0.214996
O	-1.759689	-2.573806	0.872673
H	-1.254289	-3.398965	0.873180
H	-2.180978	-2.831984	-1.145719
H	-3.464470	-3.313627	-0.014495
H	-2.951050	2.775259	1.165075
H	-4.920965	4.081356	0.563583
H	-6.769565	3.087264	-0.725165
H	-6.682219	0.717590	-1.427992
C	-5.362996	-1.797254	-1.473826
H	-4.844353	-2.718119	-1.731906
H	-5.709485	-1.341414	-2.402586
H	-6.232893	-2.043372	-0.859786
C	-1.350540	-0.047135	1.035043
C	-0.563926	1.192578	0.584948
H	0.422741	1.214917	1.053141
H	-1.096340	2.091836	0.888641
H	-0.440320	1.210283	-0.499243
C	-1.514722	-0.008492	2.559024
H	-2.025333	-0.900765	2.921310
H	-0.539076	0.063339	3.045644
H	-2.108797	0.862860	2.841089
C	-0.531534	-1.219790	0.644633
C	0.596729	-1.689150	0.268941

H	0.770892	-2.746580	0.104936
Au	2.288027	-0.469199	-0.040189
P	4.156505	0.868393	-0.377905
C	3.795629	2.626883	-0.153296
H	3.432085	2.797793	0.860441
H	3.021536	2.933074	-0.857628
H	4.695650	3.222522	-0.320917
C	4.882413	0.763984	-2.030761
H	4.135991	1.034226	-2.777695
H	5.733919	1.443892	-2.106487
H	5.216135	-0.256064	-2.221113
C	5.540368	0.545143	0.740206
H	6.360065	1.234956	0.526885
H	5.886687	-0.479835	0.607922
H	5.216598	0.675369	1.772874

PF1

SCF Energy: -1308.17977515

ZPE-corrected Energy: -1307.77872

ΔU: -1307.75338

ΔH: -1307.75244

ΔG: -1307.83492

Num. Imaginary Frequencies: 0

C	-2.732714	-0.052463	0.285168
C	-3.731421	0.968256	0.146081
C	-3.816868	2.326871	0.467697
C	-4.975246	3.016368	0.181041
C	-6.066068	2.383978	-0.424968
C	-6.021552	1.044181	-0.745935
C	-4.855472	0.350906	-0.446299
N	-4.574572	-0.978485	-0.637804
C	-3.298991	-1.197877	-0.189234
C	-2.664748	-2.528069	-0.148223
O	-1.610362	-2.396197	0.860726
H	-1.106776	-3.222921	0.955626
H	-2.193335	-2.839012	-1.085850
H	-3.330641	-3.315469	0.207962
H	-2.992199	2.843619	0.944331
H	-5.044188	4.068751	0.429712
H	-6.961085	2.955304	-0.640509
H	-6.867017	0.549236	-1.209478
C	-5.475500	-1.926907	-1.243610
H	-5.036549	-2.922270	-1.232996
H	-5.683849	-1.657959	-2.281517

H	-6.418912	-1.963675	-0.695486
C	-1.336292	0.038972	0.851778
C	-0.586821	1.192649	0.189340
H	0.414097	1.306439	0.612454
H	-1.123944	2.125659	0.354688
H	-0.492758	1.035747	-0.886956
C	-1.388882	0.271470	2.367831
H	-1.890094	-0.553953	2.876305
H	-0.376645	0.368162	2.768298
H	-1.939397	1.187928	2.589479
C	-0.605130	-1.247211	0.582827
C	0.604685	-1.632843	0.269874
H	0.766245	-2.708703	0.194124
Au	2.308411	-0.466739	-0.027139
P	4.219233	0.833327	-0.338786
C	3.903600	2.602327	-0.113868
H	3.534878	2.781284	0.896657
H	3.143569	2.929518	-0.824304
H	4.819310	3.176158	-0.272163
C	4.967398	0.725962	-1.983595
H	4.237903	1.018904	-2.738792
H	5.835409	1.386379	-2.043093
H	5.280069	-0.299947	-2.178545
C	5.591433	0.482865	0.788412
H	6.425605	1.157727	0.583017
H	5.920108	-0.548021	0.656176
H	5.263659	0.615979	1.819500

Scheme 5b: Reaction 1

Int1

SCF Energy: -1499.85153781

ZPE-corrected Energy: -1499.40081

ΔU: -1499.37153

ΔH: -1499.37059

ΔG: -1499.46209

Num. Imaginary Frequencies: 1

Imaginary Frequency: -15.1287

C	-2.698967	0.103477	0.080845
C	-3.428317	-1.101149	0.331345
C	-4.777488	-0.824385	0.037900
N	-4.869265	0.487627	-0.357135
C	-3.614275	1.042518	-0.335497
C	-3.076634	-2.370060	0.792158
C	-4.060963	-3.326308	0.923191
C	-5.393835	-3.037572	0.607642
C	-5.771635	-1.787033	0.163241
C	-3.375353	2.438755	-0.767732
H	-2.046356	-2.599895	1.044972
H	-3.803310	-4.317901	1.276062
H	-6.144438	-3.811544	0.716221
H	-6.804872	-1.569655	-0.080518
C	-6.093276	1.125212	-0.776559
H	-6.904565	0.834459	-0.108224
H	-6.358330	0.844869	-1.798621
H	-5.984217	2.206062	-0.733358
C	-1.247796	0.298677	0.194869
C	-0.759398	1.314800	1.055163
C	-1.571346	1.748777	2.212038
H	-2.357140	1.035095	2.451310
H	-2.043943	2.714200	1.996376
H	-0.942177	1.922972	3.085956
C	-0.376741	-0.597726	-0.394232
H	-0.917981	-1.374395	-0.941800
Au	1.678089	-0.797315	-0.303333
P	4.011564	-1.021089	-0.195475
C	4.714425	-0.434768	1.366495
C	4.879910	-0.048416	-1.452083
C	4.674353	-2.693421	-0.385941
C	0.501533	1.965727	0.811090
H	5.960423	-0.150934	-1.329739
H	4.594063	-0.391212	-2.446594
H	4.601874	1.002010	-1.351200
H	5.801976	-0.534847	1.354748
H	4.447664	0.613915	1.508935
H	4.306183	-1.013850	2.195002

H	5.764691	-2.673147	-0.324631
H	4.279912	-3.335591	0.401613
H	4.373985	-3.099973	-1.351637
H	-3.967799	3.132680	-0.152630
H	-2.319260	2.685907	-0.590961
O	-3.705976	2.561882	-2.133122
H	-3.679151	3.492022	-2.374752
C	1.327553	2.390578	1.863498
C	2.543646	2.981486	1.603397
C	2.943565	3.209292	0.291881
C	2.126106	2.834547	-0.761138
C	0.922724	2.205656	-0.508831
H	1.036536	2.208077	2.889967
H	3.186107	3.273791	2.424728
H	3.893108	3.692927	0.094135
H	2.424385	3.034641	-1.782794
H	0.266034	1.943091	-1.328685

TSNaz1

SCF Energy: -1499.84021432

ZPE-corrected Energy: -1499.39009

ΔU: -1499.36065

ΔH: -1499.35971

ΔG: -1499.45245

Num. Imaginary Frequencies: 1

Imaginary Frequency: -259.0106

C	-2.512995	-0.099155	-0.043019
C	-3.823406	0.477212	-0.137132
C	-4.734247	-0.593898	-0.152621
N	-4.018707	-1.766086	-0.088755
C	-2.684297	-1.469599	-0.024938
C	-4.305538	1.781232	-0.244039
C	-5.666683	1.982862	-0.328938
C	-6.558295	0.904351	-0.317314
C	-6.107176	-0.396677	-0.232236
C	-1.670231	-2.540887	0.117373
H	-3.621827	2.623547	-0.264464
H	-6.055917	2.991045	-0.407555
H	-7.623131	1.094667	-0.380420
H	-6.800670	-1.229259	-0.226136
C	-4.619626	-3.077794	-0.067122
H	-5.435383	-3.116095	-0.789811
H	-5.011175	-3.318321	0.923692
H	-3.883393	-3.829828	-0.338474

C	-1.247540	0.622827	-0.013502
C	-1.004080	1.723814	0.793481
C	-1.793847	2.085095	1.997409
H	-2.640913	1.416346	2.135858
H	-2.157853	3.114191	1.957860
H	-1.163205	2.005734	2.888222
C	-0.222134	0.209778	-0.905522
H	-0.596747	-0.142313	-1.872215
Au	1.708222	-0.420785	-0.374249
P	3.833758	-1.148553	0.239590
C	3.921055	-1.964961	1.850666
C	5.021568	0.211594	0.359904
C	4.603494	-2.319611	-0.902766
C	0.127675	2.469232	0.376432
H	5.999904	-0.164448	0.667689
H	5.110163	0.703613	-0.609492
H	4.667523	0.941286	1.089379
H	4.953060	-2.248516	2.068382
H	3.560616	-1.287483	2.624816
H	3.294388	-2.856693	1.846268
H	5.600563	-2.587369	-0.546306
H	3.991994	-3.218910	-0.975922
H	4.682029	-1.868320	-1.891761
H	-1.730543	-3.229980	-0.737333
H	-0.669355	-2.089161	0.087414
O	-1.895375	-3.221578	1.333719
H	-1.337068	-4.003756	1.356154
C	0.928694	3.232186	1.249464
C	2.146312	3.695446	0.833538
C	2.606179	3.450974	-0.474249
C	1.830366	2.763308	-1.364165
C	0.562941	2.293674	-0.963278
H	0.602460	3.395748	2.269347
H	2.774432	4.247693	1.522111
H	3.566205	3.846284	-0.783622
H	2.150115	2.627633	-2.389746
H	-0.177358	2.142141	-1.745162

TSNuc1

SCF Energy: -1499.83855896

ZPE-corrected Energy: -1499.38562

ΔU: -1499.35733

ΔH: -1499.35638

ΔG: -1499.44535

Num. Imaginary Frequencies: 1

Imaginary Frequency: -207.3718

C	-6.022230	-1.496779	-0.161439
C	-4.933389	-0.637875	-0.104336
C	-3.596337	-1.088190	-0.102591
C	-3.373704	-2.467725	-0.136609
C	-4.450974	-3.328064	-0.190033
C	-5.763540	-2.850295	-0.209589
N	-4.934292	0.733265	-0.023231
C	-3.634098	1.150369	0.029555
C	-2.763934	0.088444	-0.021685
C	-1.304991	0.209840	0.025854
C	-0.437625	-0.783761	-0.248512
Au	1.625508	-0.892323	-0.049096
P	3.953580	-1.039689	0.173507
C	4.636531	-2.709061	0.339601
C	-6.126928	1.542129	0.031607
C	-3.267013	2.574112	0.168732
O	-1.853139	2.728978	-0.061157
C	-0.779547	1.490414	0.589829
C	0.494817	2.031558	0.050845
C	-1.030673	1.667305	2.051637
C	4.620054	-0.154200	1.607474
C	4.868871	-0.329782	-1.220578
H	-2.373031	-2.877917	-0.107929
H	-4.275265	-4.397067	-0.213964
H	-6.588110	-3.551711	-0.254715
H	-7.037670	-1.118167	-0.161358
H	-6.709795	1.312365	0.926356
H	-6.750726	1.364214	-0.846541
H	-5.867806	2.598151	0.056223
H	-2.015404	1.298473	2.333065
H	-0.913010	2.701205	2.376053
H	-0.296961	1.049266	2.575664
H	-0.925945	-1.706165	-0.552942
H	5.945268	-0.405771	-1.050376
H	4.608769	-0.857971	-2.138295
H	4.592428	0.720157	-1.333159
H	5.707719	-0.249954	1.643678
H	4.351116	0.900914	1.533866
H	4.190476	-0.560377	2.523509
H	5.724095	-2.665295	0.430853
H	4.218695	-3.188413	1.225140
H	4.370704	-3.302524	-0.535328
H	-3.778537	3.228257	-0.540959
H	-3.454103	2.968662	1.171760
H	-1.670368	2.775800	-1.013986
C	1.458701	2.591005	0.883282

C	2.632741	3.098695	0.354098
C	2.846359	3.079521	-1.013596
C	1.885501	2.540557	-1.855523
C	0.724564	2.012576	-1.326214
H	1.311445	2.617006	1.955299
H	3.379425	3.521206	1.015698
H	3.761364	3.488116	-1.426078
H	2.045994	2.522595	-2.926407
H	-0.003384	1.554347	-1.988099

PFNaz1

SCF Energy: -1499.87256082

ZPE-corrected Energy: -1499.41994

ΔU: -1499.39068

ΔH: -1499.38974

ΔG: -1499.48166

Num. Imaginary Frequencies: 0

C	-2.143847	-0.001986	-0.276356
C	-3.496628	0.347512	0.087593
C	-4.257177	-0.829241	0.006390
N	-3.428192	-1.846208	-0.406103
C	-2.172131	-1.354928	-0.602331
C	-4.155047	1.544648	0.374290
C	-5.508725	1.521263	0.633164
C	-6.233095	0.325708	0.599089
C	-5.619006	-0.865500	0.276309
C	-1.097581	-2.274869	-1.048677
H	-3.619578	2.485974	0.381686
H	-6.023642	2.446679	0.862221
H	-7.294890	0.338391	0.813800
H	-6.181249	-1.790095	0.226043
C	-3.860318	-3.217041	-0.543097
H	-4.834968	-3.244098	-1.030407
H	-3.937322	-3.700523	0.432916
H	-3.155557	-3.776989	-1.151398
C	-1.037112	0.921117	-0.303310
C	-0.896419	2.021028	0.606402
C	-1.511606	2.159939	1.956458
H	-2.114757	1.292905	2.219598
H	-2.145712	3.045960	2.039727
H	-0.727169	2.260111	2.711574
C	0.009611	0.956283	-1.275609
H	-0.206286	0.535421	-2.255646
Au	1.531990	-0.296949	-0.289465

P	3.187641	-1.568127	0.718956
C	2.676175	-2.294491	2.293070
C	4.704212	-0.661971	1.098381
C	3.724620	-2.976007	-0.279852
C	0.064236	2.863634	0.108292
H	5.424175	-1.324817	1.583310
H	5.136086	-0.267812	0.178447
H	4.474677	0.170876	1.763132
H	3.484153	-2.903872	2.704408
H	2.425852	-1.501100	2.997283
H	1.793309	-2.915349	2.137734
H	4.497529	-3.538383	0.248579
H	2.872629	-3.628477	-0.474363
H	4.120748	-2.623244	-1.232087
H	-1.447681	-2.834808	-1.926606
H	-0.224236	-1.701474	-1.374231
O	-0.770673	-3.145455	0.015724
H	-0.181526	-3.828647	-0.318068
C	0.704851	3.962495	0.744690
C	1.893459	4.393568	0.265779
C	2.528009	3.778703	-0.874913
C	1.921246	2.803368	-1.562127
C	0.542794	2.390908	-1.211794
H	0.280389	4.390348	1.645538
H	2.413836	5.199672	0.770191
H	3.508516	4.130101	-1.172615
H	2.381931	2.361739	-2.439138
H	-0.081515	2.904676	-1.972352

PFNuc1

SCF Energy: -1499.83899005

ZPE-corrected Energy: -1499.38491

ΔU: -1499.35632

ΔH: -1499.35538

ΔG: -1499.44546

Num. Imaginary Frequencies: 0

C	6.048397	-1.423107	0.272127
C	4.941080	-0.606864	0.086119
C	3.612649	-1.070628	0.200043
C	3.420771	-2.415913	0.529452
C	4.516426	-3.232355	0.718916
C	5.819026	-2.744556	0.590094
N	4.916985	0.731121	-0.221507
C	3.607695	1.111796	-0.308631

C	2.755875	0.059994	-0.070619
C	1.298999	0.162463	-0.124655
C	0.434743	-0.852287	0.041981
Au	-1.636077	-0.901585	-0.089206
P	-3.976527	-0.971609	-0.209589
C	-4.726568	-2.612877	-0.371269
C	6.092637	1.540235	-0.426595
C	3.186840	2.491591	-0.606975
O	1.806204	2.646902	-0.171522
C	0.745545	1.497025	-0.583255
C	-0.460636	2.002284	0.153558
C	0.707953	1.609187	-2.080556
C	-4.676068	-0.038297	-1.597705
C	-4.807594	-0.258068	1.234798
H	2.430326	-2.834363	0.643933
H	4.362910	-4.274444	0.973051
H	6.658645	-3.411945	0.743498
H	7.055343	-1.035371	0.171809
H	6.660878	1.187683	-1.290082
H	6.737700	1.507189	0.453557
H	5.812264	2.575906	-0.604576
H	1.629025	1.232343	-2.524806
H	0.529152	2.630947	-2.417486
H	-0.100138	0.968273	-2.436124
H	0.911487	-1.808289	0.236798
H	-5.892096	-0.299610	1.110477
H	-4.524015	-0.809760	2.131493
H	-4.495015	0.781070	1.352753
H	-5.767408	-0.084243	-1.578429
H	-4.356908	1.003077	-1.528960
H	-4.313785	-0.452095	-2.539102
H	-5.815025	-2.529306	-0.411498
H	-4.366822	-3.092275	-1.281880
H	-4.443356	-3.229926	0.481706
H	3.754937	3.256445	-0.075083
H	3.187373	2.750349	-1.668448
H	1.760276	2.759913	0.795556
C	-1.540687	2.578097	-0.503403
C	-2.620927	3.060225	0.216947
C	-2.623265	2.994466	1.599688
C	-1.543145	2.436326	2.265567
C	-0.473917	1.938648	1.546067
H	-1.560099	2.637868	-1.584339
H	-3.462646	3.495976	-0.308332
H	-3.467511	3.377955	2.160392
H	-1.538612	2.378853	3.347021
H	0.347585	1.459743	2.070825

Scheme 5b: Reaction 2

Int2

SCF Energy: -1539.15209593

ZPE-corrected Energy: -1538.67393

ΔU: -1538.64202

ΔH: -1538.64107

ΔG: -1538.74043

Num. Imaginary Frequencies: 0

C	2.224588	-0.777902	0.256318
C	2.582283	0.135171	1.295016
C	3.988231	0.215309	1.300723
N	4.465172	-0.616843	0.320480
C	3.395869	-1.209723	-0.310909
C	1.849358	0.887705	2.212727
C	2.526532	1.699724	3.097139
C	3.925341	1.770884	3.084286
C	4.674750	1.033506	2.190584
C	3.580695	-2.105479	-1.474859
H	0.764191	0.834183	2.225563
H	1.973237	2.293758	3.814987
H	4.429281	2.420251	3.790506
H	5.756651	1.097455	2.182105
C	5.863112	-0.788455	0.011136
H	6.432721	-0.914711	0.933094
H	6.257822	0.073236	-0.532439
H	6.002555	-1.674550	-0.603002
C	0.835247	-1.124420	-0.117515
C	0.393794	-2.420662	0.079108
C	1.231414	-3.426646	0.772755
H	1.964006	-2.979219	1.441858
H	1.776011	-4.034010	0.039723
H	0.591837	-4.124496	1.318611
C	-0.031004	-0.072961	-0.534151
C	0.540103	1.130976	-1.088422
Au	-2.088223	-0.043972	-0.130921
P	-4.376500	0.013546	0.396257
C	-4.900116	-1.354741	1.458151
C	-5.475547	-0.086130	-1.037803
C	-4.923217	1.496589	1.275694
C	-0.883763	-2.969330	-0.429900
H	-1.552968	-3.173256	0.415139
H	-1.395284	-2.335283	-1.148315
H	-0.692424	-3.941269	-0.895250
H	-6.517570	-0.068147	-0.710874
H	-5.290127	0.758263	-1.701684
H	-5.284244	-1.009628	-1.584690
H	-5.971944	-1.289153	1.657626

H	-4.680477	-2.303434	0.967234
H	-4.354248	-1.314836	2.401068
H	-5.991762	1.428437	1.491580
H	-4.371210	1.594368	2.210551
H	-4.733515	2.377912	0.662880
H	4.216344	-2.962177	-1.206431
H	2.600467	-2.510138	-1.758899
O	4.152070	-1.363130	-2.534157
H	4.341982	-1.960973	-3.262868
C	-0.018219	2.384584	-0.793422
C	0.530221	3.543543	-1.299337
C	1.602132	3.474234	-2.177219
C	2.131090	2.241552	-2.534172
C	1.627029	1.085357	-1.979230
H	-0.873617	2.431971	-0.127334
H	0.110109	4.504106	-1.027908
H	2.017617	4.383068	-2.596221
H	2.949784	2.184487	-3.241377
H	2.049563	0.131188	-2.266652

TSNaz2

SCF Energy: -1539.13650015

ZPE-corrected Energy: -1538.65800

ΔU: -1538.62736

ΔH: -1538.62642

ΔG: -1538.72061

Num. Imaginary Frequencies: 1

Imaginary Frequency: -346.9600

C	-2.386937	3.233090	1.477208
C	-1.214501	2.918451	0.800566
C	-0.482247	1.748253	1.065265
C	-0.919610	0.894483	2.079635
C	-2.082618	1.200162	2.755923
C	-2.813584	2.354379	2.451712
N	-0.566665	3.612489	-0.194165
C	0.536340	2.904791	-0.582485
C	0.637166	1.745102	0.162168
C	1.618758	0.678393	0.029288
C	1.263496	-0.663768	0.030078
Au	-0.673585	-1.344273	-0.307164
P	-2.957168	-1.827269	-0.474603
C	-3.566192	-2.830644	-1.851870
C	-1.036730	4.863854	-0.738962
C	1.374168	3.370143	-1.712458

O	0.581302	3.420918	-2.877089
C	3.043906	0.968682	0.021272
C	3.904432	0.550596	-1.132219
C	3.564310	2.161717	0.748282
C	-3.900904	-0.284238	-0.579367
C	-3.631156	-2.635345	0.999243
H	-0.352301	0.006241	2.336337
H	-2.434130	0.541412	3.541565
H	-3.724814	2.566651	2.998207
H	-2.949099	4.130861	1.249004
H	-1.372740	5.509537	0.072952
H	-1.864488	4.706812	-1.433920
H	-0.232264	5.366942	-1.268758
H	2.935627	2.444536	1.592058
H	3.649731	3.020773	0.075114
H	4.577863	1.954214	1.101451
C	2.383412	-1.444355	0.414692
H	4.947766	0.443006	-0.831213
H	3.564167	-0.364680	-1.612780
H	3.874007	1.350603	-1.880730
H	-4.715428	-2.735901	0.913362
H	-3.183804	-3.621909	1.120281
H	-3.392382	-2.031975	1.876674
H	-4.973941	-0.488704	-0.586818
H	-3.653147	0.340776	0.282857
H	-3.627065	0.253787	-1.487567
H	-4.652776	-2.924723	-1.792031
H	-3.295296	-2.360807	-2.797294
H	-3.115730	-3.822470	-1.812198
H	2.215234	2.680183	-1.837817
H	1.802792	4.357083	-1.480334
H	1.096060	3.823021	-3.582513
C	2.678393	-2.711327	-0.118329
C	3.948140	-3.208574	-0.009989
C	4.947014	-2.508462	0.699502
C	4.653251	-1.338812	1.341236
C	3.362613	-0.784560	1.221785
H	1.918104	-3.226342	-0.694997
H	4.201769	-4.146805	-0.488850
H	5.937747	-2.939383	0.779250
H	5.393161	-0.848979	1.963636
H	3.026871	-0.124010	2.012885

TSNuc2

SCF Energy: -1539.13460161

ZPE-corrected Energy: -1538.65479

ΔU: -1538.62451

ΔH: -1538.62357

ΔG: -1538.71861

Num. Imaginary Frequencies: 1

Imaginary Frequency: -20.0208

C	5.606341	0.758234	0.884443
C	4.541363	-0.002111	0.419223
C	3.195653	0.359356	0.631835
C	2.927886	1.505970	1.381240
C	3.980076	2.262436	1.852502
C	5.306921	1.899232	1.599072
N	4.569211	-1.193858	-0.265471
C	3.274887	-1.578320	-0.480806
C	2.387789	-0.662036	0.020341
C	0.924221	-0.860451	-0.017882
C	0.000023	0.110145	-0.217736
Au	-2.090760	0.011588	0.004641
P	-4.416393	-0.045931	0.271775
C	-5.008321	0.211362	1.963730
C	5.772717	-1.899620	-0.629207
C	2.920793	-2.889589	-1.063100
O	1.478360	-3.022903	-1.096136
C	0.572990	-2.301721	0.163966
C	-0.772333	-2.826617	-0.222585
C	1.103209	-2.980235	1.388461
C	-5.119684	-1.651030	-0.189296
C	-5.366178	1.147271	-0.703613
H	1.907382	1.799144	1.592914
H	3.776952	3.154728	2.433134
H	6.111565	2.516922	1.980103
H	6.632874	0.460426	0.704588
H	5.529049	-2.778615	-1.222224
H	6.320705	-2.222794	0.259088
H	6.424561	-1.259585	-1.227036
H	2.105375	-2.658166	1.662133
H	1.070172	-4.066230	1.290563
H	0.431724	-2.699548	2.204196
C	0.423586	1.468783	-0.605642
H	-0.742450	-3.916144	-0.264566
H	-1.496212	-2.541314	0.544246
H	-1.124225	-2.430236	-1.175261
H	-6.433646	1.026428	-0.505926
H	-5.063058	2.160909	-0.441008
H	-5.174233	0.989009	-1.764977
H	-6.199997	-1.659409	-0.028225
H	-4.908749	-1.852316	-1.240100

H	-4.657198	-2.433663	0.413996
H	-6.096439	0.118909	1.996079
H	-4.562088	-0.531383	2.625317
H	-4.719557	1.204374	2.308493
H	3.270843	-3.039425	-2.086788
H	3.285492	-3.726361	-0.460545
H	1.131329	-2.630622	-1.915043
C	1.249921	1.668467	-1.712548
C	1.619889	2.941774	-2.108111
C	1.181513	4.049652	-1.400828
C	0.346803	3.871058	-0.308277
C	-0.042421	2.599384	0.069488
H	1.602705	0.807739	-2.270765
H	2.258245	3.069637	-2.975074
H	1.477052	5.046228	-1.706729
H	-0.012559	4.729873	0.247111
H	-0.706902	2.466887	0.918404

PFNaz2

SCF Energy: -1539.15441568

ZPE-corrected Energy: -1538.67368

ΔU: -1538.64296

ΔH: -1538.64201

ΔG: -1538.73687

Num. Imaginary Frequencies: 0

C	1.998663	3.586148	-1.177945
C	0.818348	3.085527	-0.643865
C	0.300159	1.830378	-0.991406
C	0.966499	1.079586	-1.961834
C	2.143001	1.564849	-2.495373
C	2.661521	2.801007	-2.098216
N	-0.054548	3.671631	0.247981
C	-1.121602	2.853820	0.444887
C	-0.942254	1.665693	-0.266913
C	-1.696150	0.445357	-0.235899
C	-1.091019	-0.817297	-0.255394
Au	0.911314	-1.221271	0.179409
P	3.205828	-1.414809	0.583523
C	3.778555	-2.407724	1.984835
C	0.164936	4.956920	0.874283
C	-2.164871	3.267917	1.418283
O	-1.731259	2.905104	2.712327
C	-3.211403	0.332808	-0.239072
C	-3.772596	0.203496	1.182750

C	-3.937331	1.393187	-1.046329
C	3.914759	0.228163	0.872136
C	4.156724	-2.042241	-0.825084
H	0.560378	0.136596	-2.306504
H	2.667011	0.985433	-3.246521
H	3.584729	3.159200	-2.537635
H	2.378944	4.562050	-0.902100
H	-0.302972	5.762054	0.303987
H	1.235139	5.144693	0.931600
H	-0.229133	4.953304	1.888410
H	-3.481374	1.517232	-2.031207
H	-3.947694	2.366069	-0.556221
H	-4.979705	1.096983	-1.190984
C	-2.106764	-1.746781	-0.482615
H	-4.862743	0.136073	1.140227
H	-3.390835	-0.697201	1.668416
H	-3.508944	1.048123	1.816796
H	5.226987	-2.002667	-0.609653
H	3.868484	-3.071326	-1.039650
H	3.941747	-1.428910	-1.701707
H	4.999768	0.169060	0.983869
H	3.668677	0.873617	0.024891
H	3.479906	0.660471	1.773890
H	4.866774	-2.356726	2.063791
H	3.331544	-2.032996	2.905794
H	3.475521	-3.445814	1.847080
H	-3.120074	2.803017	1.171653
H	-2.320223	4.350710	1.337239
H	-2.359504	3.248071	3.355403
C	-2.087432	-3.133596	-0.245693
C	-3.268355	-3.807542	-0.183934
C	-4.534127	-3.165242	-0.388138
C	-4.597310	-1.865185	-0.716379
C	-3.361823	-1.085184	-0.903950
H	-1.146857	-3.624018	-0.020763
H	-3.264775	-4.863146	0.063733
H	-5.436790	-3.756668	-0.298256
H	-5.550145	-1.388544	-0.923396
H	-3.293934	-0.888839	-1.987601

^{1}H and ^{13}C NMR Spectra

^{1}H NMR (CDCl$_3$, 300 MHz)

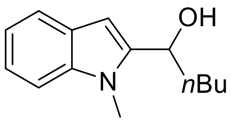

S3

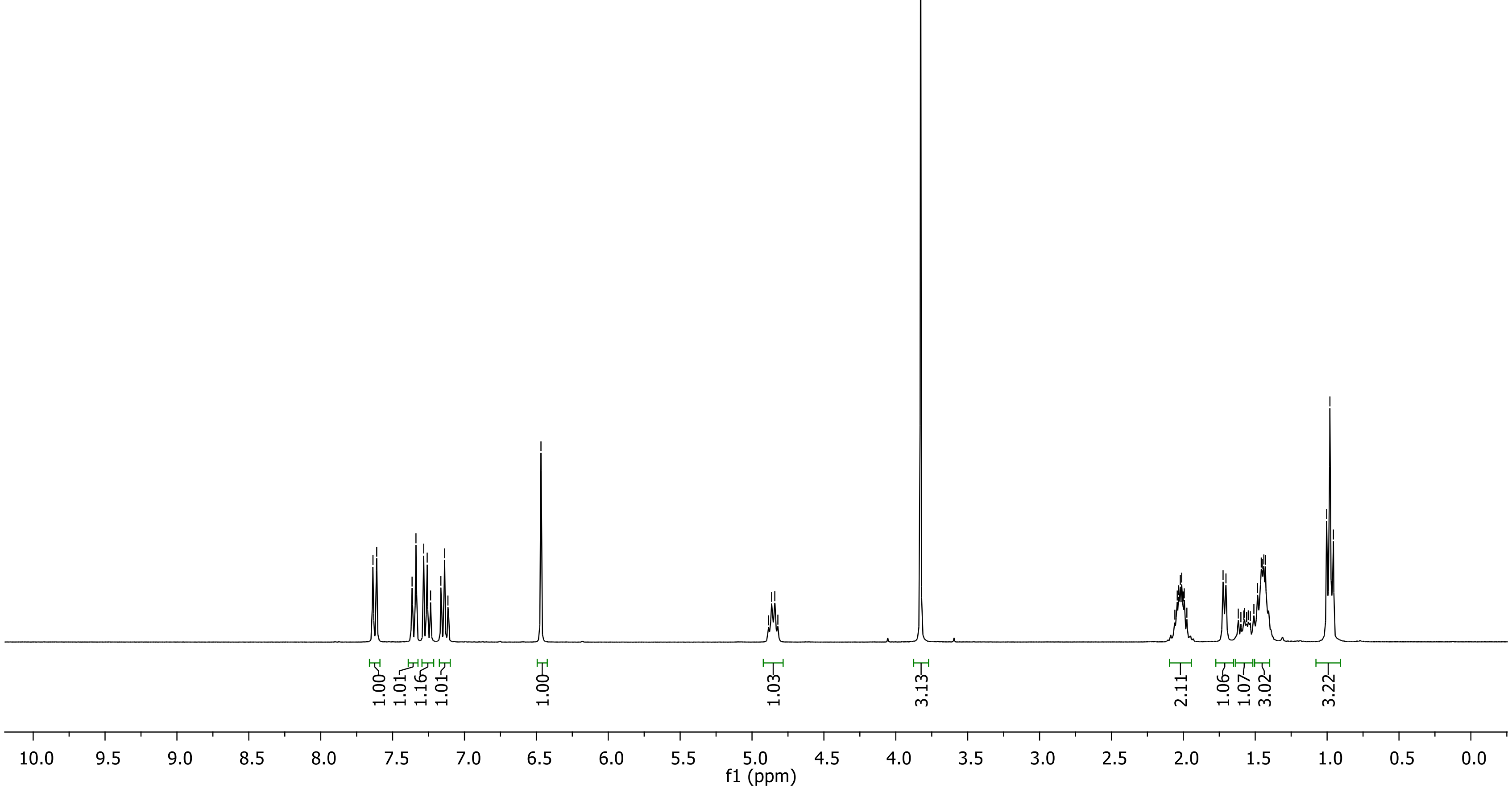

^{13}C NMR ($CDCl_3$, 75.4 MHz)

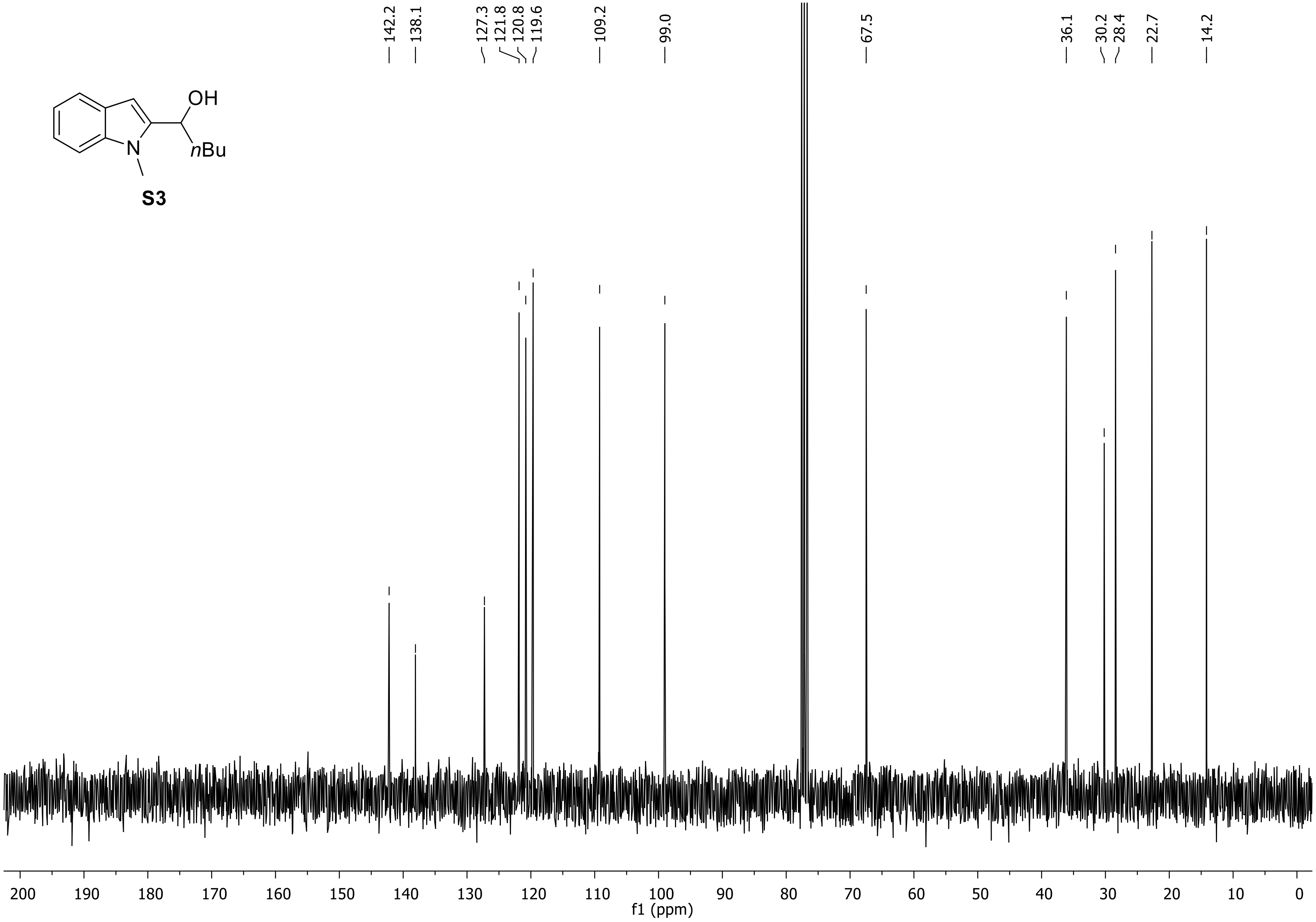

DEPT ($CDCl_3$, 75.4 MHz)

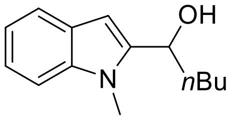

S3

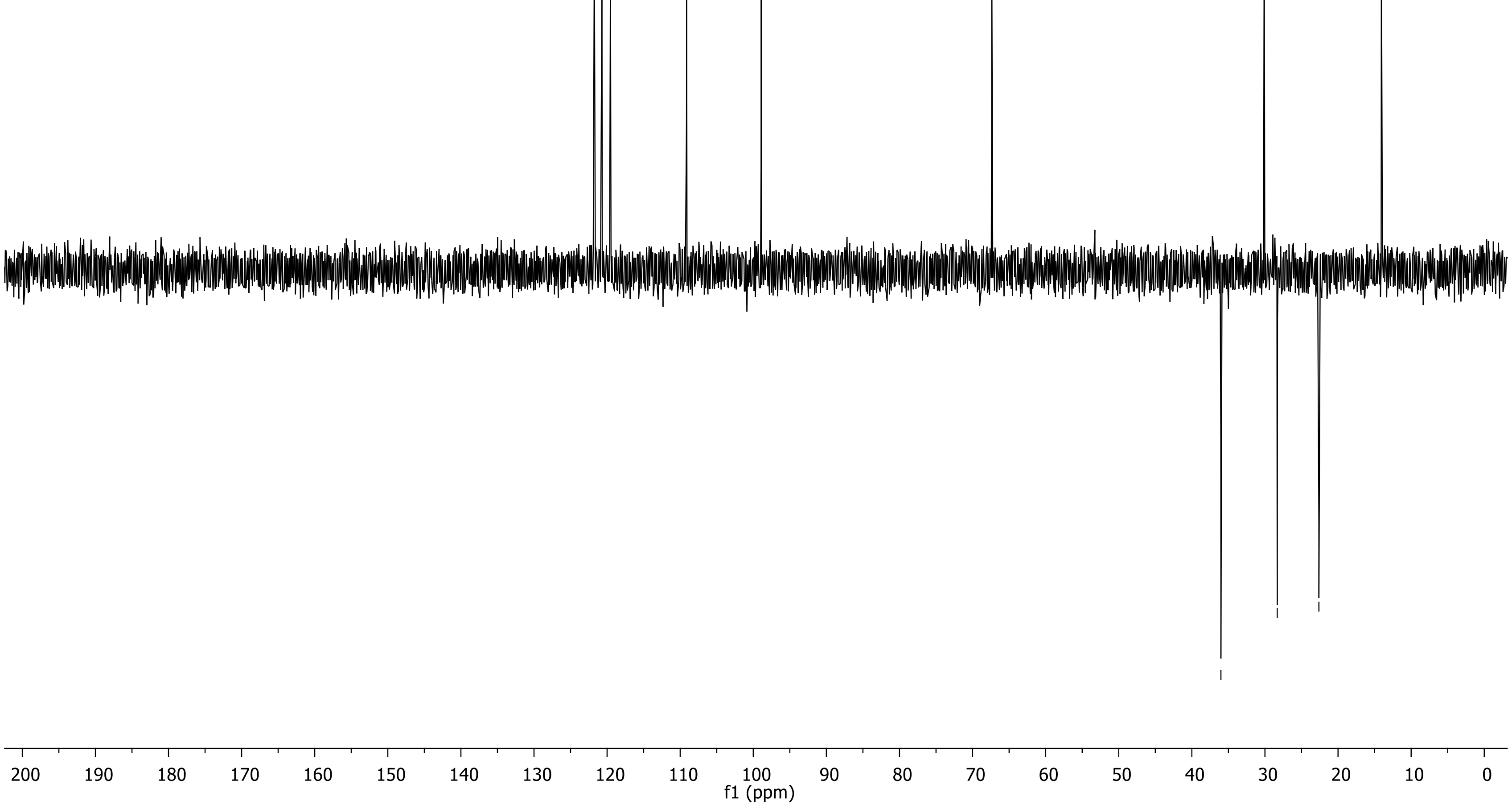

^{1}H NMR ($CDCl_3$, 300 MHz)

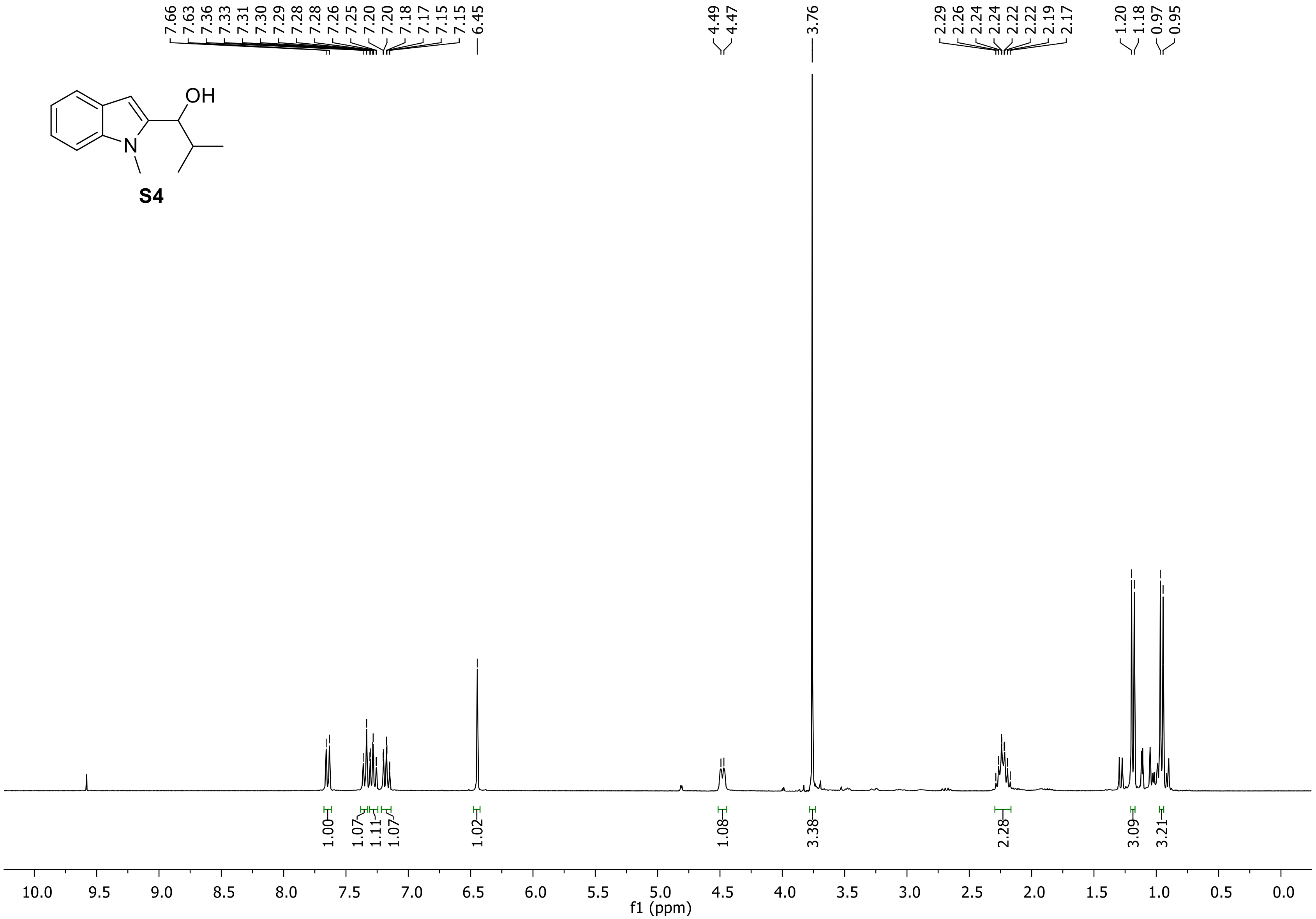

^{13}C NMR ($CDCl_3$, 75.4 MHz)

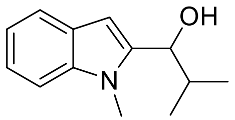

S4

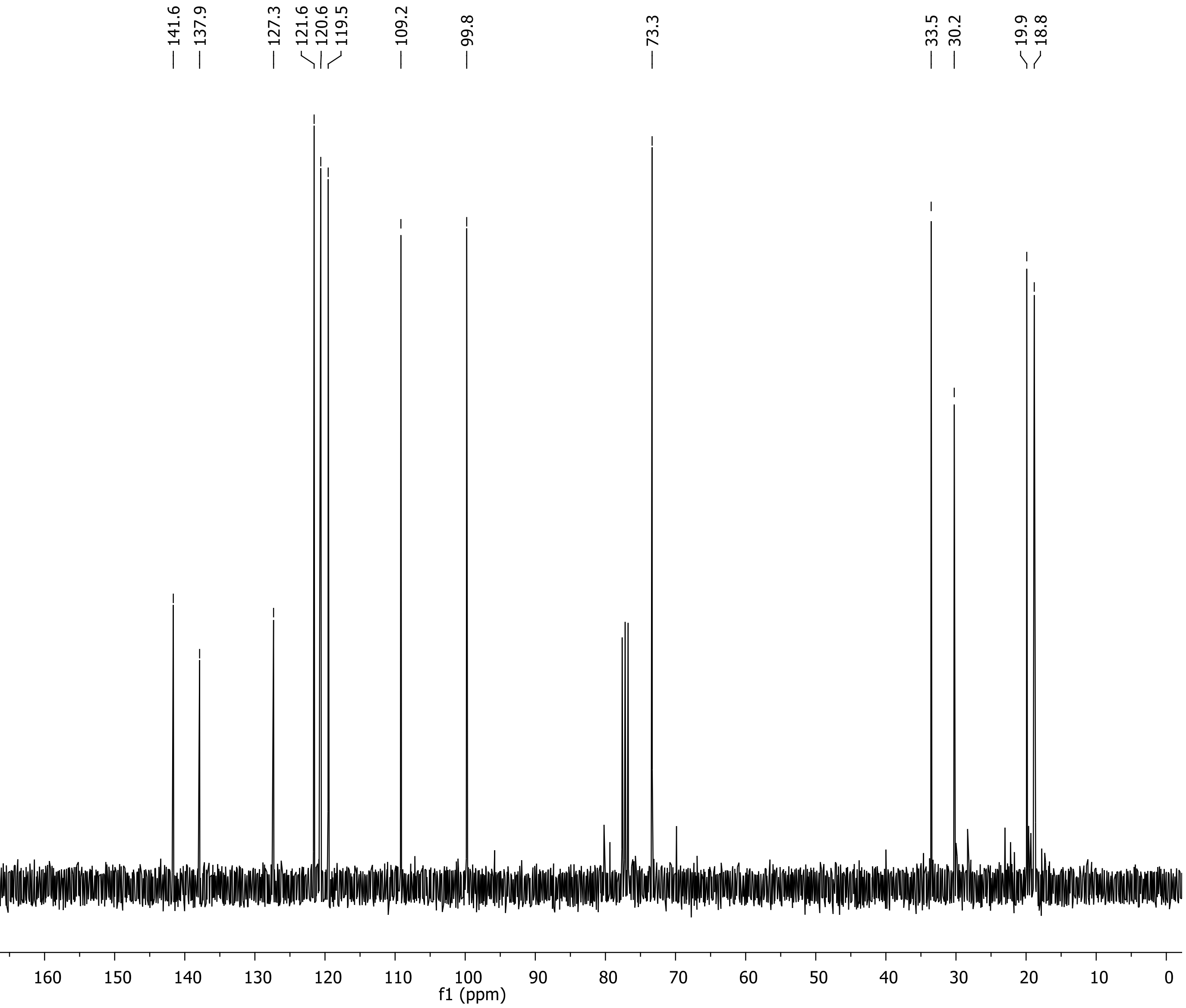

^{1}H NMR ($CDCl_3$, 300 MHz)

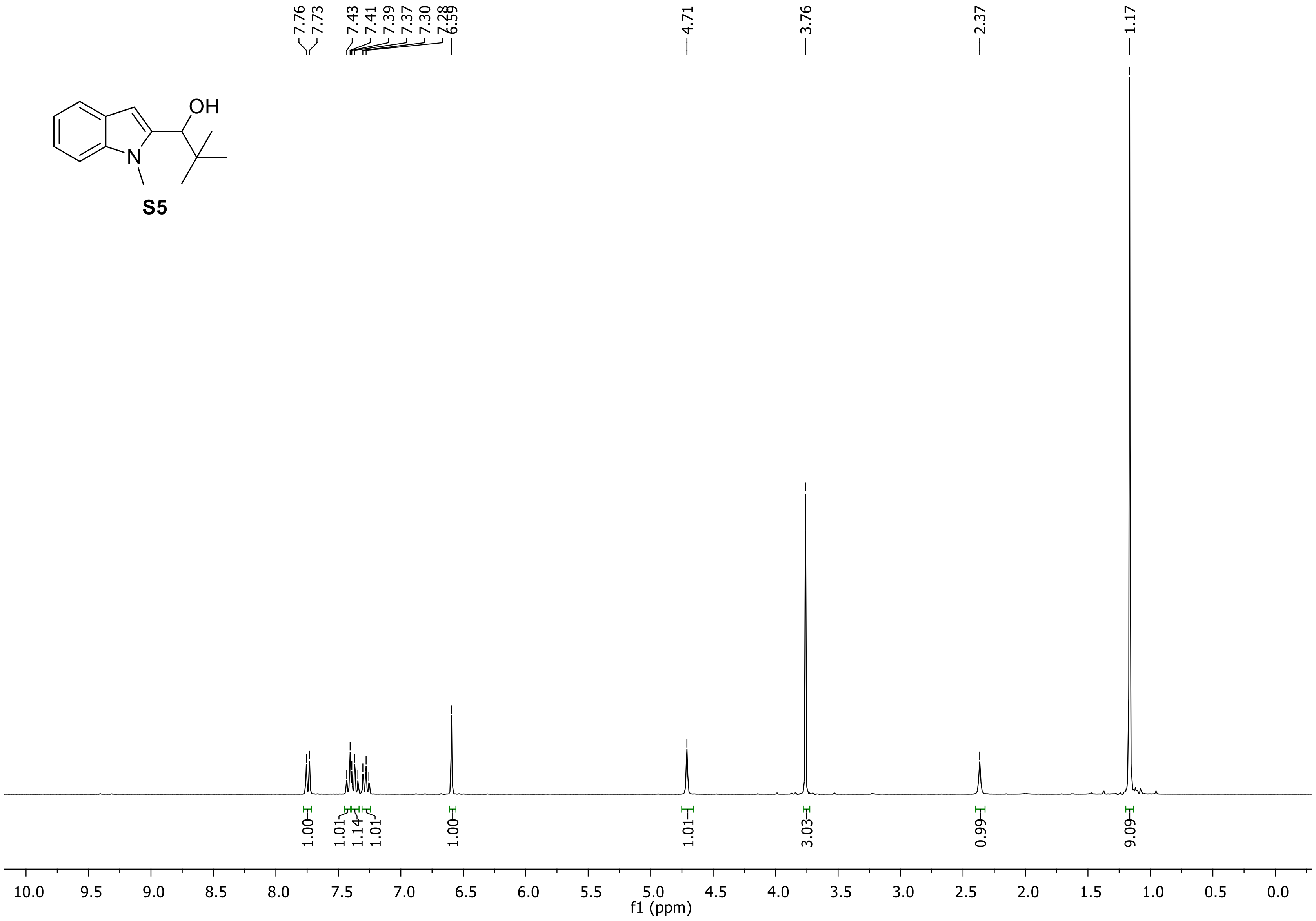

^{13}C NMR ($CDCl_3$, 75.4 MHz)

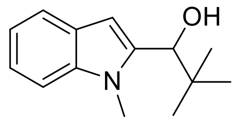

S5

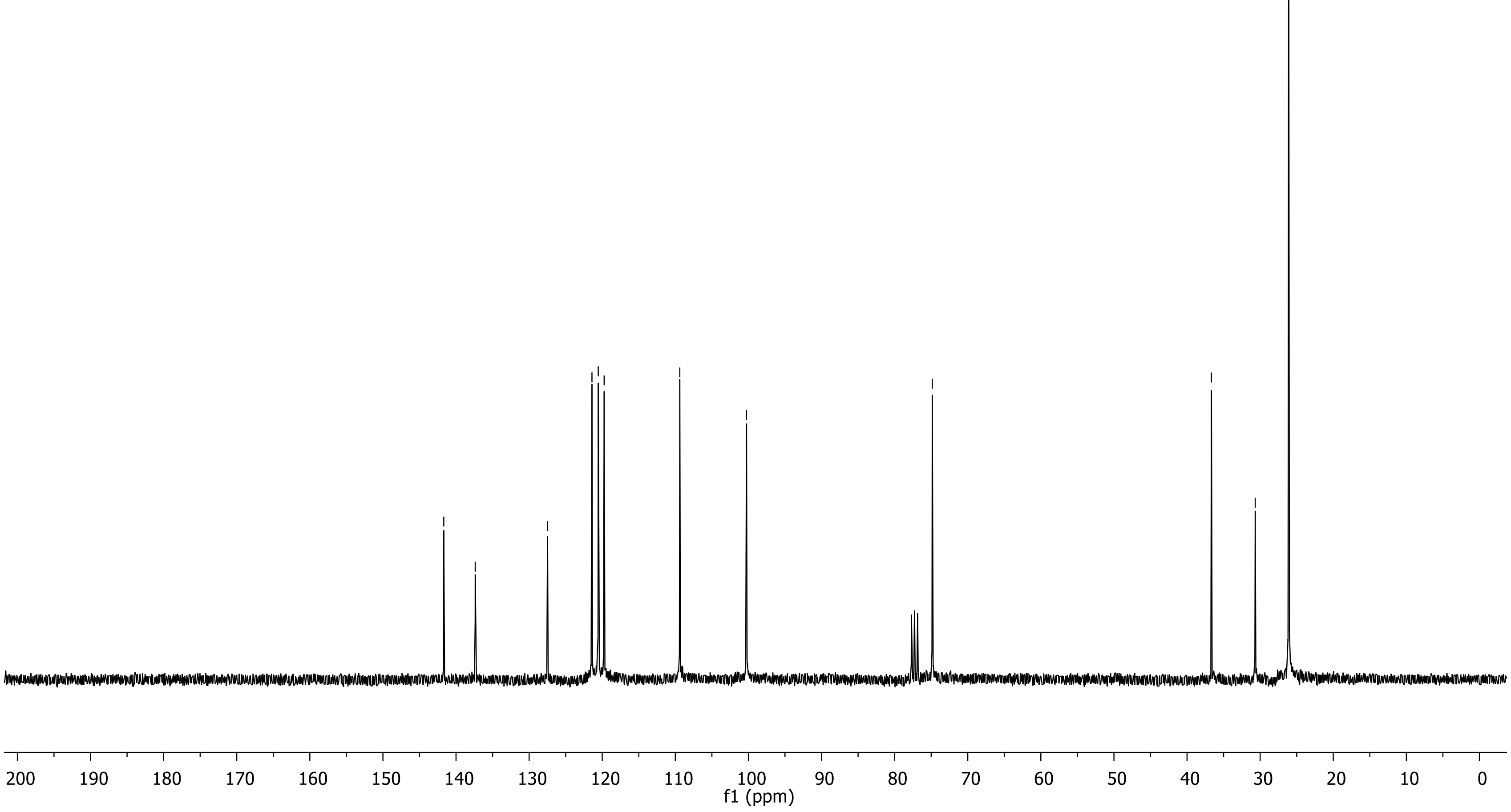

^{1}H NMR ($CDCl_3$, 300 MHz)

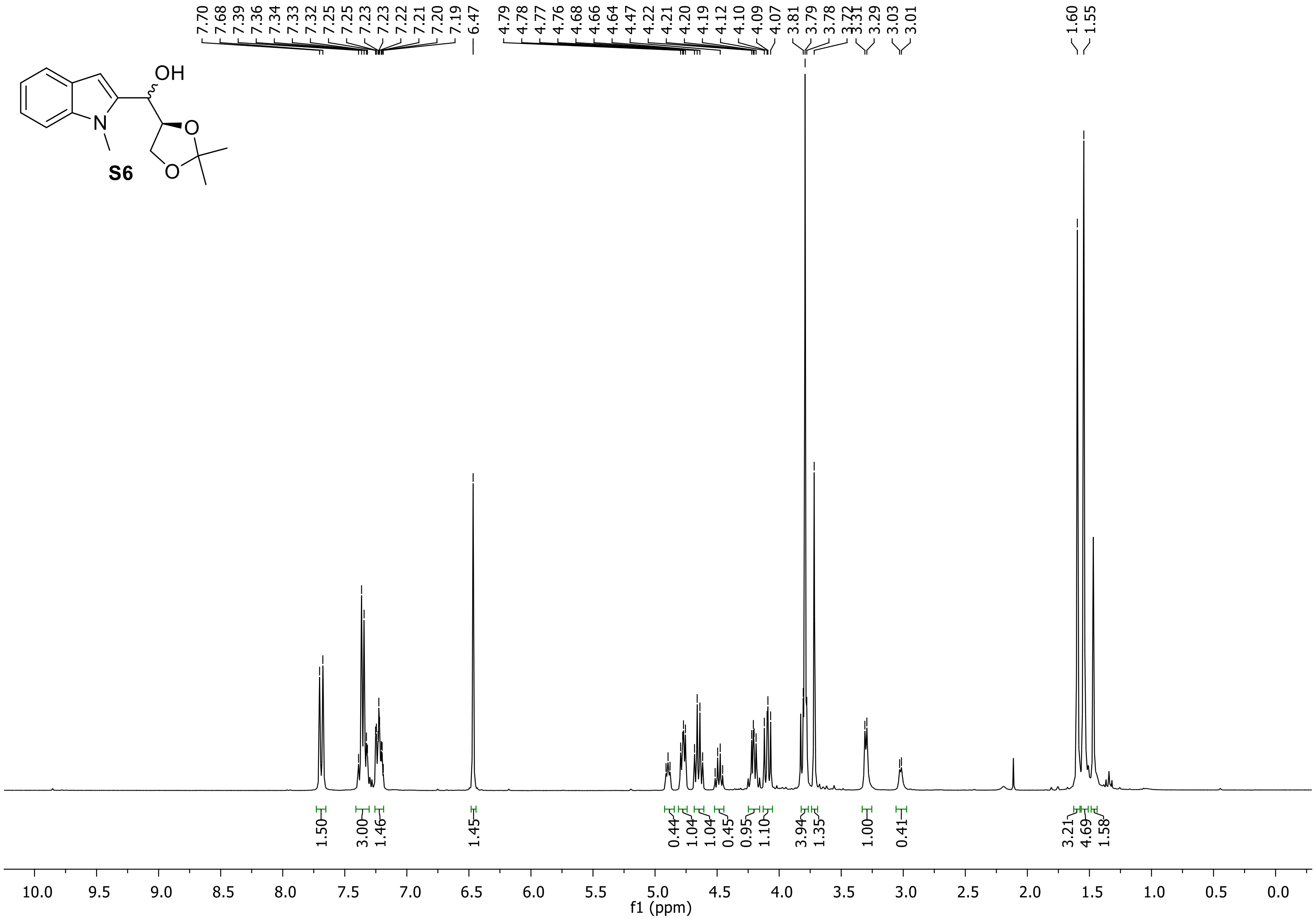

^{13}C NMR ($CDCl_3$, 75.4 MHz)

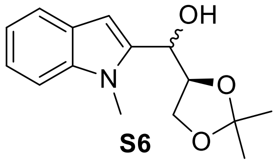

S6

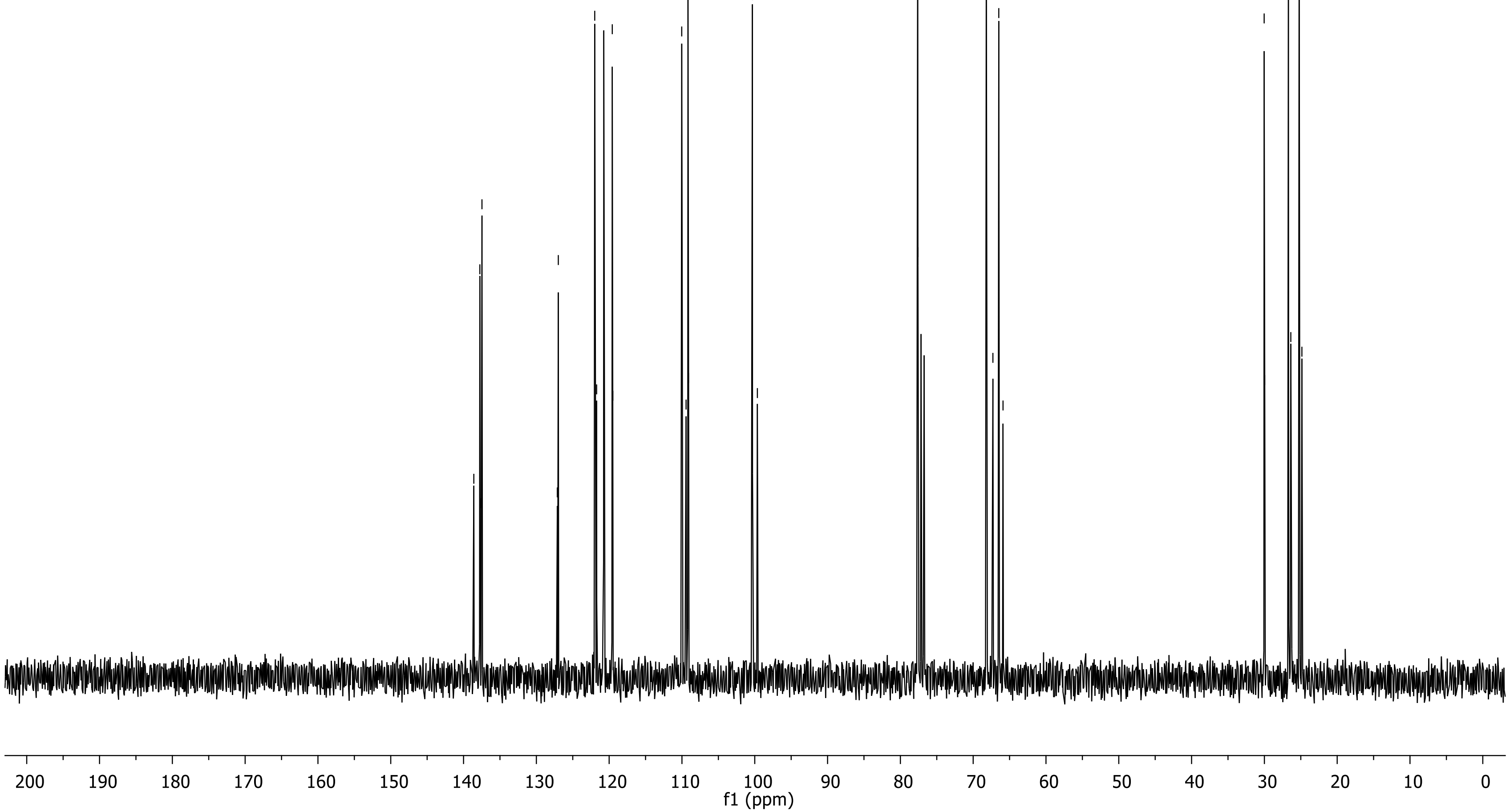

DEPT ($CDCl_3$, 75.4 MHz)

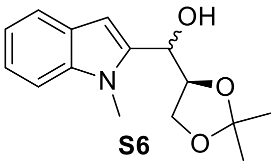

S6

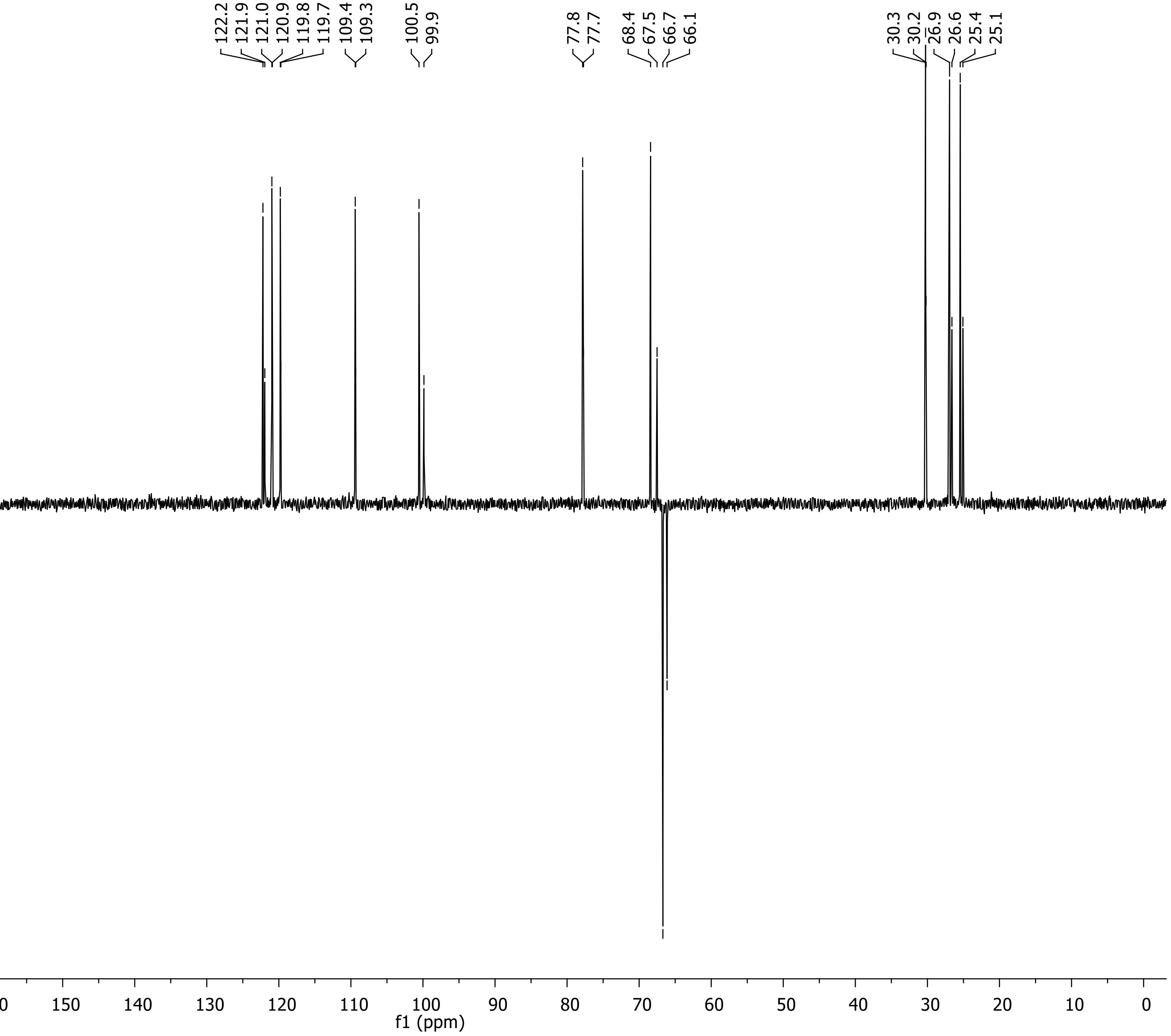

^{1}H NMR ($CDCl_3$, 300 MHz)

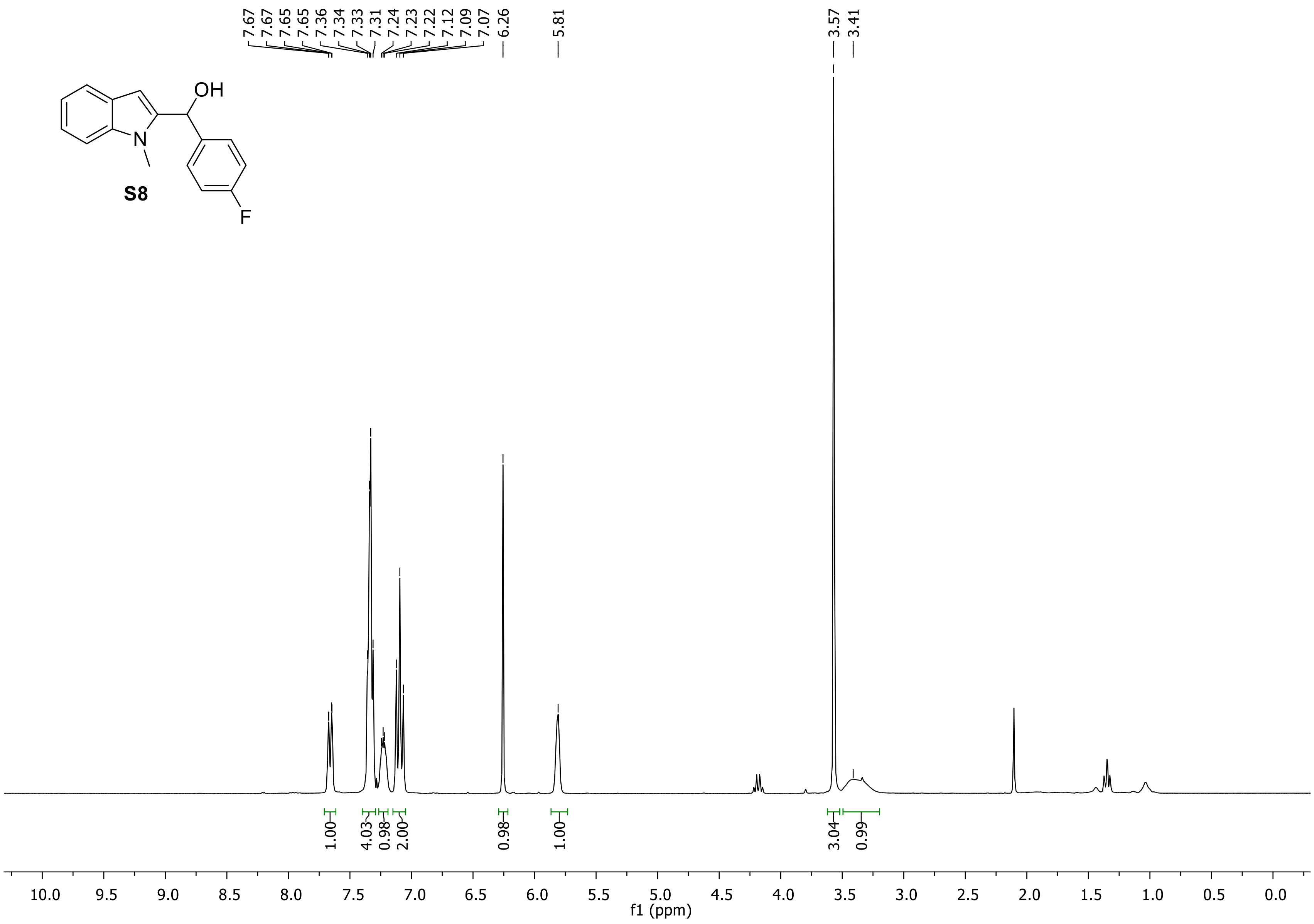

^{13}C NMR ($CDCl_3$, 75.4 MHz)

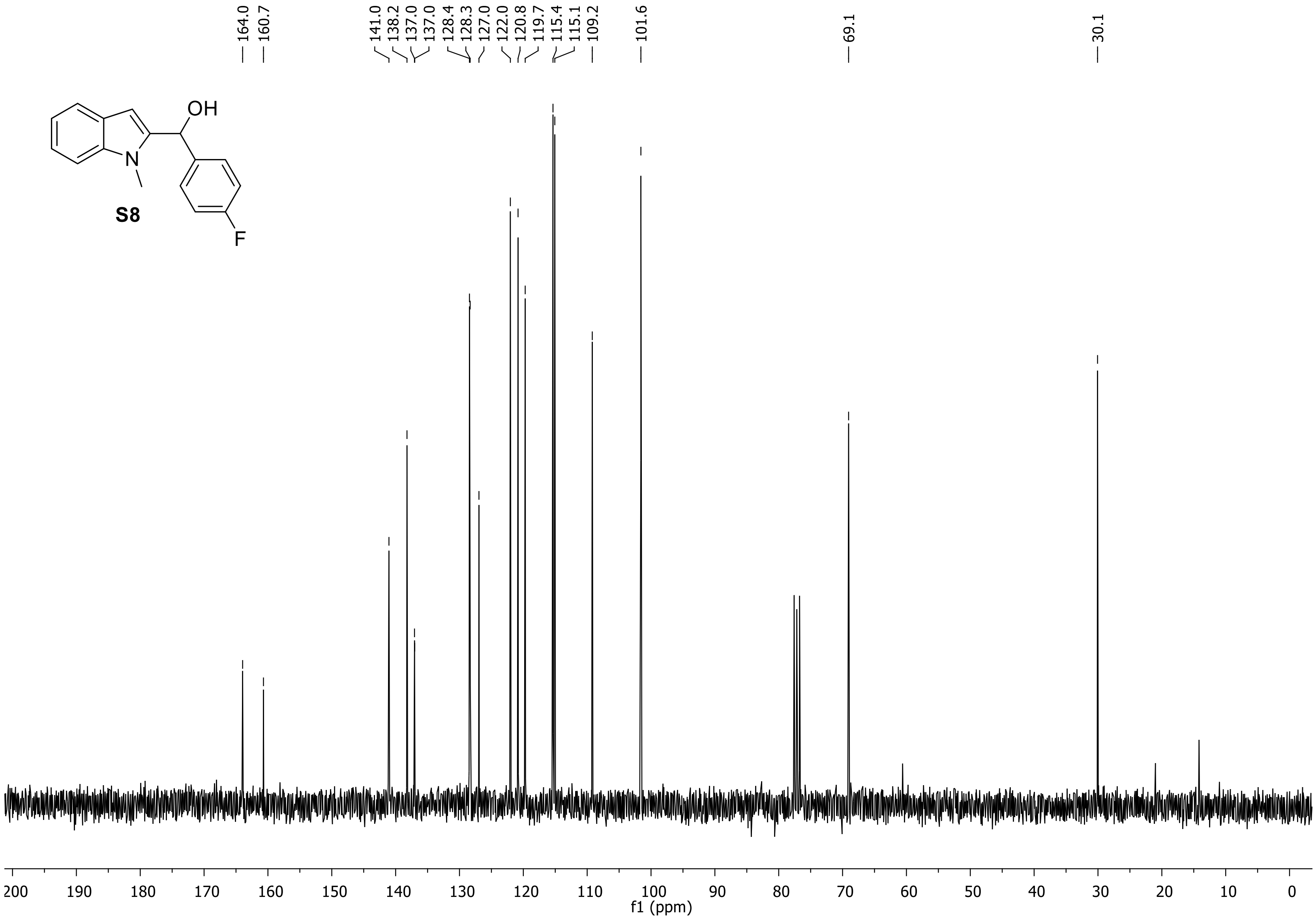

DEPT ($CDCl_3$, 75.4 MHz)

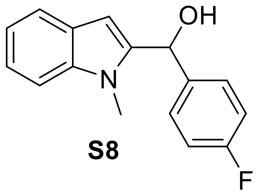

S8

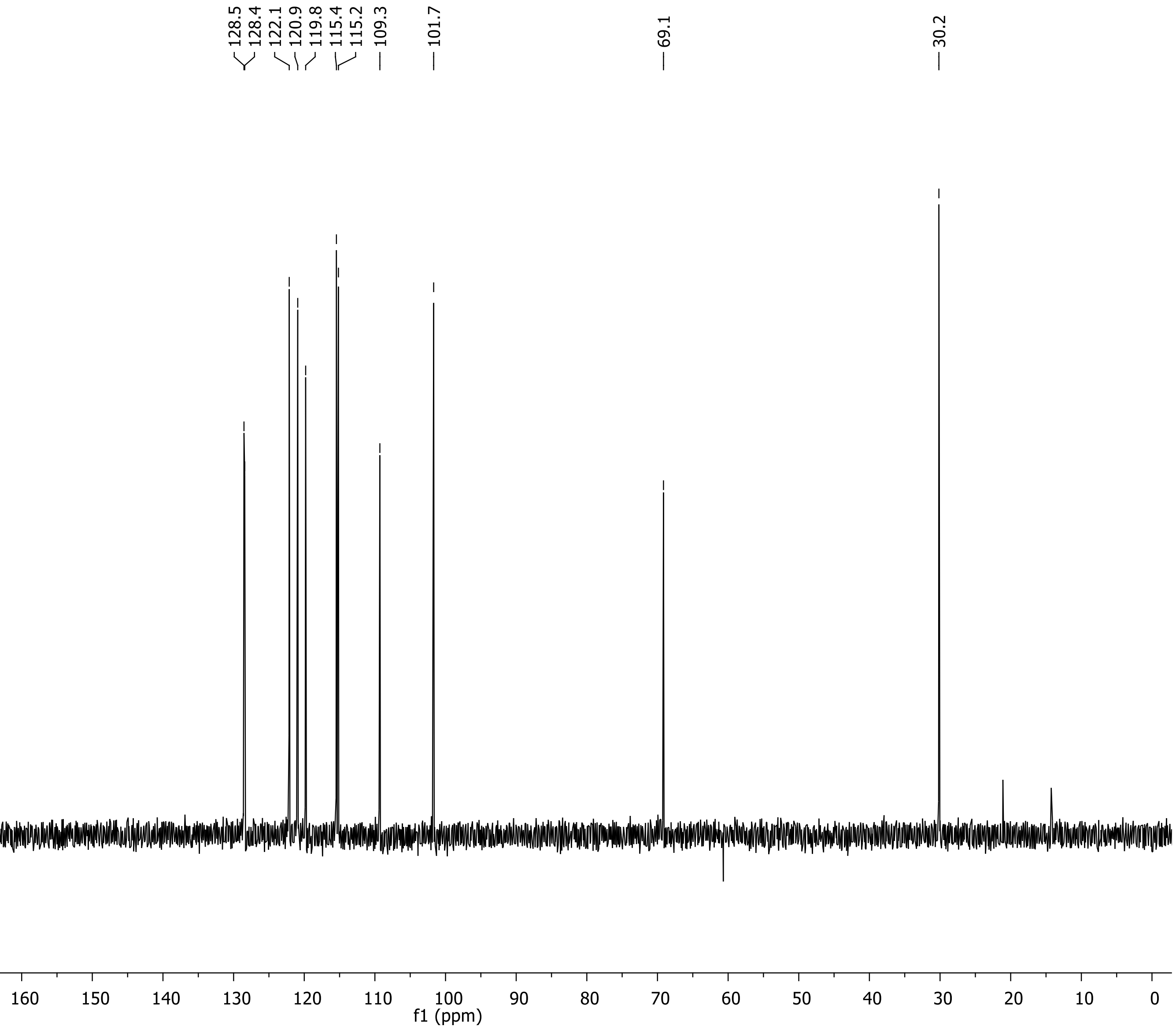

^{1}H NMR ($CDCl_3$, 300 MHz)

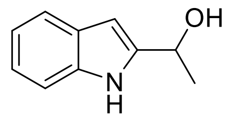

S10

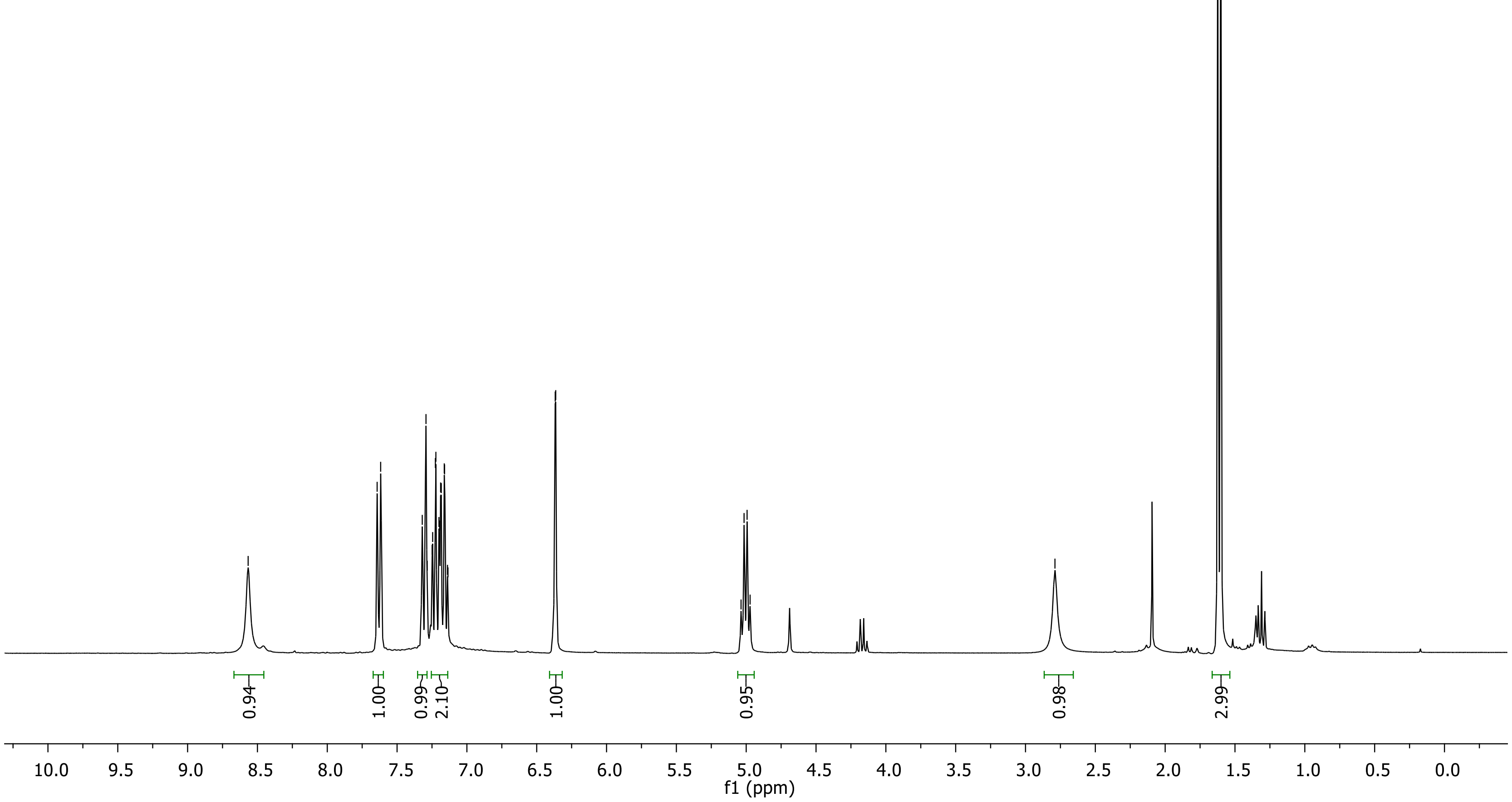

^{13}C NMR ($CDCl_3$, 75.4 MHz)

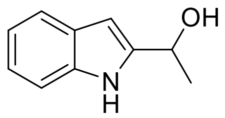

S10

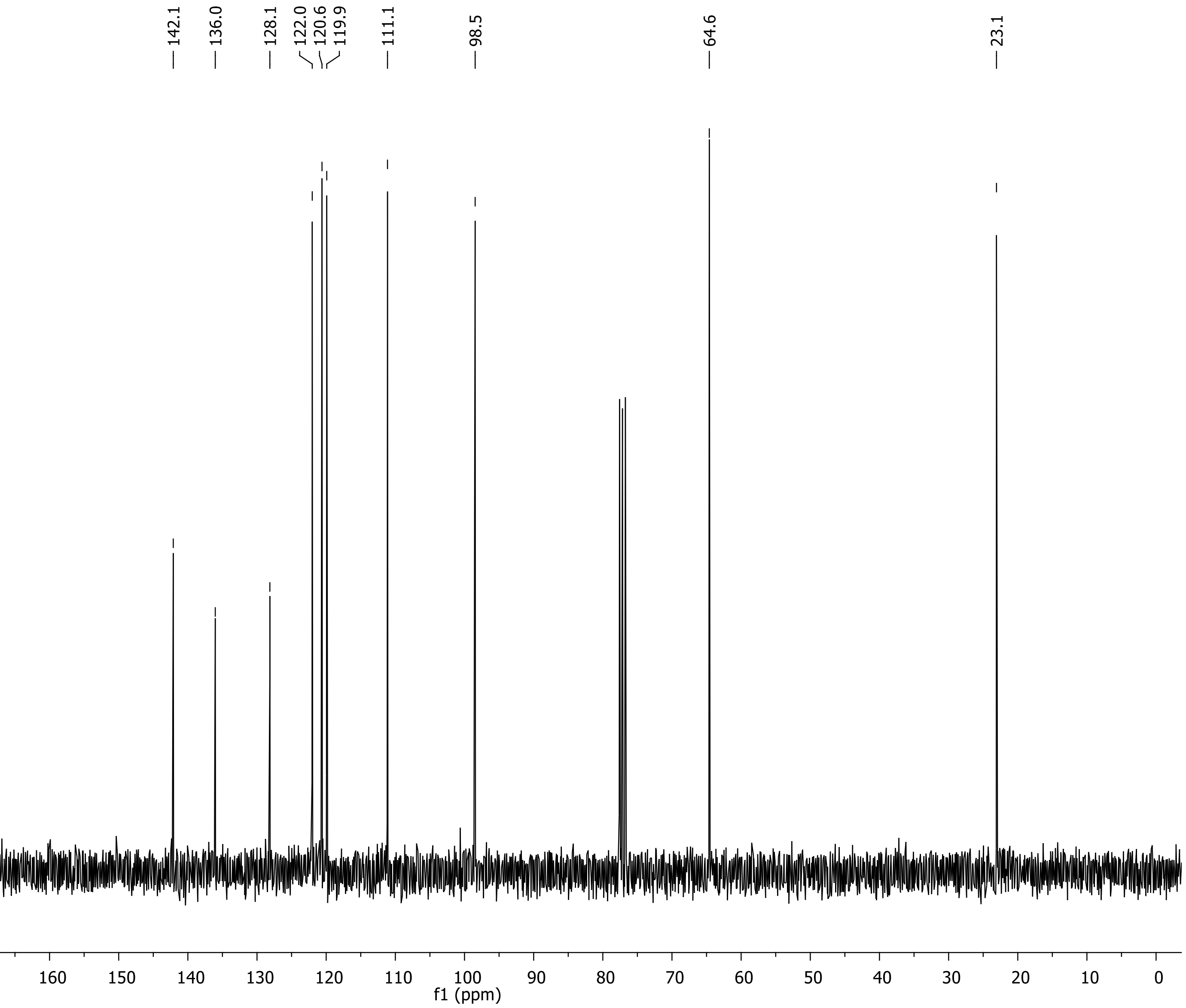

DEPT ($CDCl_3$, 75.4 MHz)

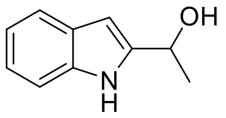

S10

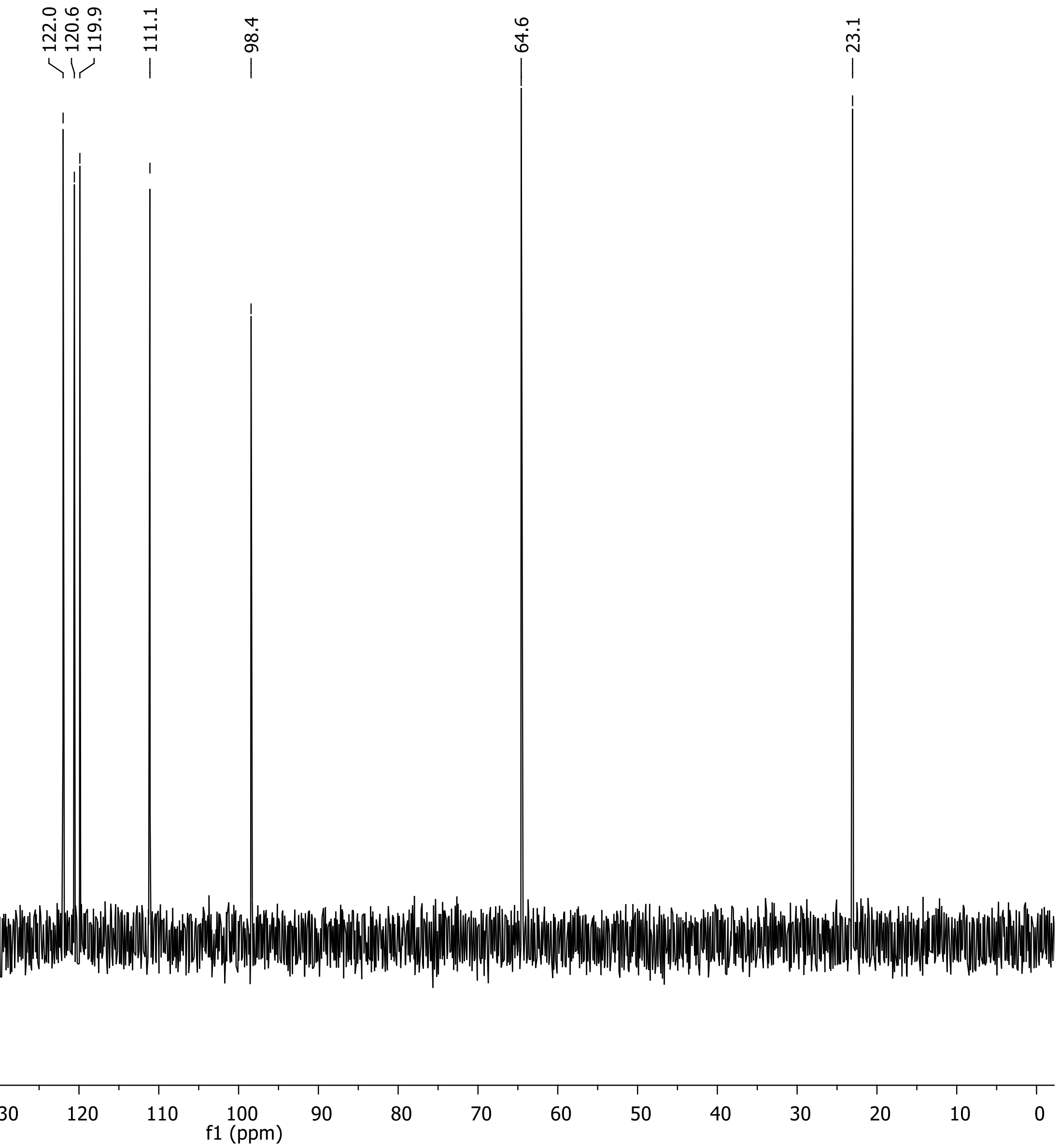

^{1}H NMR ($CDCl_3$, 300 MHz)

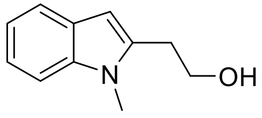

S11

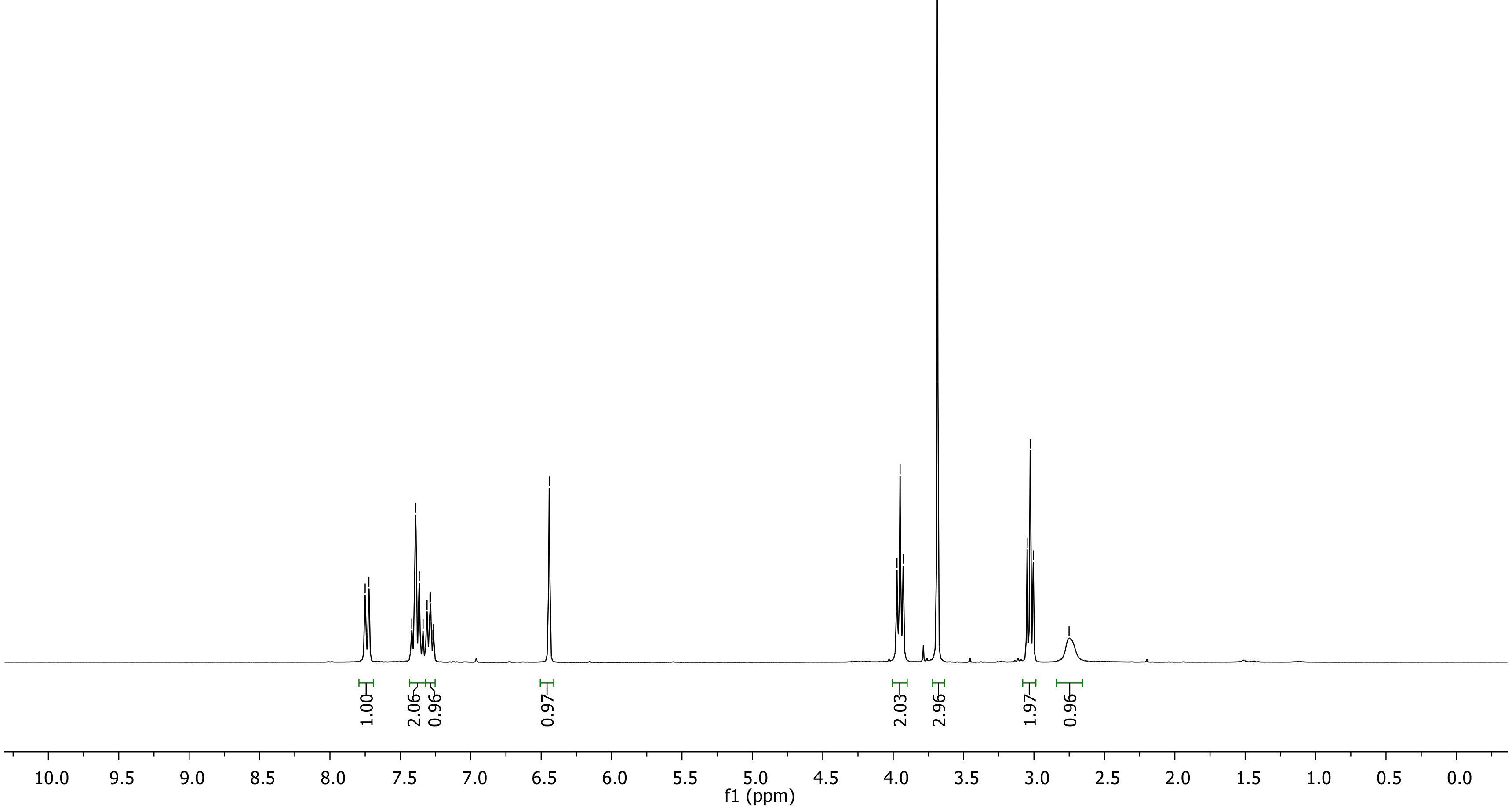

^{13}C NMR (CDCl$_3$, 75.4 MHz)

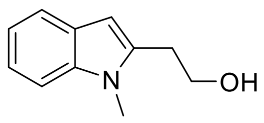

S11

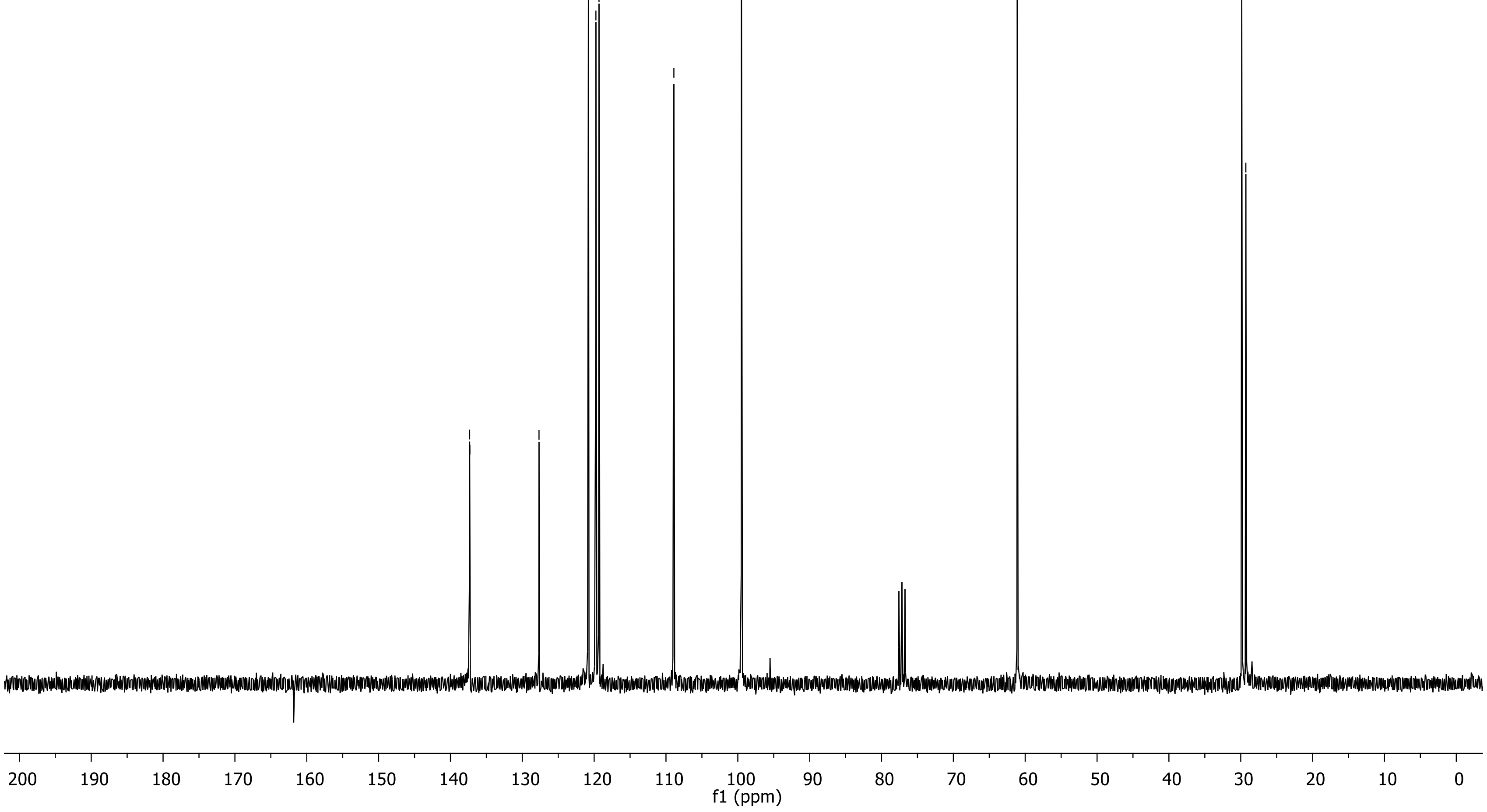

^{1}H NMR ($CDCl_3$, 300 MHz)

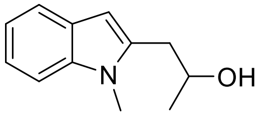

S12

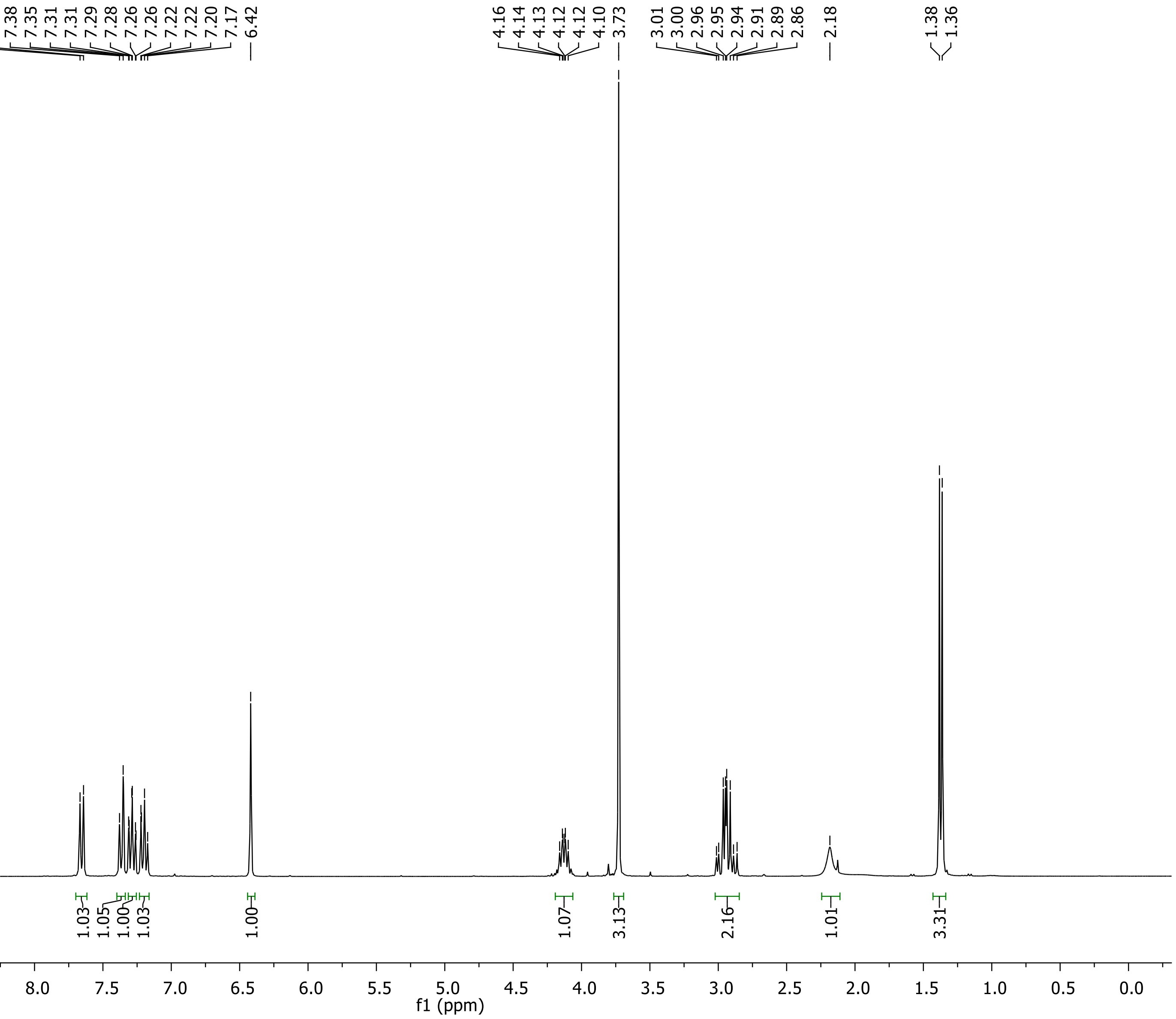

^{13}C NMR ($CDCl_3$, 75.4 MHz)

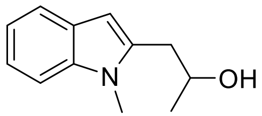

S12

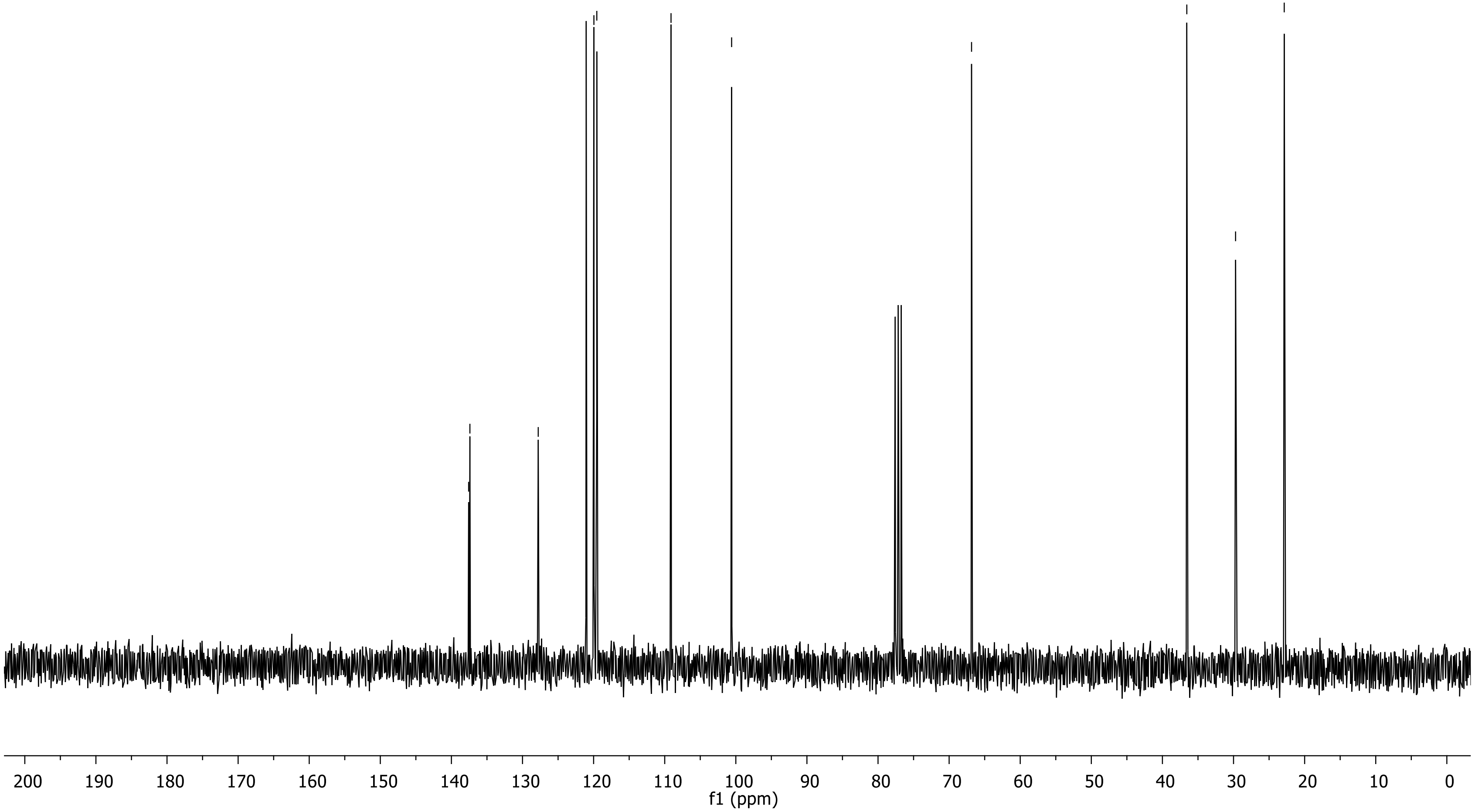

DEPT ($CDCl_3$, 75.4 MHz)

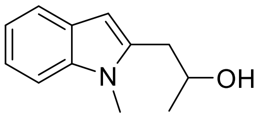

S12

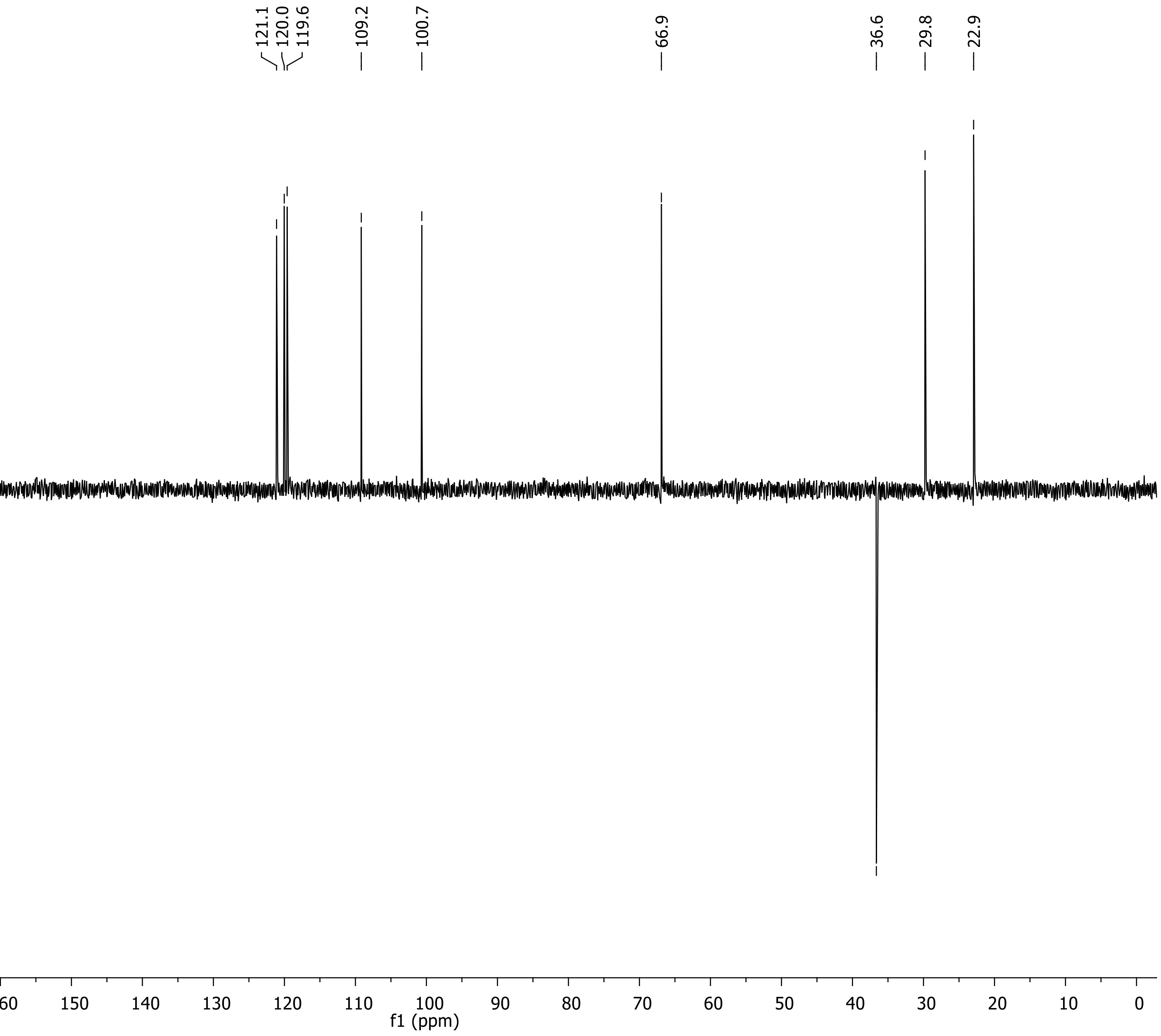

^{1}H NMR ($CDCl_3$, 300 MHz)

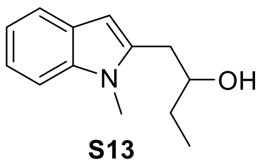

S13

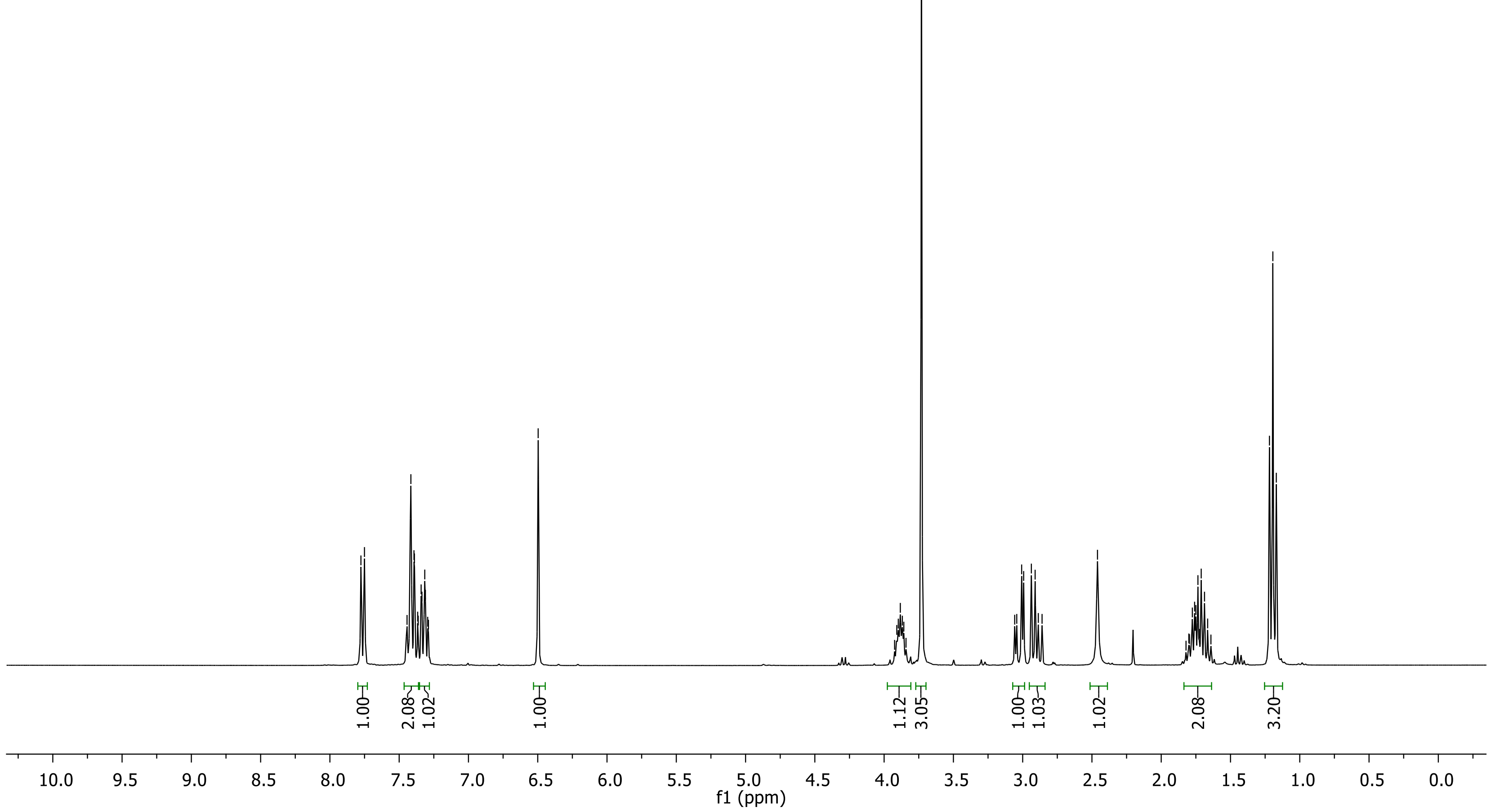

^{13}C NMR ($CDCl_3$, 75.4 MHz)

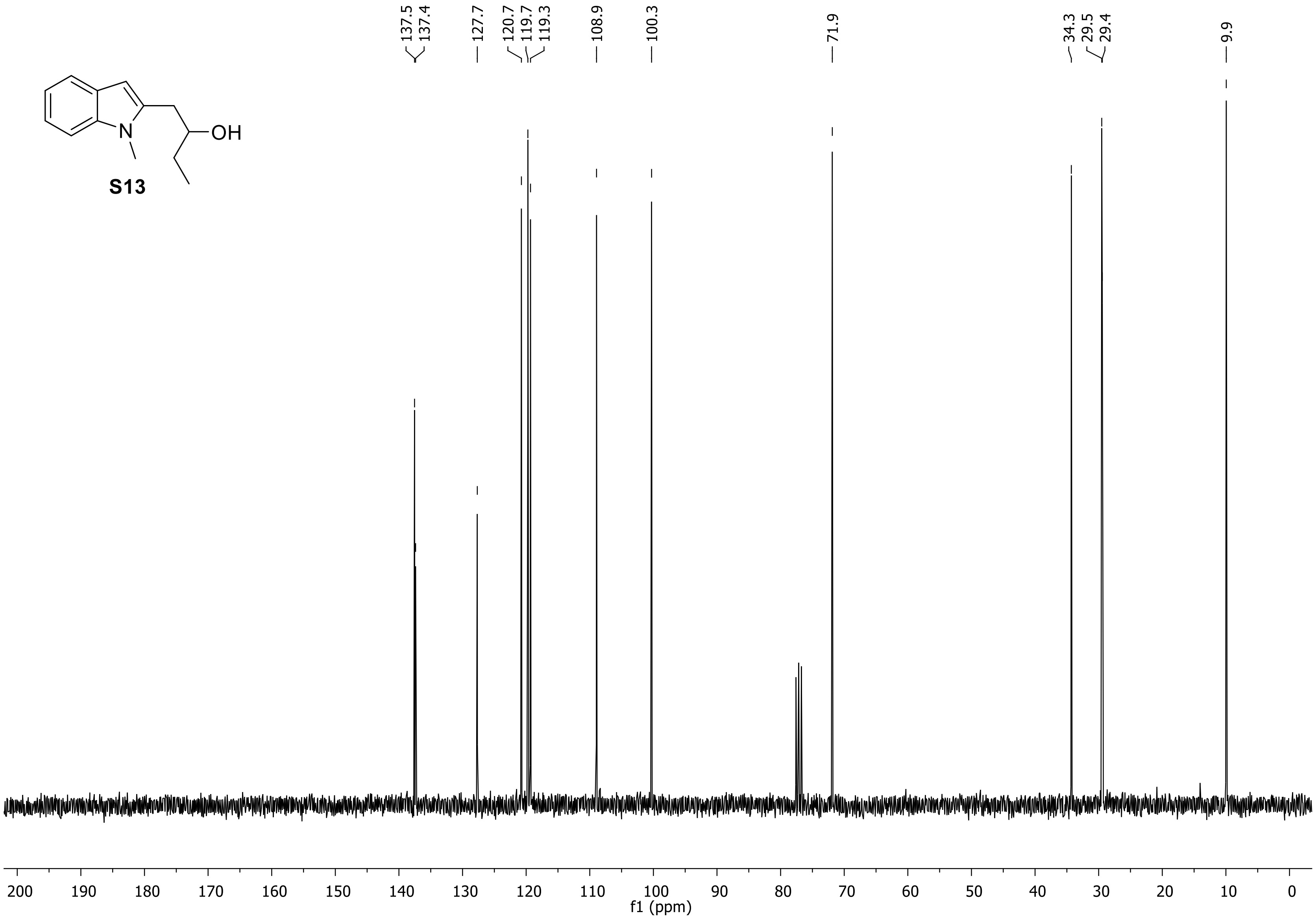

^{1}H NMR ($CDCl_3$, 300 MHz)

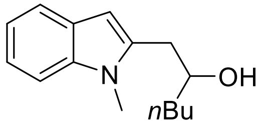

S14

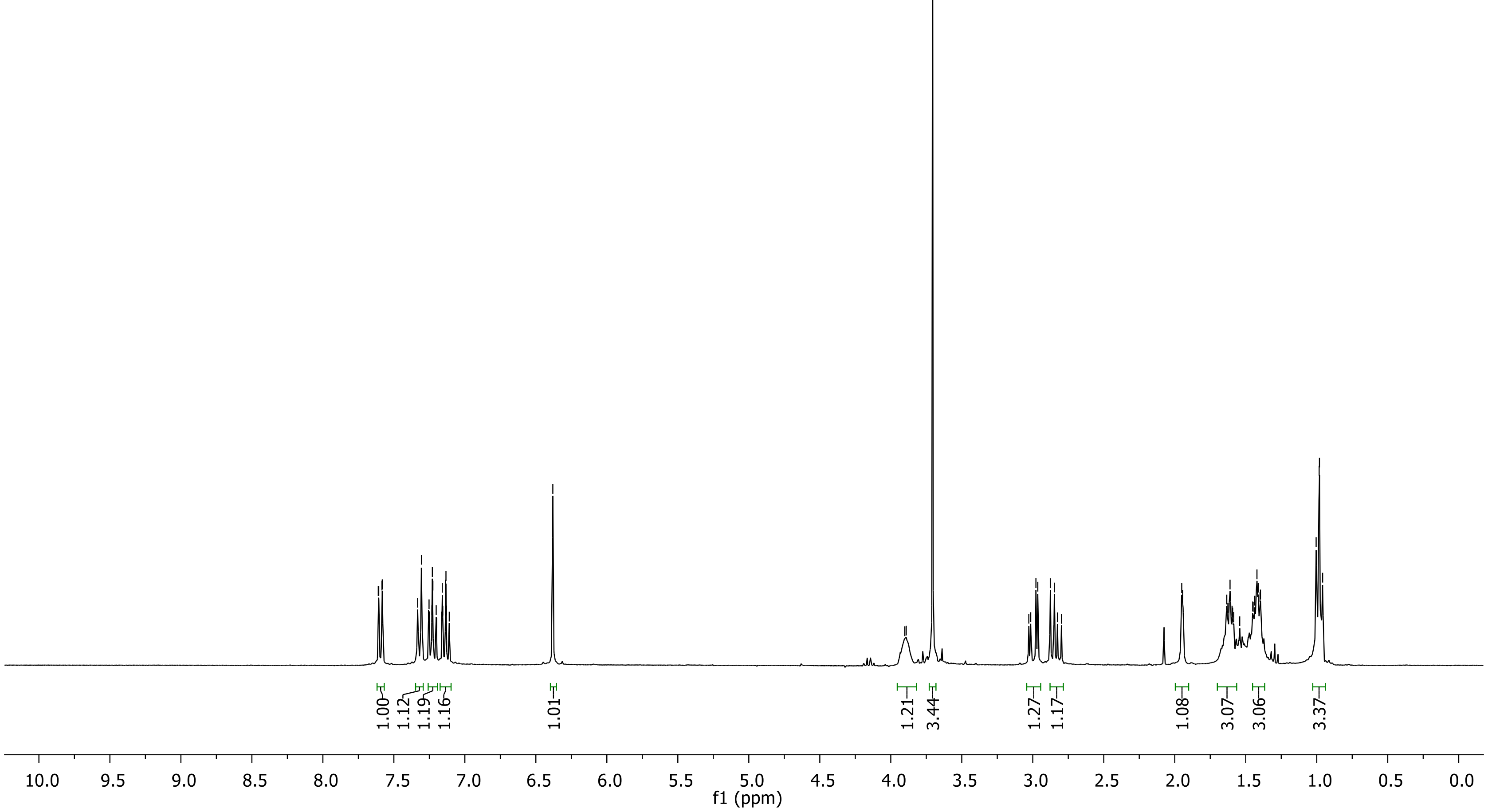

^{13}C NMR ($CDCl_3$, 75.4 MHz)

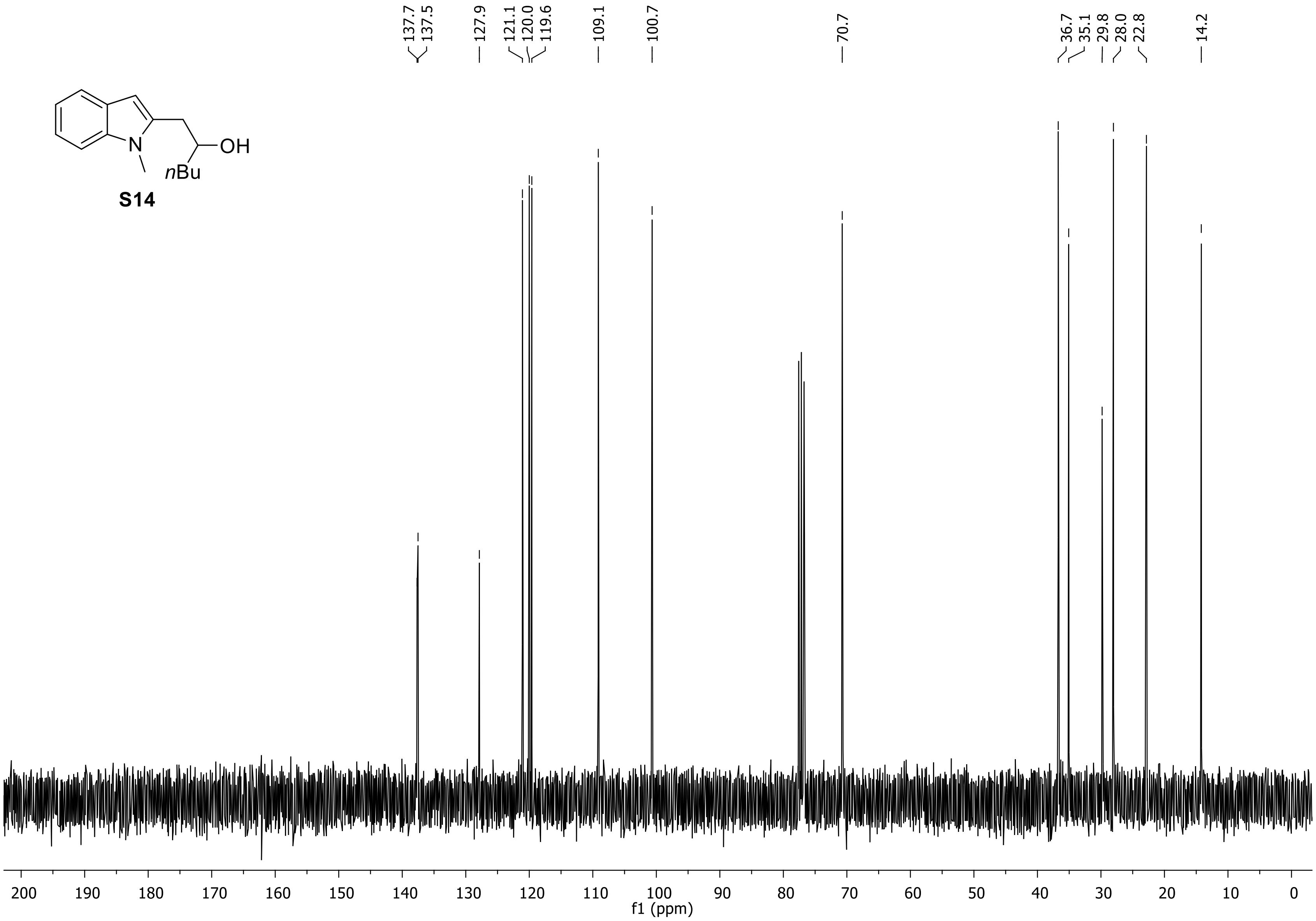

S14

^{1}H NMR ($CDCl_3$, 500 MHz)

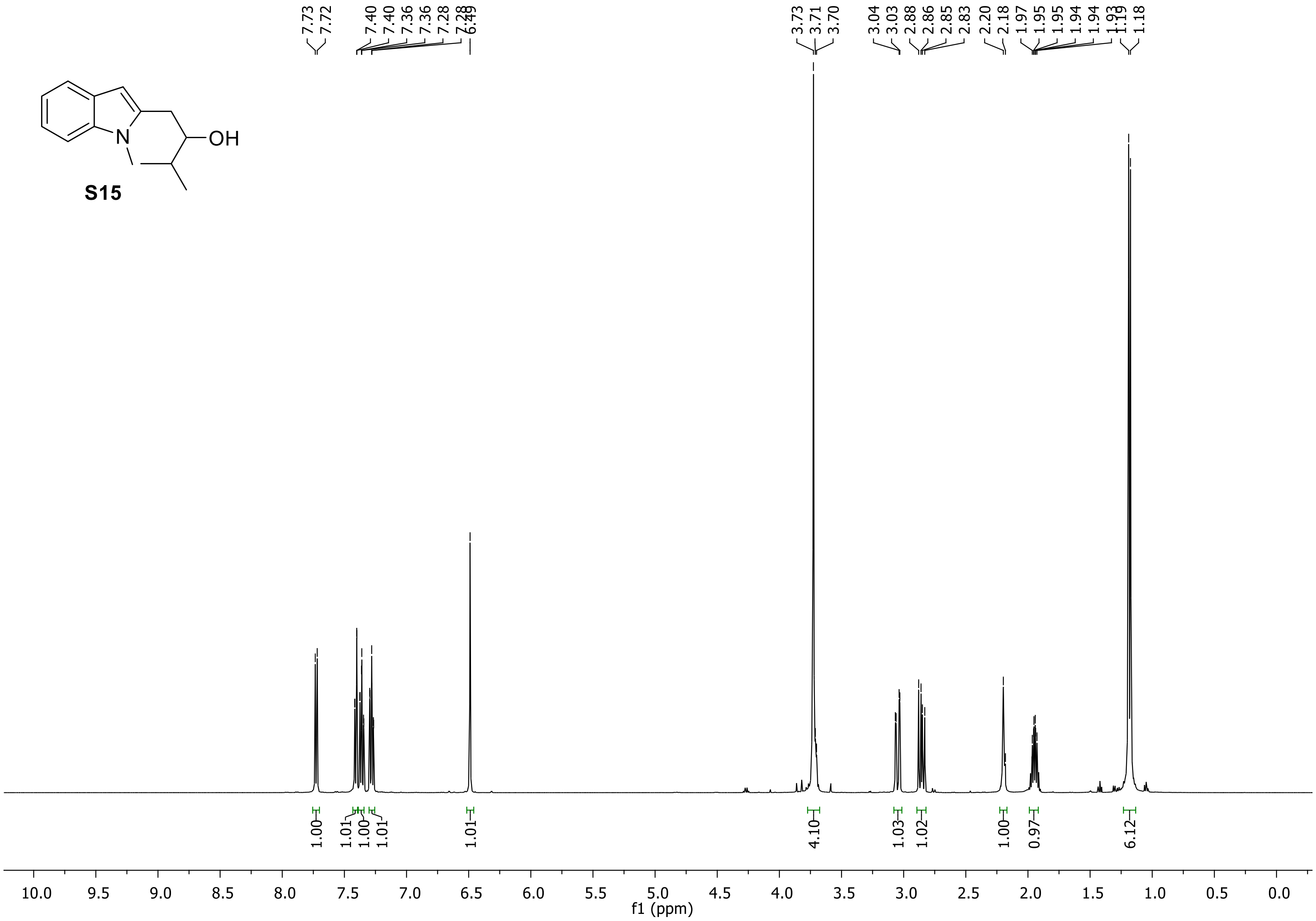

S15

^{13}C NMR ($CDCl_3$, 125.7 MHz)

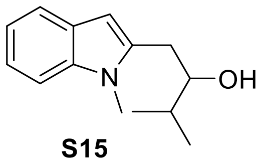

S15

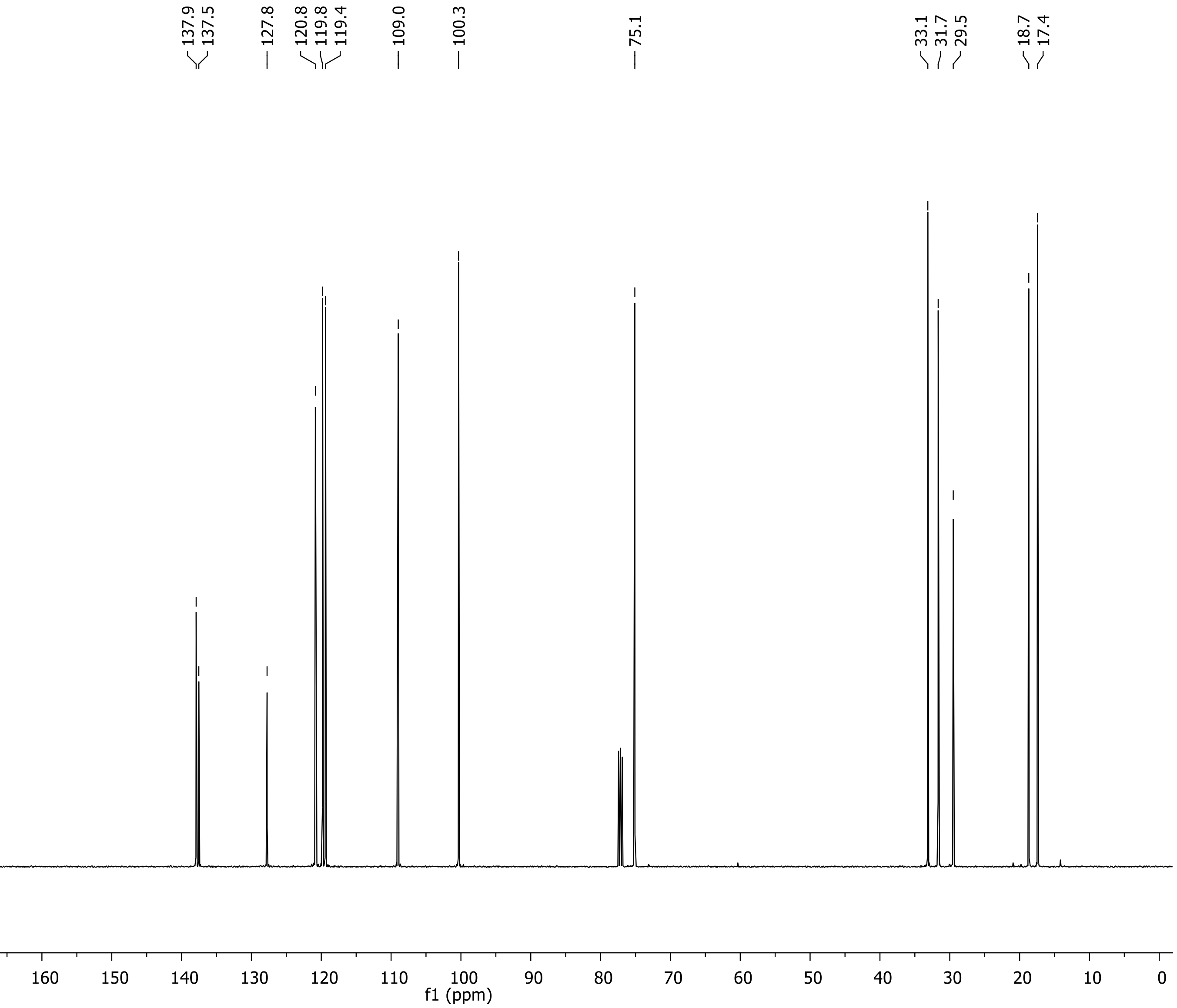

^1H NMR ($CDCl_3$, 300 MHz)

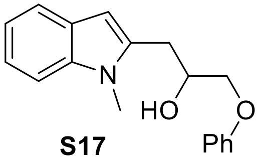

S17

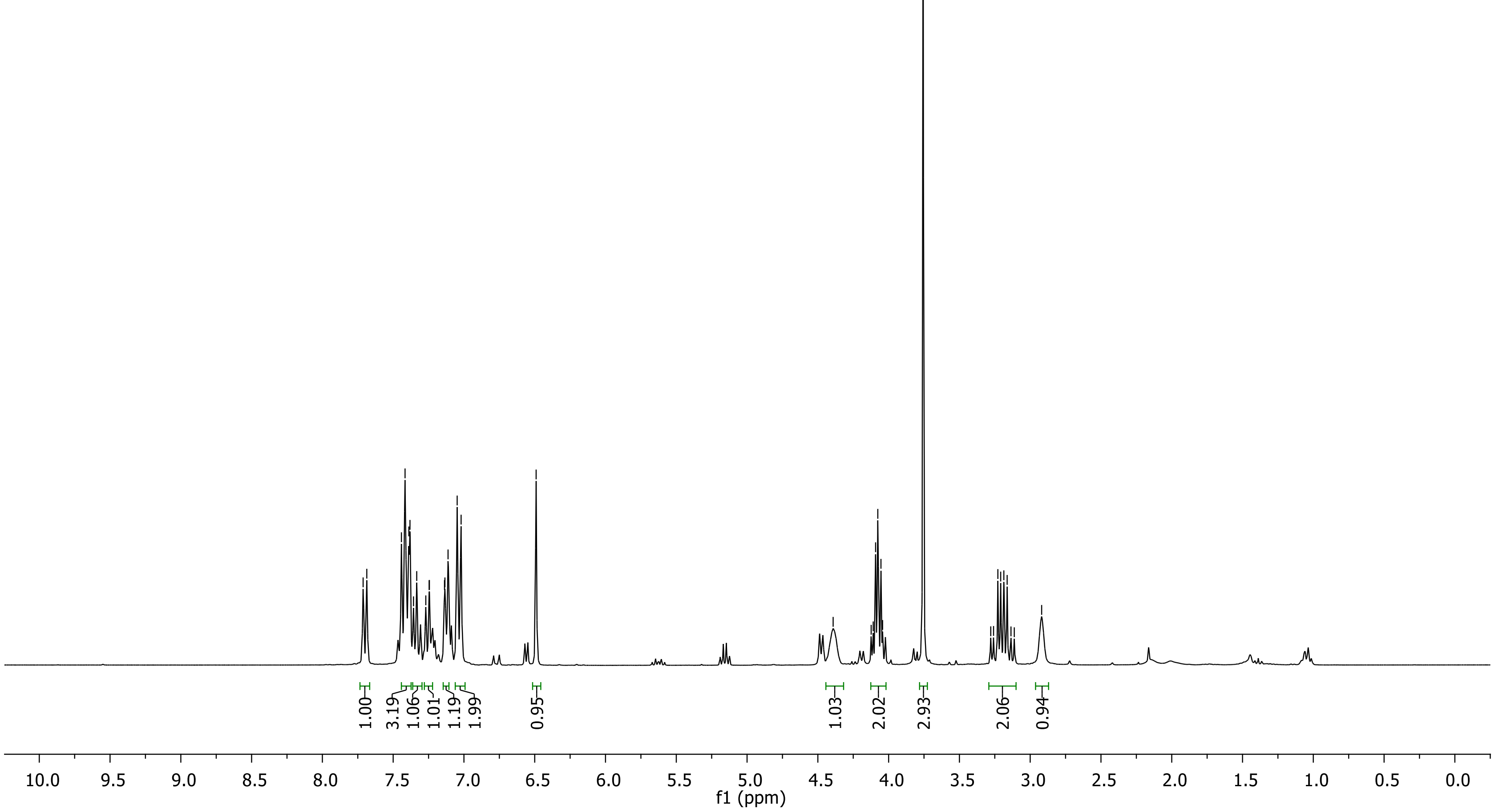

^{13}C NMR (CDCl$_3$, 75.4 MHz)

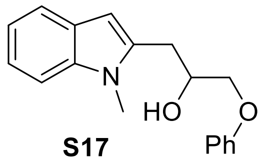

S17

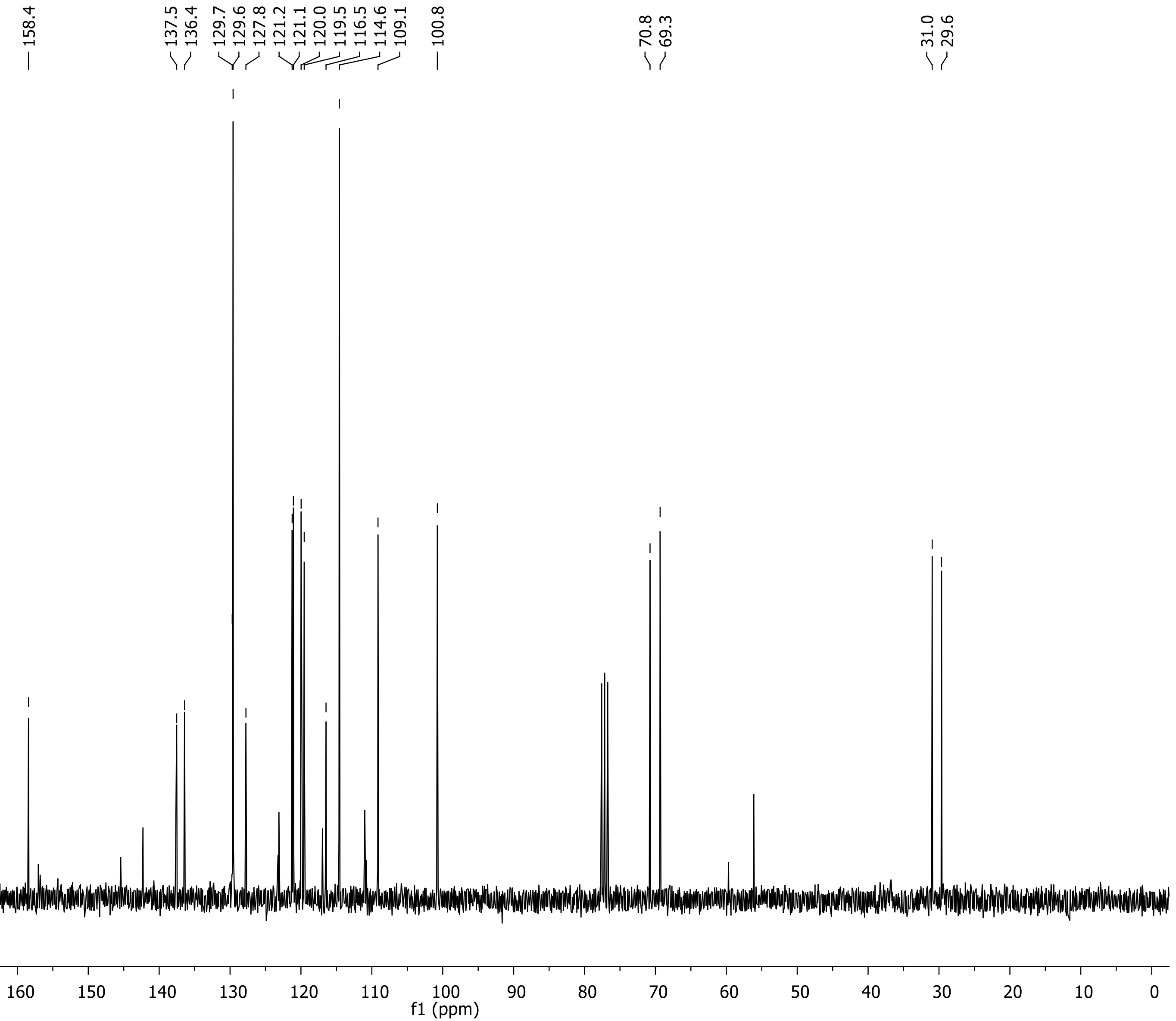

DEPT ($CDCl_3$, 75.4 MHz)

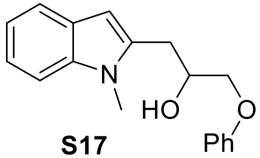

S17

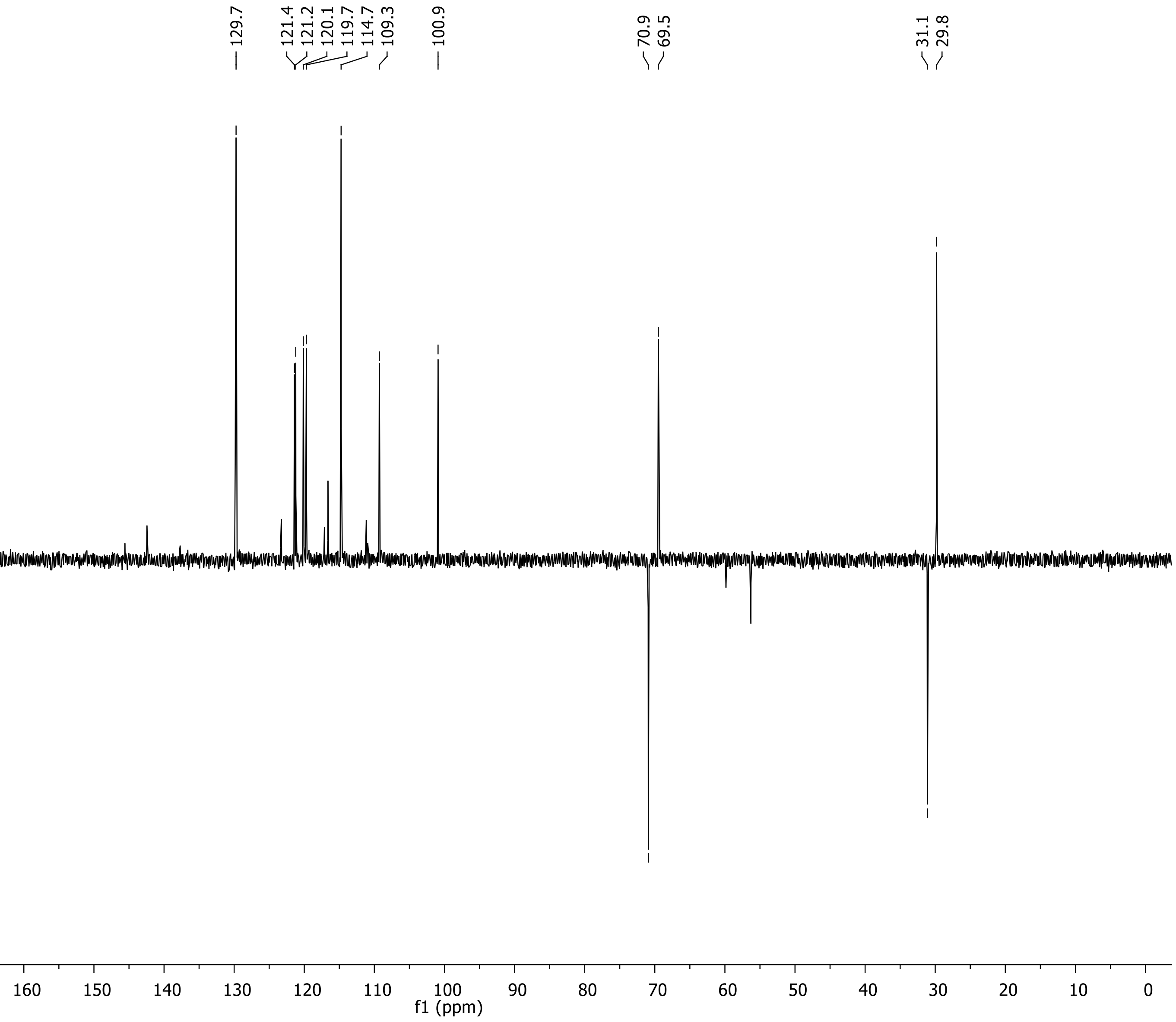

^{1}H NMR ($CDCl_3$, 300 MHz)

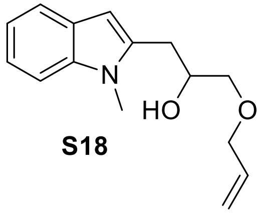

S18

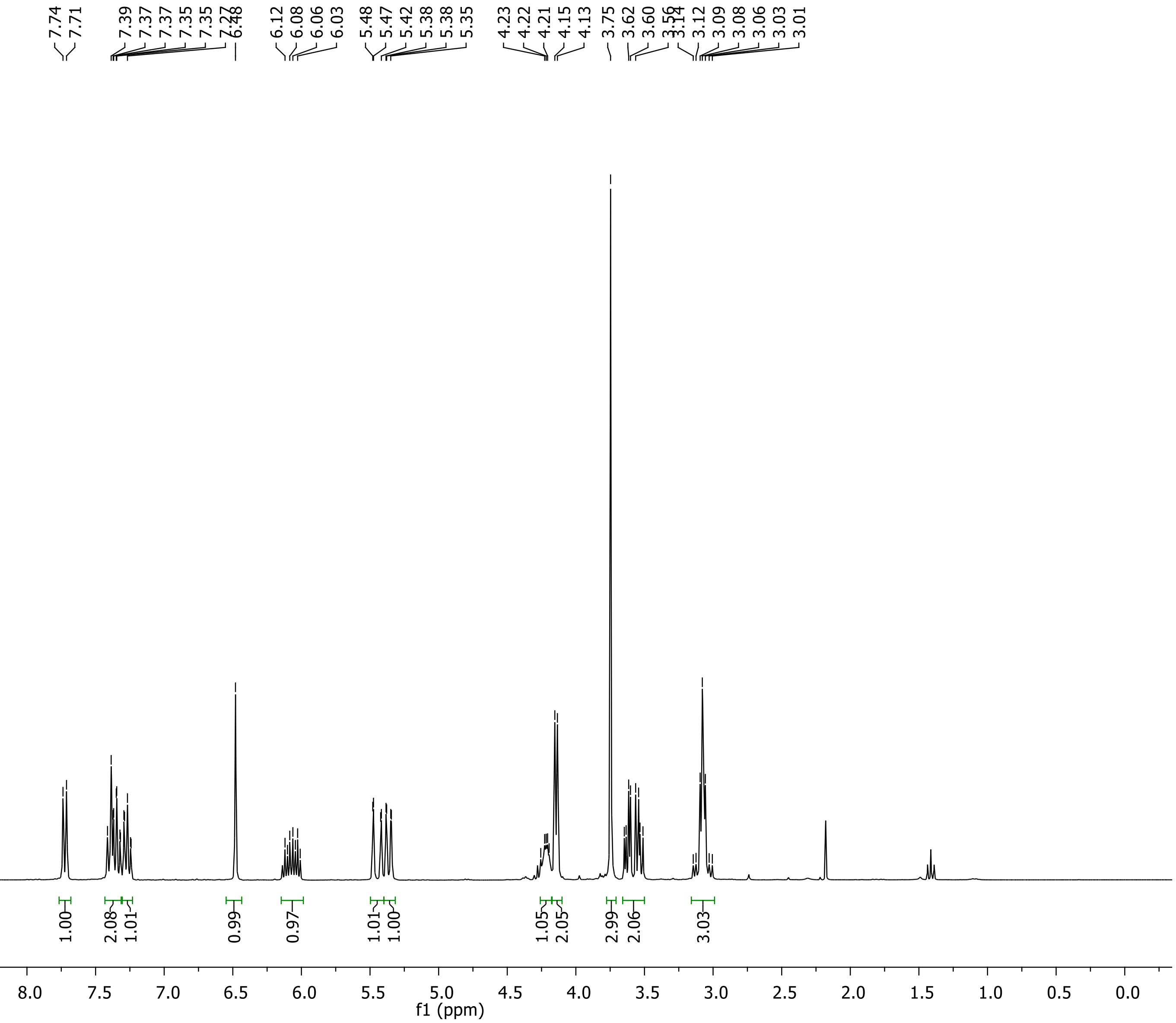

^{13}C NMR ($CDCl_3$, 75.4 MHz)

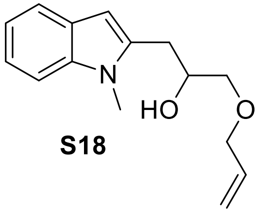

S18

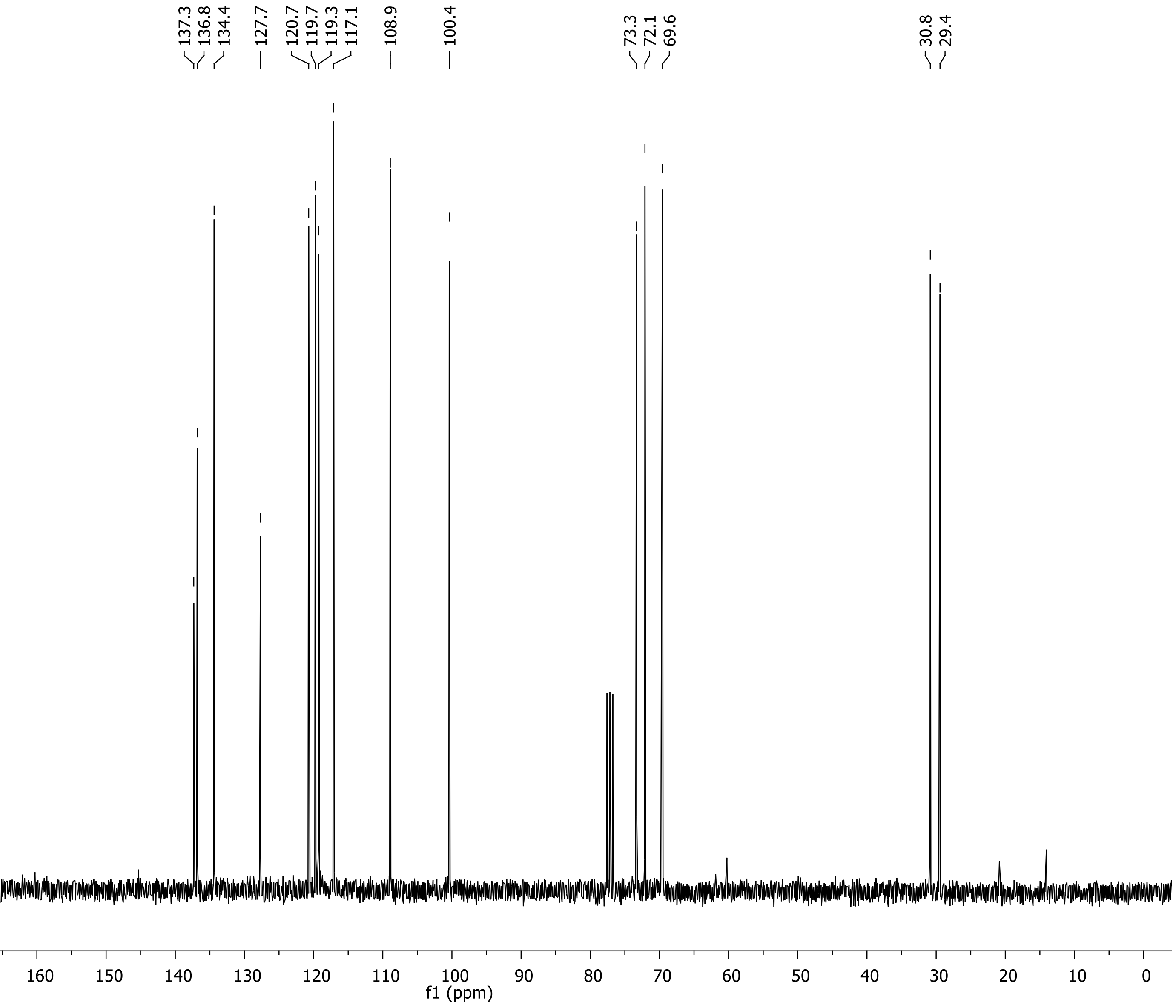

DEPT ($CDCl_3$, 75.4 MHz)

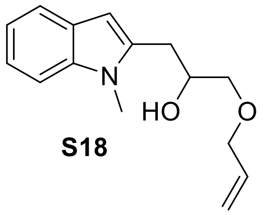

S18

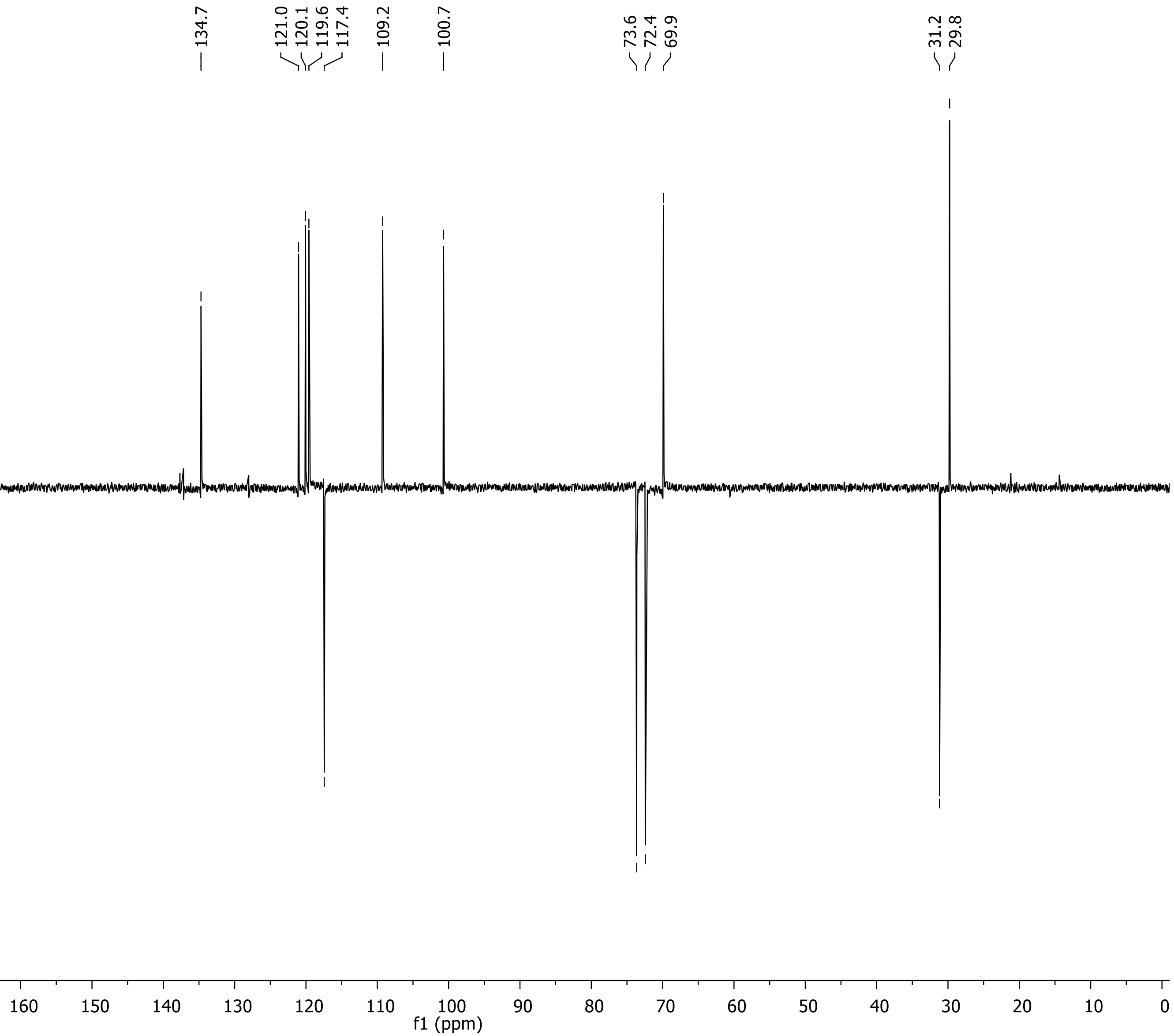

^{1}H NMR ($CDCl_3$, 300 MHz)

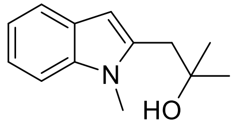

S19

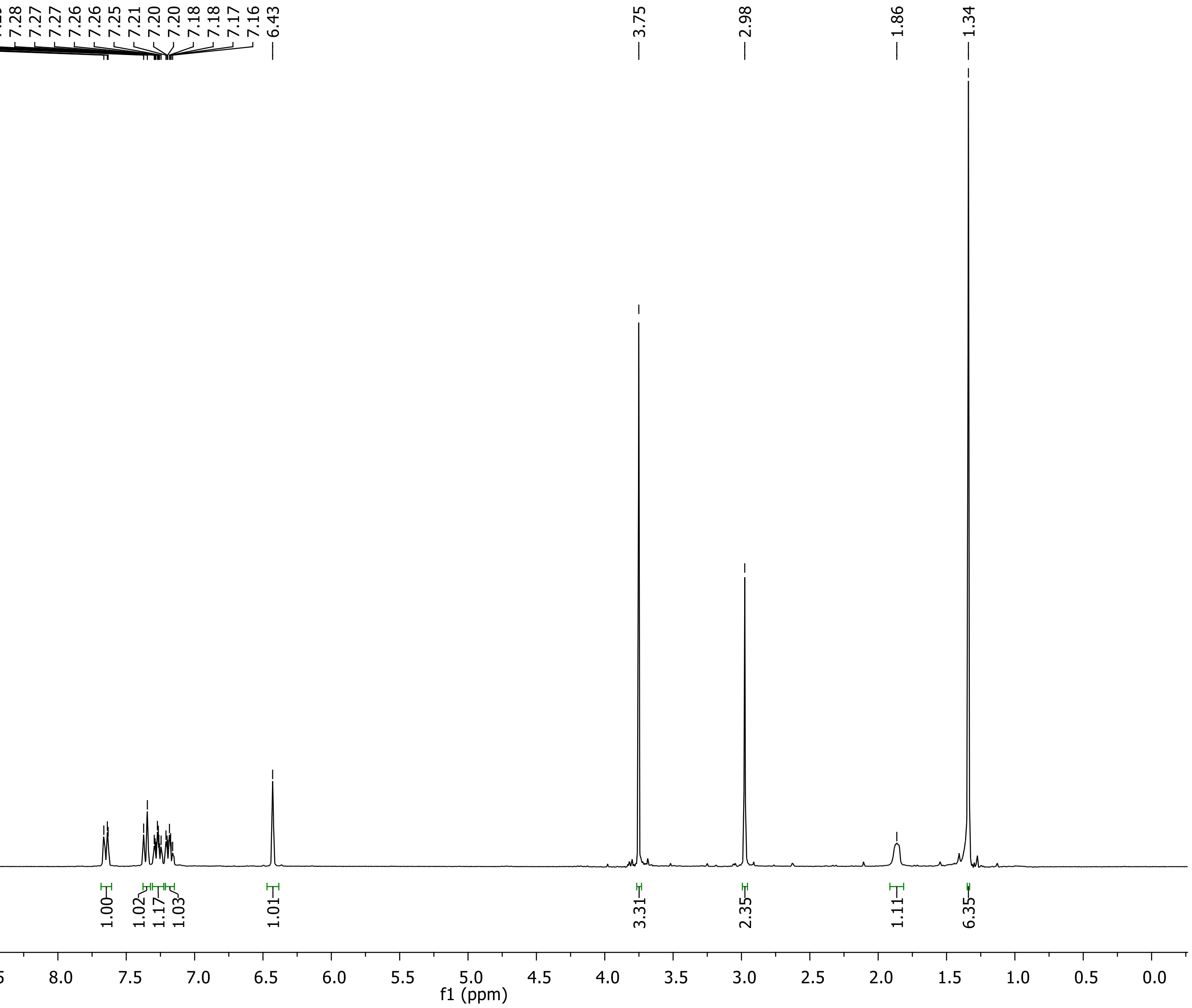

^{13}C NMR ($CDCl_3$, 75.4 MHz)

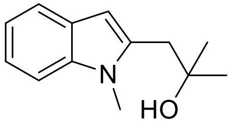

S19

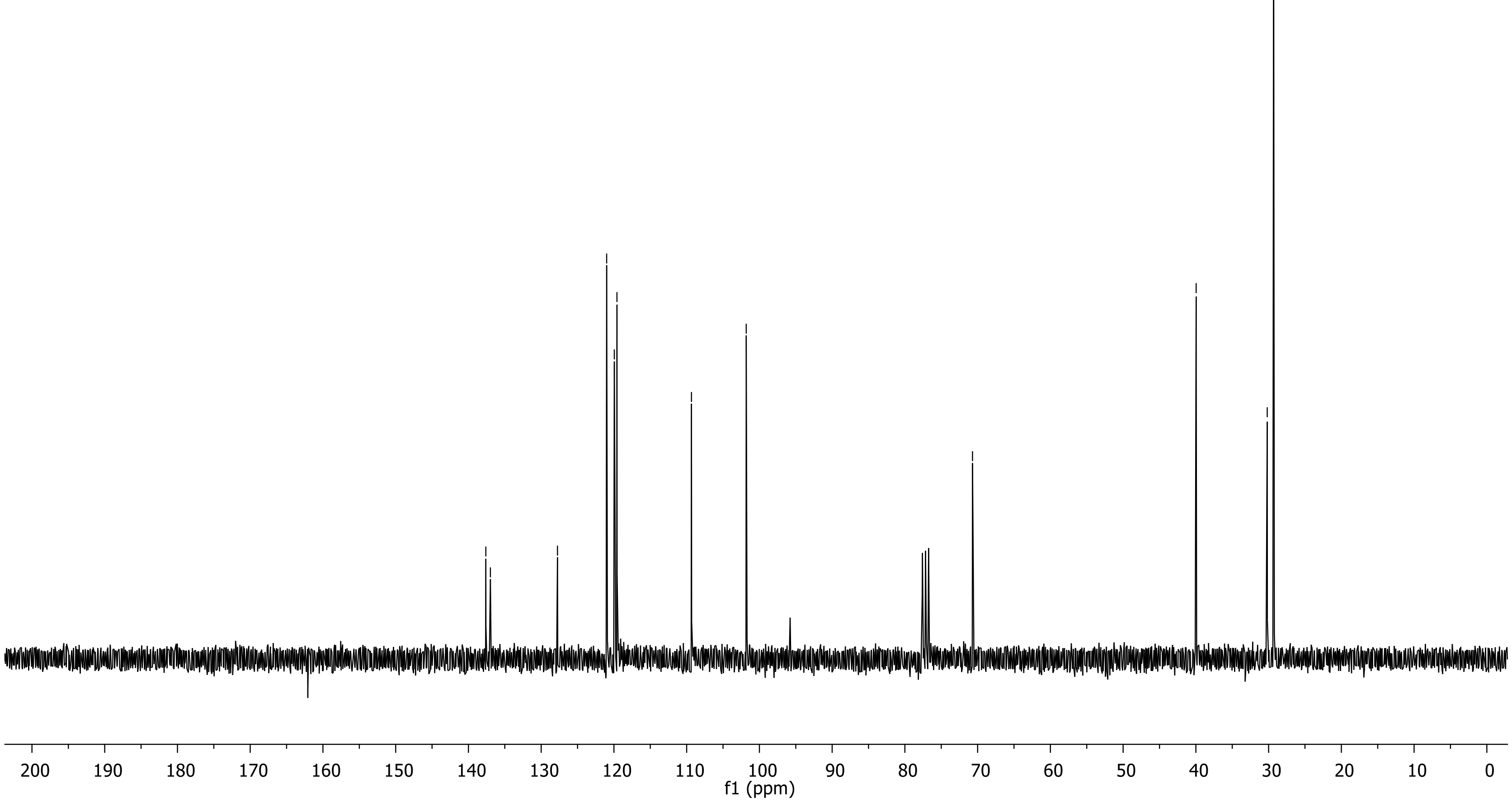

^{1}H NMR ($CDCl_3$, 300 MHz)

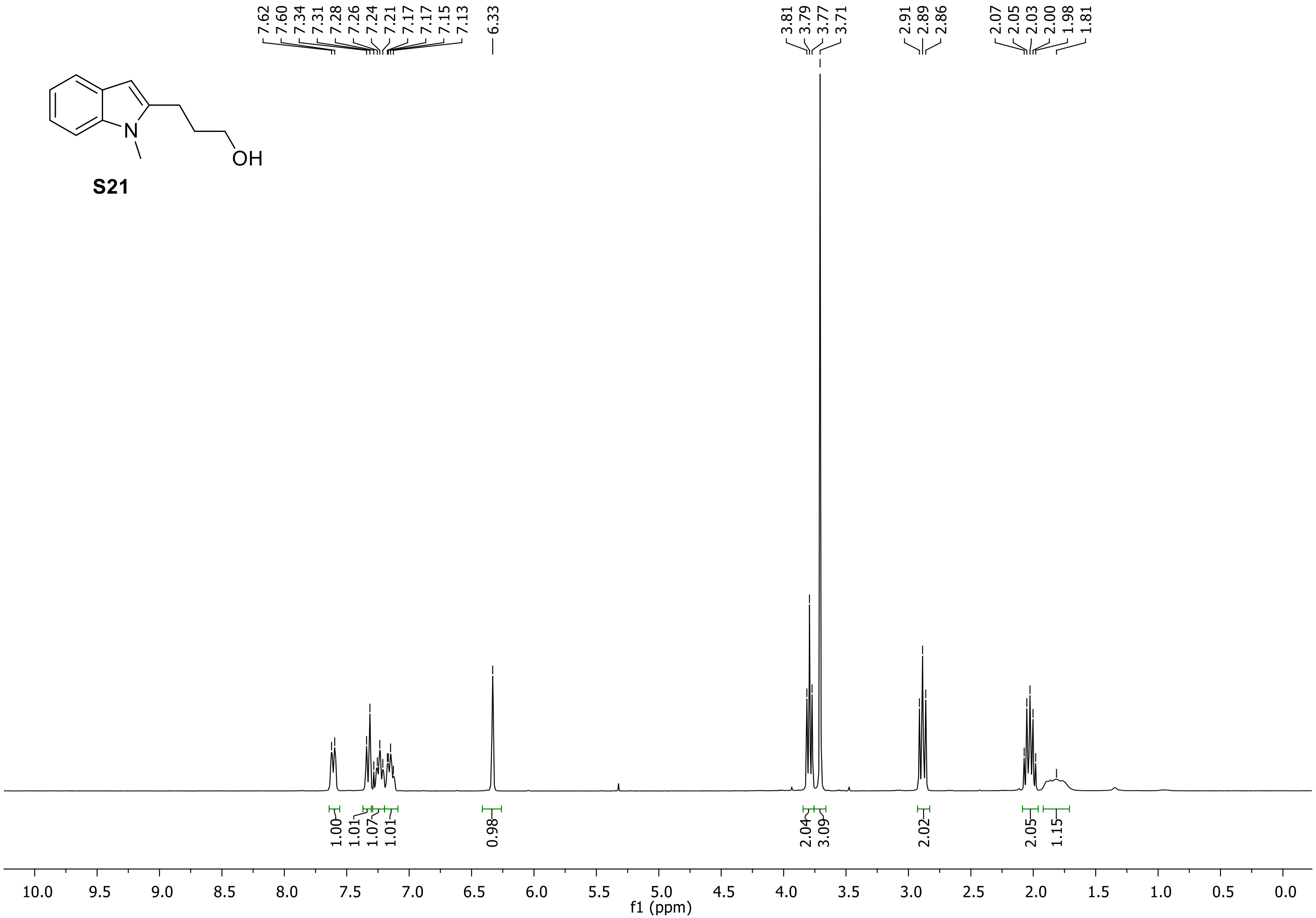

^{13}C NMR ($CDCl_3$, 75.4 MHz)

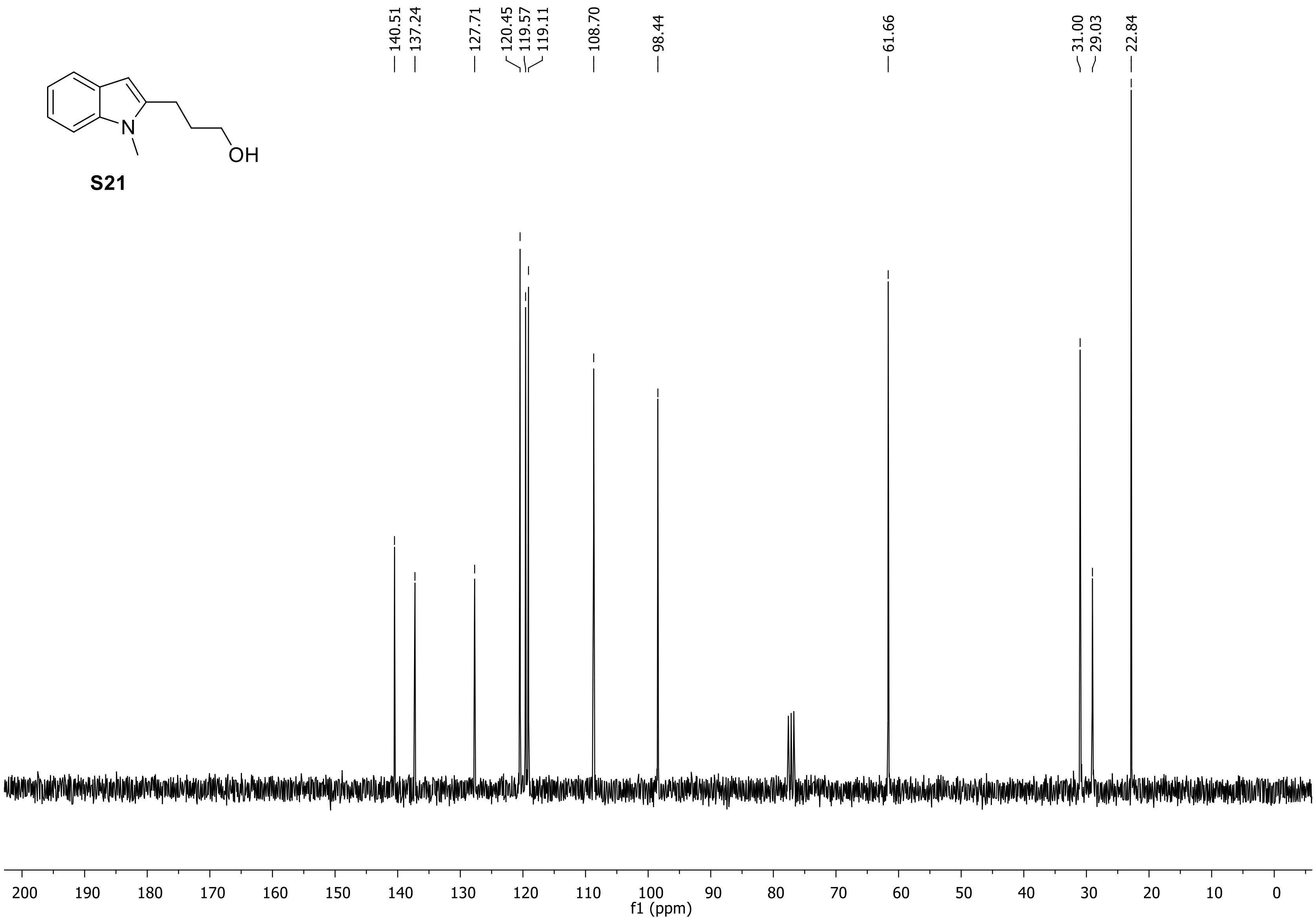

DEPT ($CDCl_3$, 75.4 MHz)

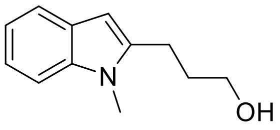

S21

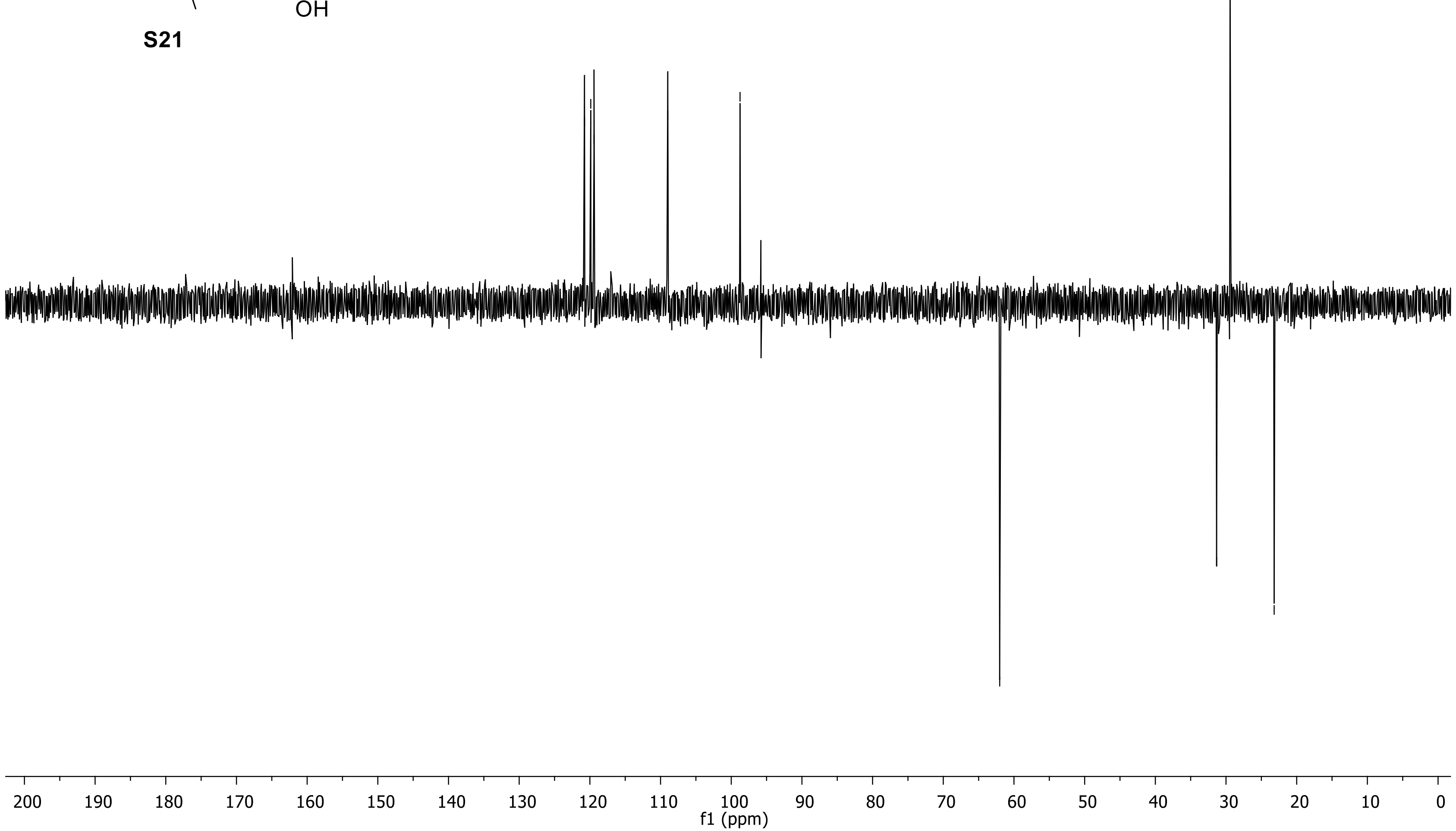

^{1}H NMR ($CDCl_3$, 300 MHz)

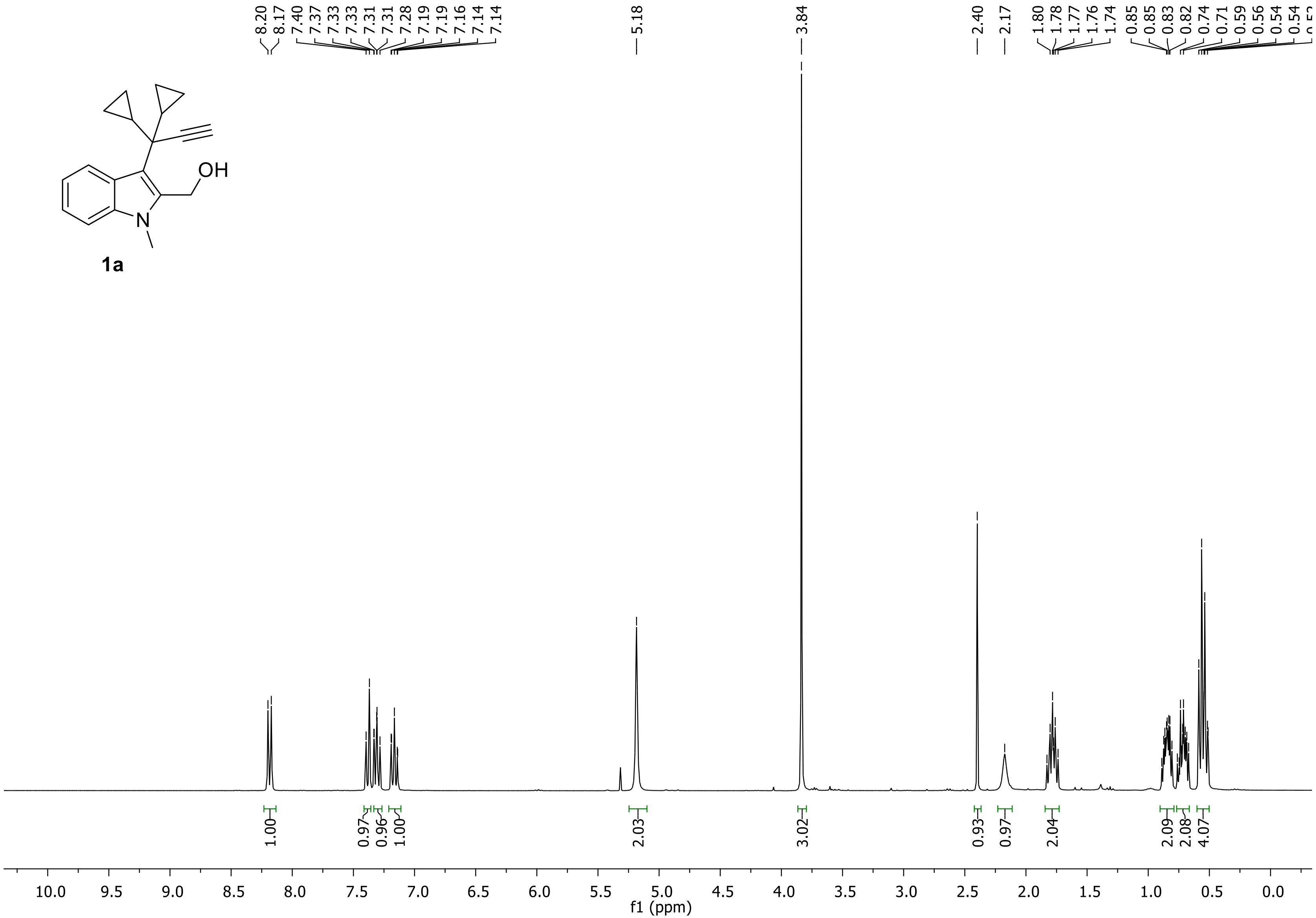

1a

^{13}C NMR ($CDCl_3$, 75.4 MHz)

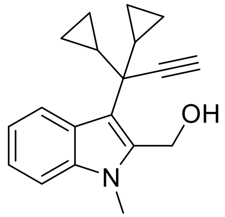

1a

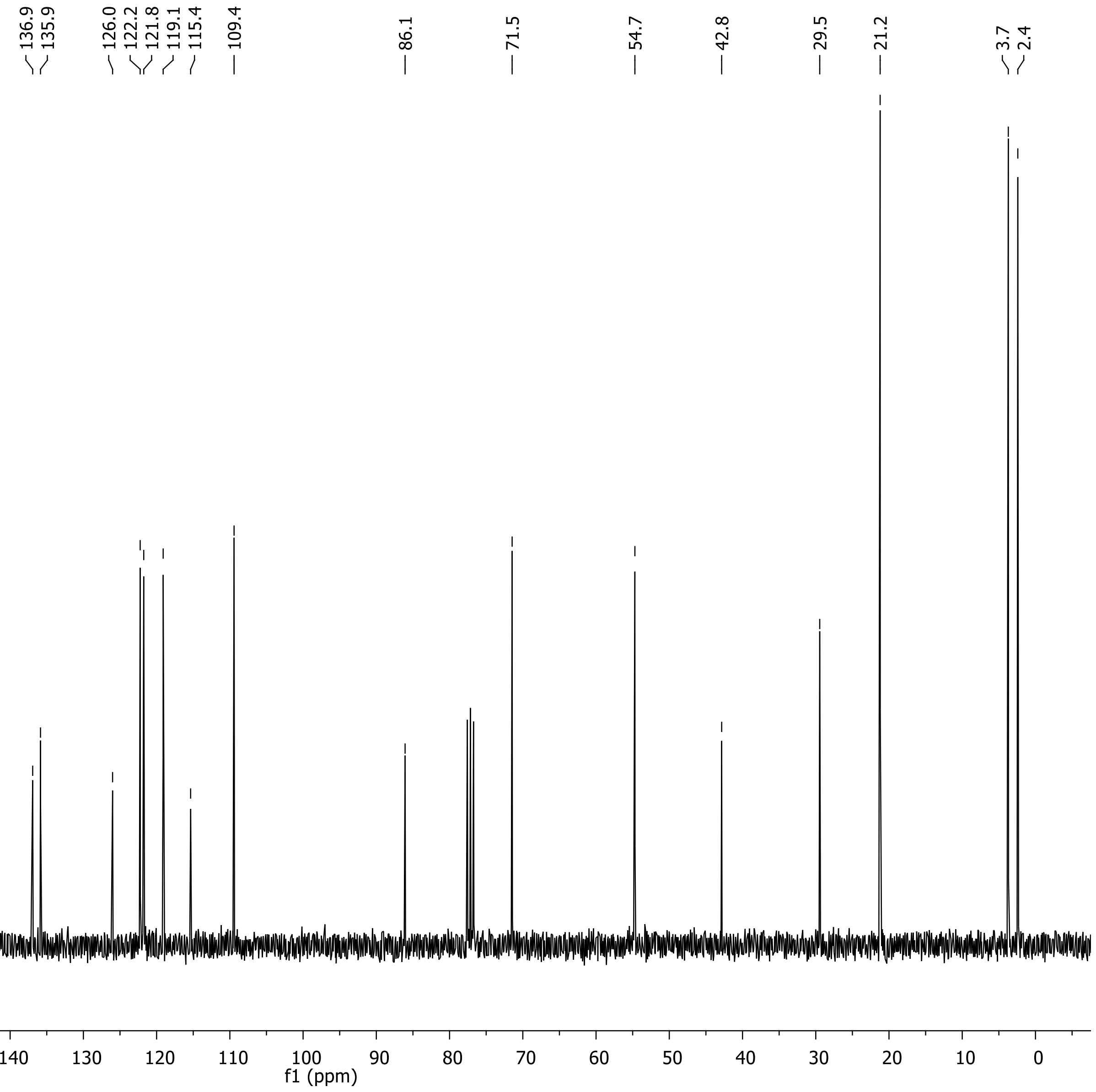

DEPT ($CDCl_3$, 75.4 MHz)

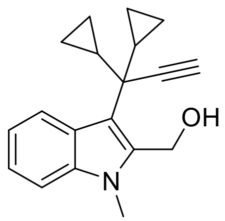

1a

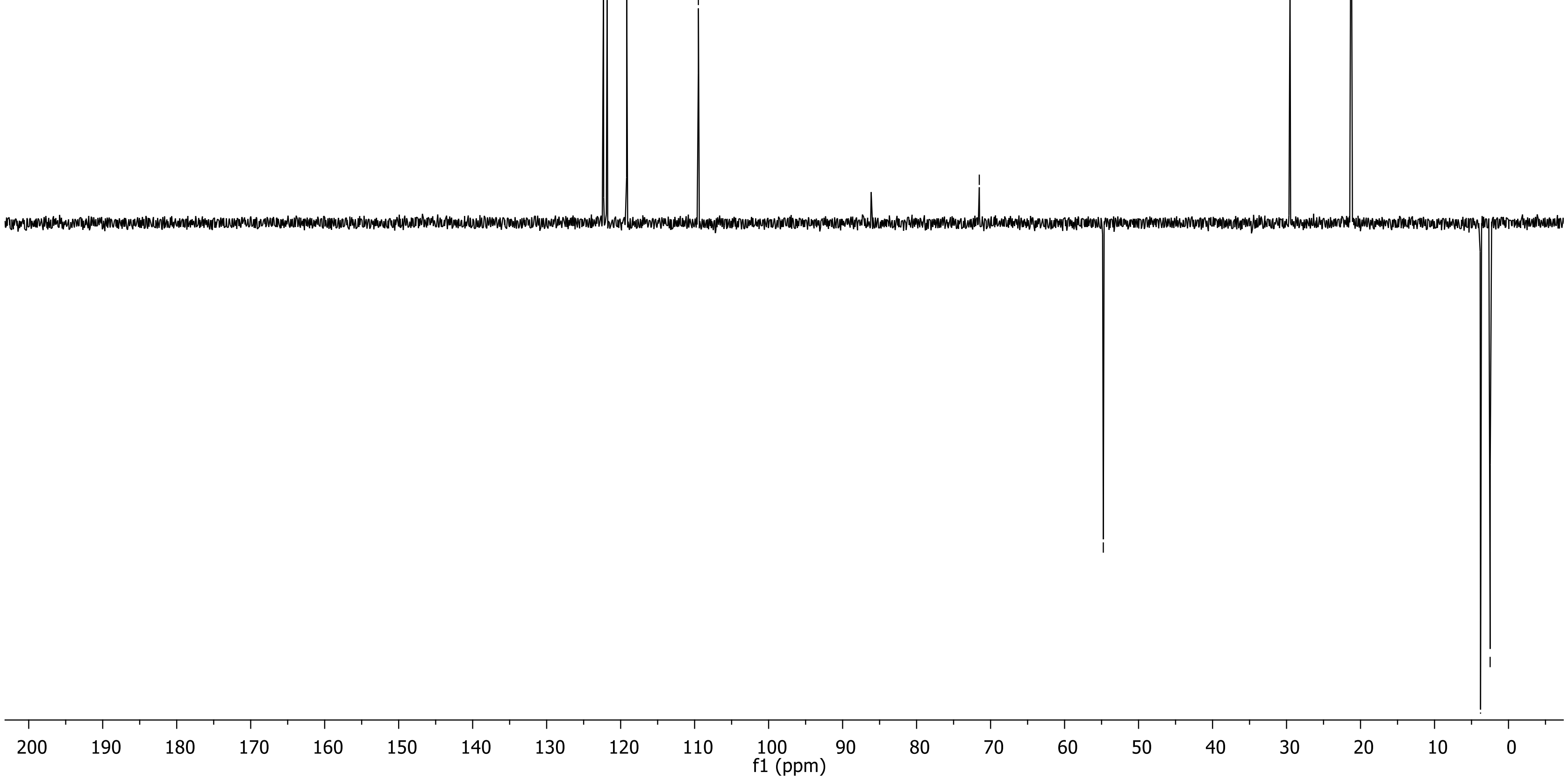

^{1}H NMR (CDCl$_3$, 300 MHz)

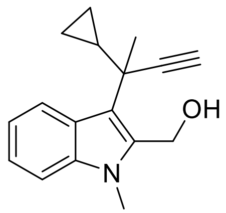

1b

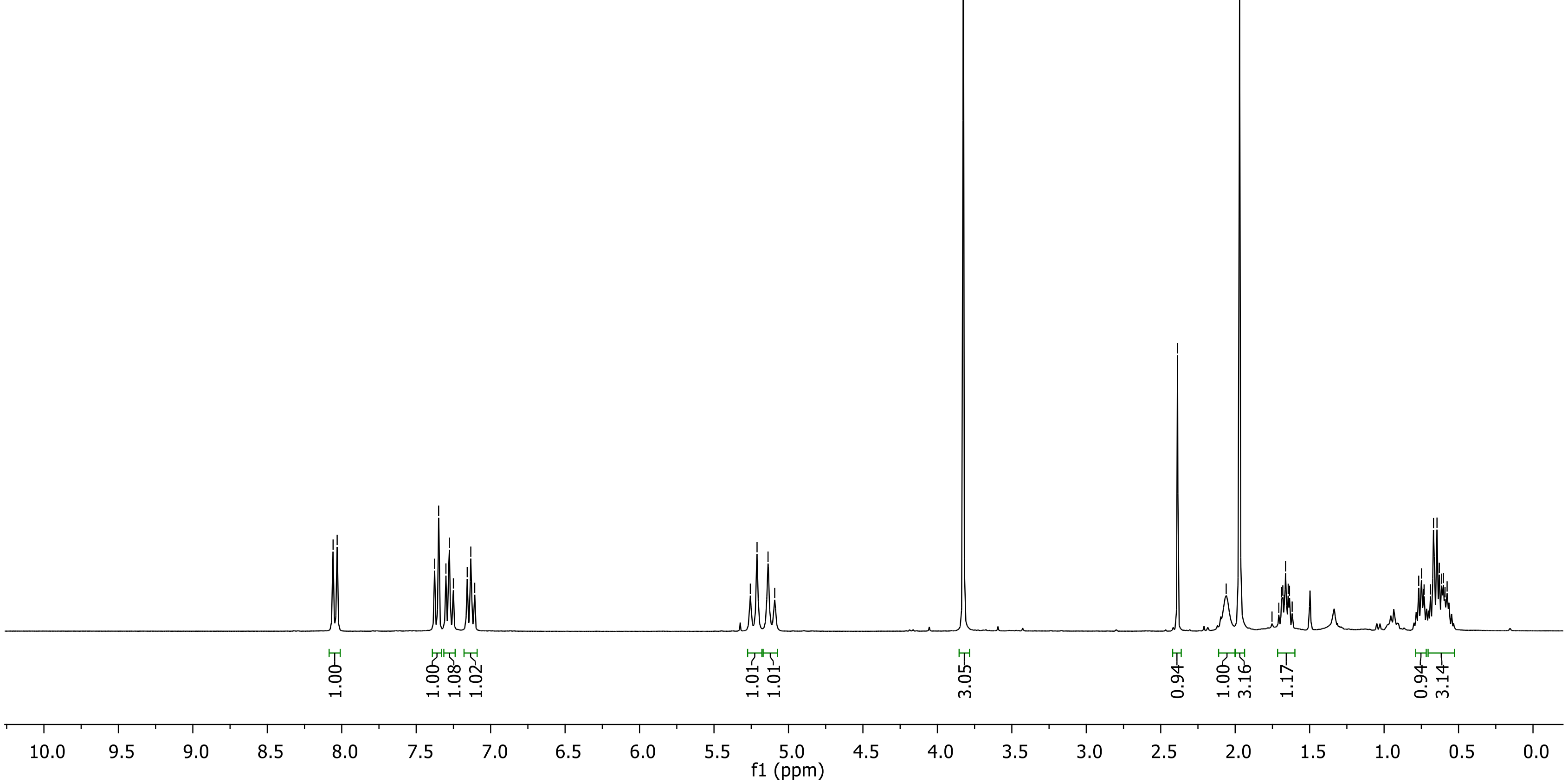

^{13}C NMR ($CDCl_3$, 75.4 MHz)

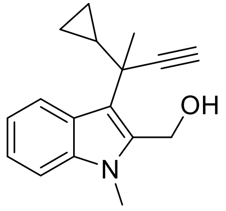

1b

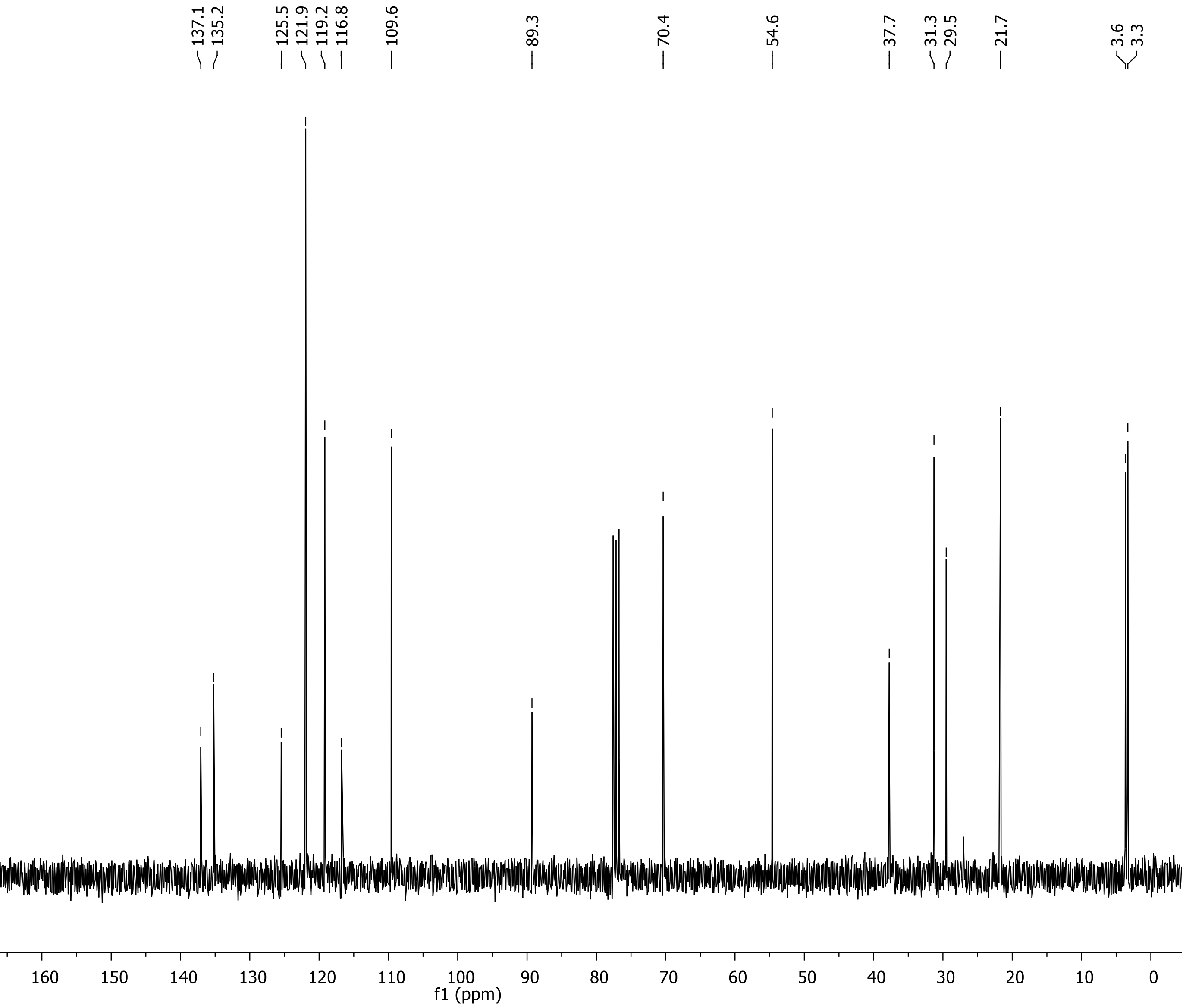

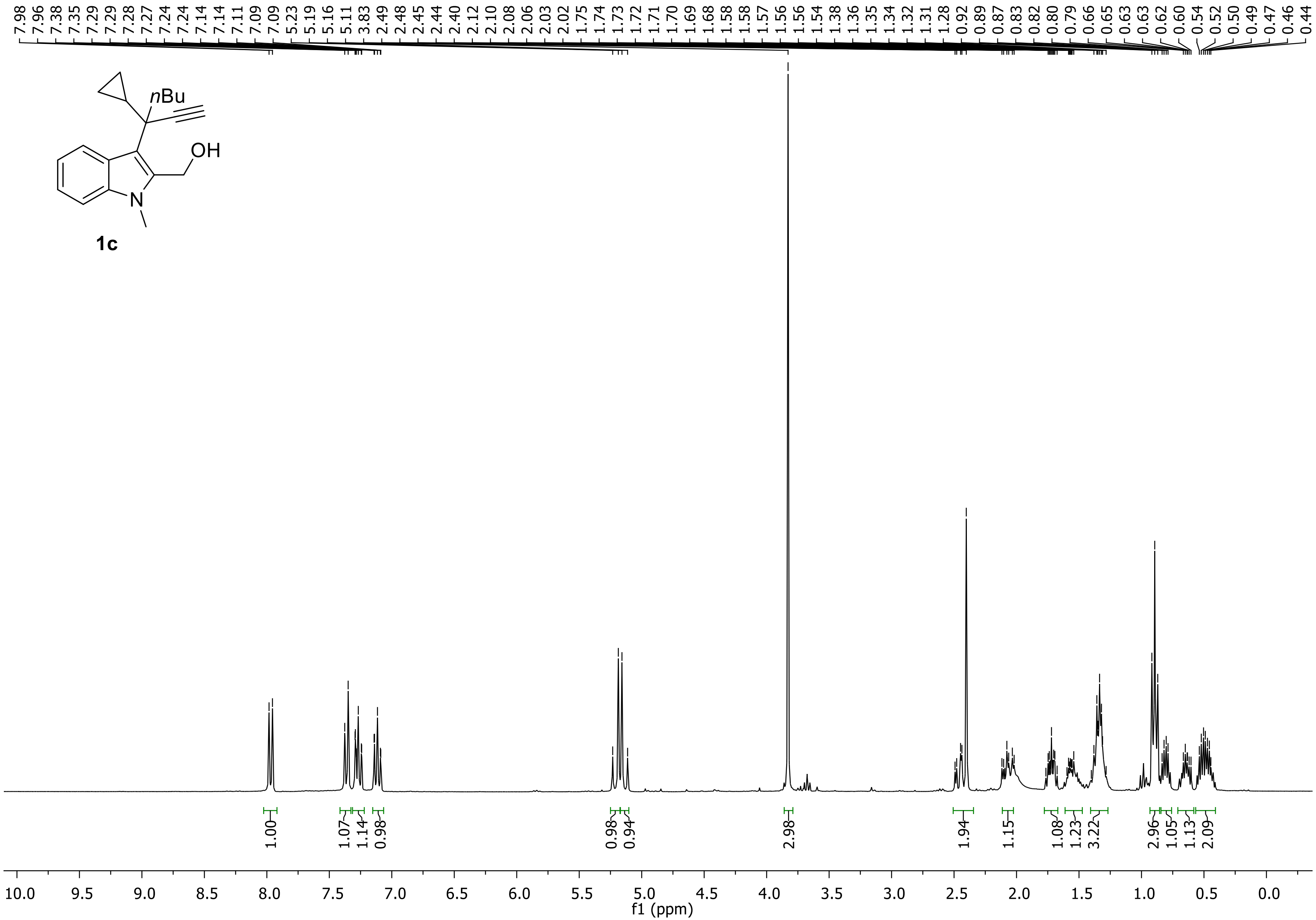

1c

^{13}C NMR ($CDCl_3$, 75.4 MHz)

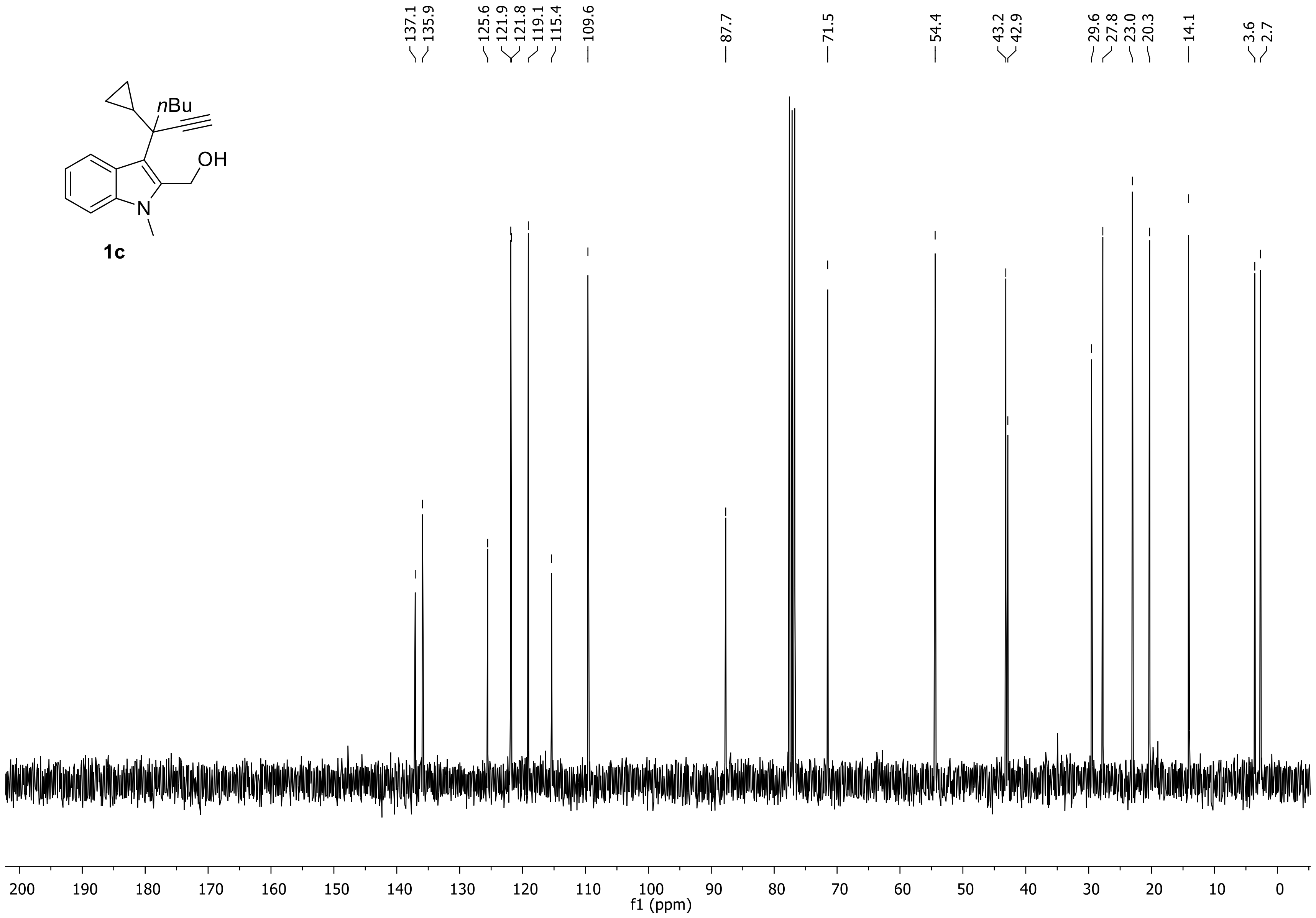

DEPT ($CDCl_3$, 75.4 MHz)

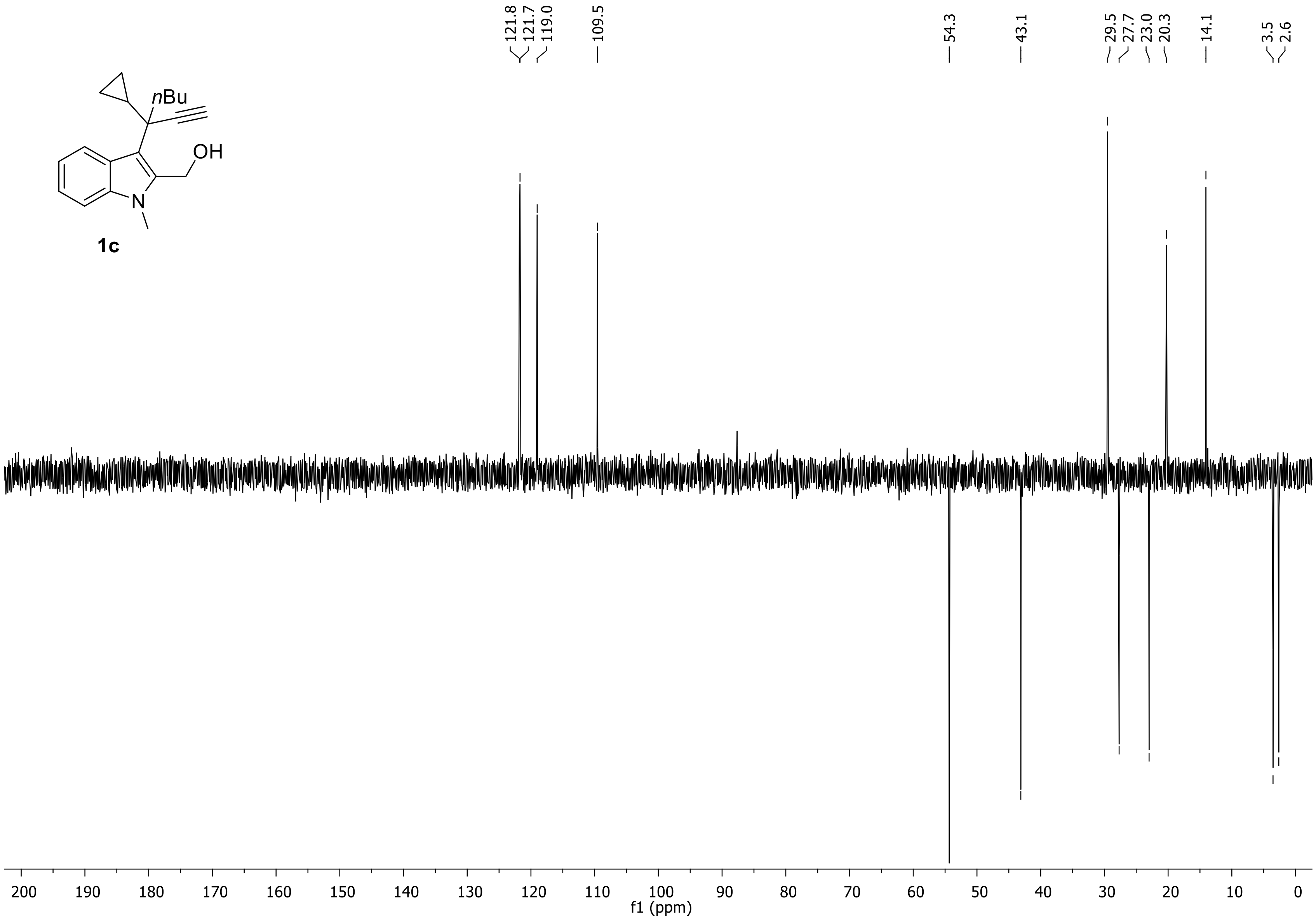

^{1}H NMR (CDCl$_3$, 300 MHz)

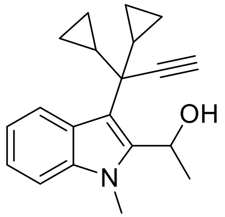

1d

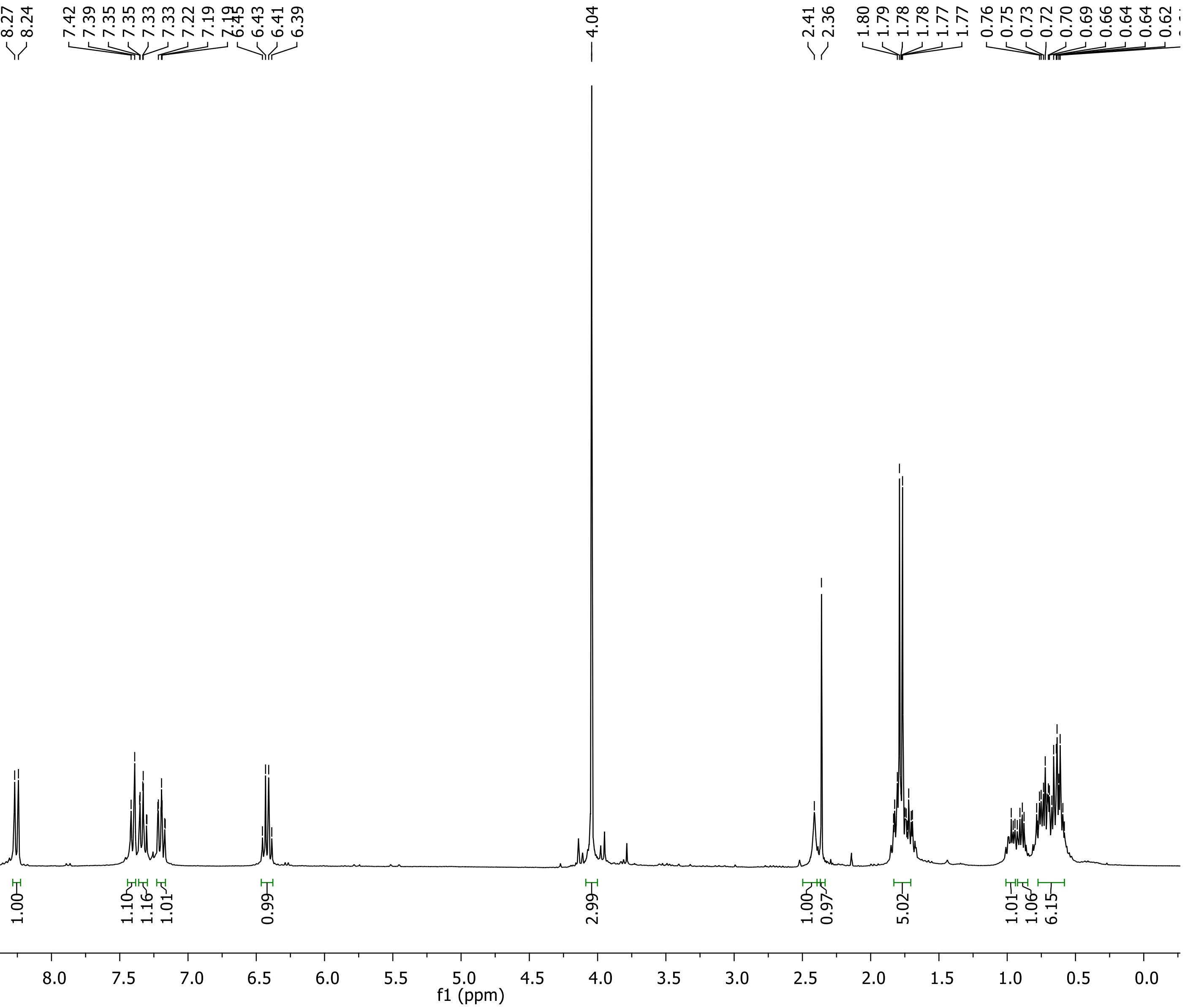

^{13}C NMR ($CDCl_3$, 75.4 MHz)

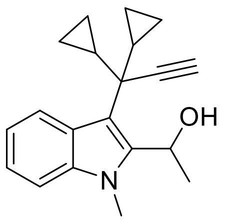

1d

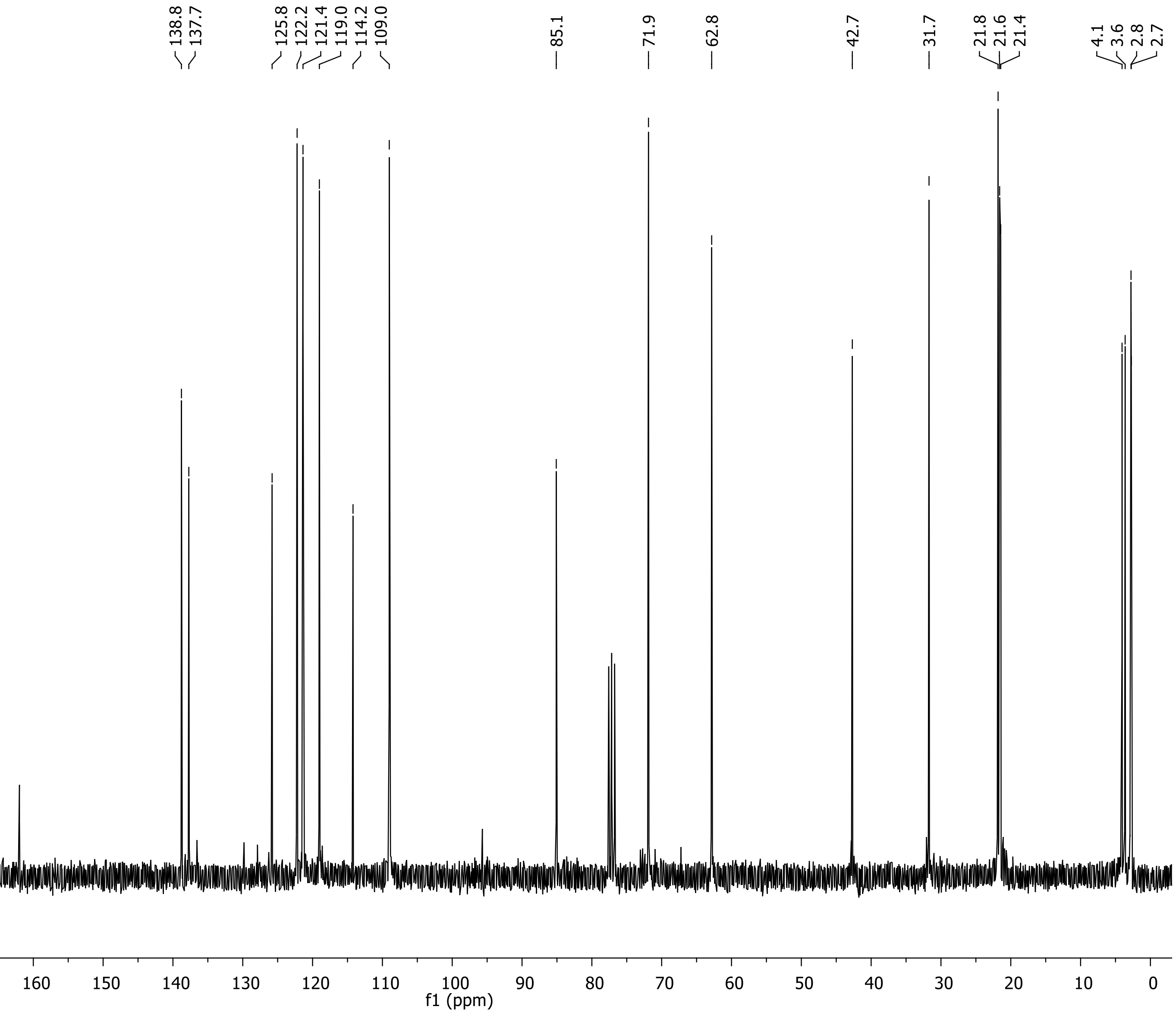

DEPT ($CDCl_3$, 75.4 MHz)

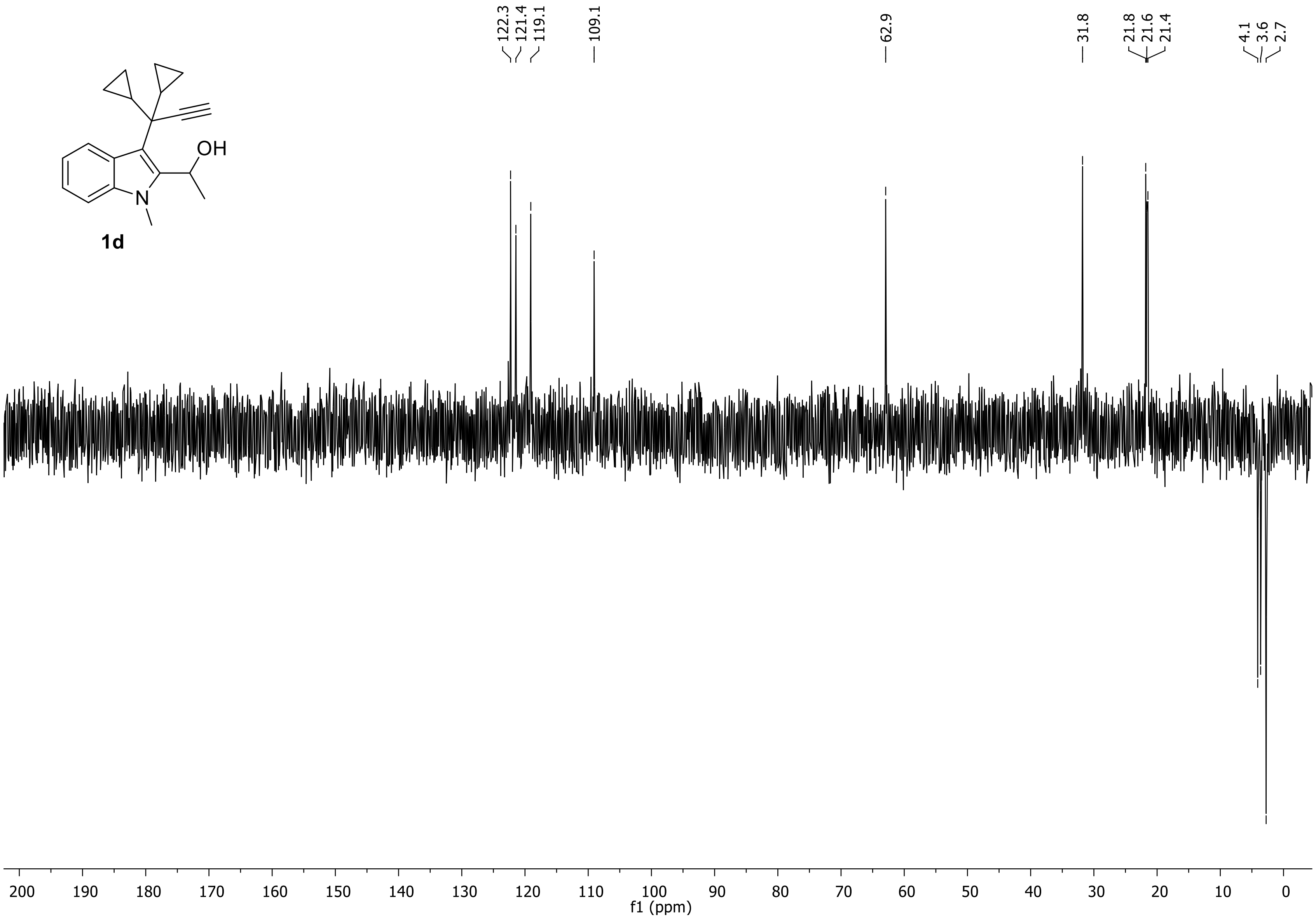

^{1}H NMR ($CDCl_3$, 300 MHz)

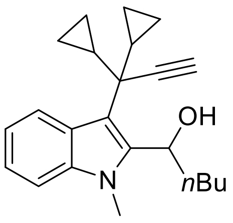

1e

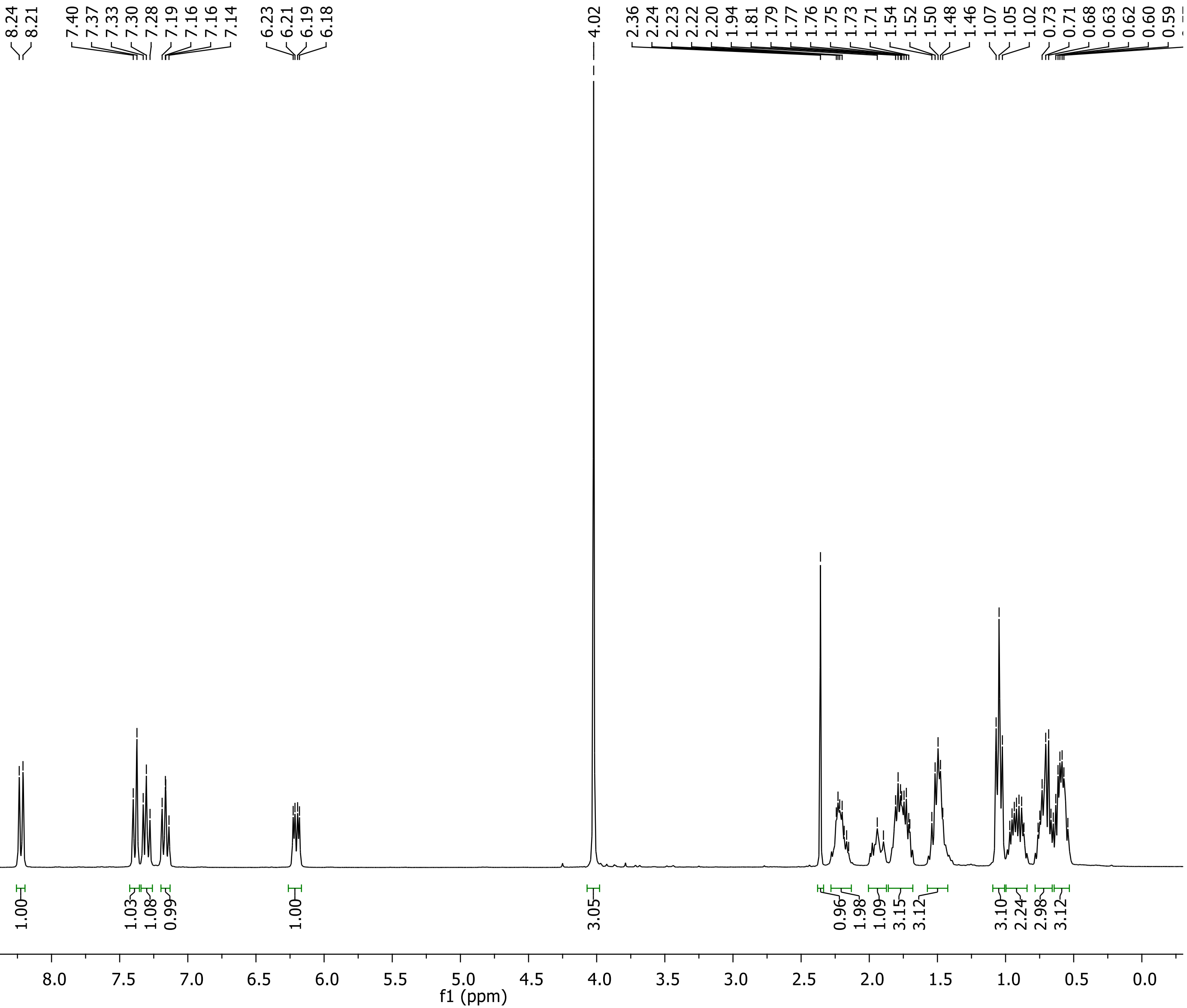

^{13}C NMR ($CDCl_3$, 75.4 MHz)

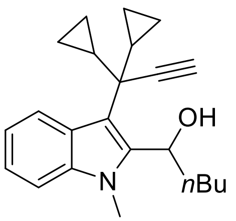

1e

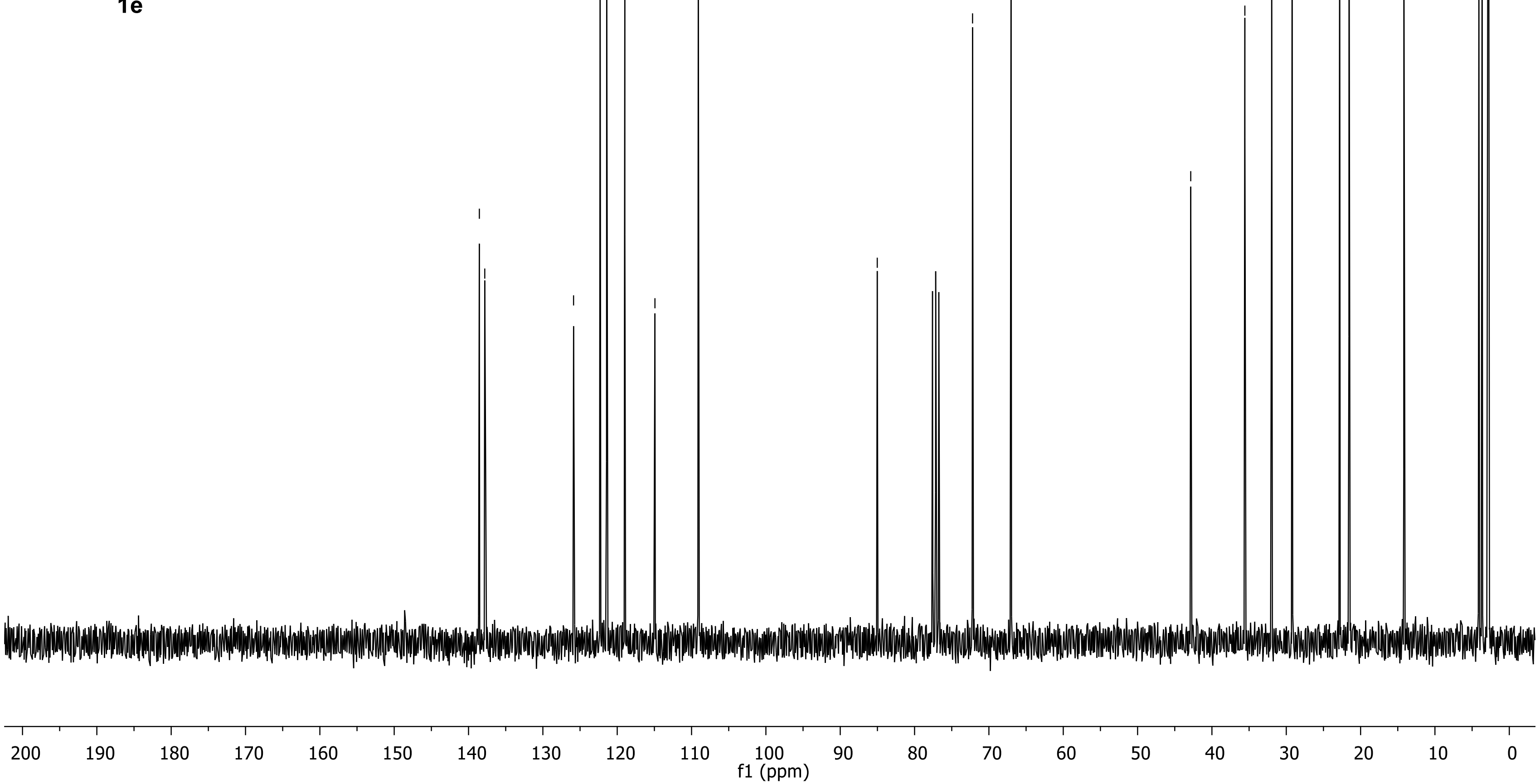

DEPT ($CDCl_3$, 75.4 MHz)

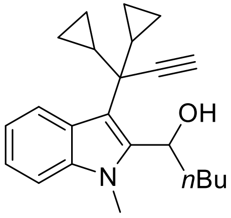

1e

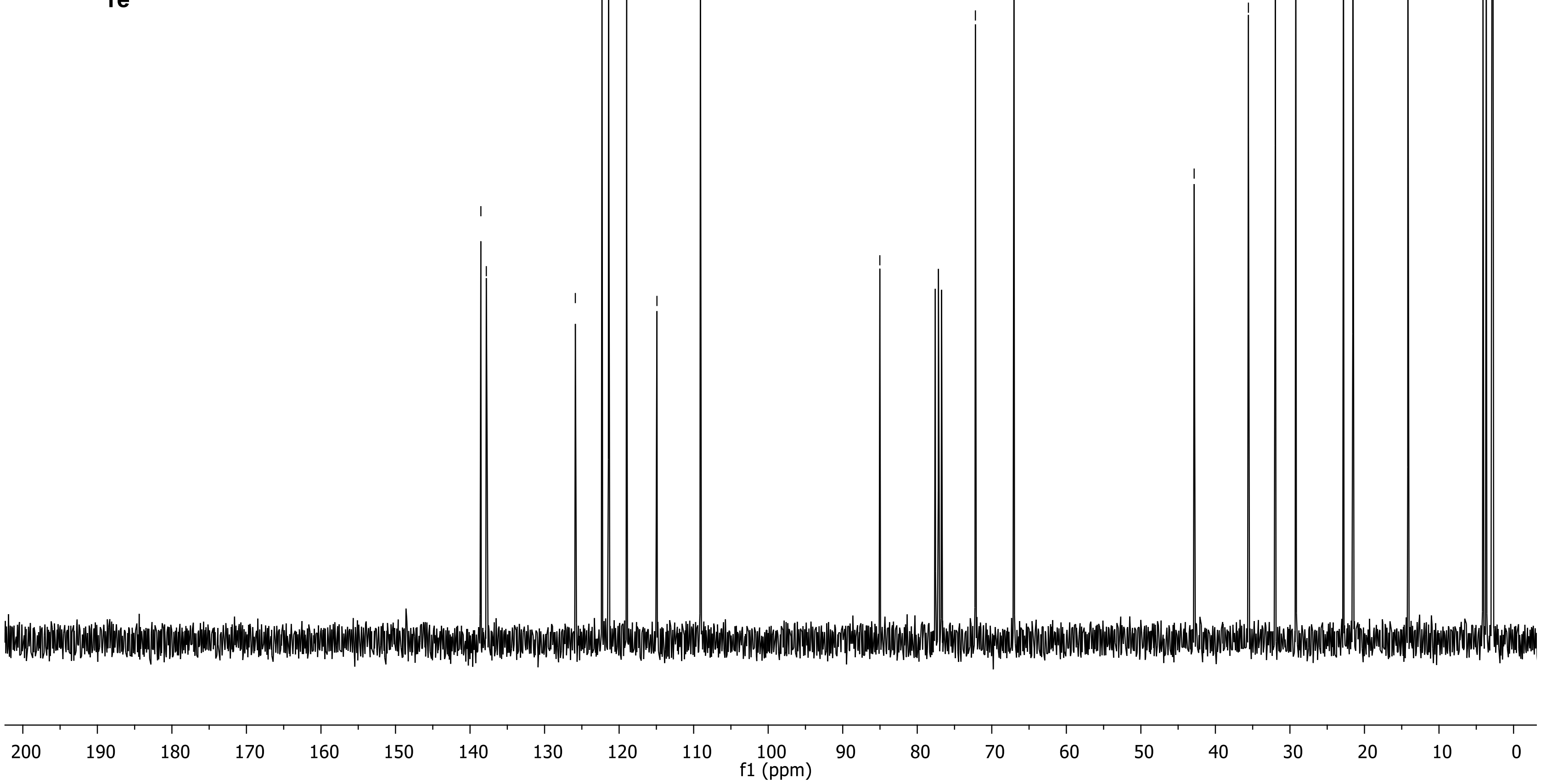

^{1}H NMR ($CDCl_3$, 300 MHz)

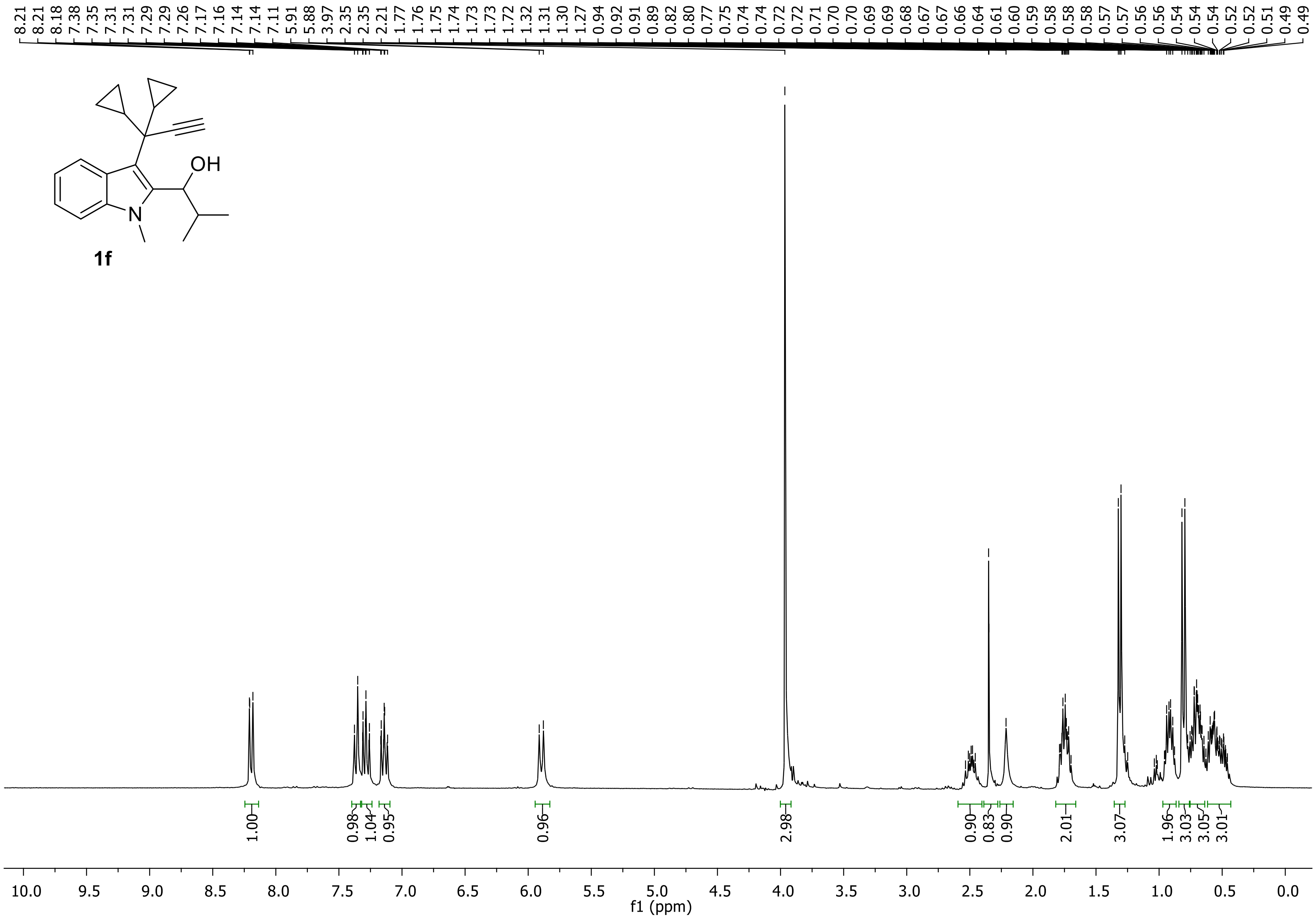

^{13}C NMR ($CDCl_3$, 75.4 MHz)

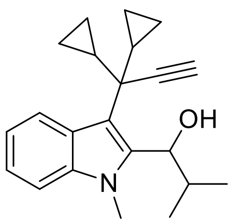

1f

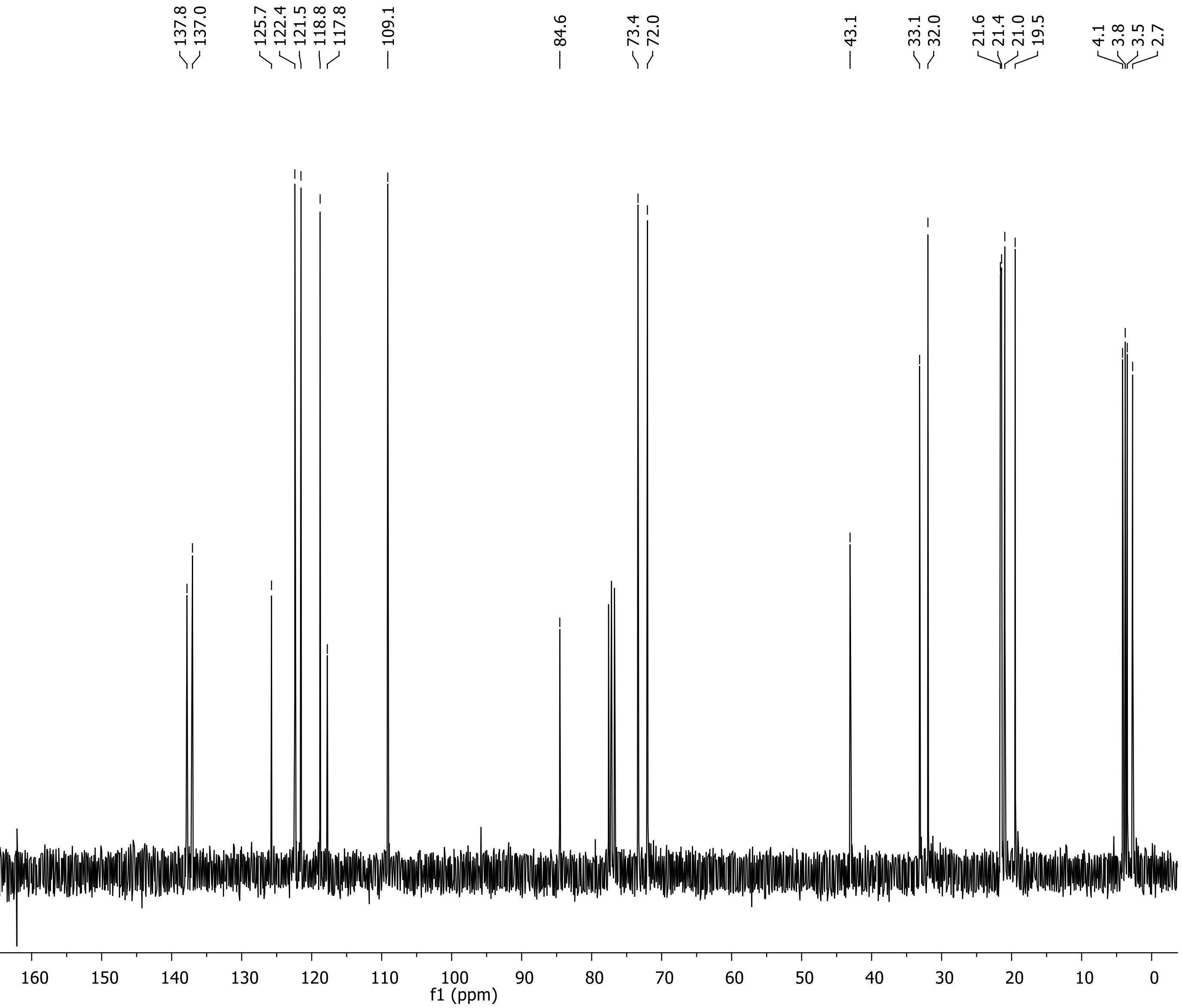

DEPT ($CDCl_3$, 75.4 MHz)

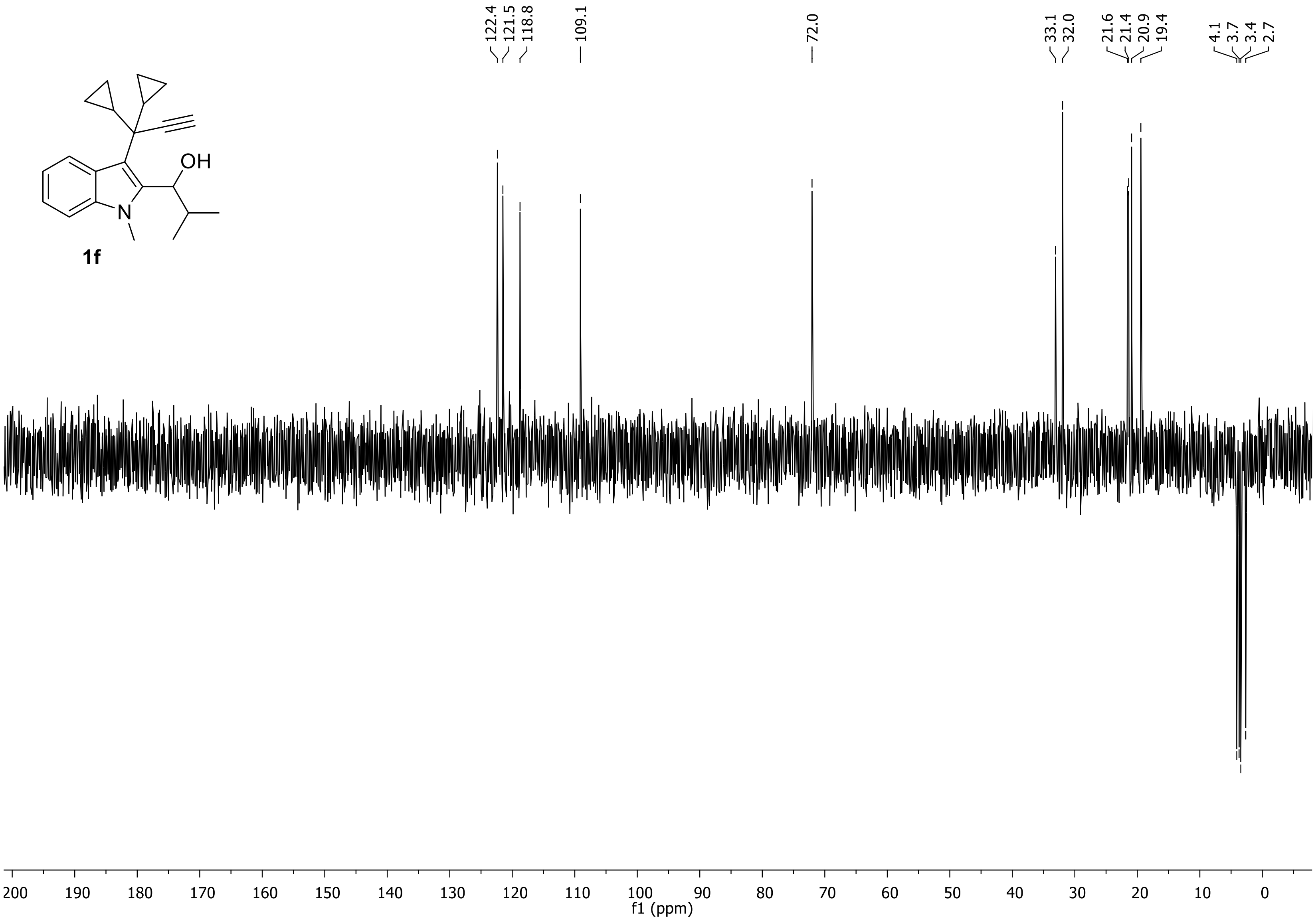

1f

^{1}H NMR ($CDCl_3$, 300 MHz)

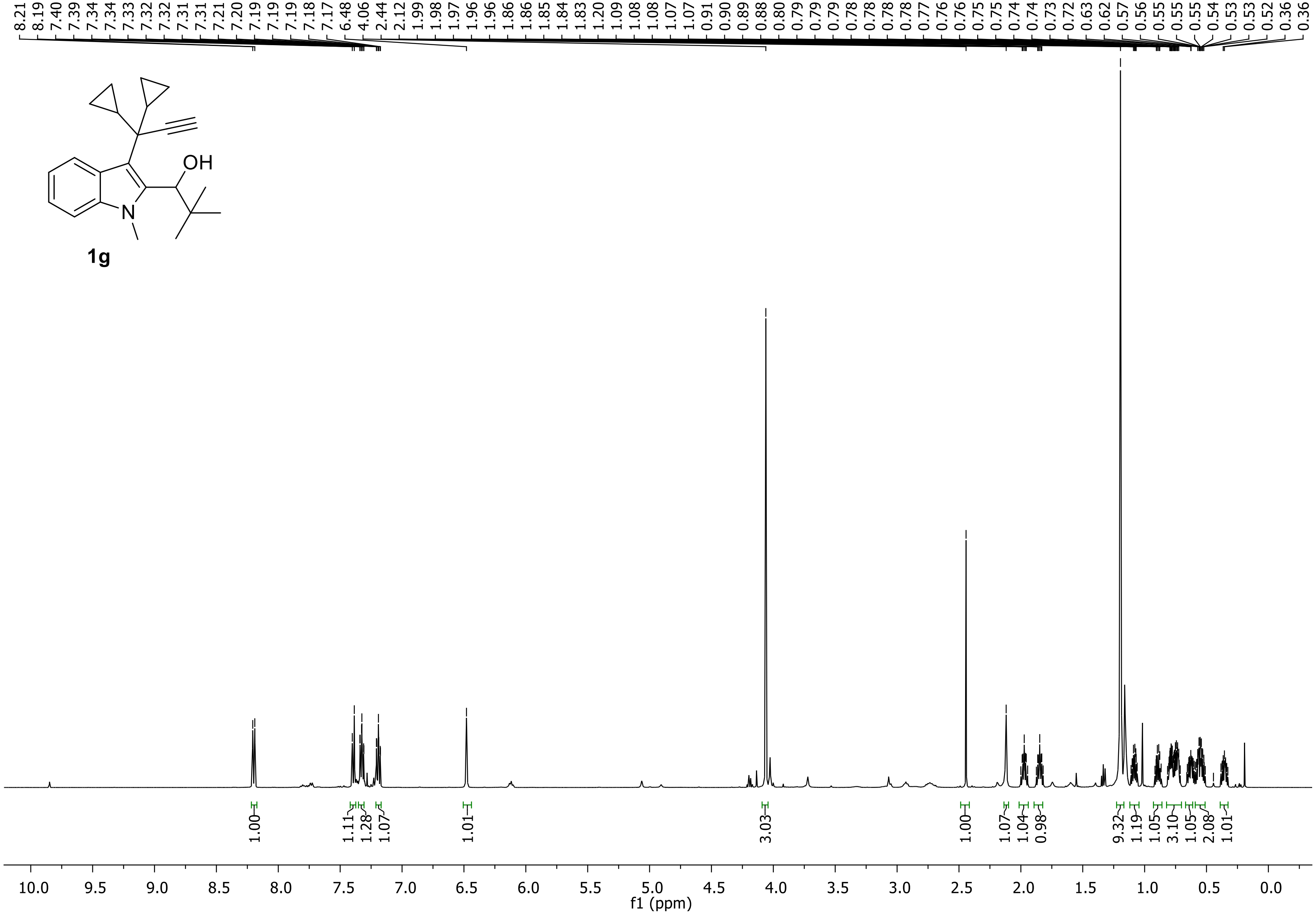

^{13}C NMR ($CDCl_3$, 75.4 MHz)

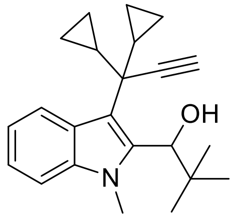

1g

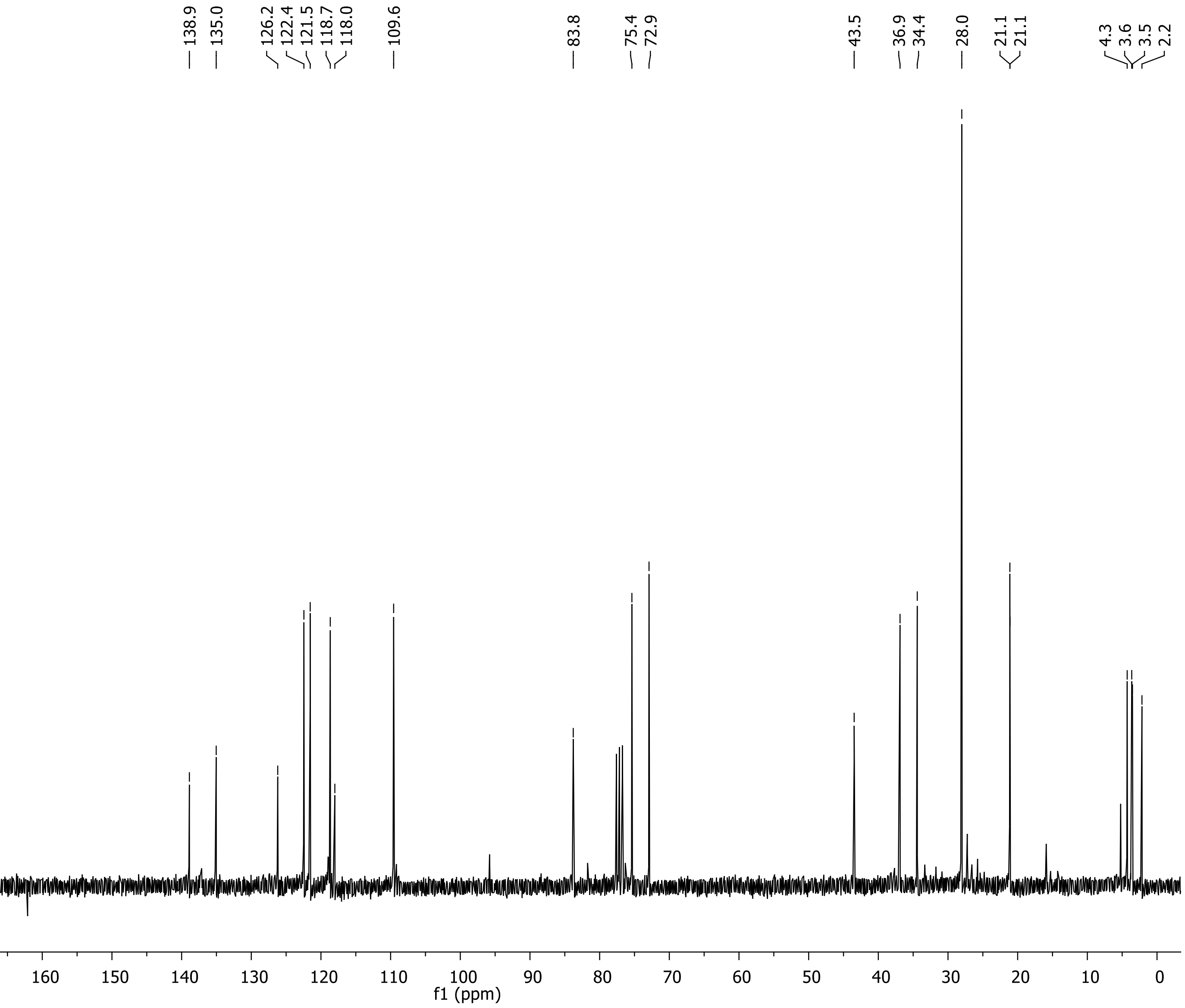

DEPT ($CDCl_3$, 75.4 MHz)

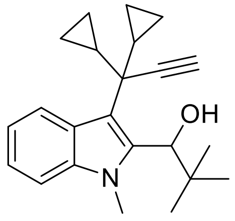

1g

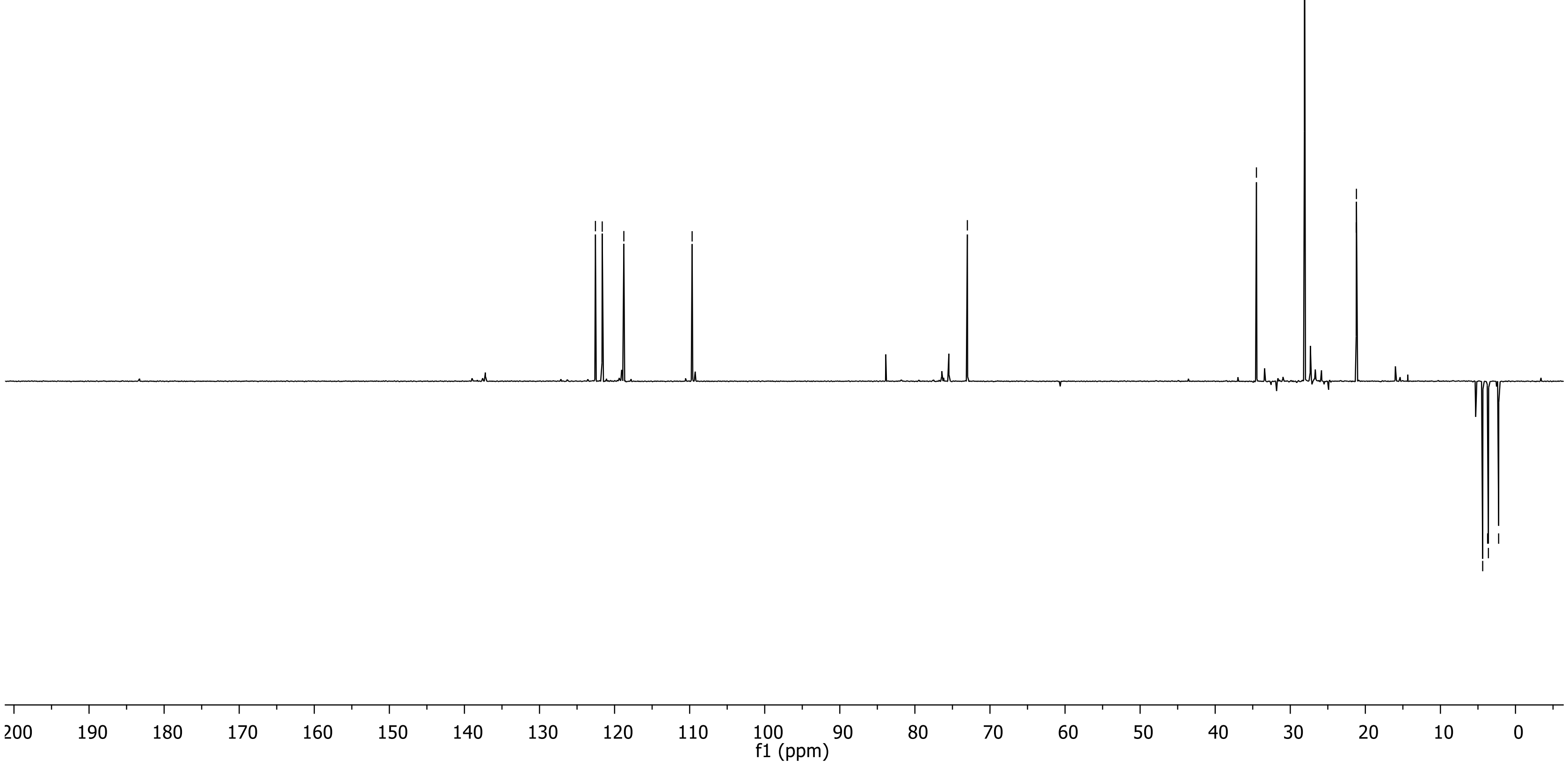

^{1}H NMR ($CDCl_3$, 300 MHz)

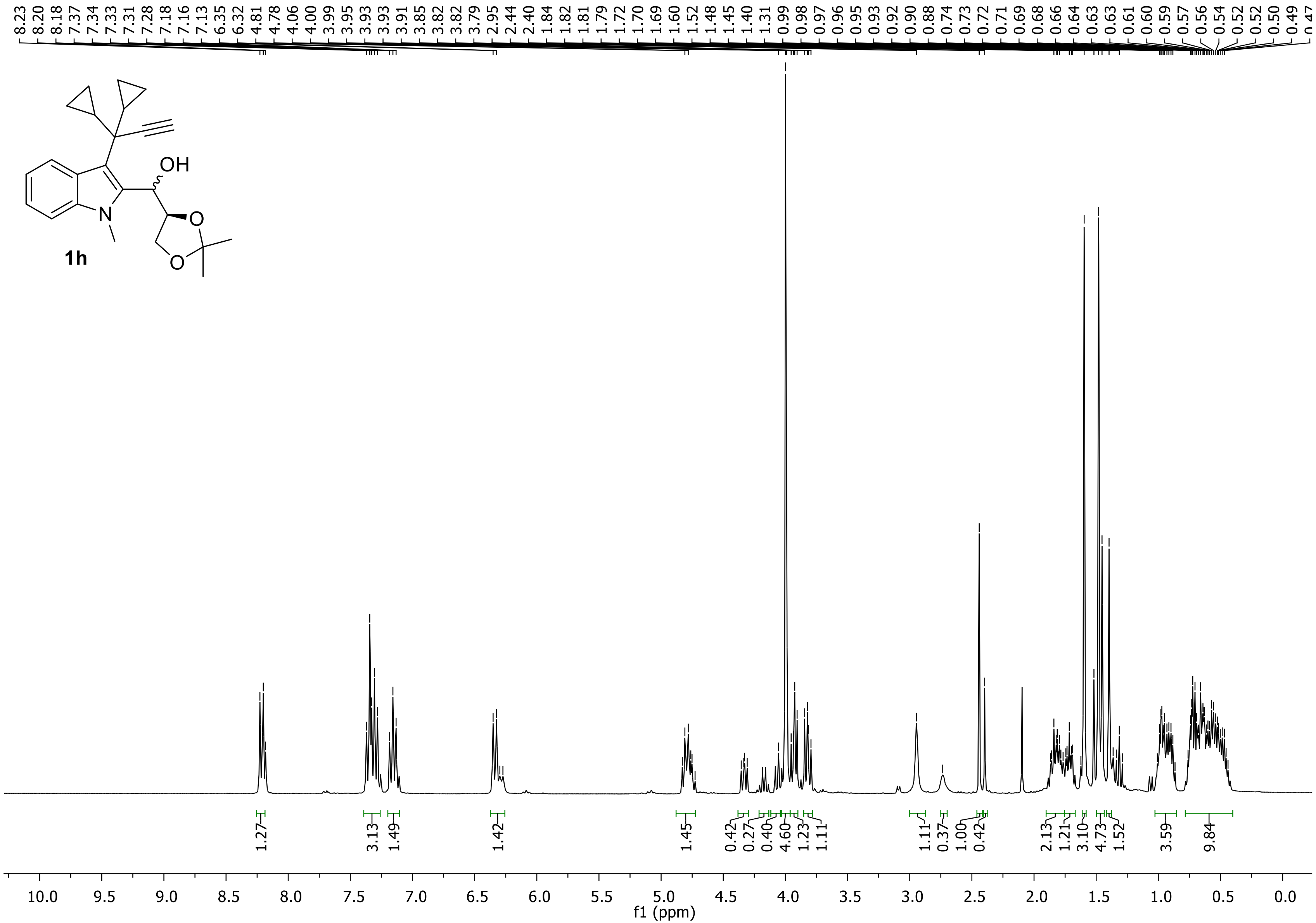

1h

^{13}C NMR ($CDCl_3$, 75.4 MHz)

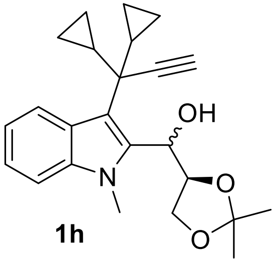

1h

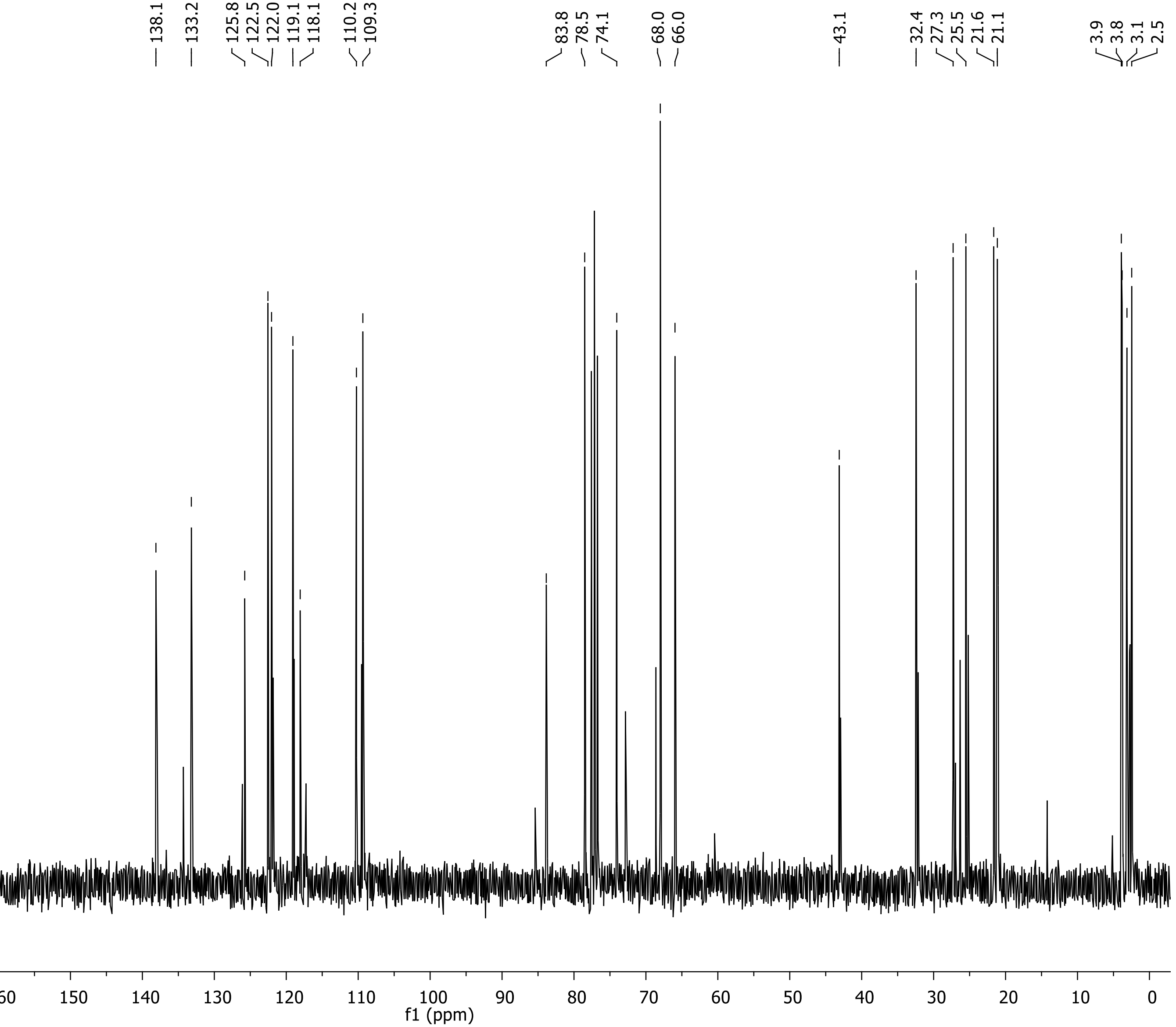

DEPT ($CDCl_3$, 75.4 MHz)

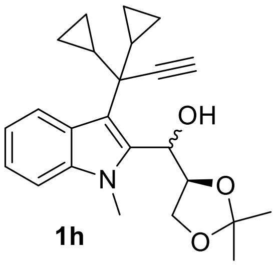

1h

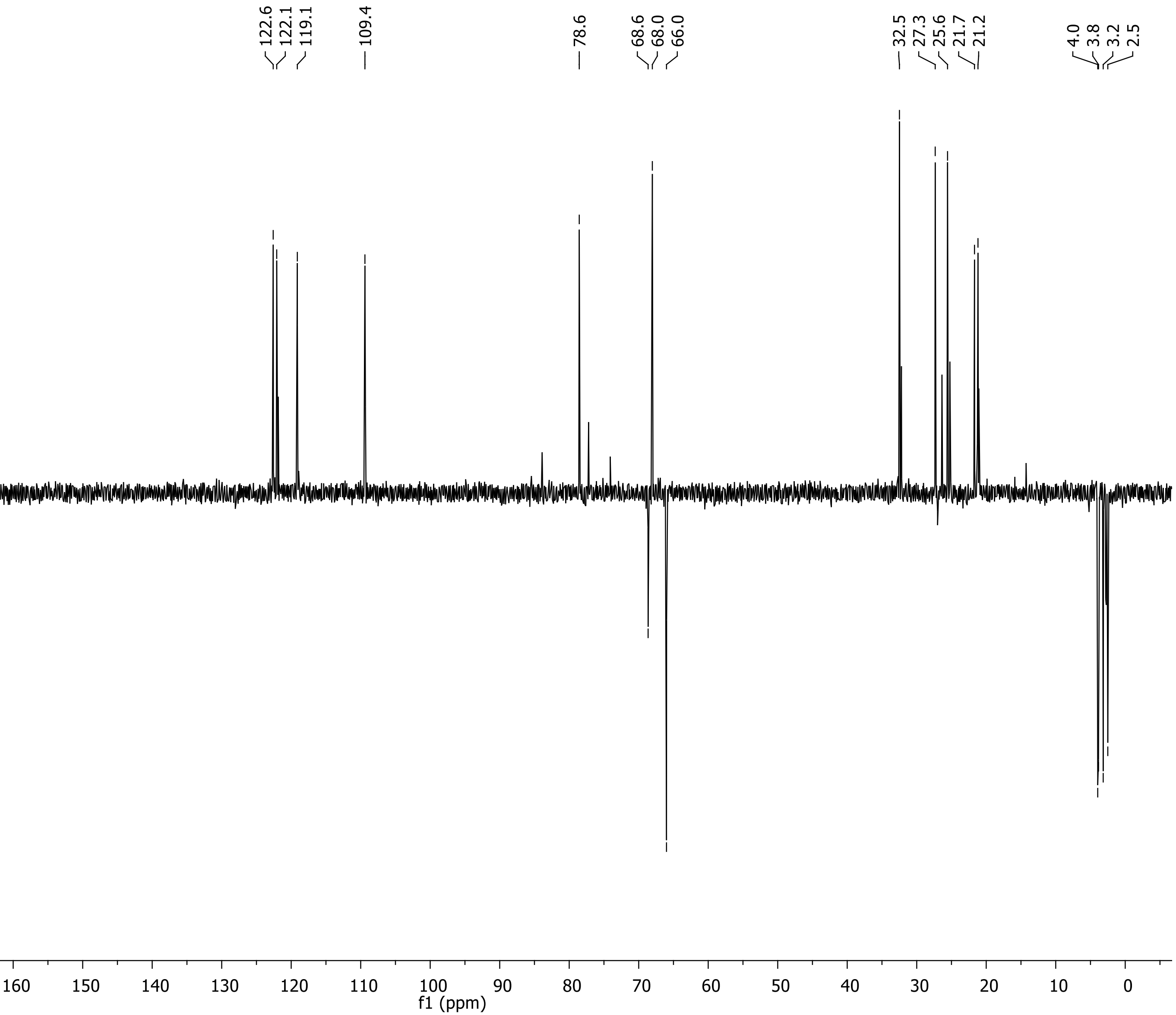

^{1}H NMR ($CDCl_3$, 300 MHz)

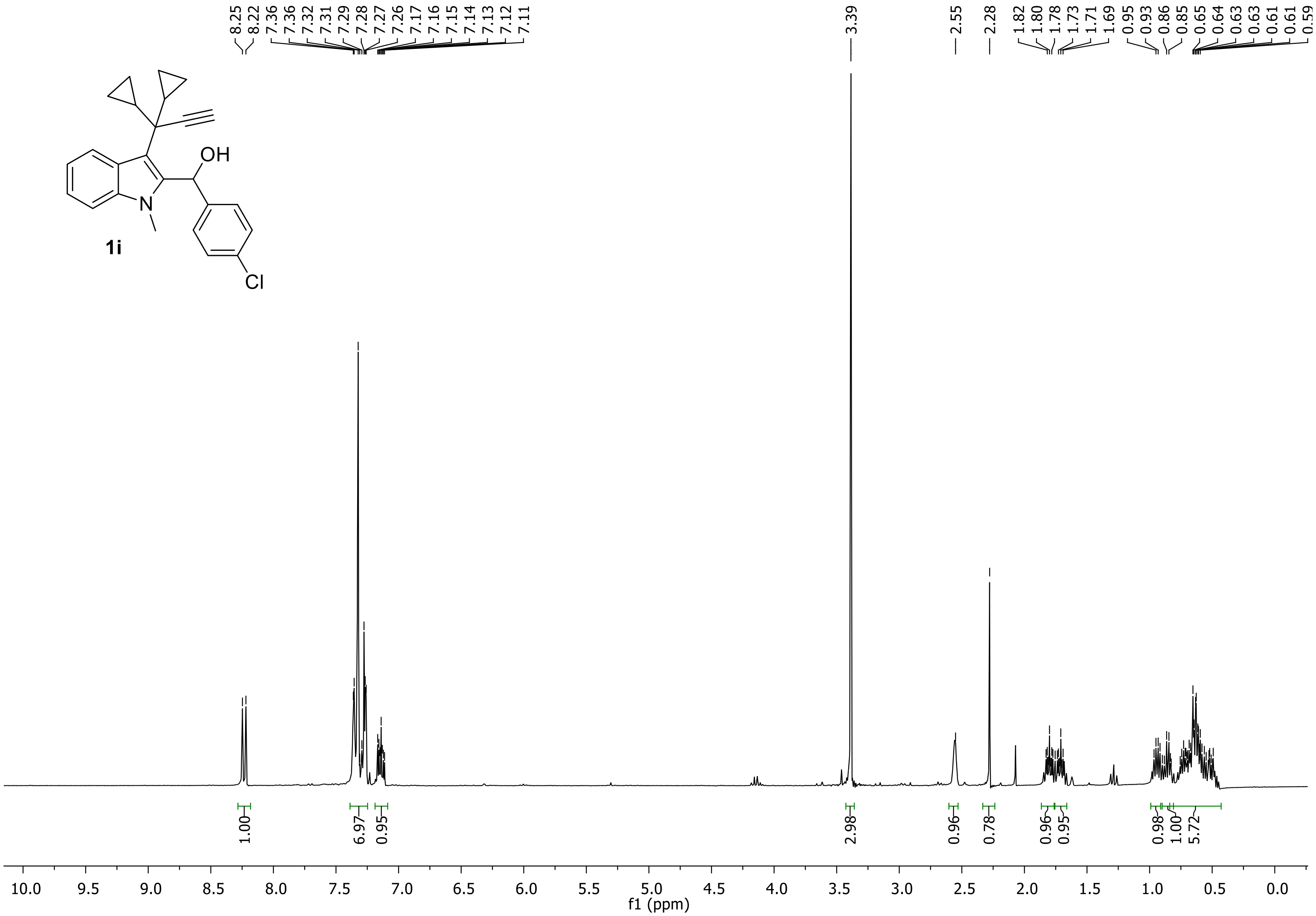

^{13}C NMR ($CDCl_3$, 75.4 MHz)

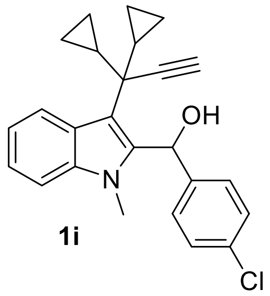

1i

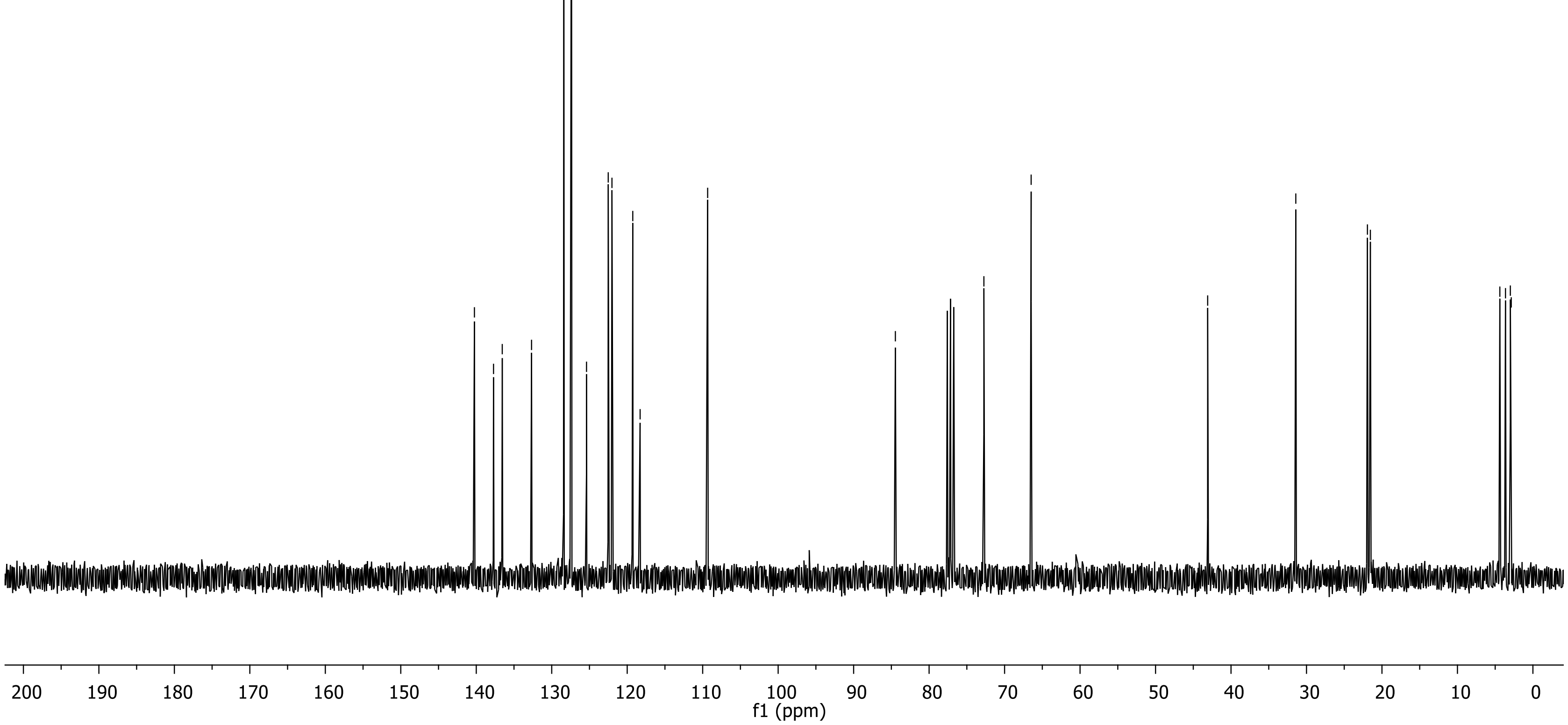

DEPT ($CDCl_3$, 75.4 MHz)

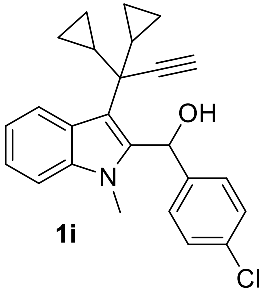

1i

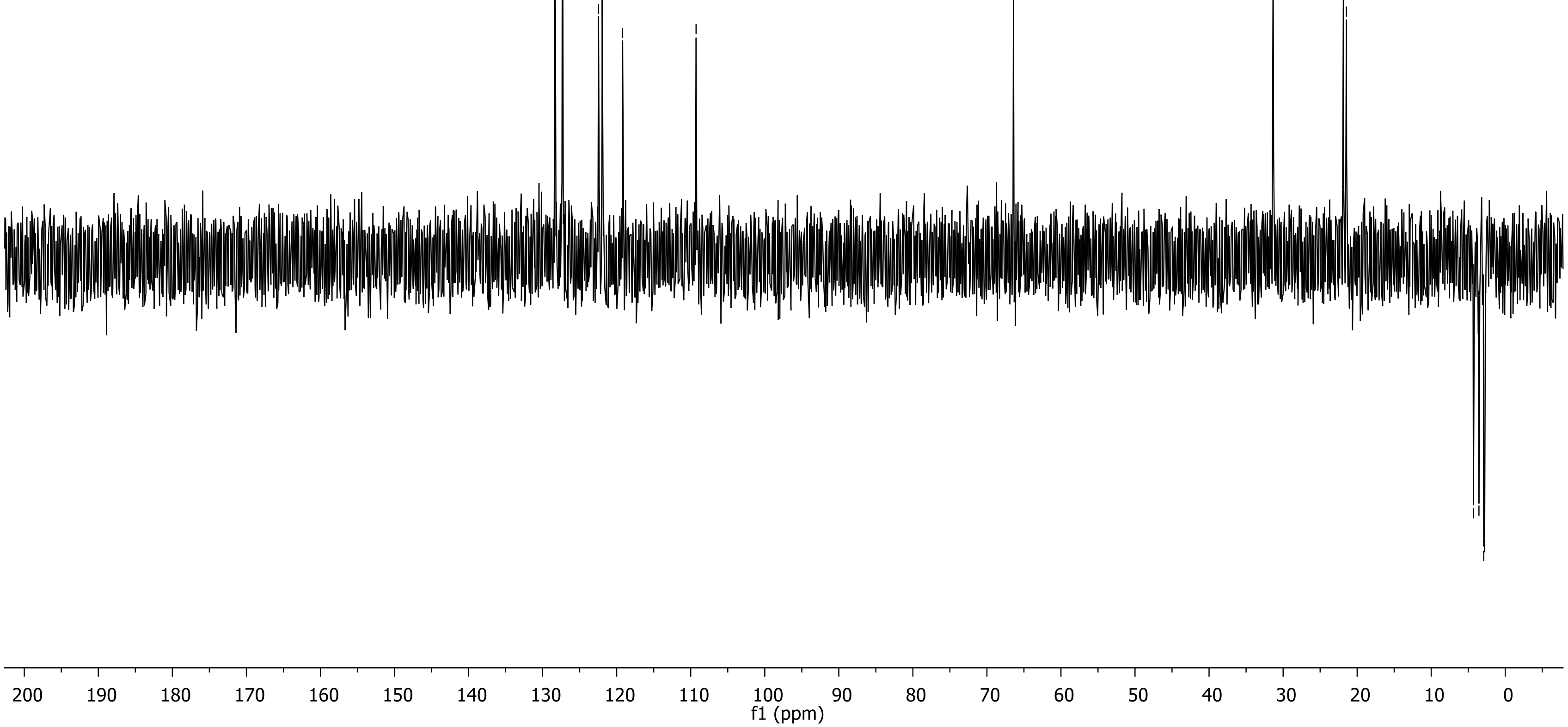

^{1}H NMR ($CDCl_3$, 300 MHz)

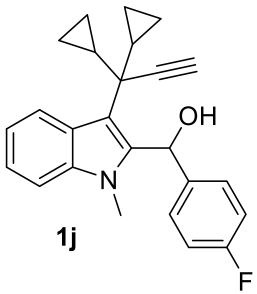

1j

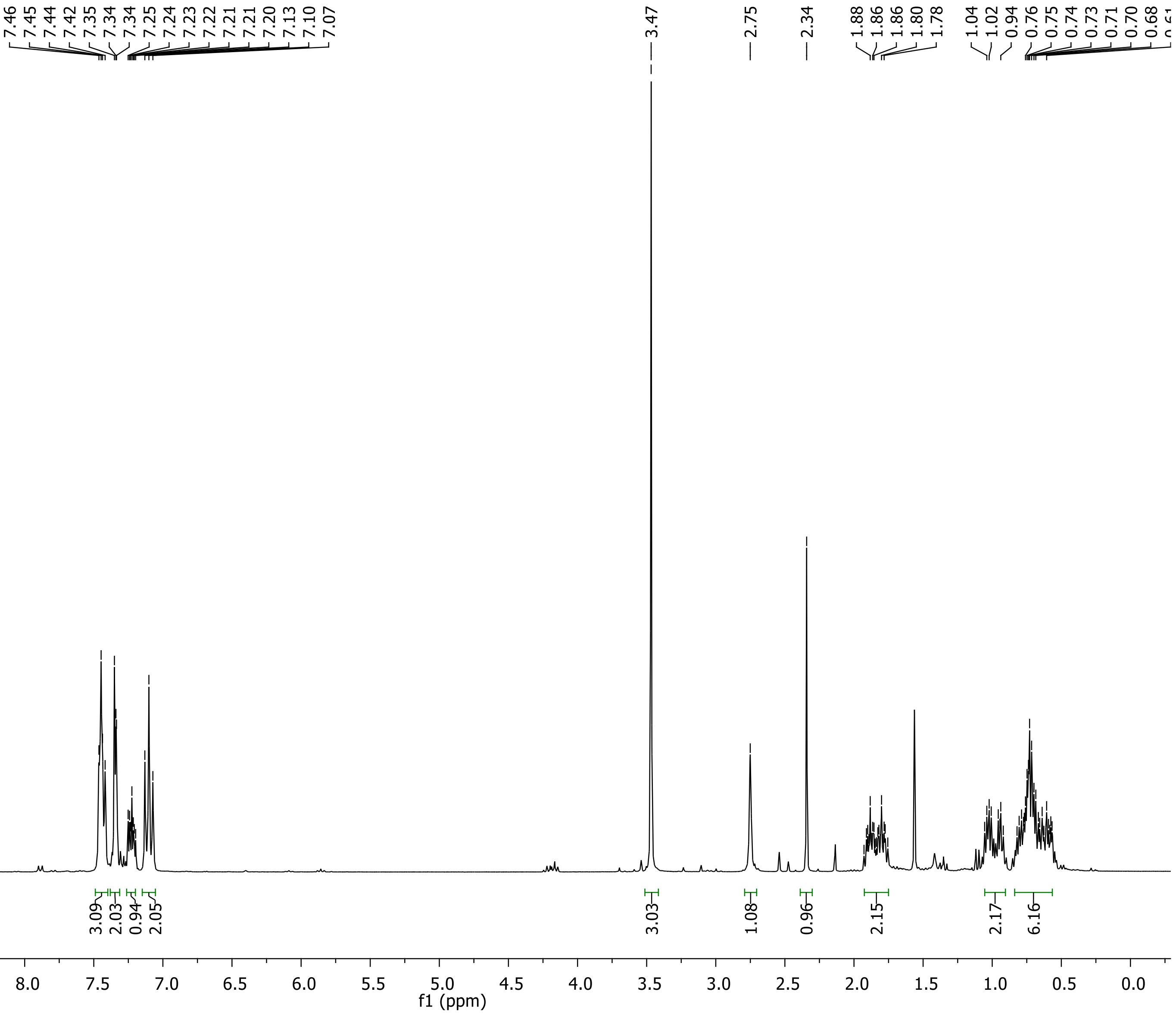

^{13}C NMR ($CDCl_3$, 75.4 MHz)

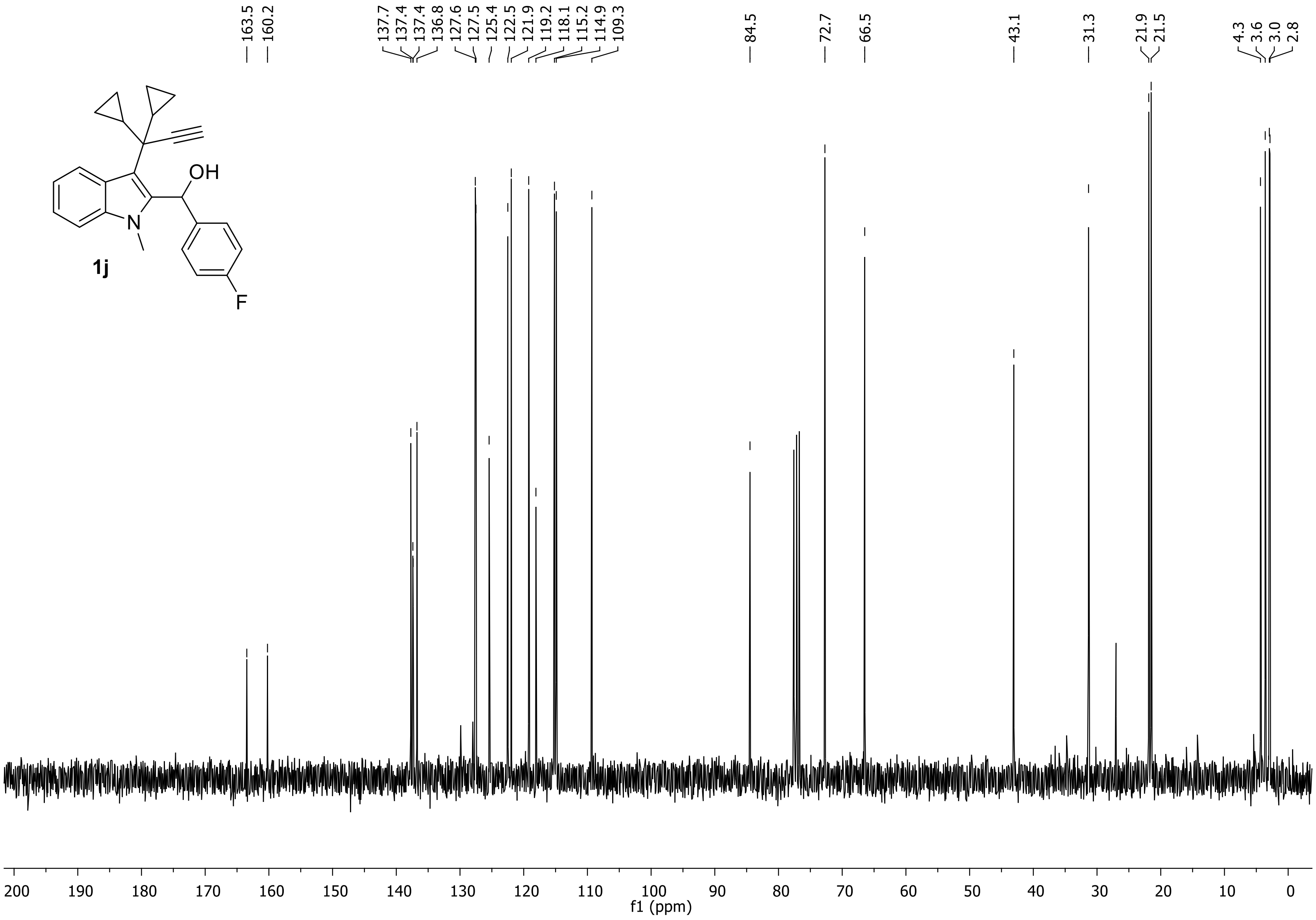

DEPT ($CDCl_3$, 75.4 MHz)

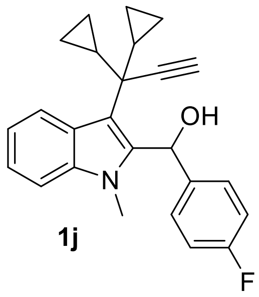

1j

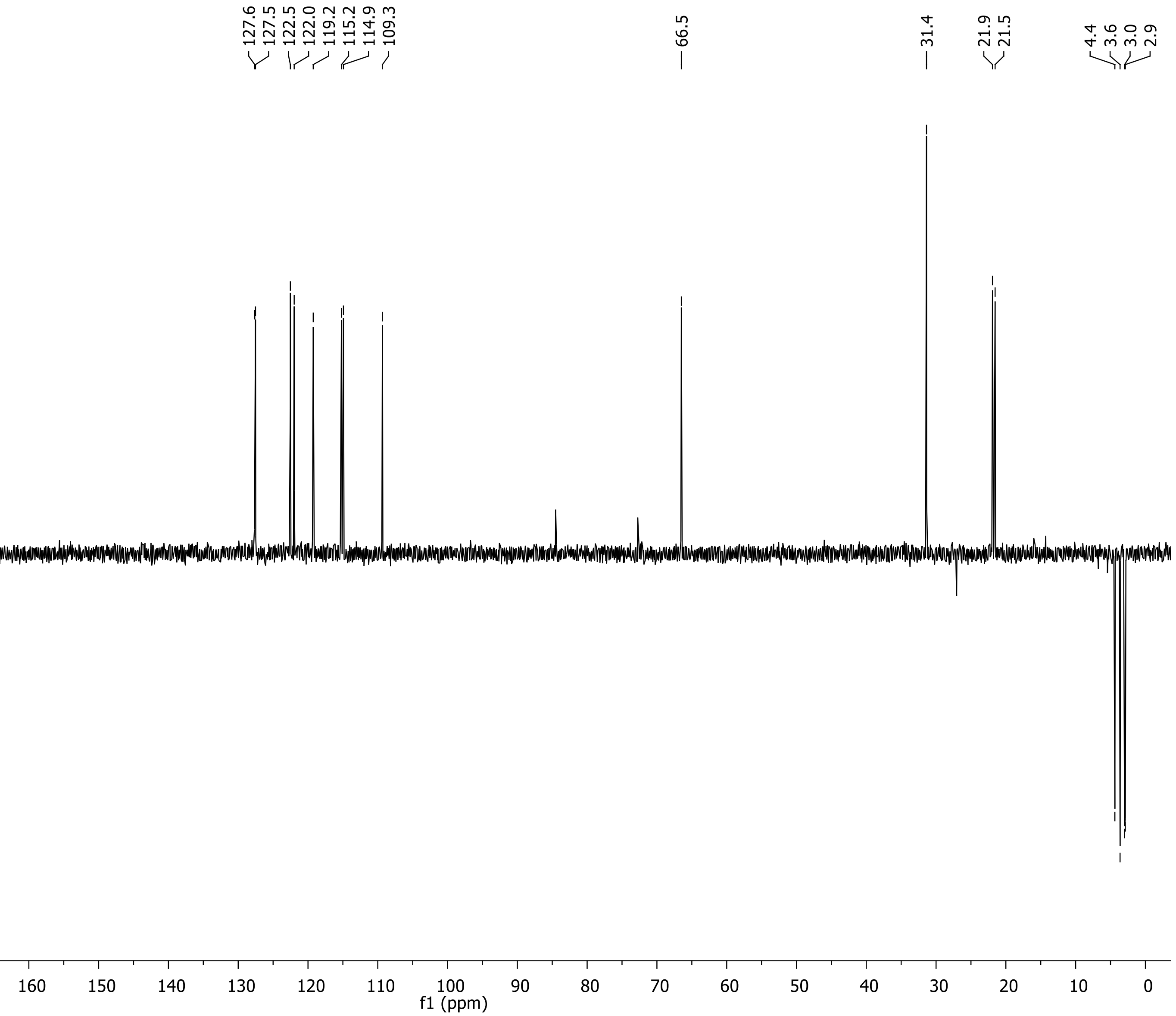

^{1}H NMR ($CDCl_3$, 300 MHz)

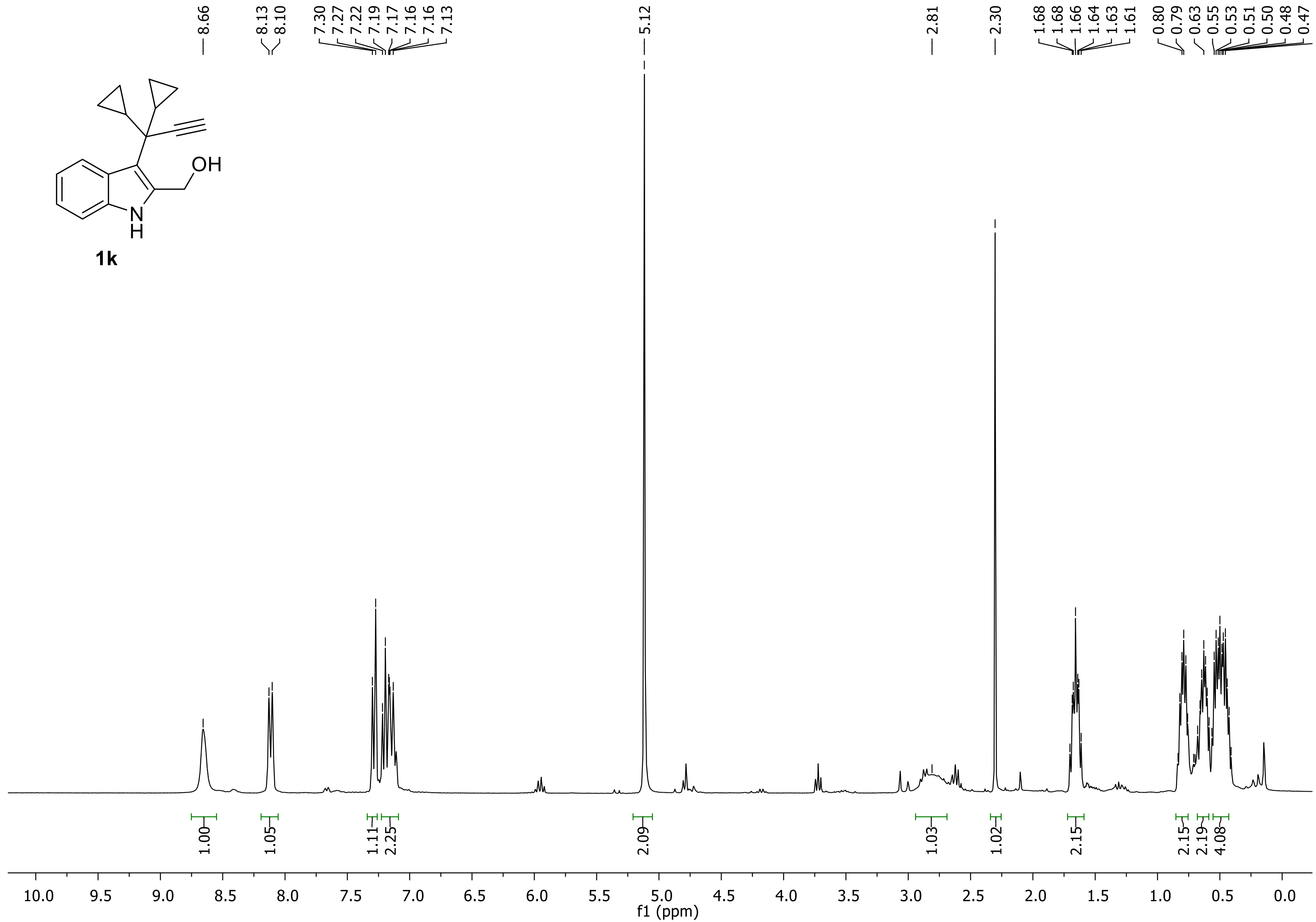

^{13}C NMR ($CDCl_3$, 75.4 MHz)

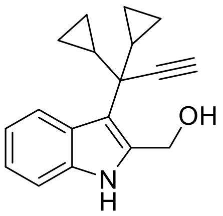

1k

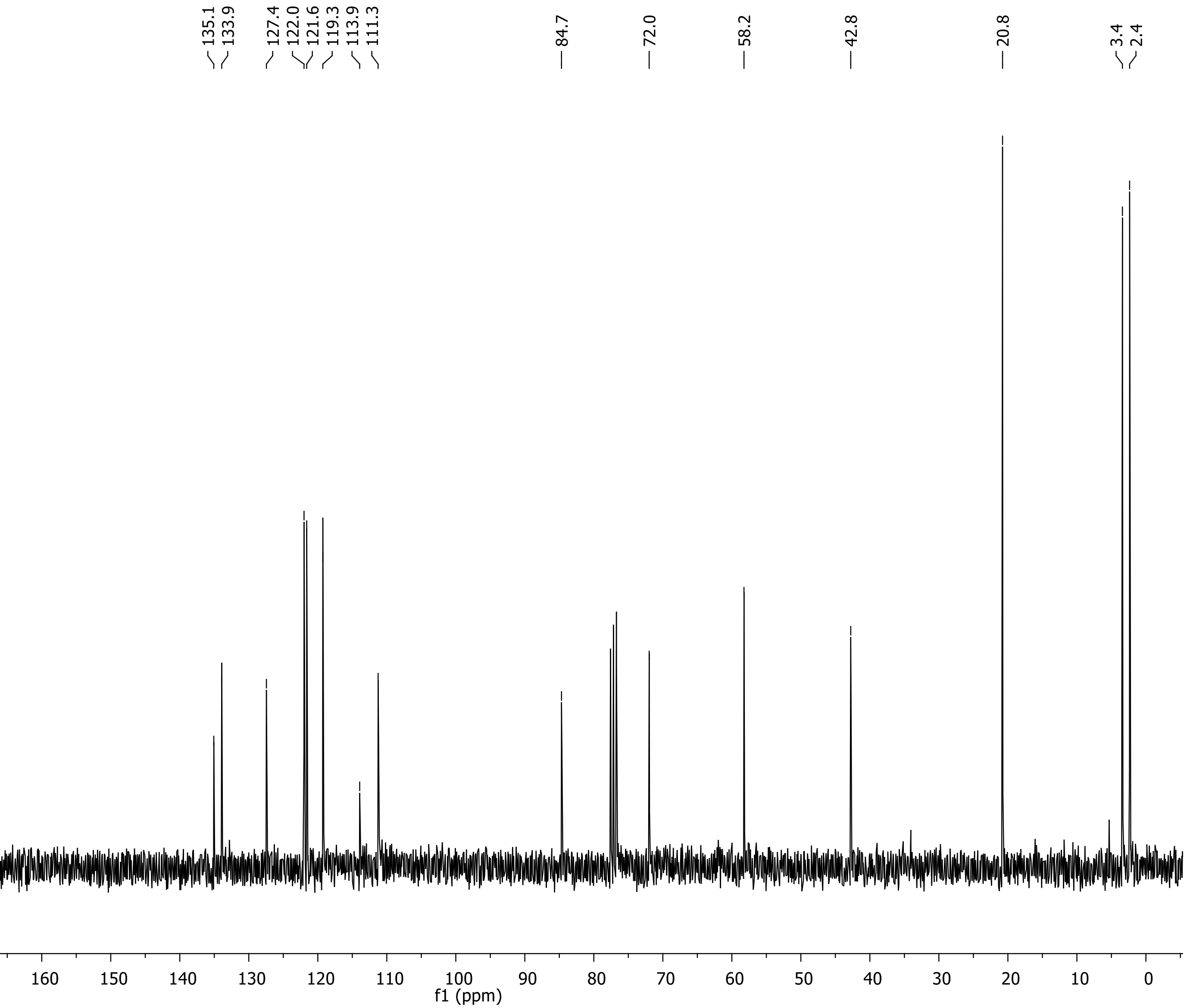

DEPT ($CDCl_3$, 75.4 MHz)

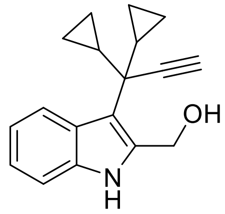

1k

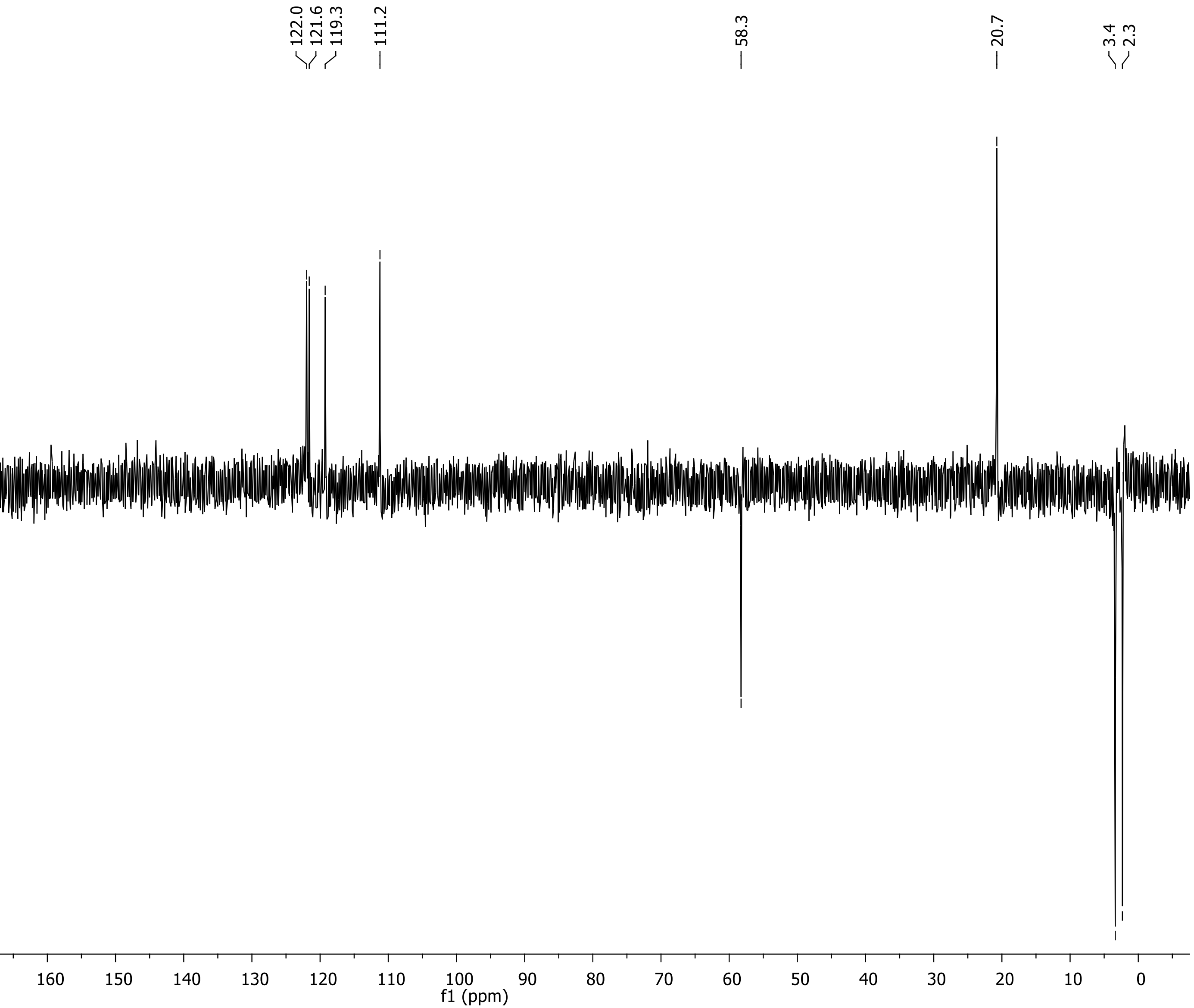

^1H NMR ($CDCl_3$, 300 MHz)

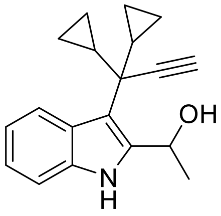

1l

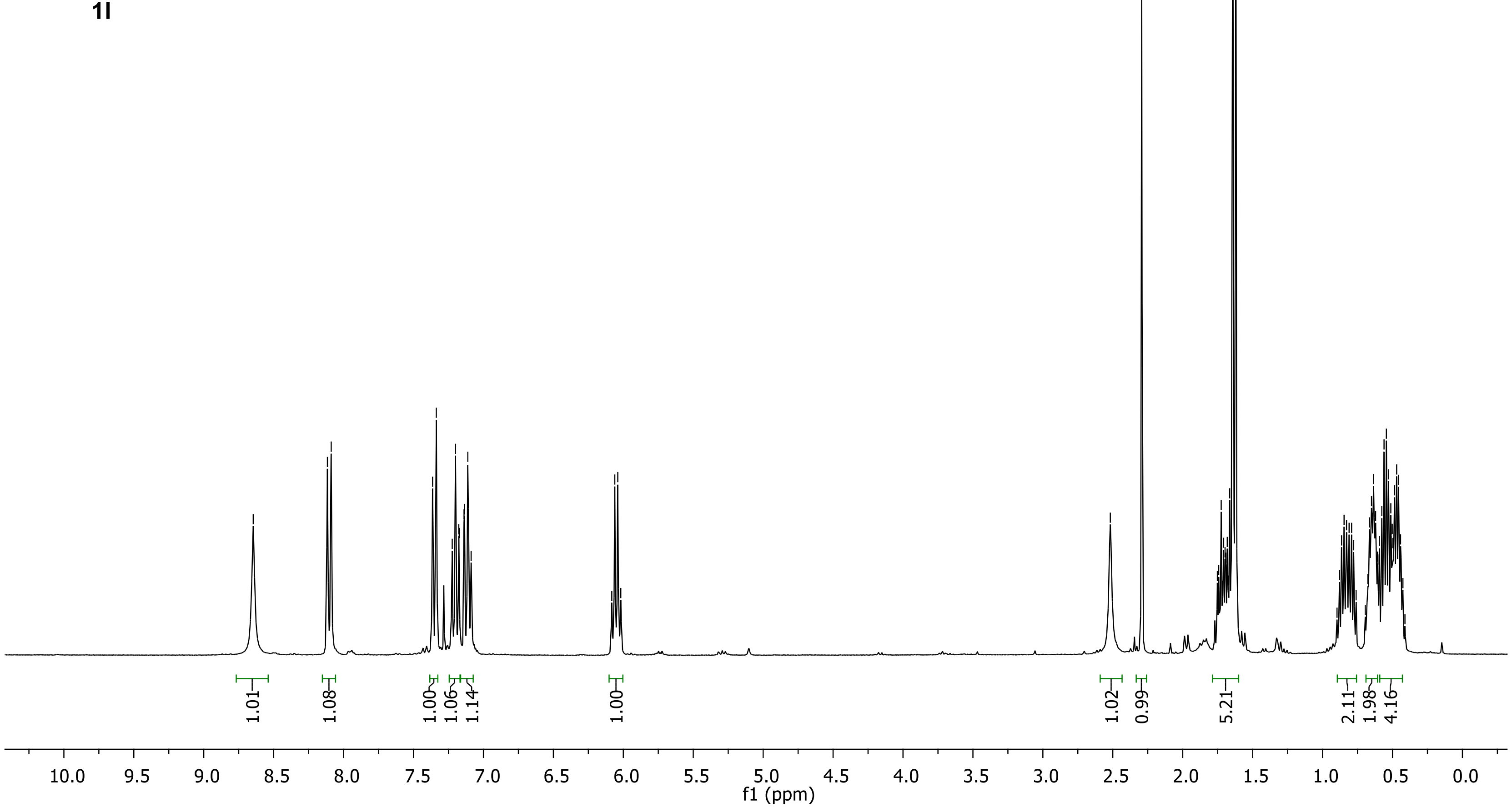

^{13}C NMR ($CDCl_3$, 75.4 MHz)

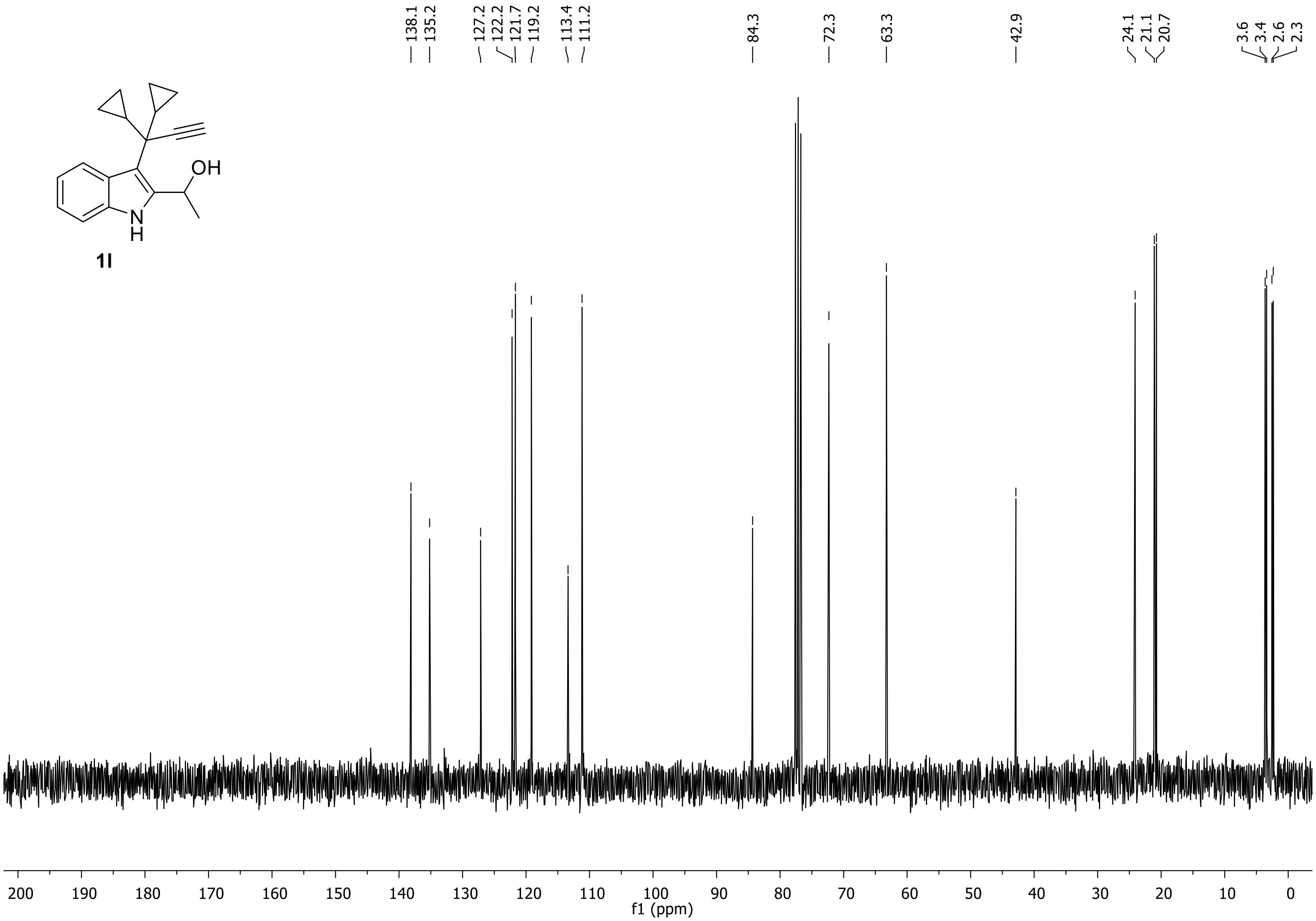

1l

DEPT ($CDCl_3$, 75.4 MHz)

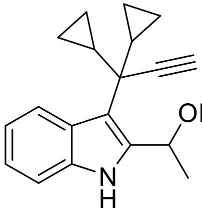

1l

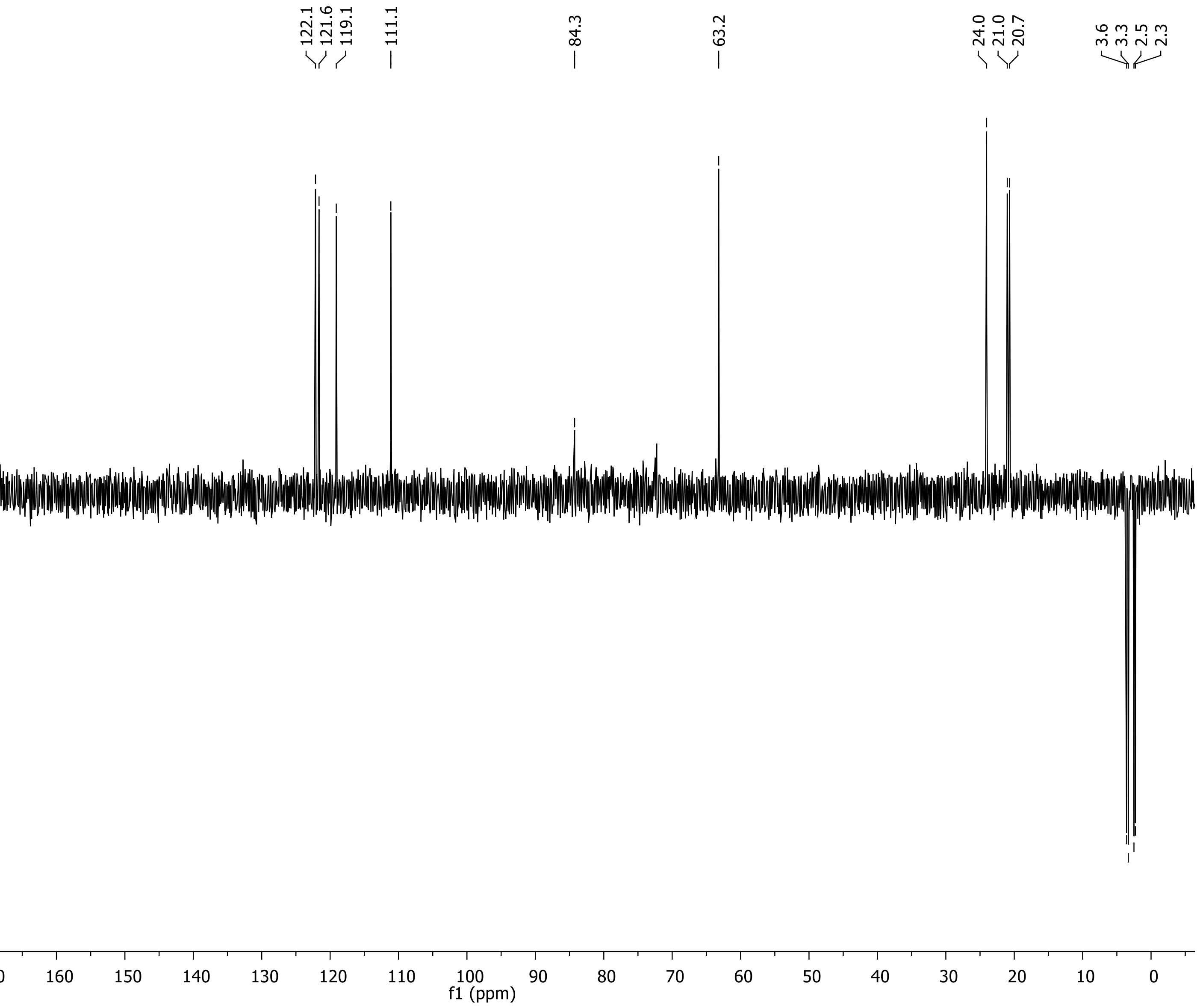

^{1}H NMR ($CDCl_3$, 300 MHz)

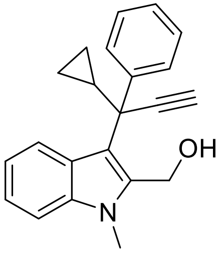

1m

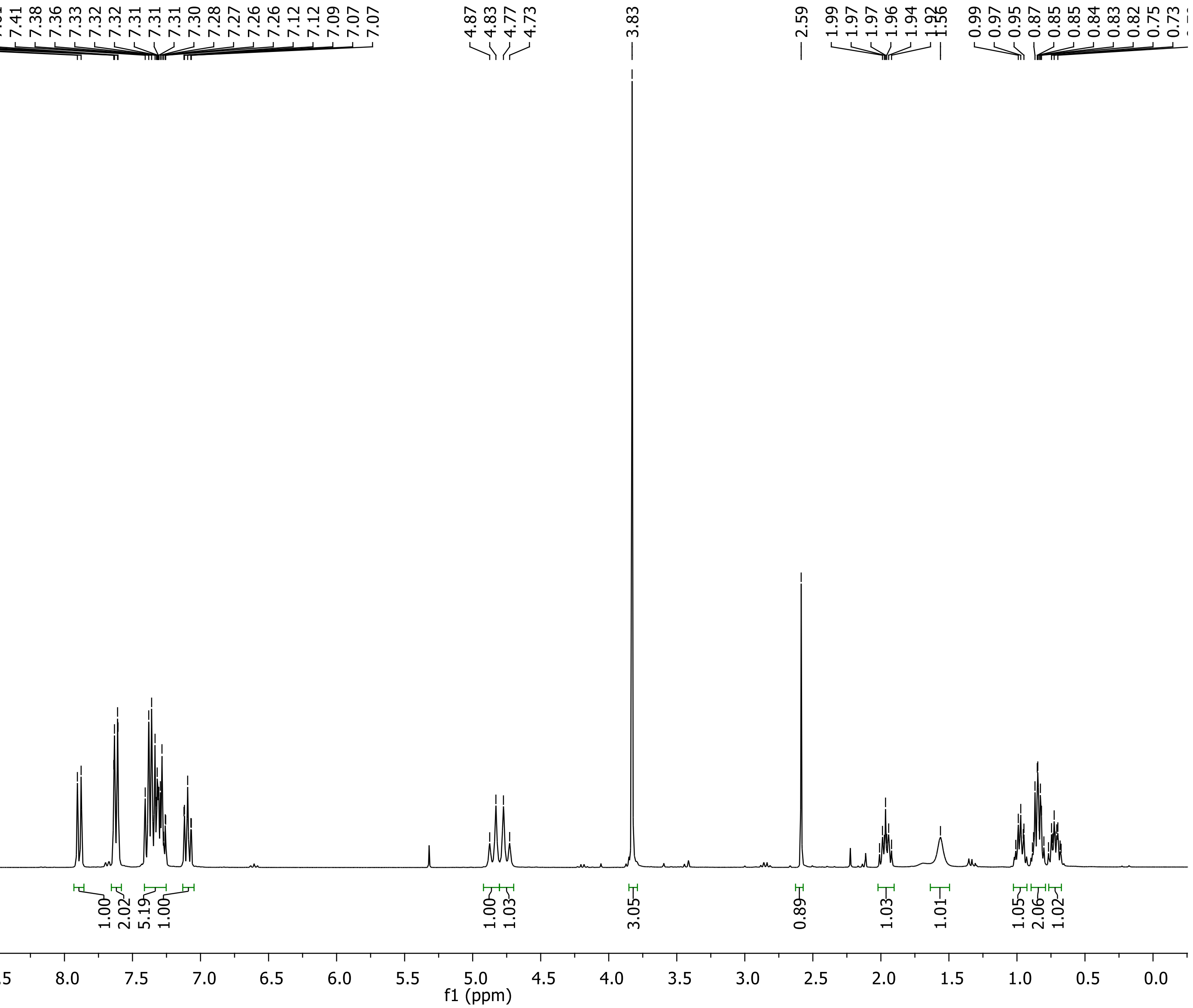

^{13}C NMR ($CDCl_3$, 75.4 MHz)

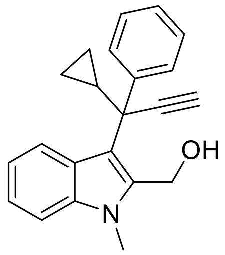

1m

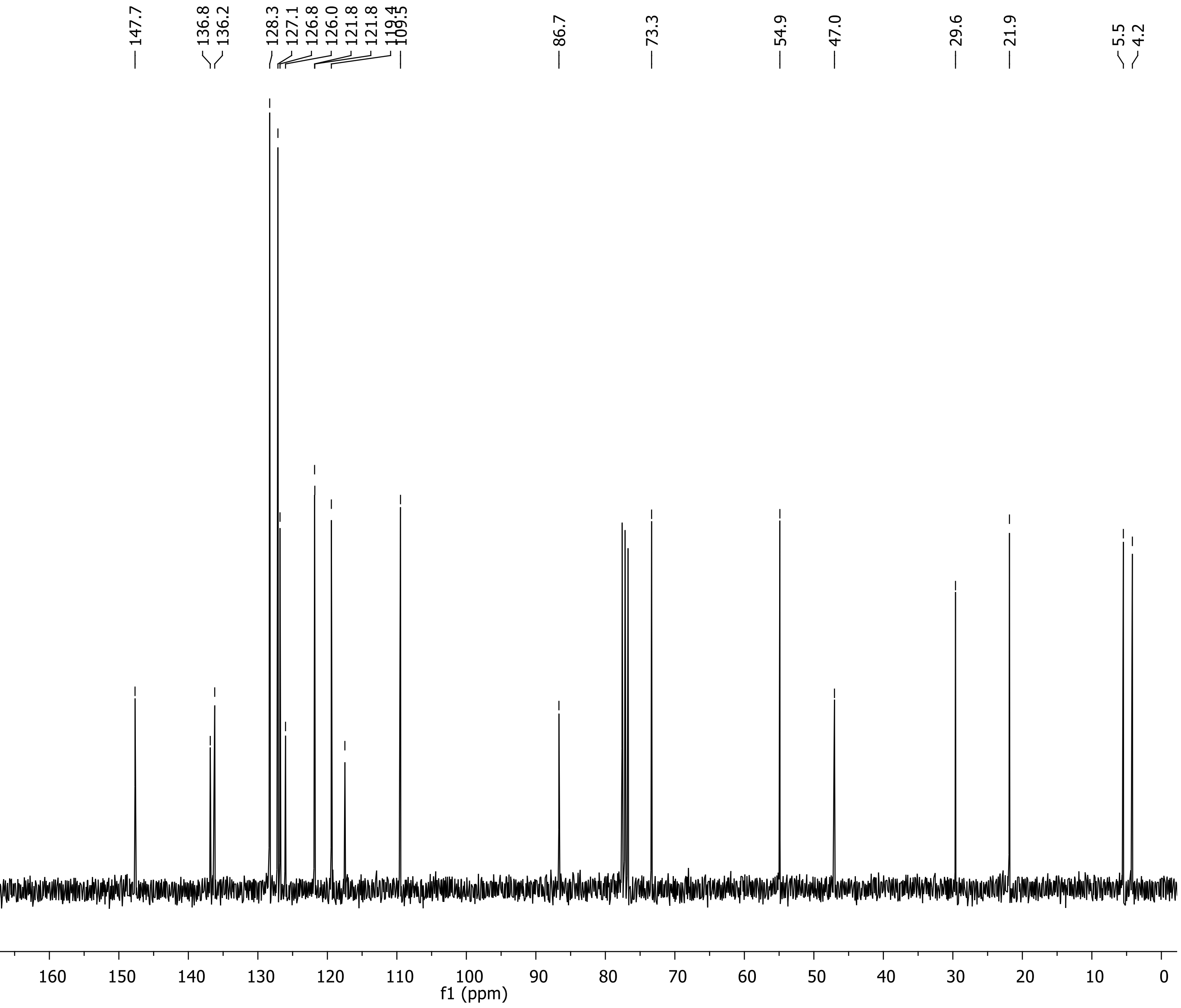

DEPT ($CDCl_3$, 75.4 MHz)

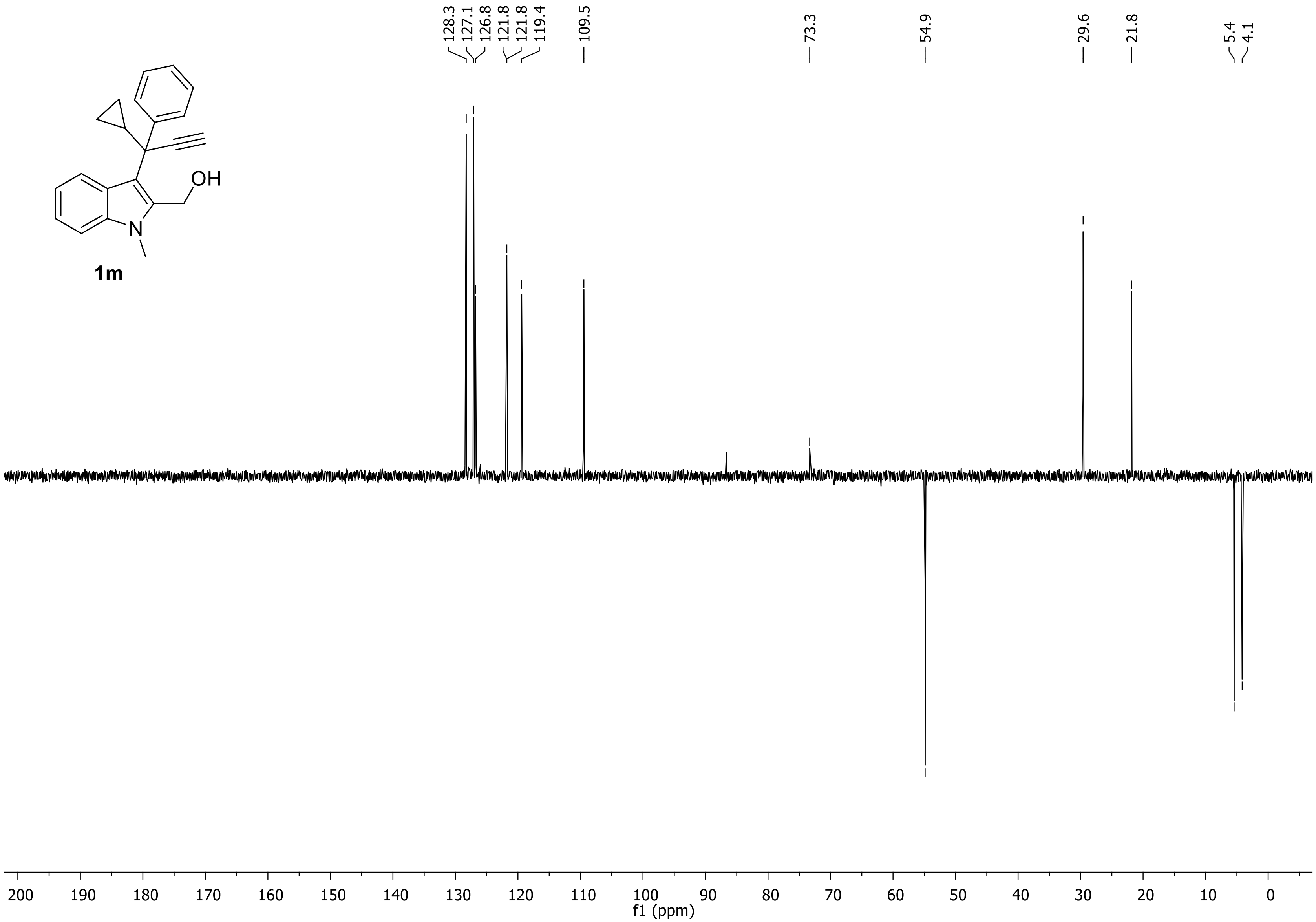

^{1}H NMR ($CDCl_3$, 300 MHz)

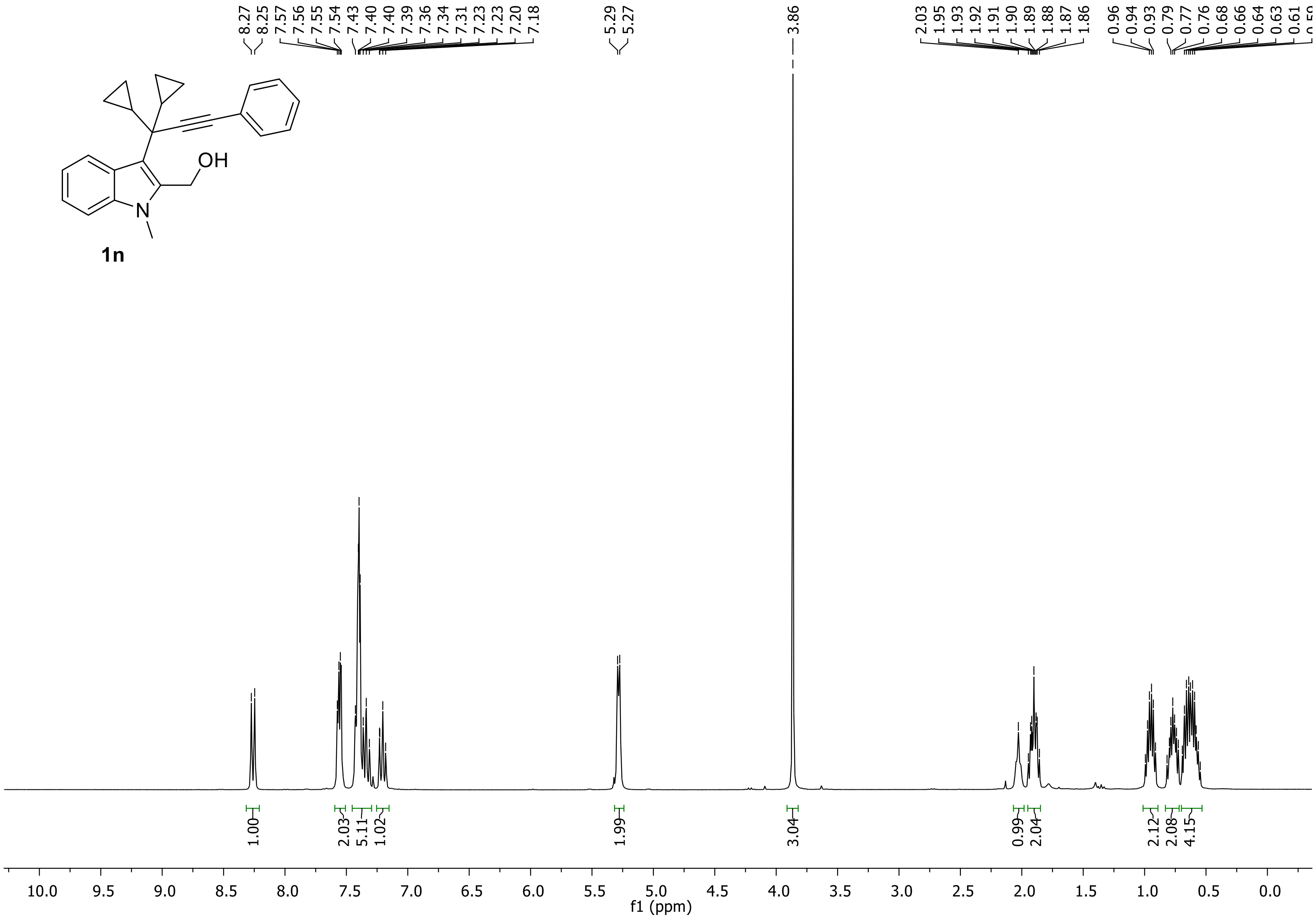

^{13}C NMR ($CDCl_3$, 75.4 MHz)

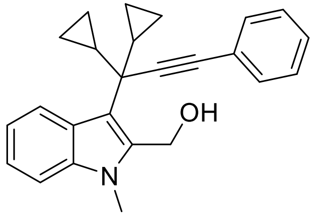

1n

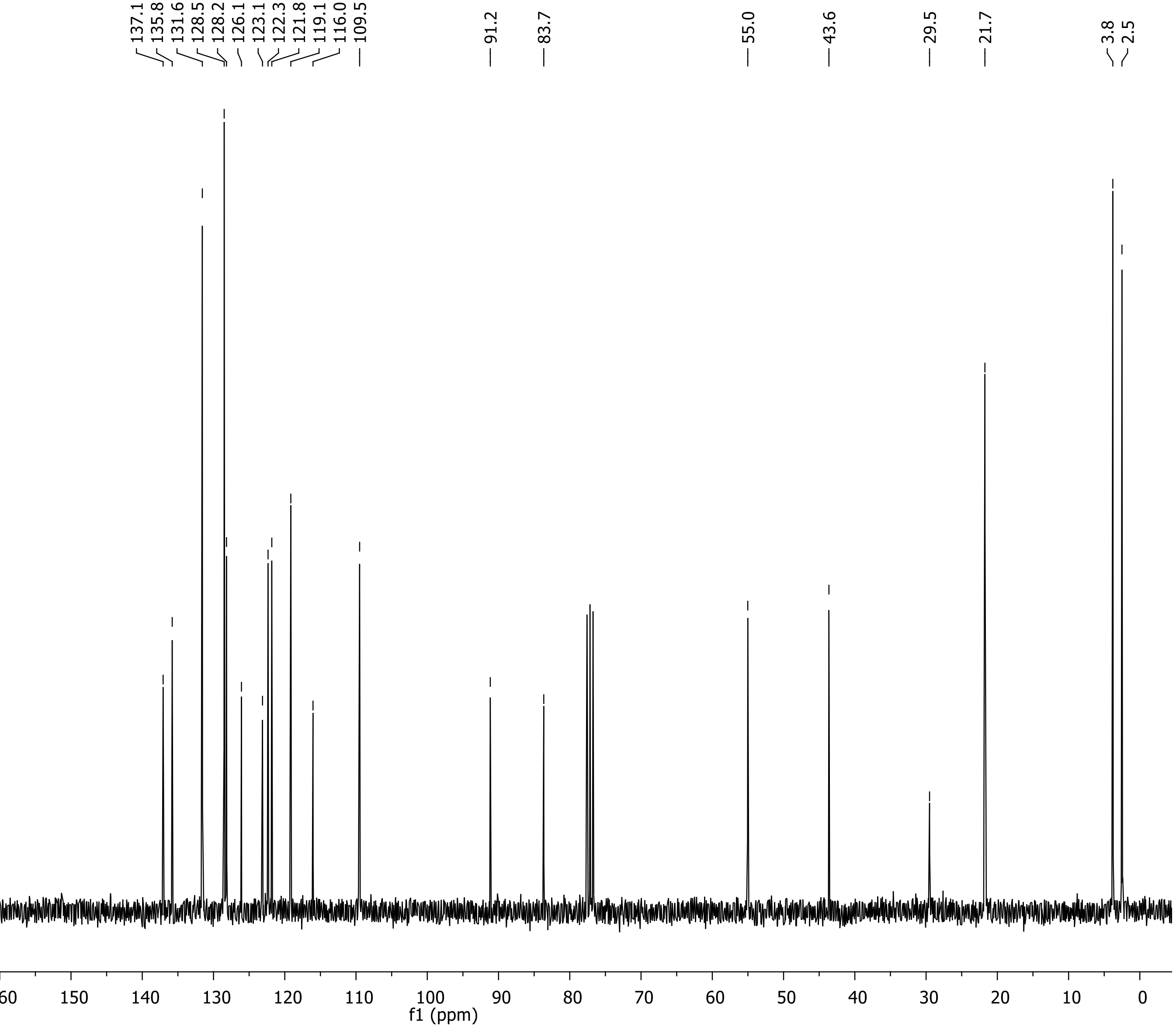

DEPT ($CDCl_3$, 75.4 MHz)

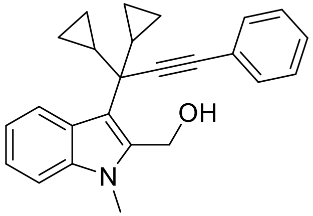

1n

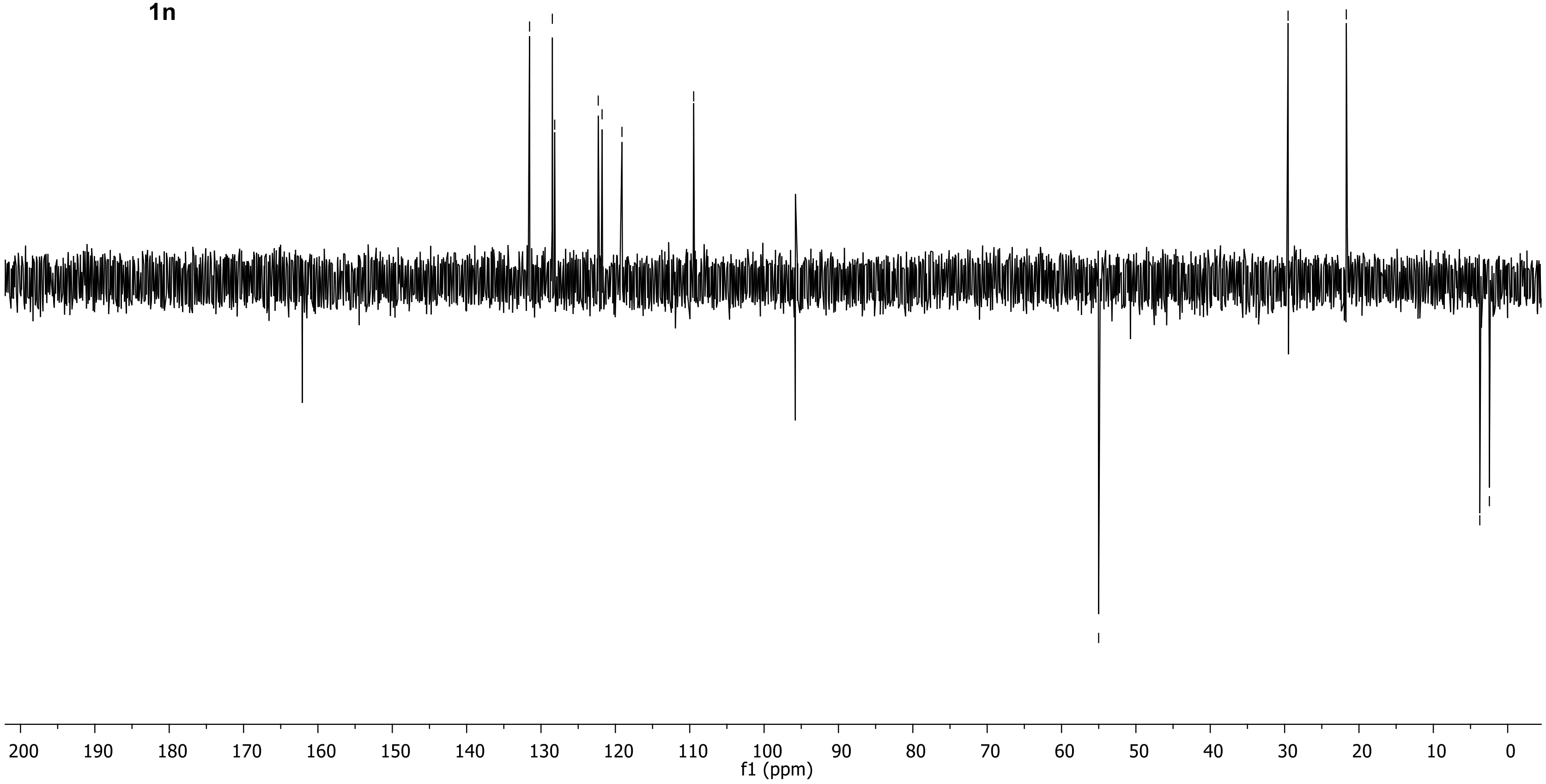

^{1}H NMR ($CDCl_3$, 300 MHz)

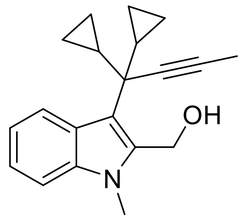

1o

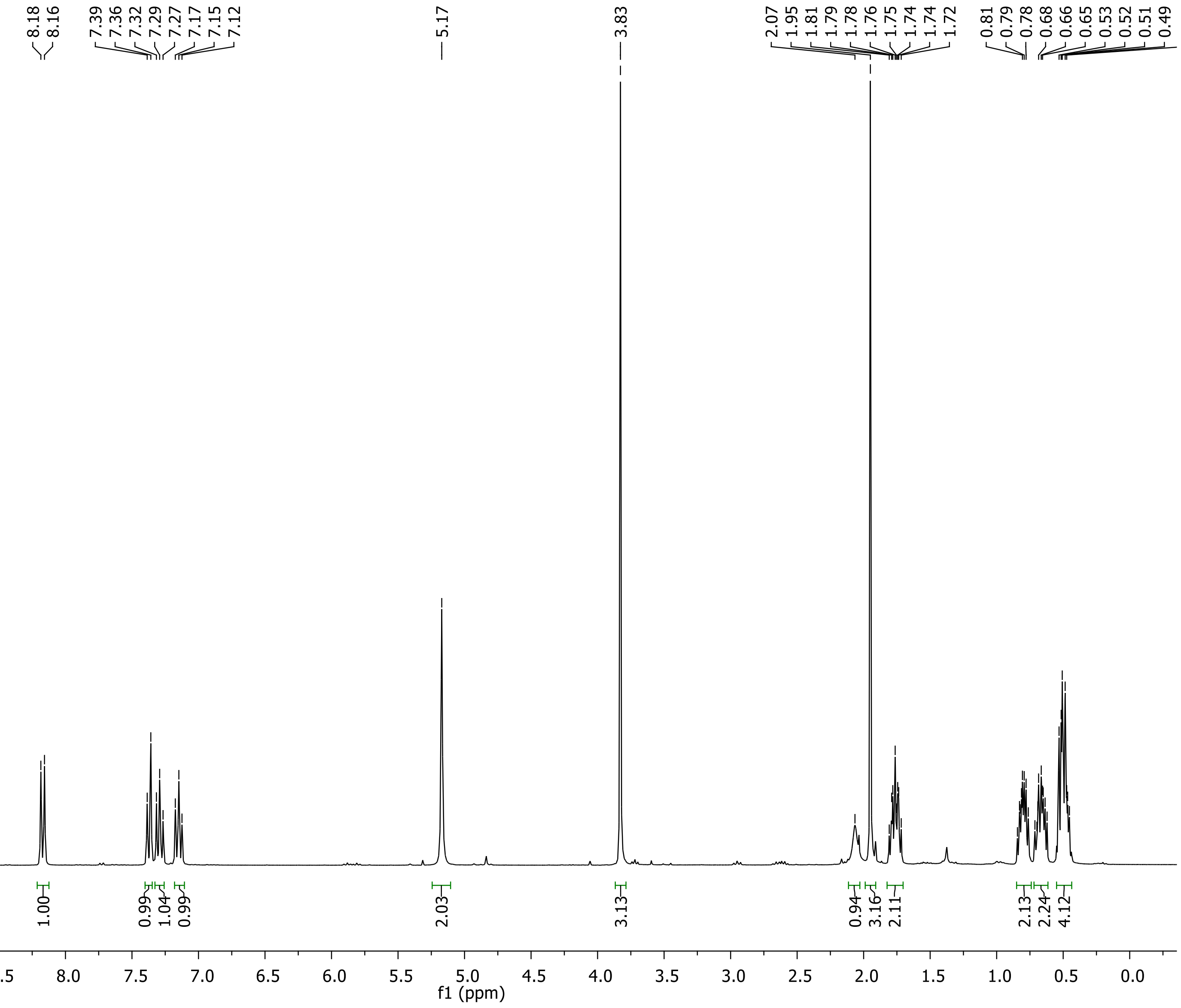

^{13}C NMR ($CDCl_3$, 75.4 MHz)

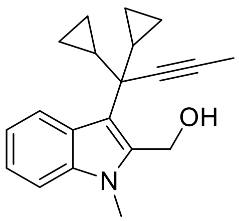

1o

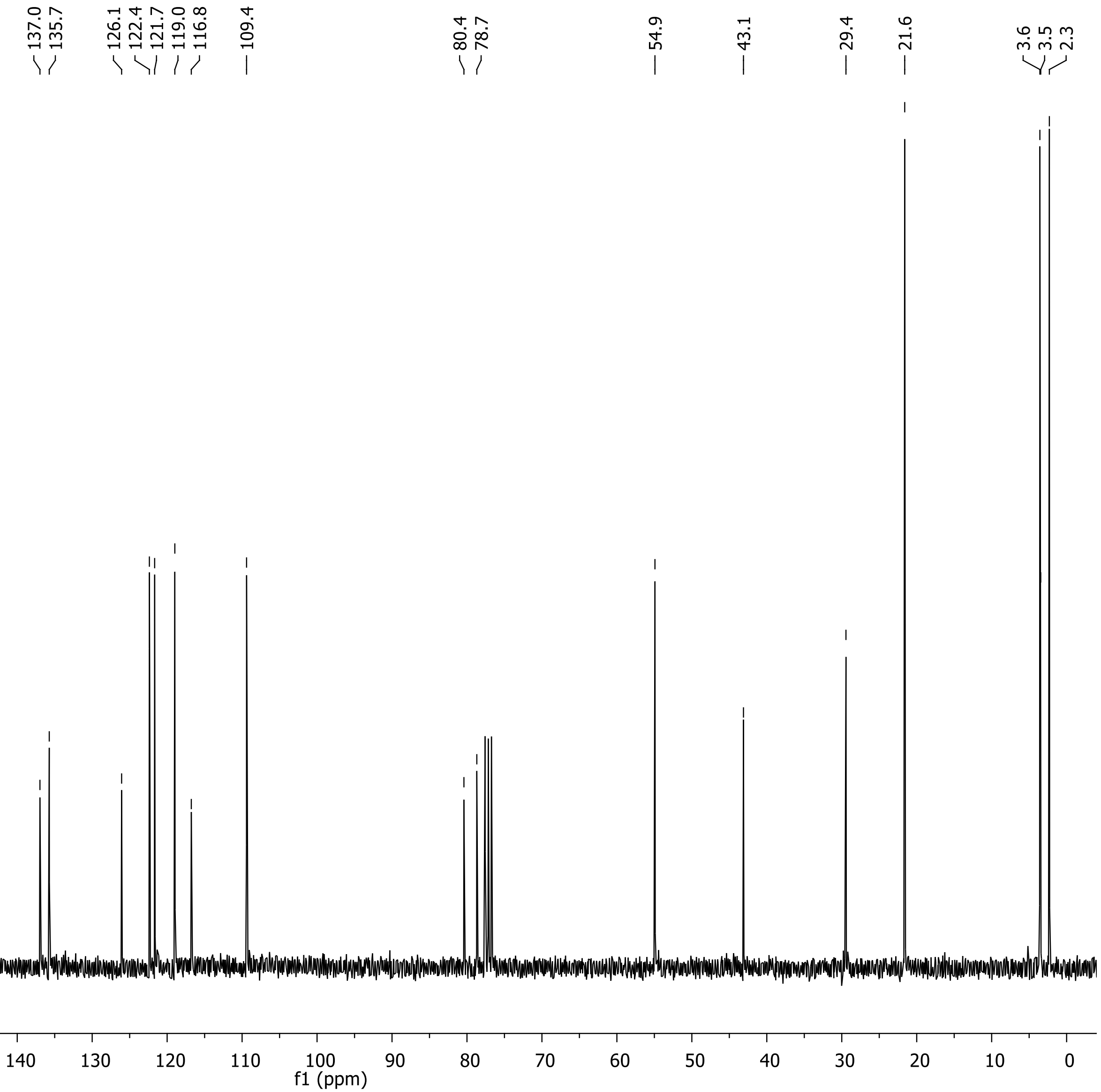

DEPT ($CDCl_3$, 75.4 MHz)

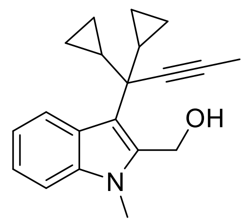

1o

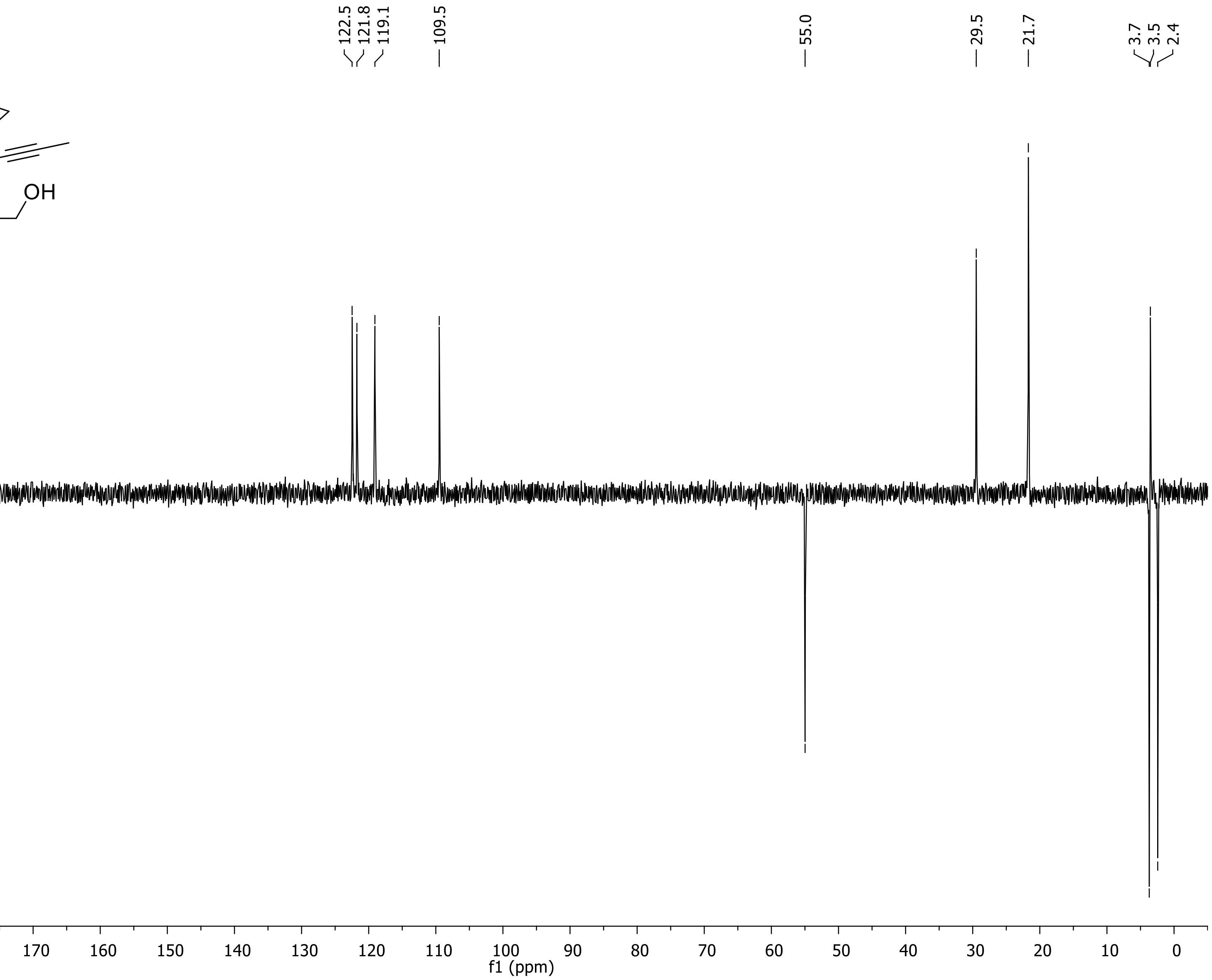

^{1}H NMR ($CDCl_3$, 300 MHz)

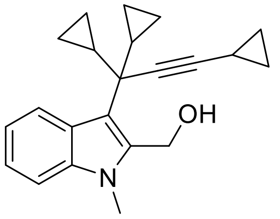

1p

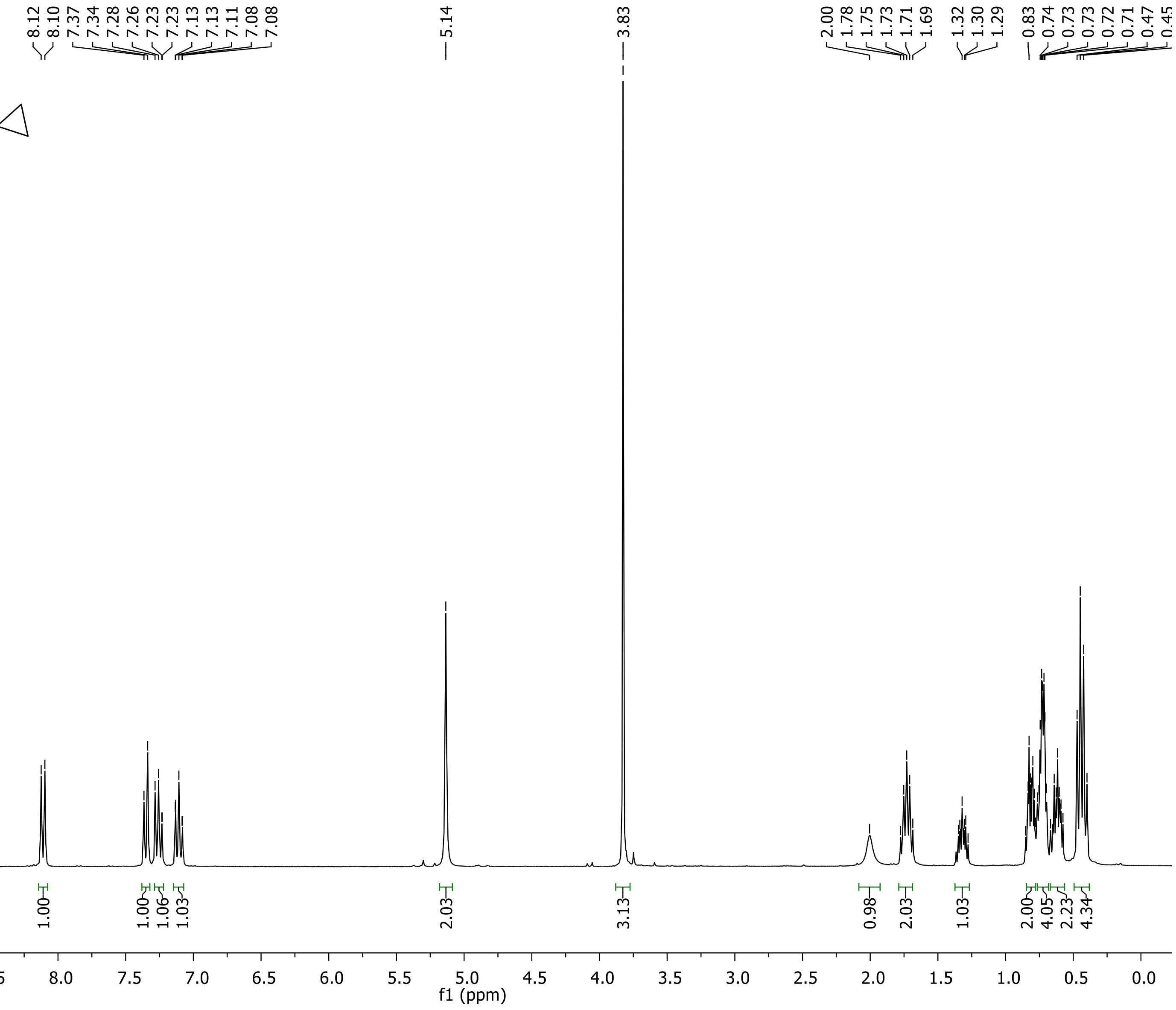

^{13}C NMR ($CDCl_3$, 75.4 MHz)

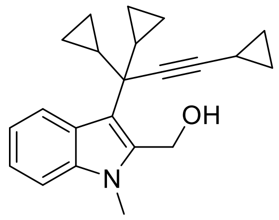

1p

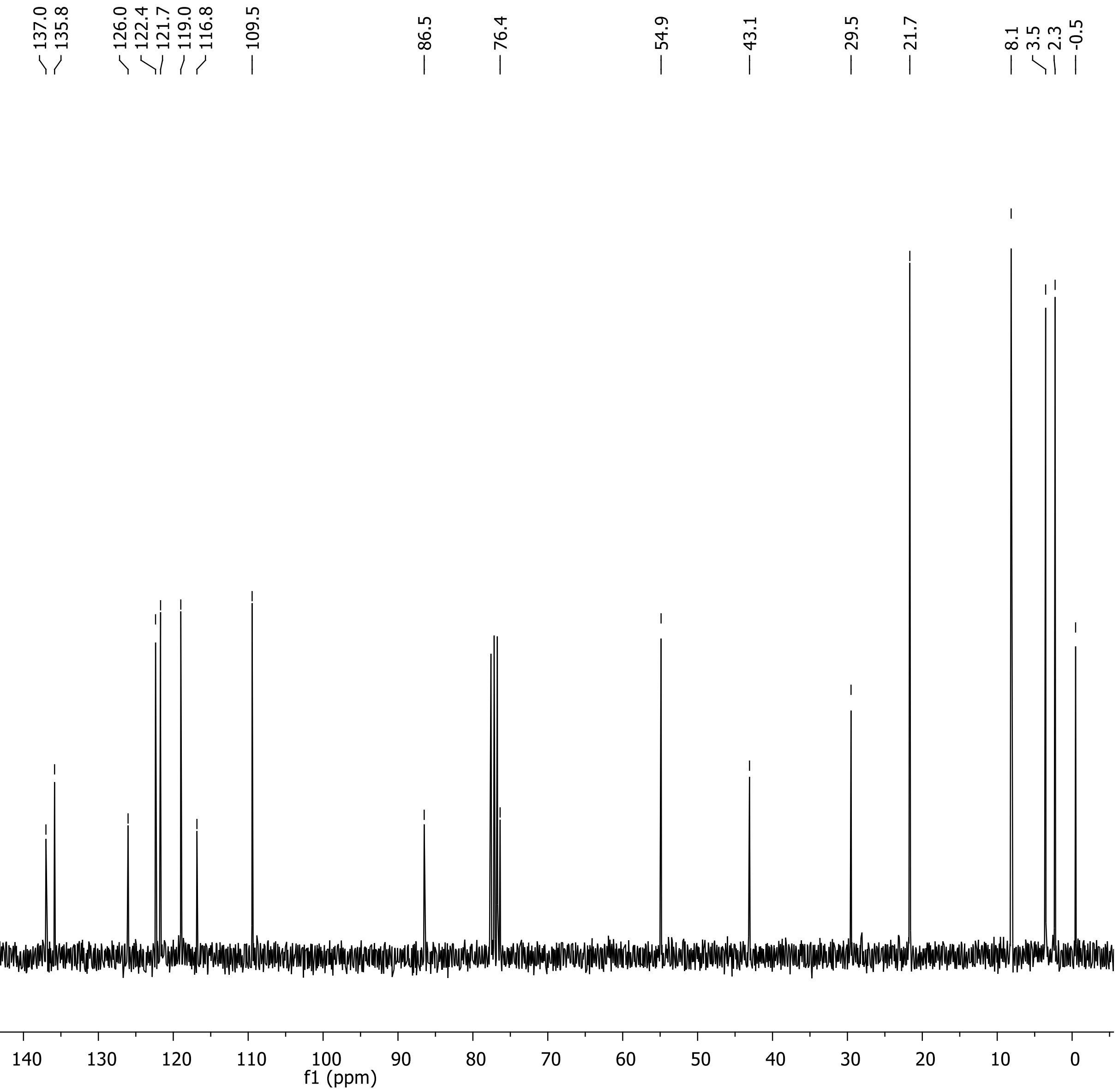

DEPT ($CDCl_3$, 75.4 MHz)

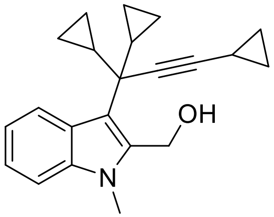

1p

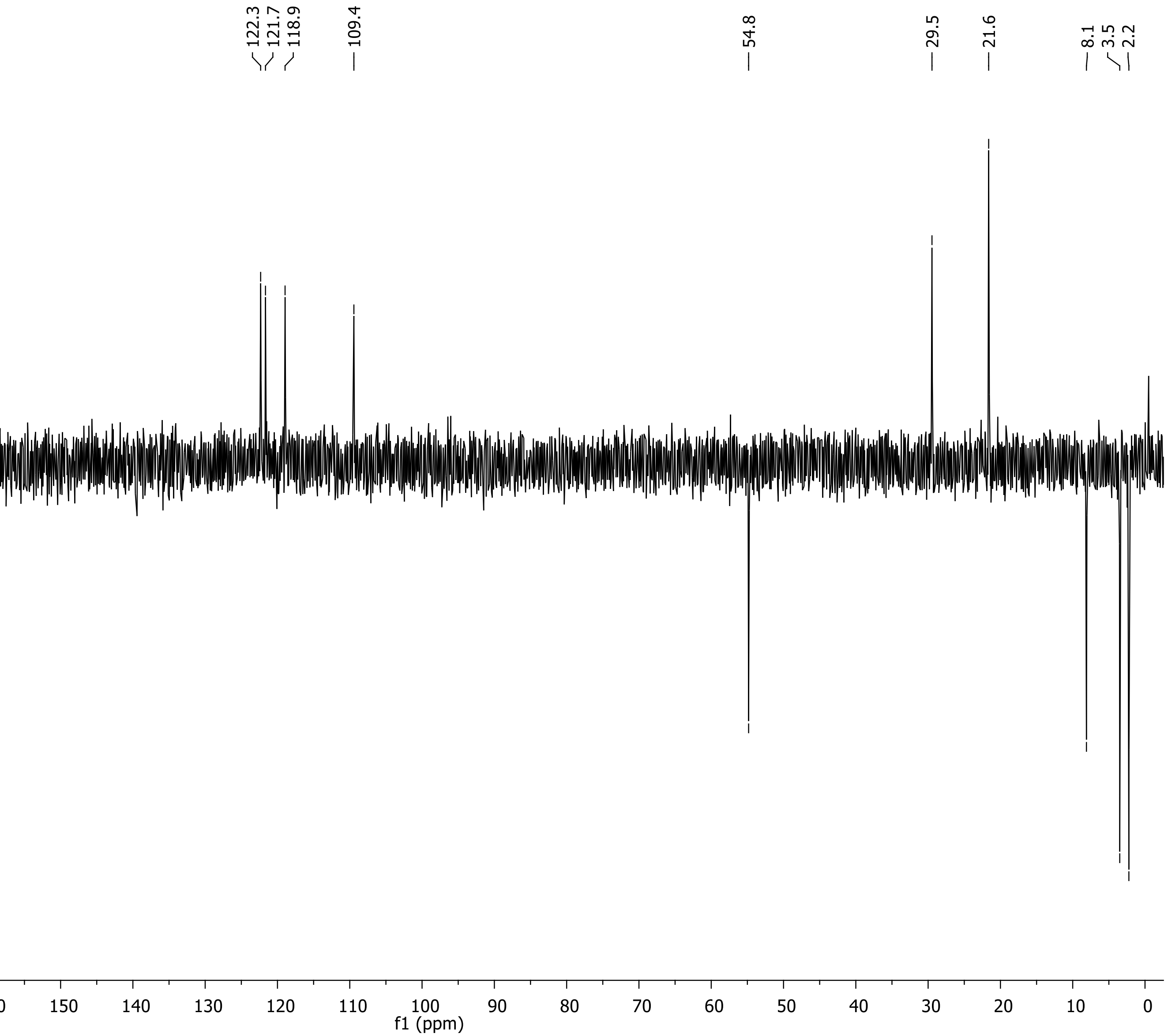

^{1}H NMR ($CDCl_3$, 300 MHz)

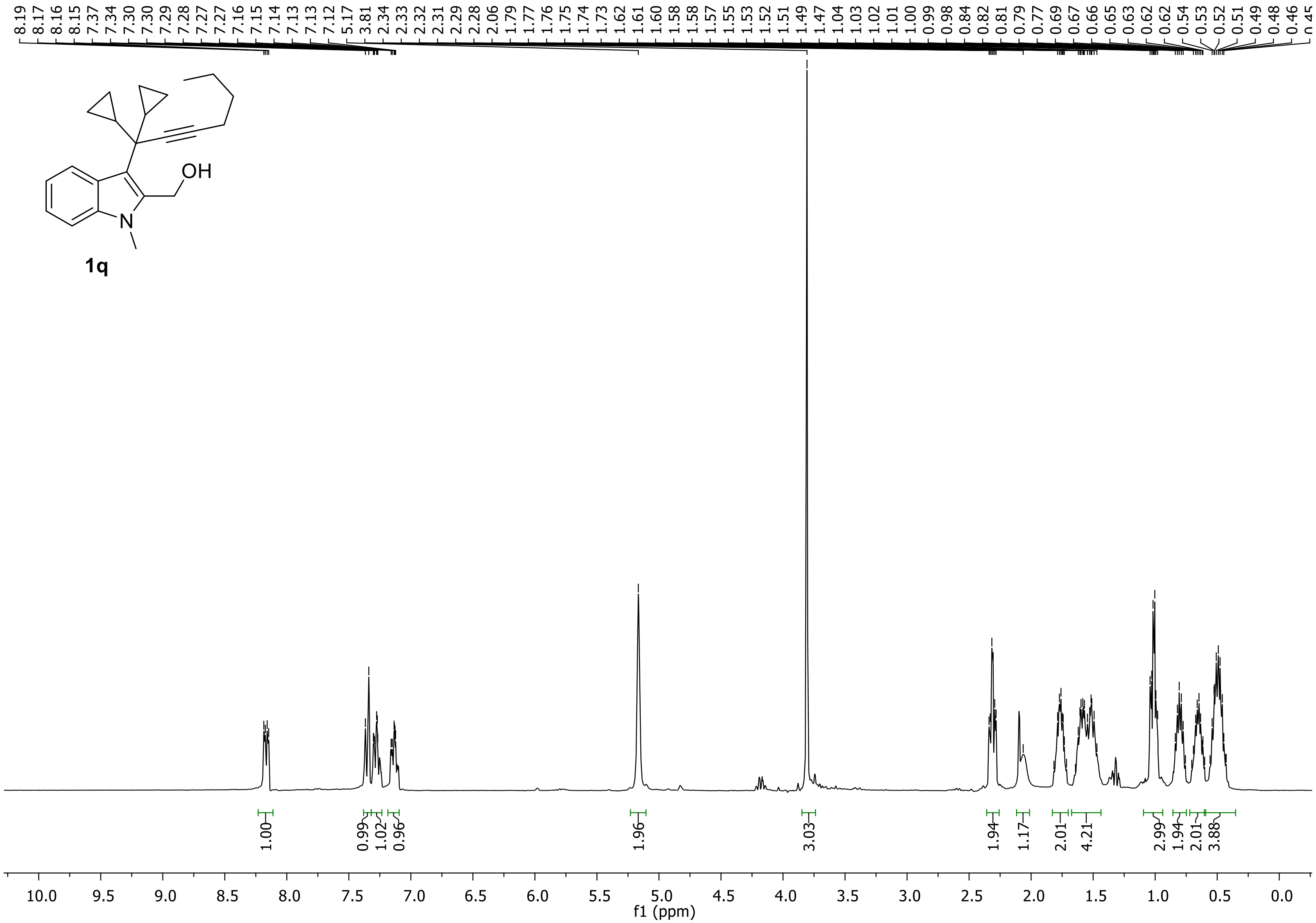

^{13}C NMR ($CDCl_3$, 75.4 MHz)

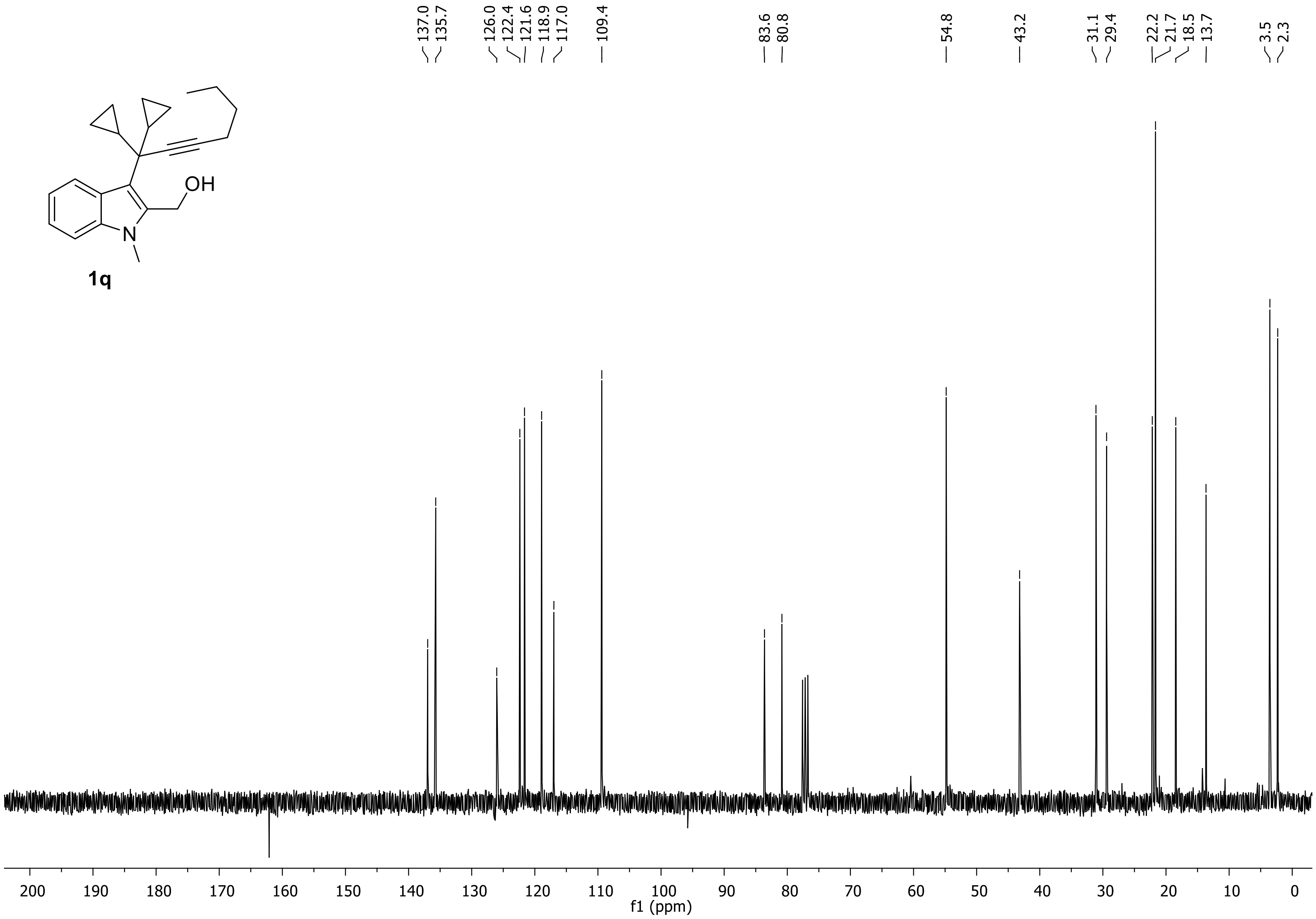

DEPT ($CDCl_3$, 75.4 MHz)

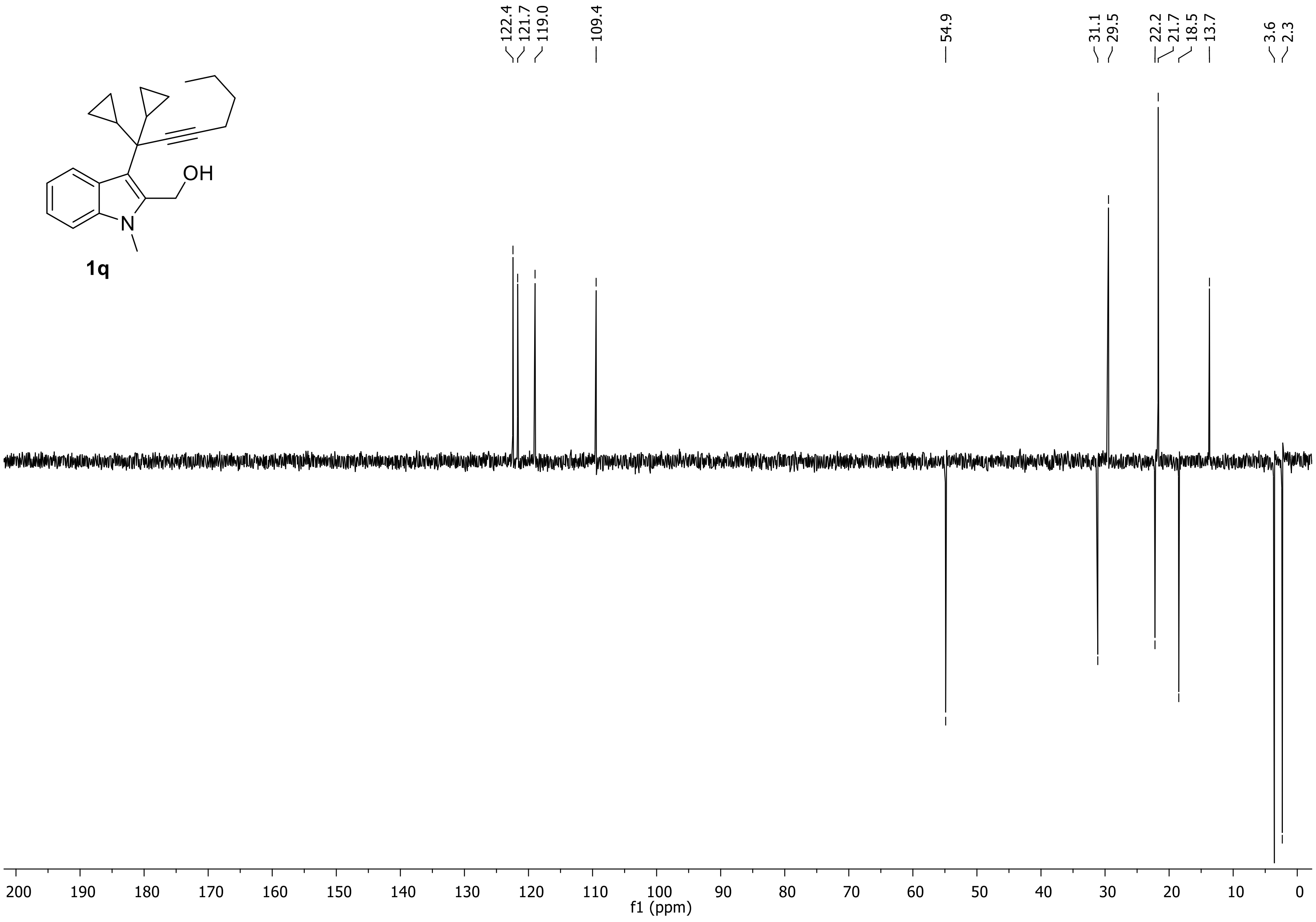

^{1}H NMR ($CDCl_3$, 300 MHz)

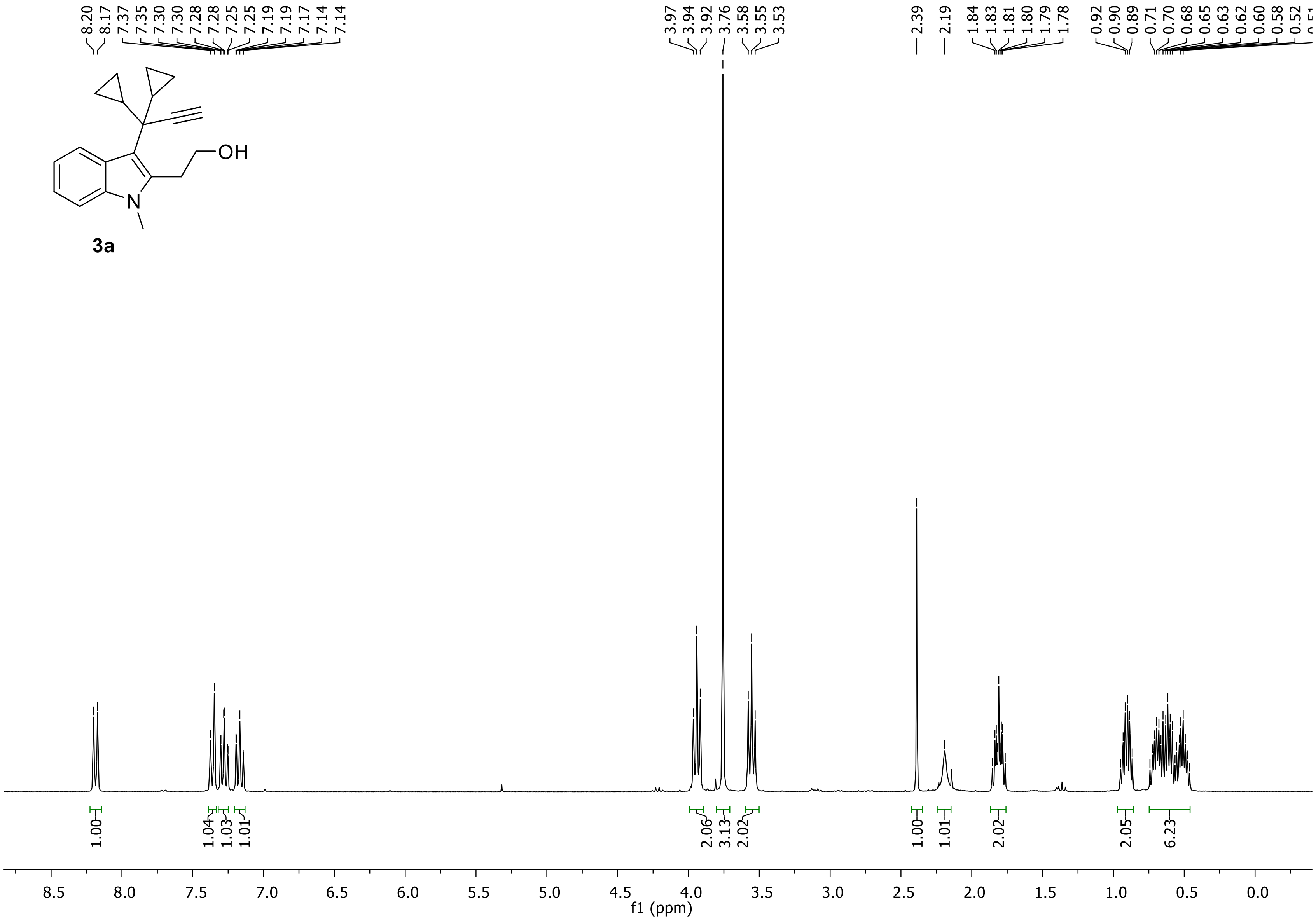

3a

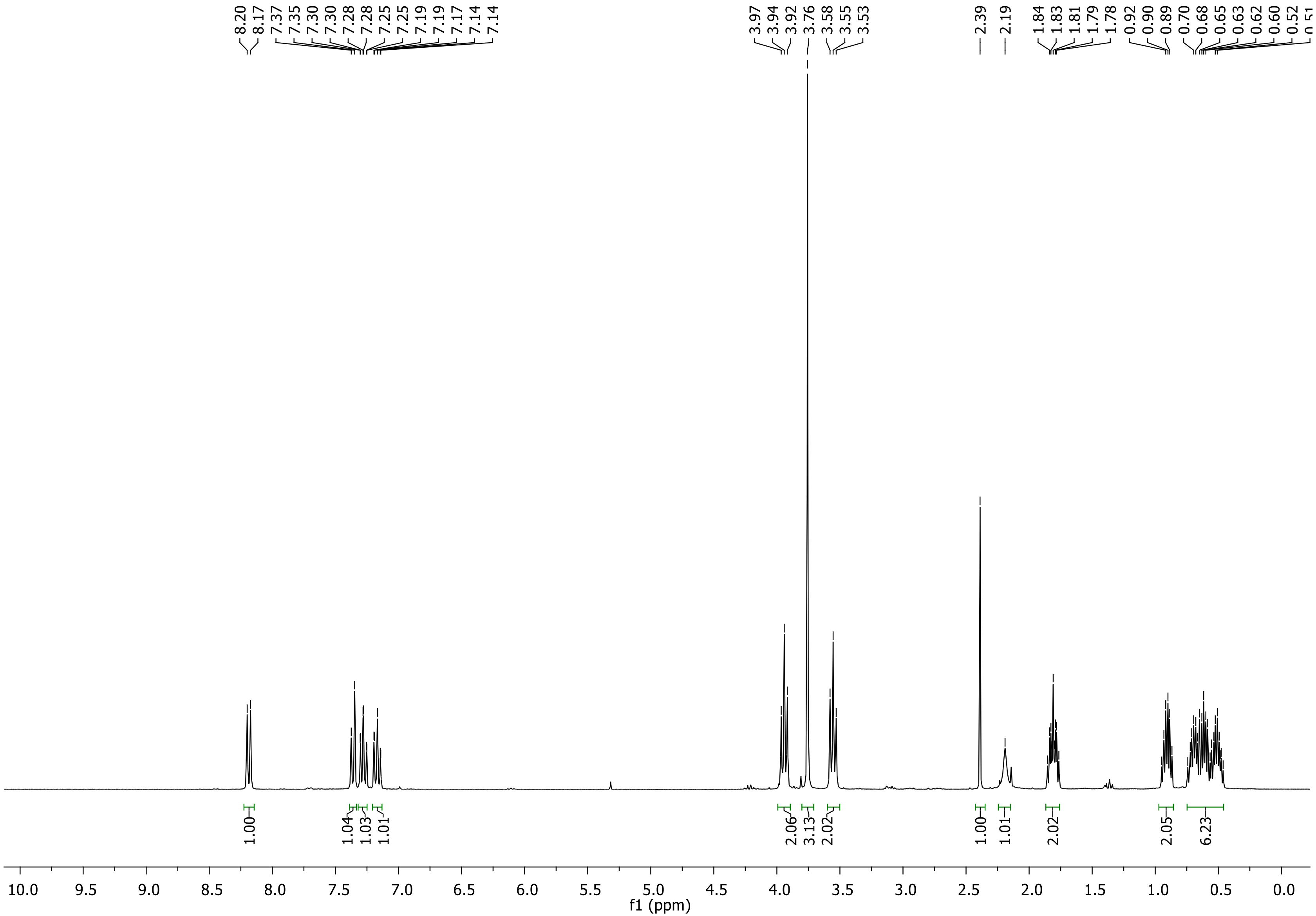

8.20
8.17
7.37
7.35
7.30
7.30
7.28
7.28
7.25
7.25
7.19
7.19
7.17
7.14
7.14
3.97
3.94
3.92
3.76
3.58
3.55
3.53
2.39
2.19
1.84
1.83
1.81
1.79
1.78
0.92
0.90
0.89
0.70
0.68
0.65
0.63
0.62
0.60
0.52
1.00
1.04
1.03
1.01
2.06
3.13
2.02
1.00
1.01
2.02
2.05
6.23
10.0
9.5
9.0
8.5
8.0
7.5
7.0
6.5
6.0
5.5
5.0
4.5
4.0
3.5
3.0
2.5
2.0
1.5
1.0
0.5
0.0
f1 (ppm)

^{13}C NMR ($CDCl_3$, 75.4 MHz)

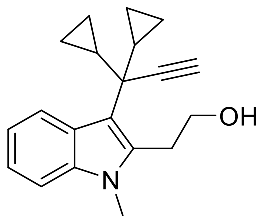

3a

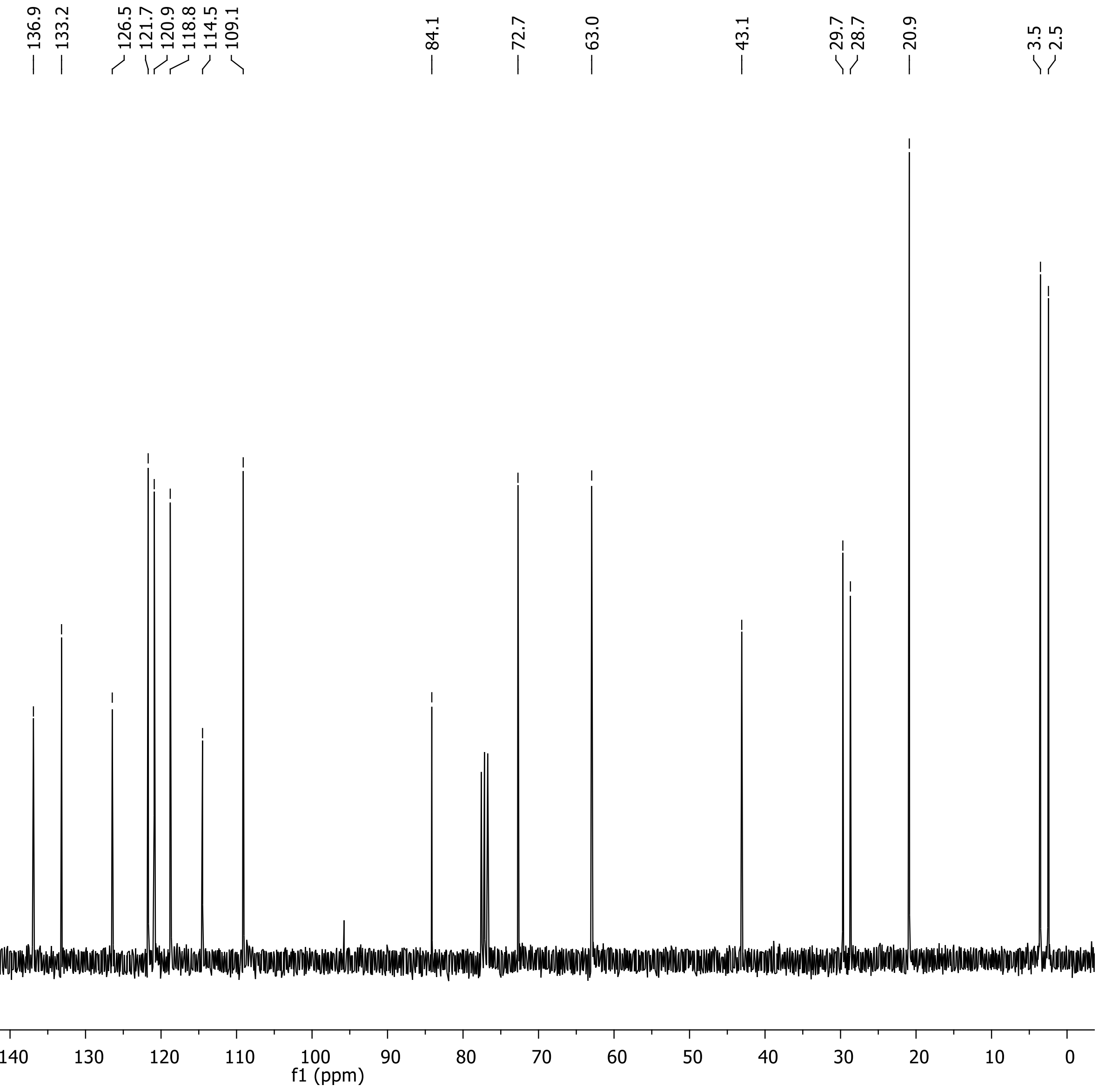

DEPT ($CDCl_3$, 75.4 MHz)

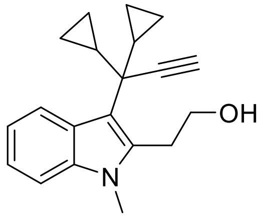

3a

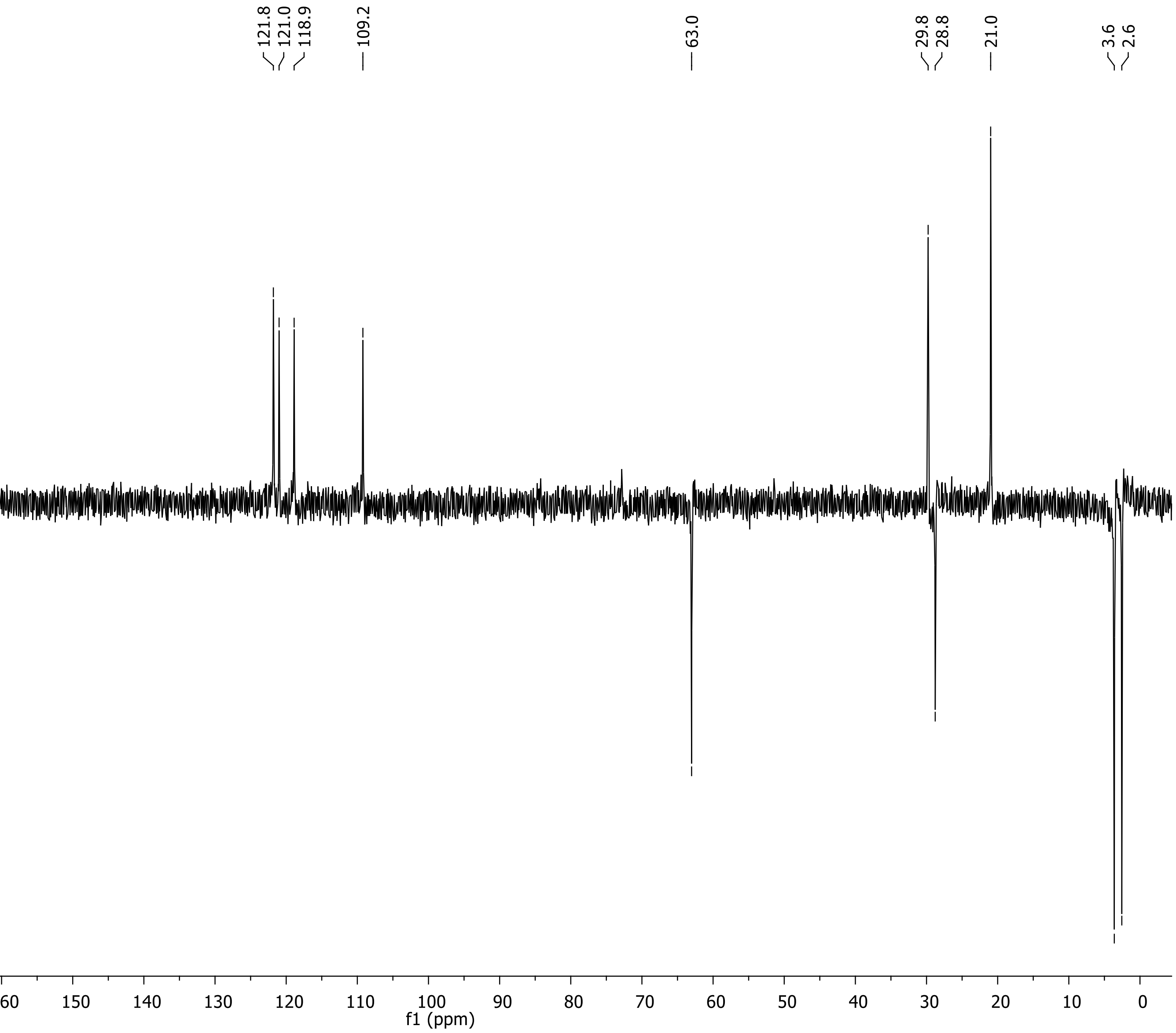

^{1}H NMR ($CDCl_3$, 300 MHz)

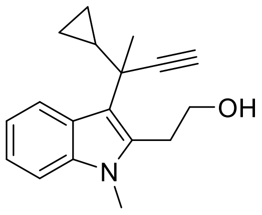

3b

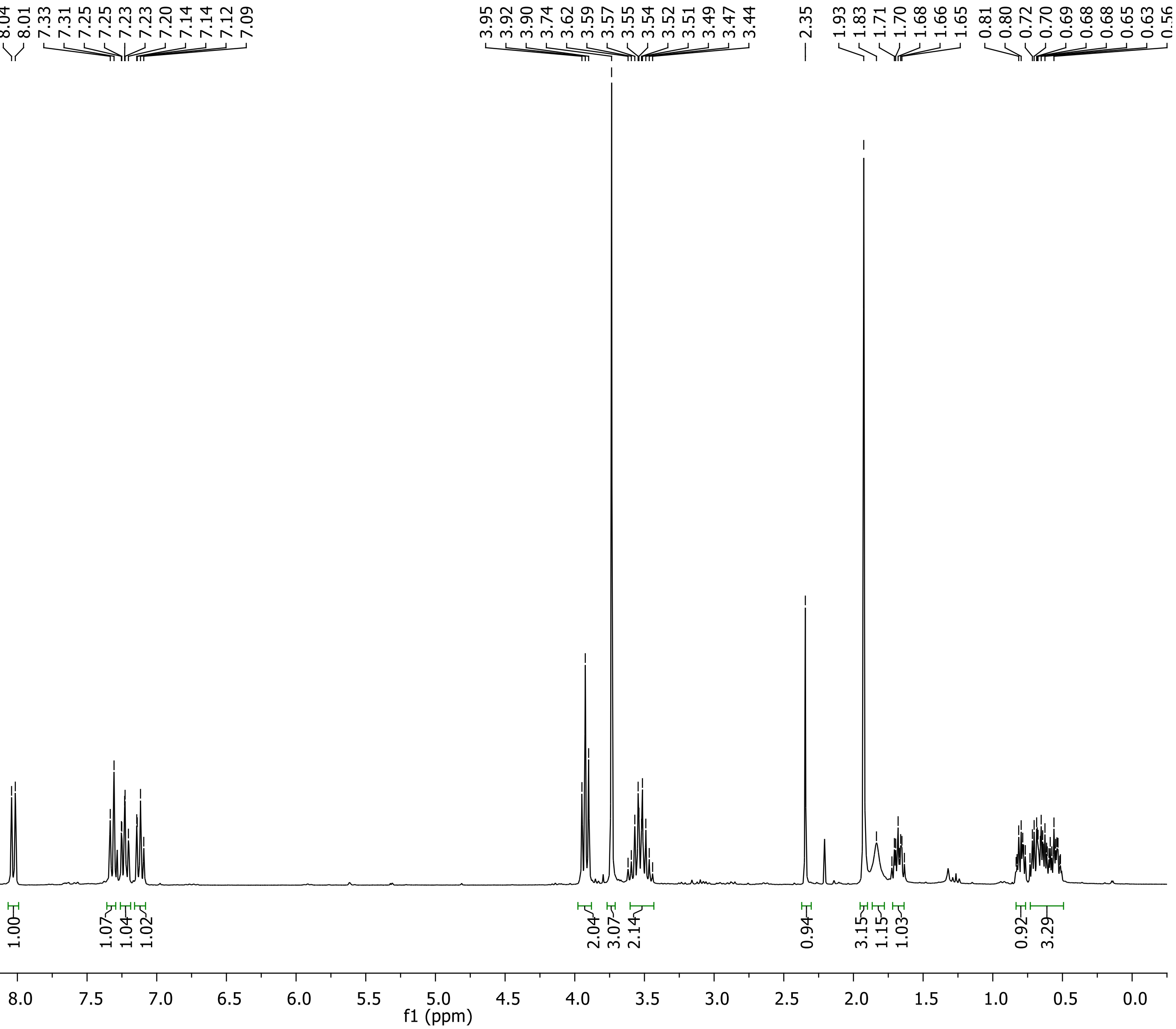

^{13}C NMR ($CDCl_3$, 75.4 MHz)

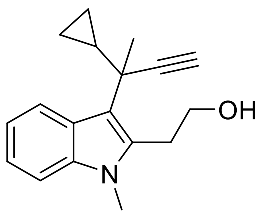

3b

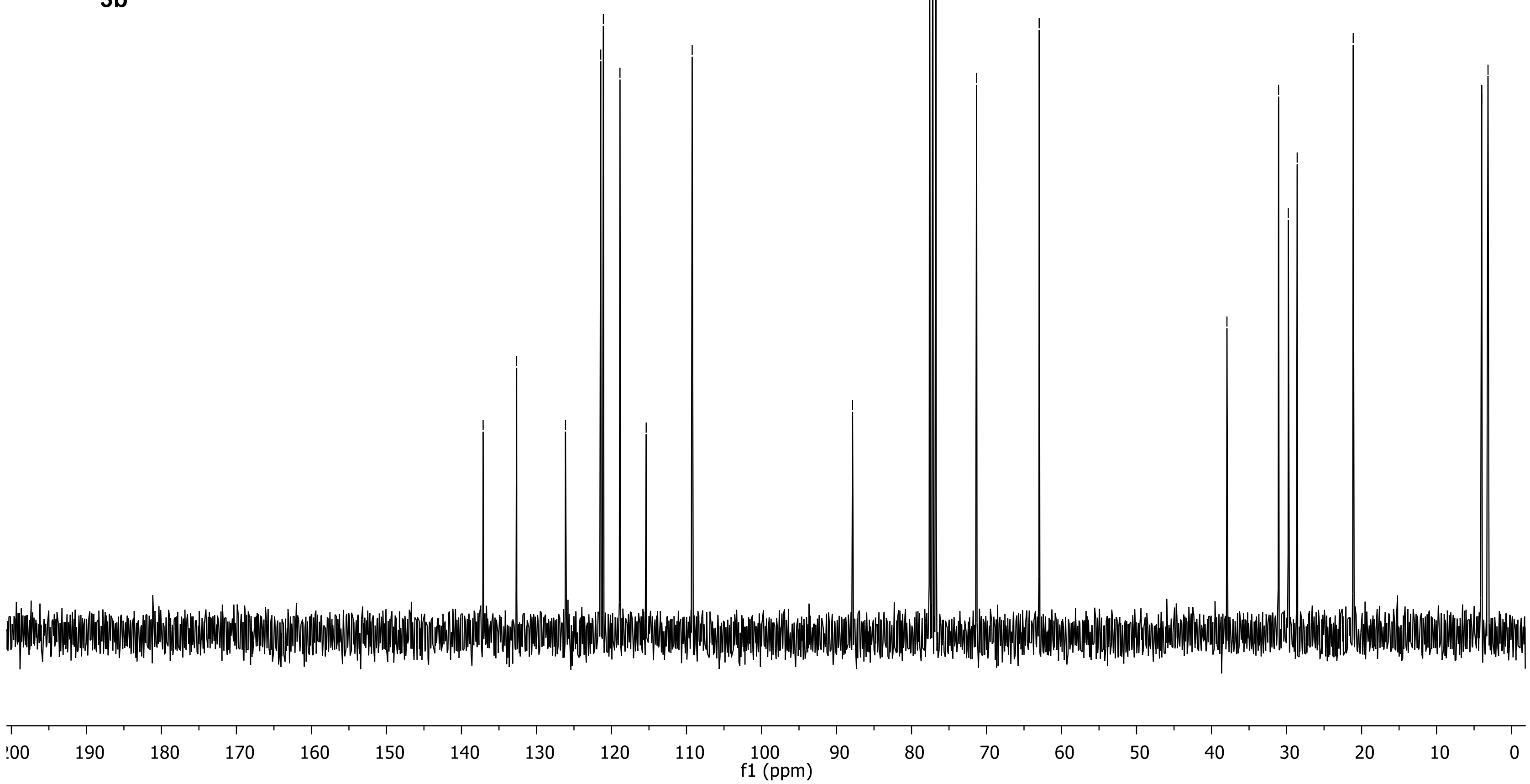

DEPT ($CDCl_3$, 75.4 MHz)

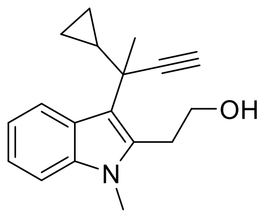

3b

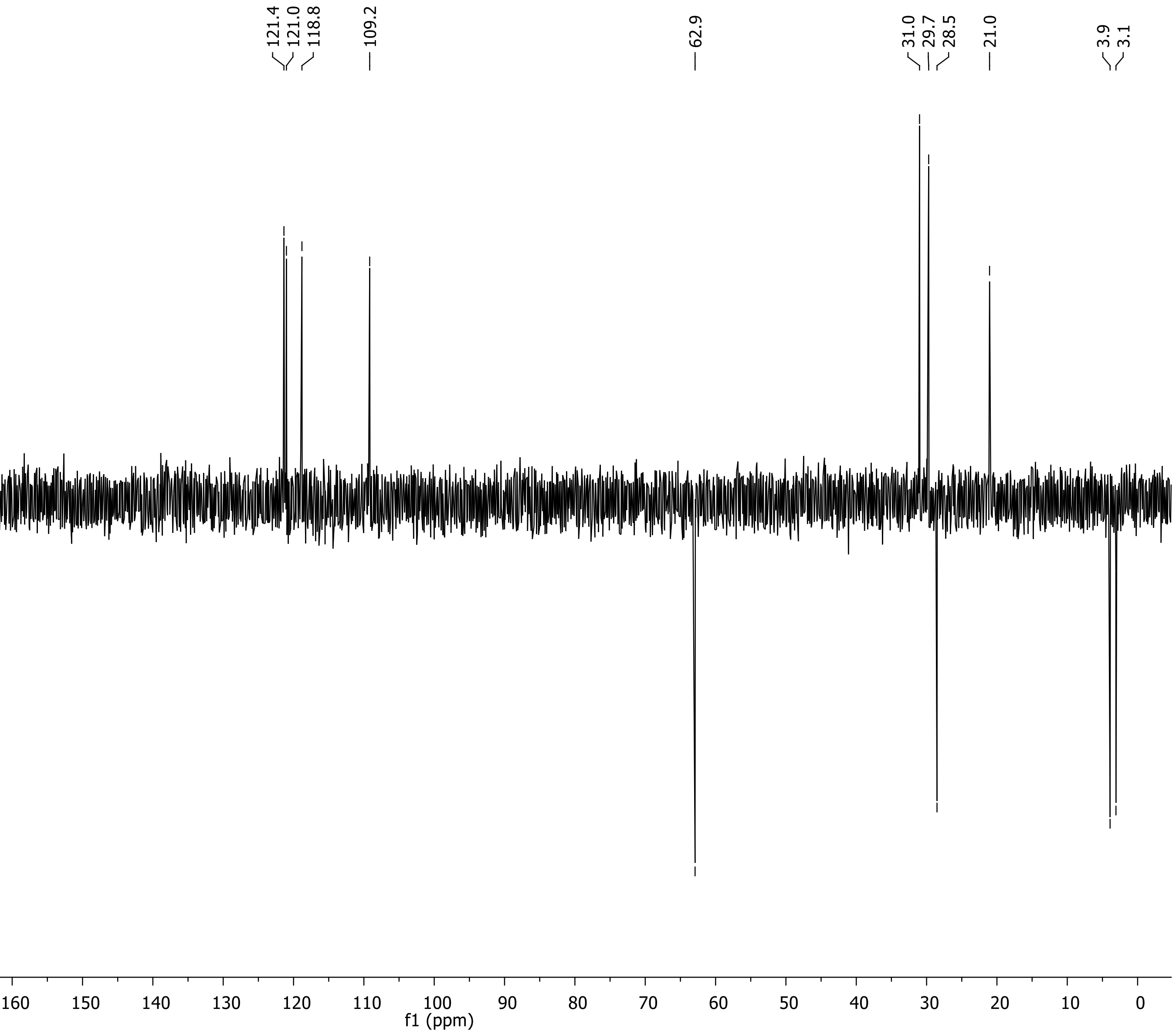

^{1}H NMR ($CDCl_3$, 300 MHz)

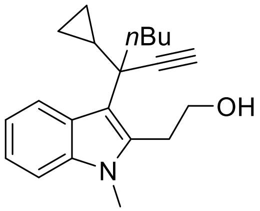

3c

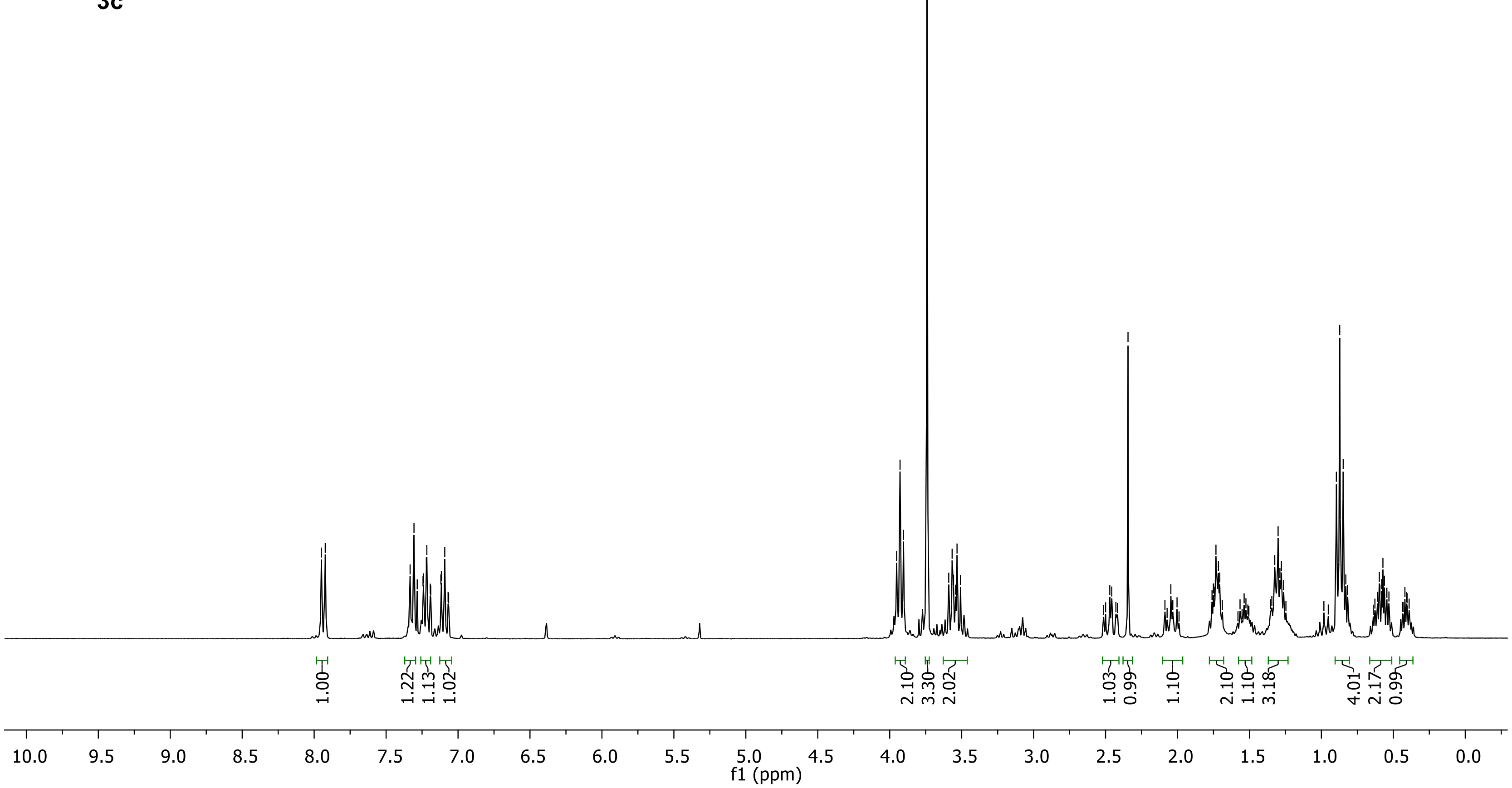

^{13}C NMR ($CDCl_3$, 75.4 MHz)

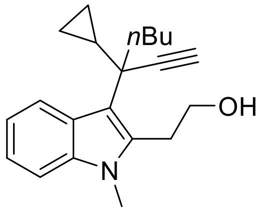

3c

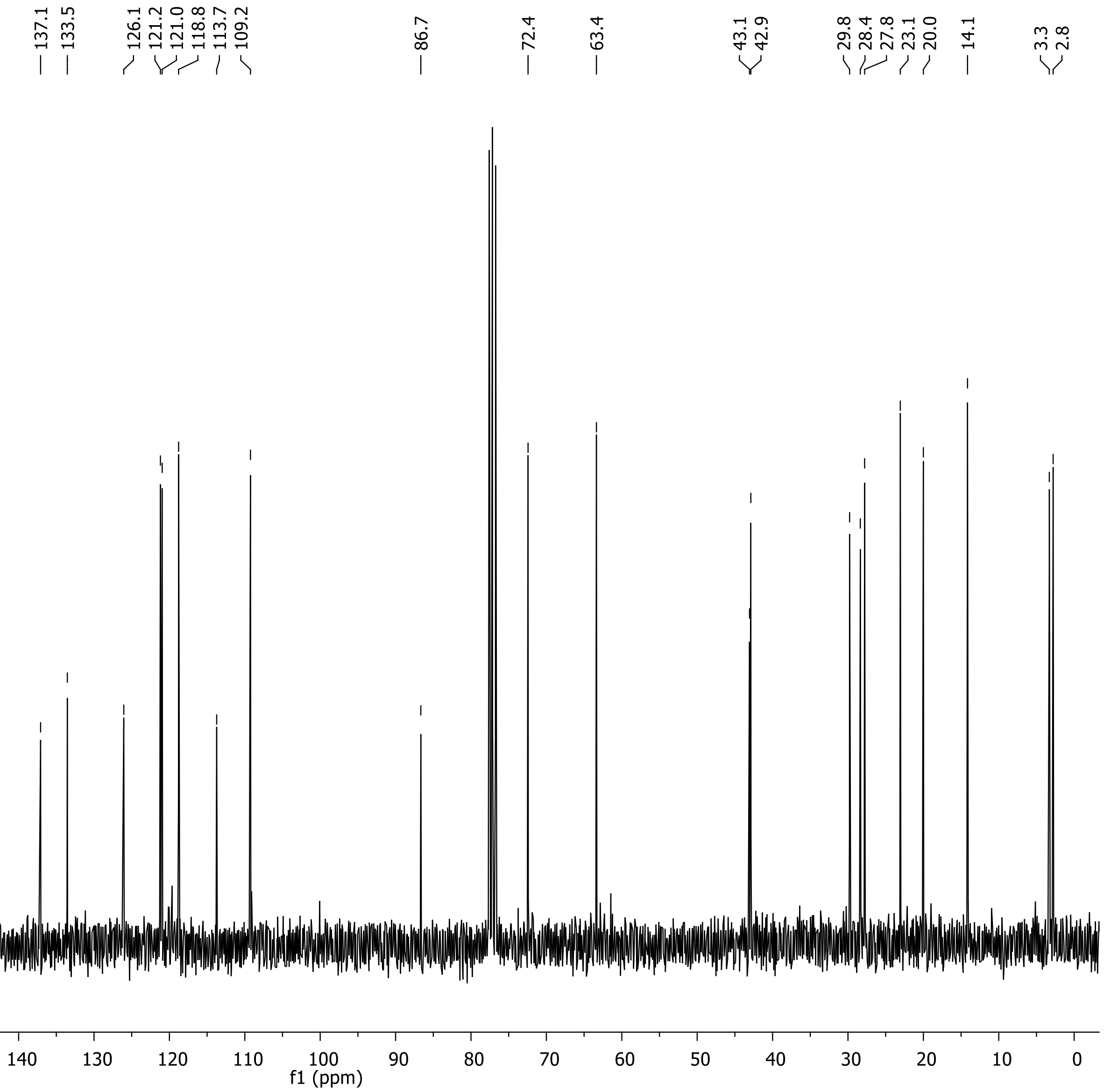

DEPT ($CDCl_3$, 75.4 MHz)

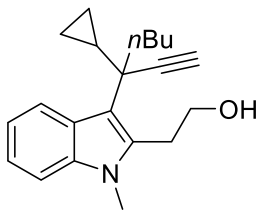

3c

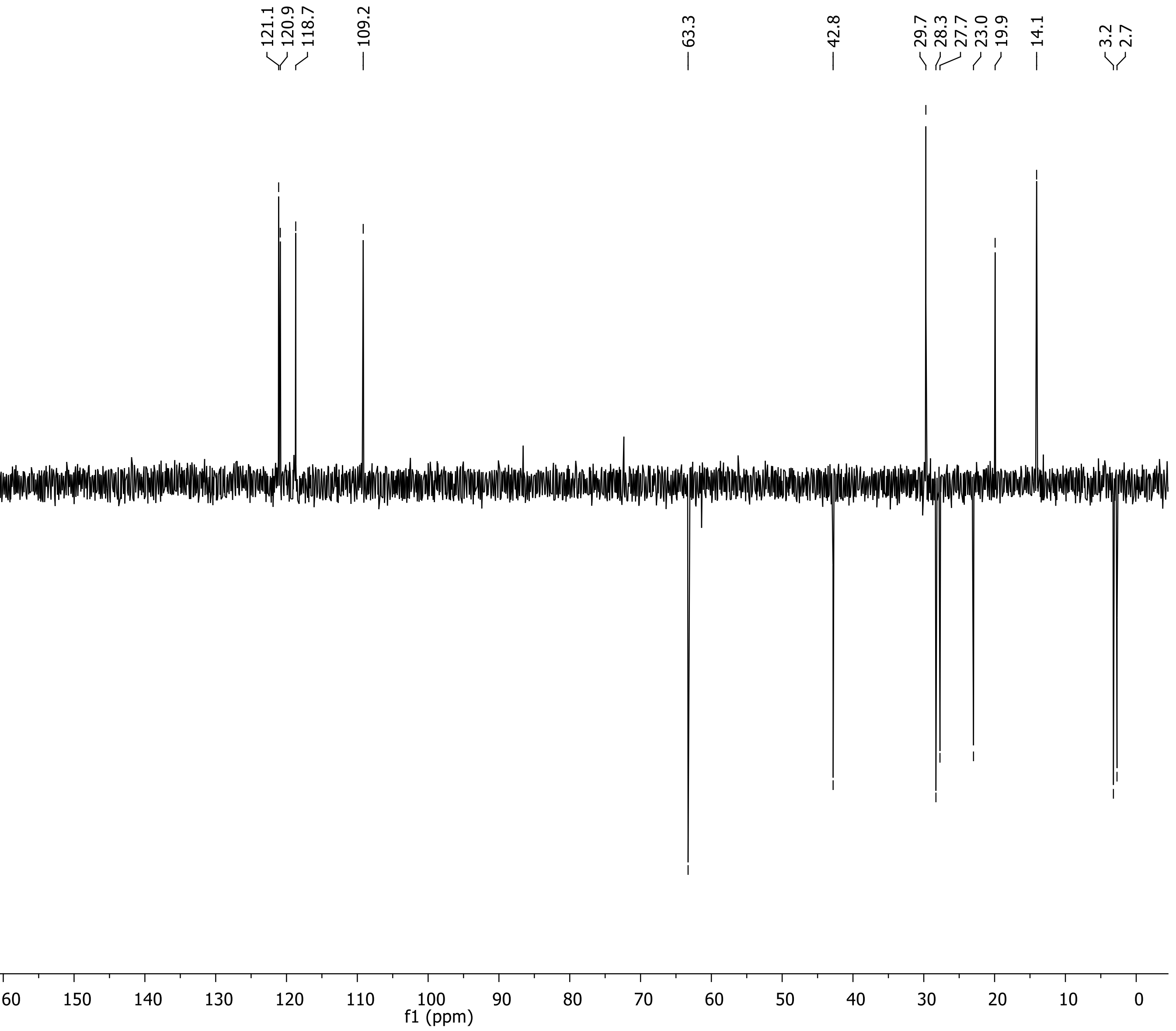

^{1}H NMR ($CDCl_3$, 500 MHz)

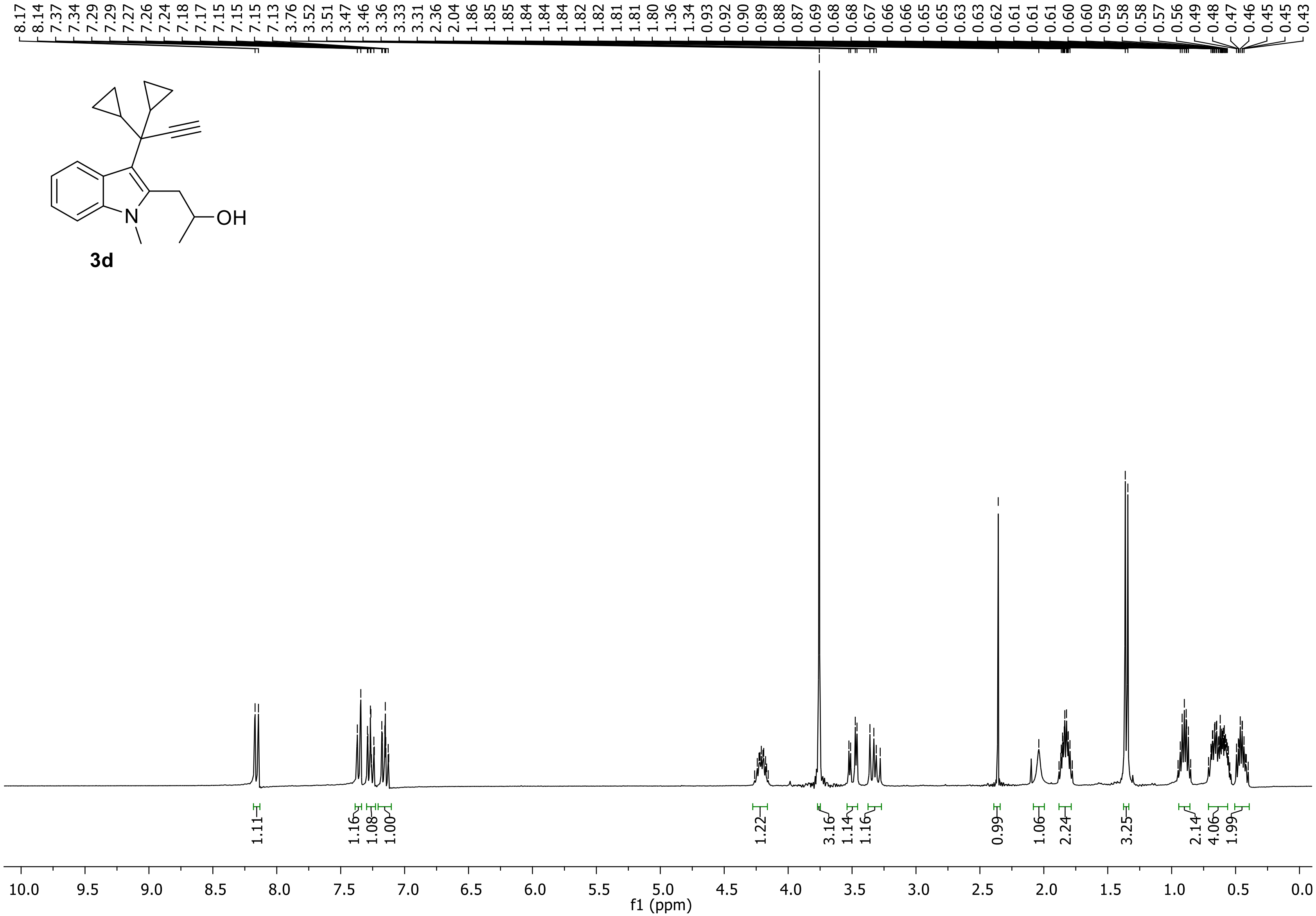

3d

^{13}C NMR ($CDCl_3$, 125.7 MHz)

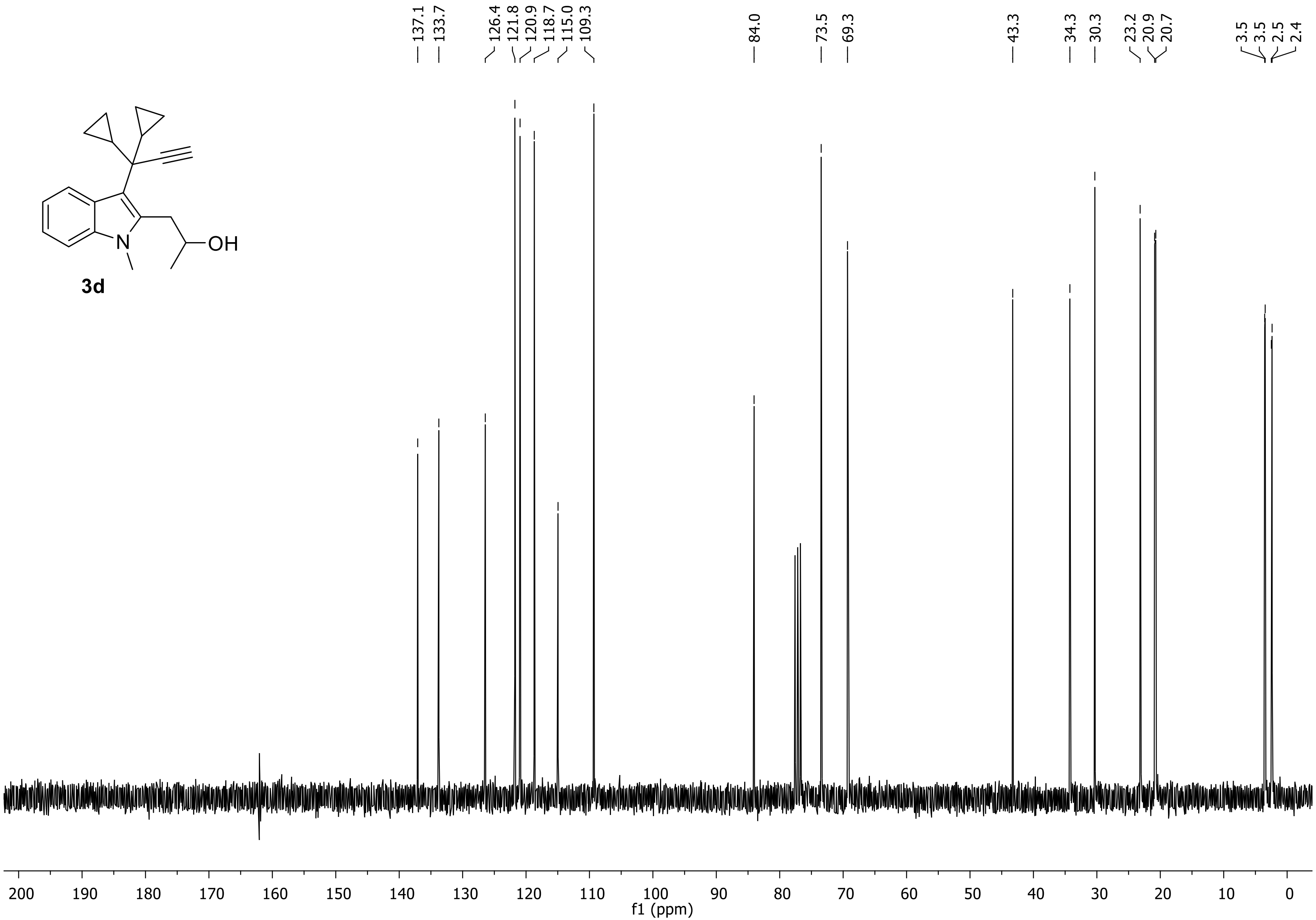

3d

DEPT ($CDCl_3$, 125.7 MHz)

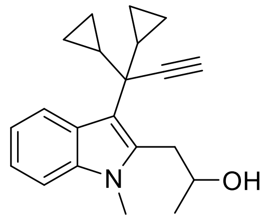

3d

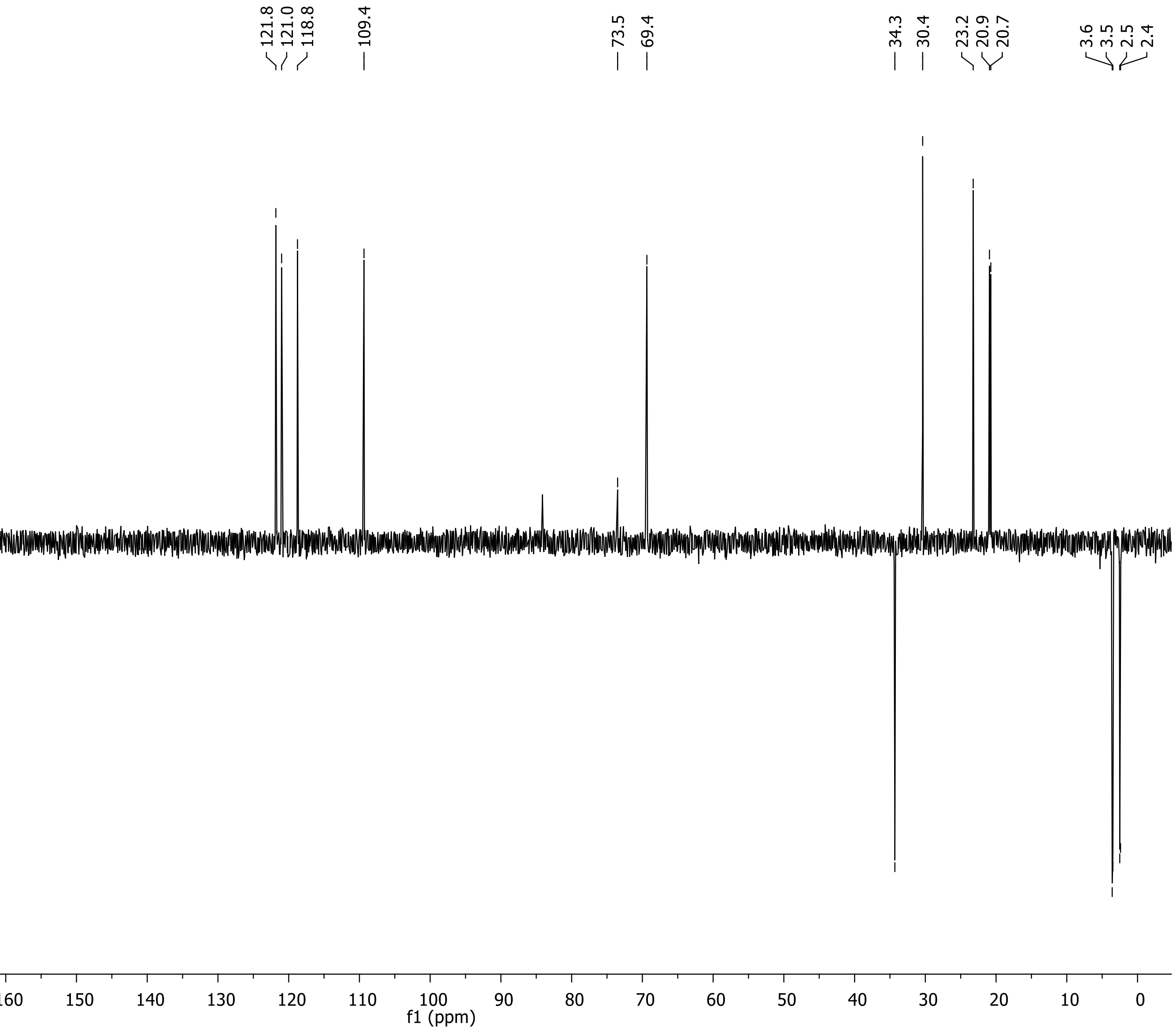

^{1}H NMR ($CDCl_3$, 300 MHz)

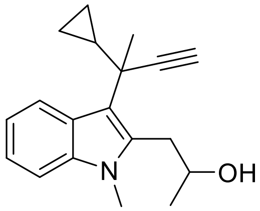

3e

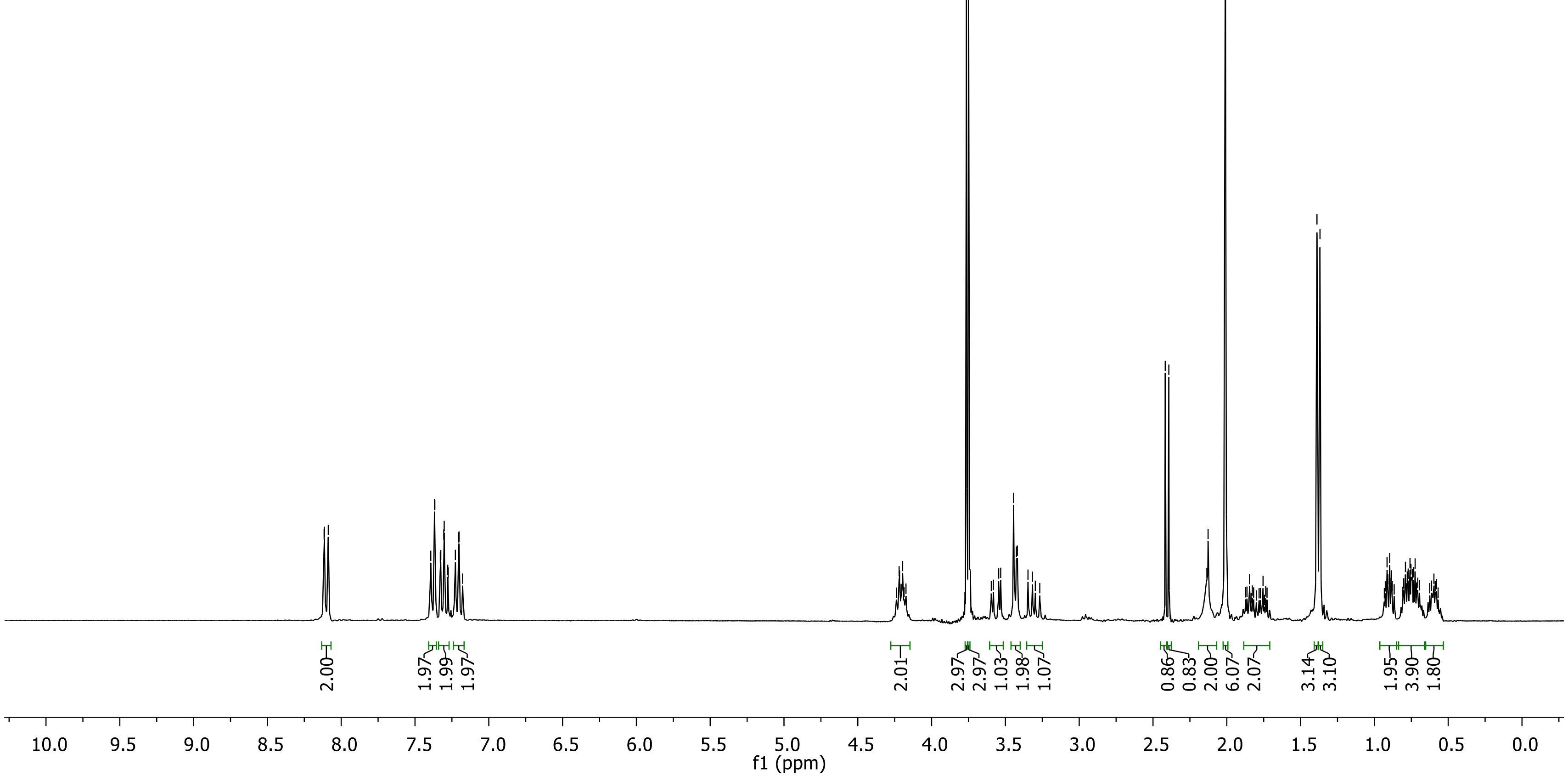

^{13}C NMR ($CDCl_3$, 75.4 MHz)

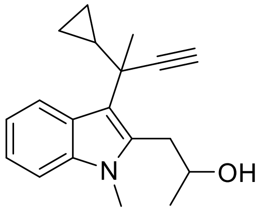

3e

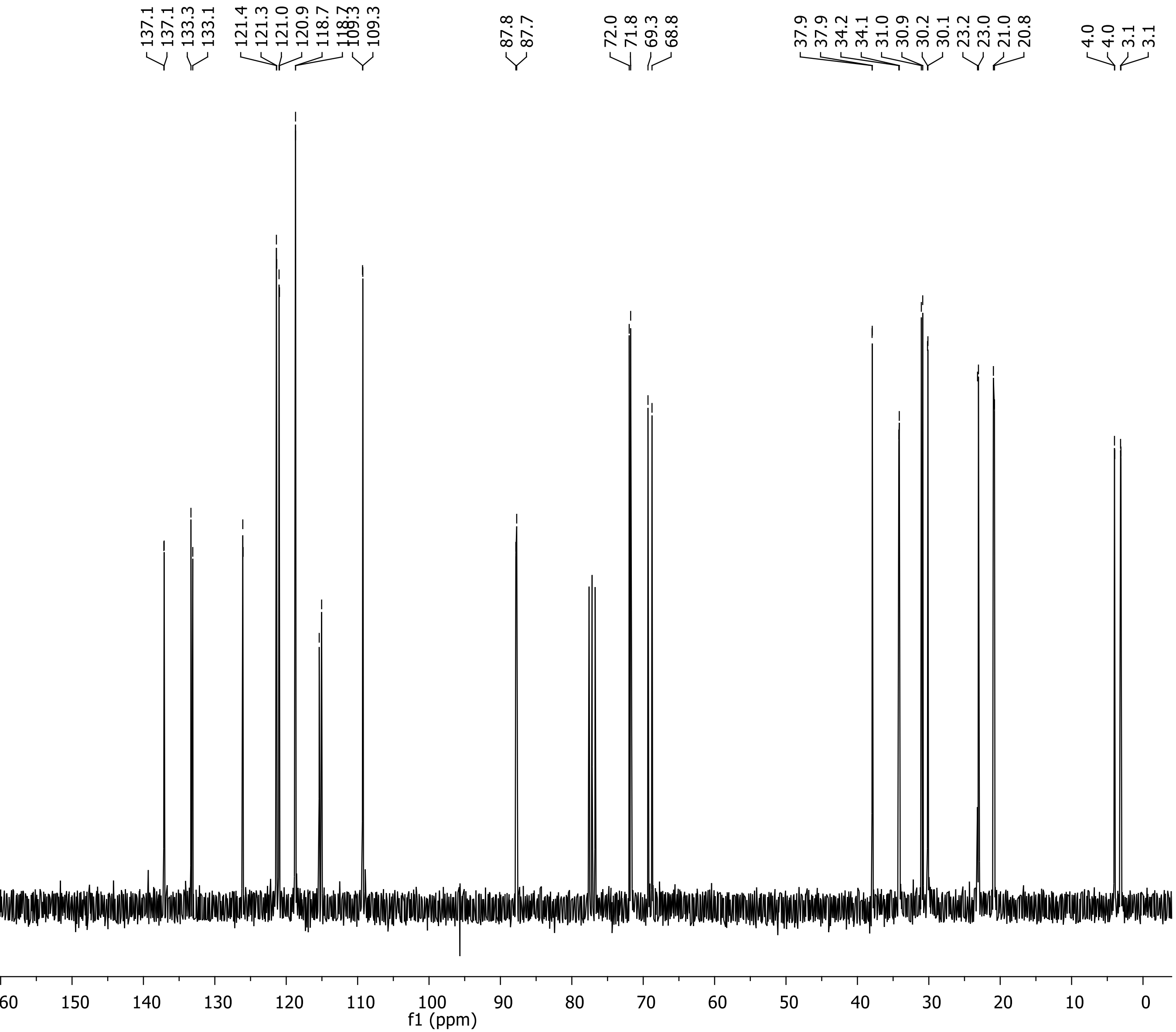

DEPT ($CDCl_3$, 75.4 MHz)

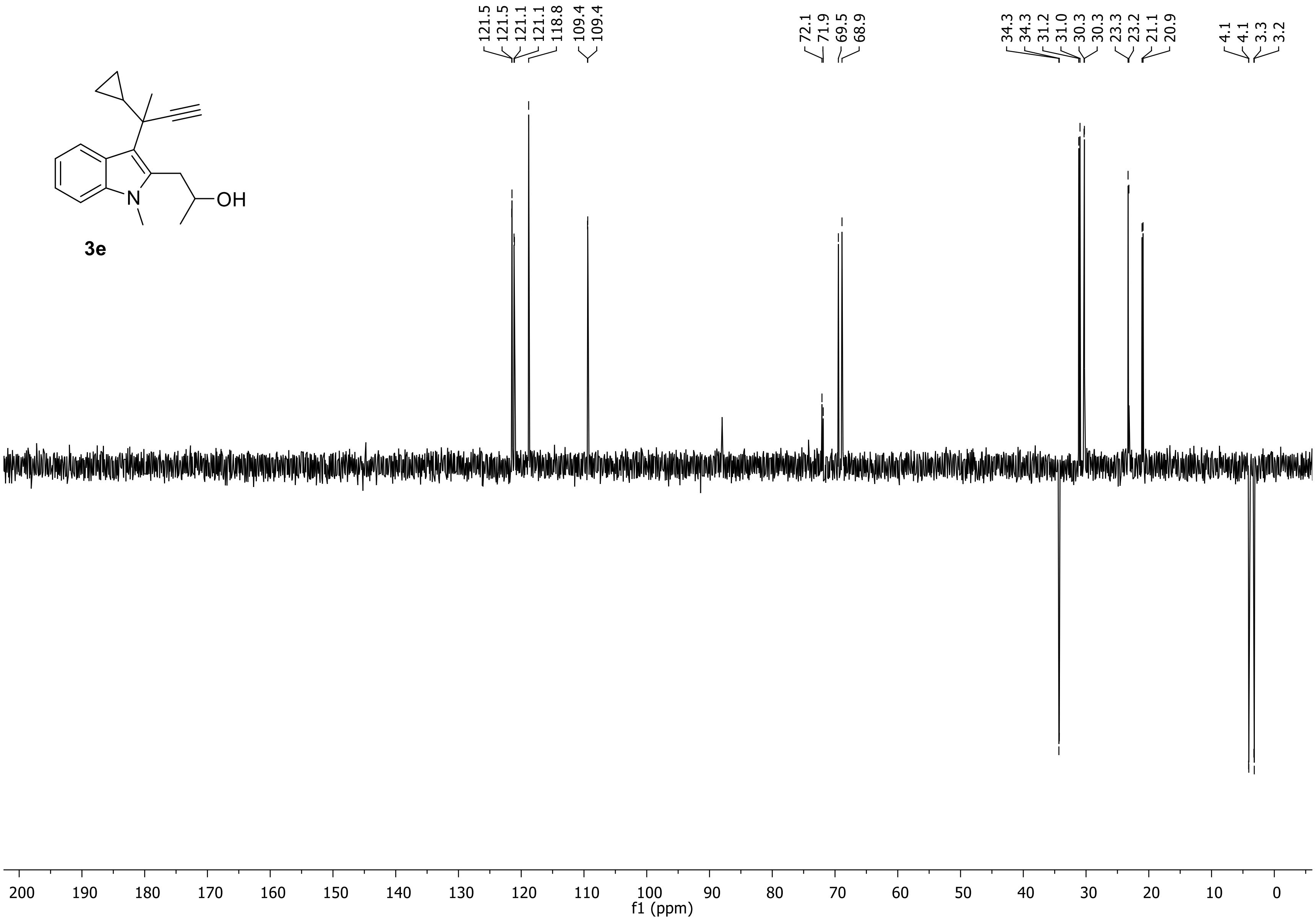

^{1}H NMR ($CDCl_3$, 300 MHz)

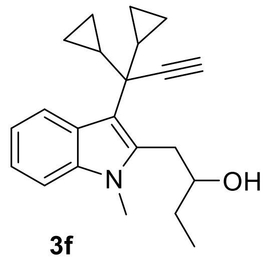

3f

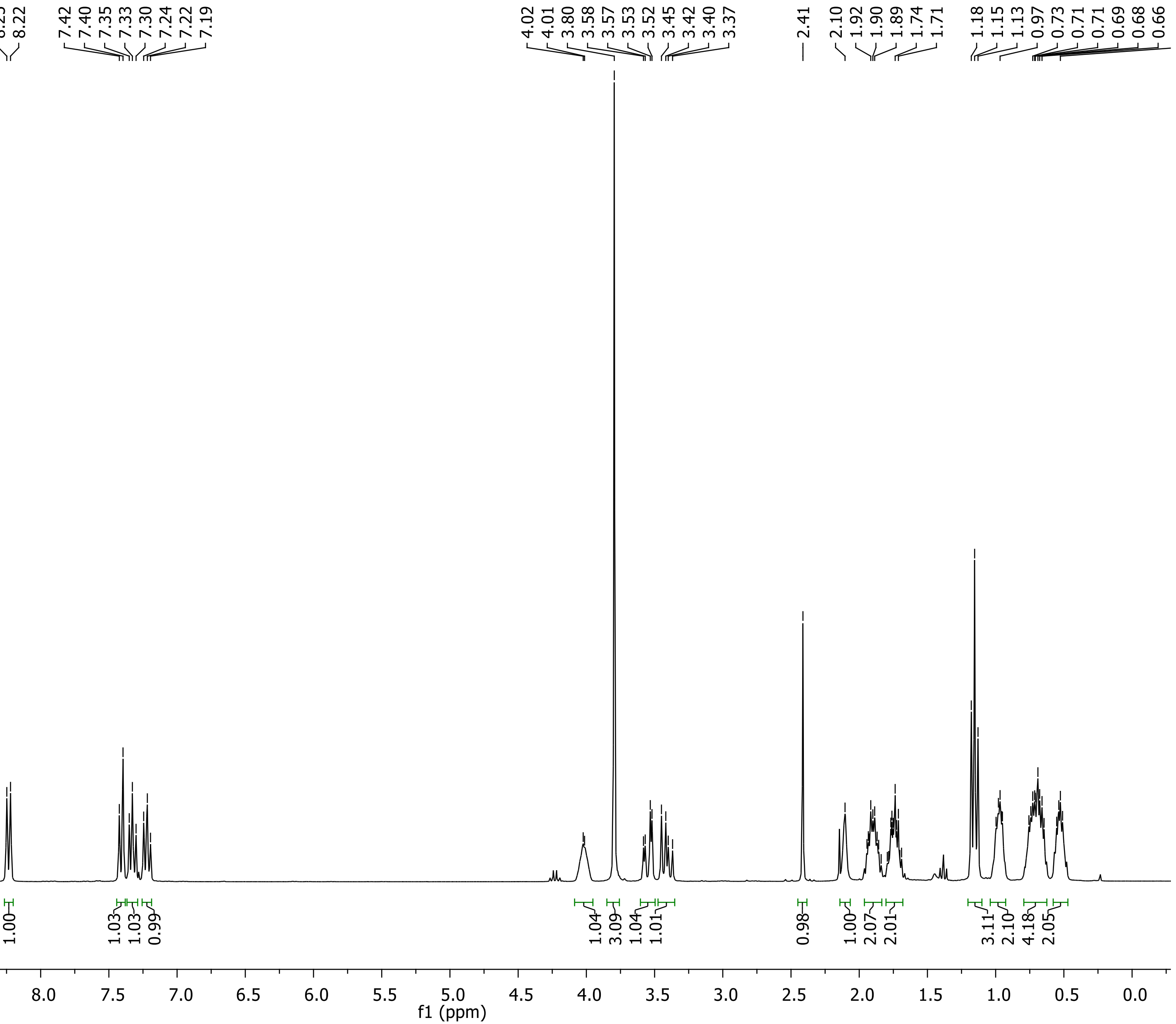

^{13}C NMR ($CDCl_3$, 75.4 MHz)

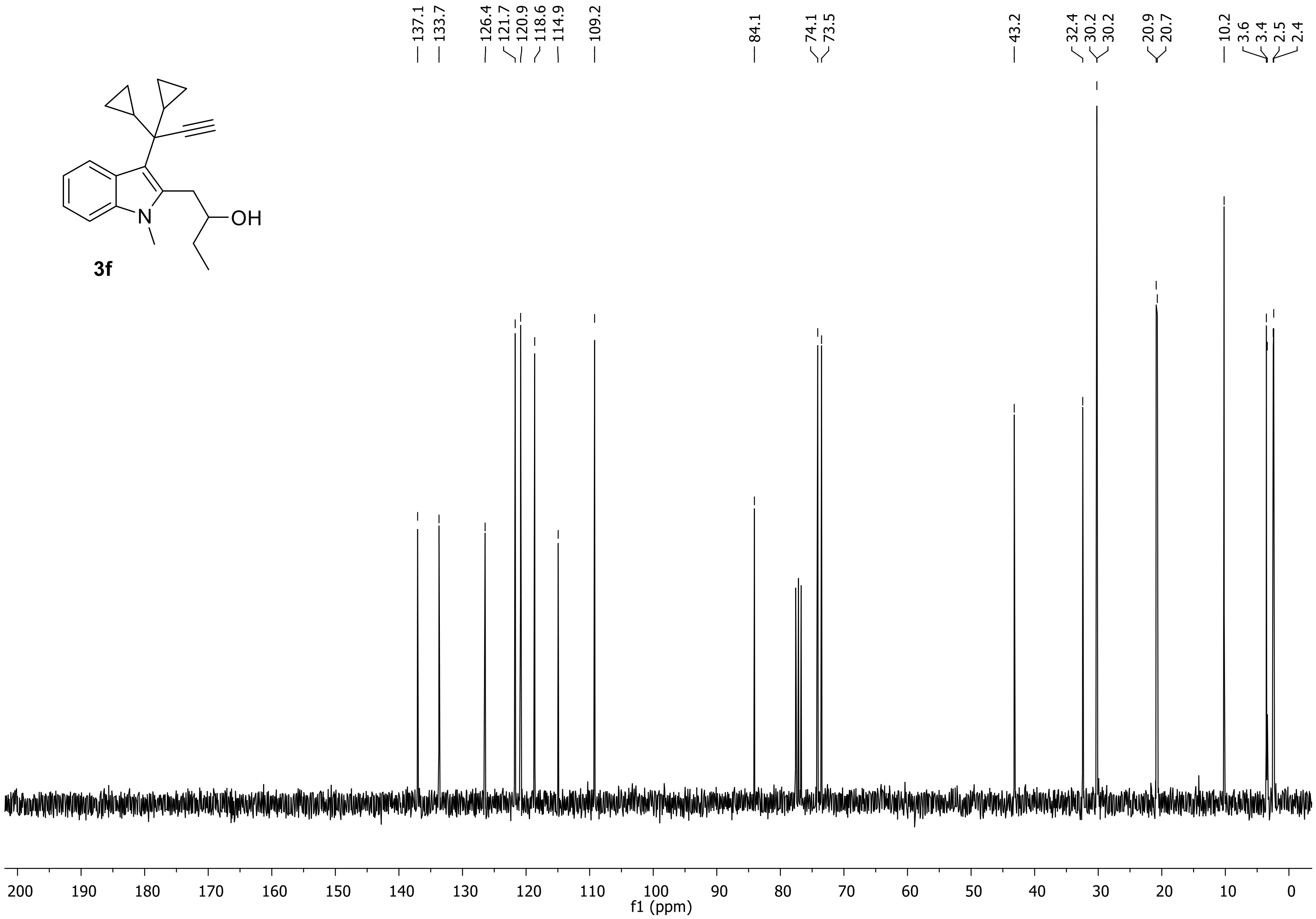

DEPT ($CDCl_3$, 75.4 MHz)

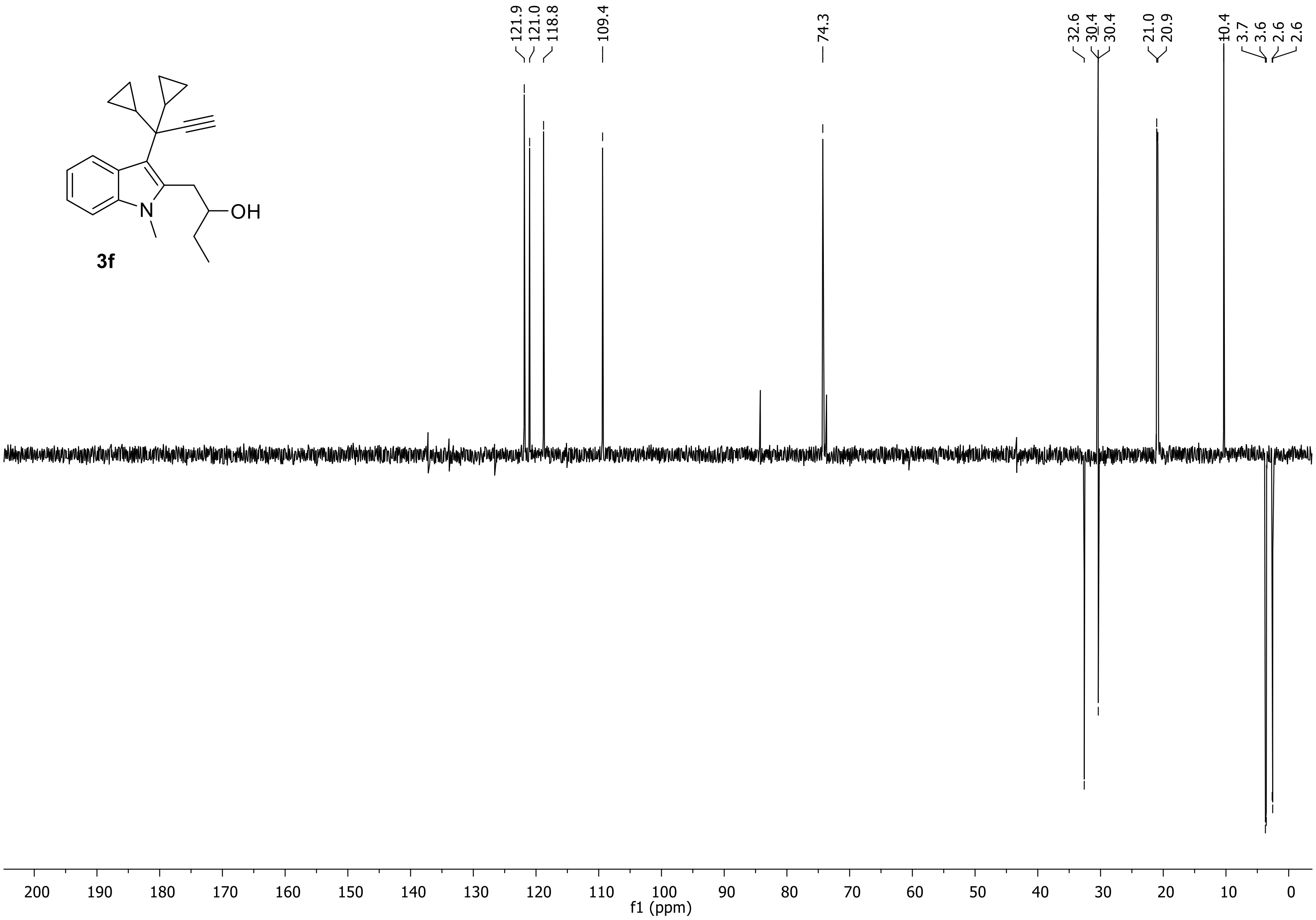

^{1}H NMR ($CDCl_3$, 300 MHz)

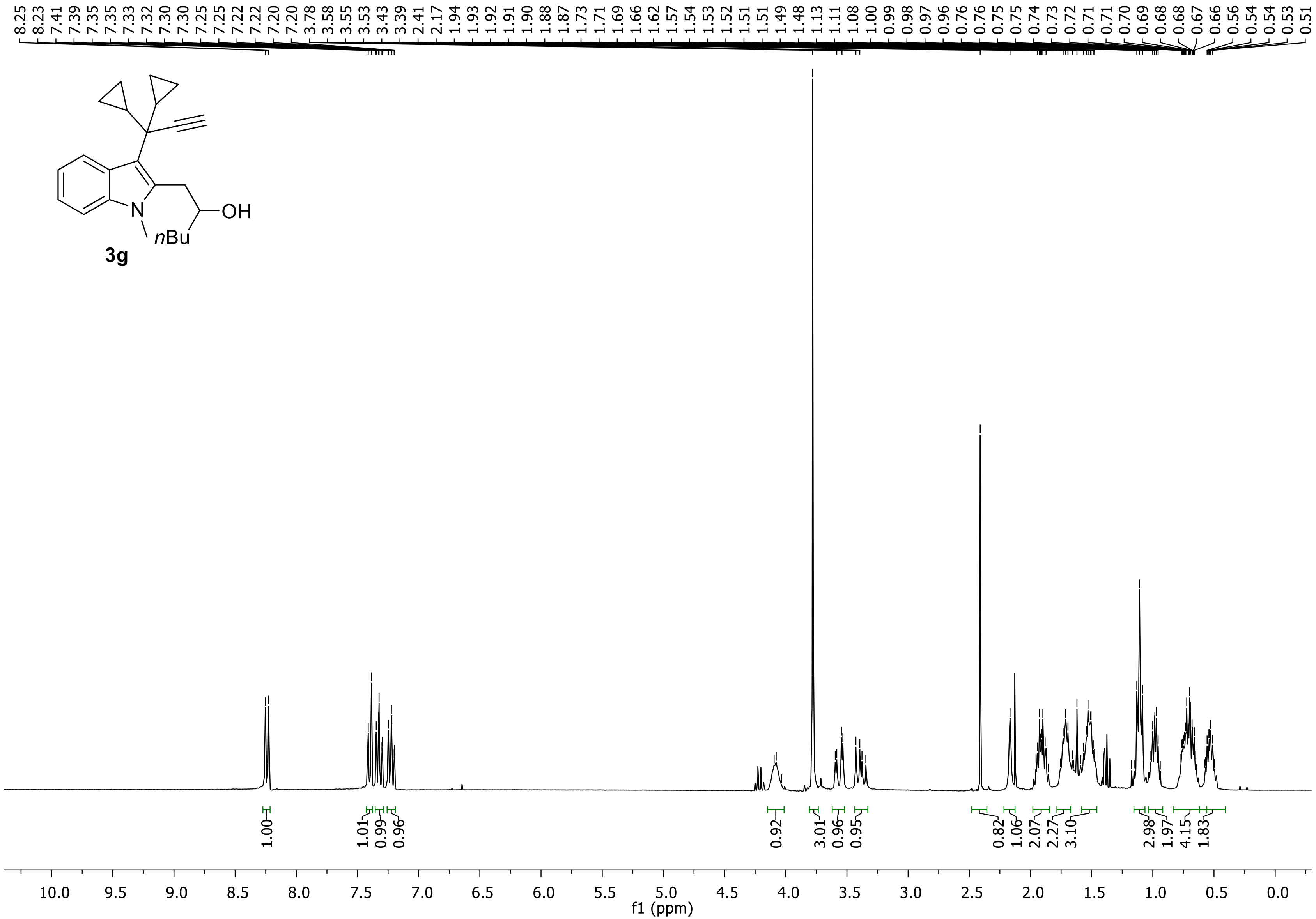

^{13}C NMR ($CDCl_3$, 75.4 MHz)

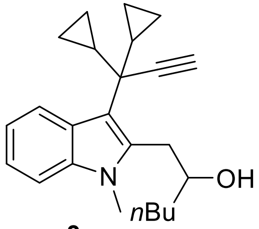

3g

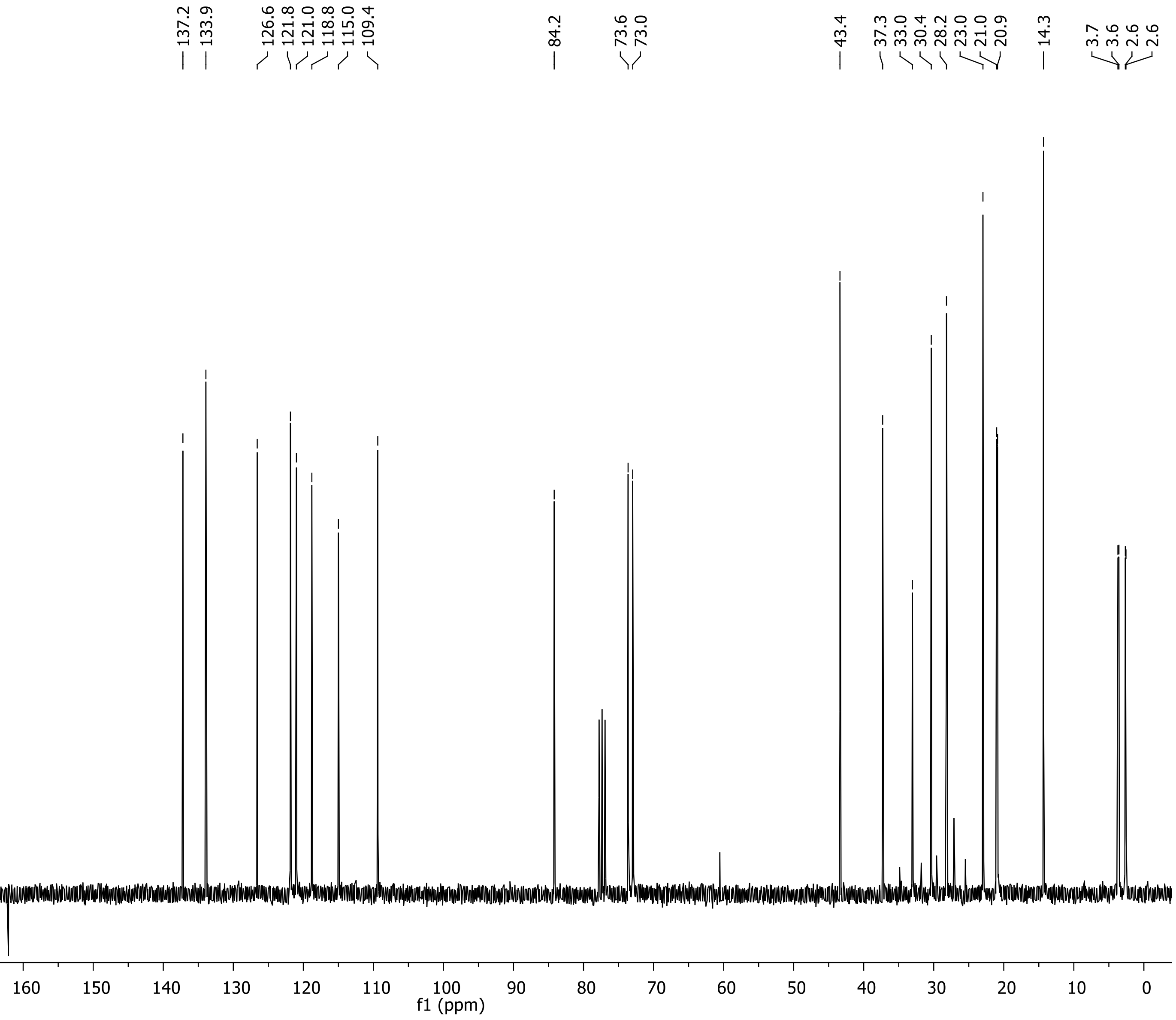

DEPT ($CDCl_3$, 75.4 MHz)

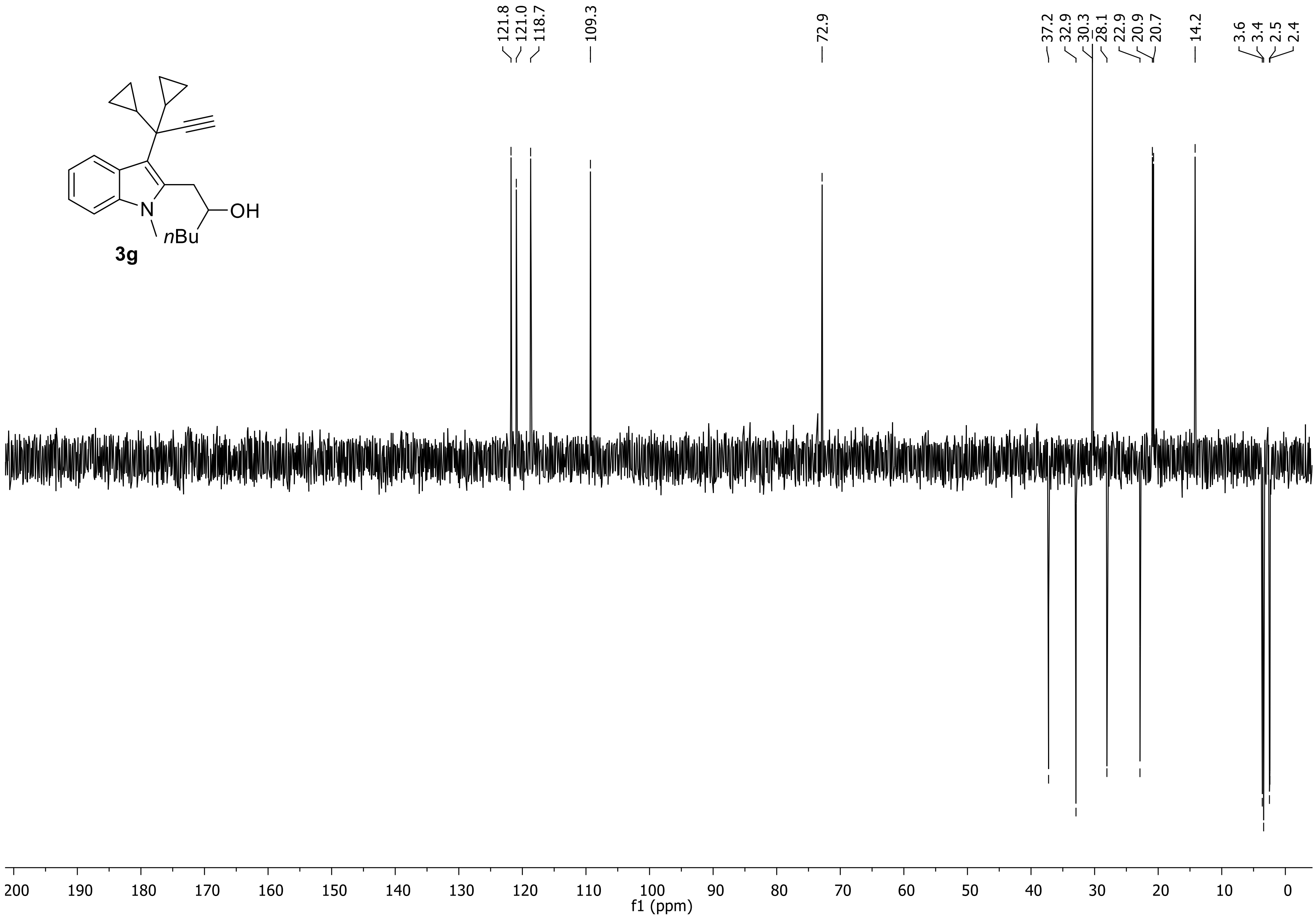

^{1}H NMR ($CDCl_3$, 500 MHz)

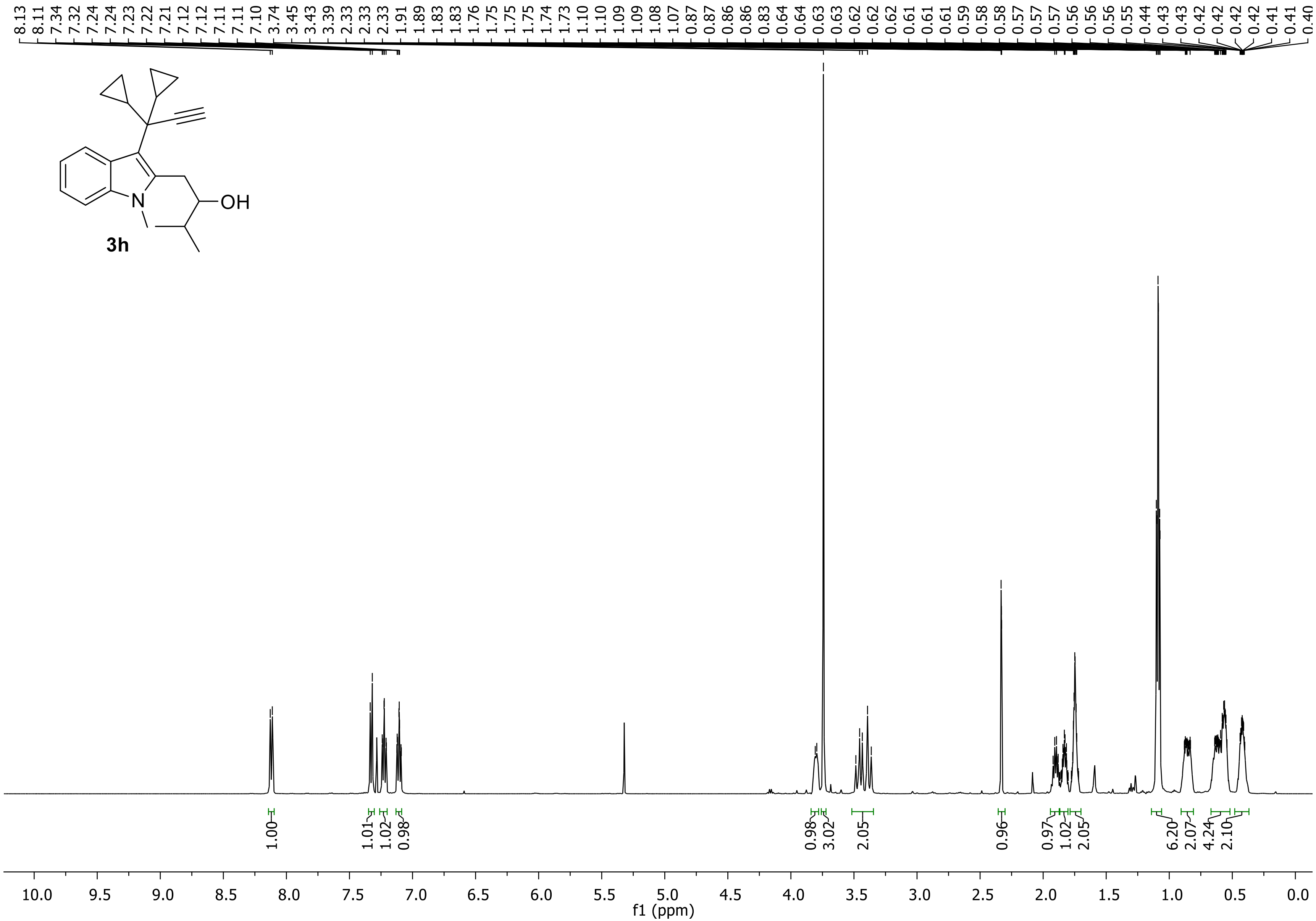

^{13}C NMR ($CDCl_3$, 125.7 MHz)

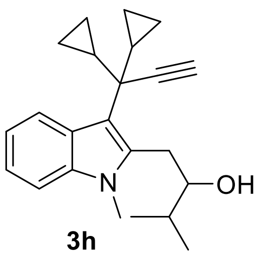

3h

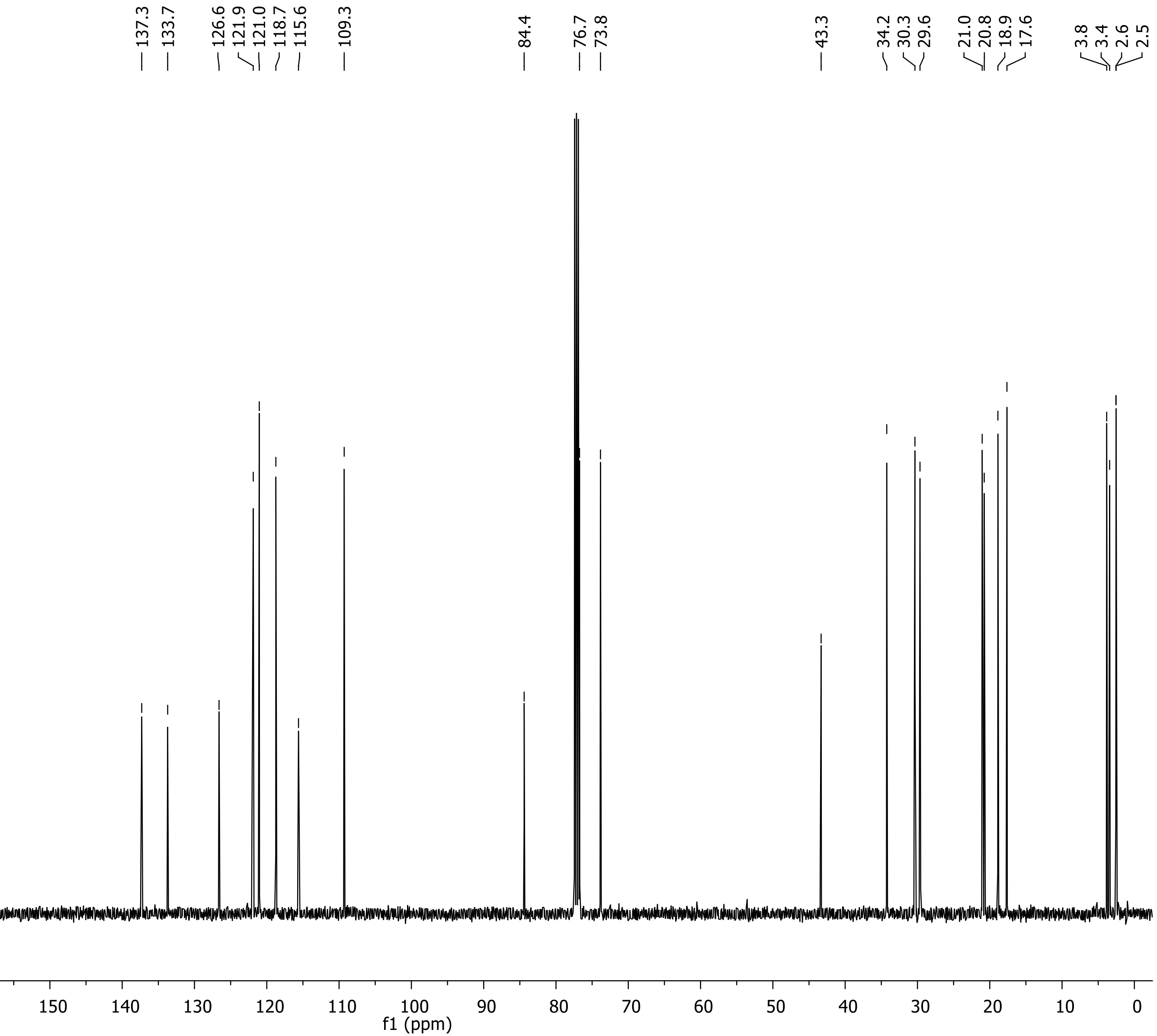

DEPT ($CDCl_3$, 125.7 MHz)

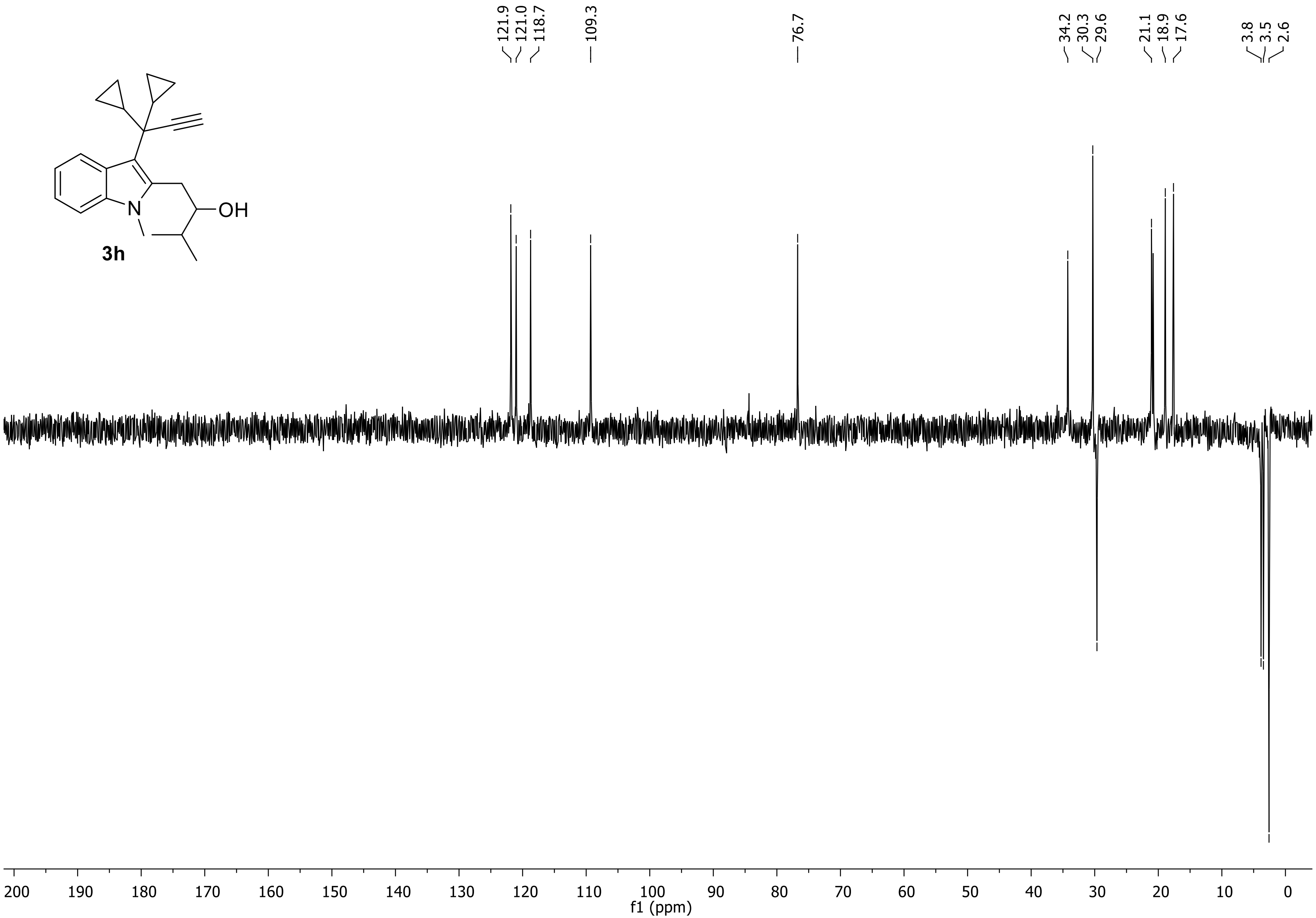

^{1}H NMR ($CDCl_3$, 300 MHz)

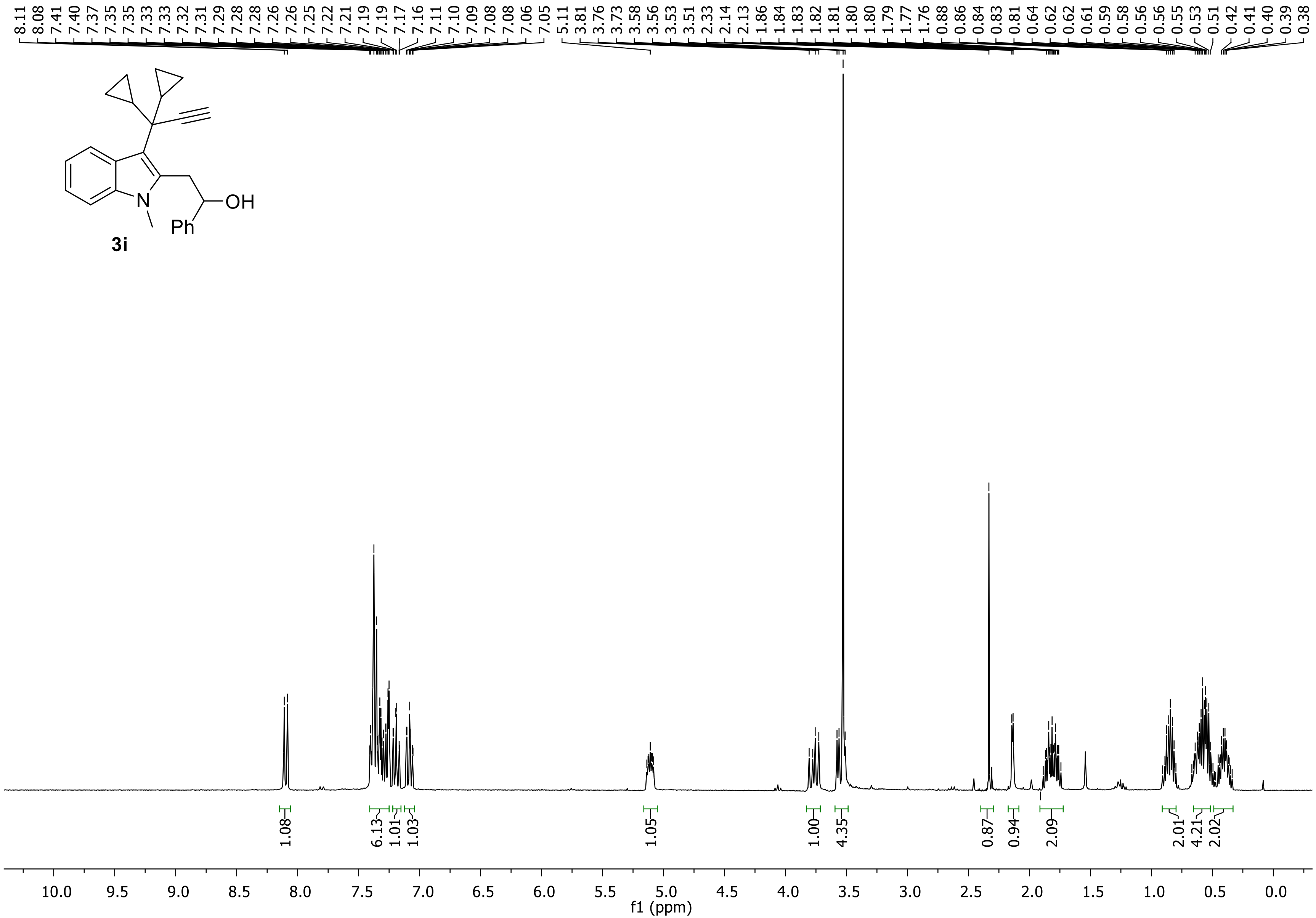

^{13}C NMR ($CDCl_3$, 75.4 MHz)

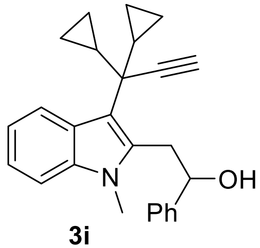

3i

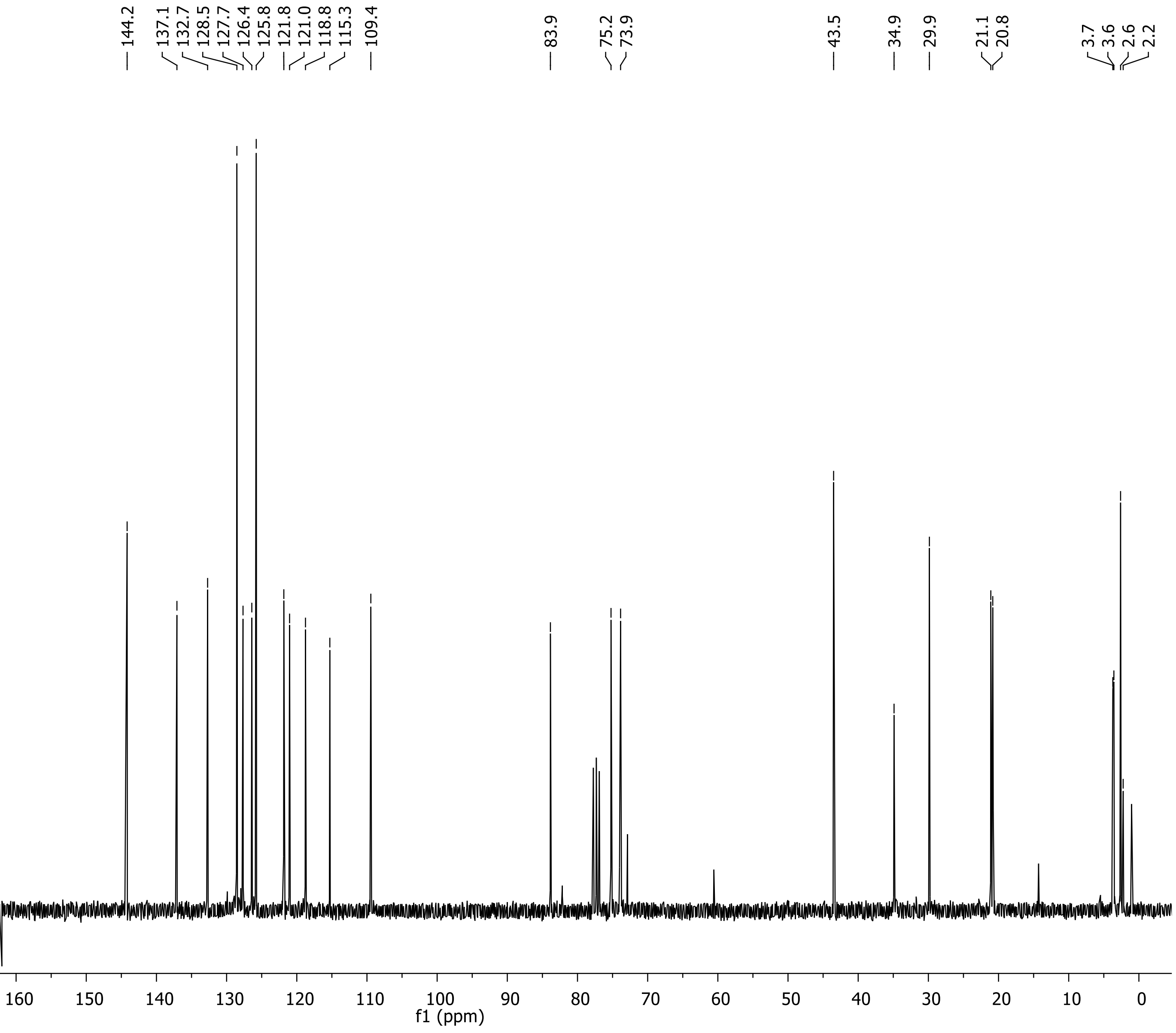

DEPT ($CDCl_3$, 75.4 MHz)

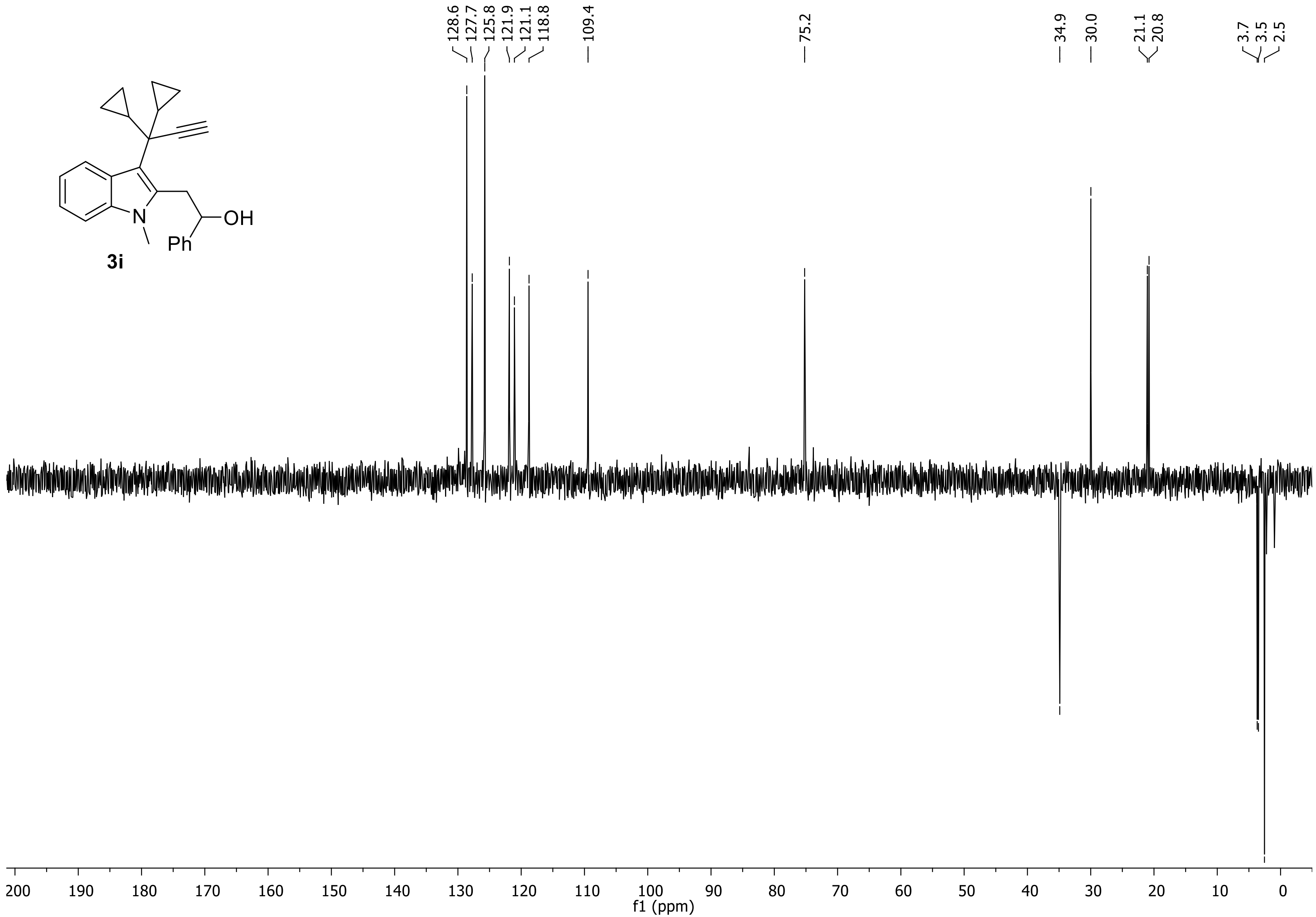

3i

^{1}H NMR ($CDCl_3$, 300 MHz)

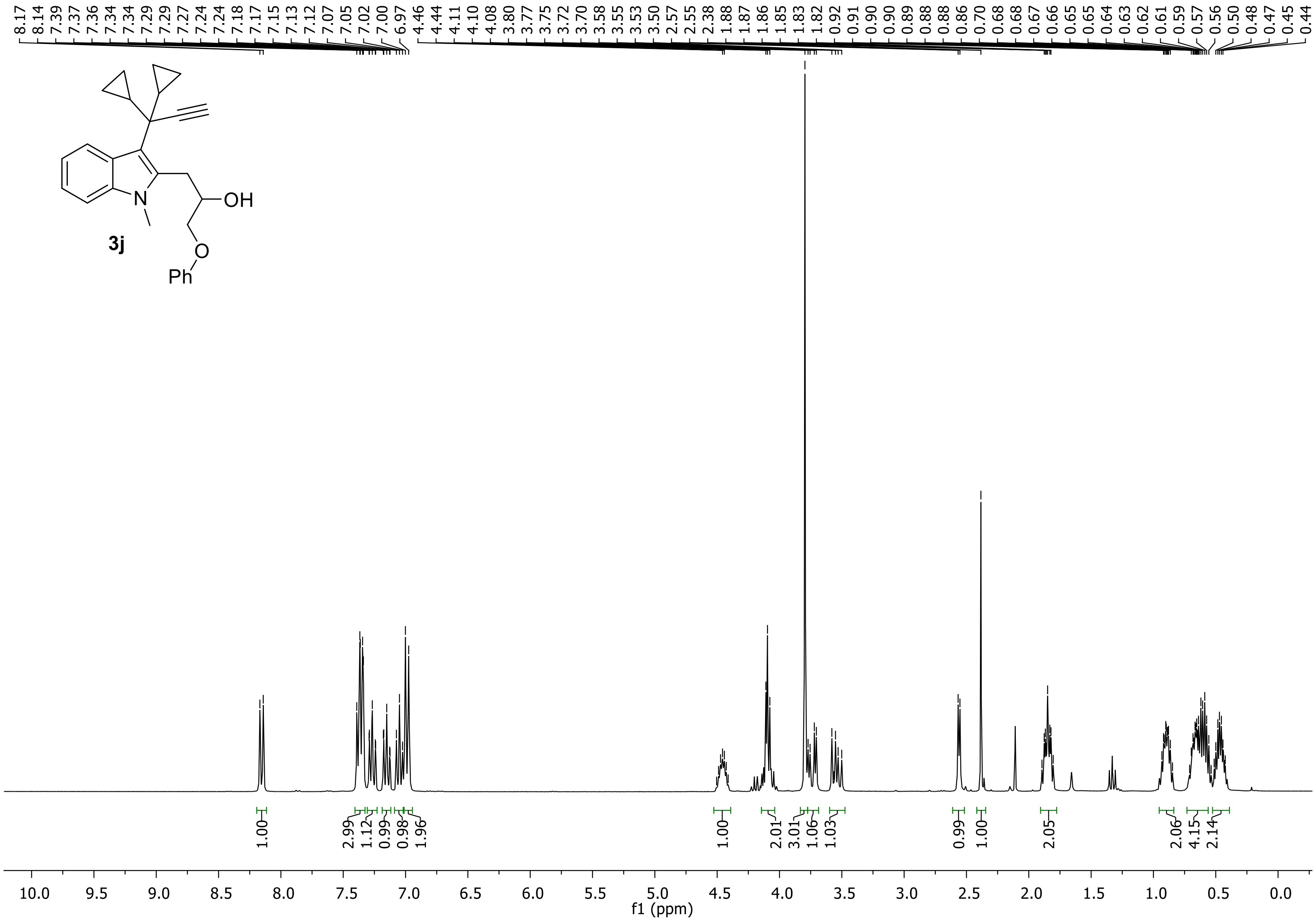

^{13}C NMR ($CDCl_3$, 75.4 MHz)

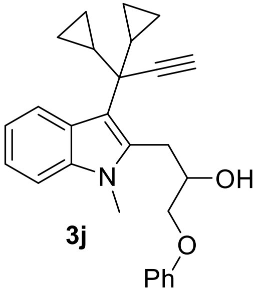

3j

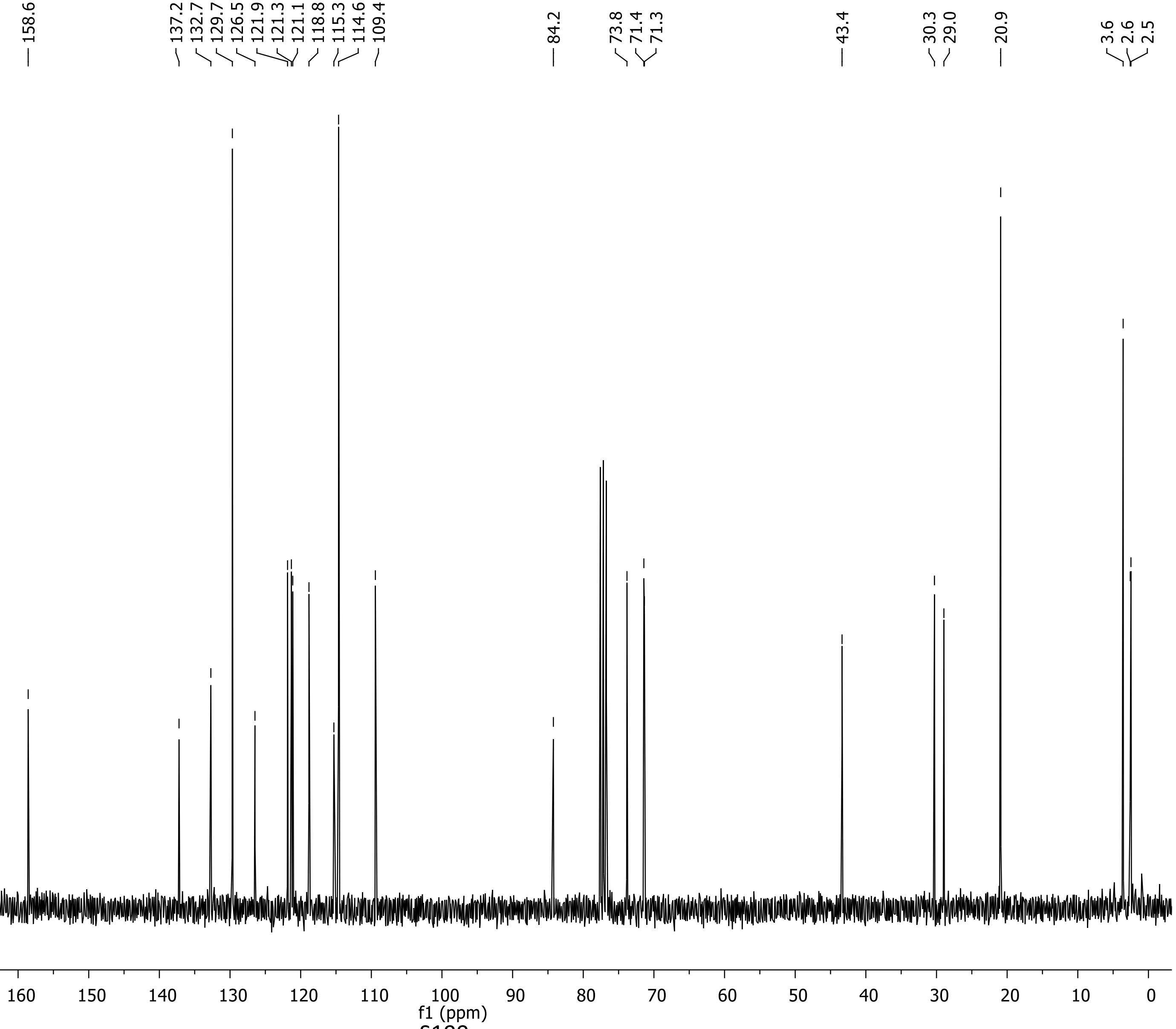

DEPT ($CDCl_3$, 75.4 MHz)

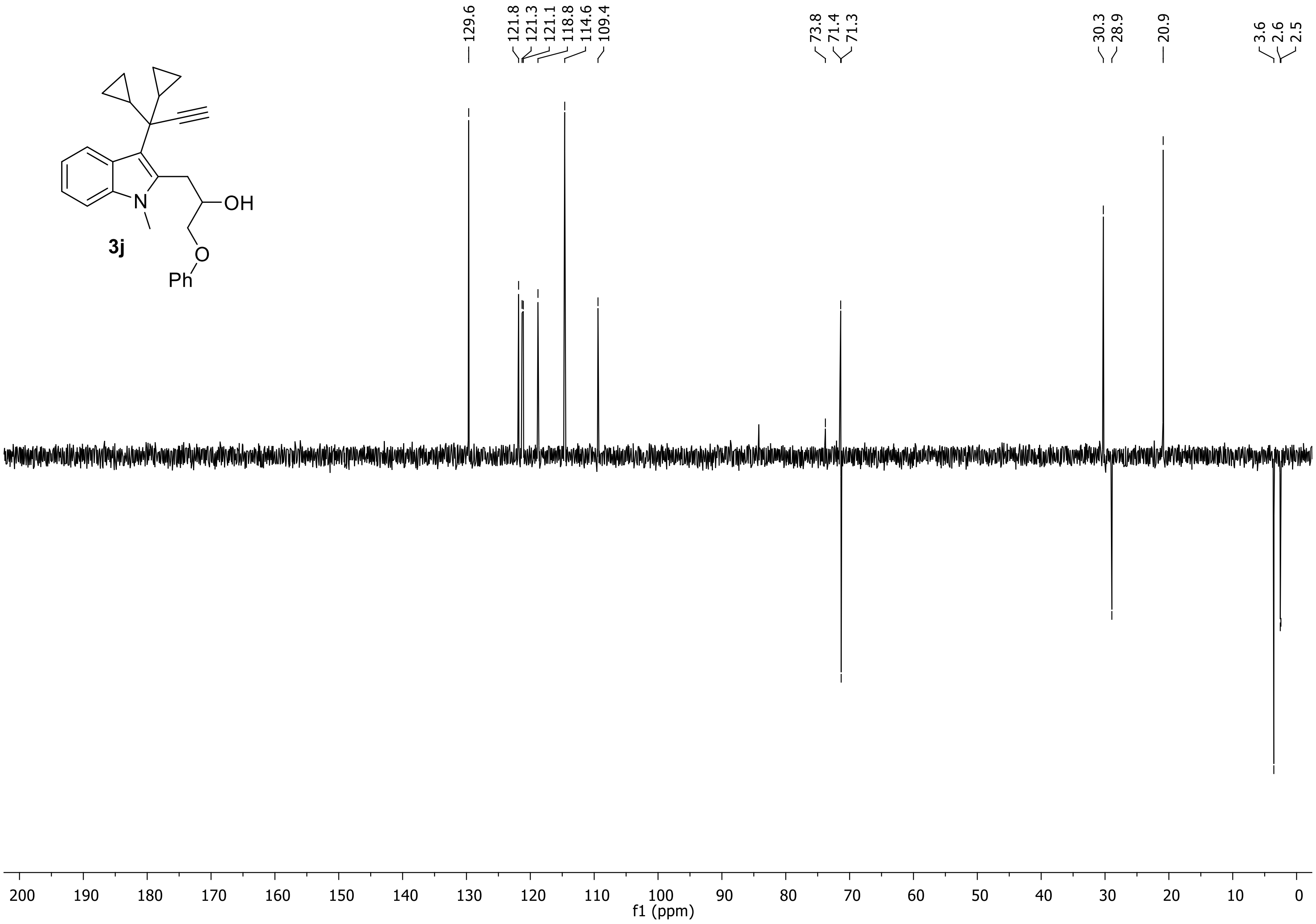

^{1}H NMR ($CDCl_3$, 300 MHz)

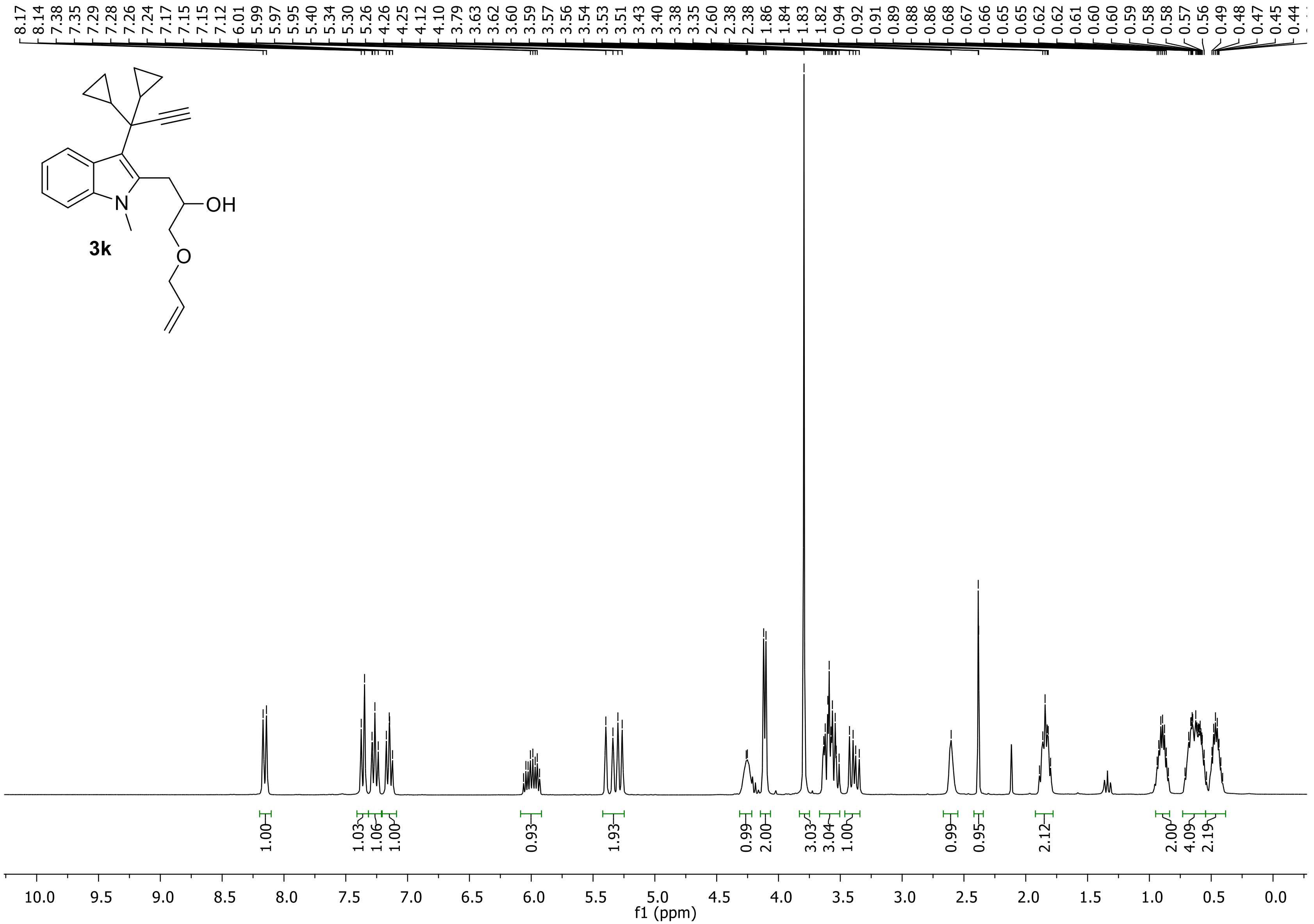

^{13}C NMR ($CDCl_3$, 75.4 MHz)

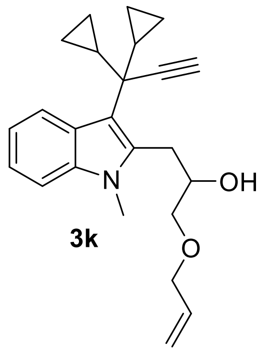

3k

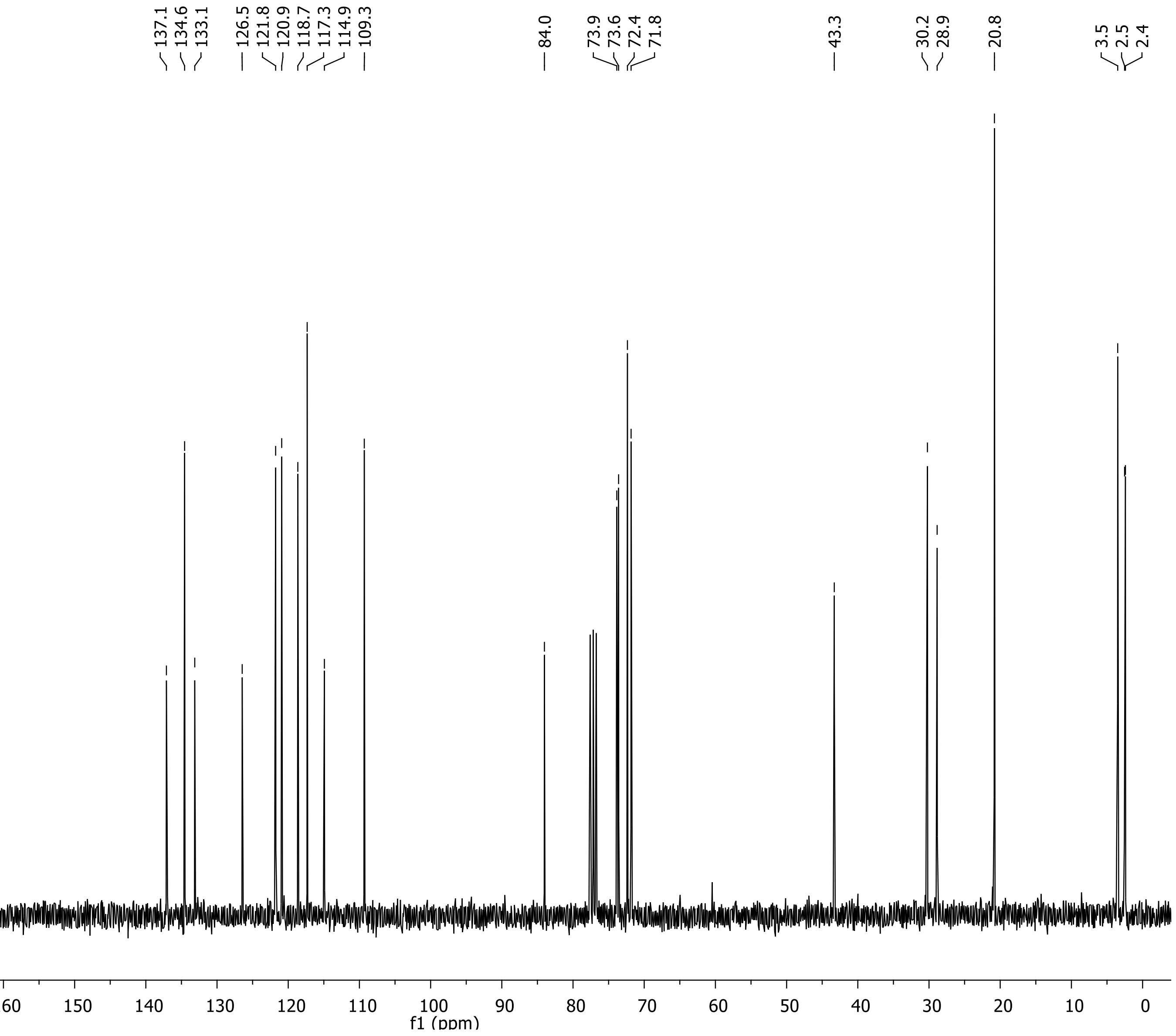

DEPT (CDCl$_3$, 75.4 MHz)

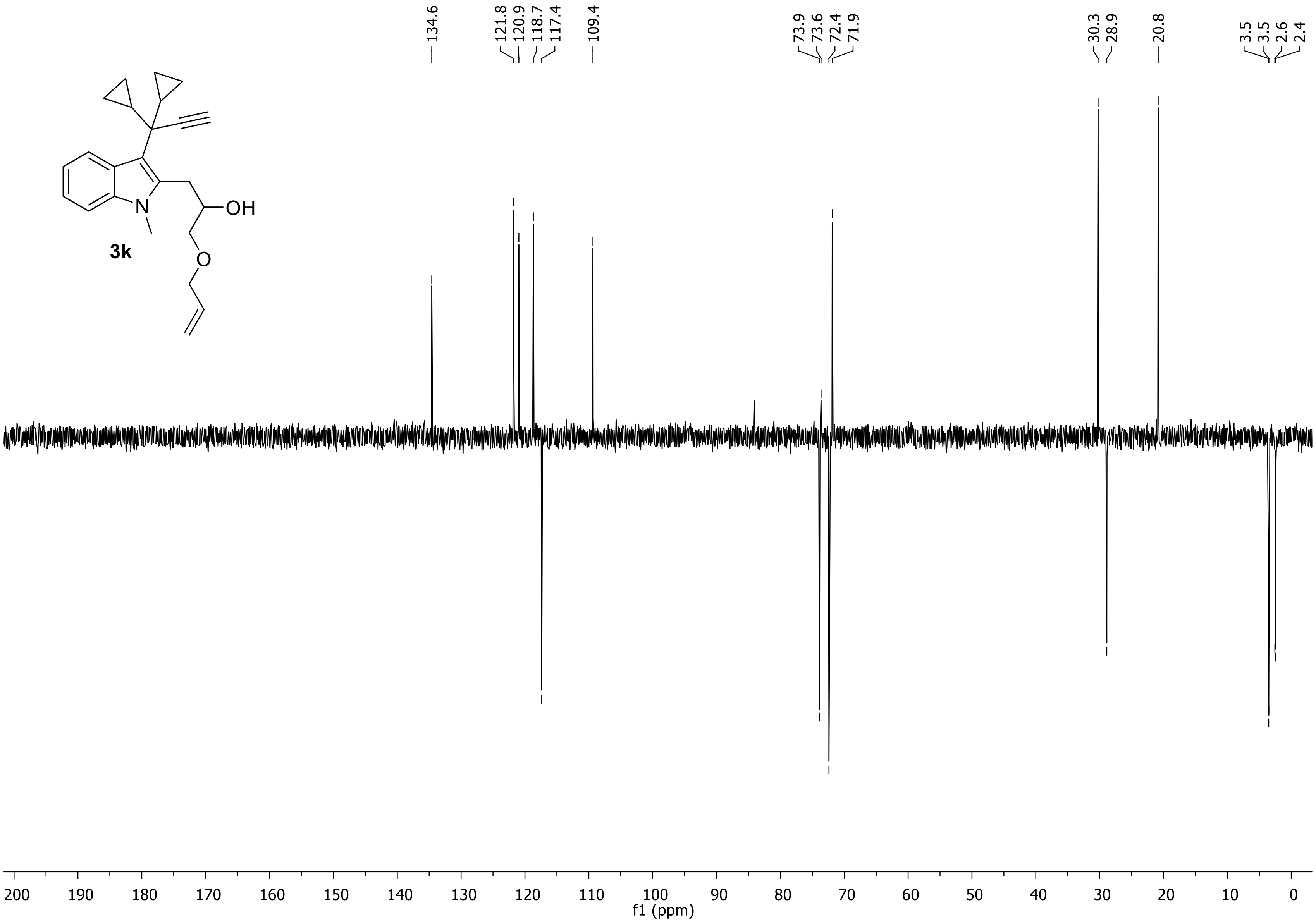

^{1}H NMR ($CDCl_3$, 300 MHz)

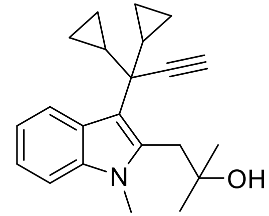

3l

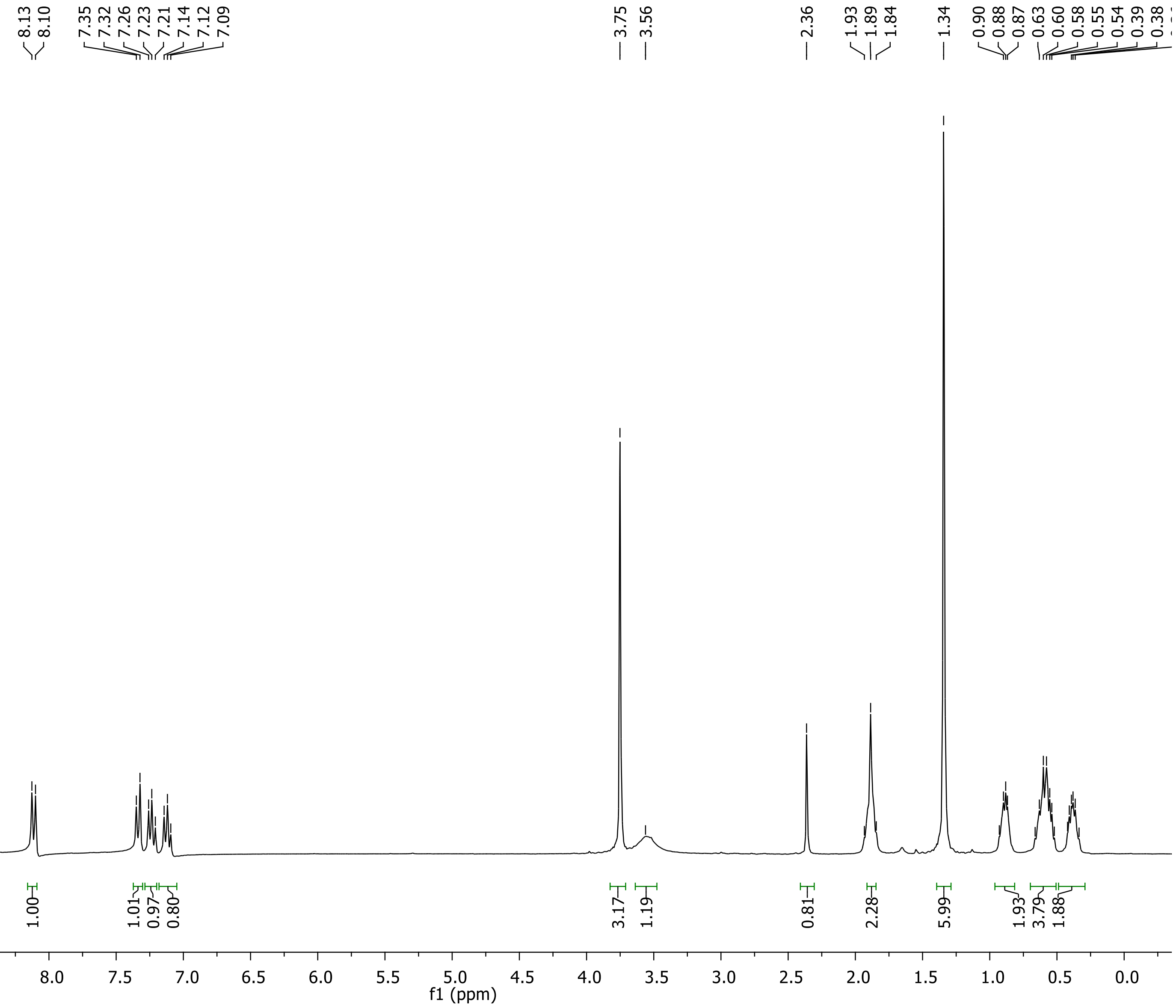

^{13}C NMR ($CDCl_3$, 75.4 MHz)

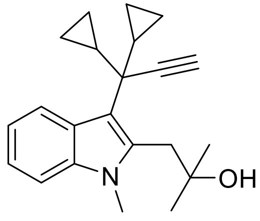

3l

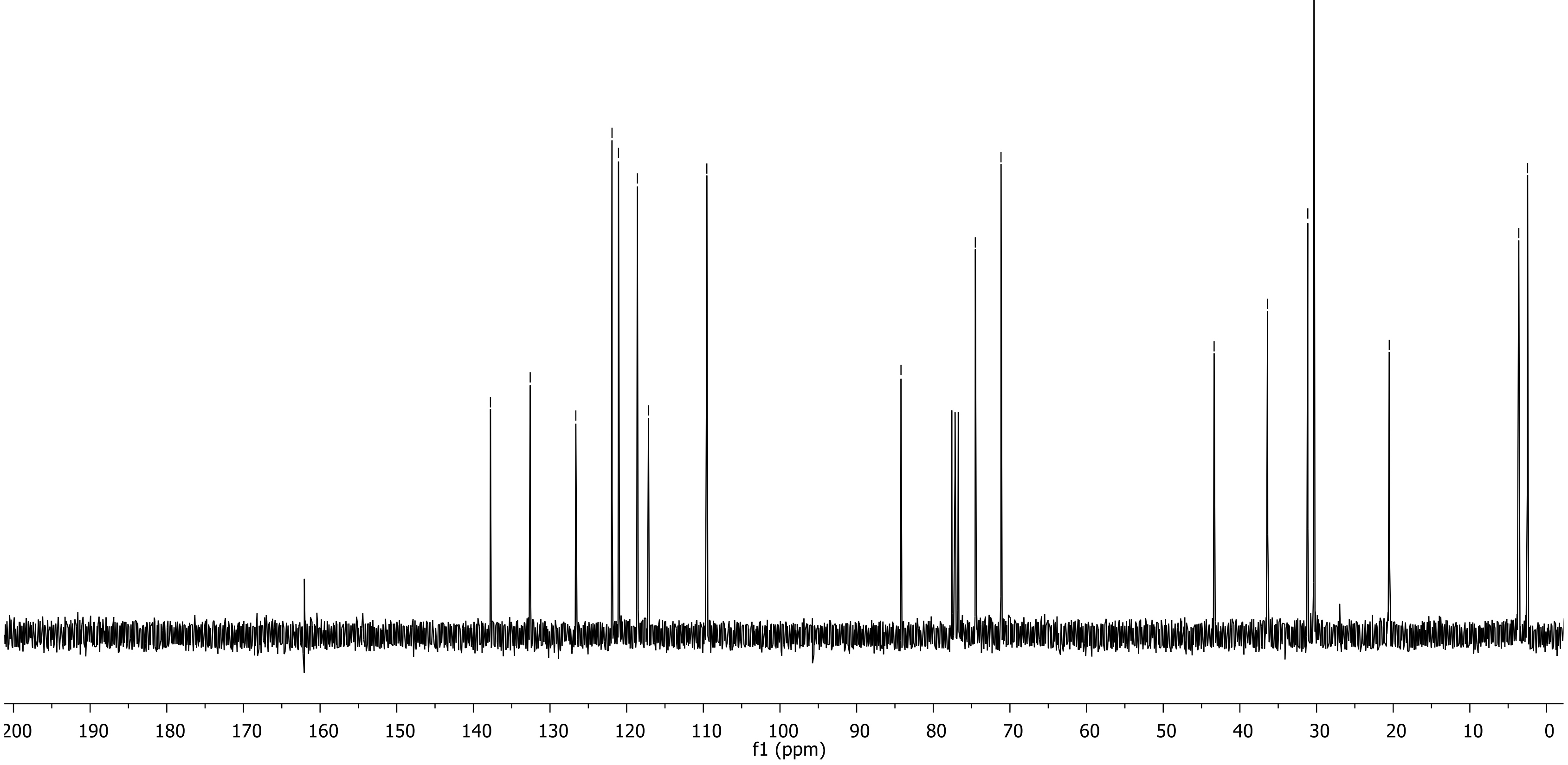

DEPT ($CDCl_3$, 75.4 MHz)

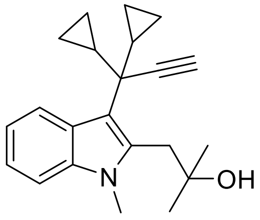

3l

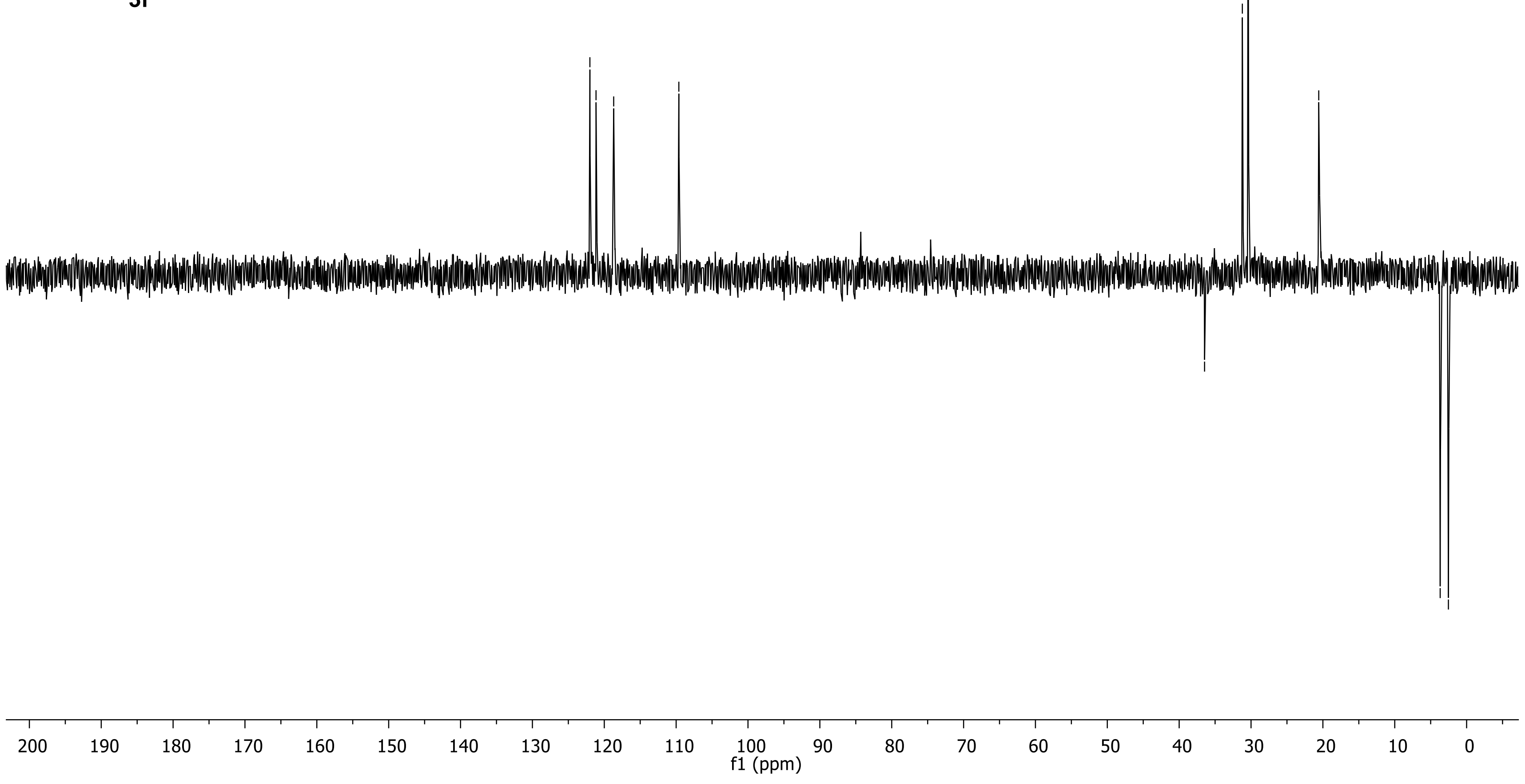

^{1}H NMR ($CDCl_3$, 300 MHz)

3m

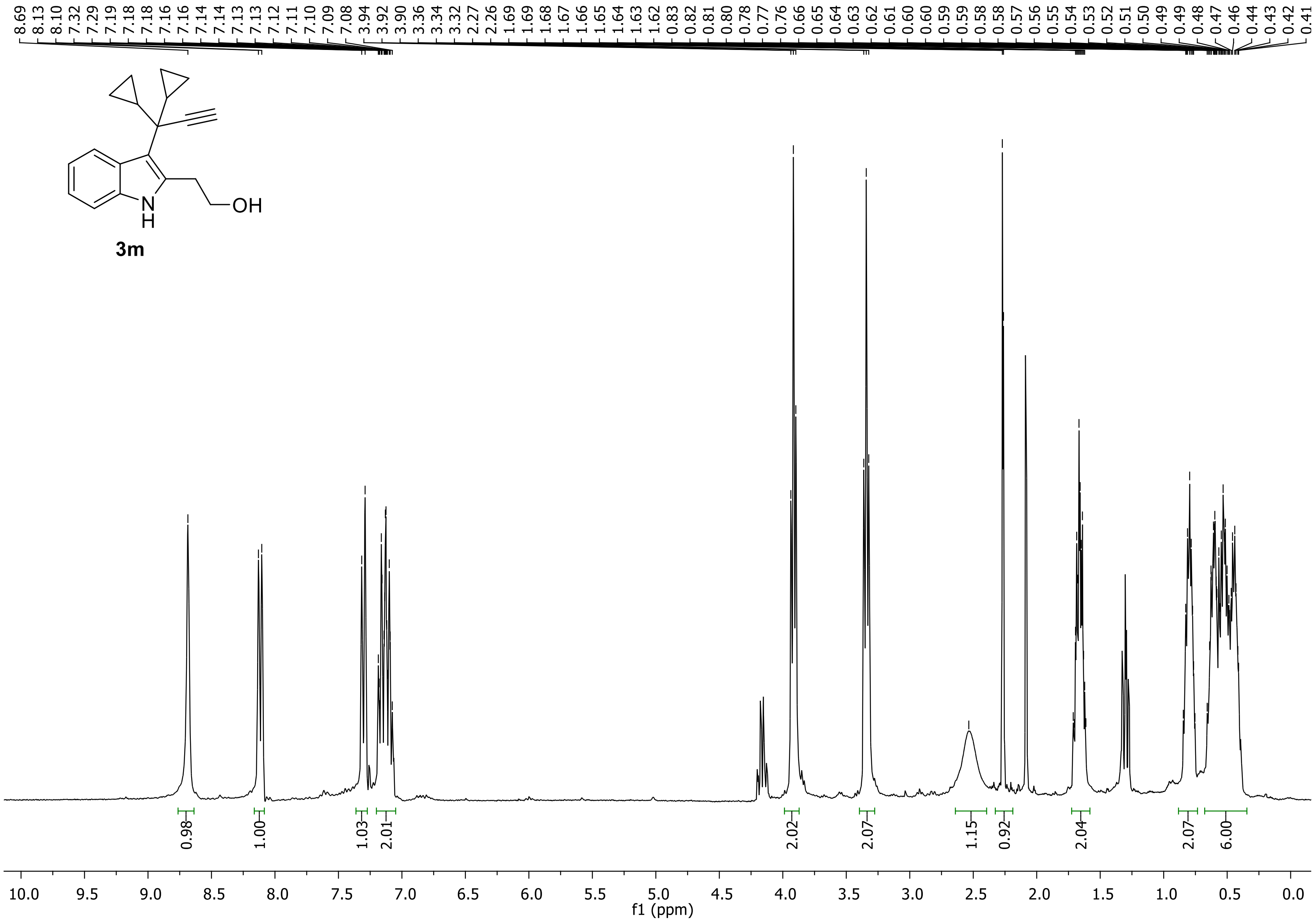

^{13}C NMR ($CDCl_3$, 75.4 MHz)

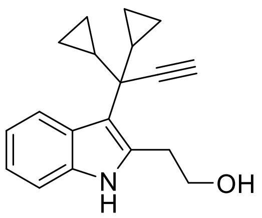

3m

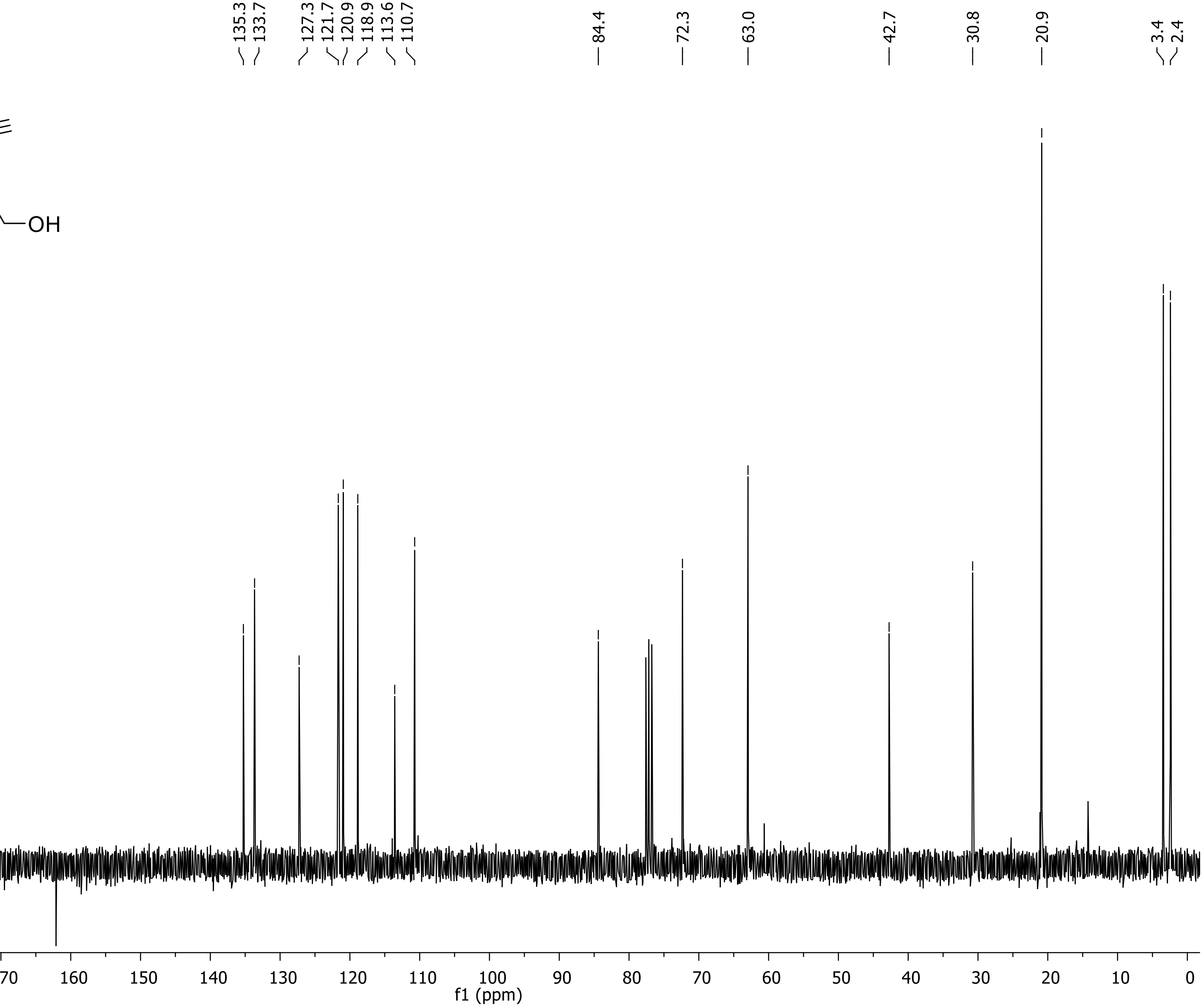

^{1}H NMR ($CDCl_3$, 300 MHz)

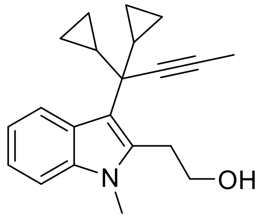

3n

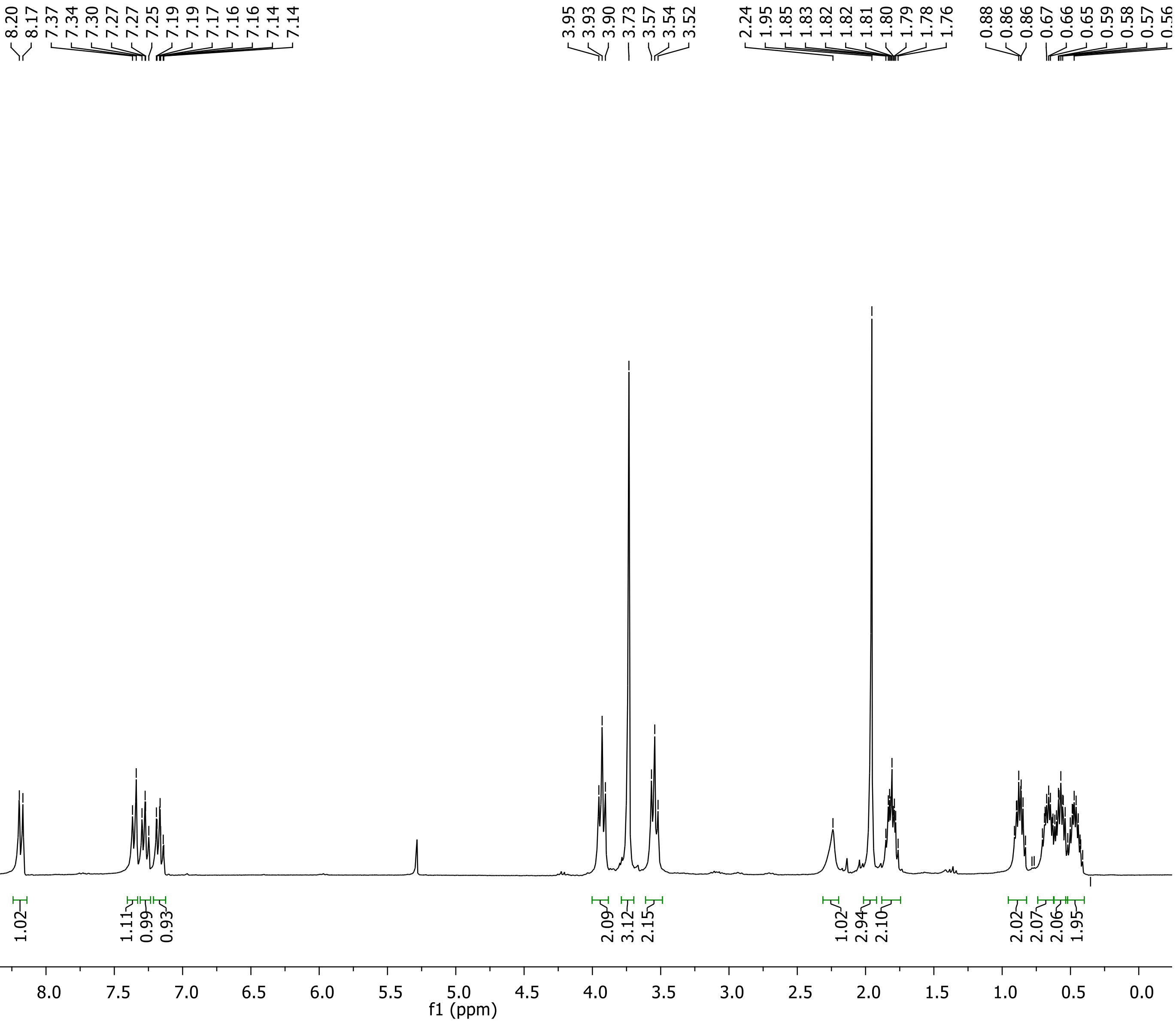

^{13}C NMR ($CDCl_3$, 75.4 MHz)

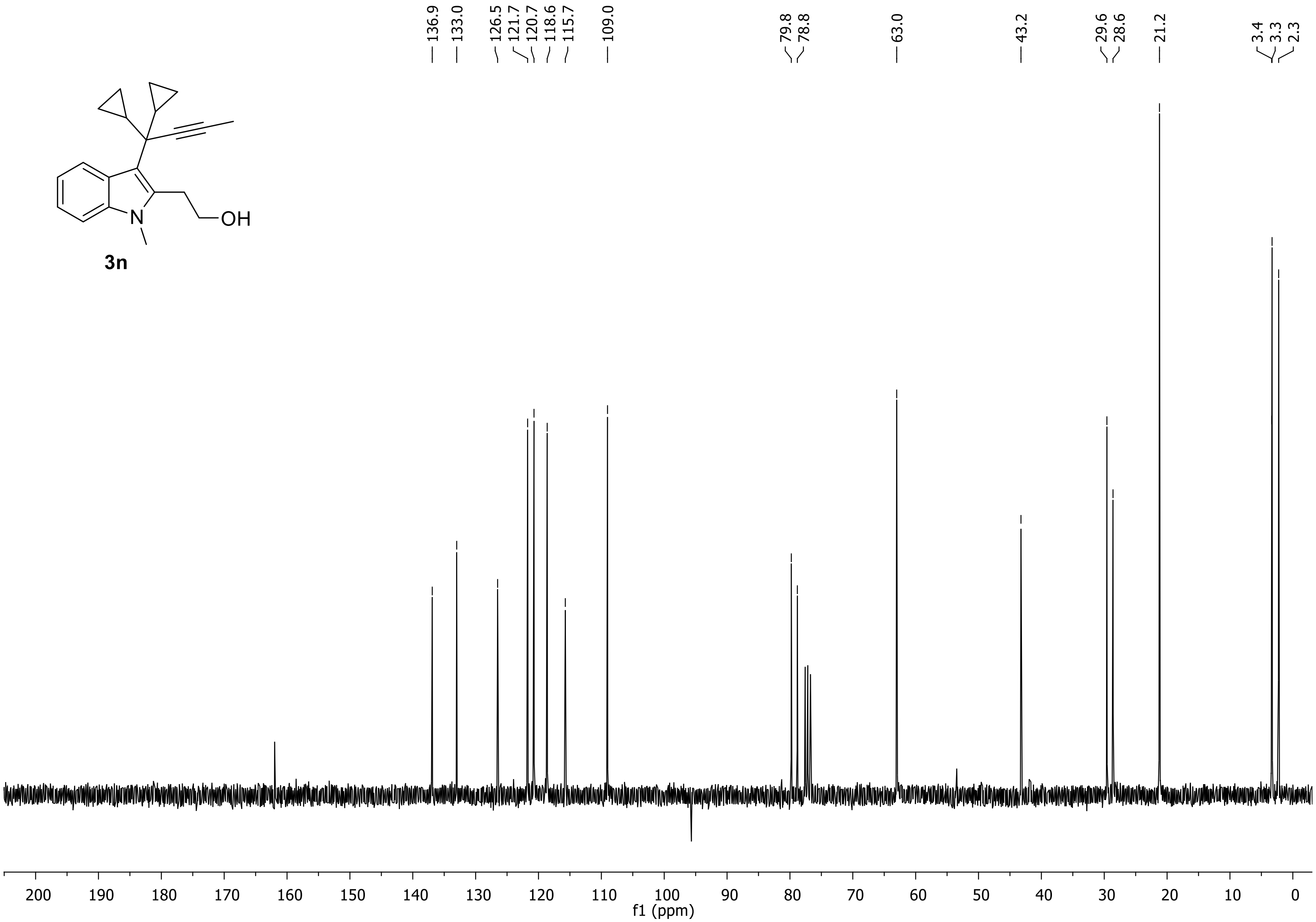

DEPT ($CDCl_3$, 75.4 MHz)

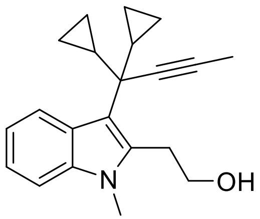

3n

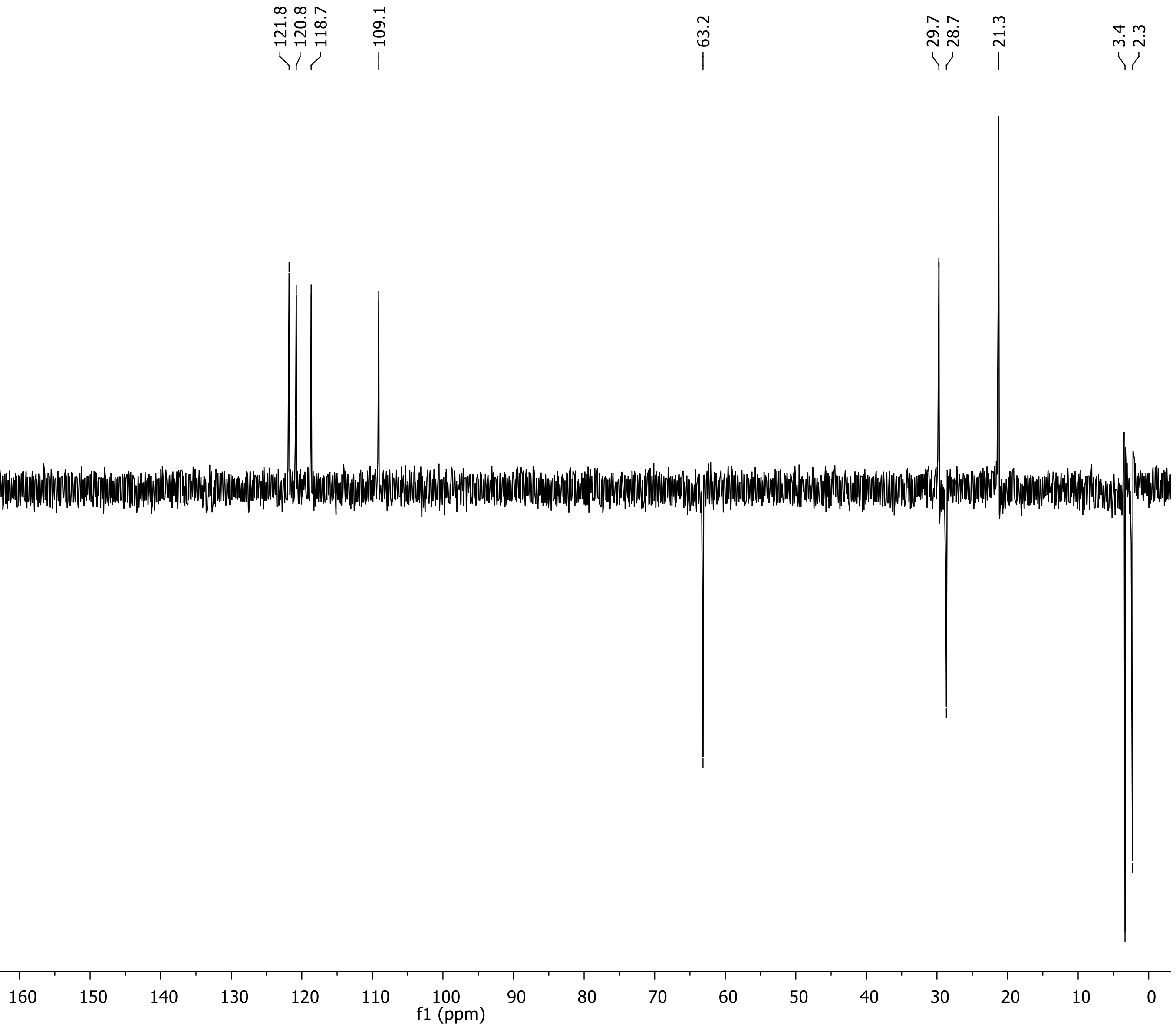

^{1}H NMR ($CDCl_3$, 300 MHz)

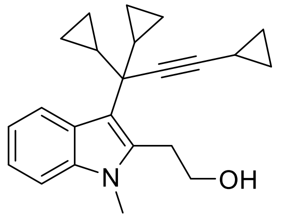

3o

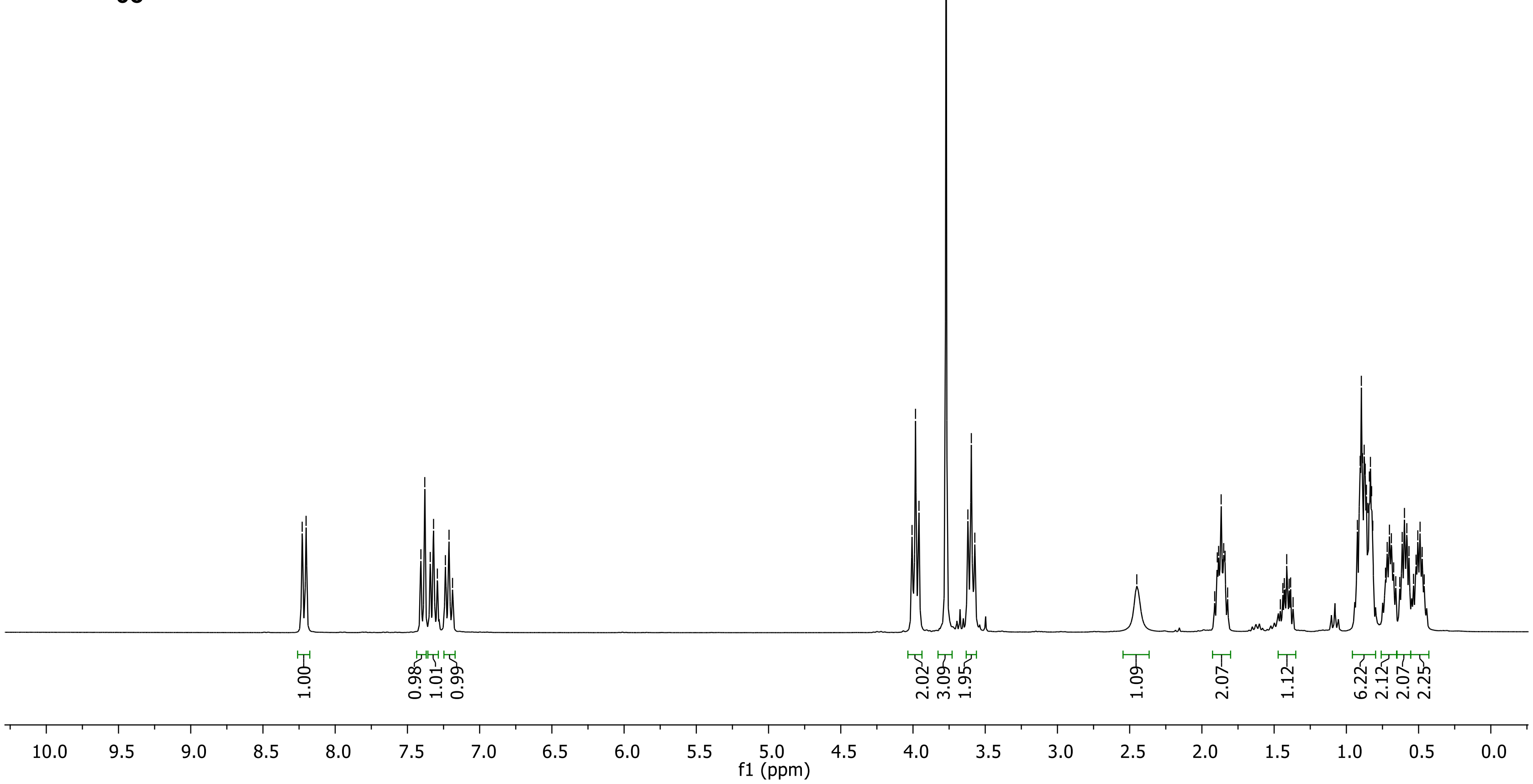

^{13}C NMR ($CDCl_3$, 75.4 MHz)

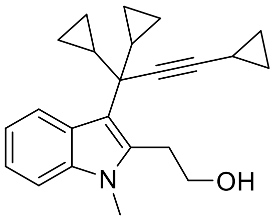

3o

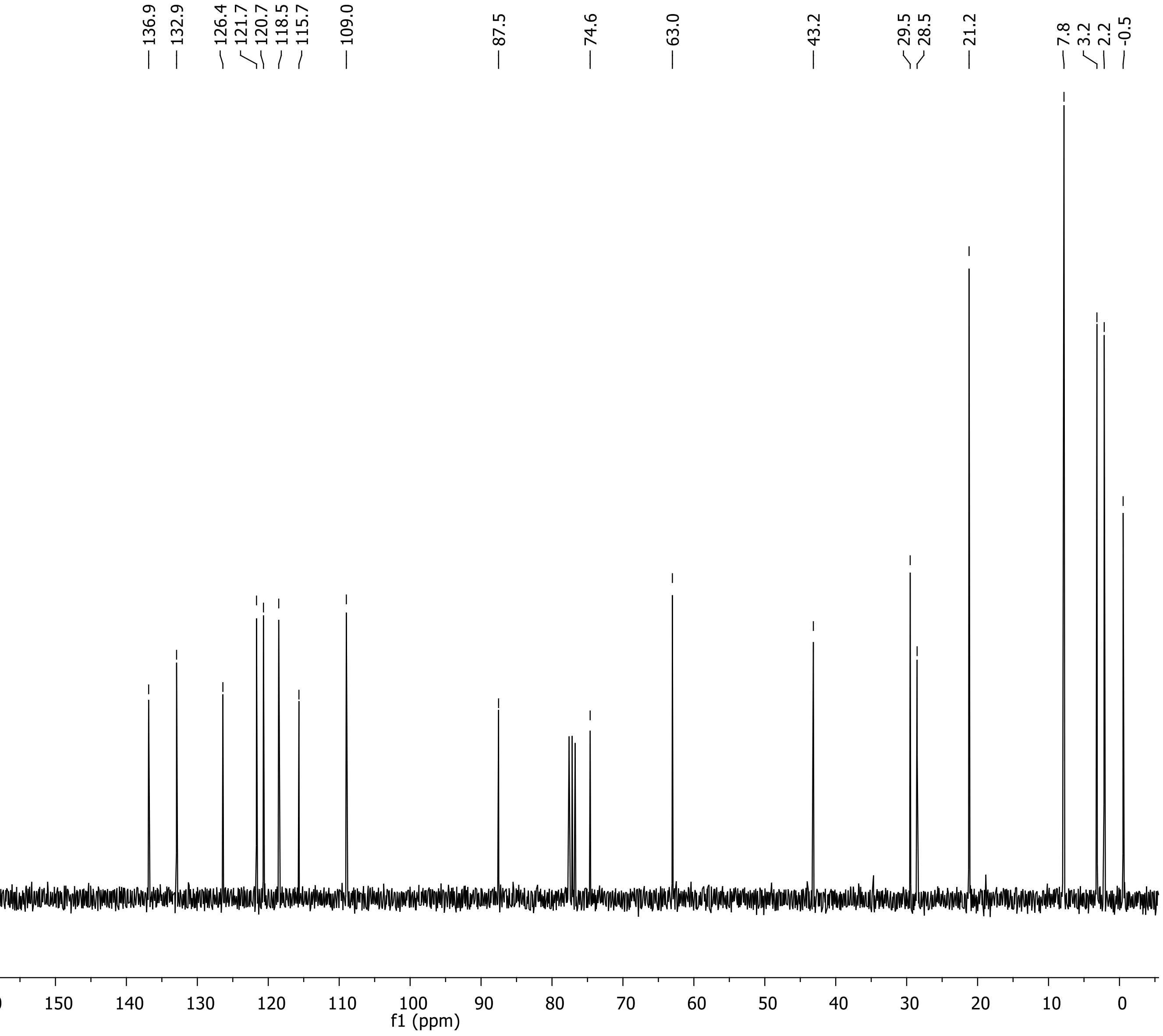

DEPT ($CDCl_3$, 75.4 MHz)

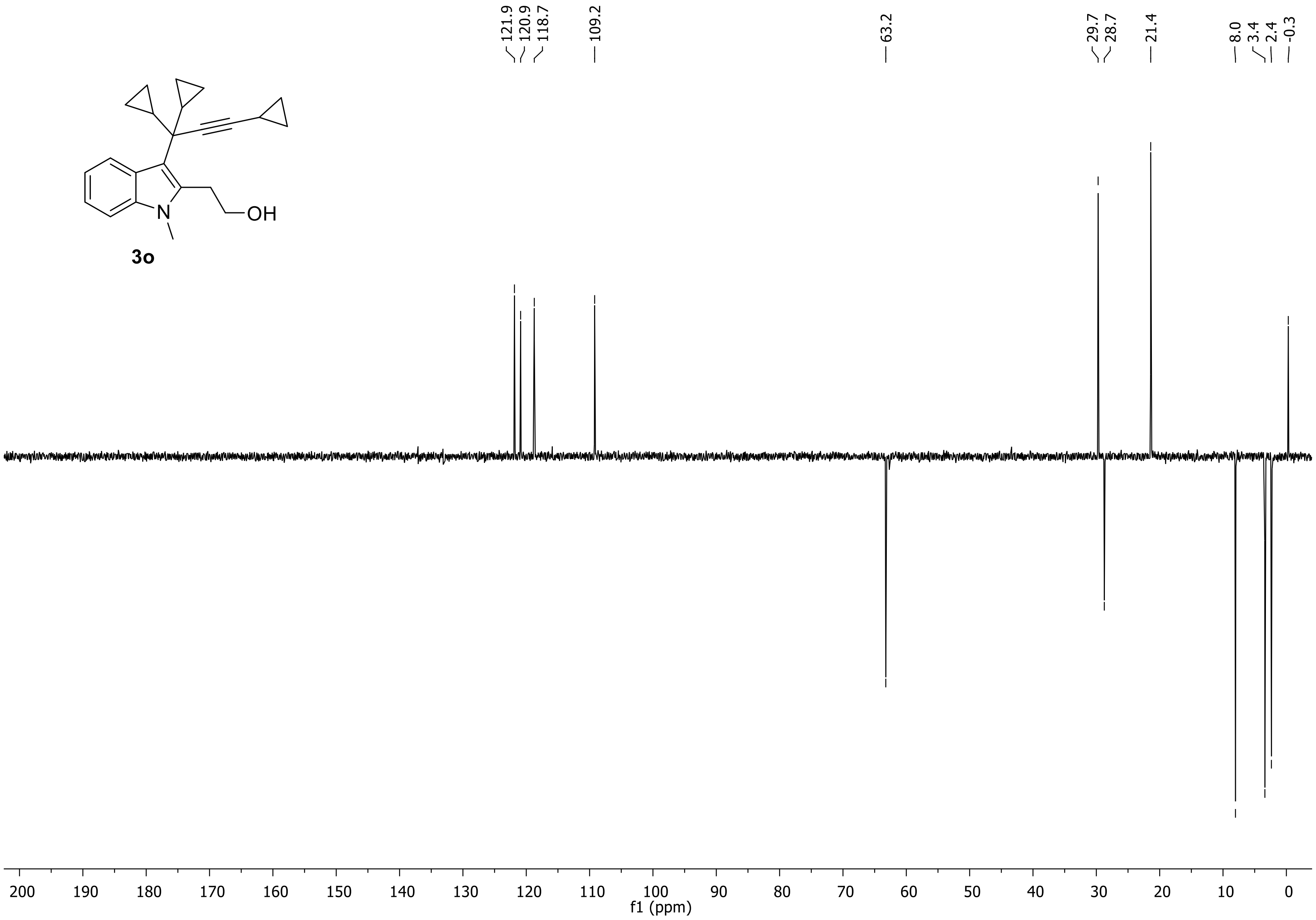

^{1}H NMR ($CDCl_3$, 300 MHz)

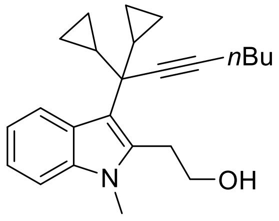

3p

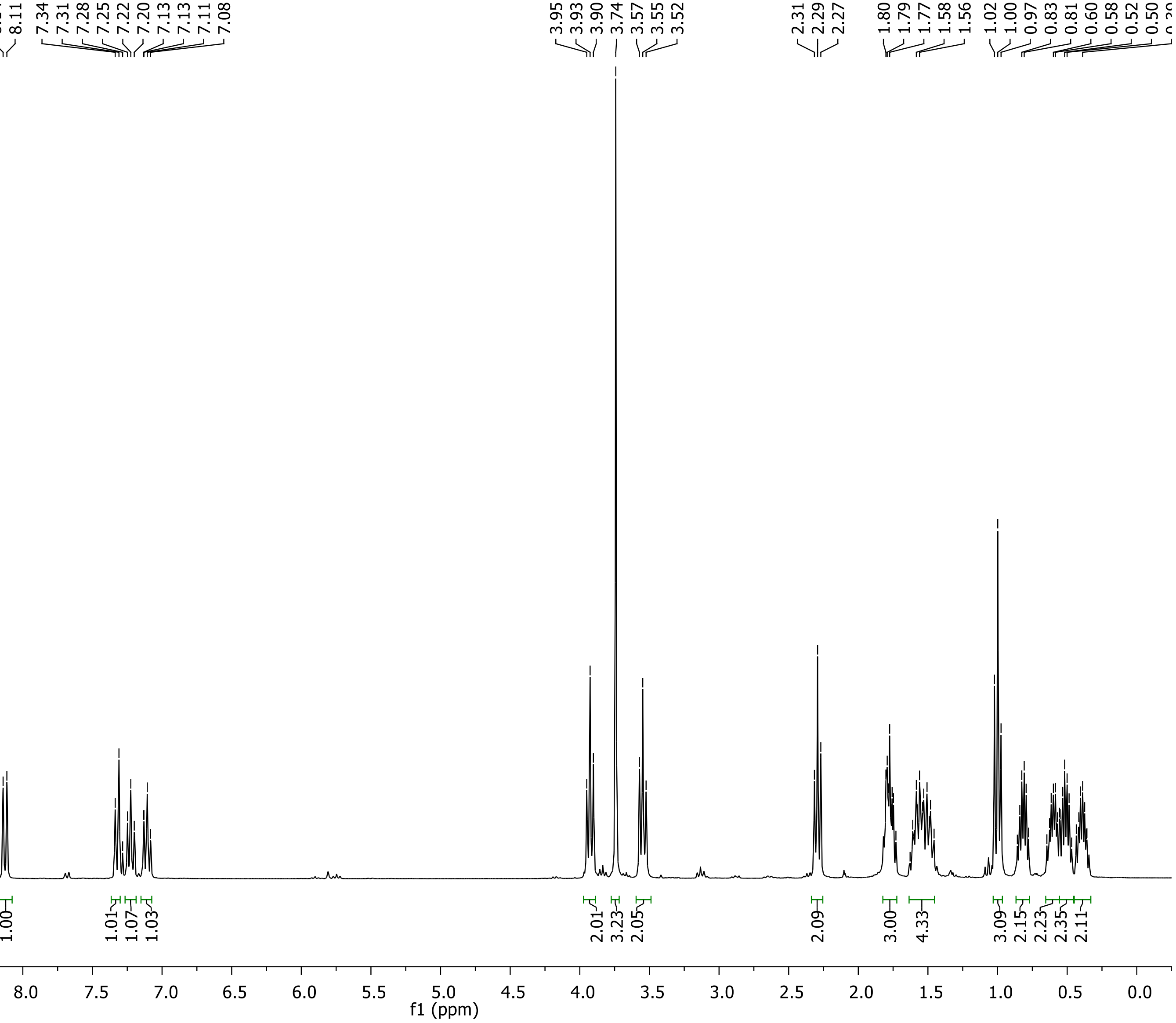

^{13}C NMR ($CDCl_3$, 75.4 MHz)

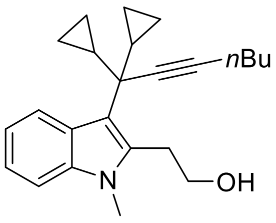

3p

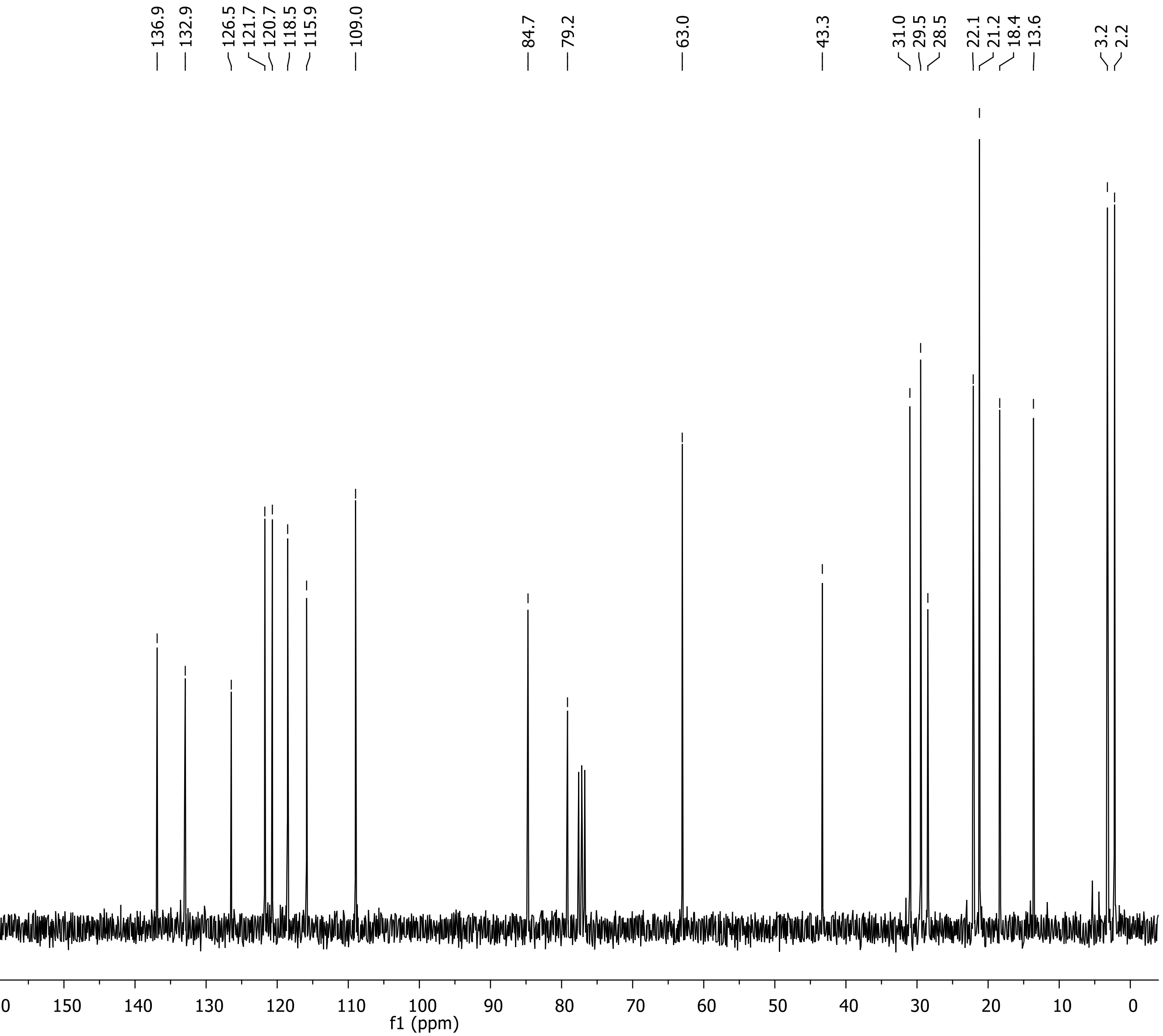

DEPT ($CDCl_3$, 75.4 MHz)

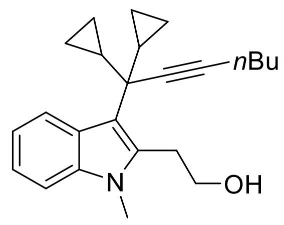

3p

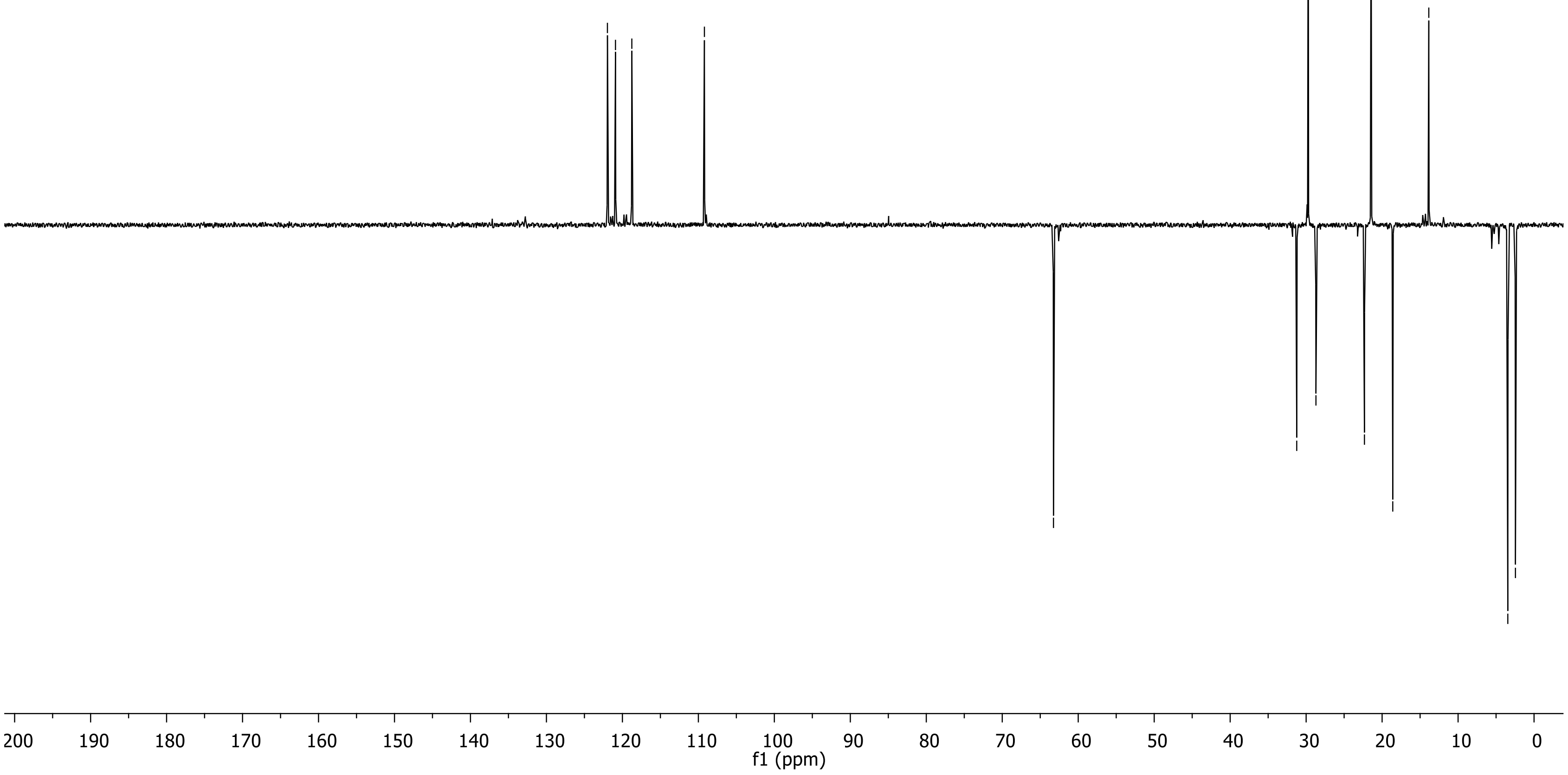

^{1}H NMR ($CDCl_3$, 300 MHz)

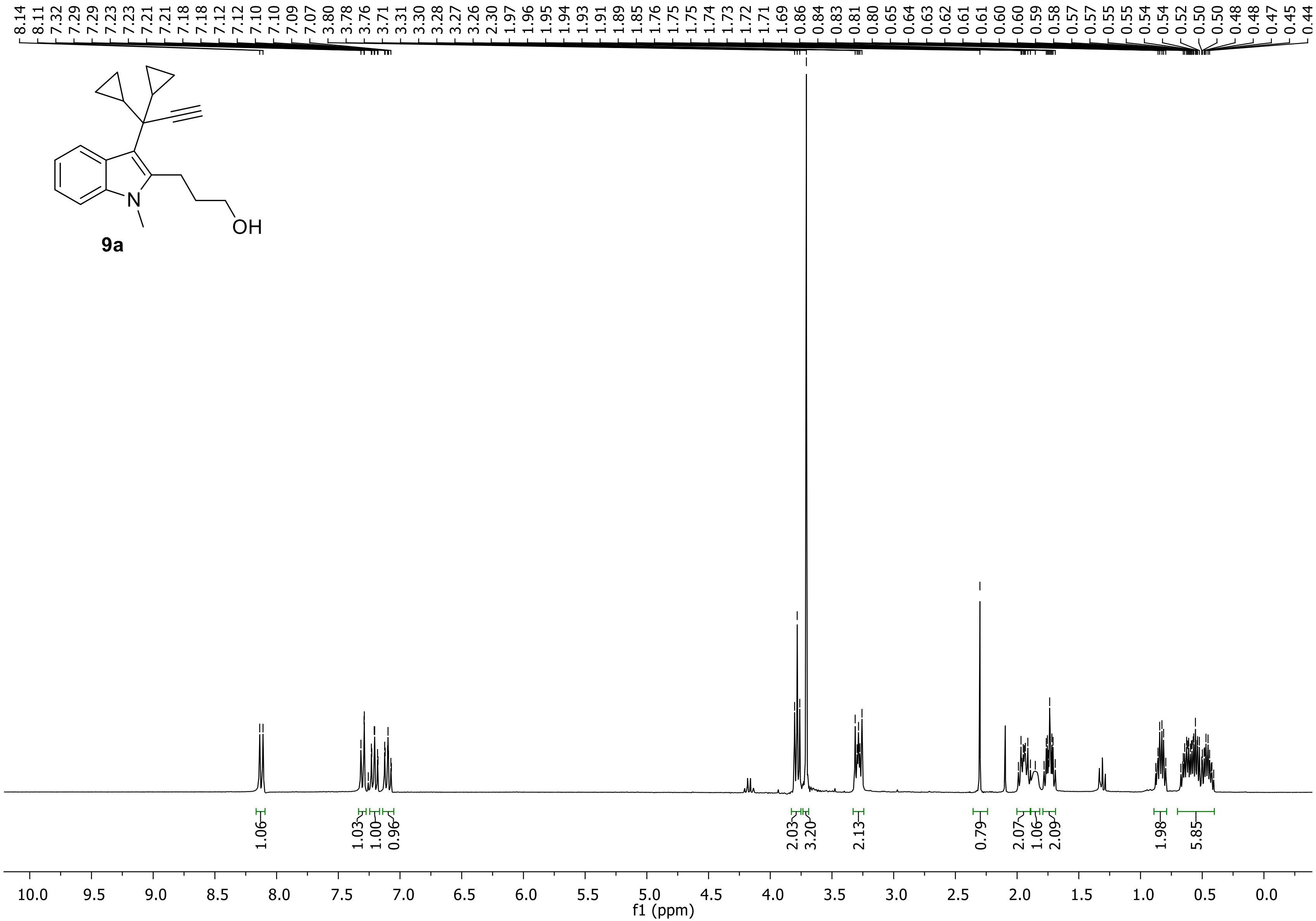

^{13}C NMR ($CDCl_3$, 75.4 MHz)

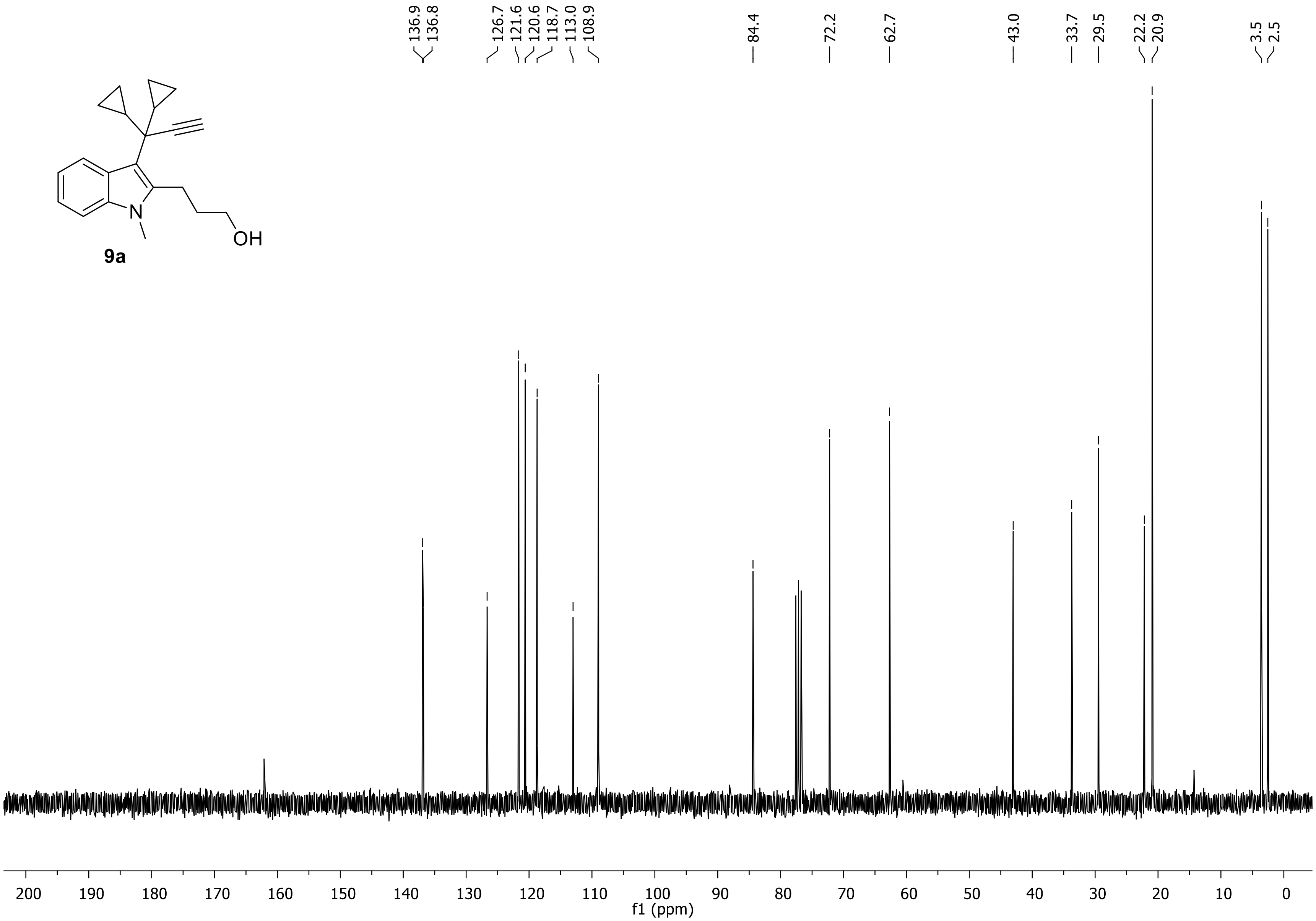

DEPT ($CDCl_3$, 75.4 MHz)

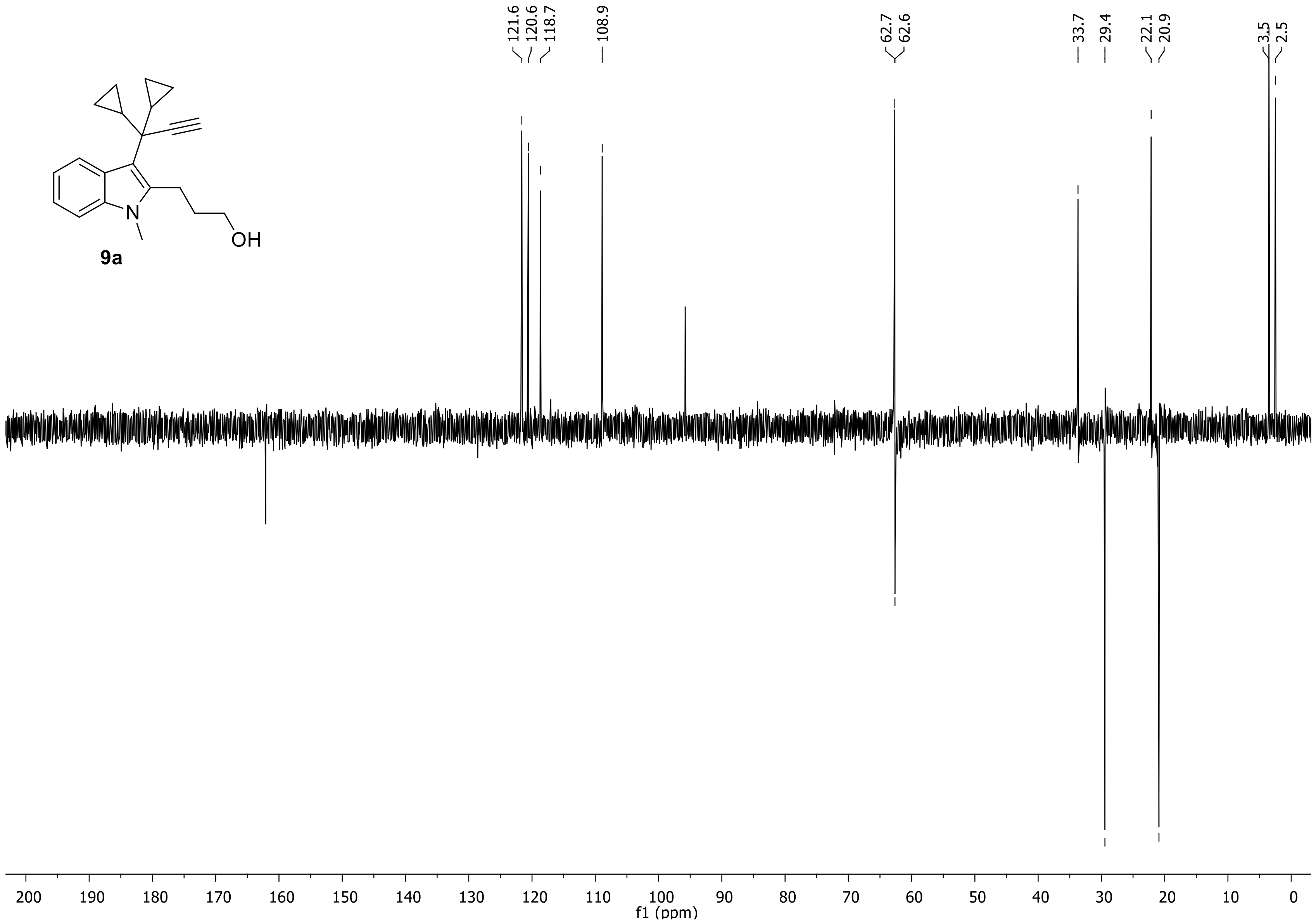

^{1}H NMR ($CDCl_3$, 300 MHz)

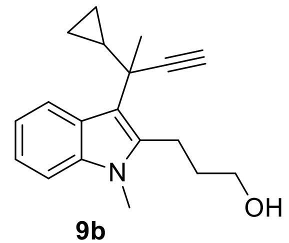

9b

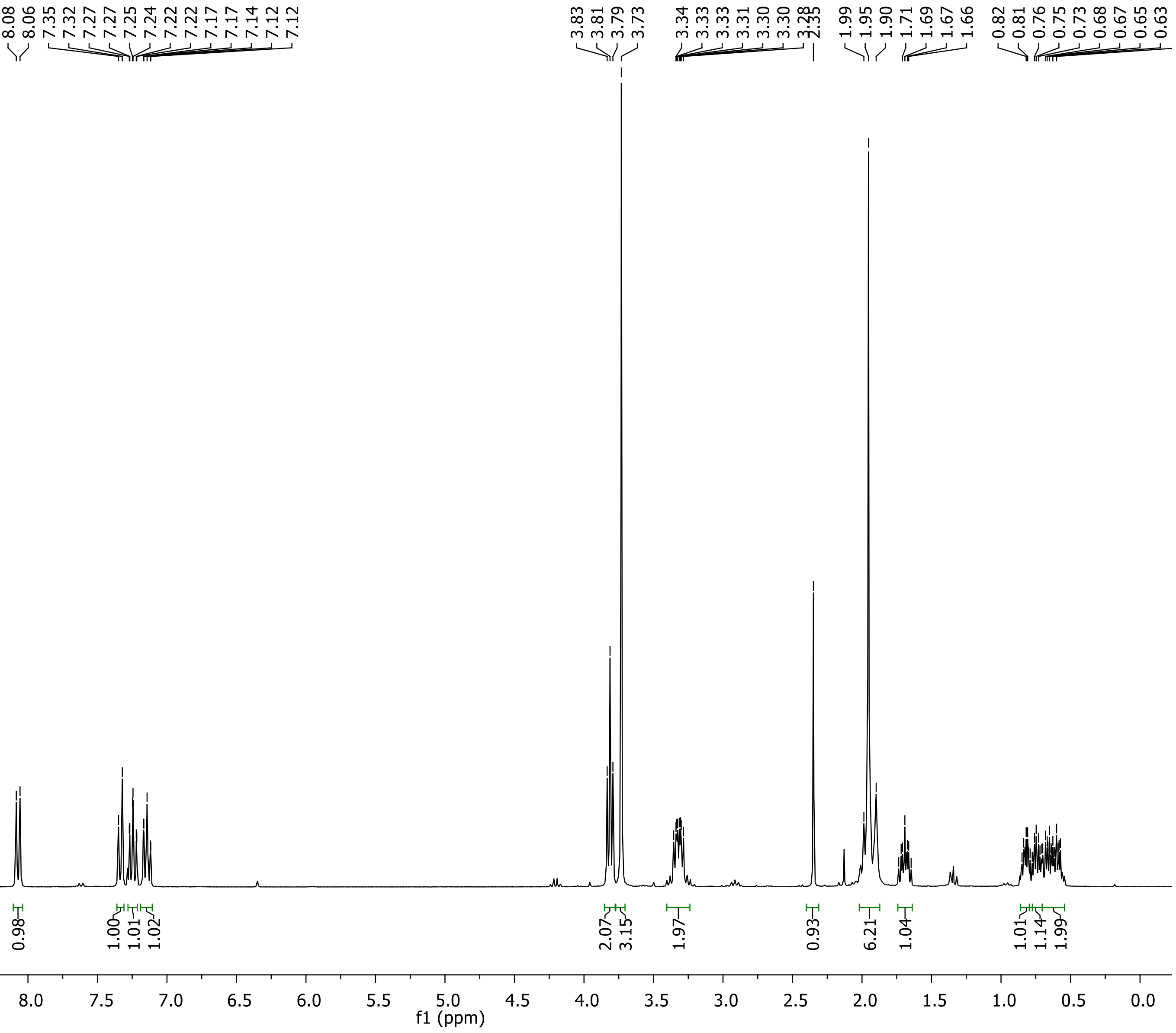

^{13}C NMR ($CDCl_3$, 75.4 MHz)

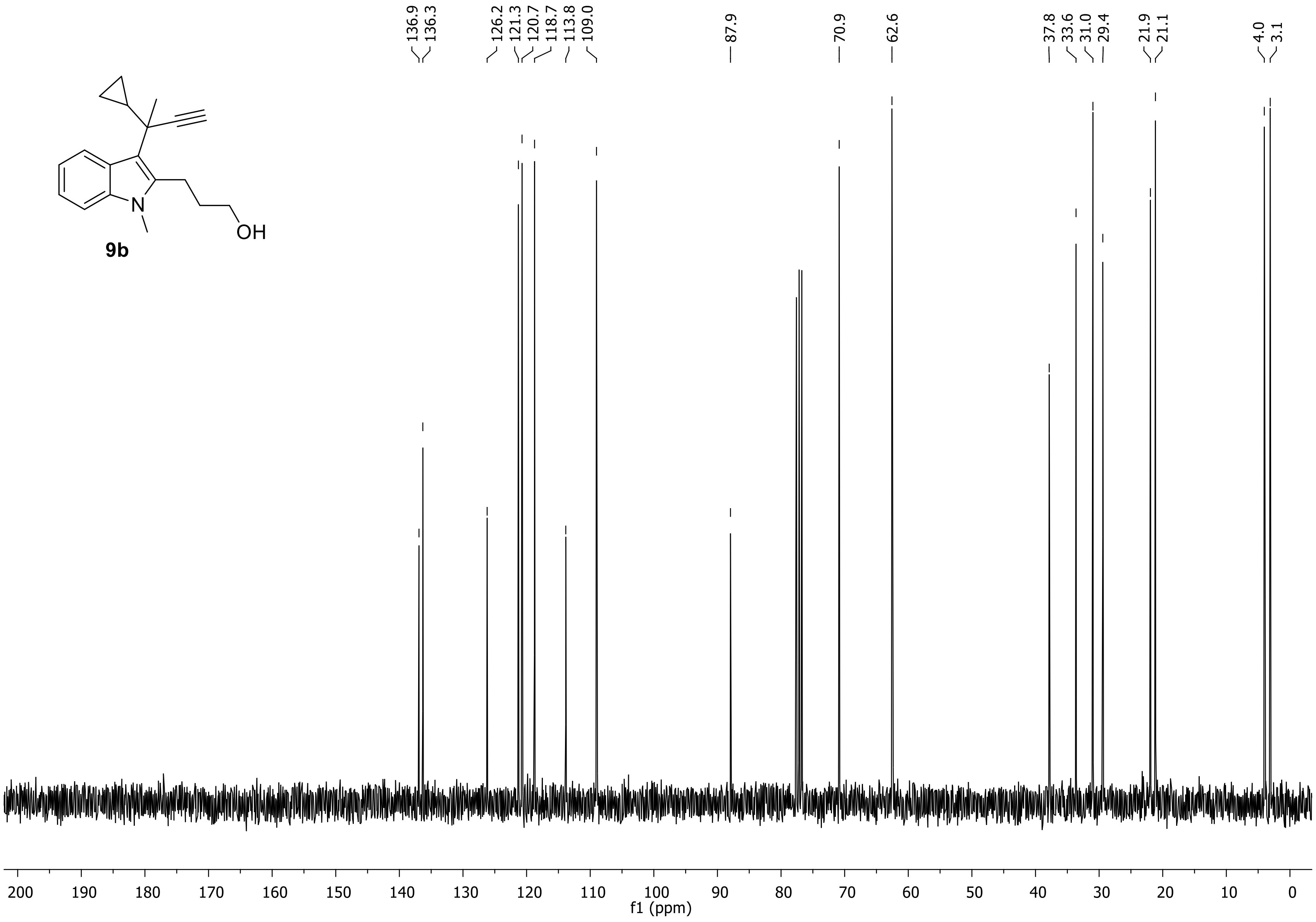

DEPT ($CDCl_3$, 75.4 MHz)

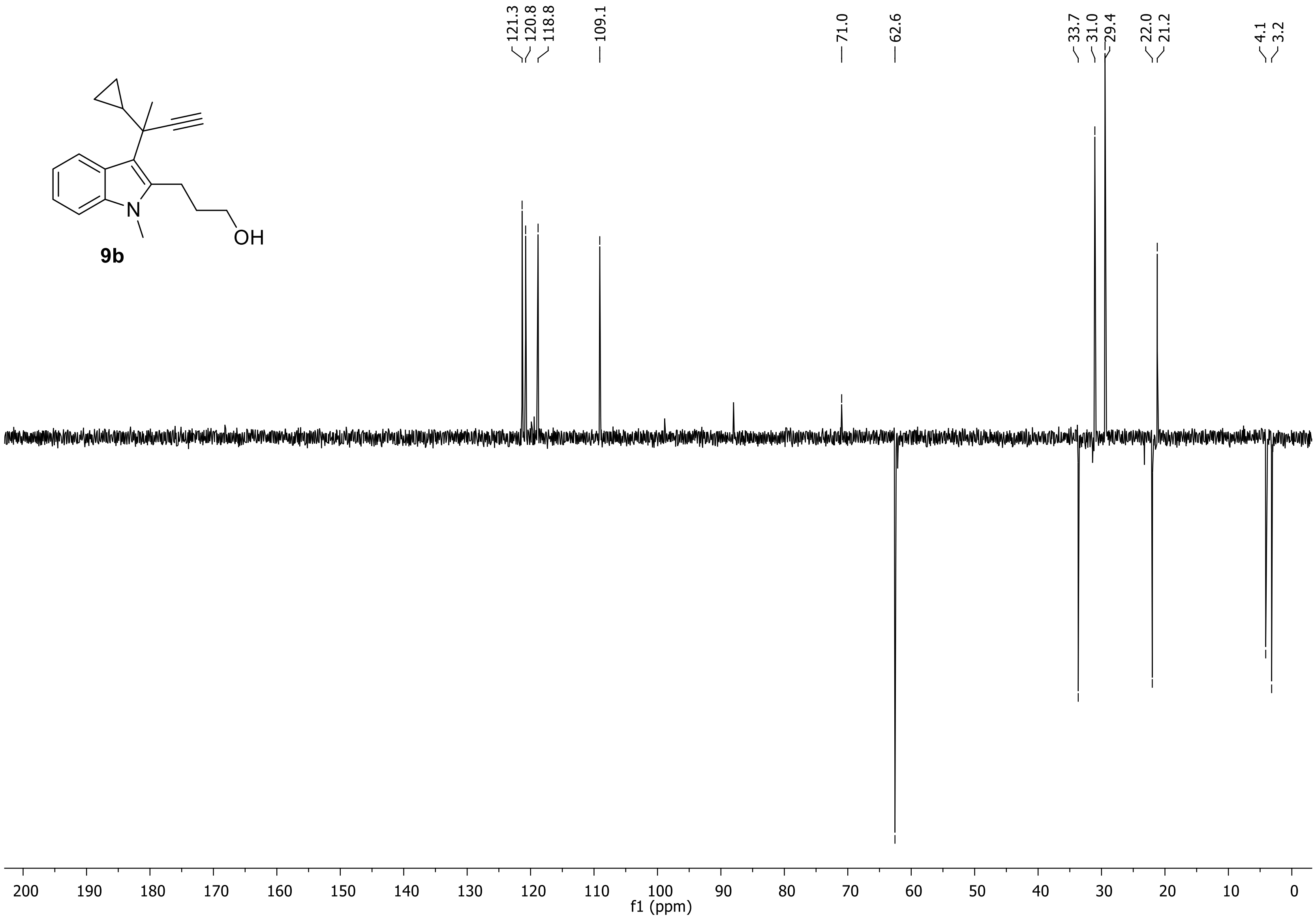

^{1}H NMR ($CDCl_3$, 300 MHz)

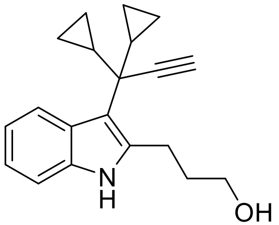

9c

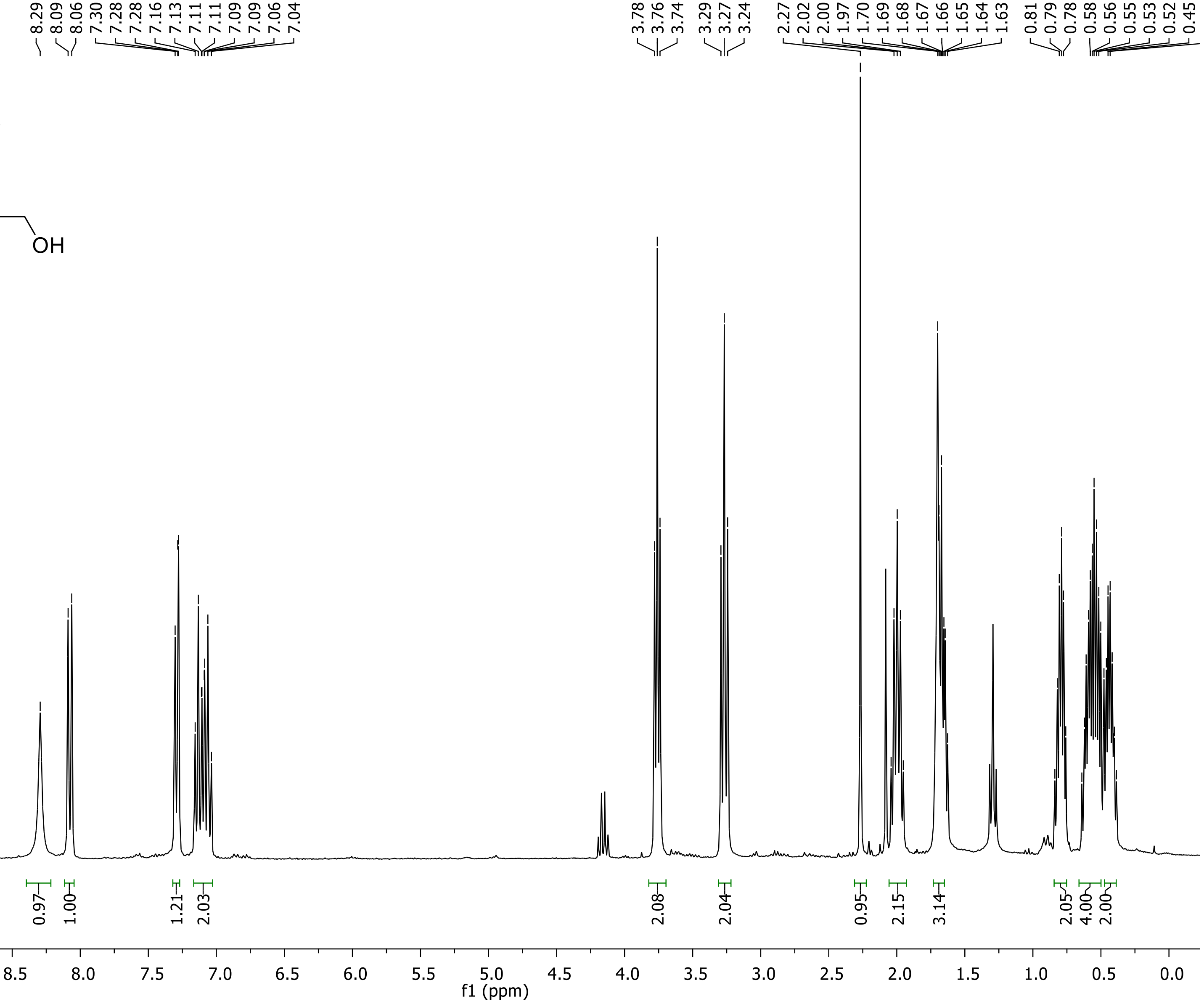

^{13}C NMR ($CDCl_3$, 75.4 MHz)

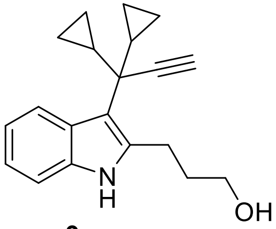

9c

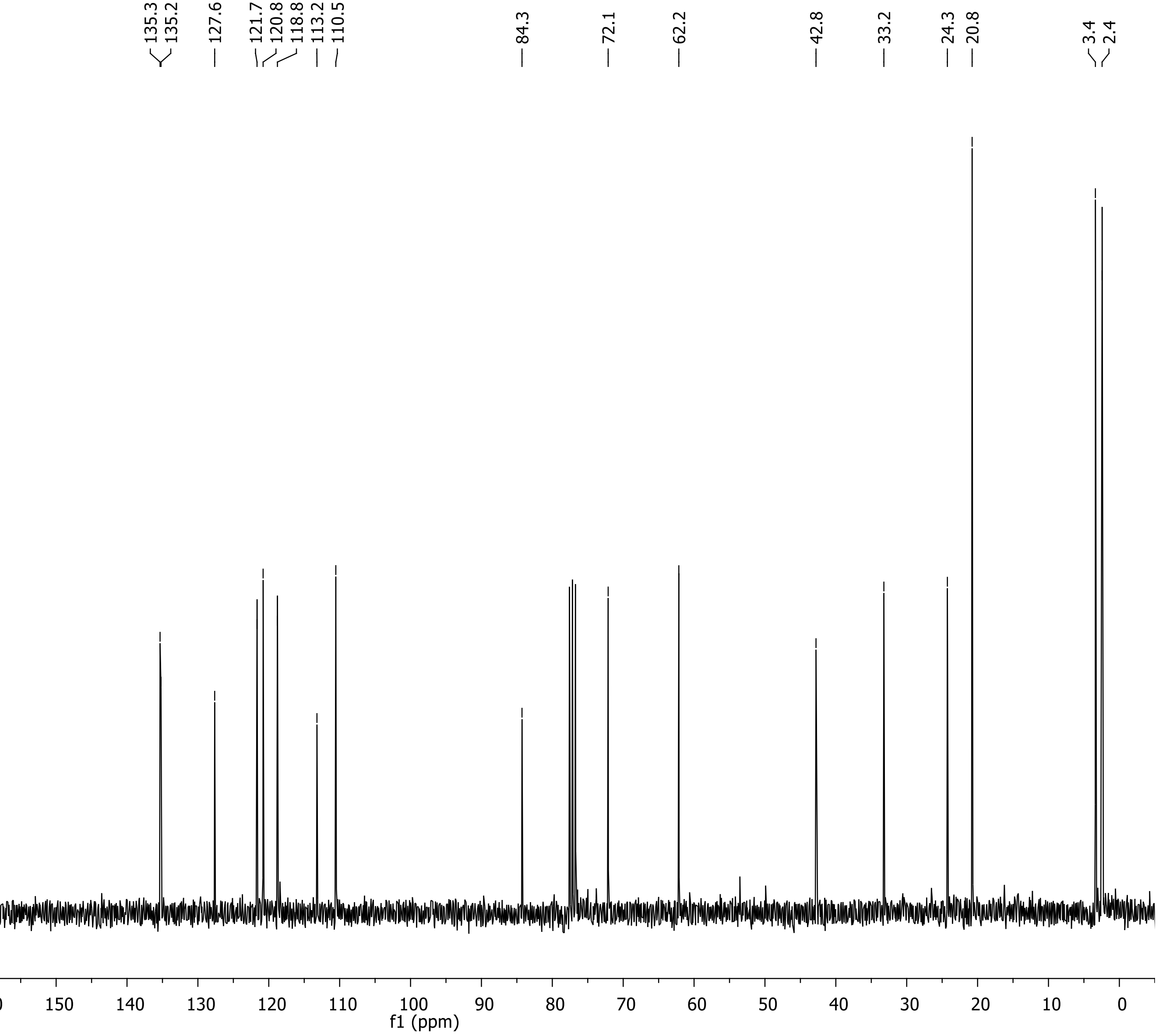

DEPT ($CDCl_3$, 75.4 MHz)

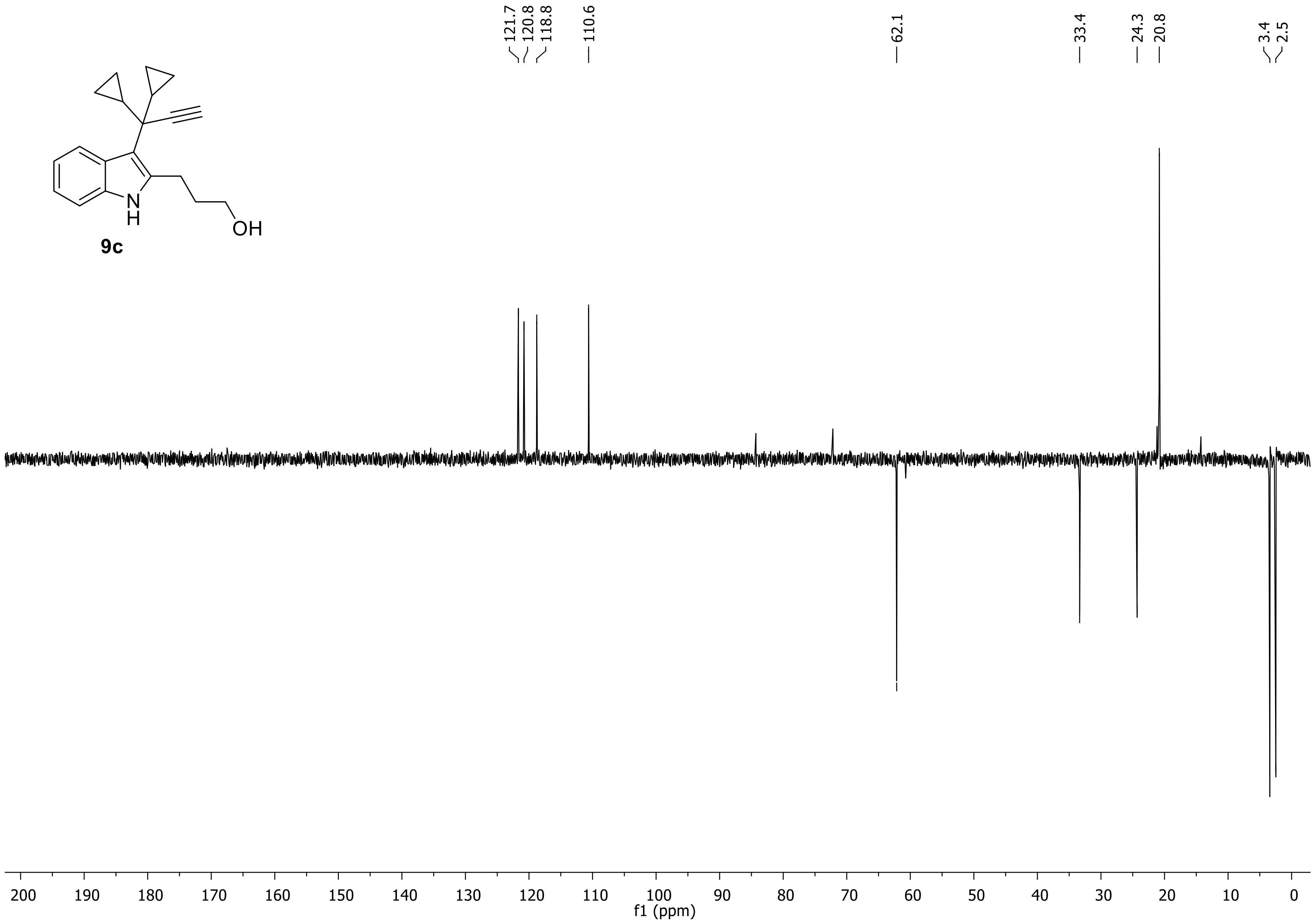

^{1}H NMR ($CDCl_3$, 300 MHz)

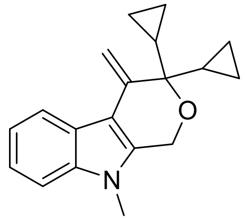

2a

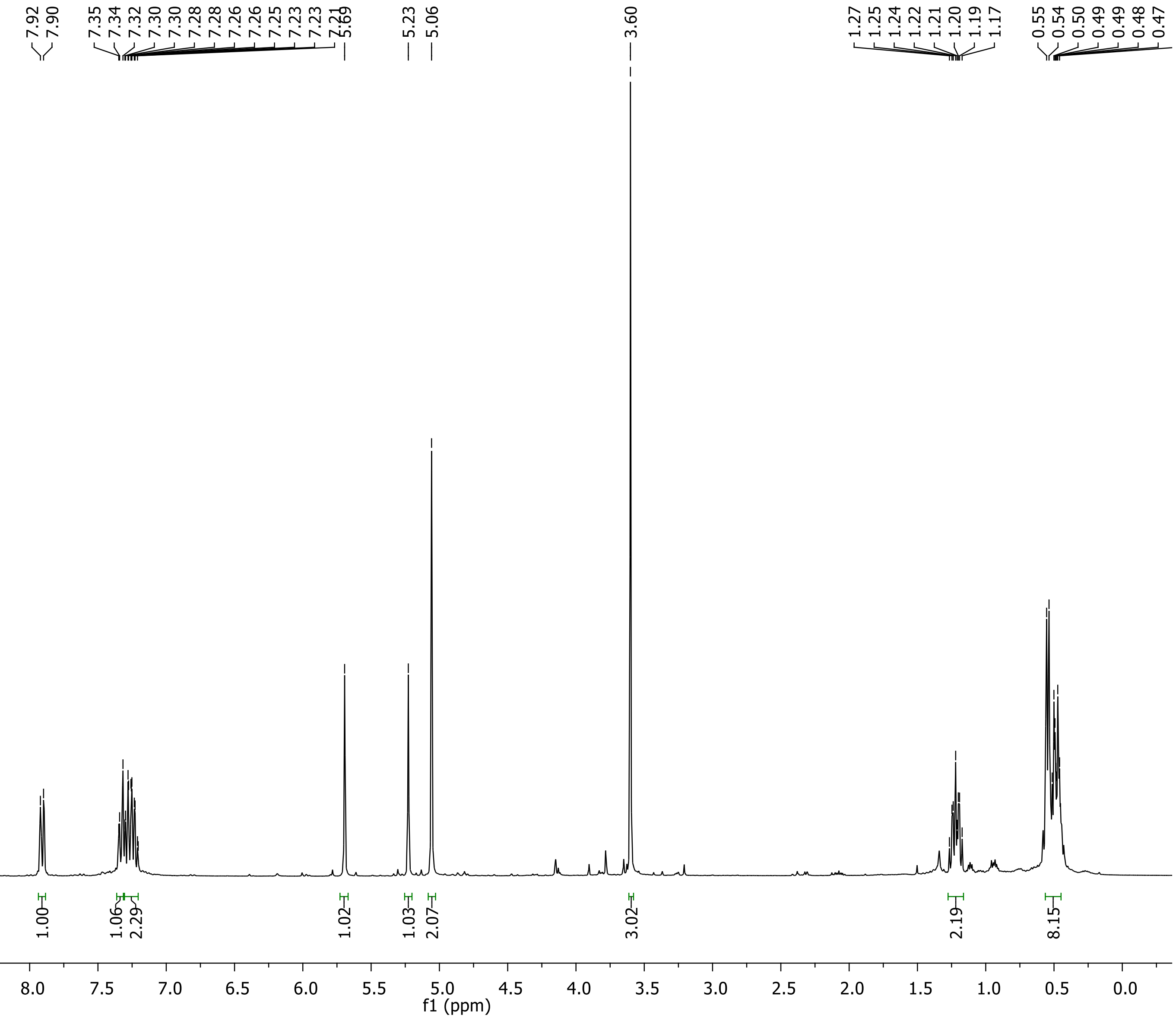

^{13}C NMR ($CDCl_3$, 75.4 MHz)

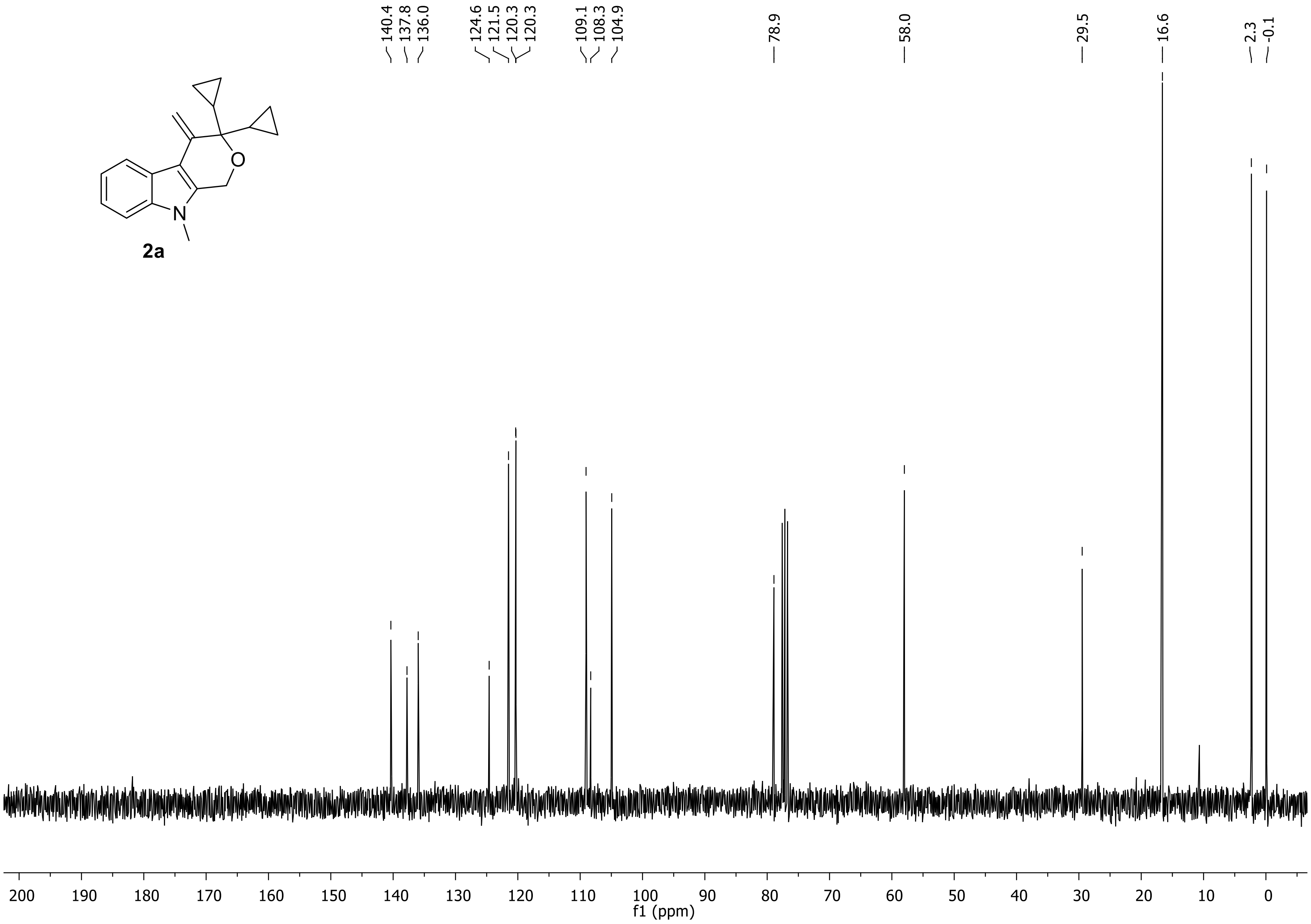

^{1}H NMR ($CDCl_3$, 300 MHz)

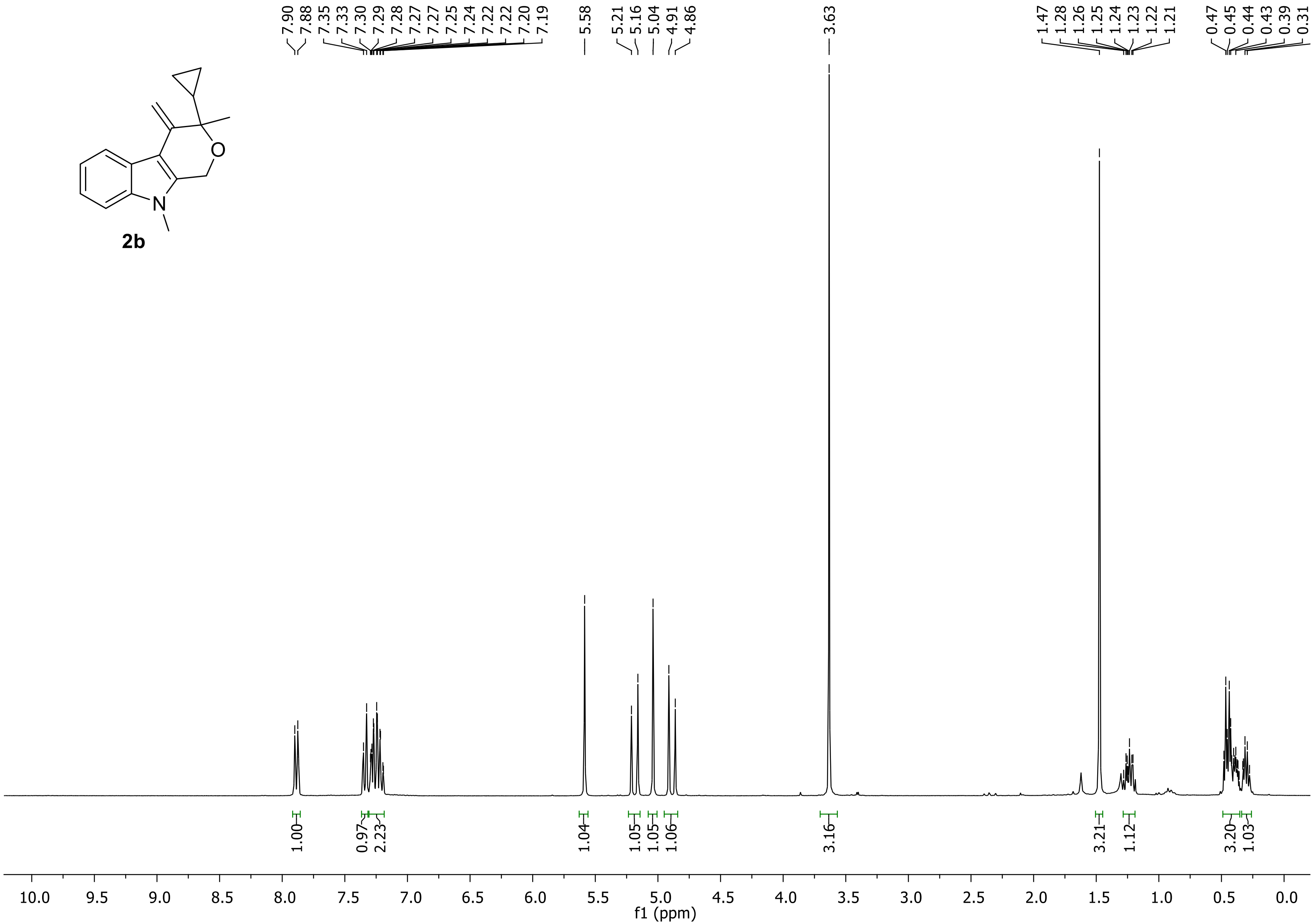

^{13}C NMR ($CDCl_3$, 75.4 MHz)

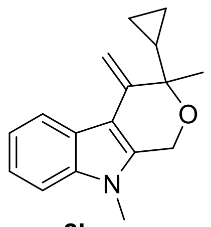

2b

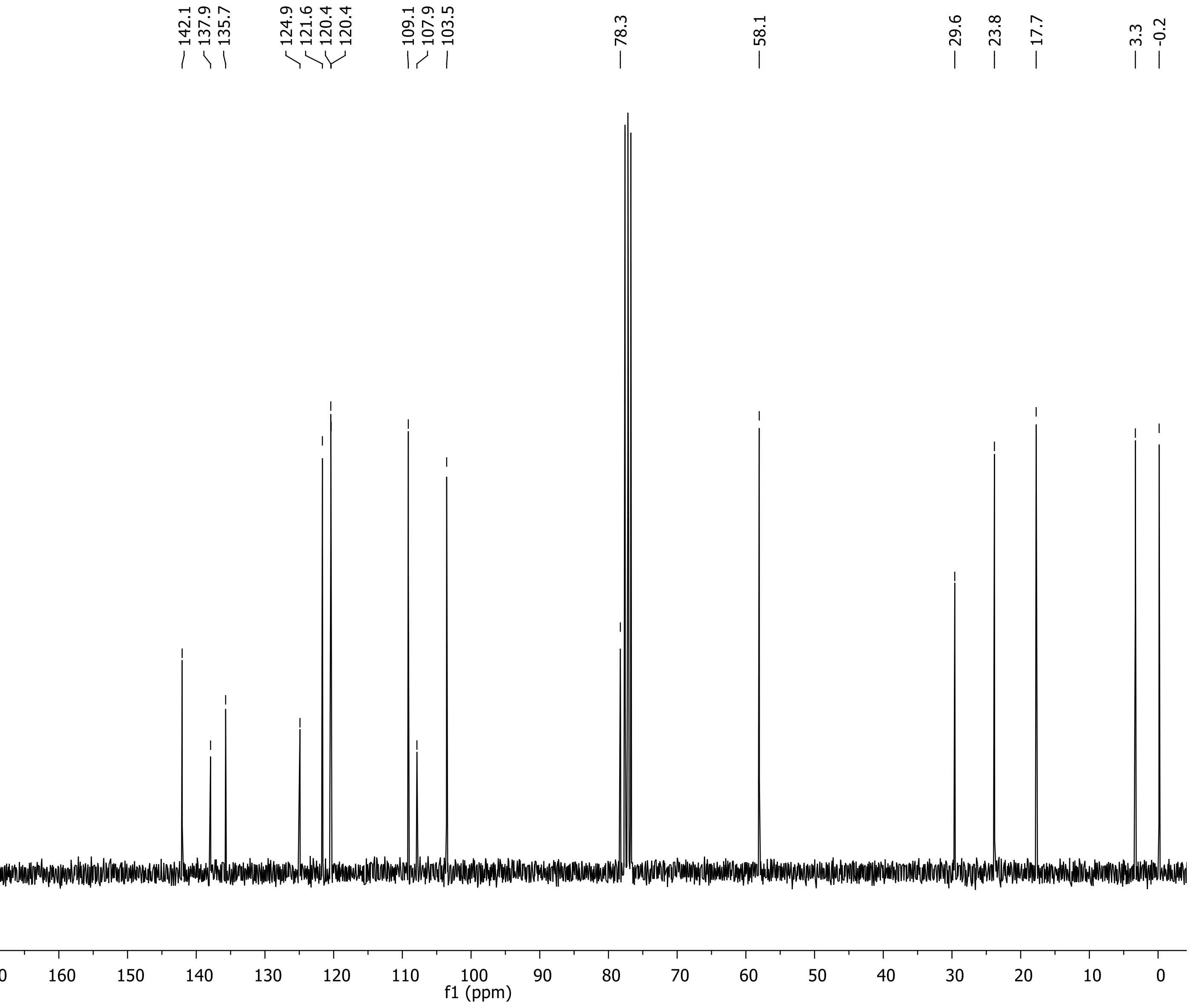

DEPT ($CDCl_3$, 75.4 MHz)

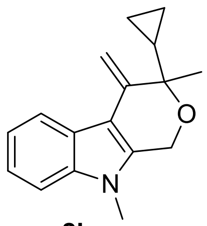

2b

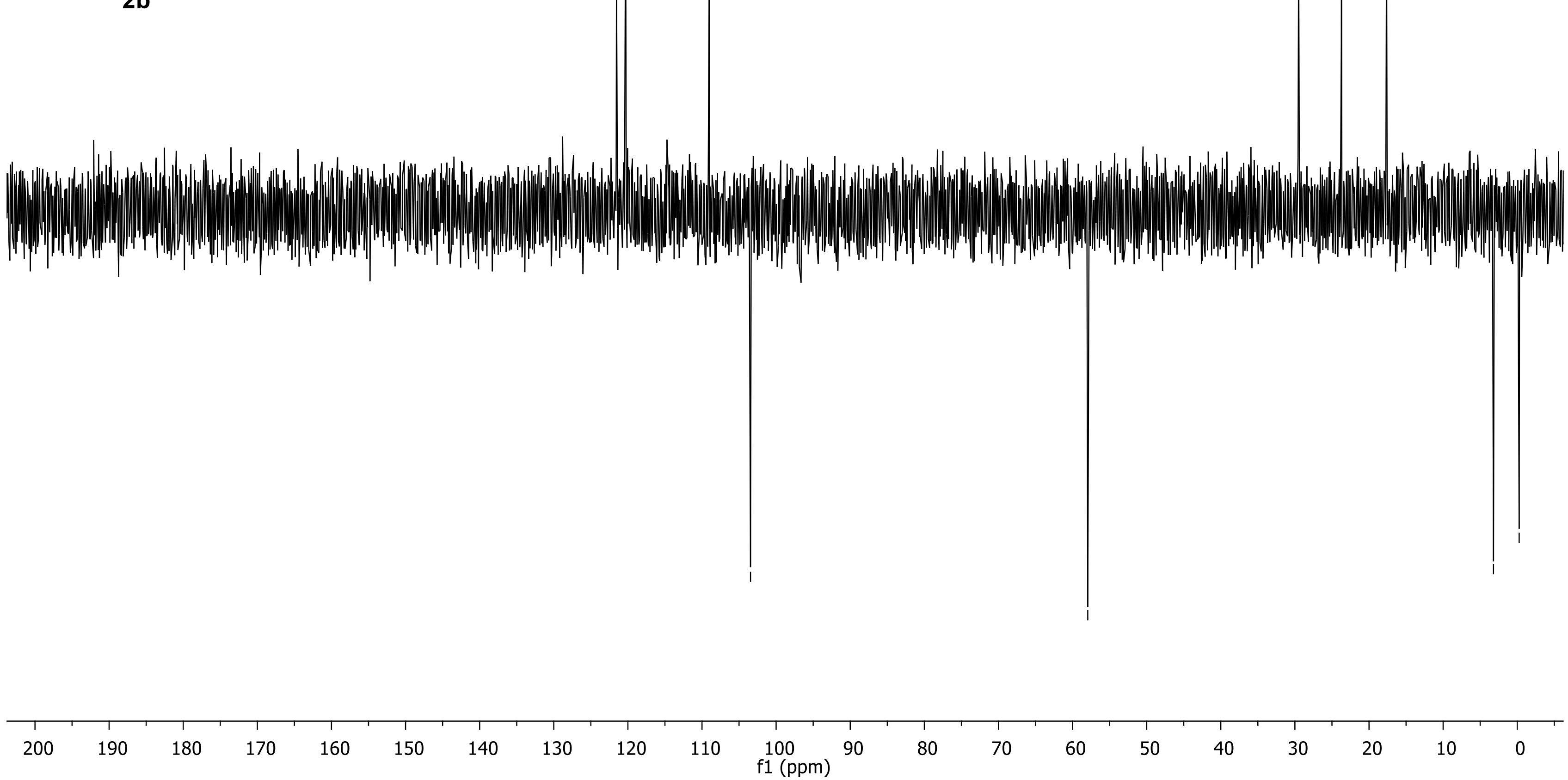

^{1}H NMR ($CDCl_3$, 300 MHz)

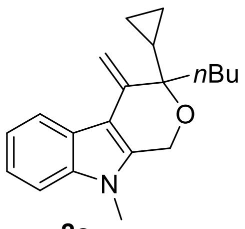

2c

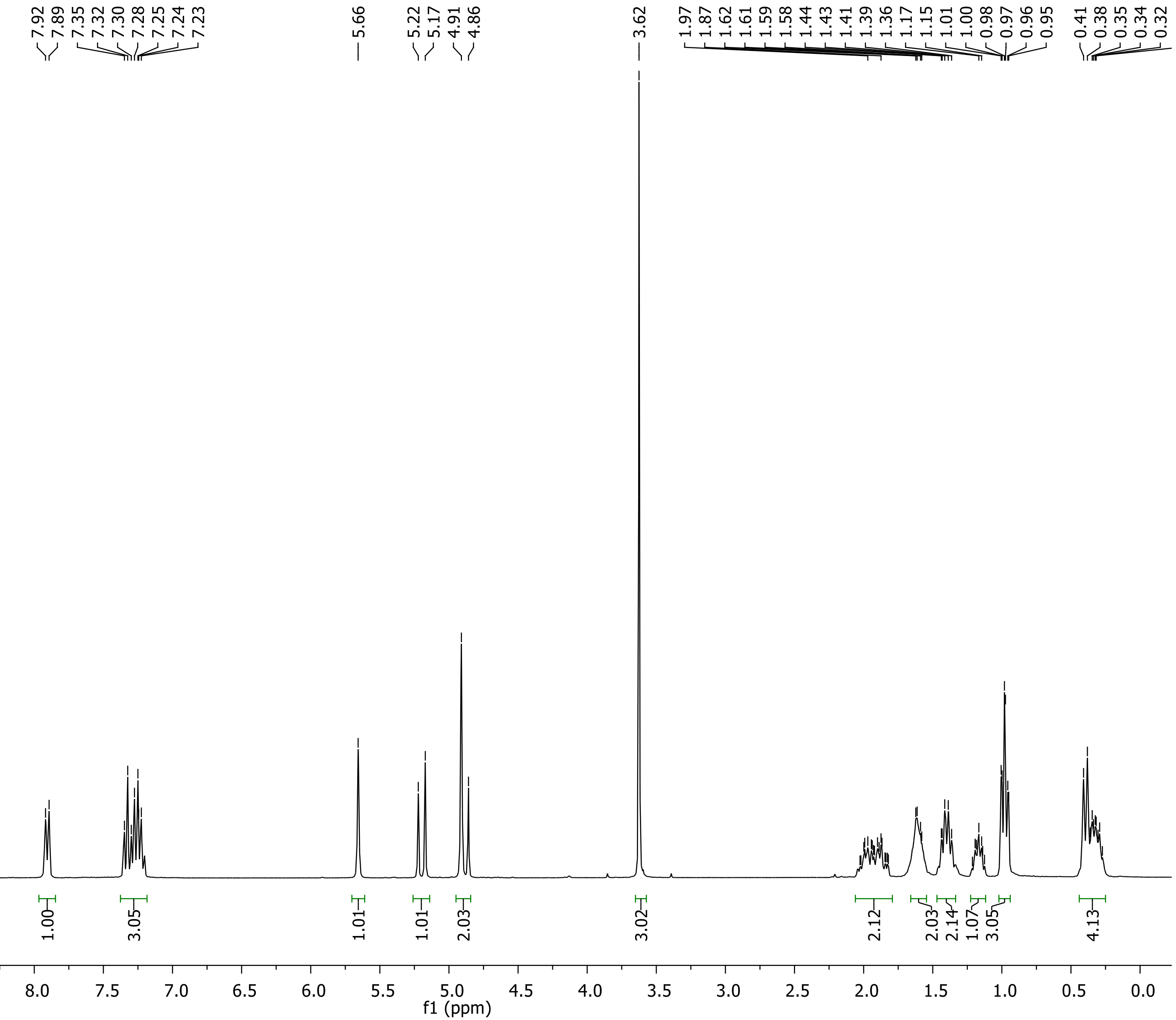

^{13}C NMR ($CDCl_3$, 75.4 MHz)

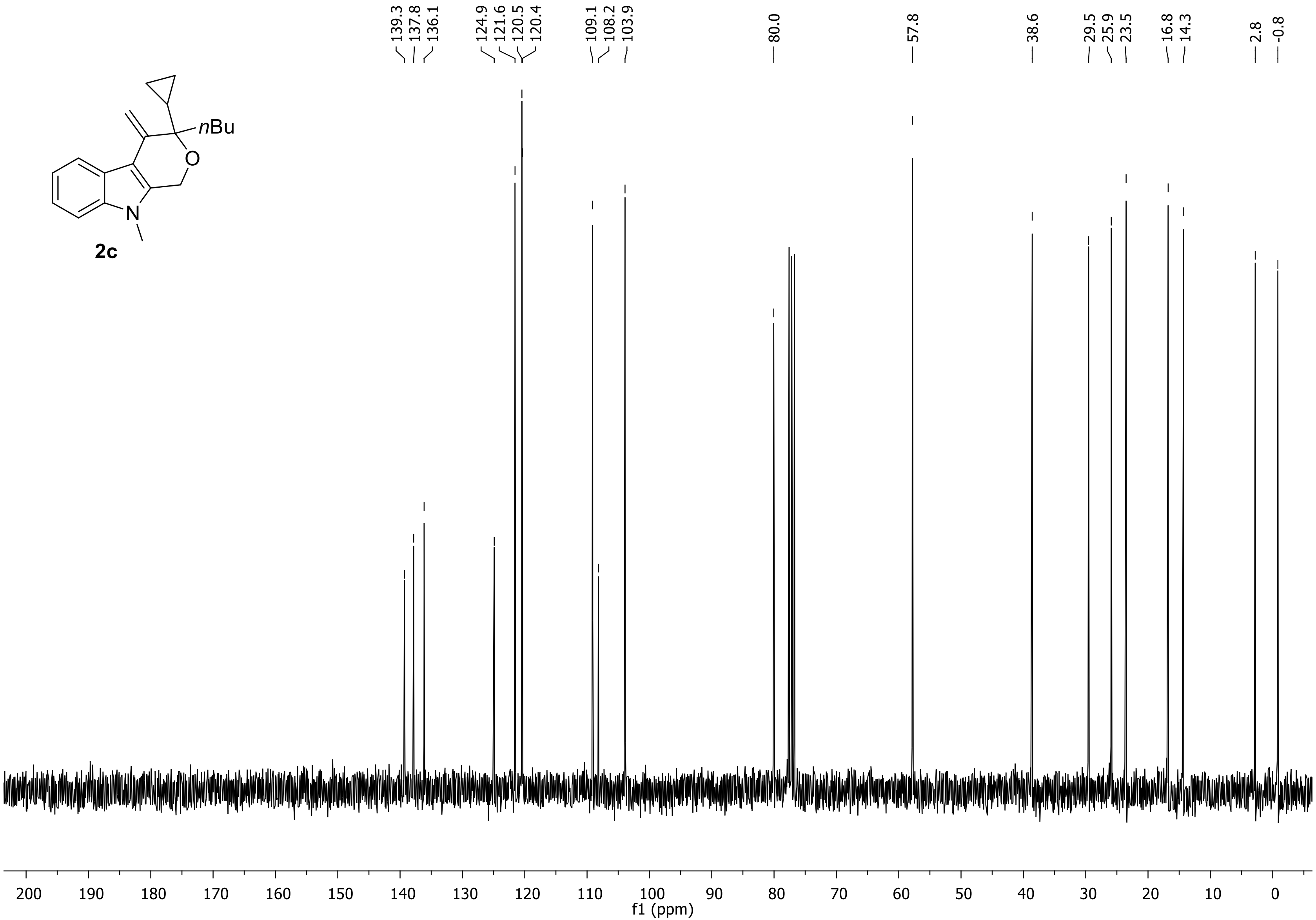

DEPT ($CDCl_3$, 75.4 MHz)

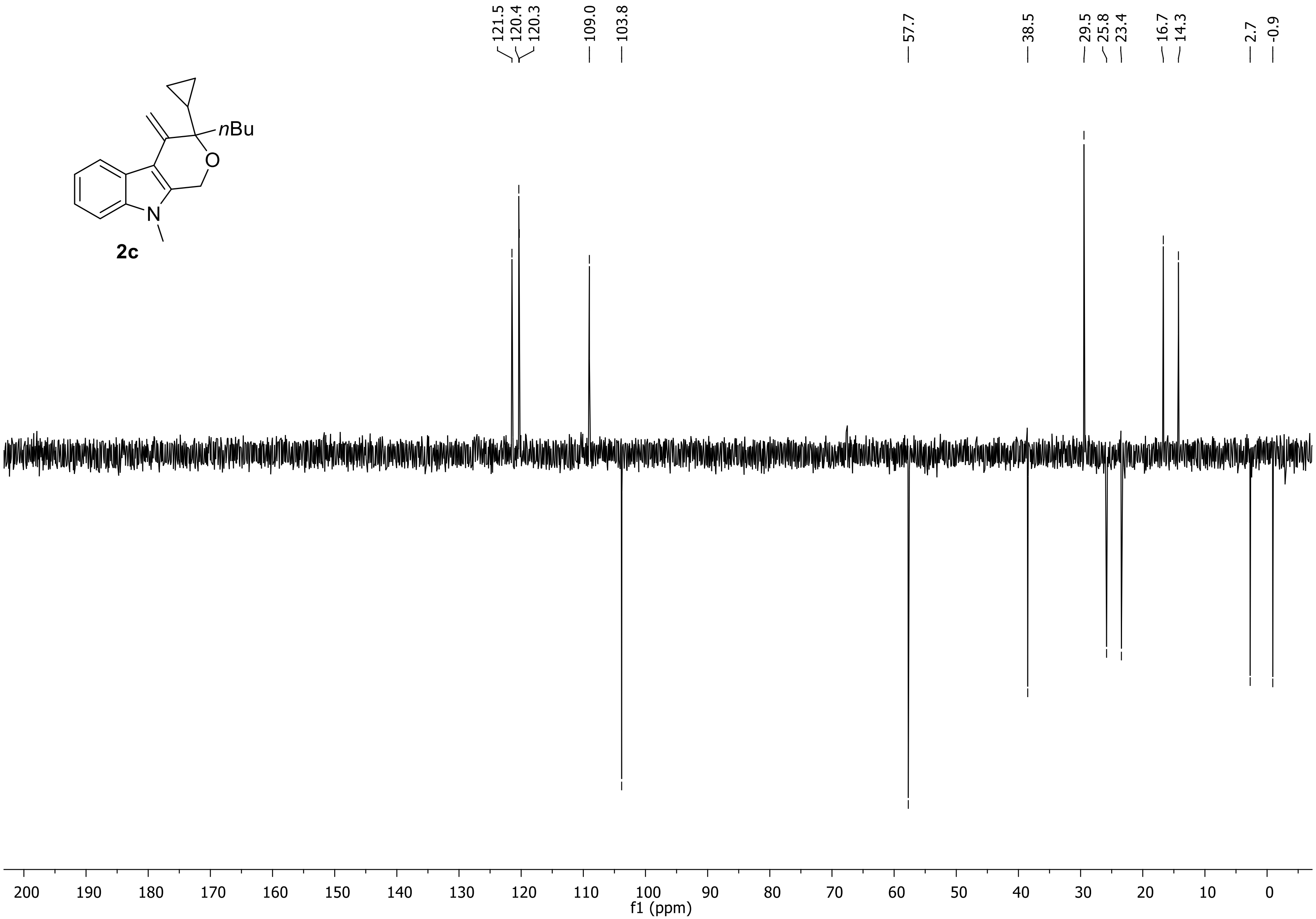

^{1}H NMR ($CDCl_3$, 500 MHz)

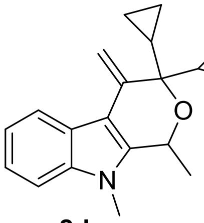

2d

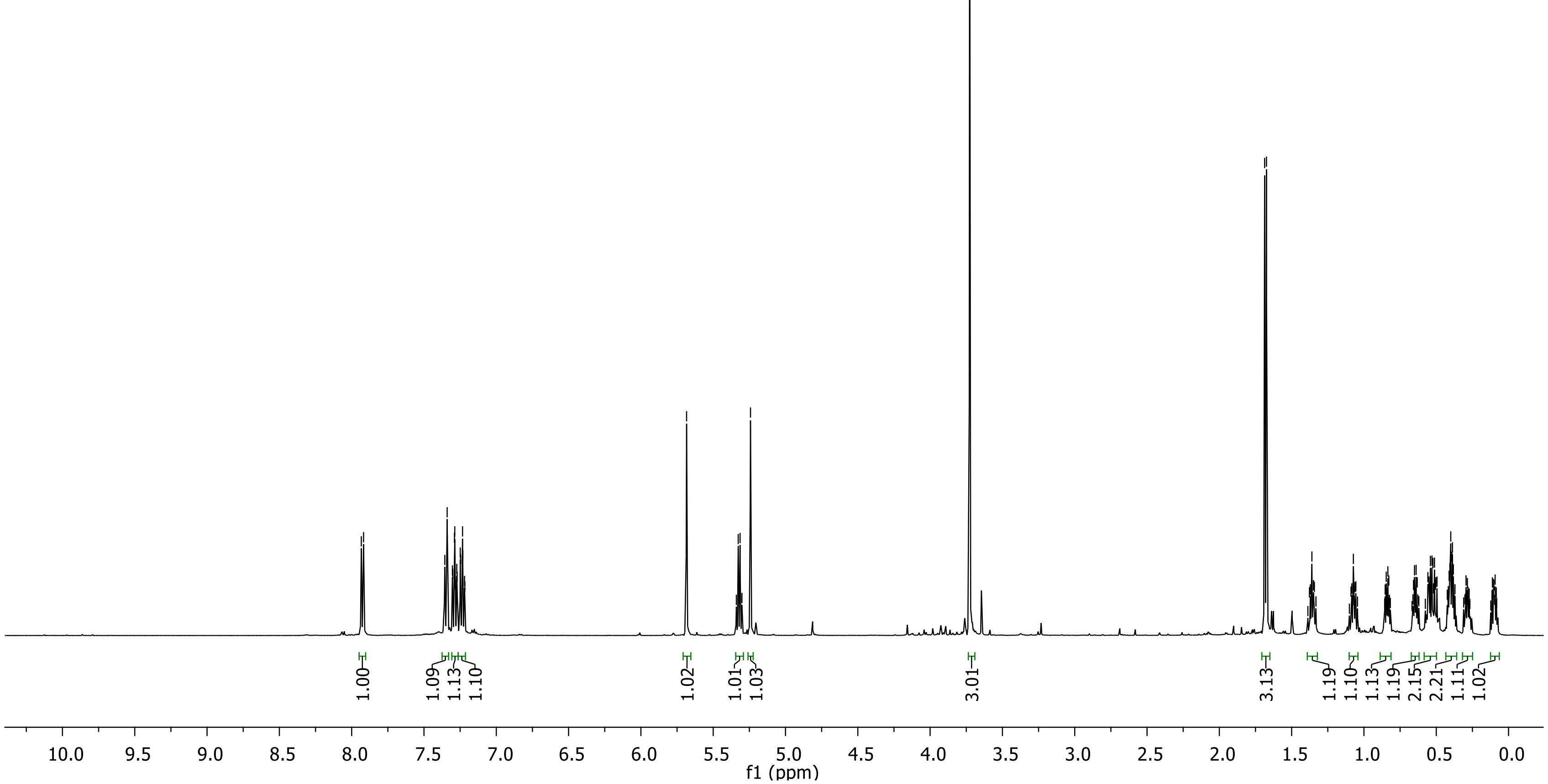

^{13}C NMR ($CDCl_3$, 125.7 MHz)

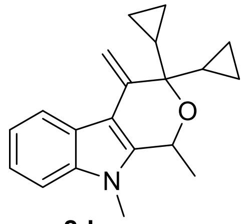

2d

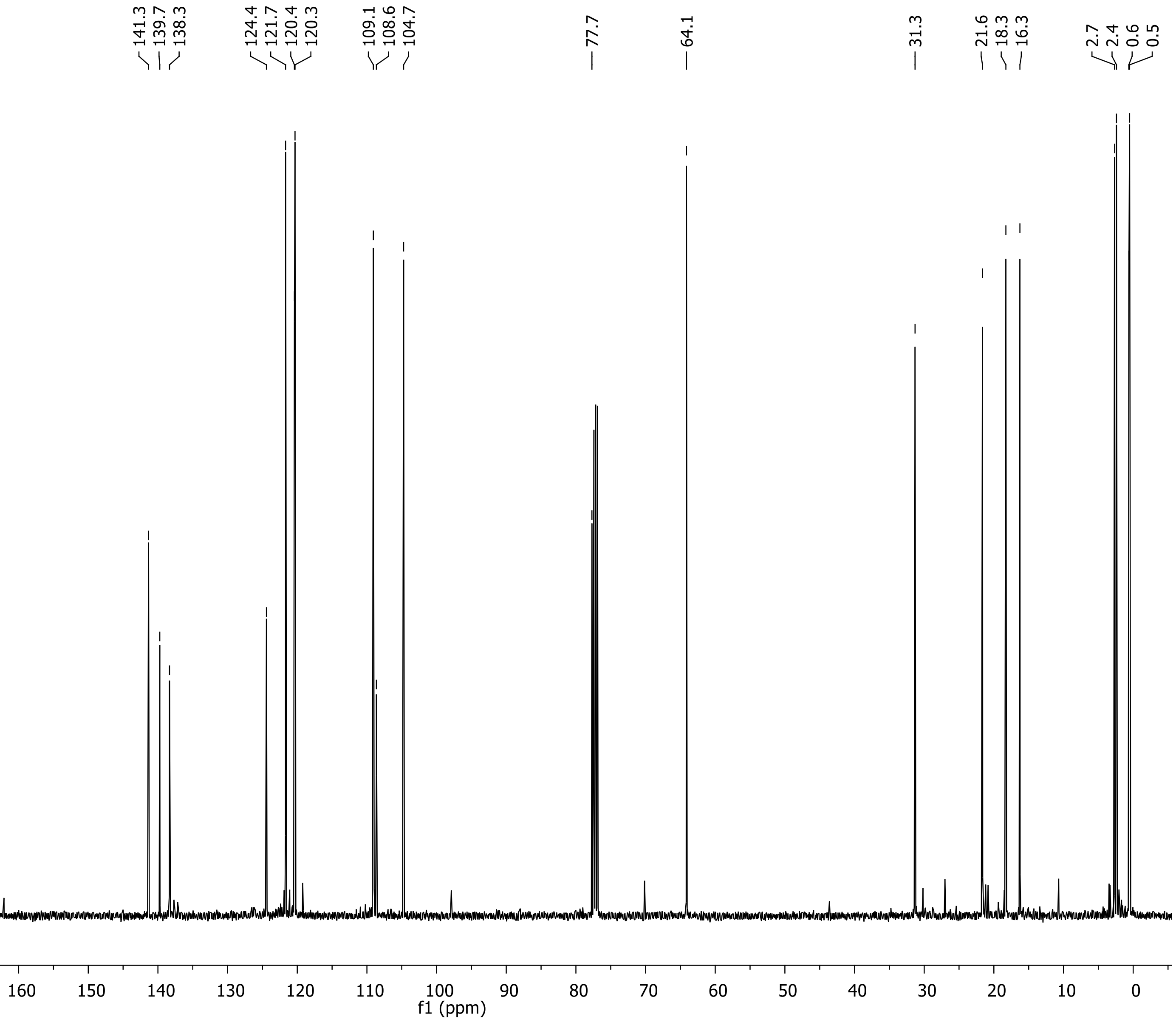

DEPT ($CDCl_3$, 125.7 MHz)

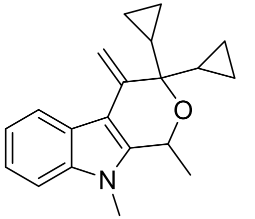

2d

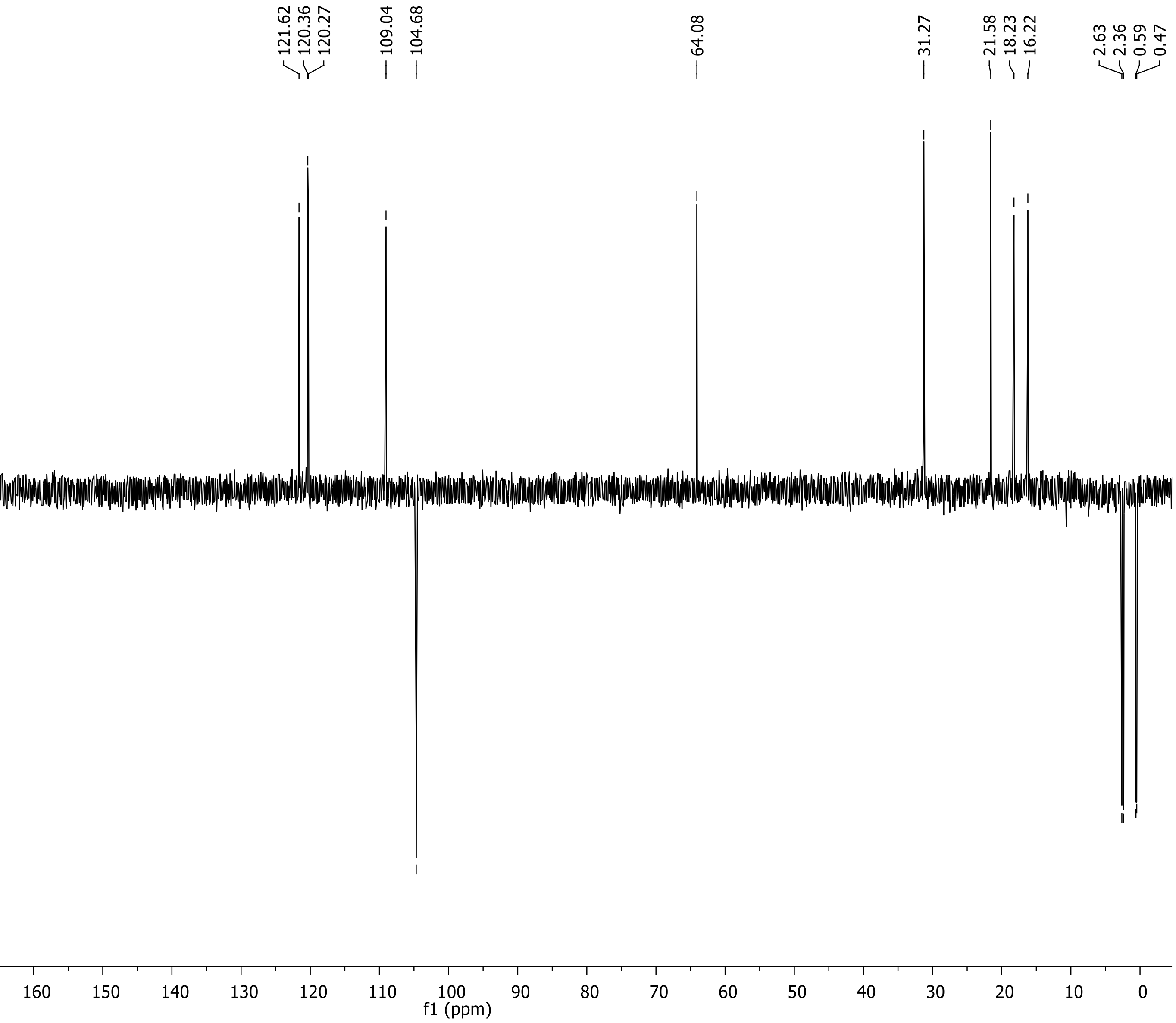

^{1}H NMR ($CDCl_3$, 300 MHz)

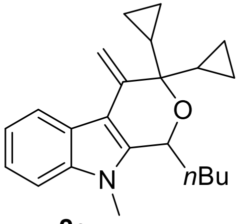

2e

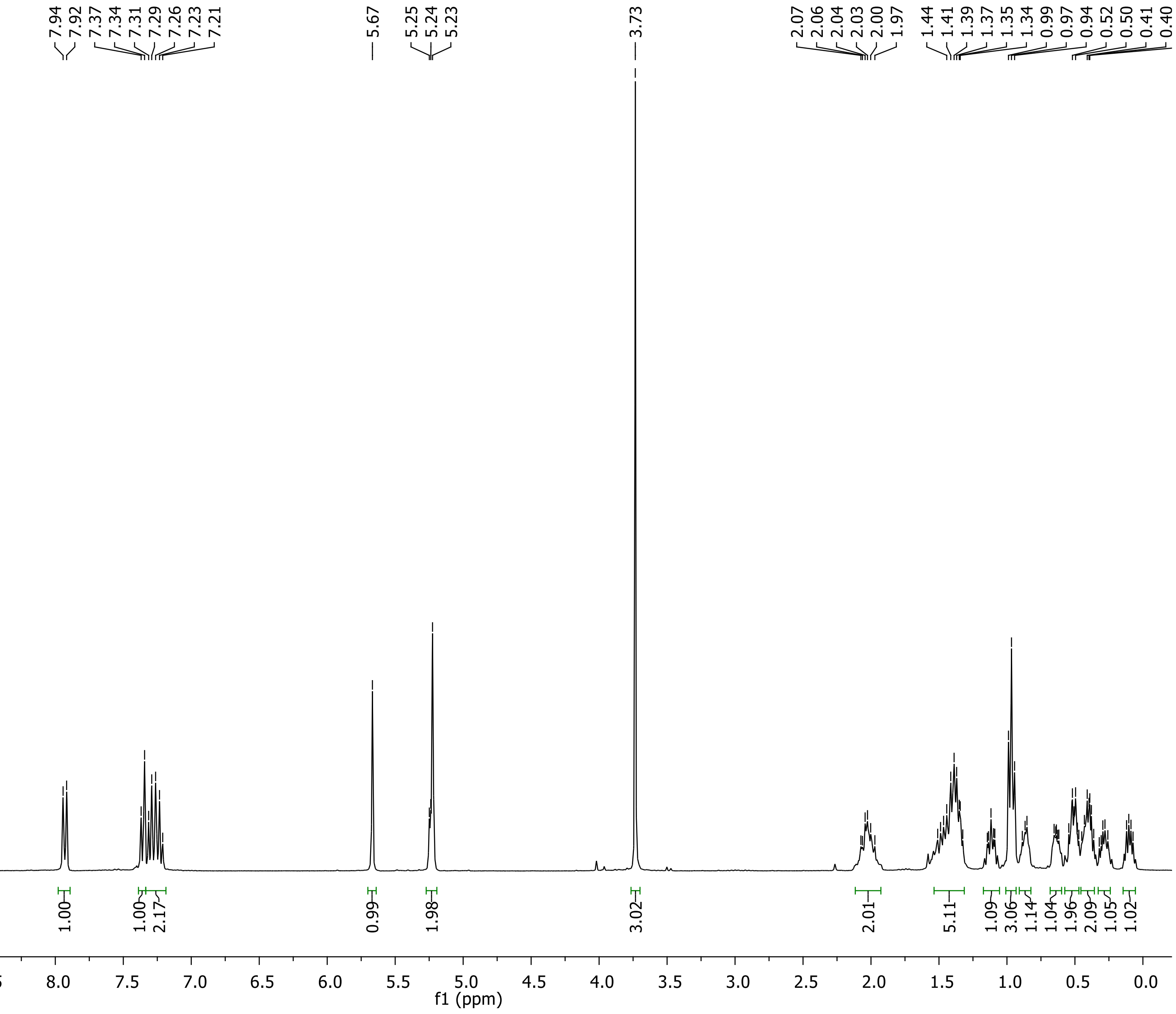

^{13}C NMR ($CDCl_3$, 75.4 MHz)

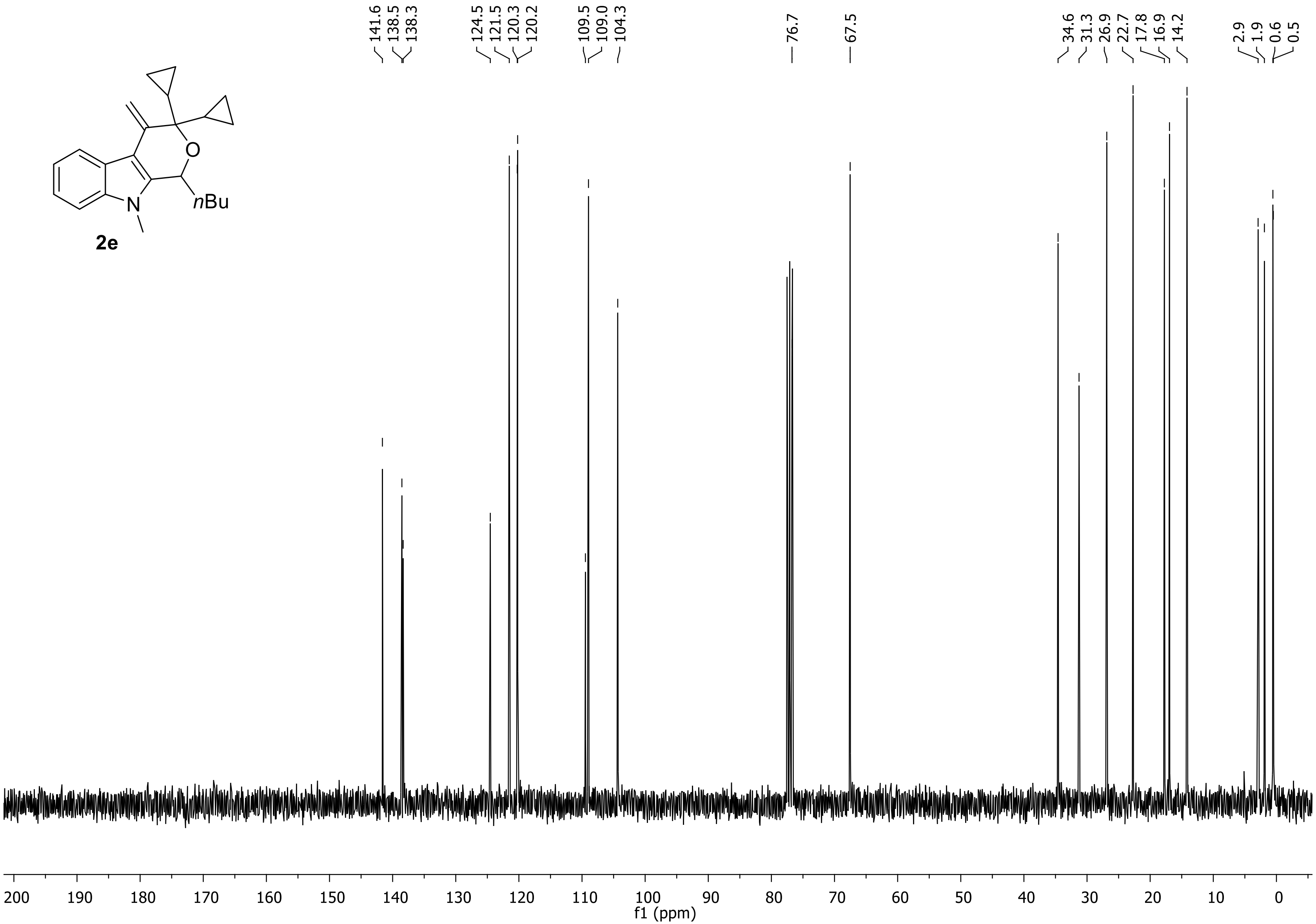

DEPT ($CDCl_3$, 75.4 MHz)

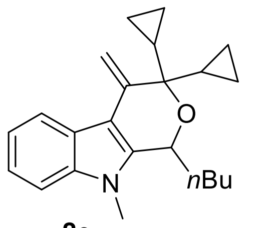

2e

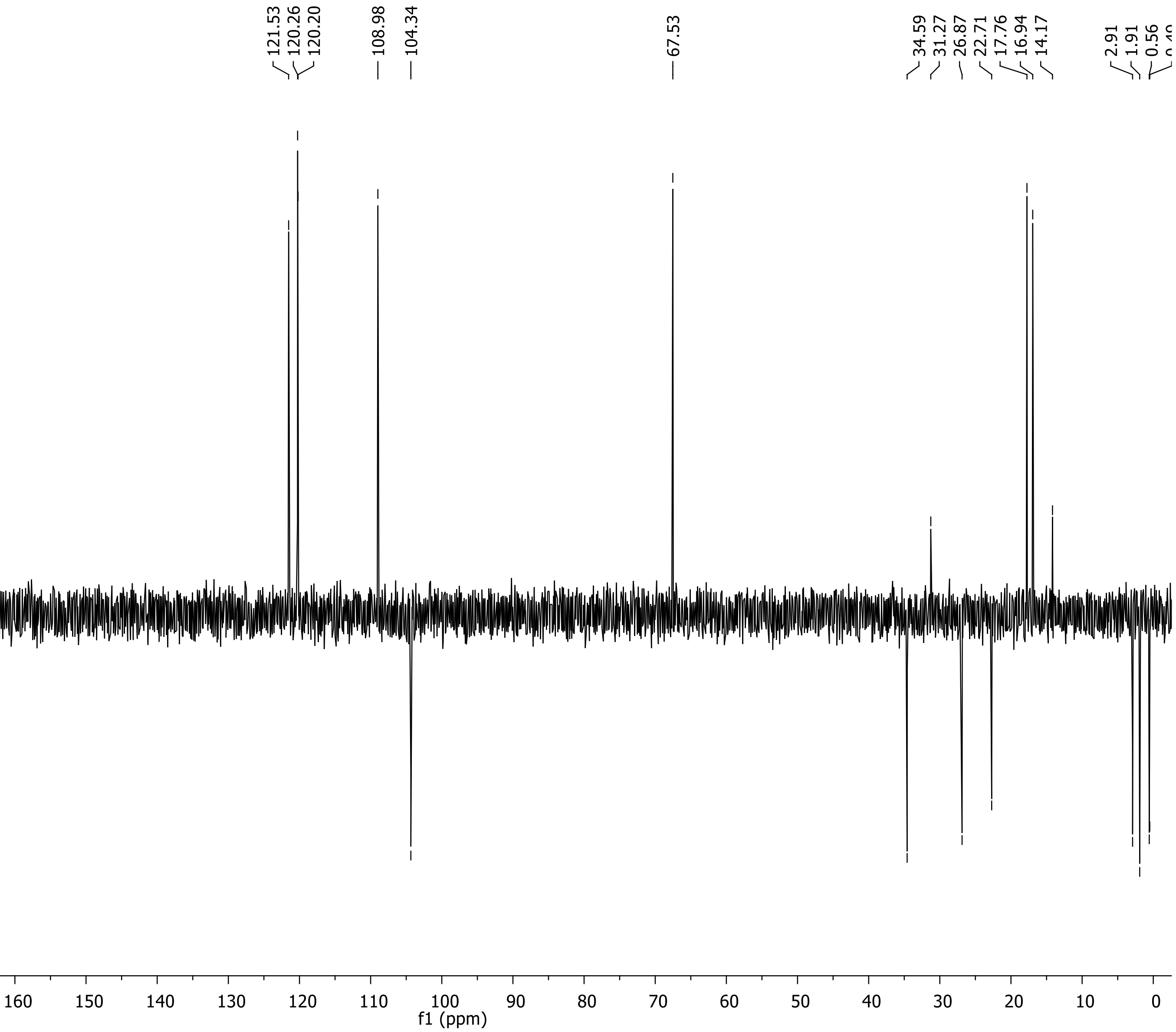

^1H NMR ($CDCl_3$, 300 MHz)

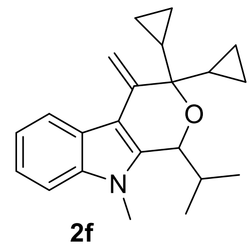

2f

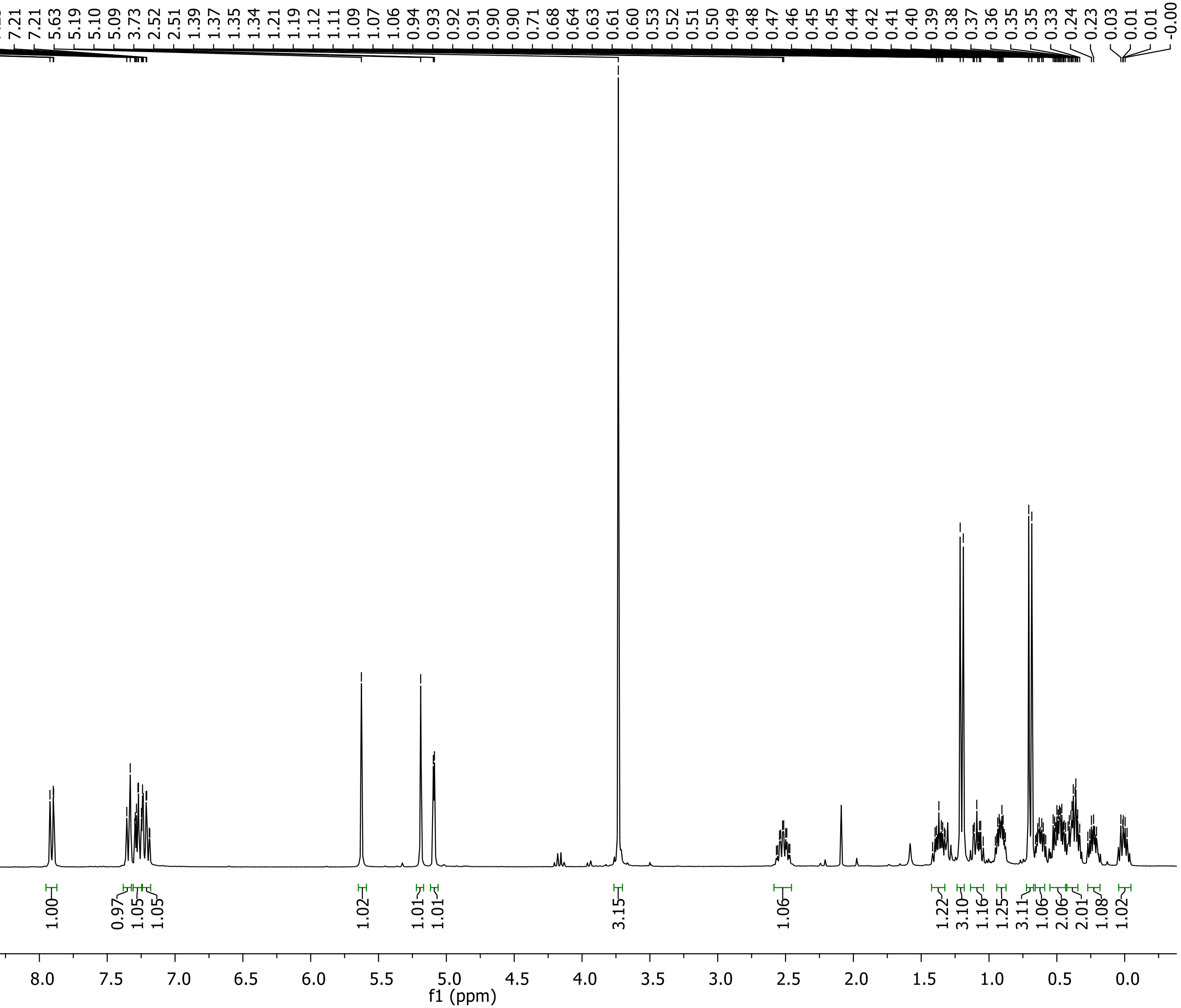

^{13}C NMR ($CDCl_3$, 75.4 MHz)

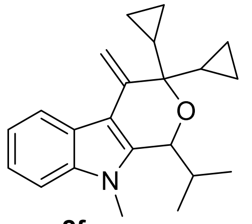

2f

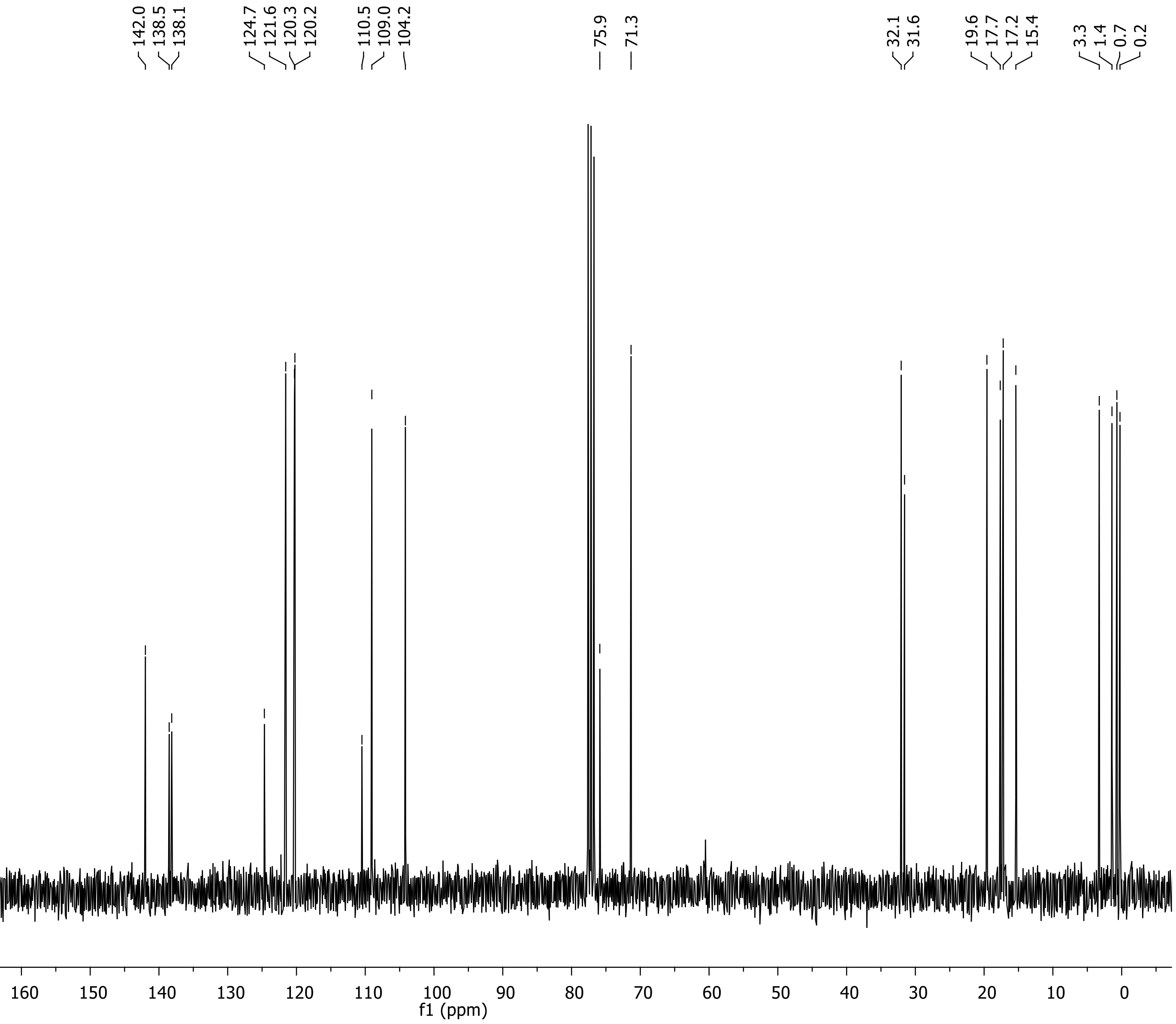

DEPT ($CDCl_3$, 75.4 MHz)

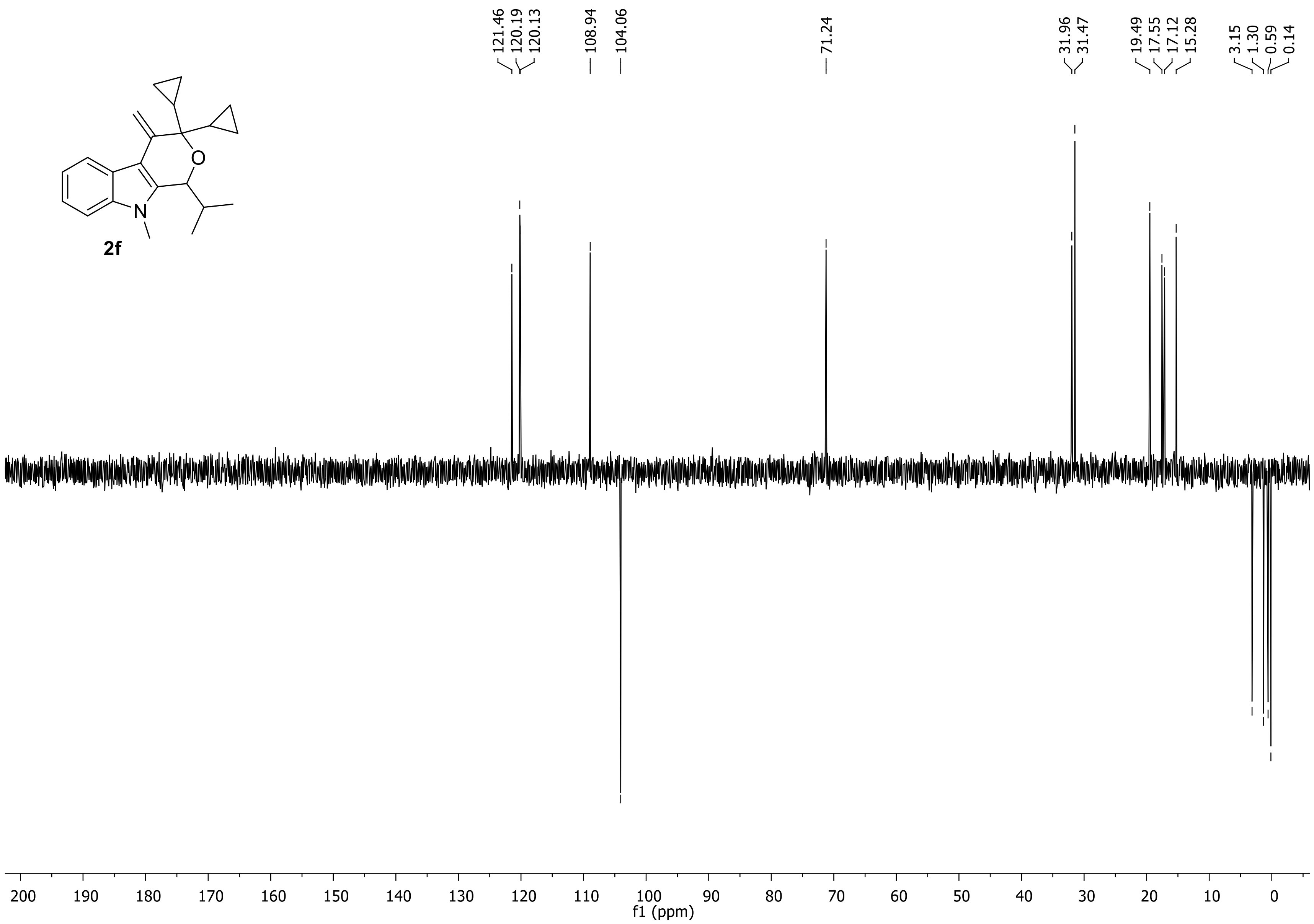

1H NMR (CDCl$_3$, 500 MHz)

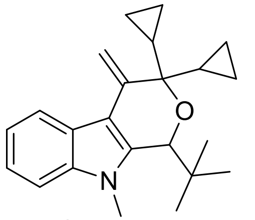

2g

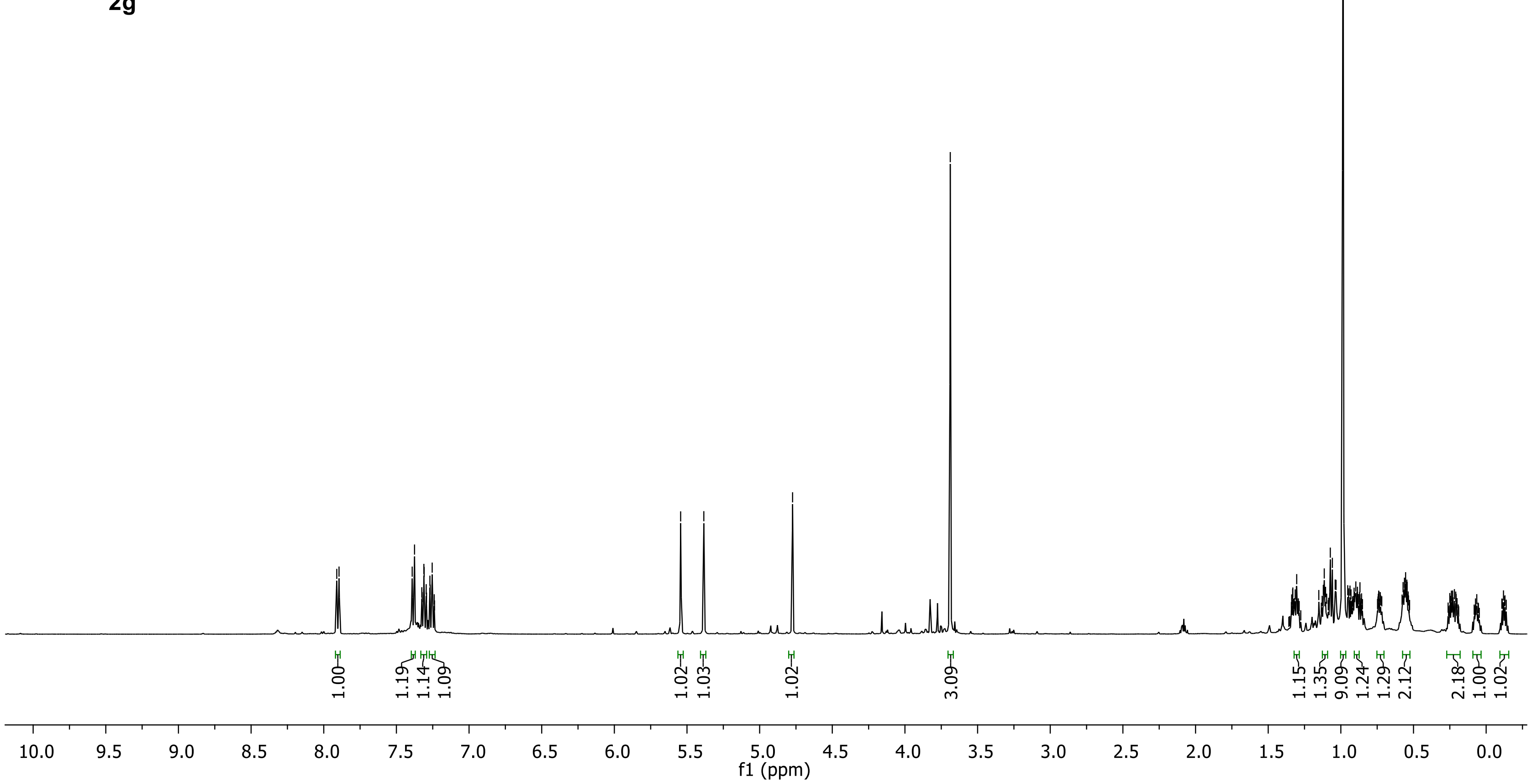

^{13}C NMR ($CDCl_3$, 125.7 MHz)

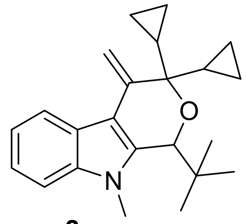

2g

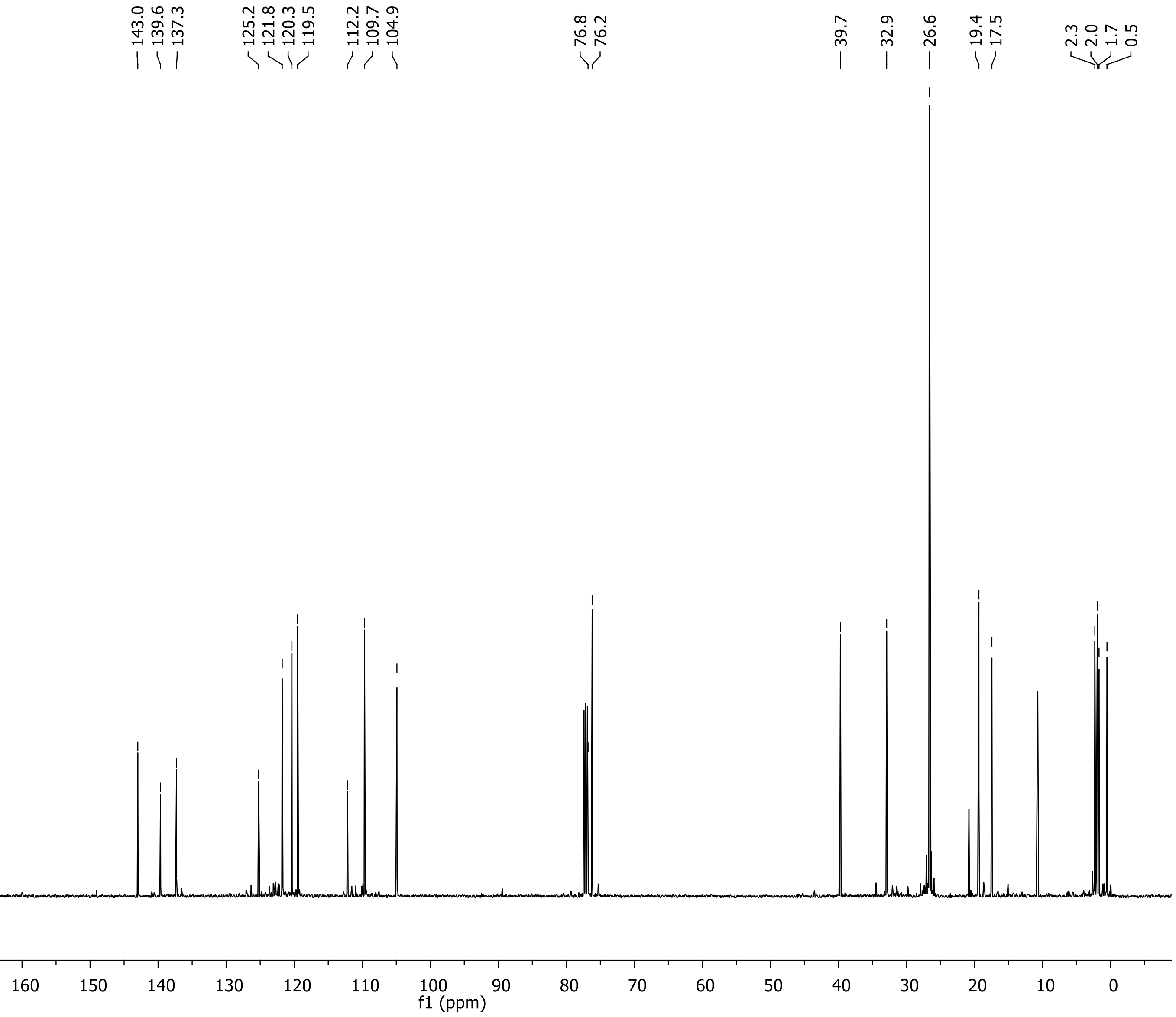

DEPT ($CDCl_3$, 125.7 MHz)

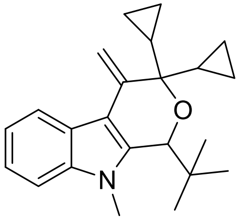

2g

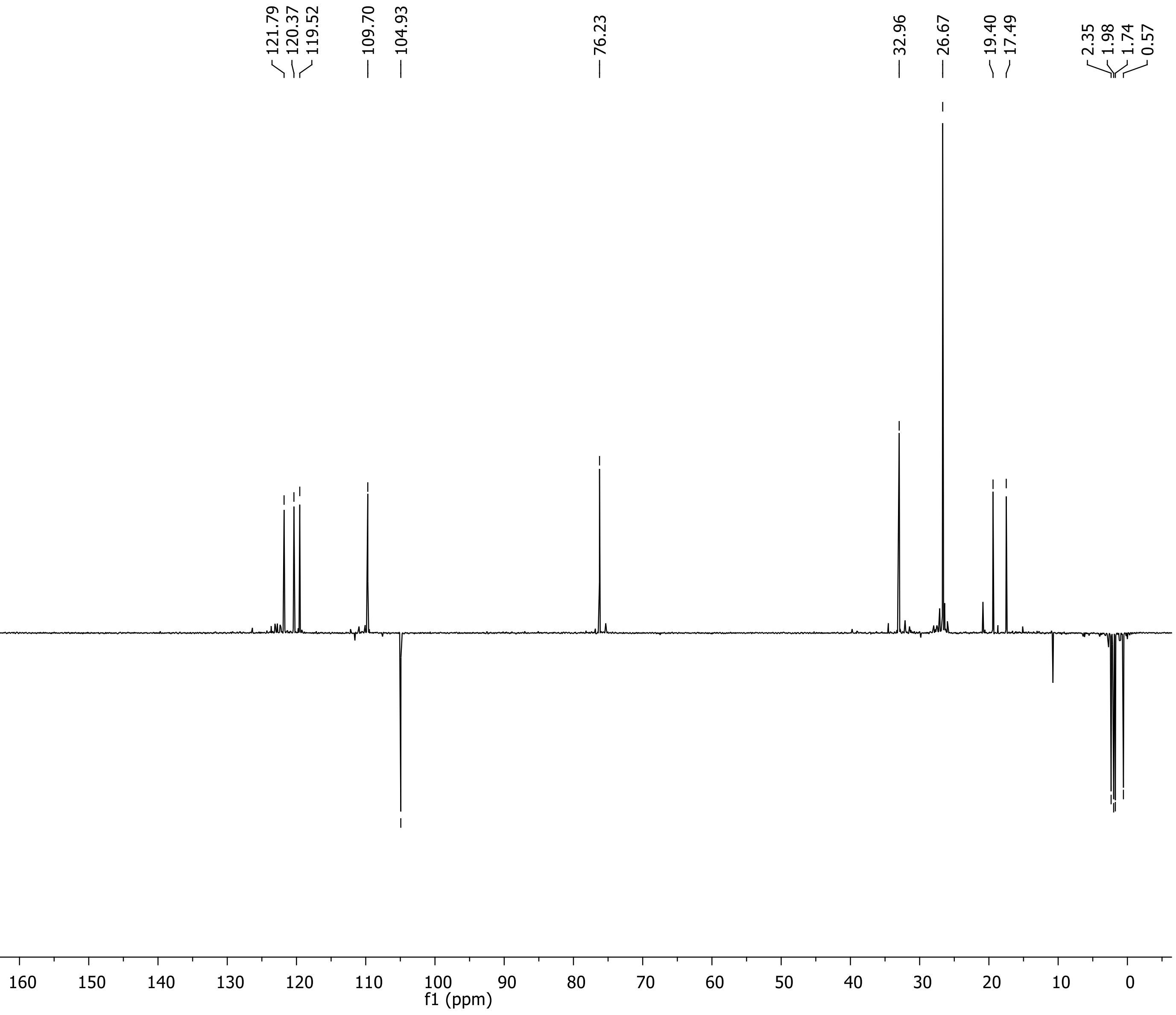

^{1}H NMR ((CD$_3$)$_2$O, 300 MHz)

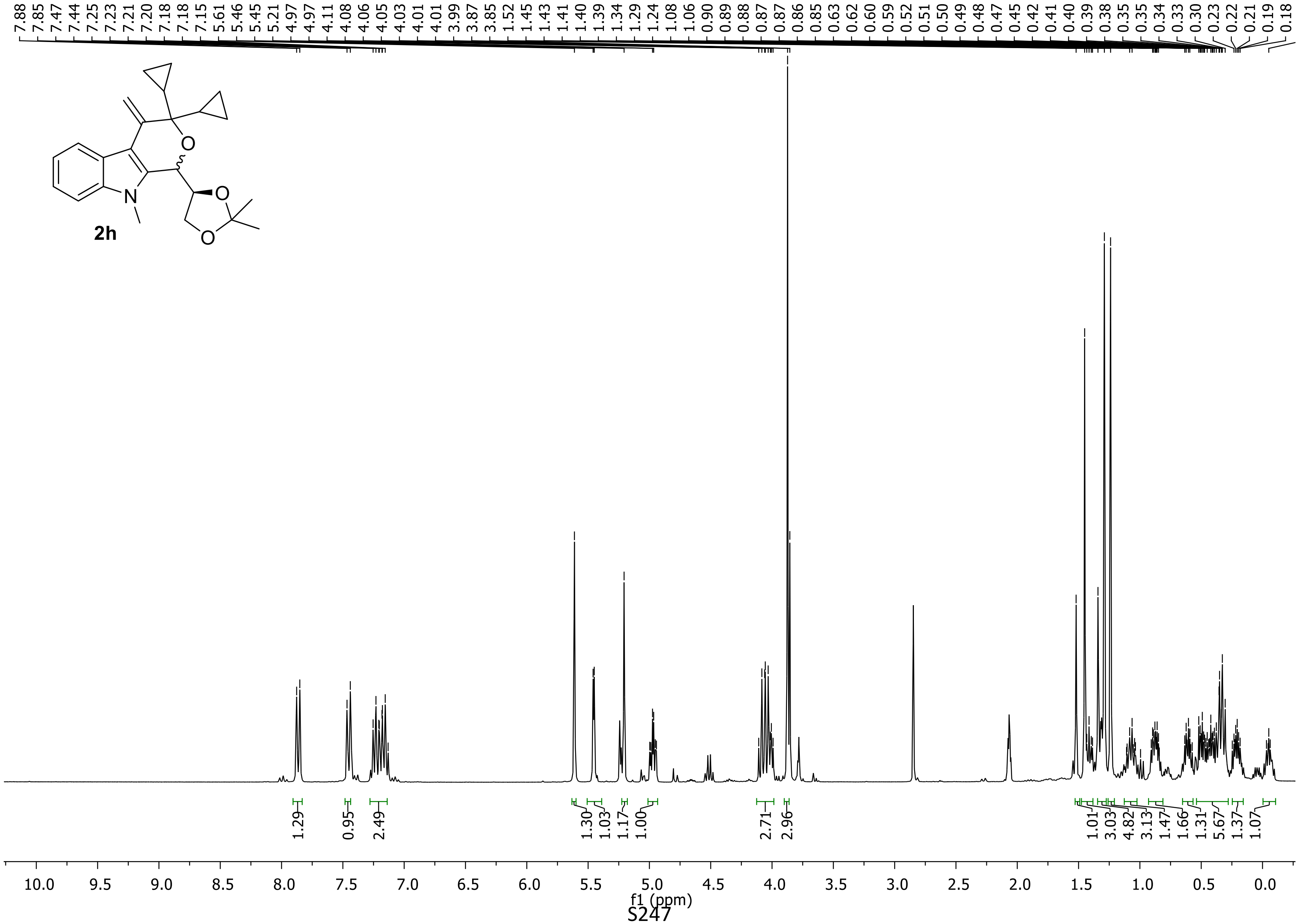

^{13}C NMR ($(CD_3)_2O$, 75.4 MHz)

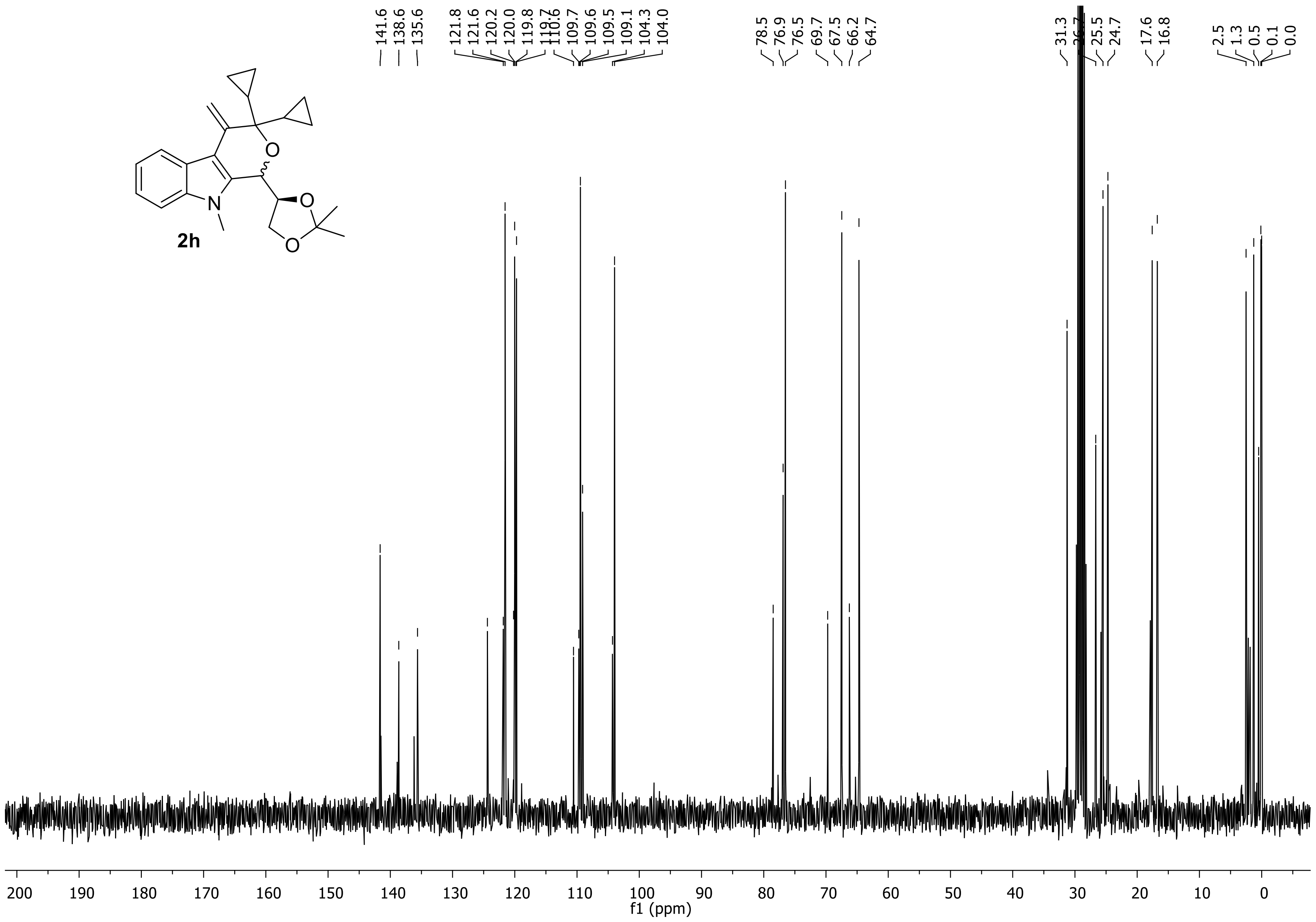

DEPT ($(CD_3)_2O$, 75.4 MHz)

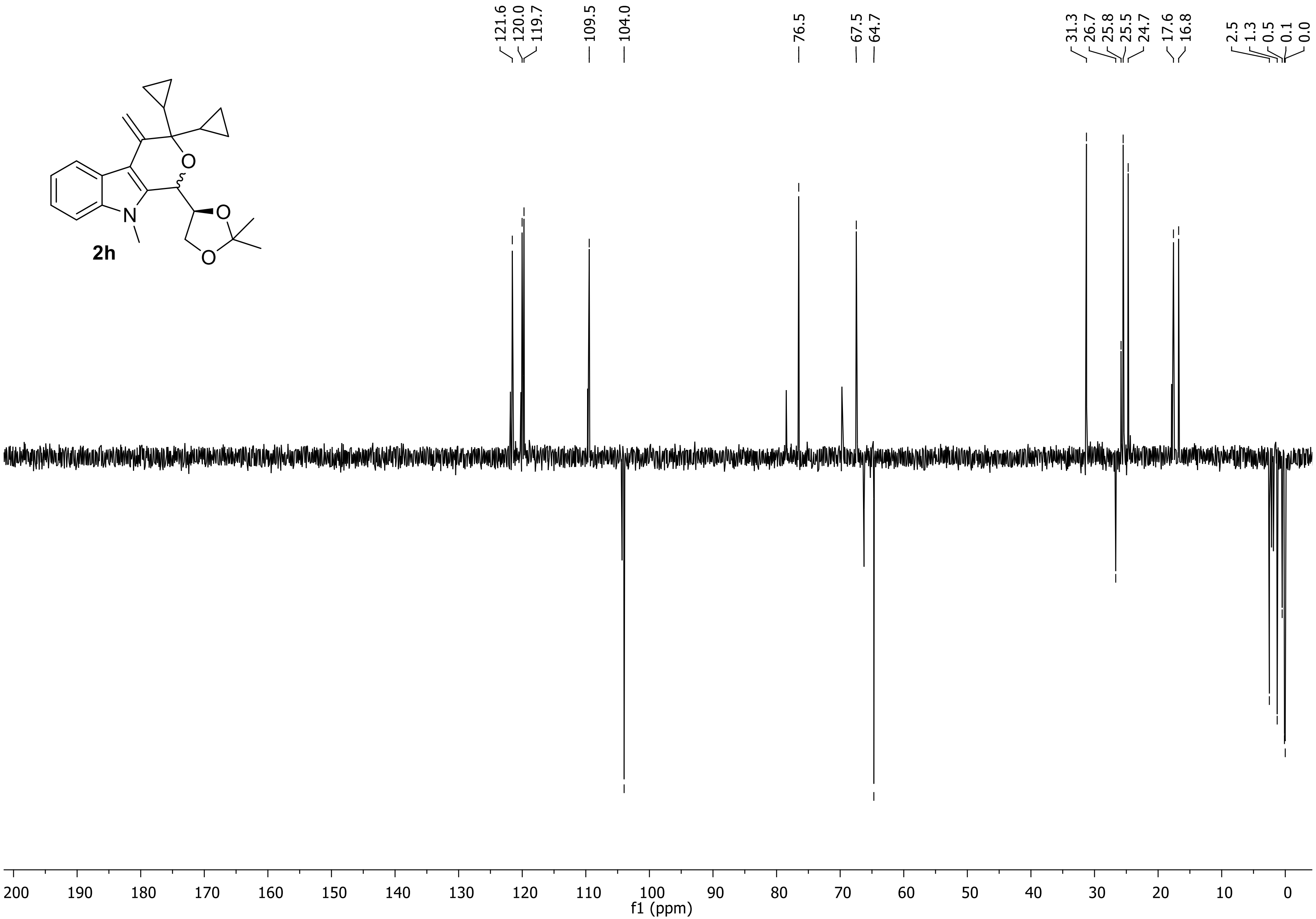

2h

^{1}H NMR ($CDCl_3$, 300 MHz)

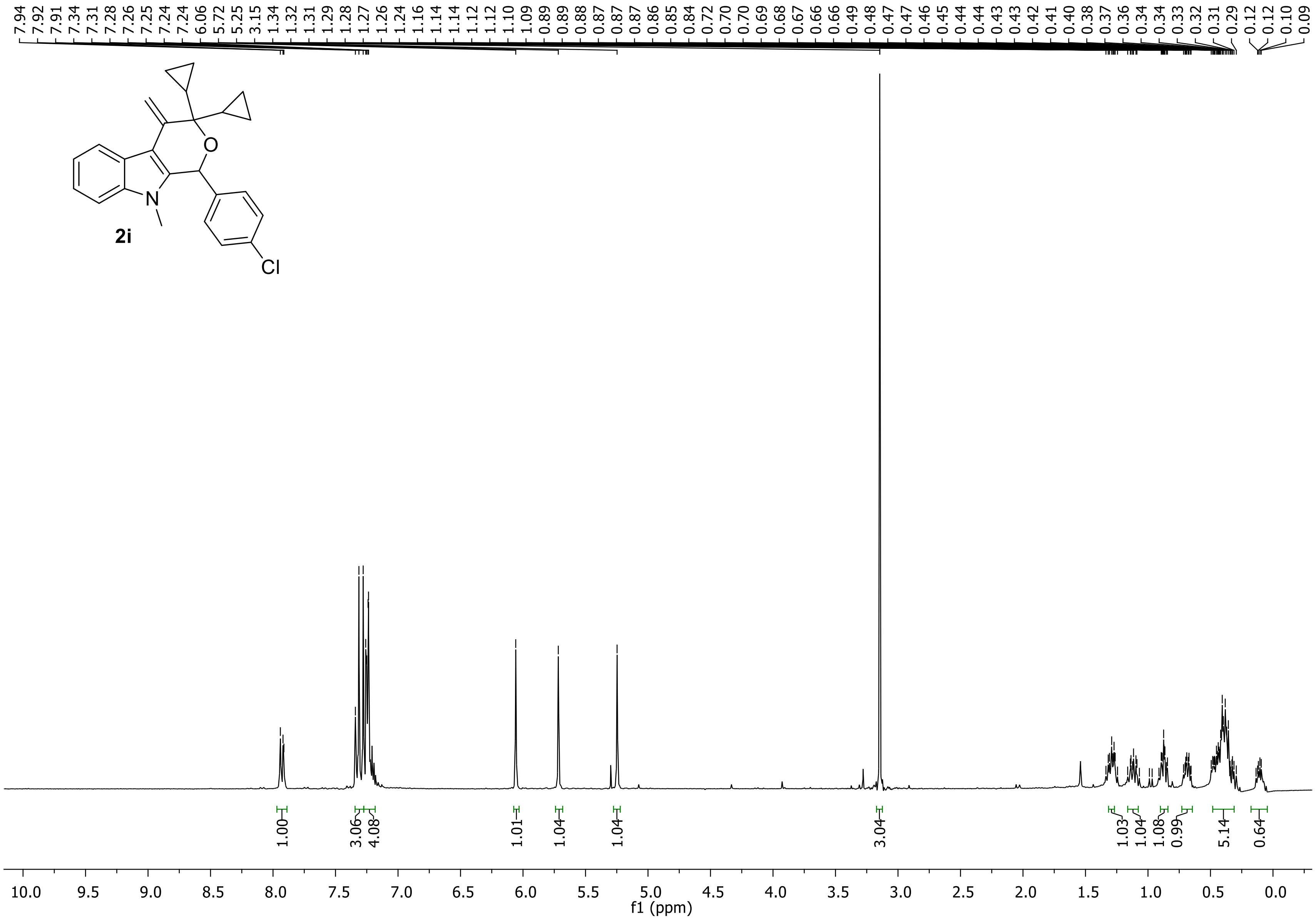

^{13}C NMR ($CDCl_3$, 75.4 MHz)

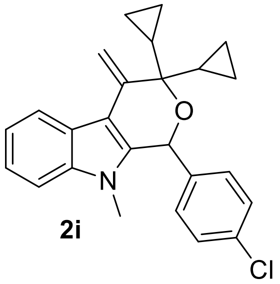

2i

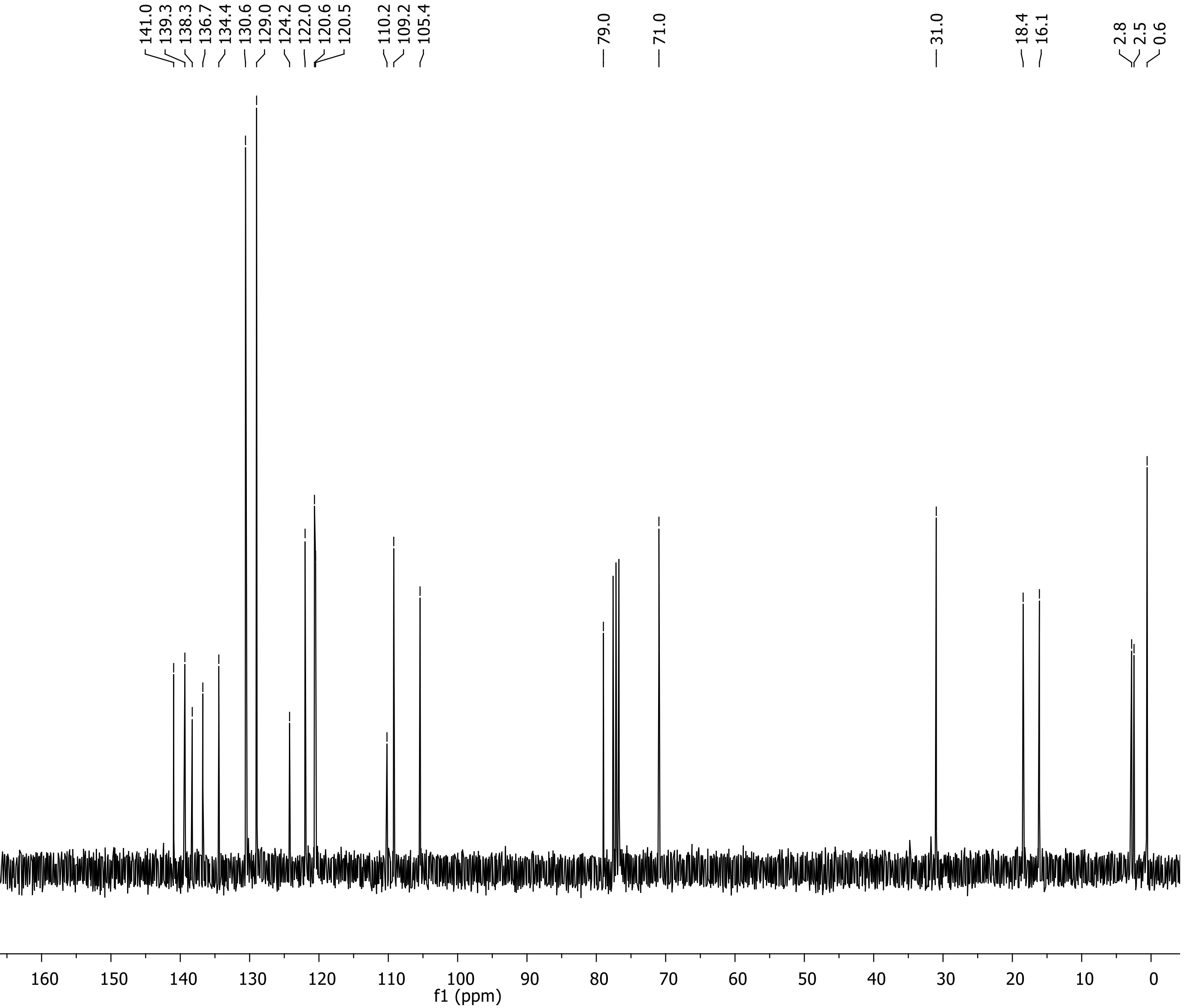

DEPT ($CDCl_3$, 75.4 MHz)

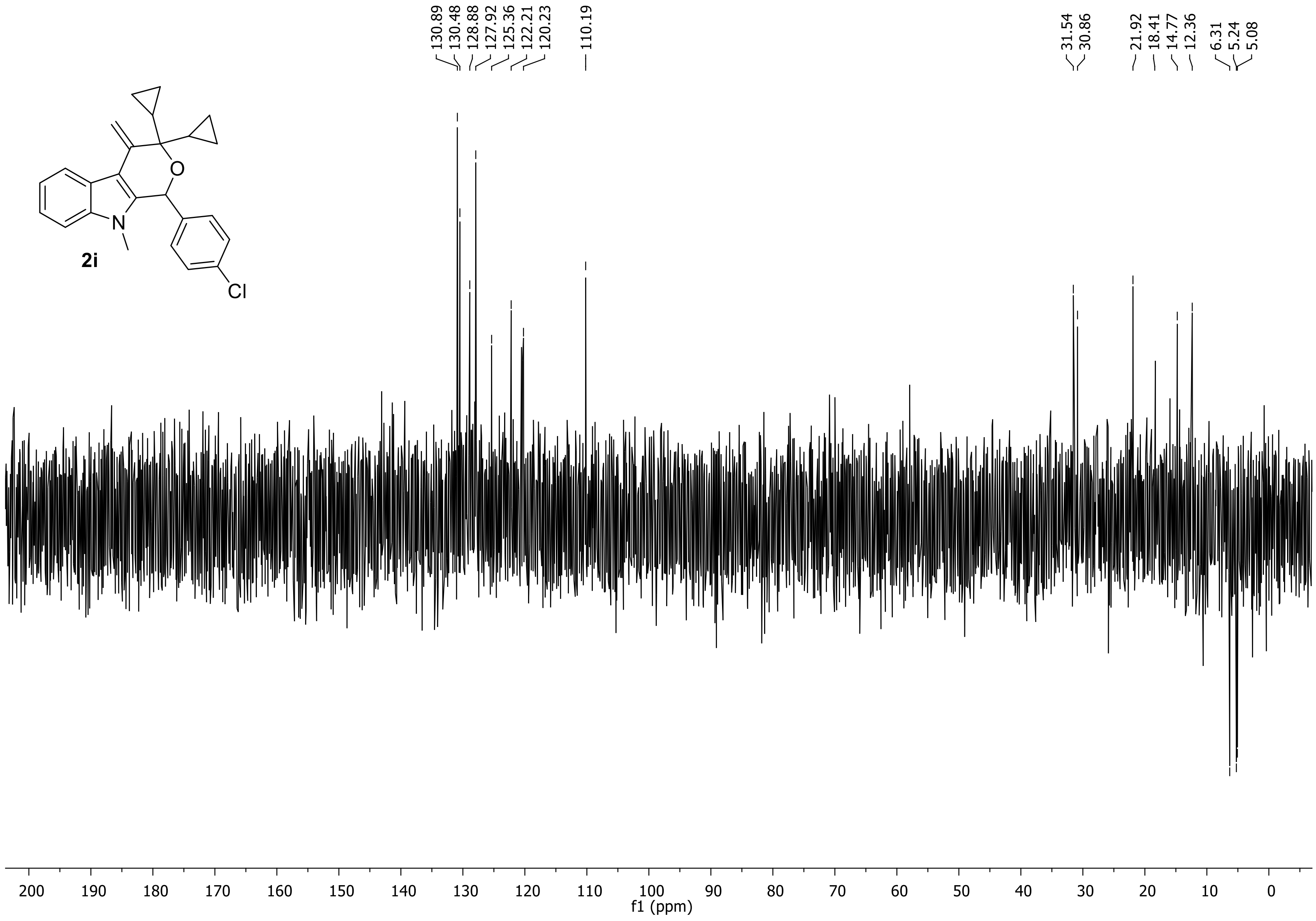

^{1}H NMR ((CD$_3$)$_2$O, 300 MHz)

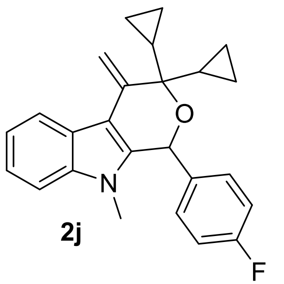

2j

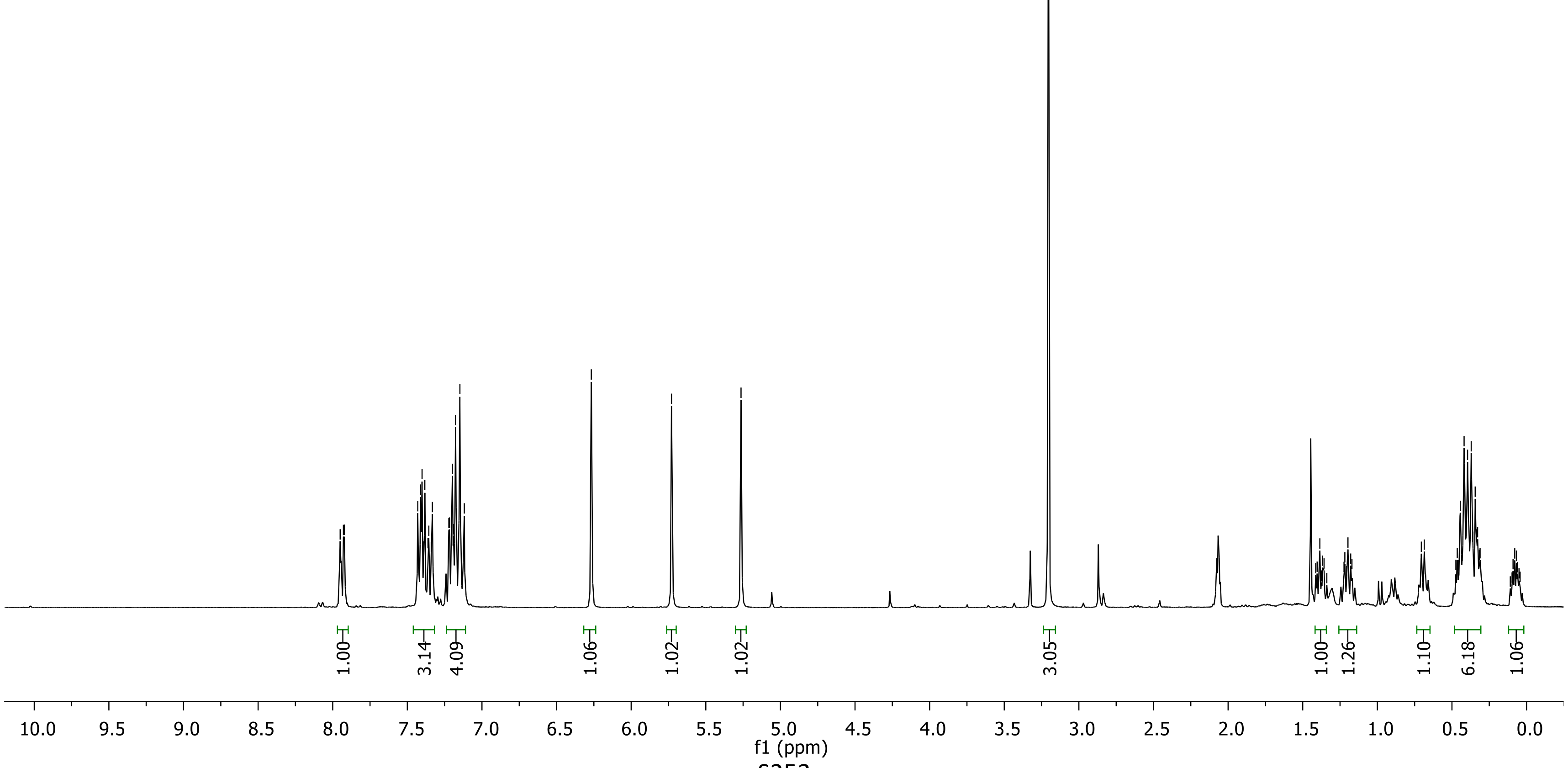

^{13}C NMR $((CD_3)_2O$, 75.4 MHz)

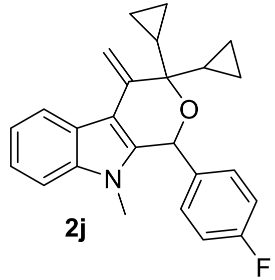

2j

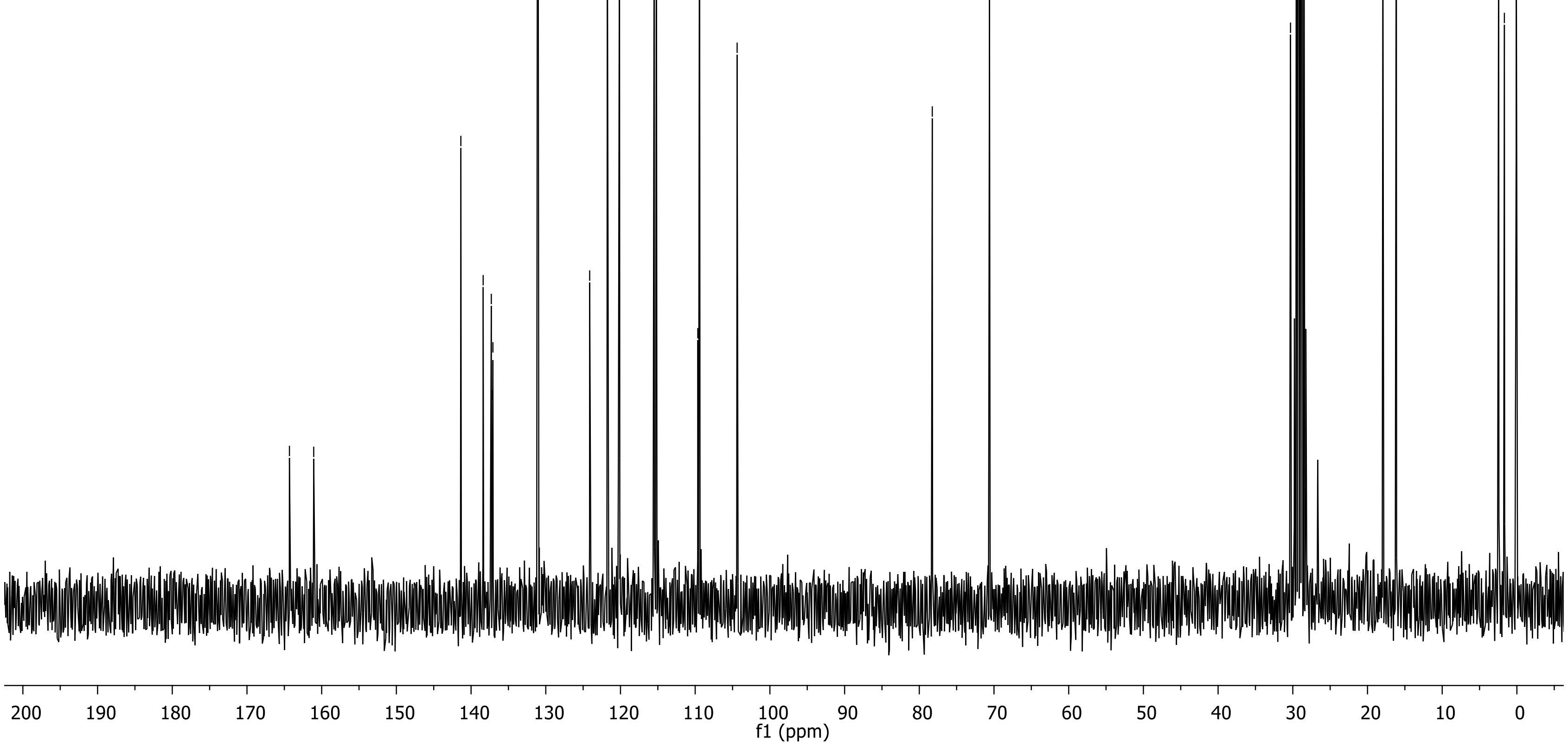

DEPT ($(CD_3)_2O$, 75.4 MHz)

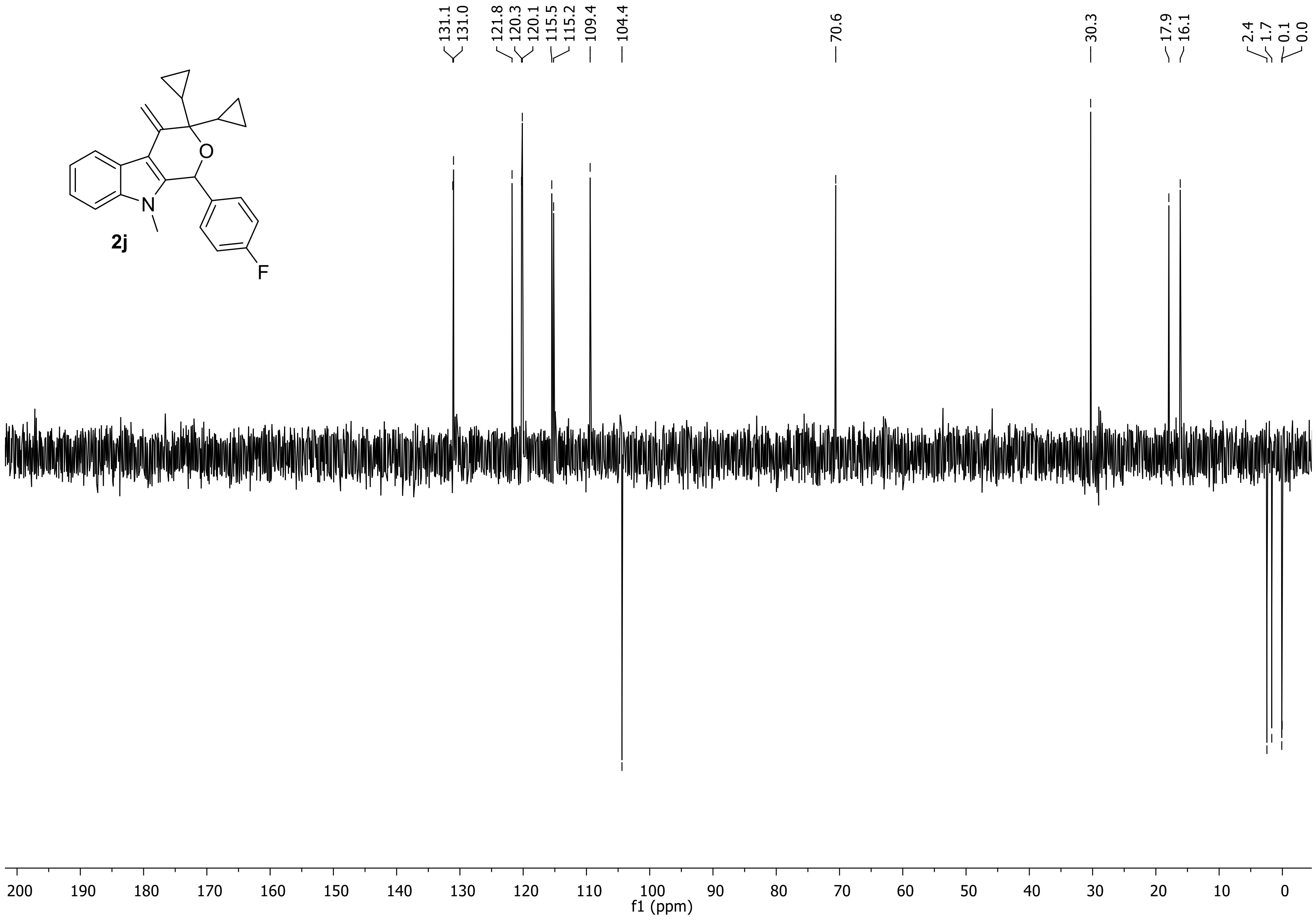

^{1}H NMR ($CDCl_3$, 300 MHz)

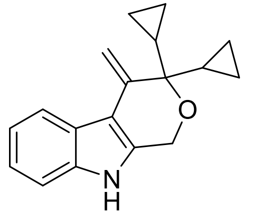

2k

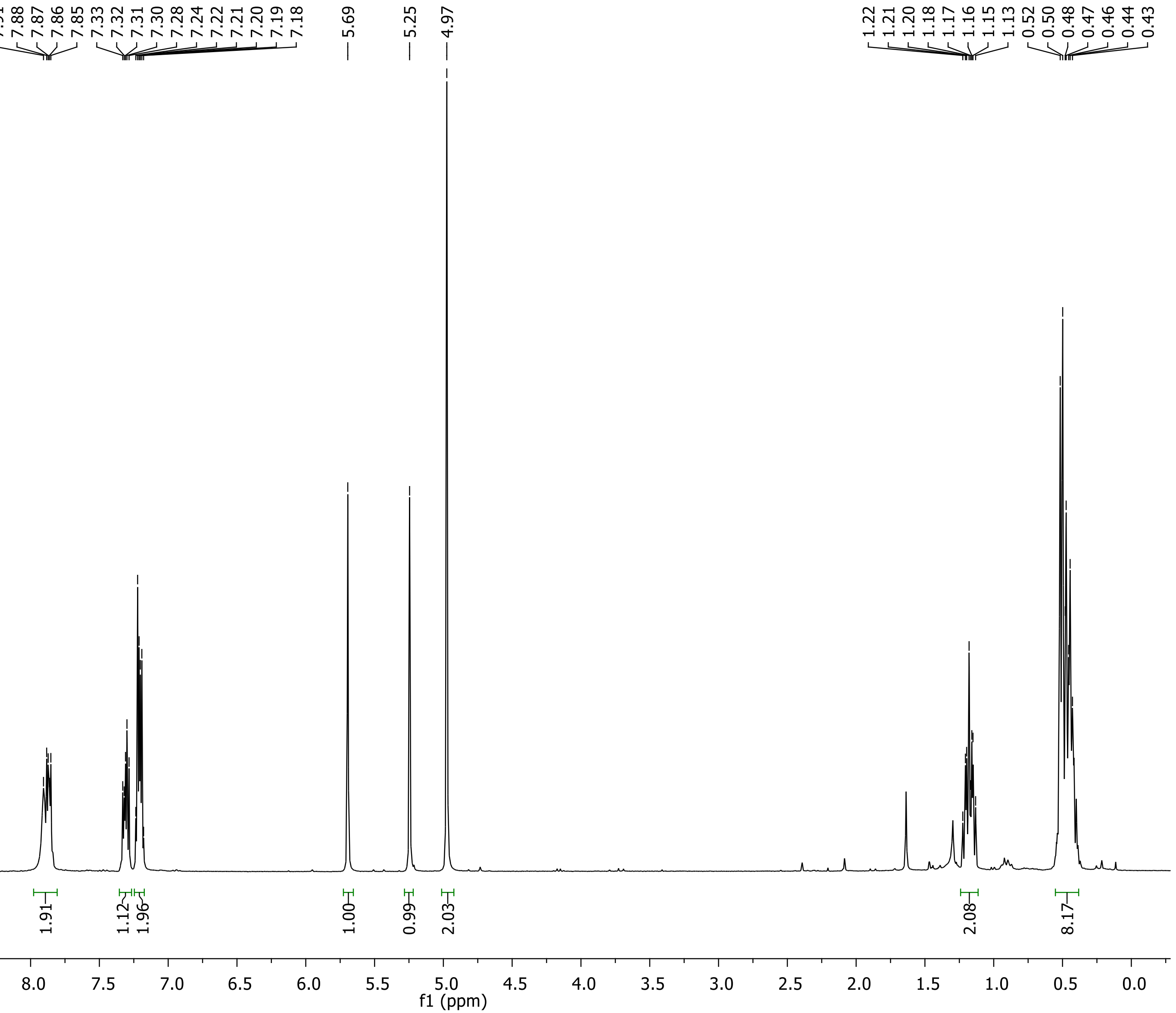

^{13}C NMR ($CDCl_3$, 75.4 MHz)

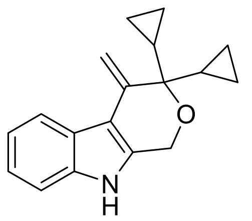

2k

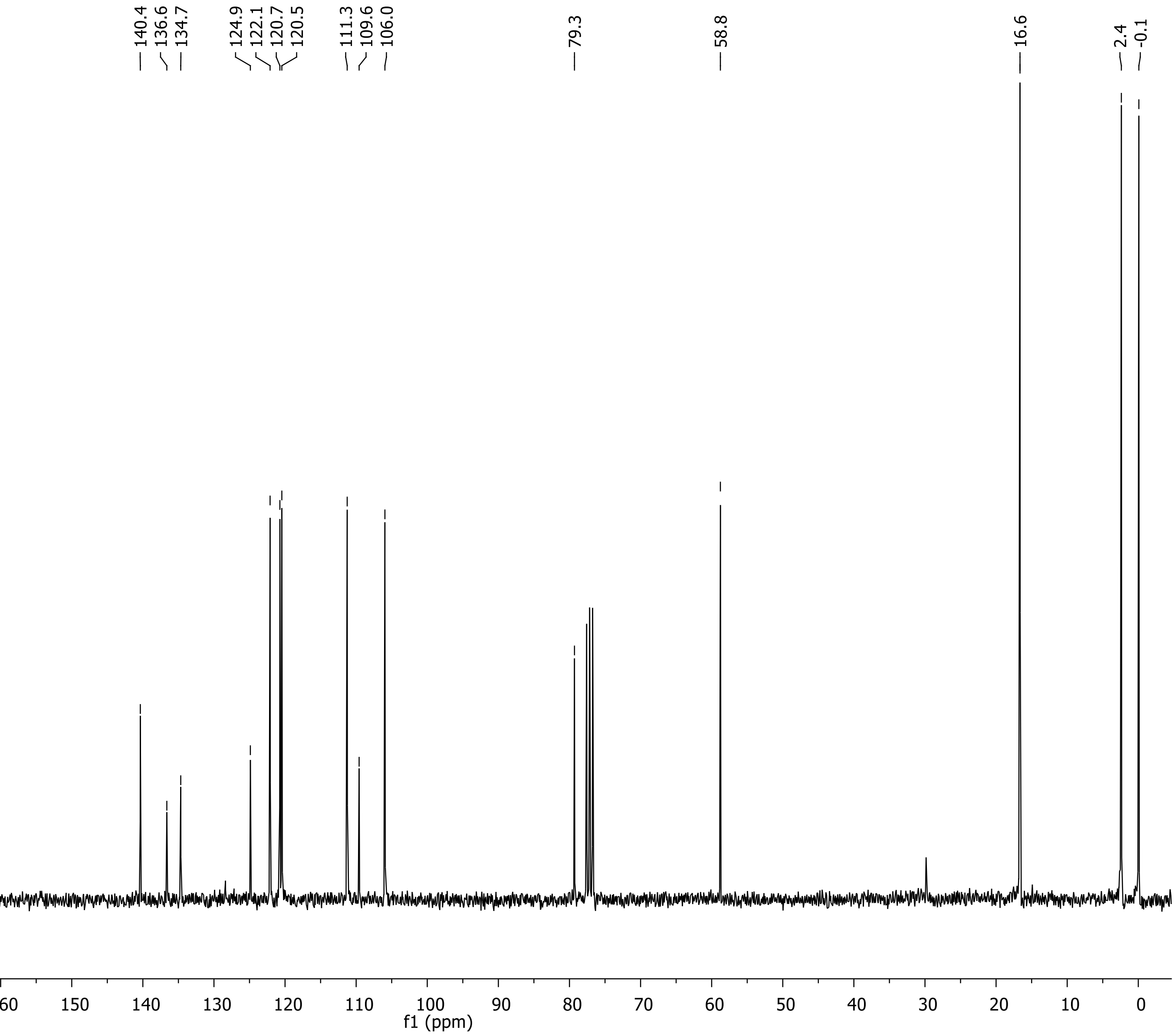

DEPT ($CDCl_3$, 75.4 MHz)

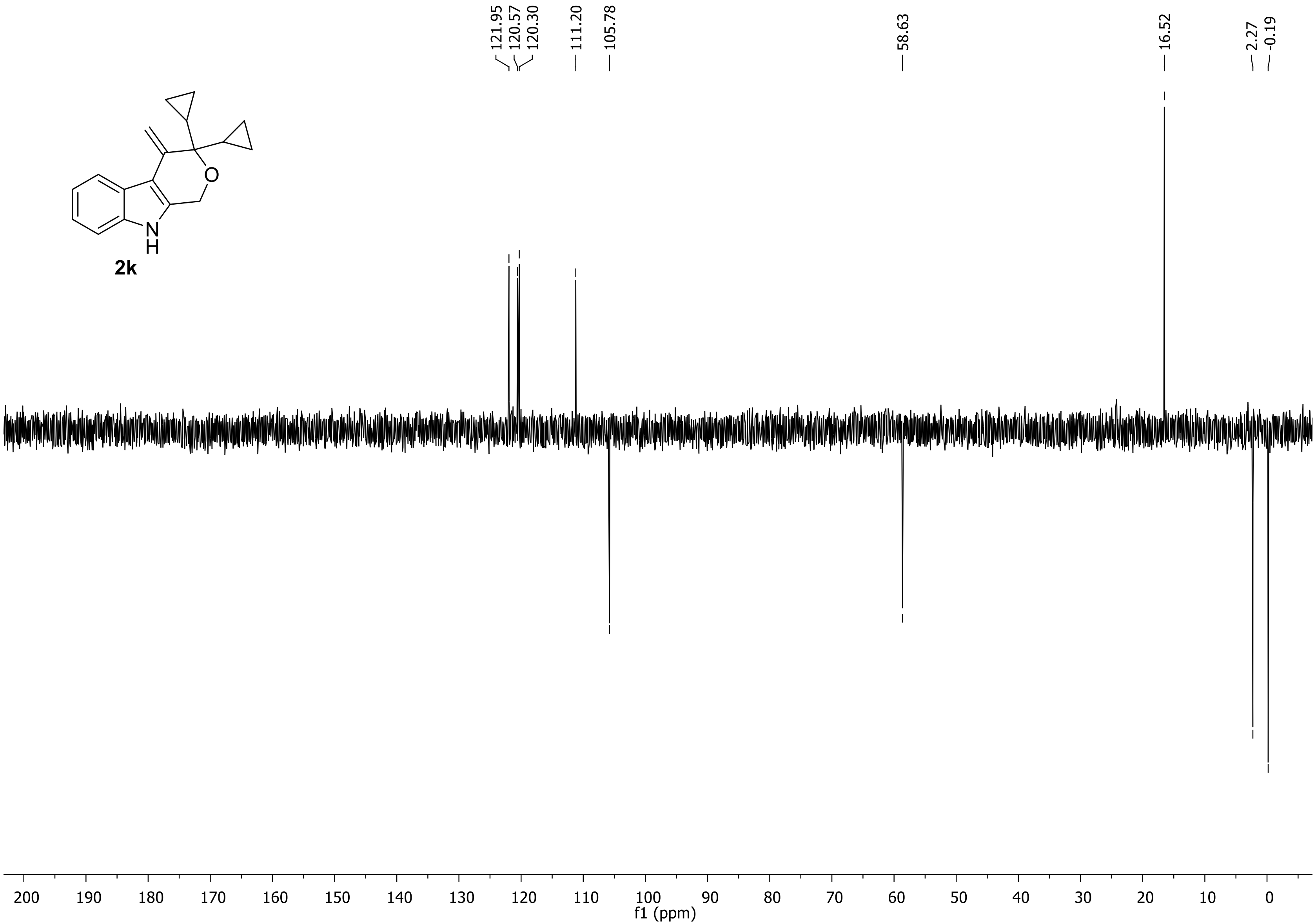

2k

^{1}H NMR ((CD_3)$_2$O, 300 MHz)

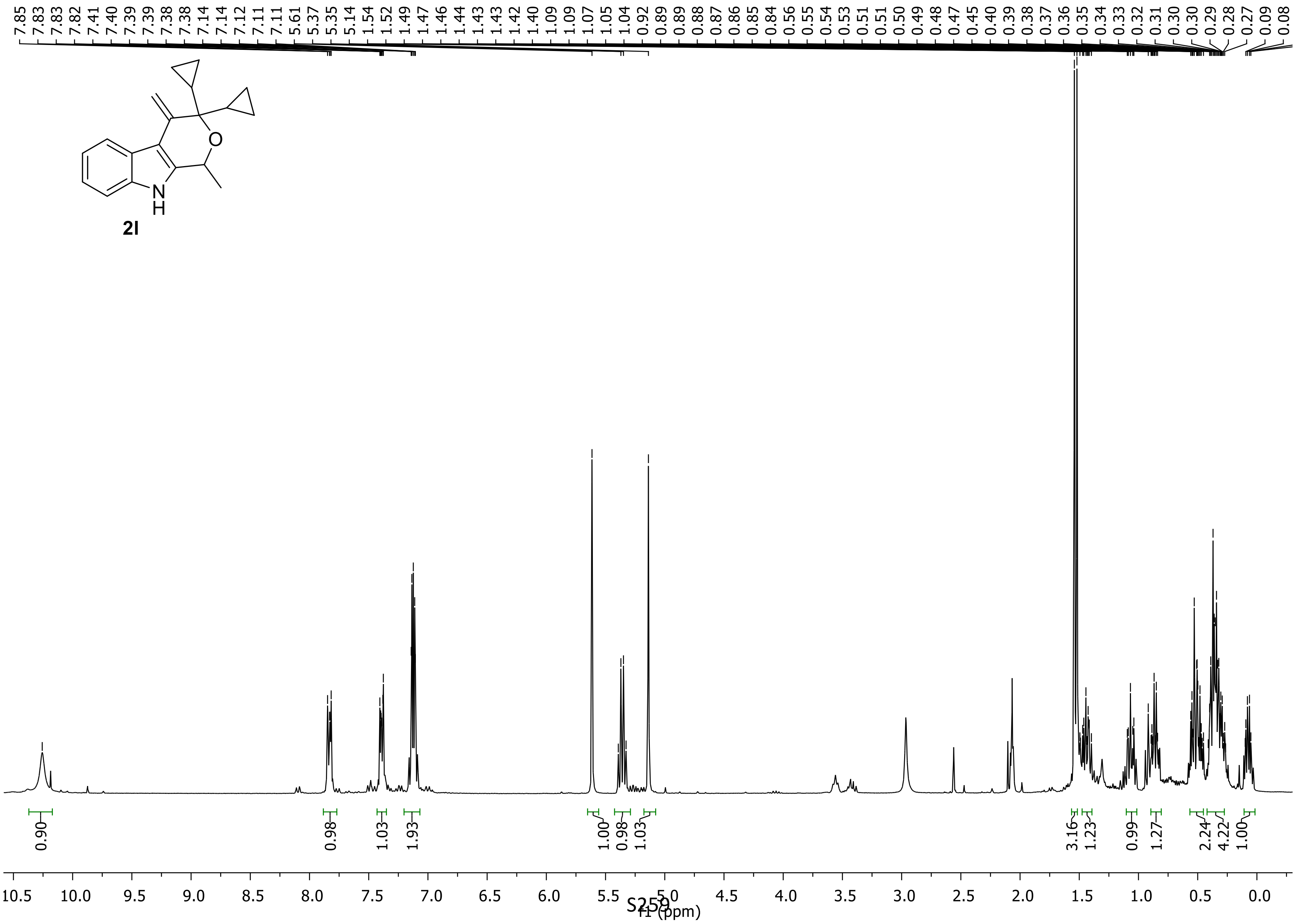

^{13}C NMR ($(CD_3)_2O$, 75.4 MHz)

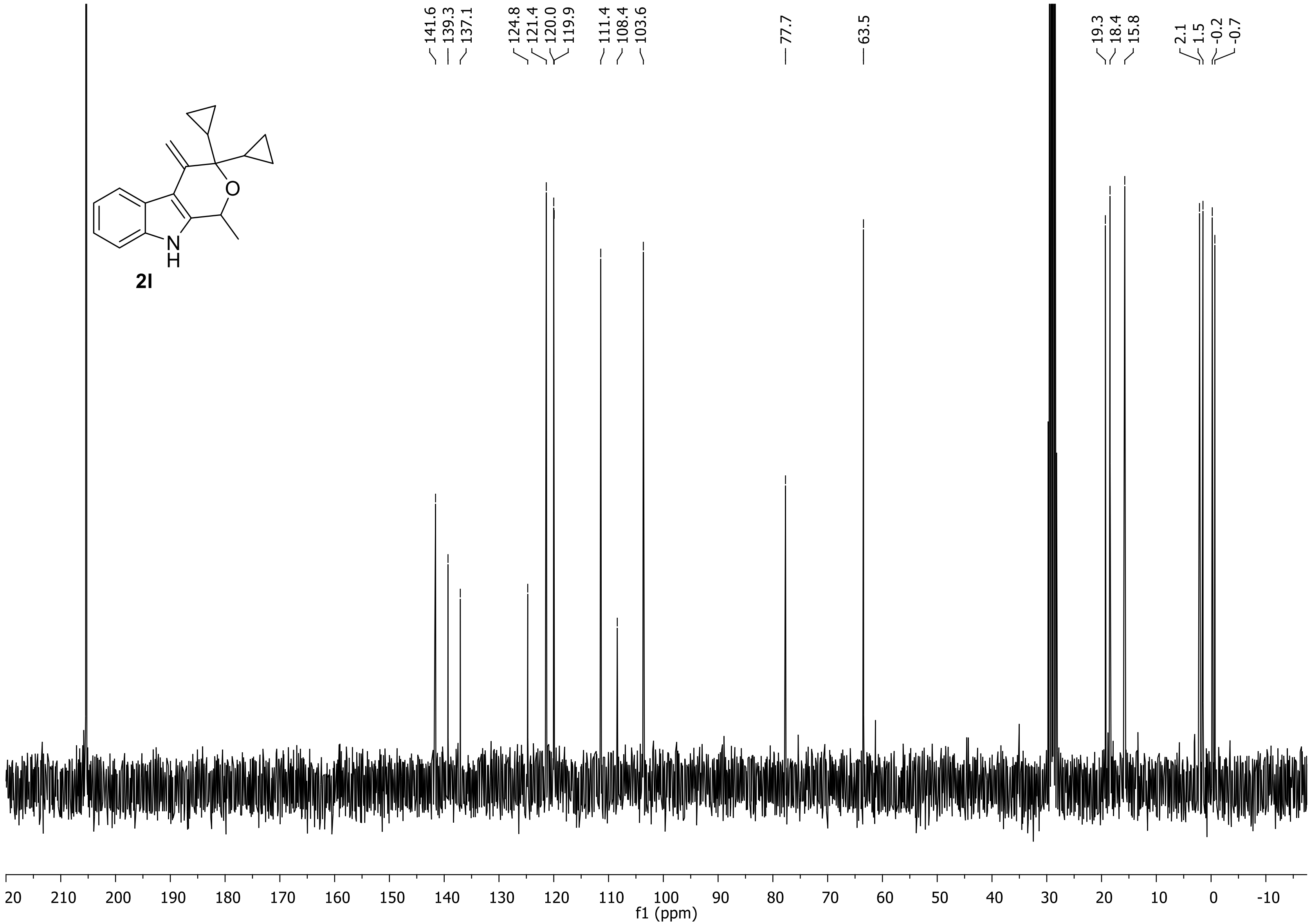

DEPT ($(CD_3)_2O$, 75.4 MHz)

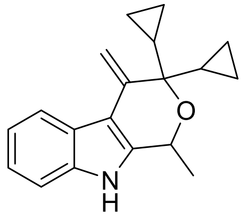

2l

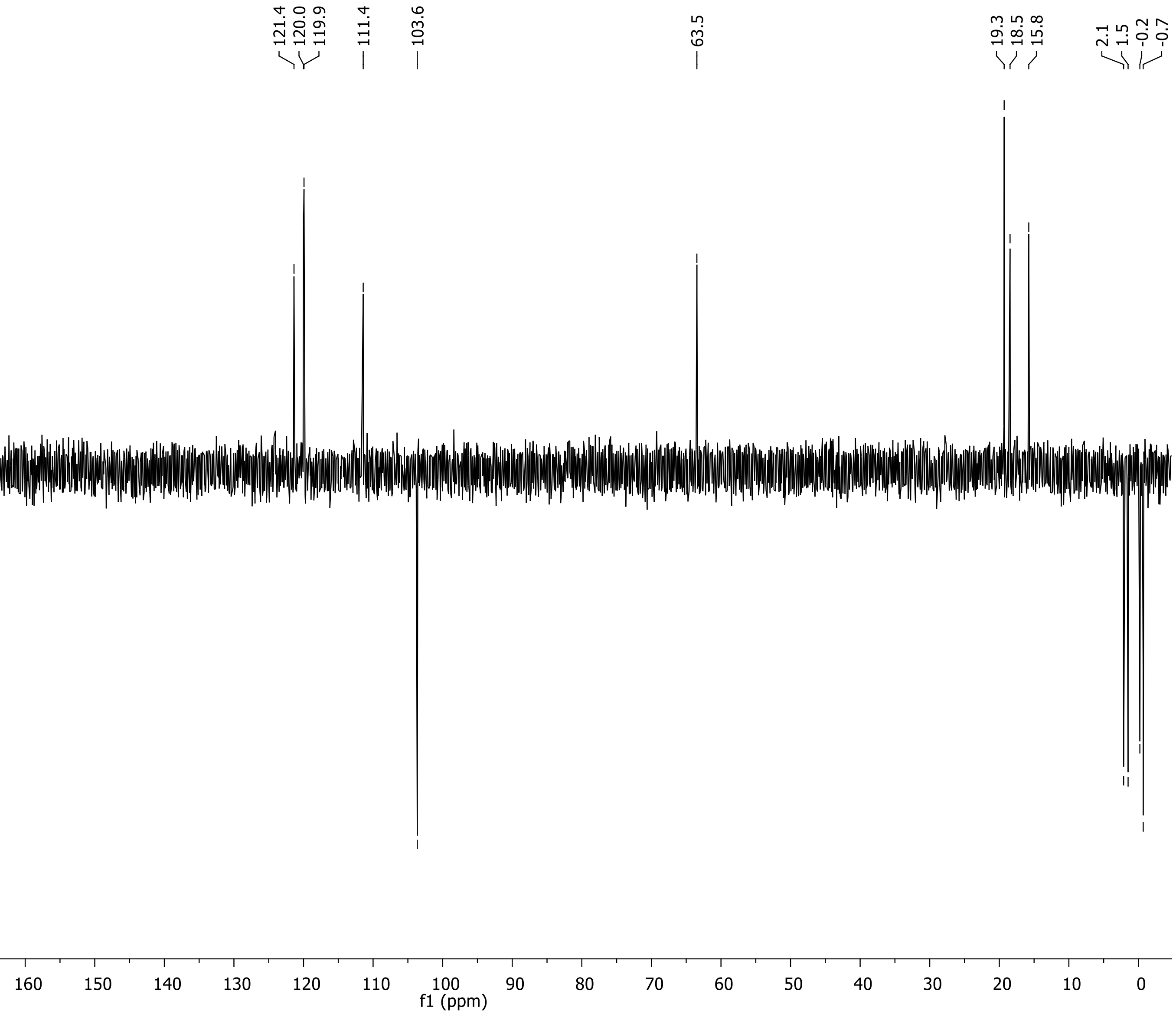

^{1}H NMR ($CDCl_3$, 300 MHz)

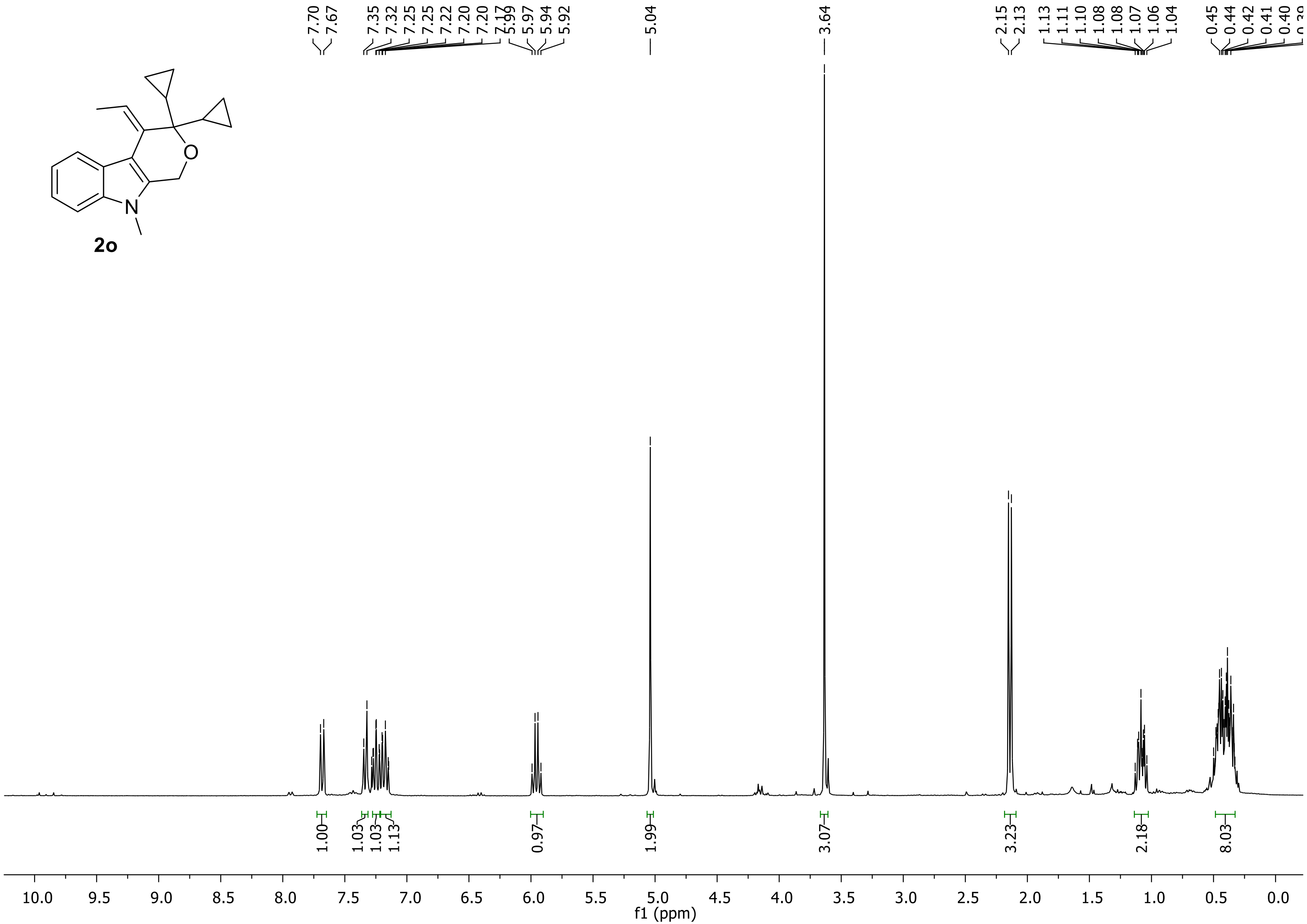

^{13}C NMR ($CDCl_3$, 75.4 MHz)

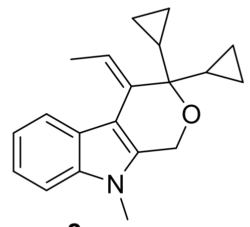

2o

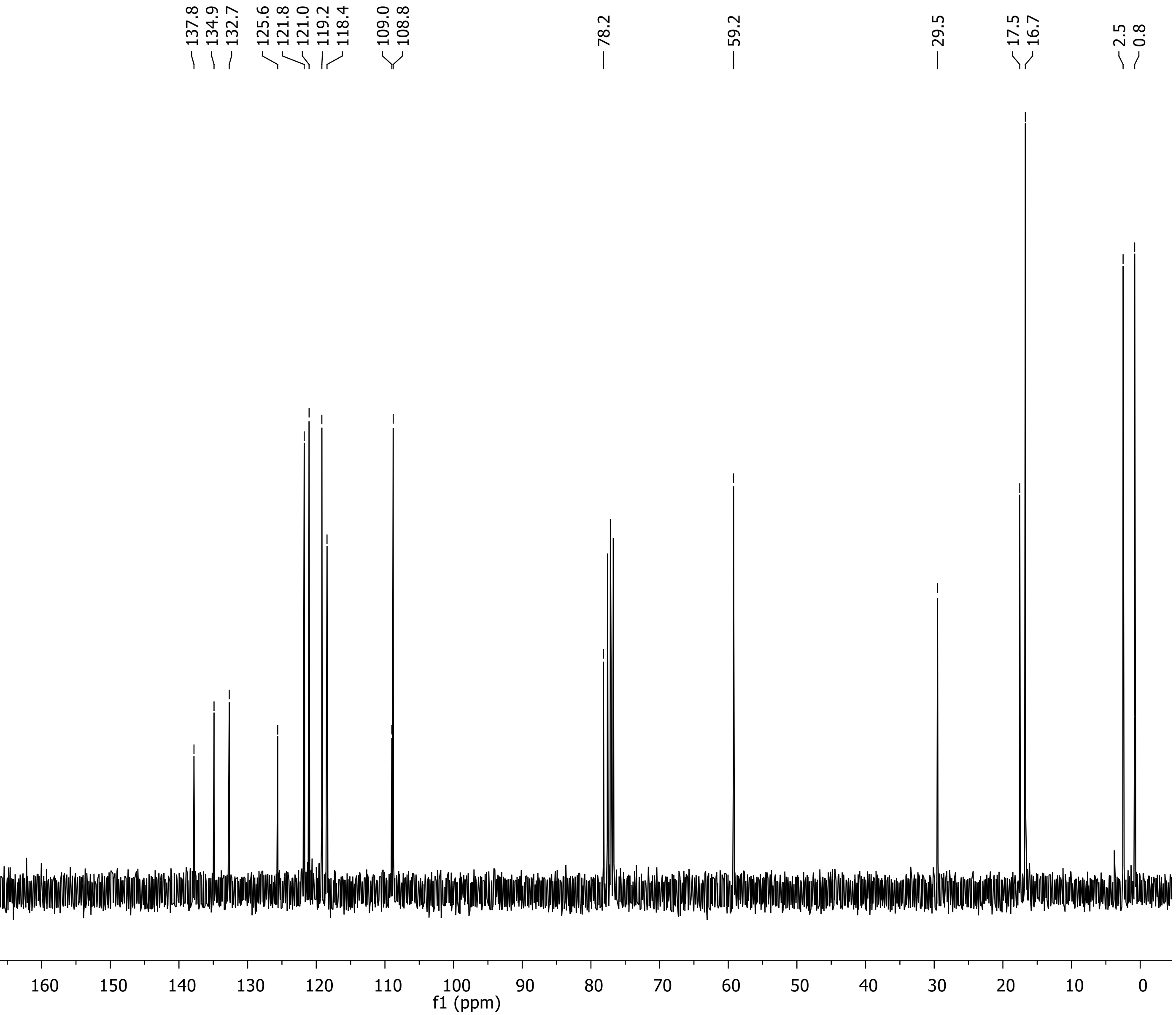

DEPT ($CDCl_3$, 75.4 MHz)

2o

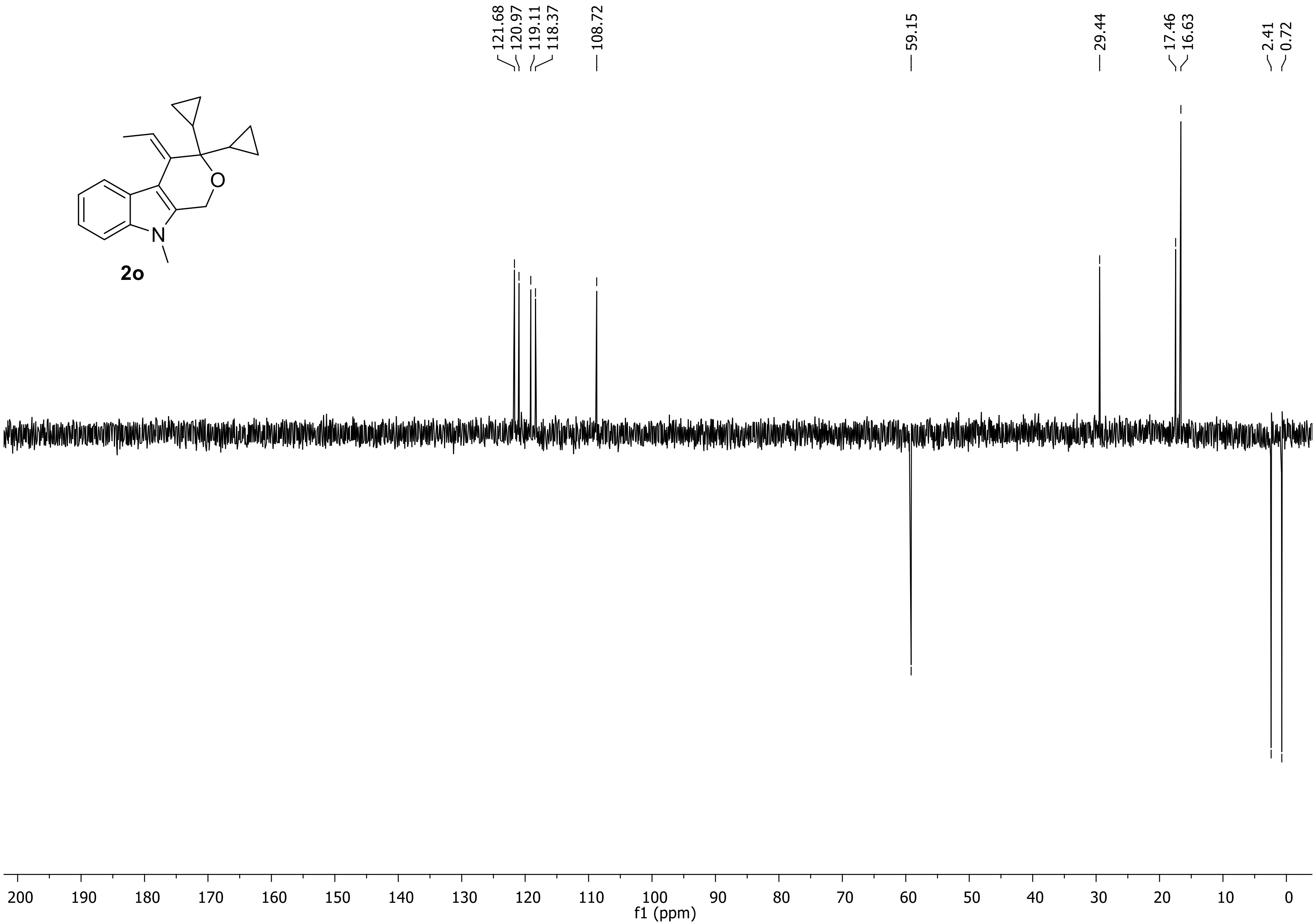

NOE-1D ($CDCl_3$, 300 MHz)

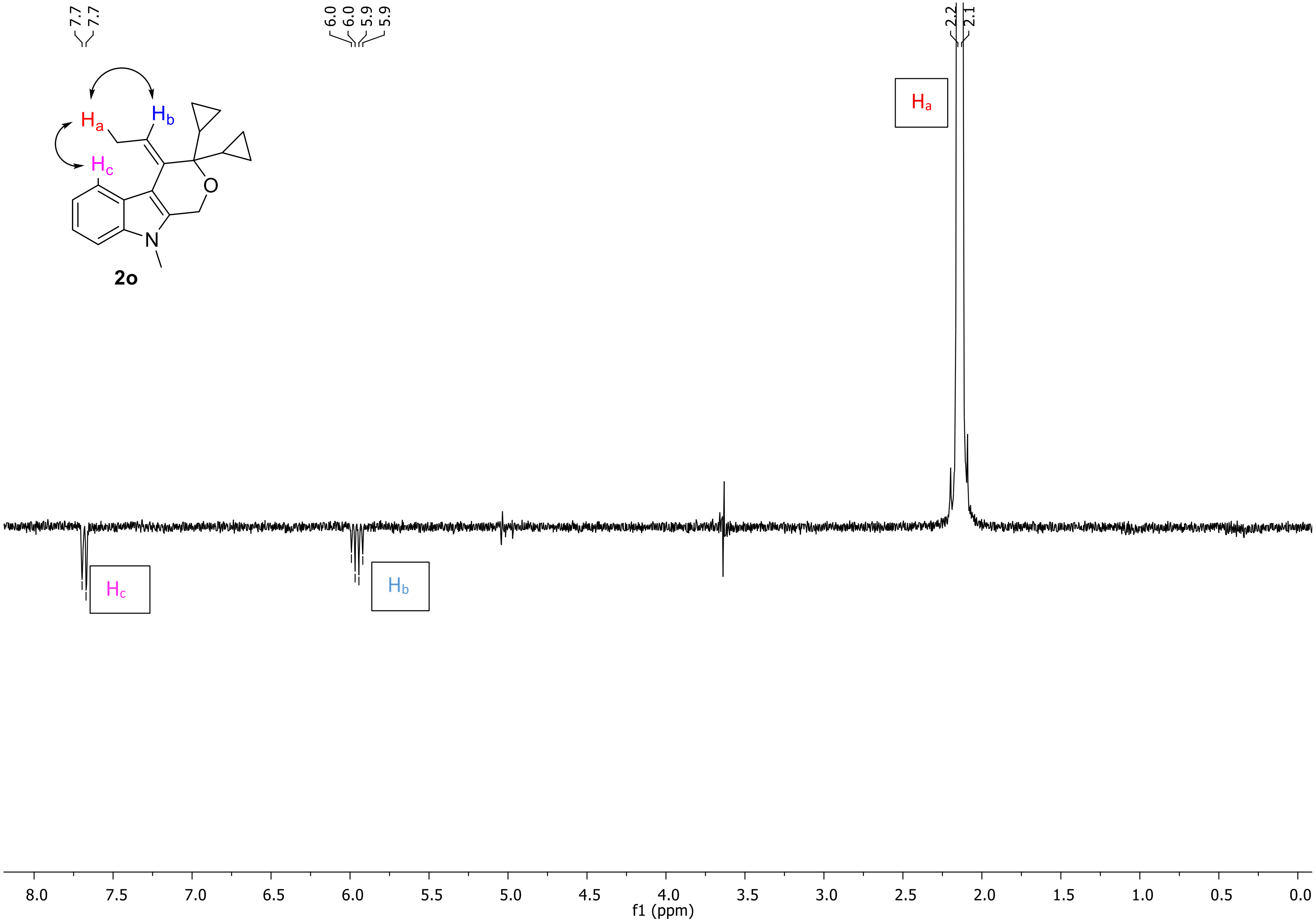

^{1}H NMR (CDCl$_3$, 300 MHz)

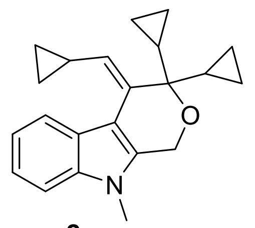

2p

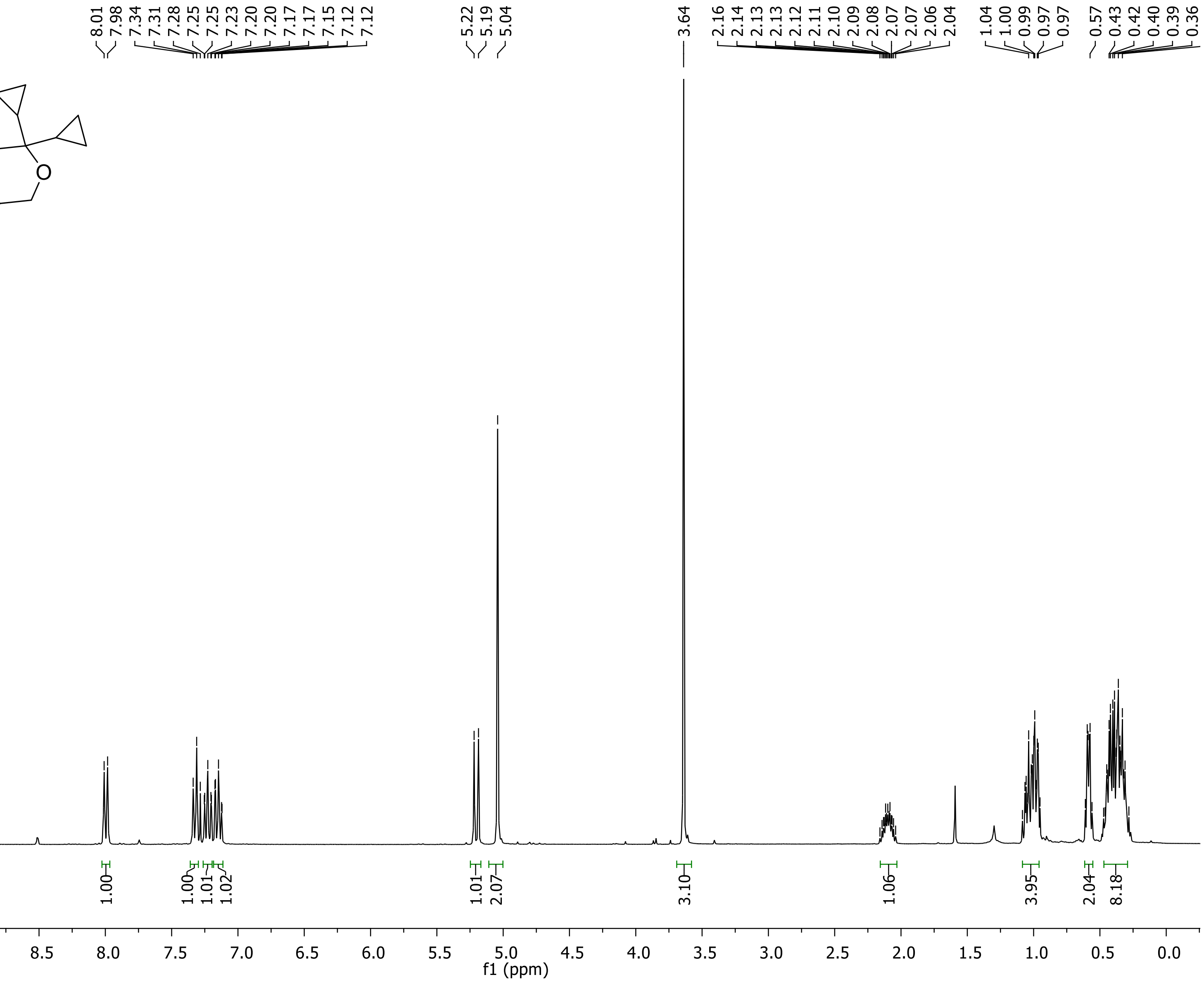

^{13}C NMR (CDCl$_3$, 75.4 MHz)

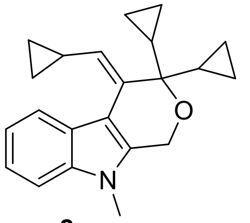

2p

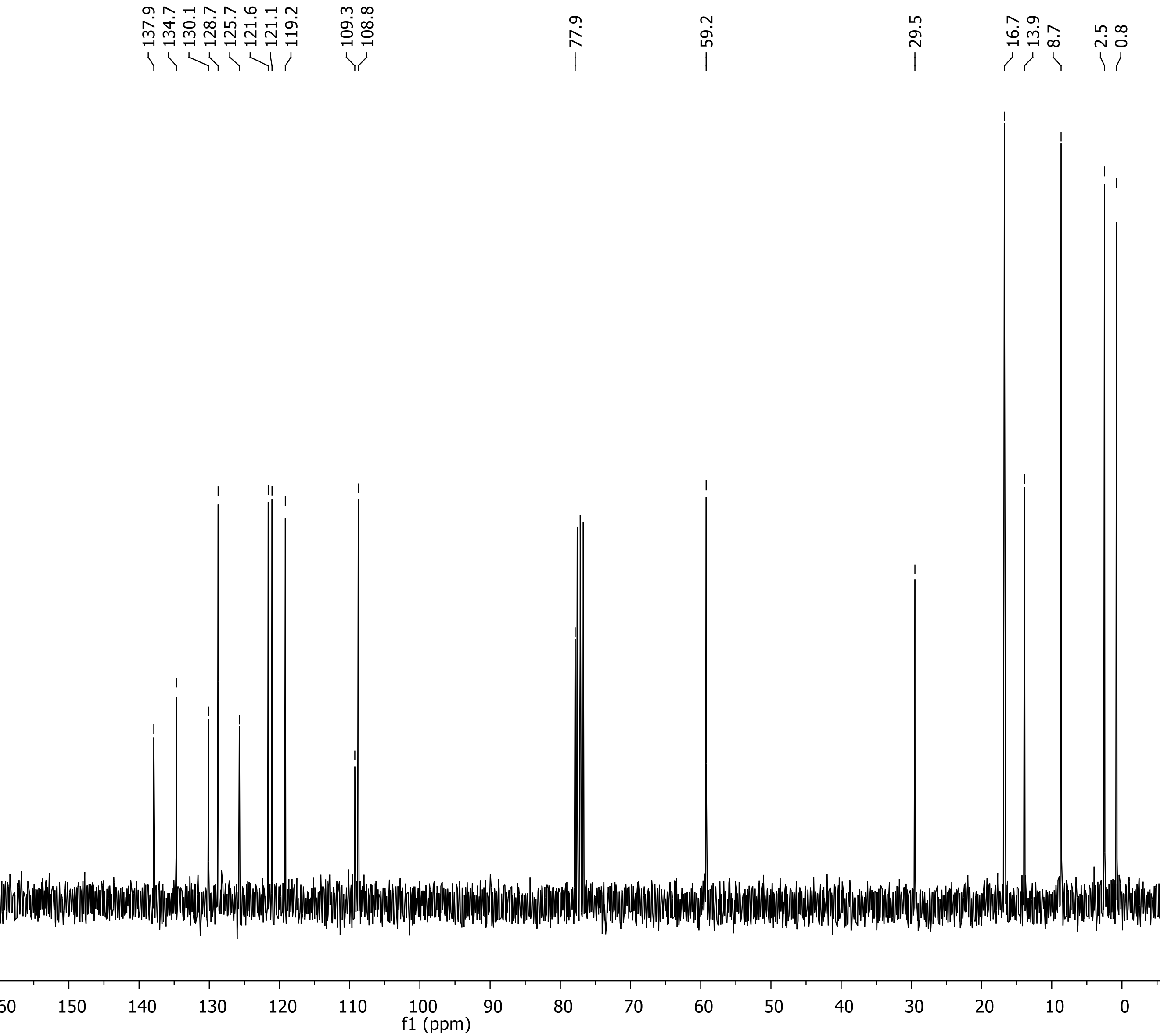

DEPT (CDCl$_3$, 75.4 MHz)

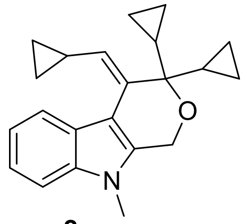

2p

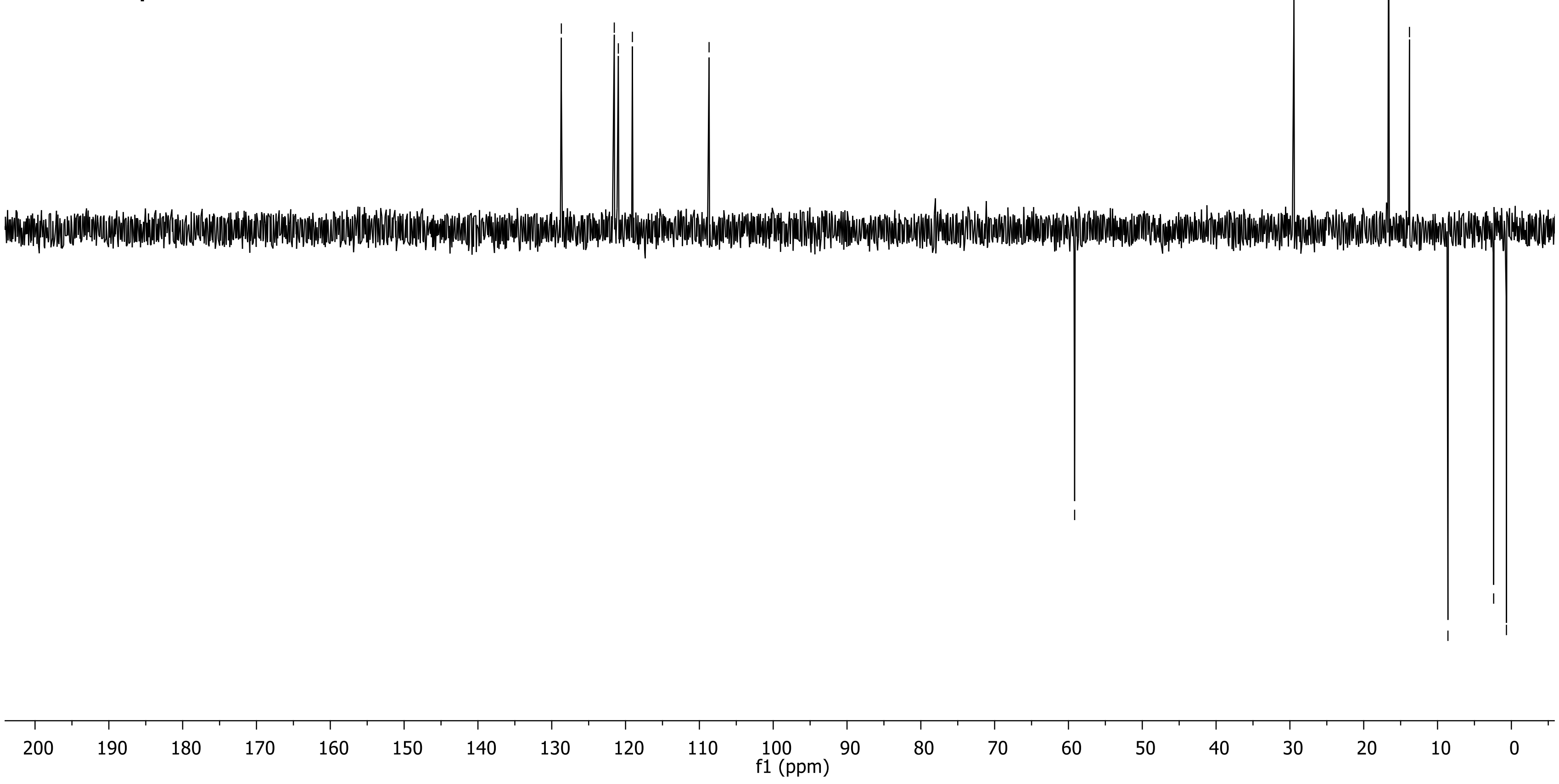

^{1}H NMR ($CDCl_3$, 300 MHz)

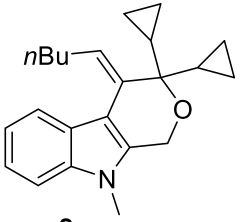

2q

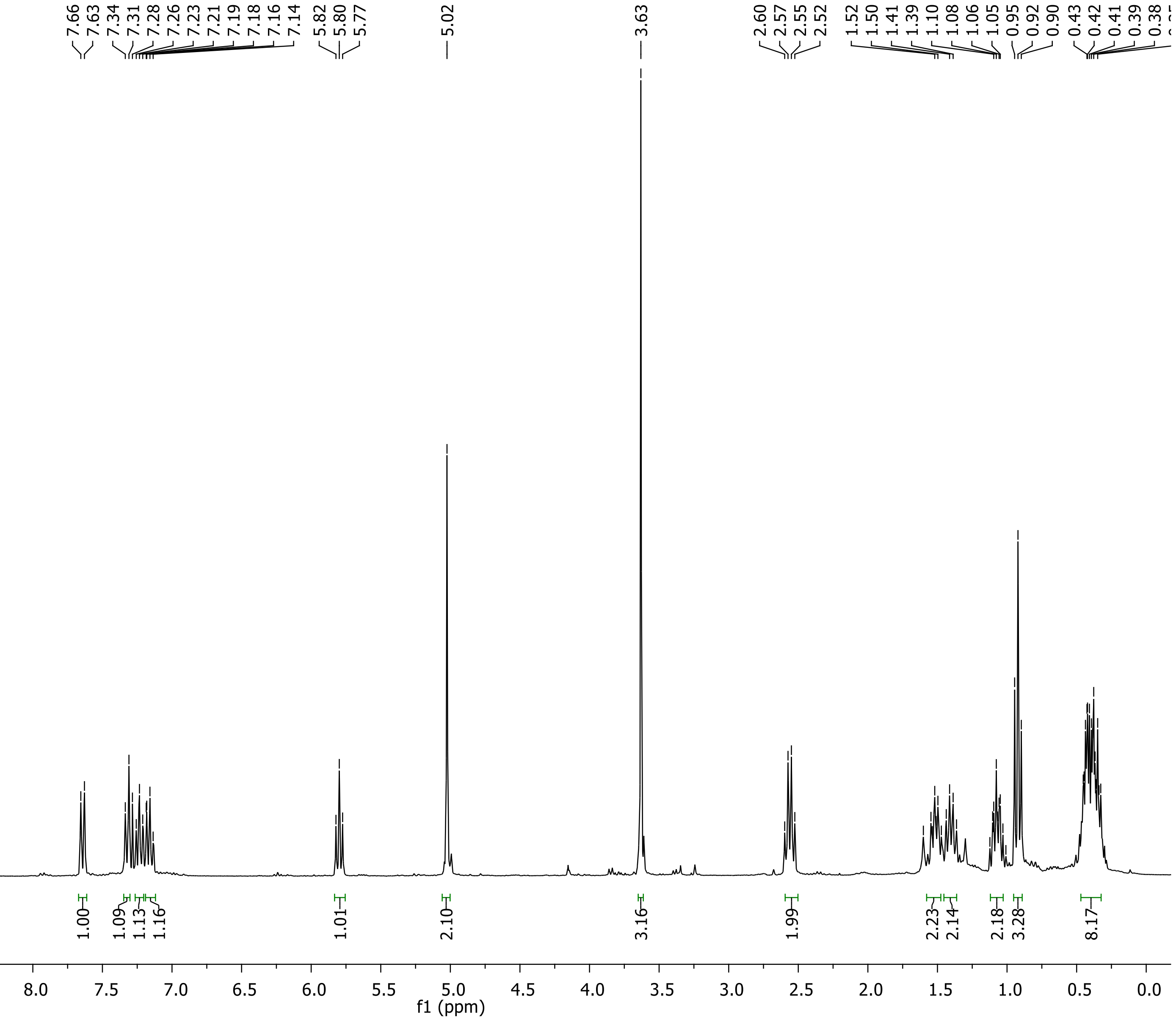

^{13}C NMR ($CDCl_3$, 75.4 MHz)

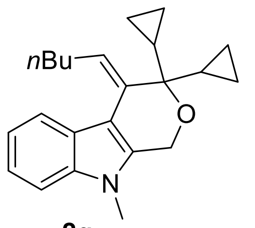

2q

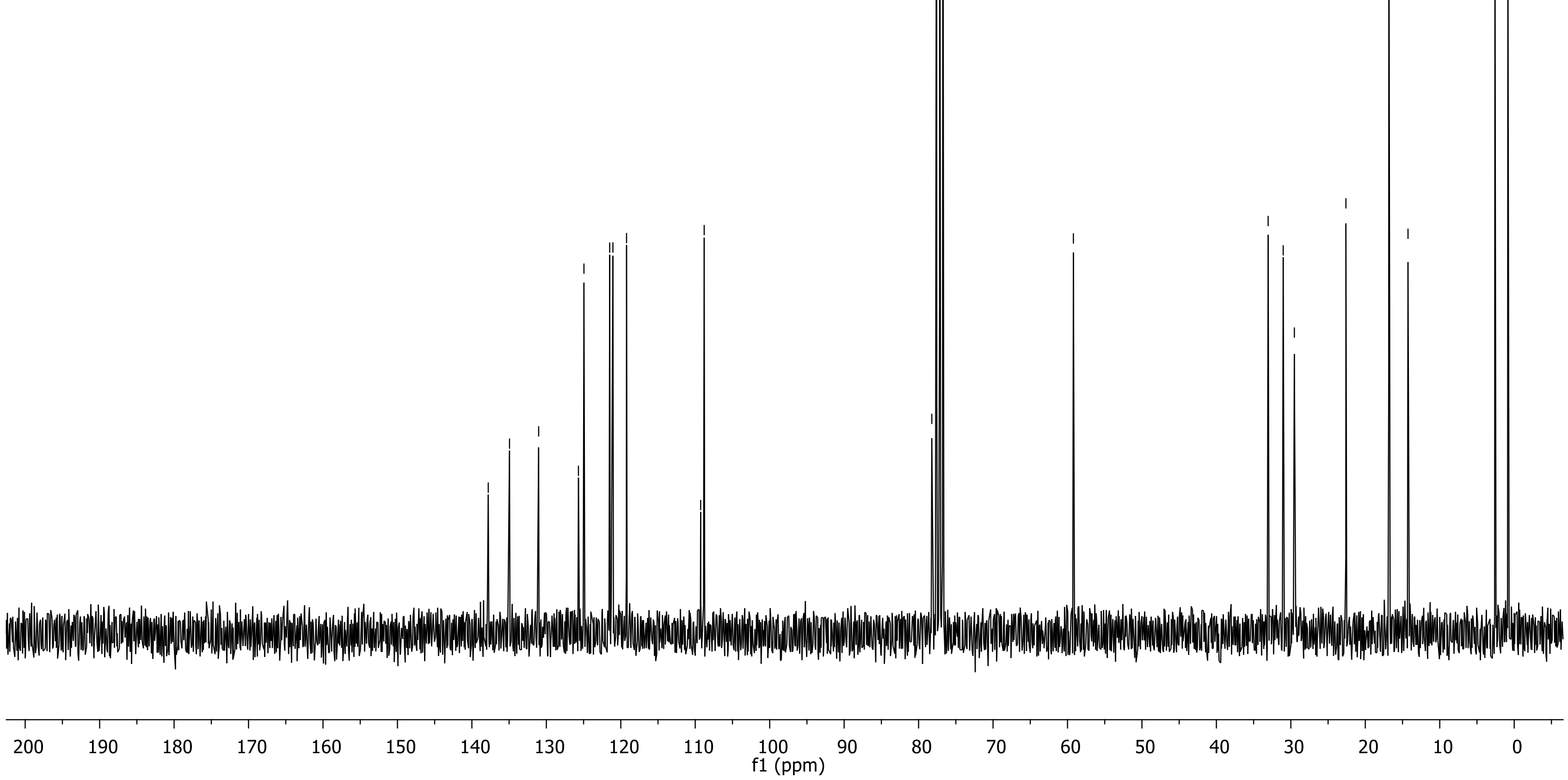

DEPT ($CDCl_3$, 75.4 MHz)

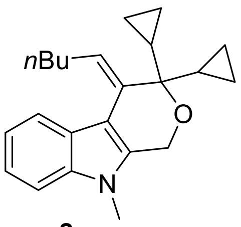

2q

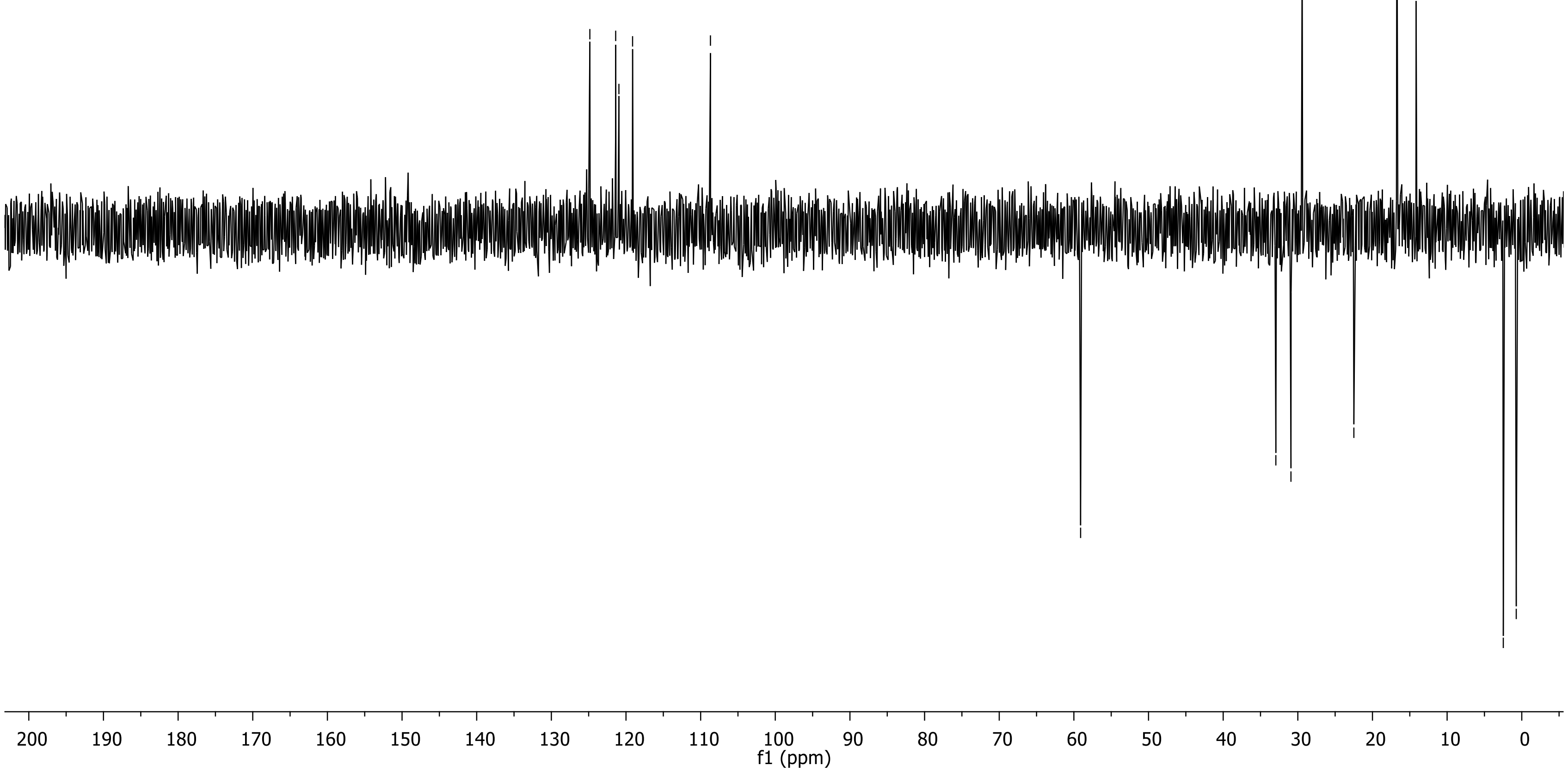

^{1}H NMR ($CDCl_3$, 300 MHz)

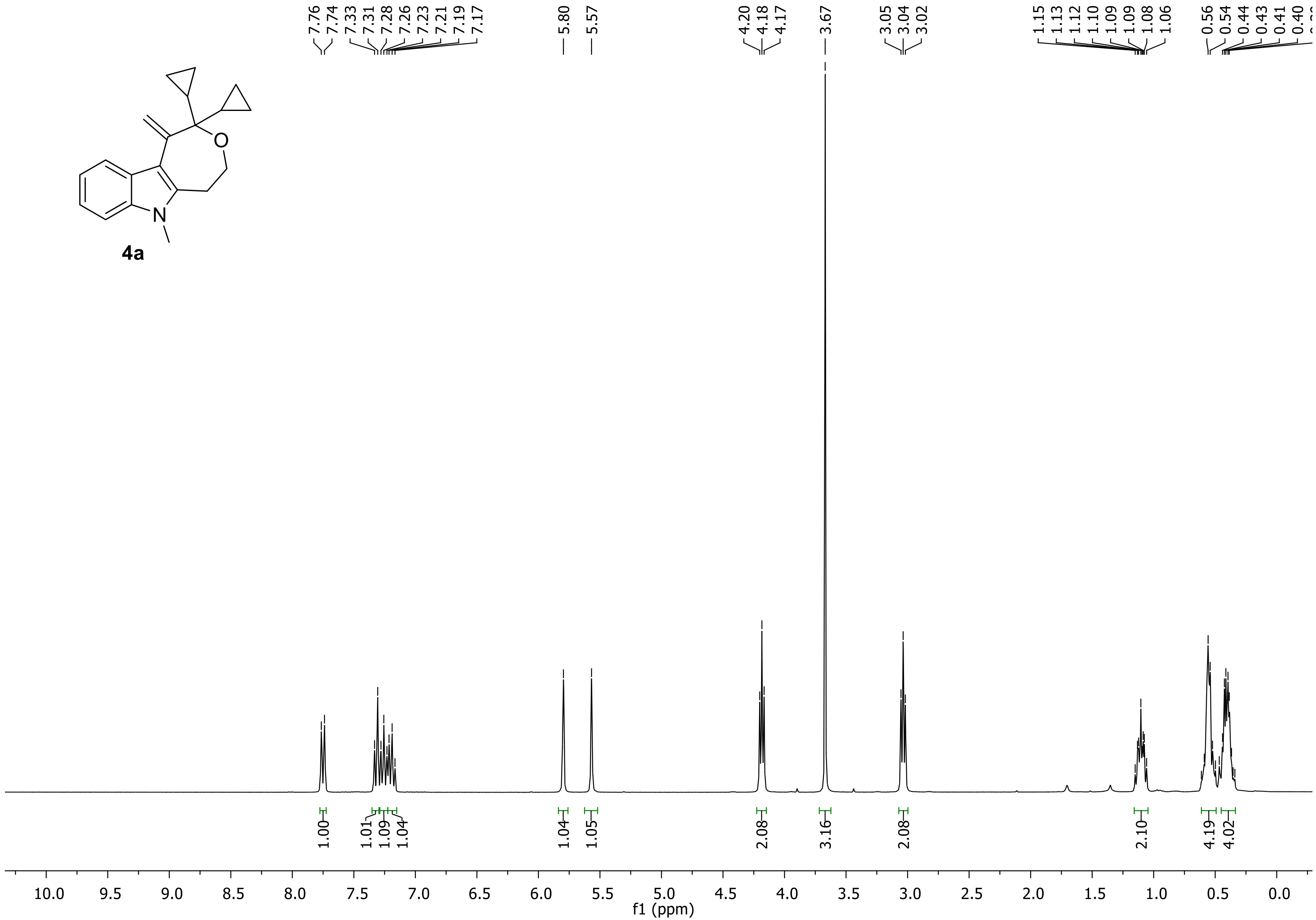

^{13}C NMR ($CDCl_3$, 75.4 MHz)

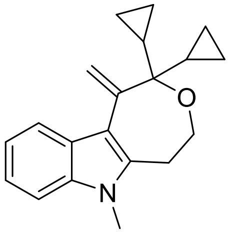

4a

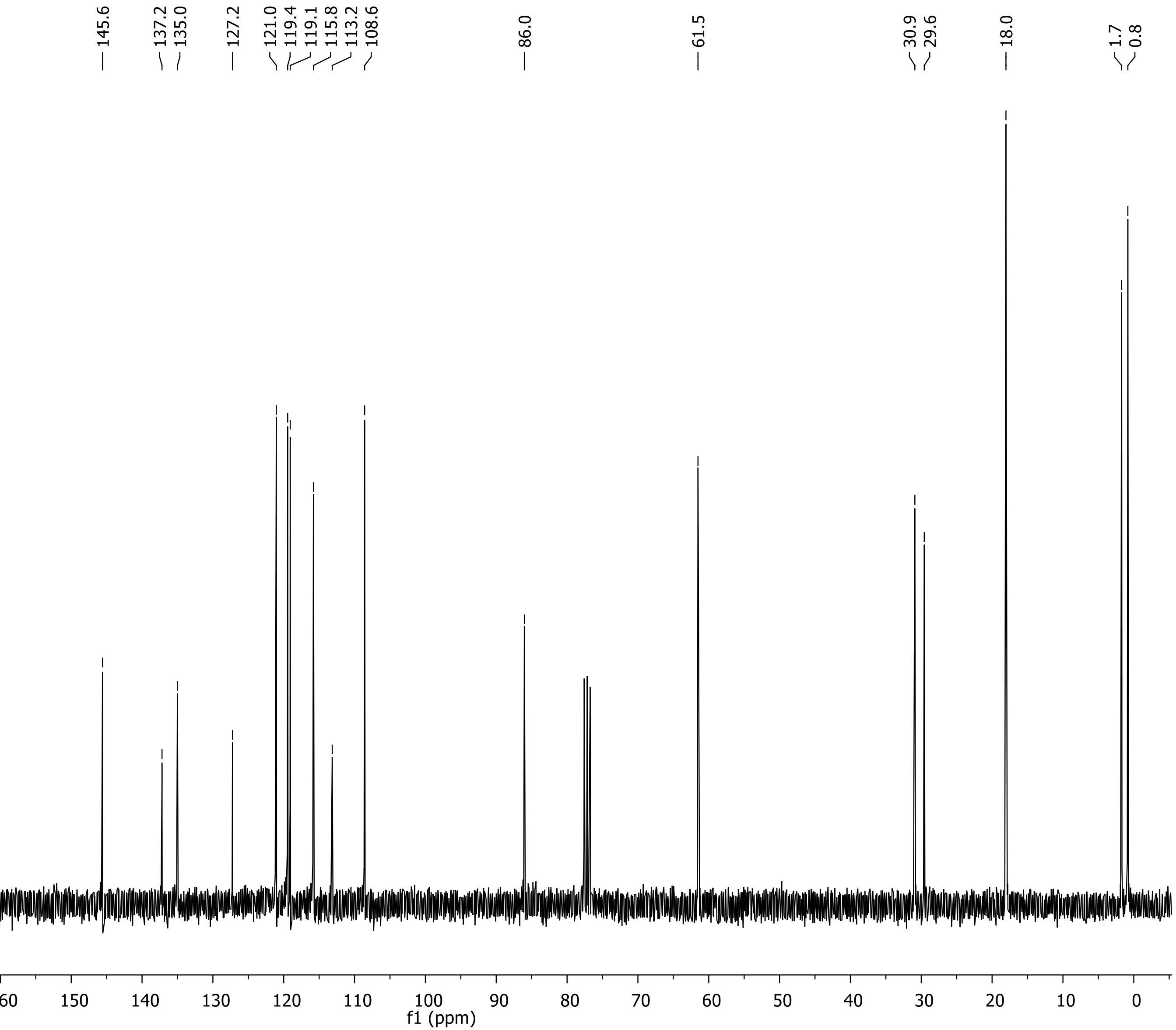

DEPT ($CDCl_3$, 75.4 MHz)

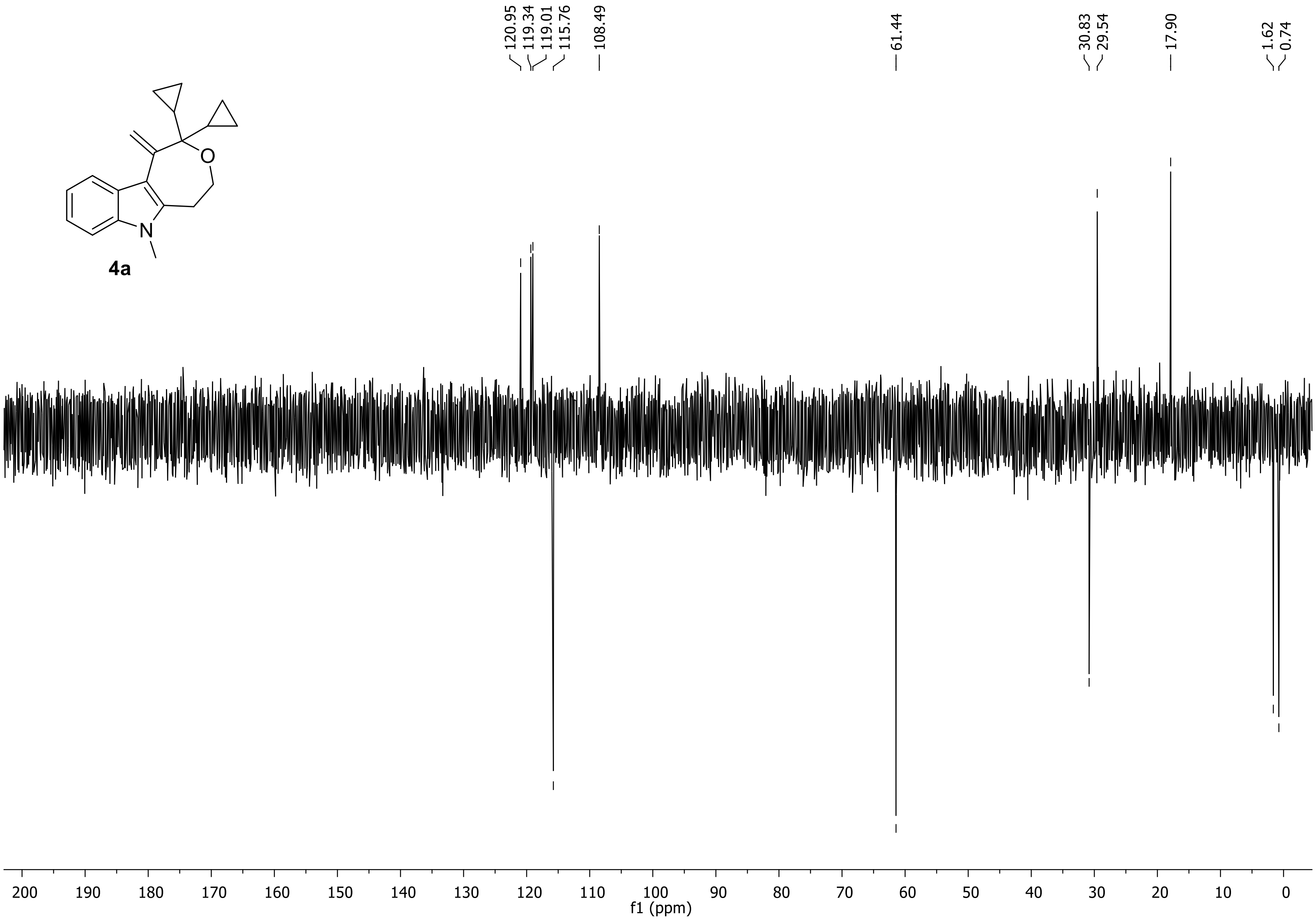

^{1}H NMR ($CDCl_3$, 300 MHz)

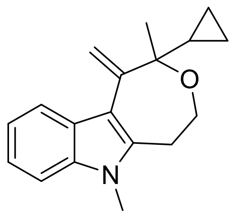

4b

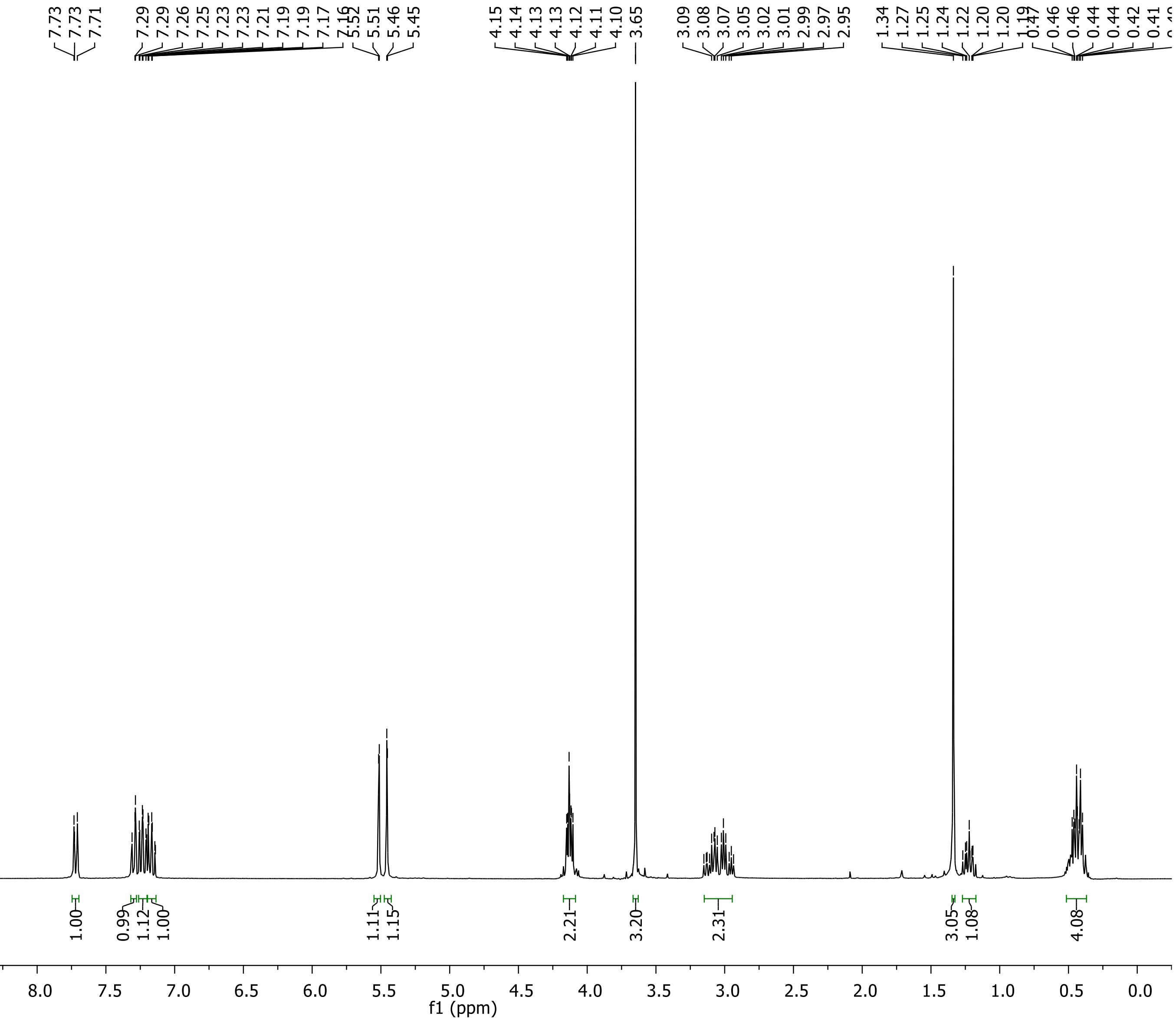

^{13}C NMR ($CDCl_3$, 75.4 MHz)

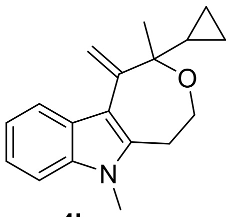

4b

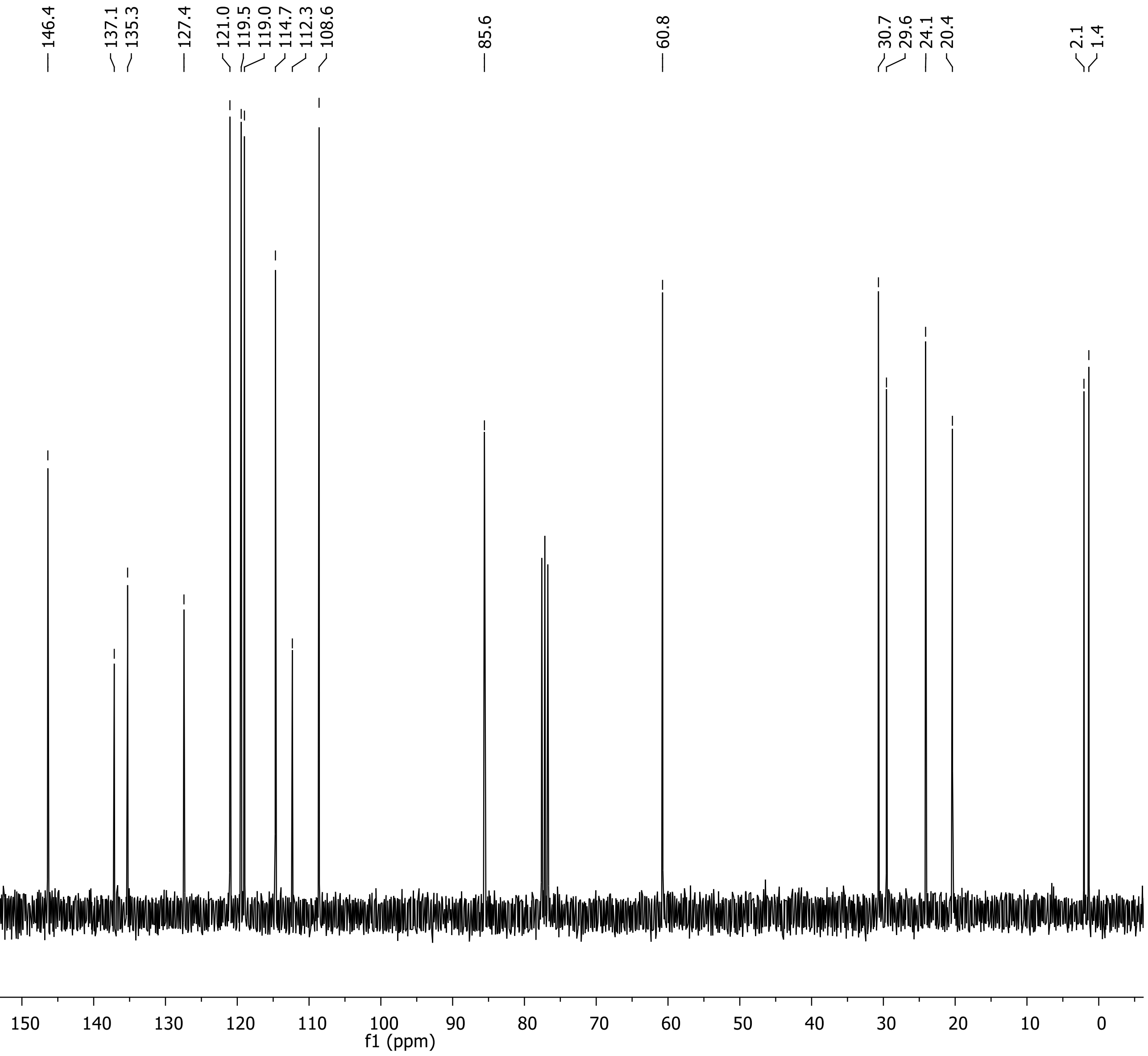

DEPT ($CDCl_3$, 75.4 MHz)

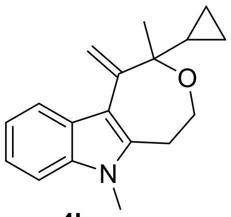

4b

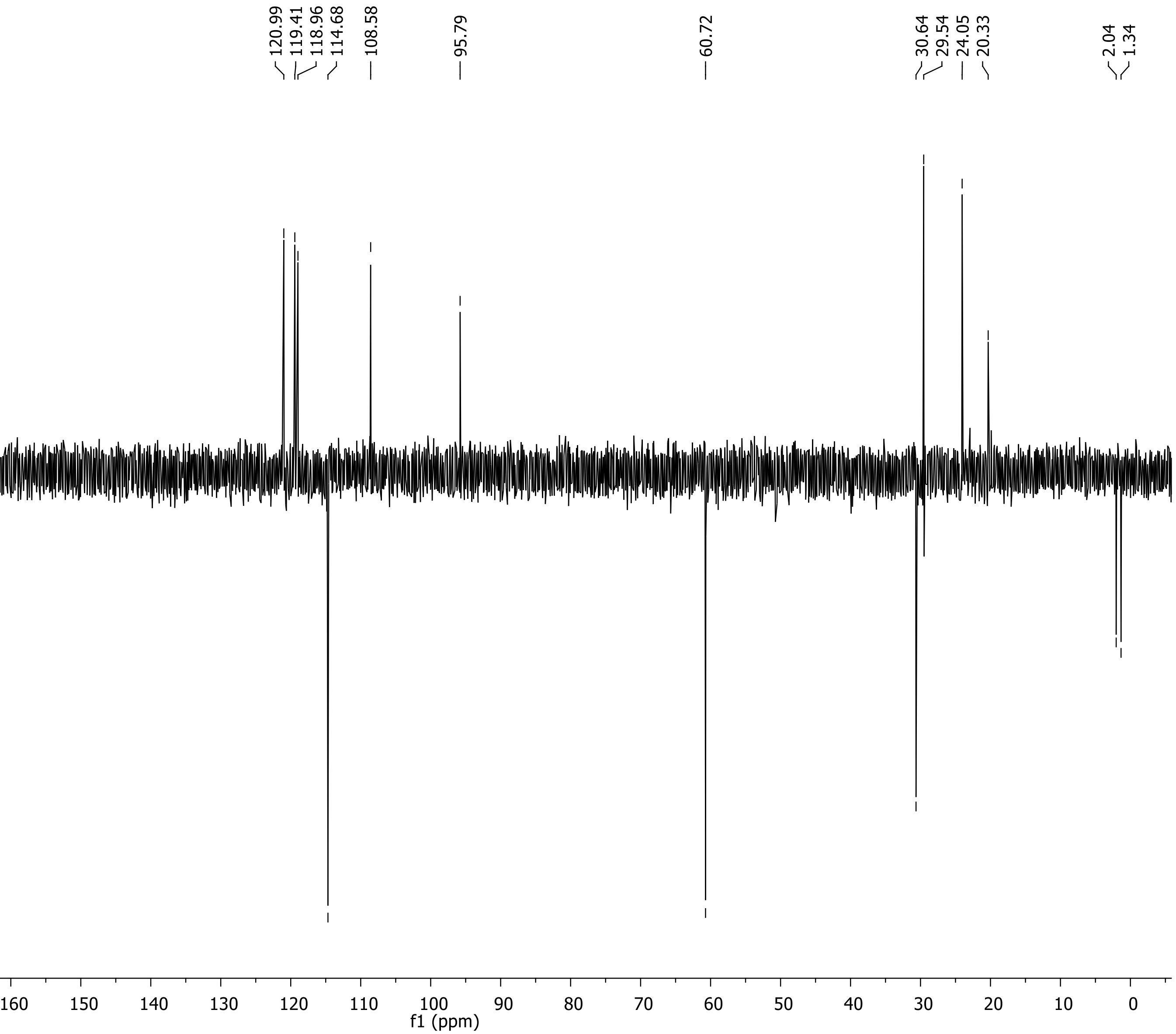

^1H NMR ($CDCl_3$, 300 MHz)

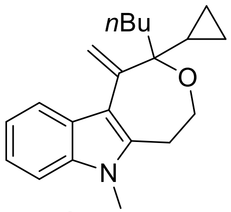

4c

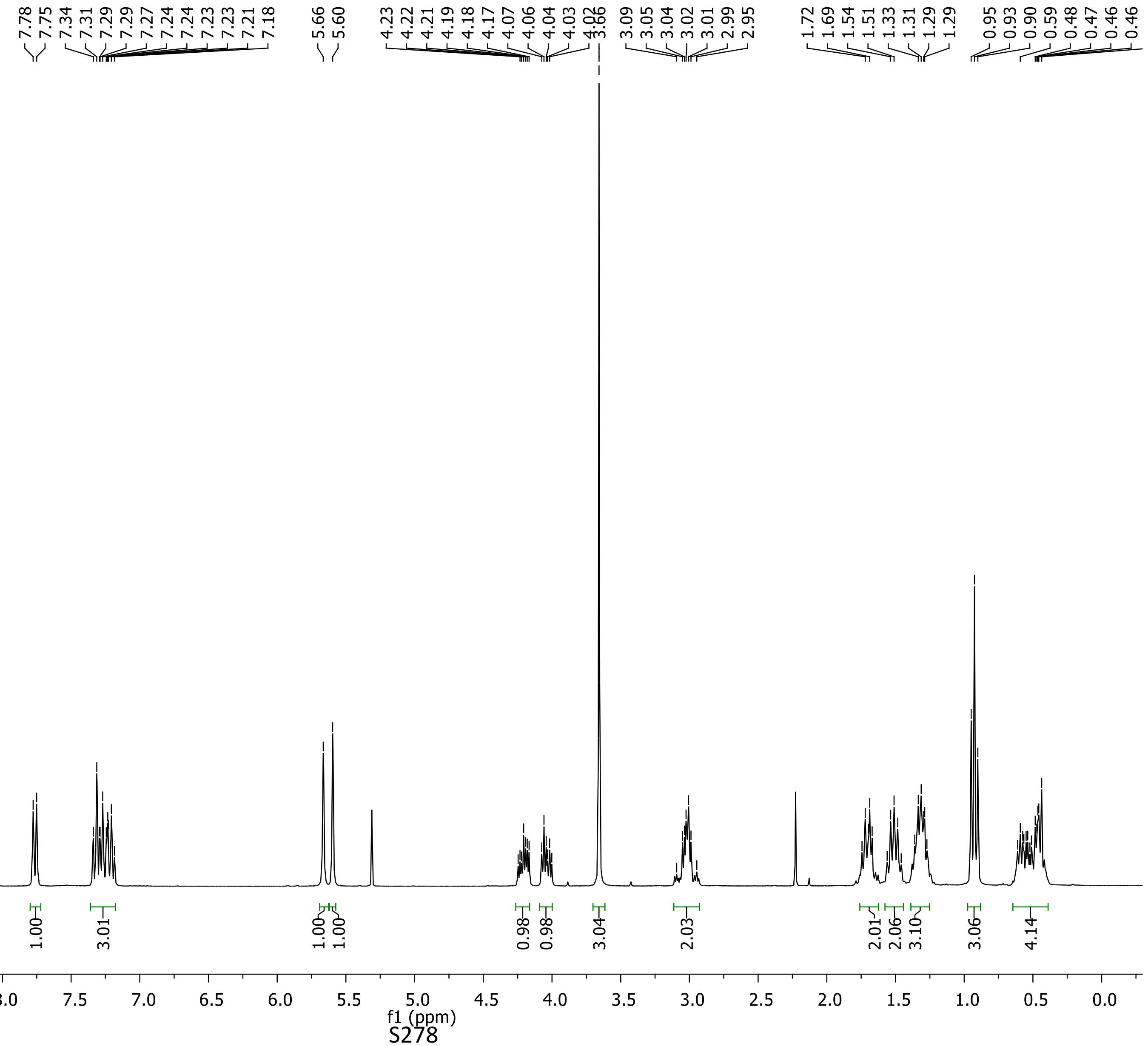

^{13}C NMR ($CDCl_3$, 75.4 MHz)

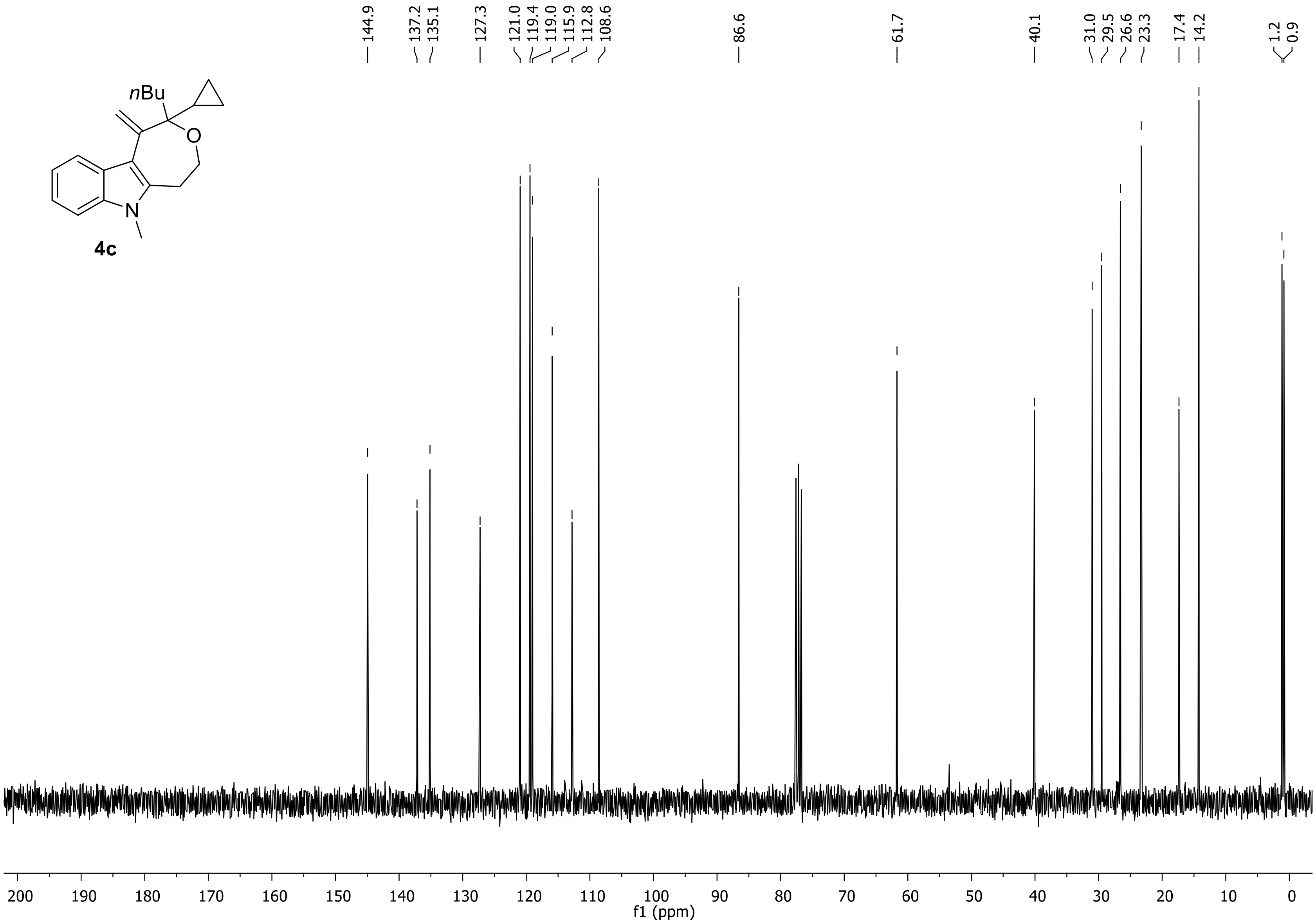

DEPT ($CDCl_3$, 75.4 MHz)

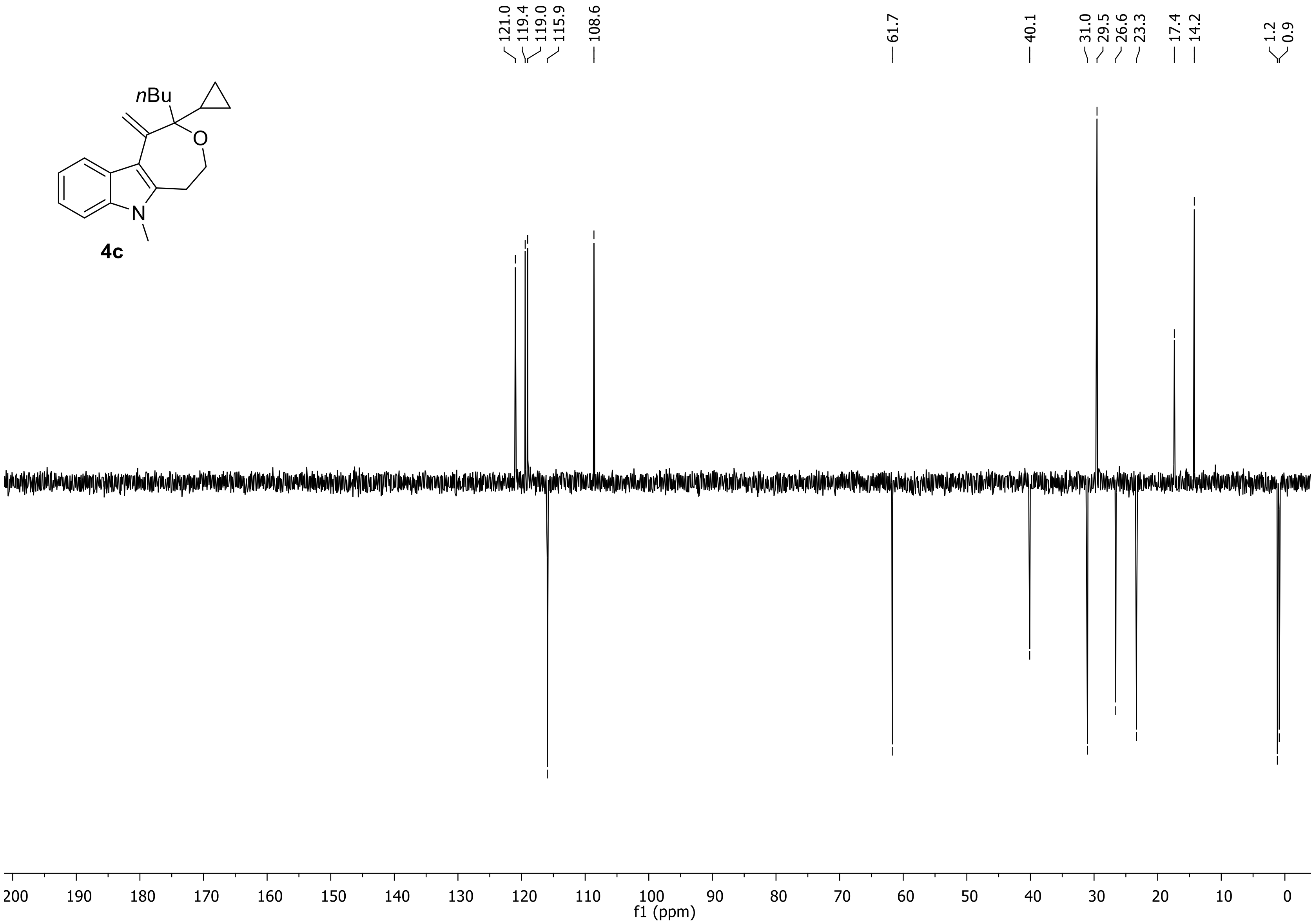

^{1}H NMR ($CDCl_3$, 500 MHz)

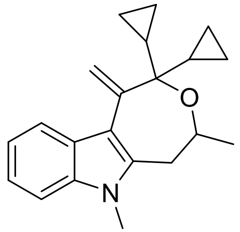
4d

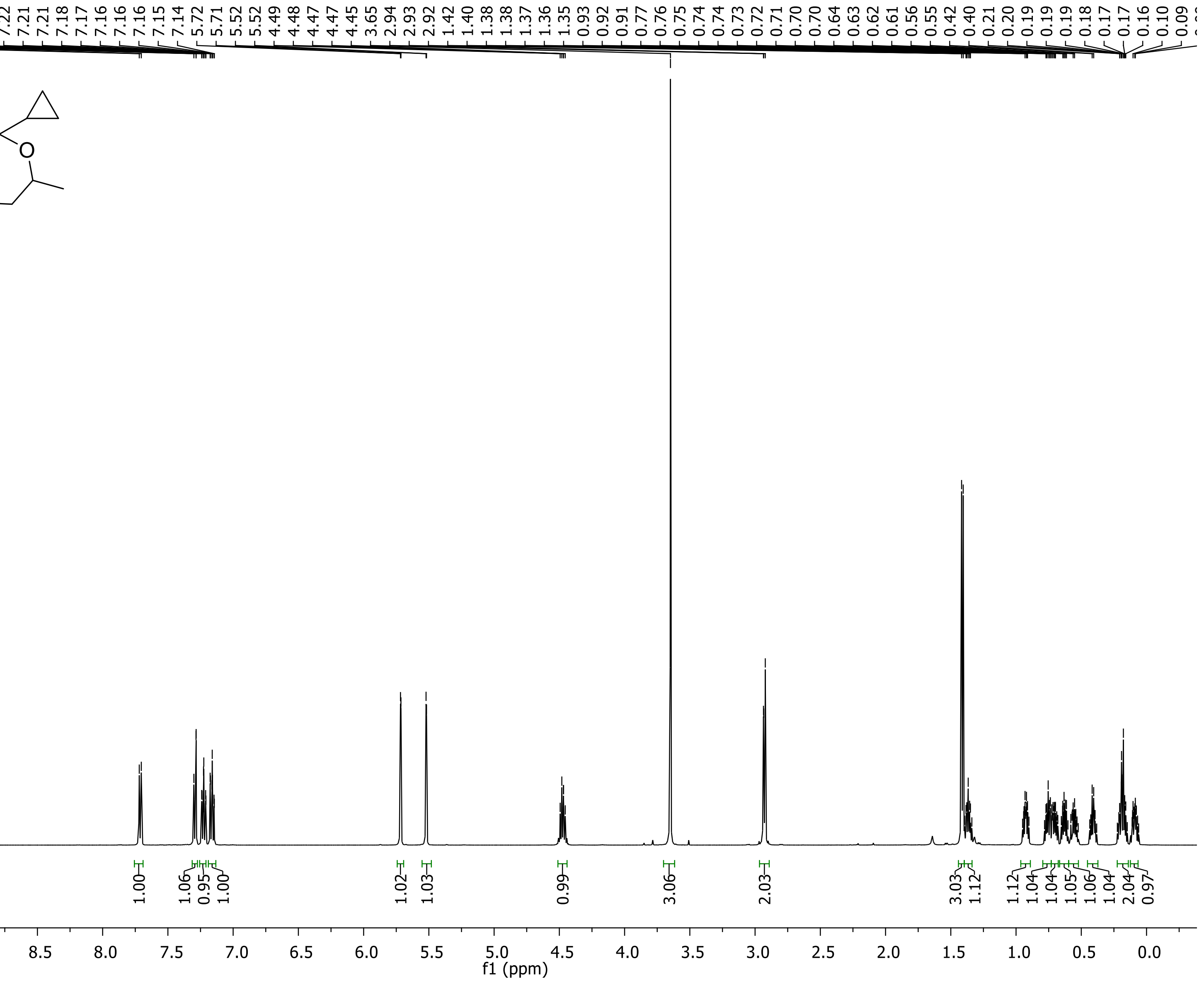

^{13}C NMR ($CDCl_3$, 125.7 MHz)

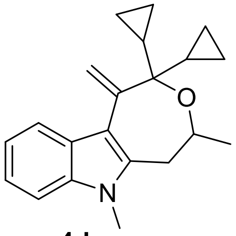

4d

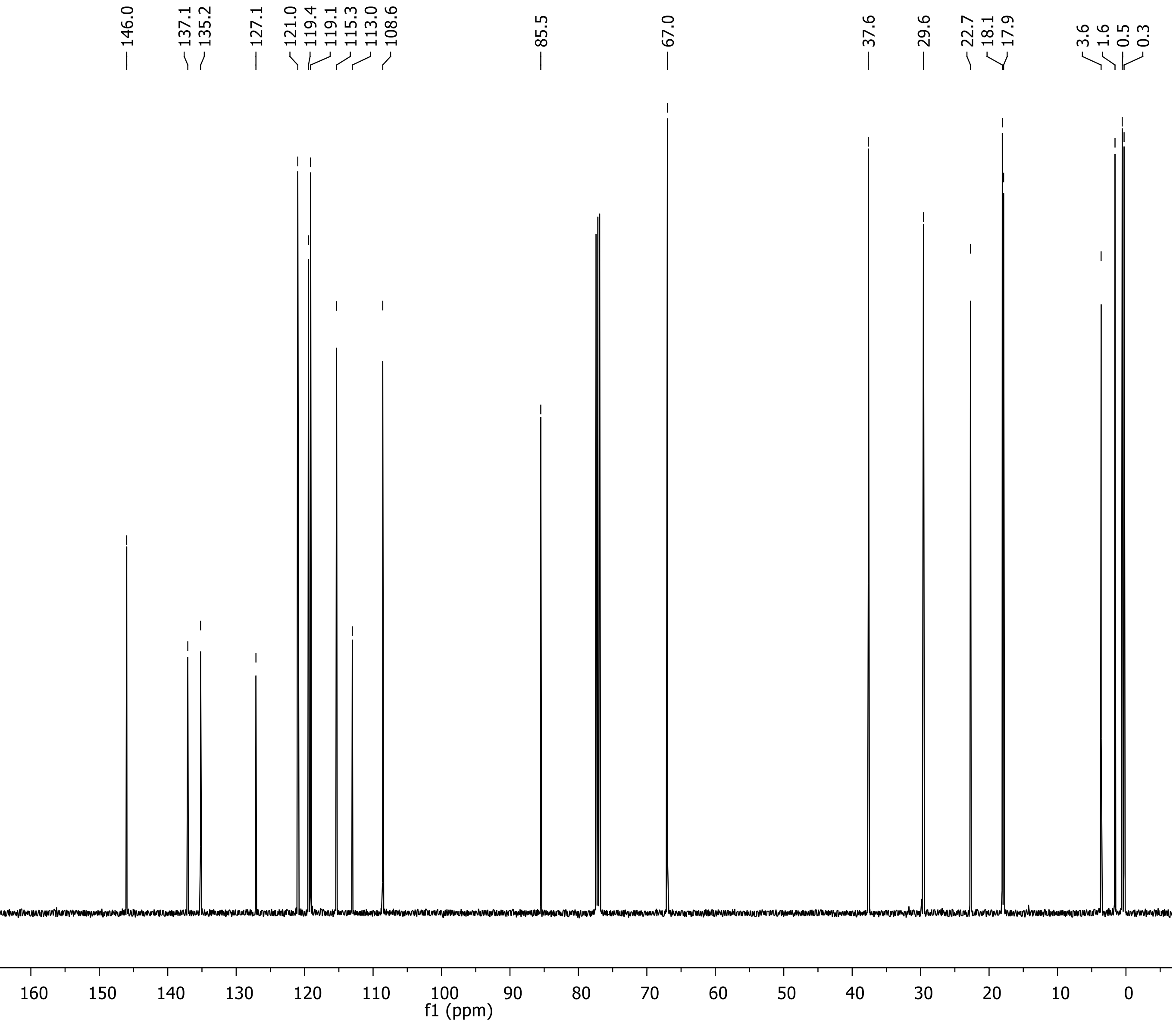

DEPT (CDCl$_3$, 125.7 MHz)

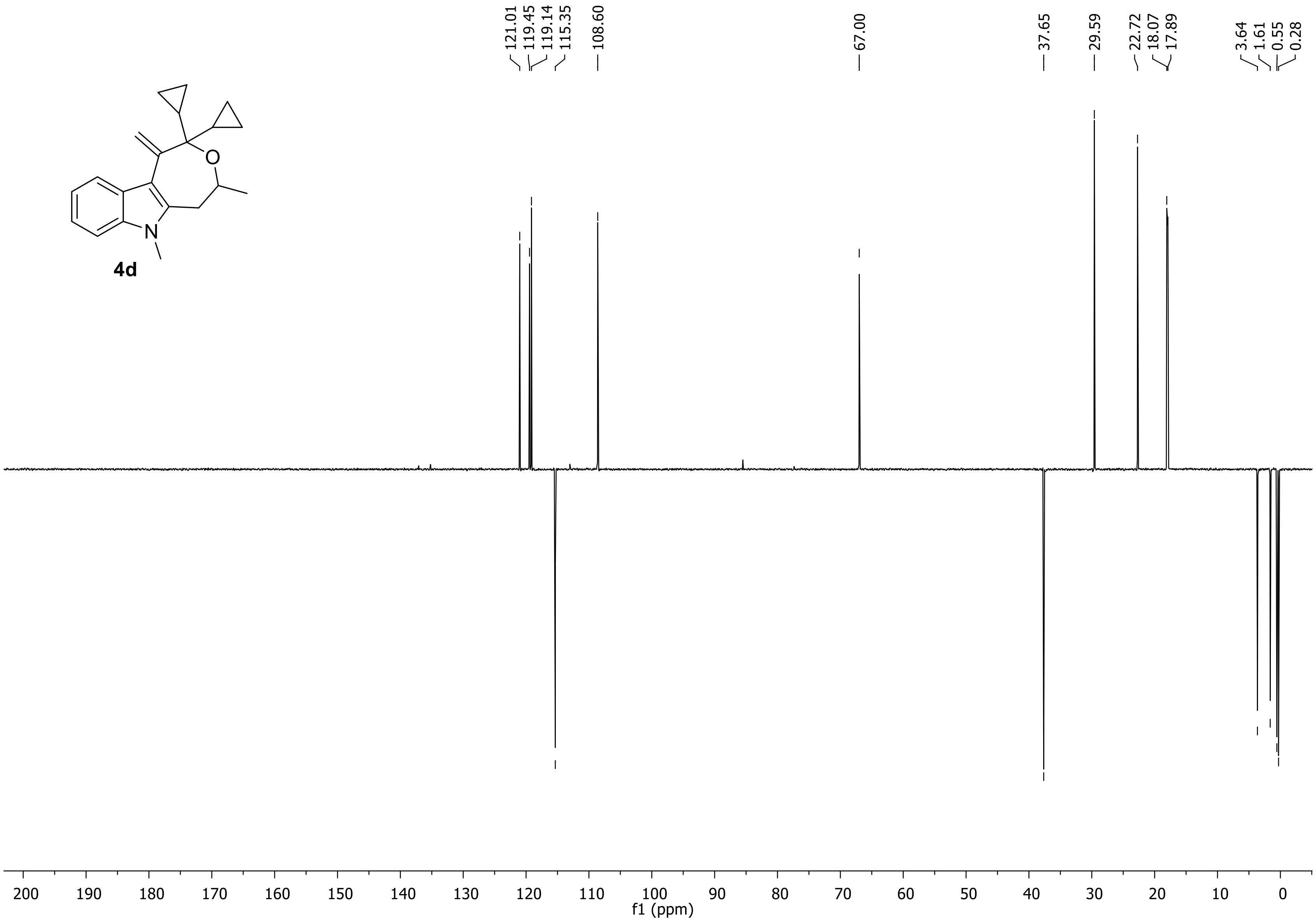

4d

^{1}H NMR ($CDCl_3$, 300 MHz)

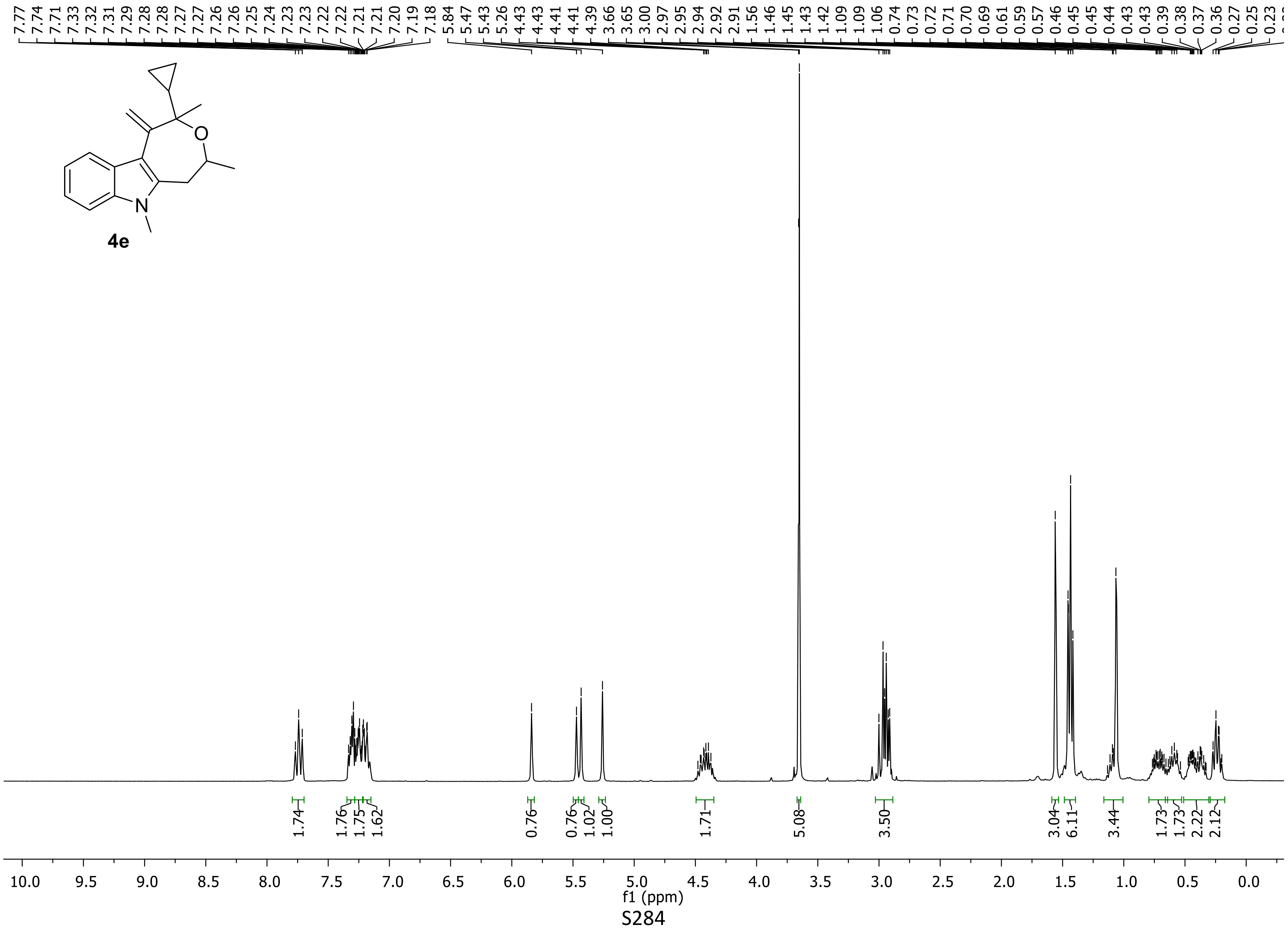

^{13}C NMR ($CDCl_3$, 75.4 MHz)

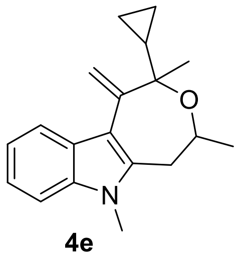

4e

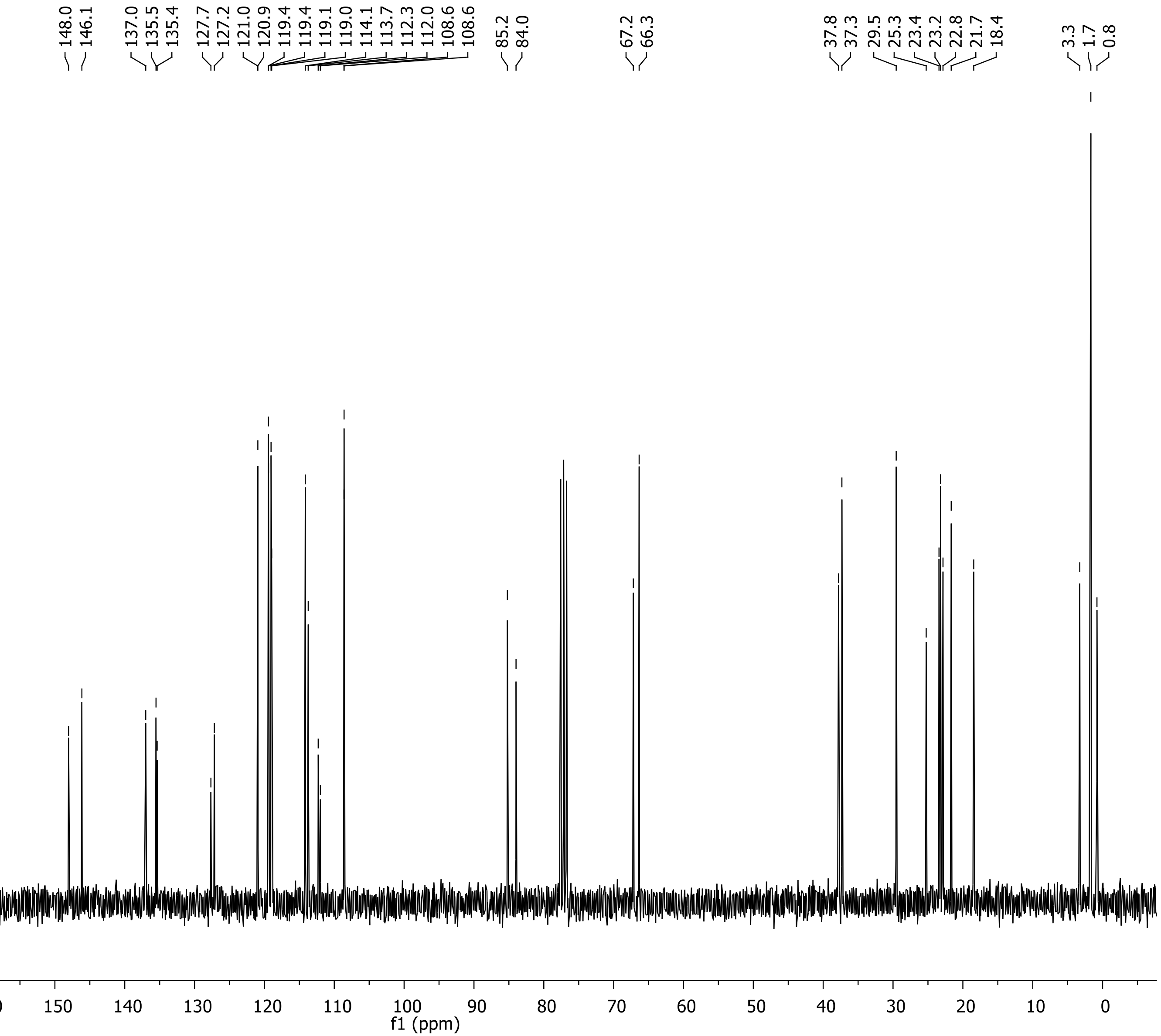

DEPT ($CDCl_3$, 75.4 MHz)

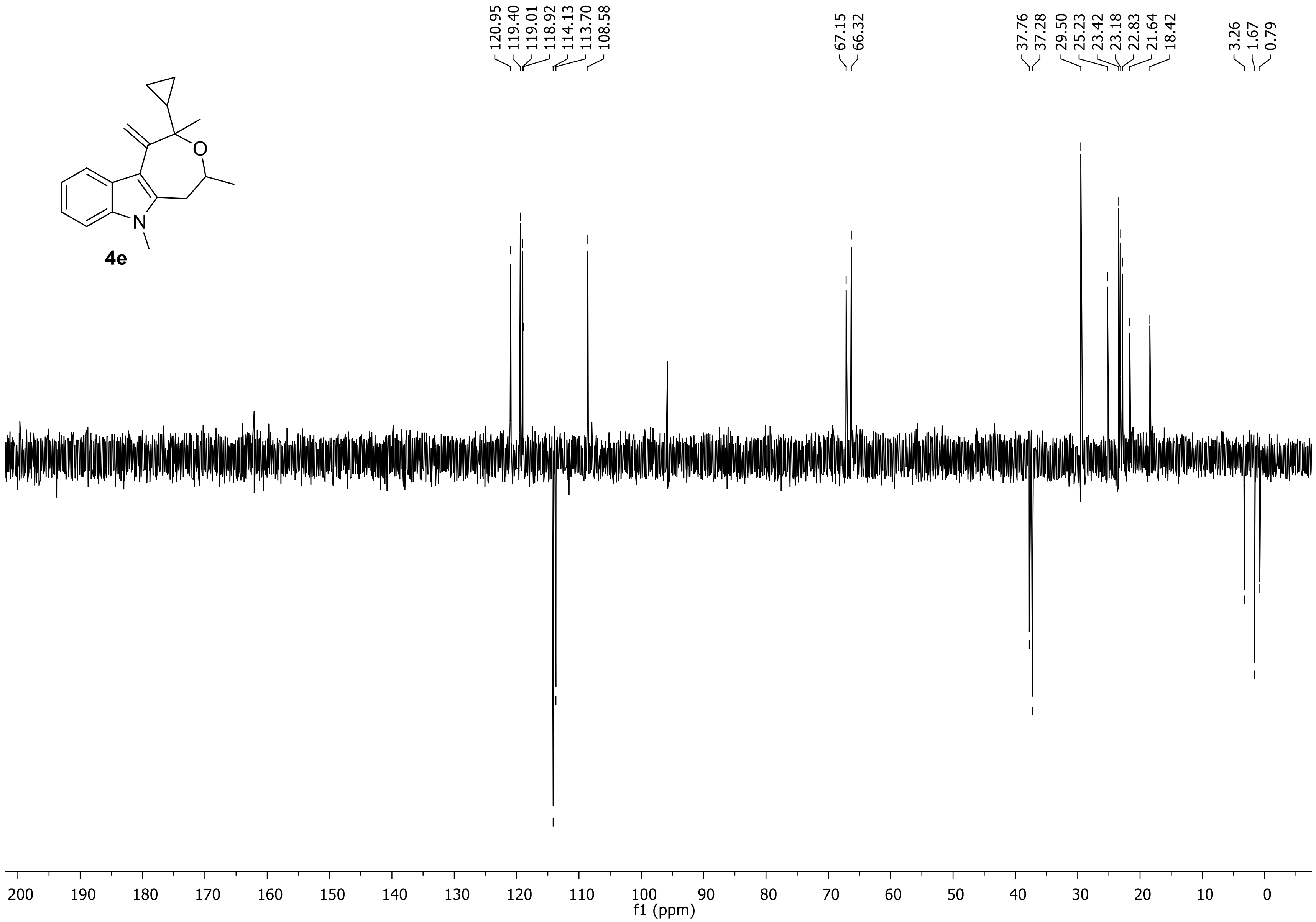

^{1}H NMR ($CDCl_3$, 300 MHz)

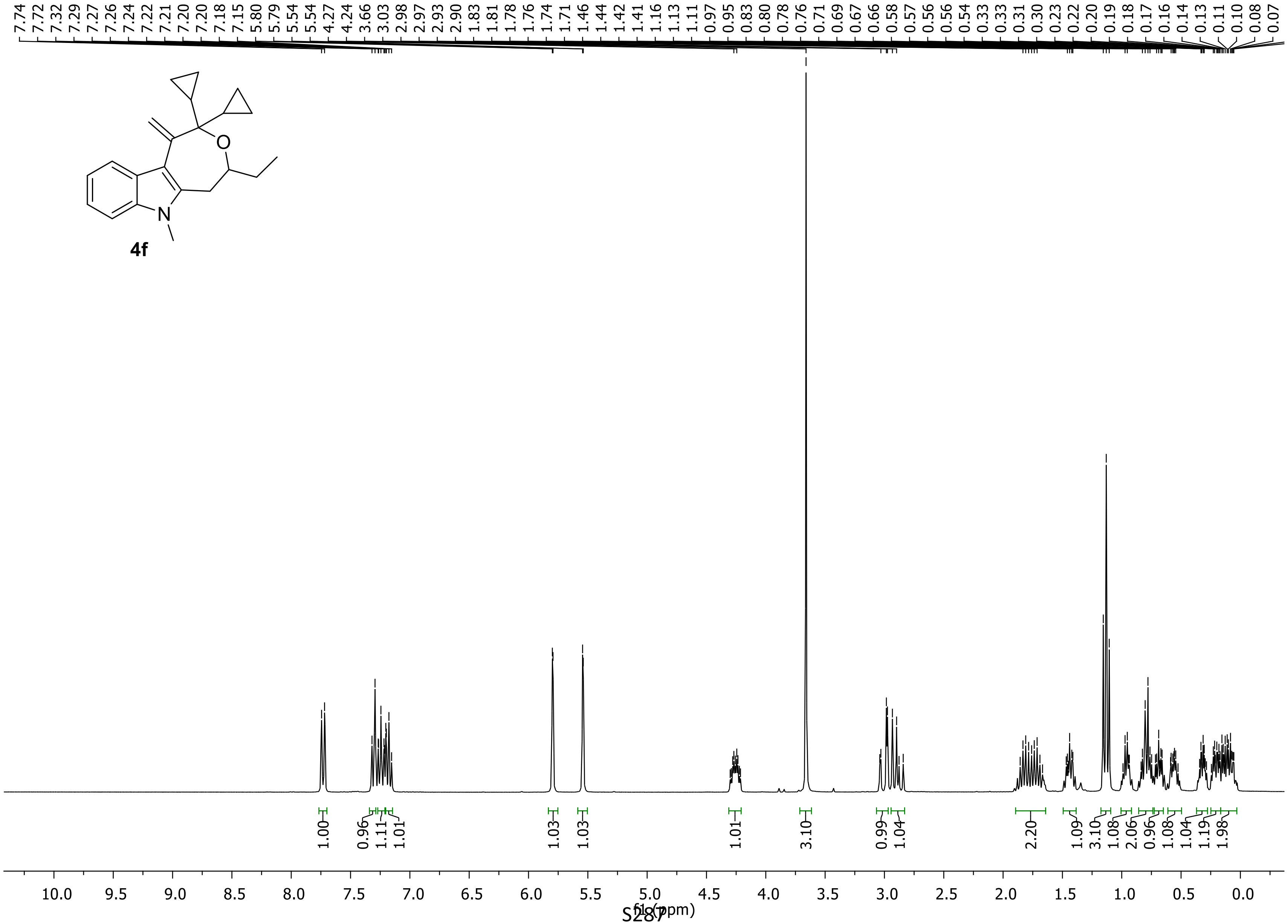

^{13}C NMR ($CDCl_3$, 75.4 MHz)

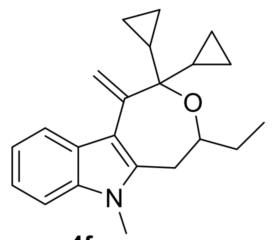

4f

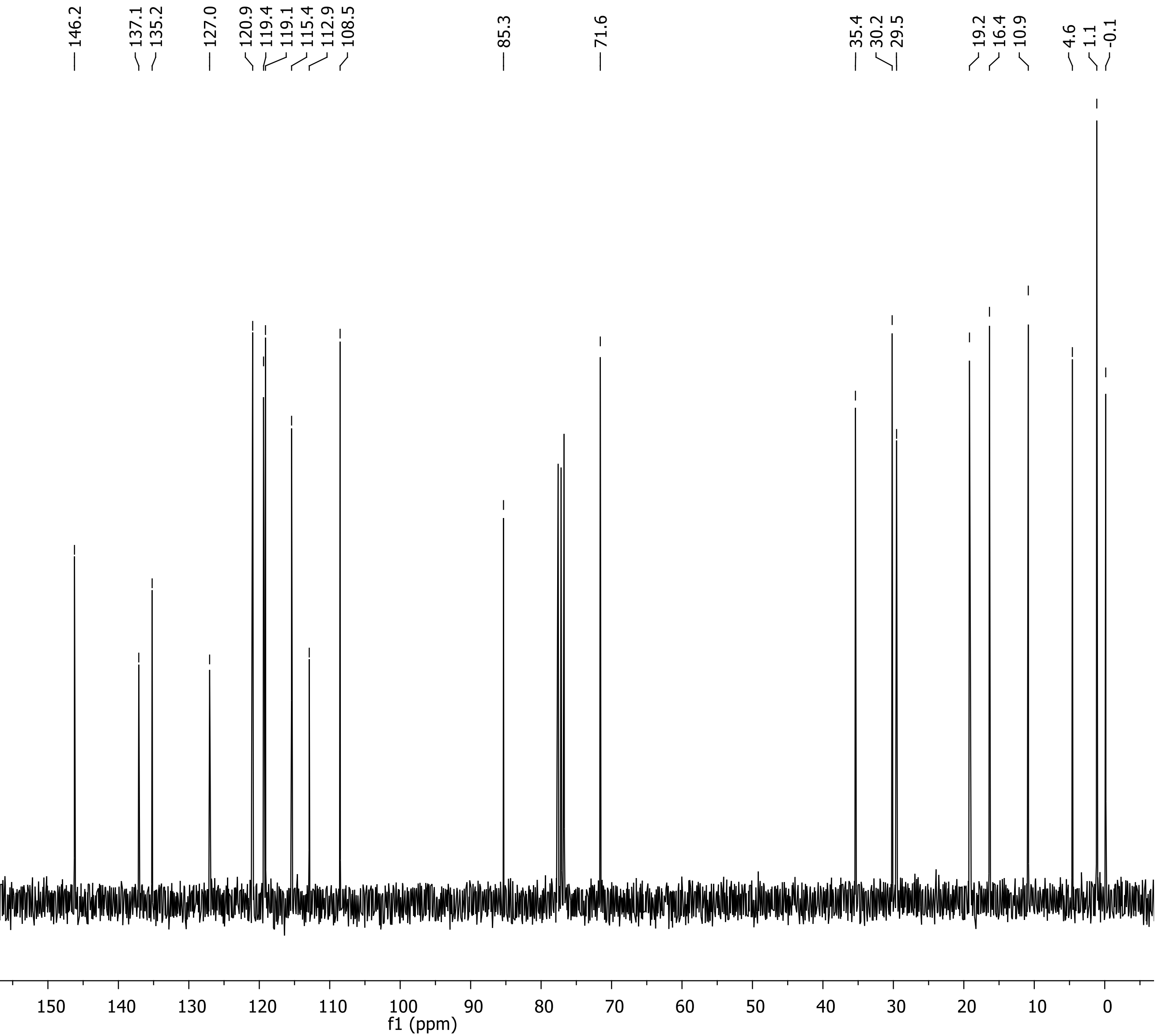

DEPT ($CDCl_3$, 75.4 MHz)

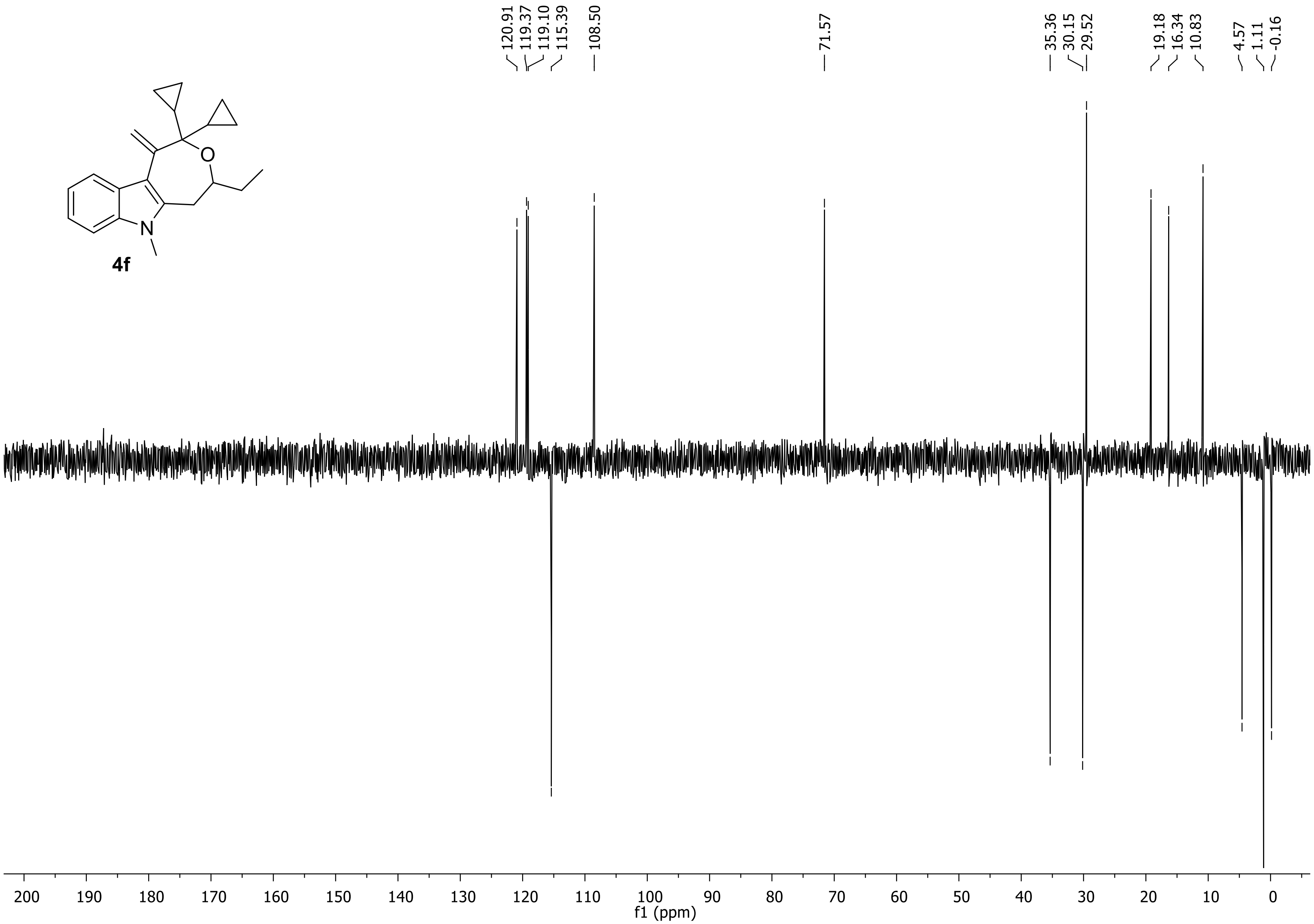

^{1}H NMR ($CDCl_3$, 300 MHz)

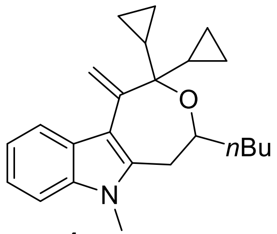

4g

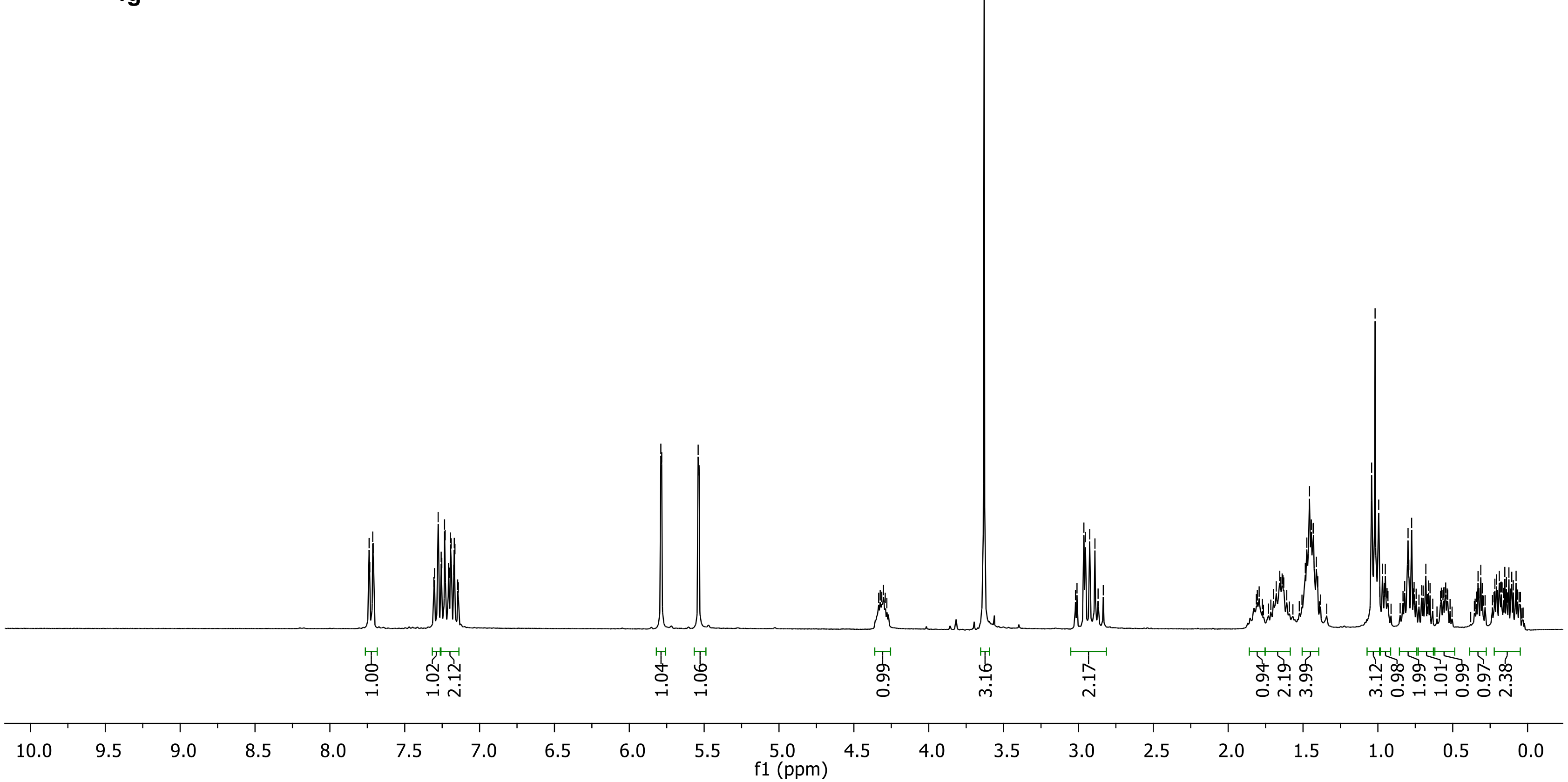

^{13}C NMR ($CDCl_3$, 75.4 MHz)

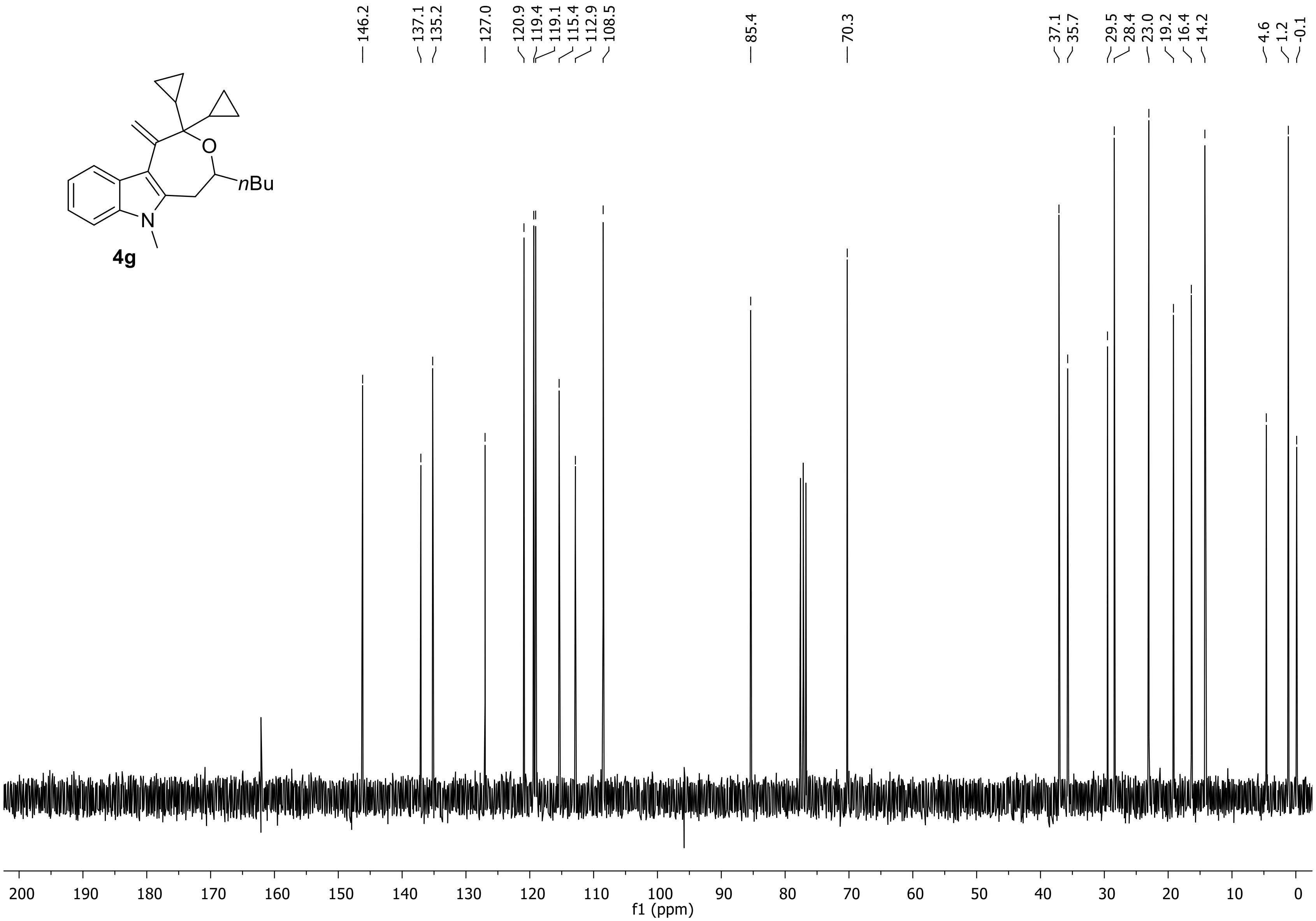

DEPT ($CDCl_3$, 75.4 MHz)

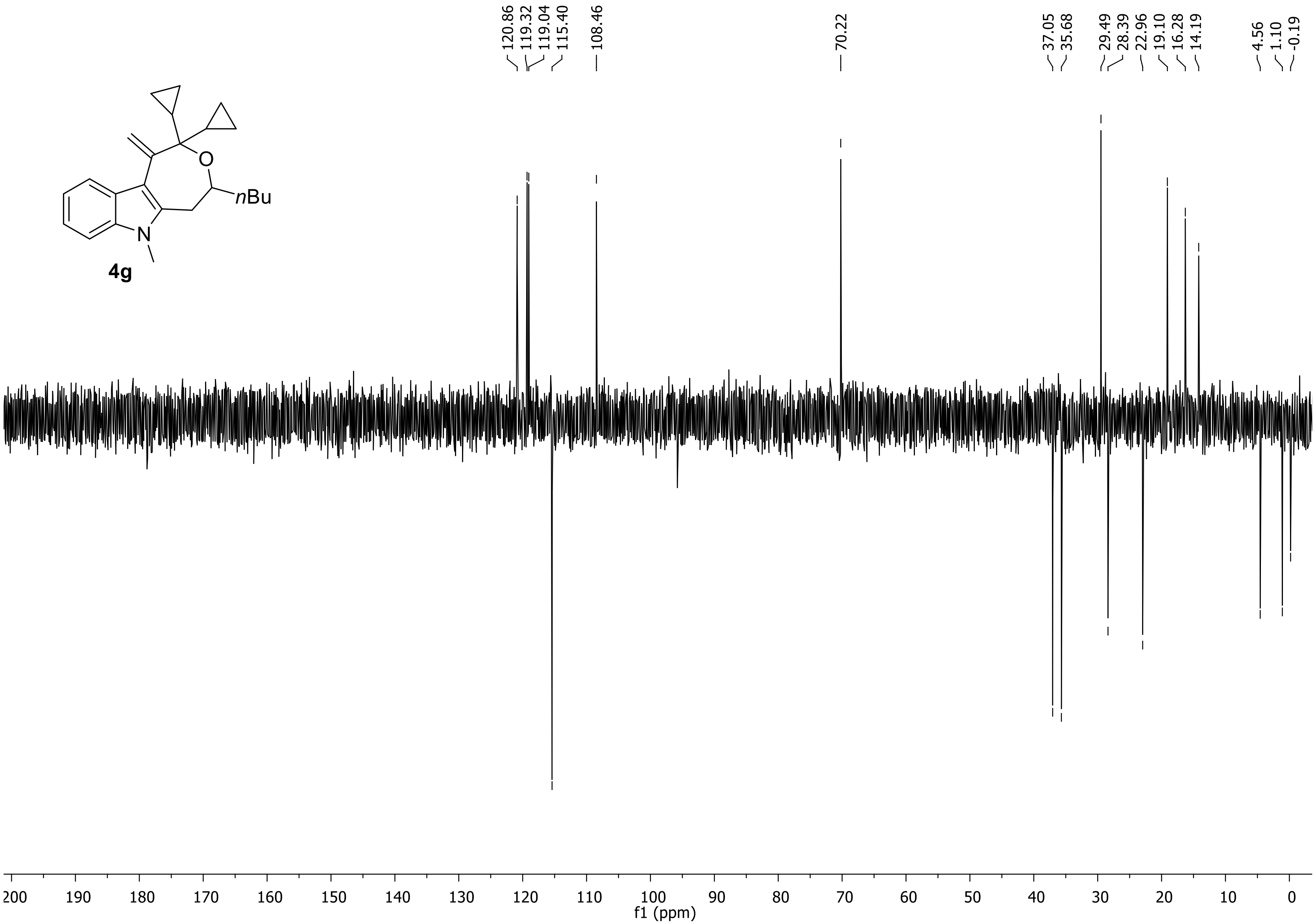

4g

^{1}H NMR ($CDCl_3$, 300 MHz)

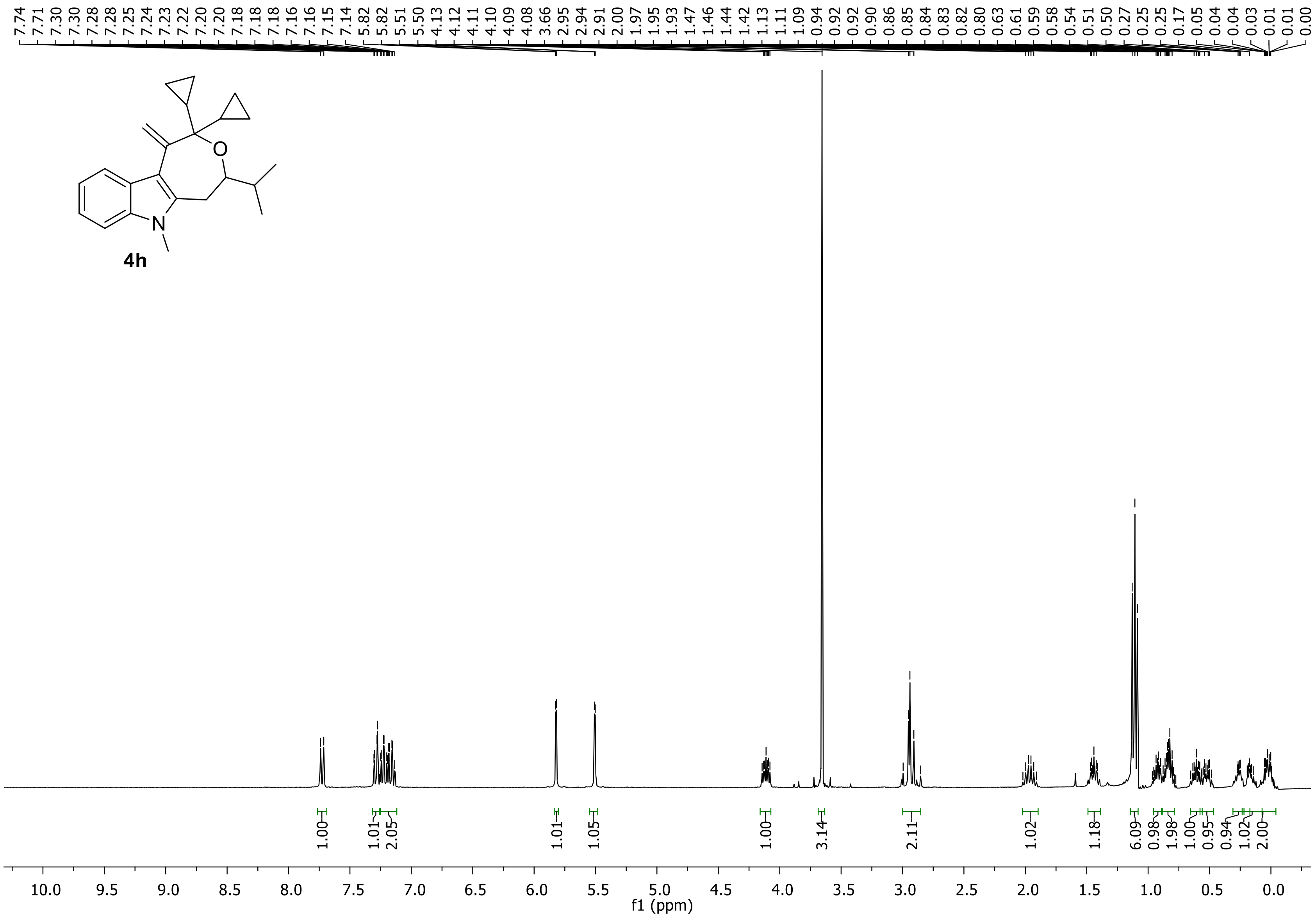

4h

^{13}C NMR ($CDCl_3$, 75.4 MHz)

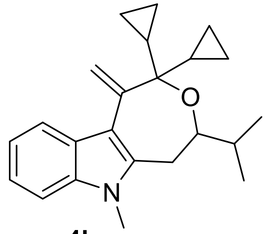

4h

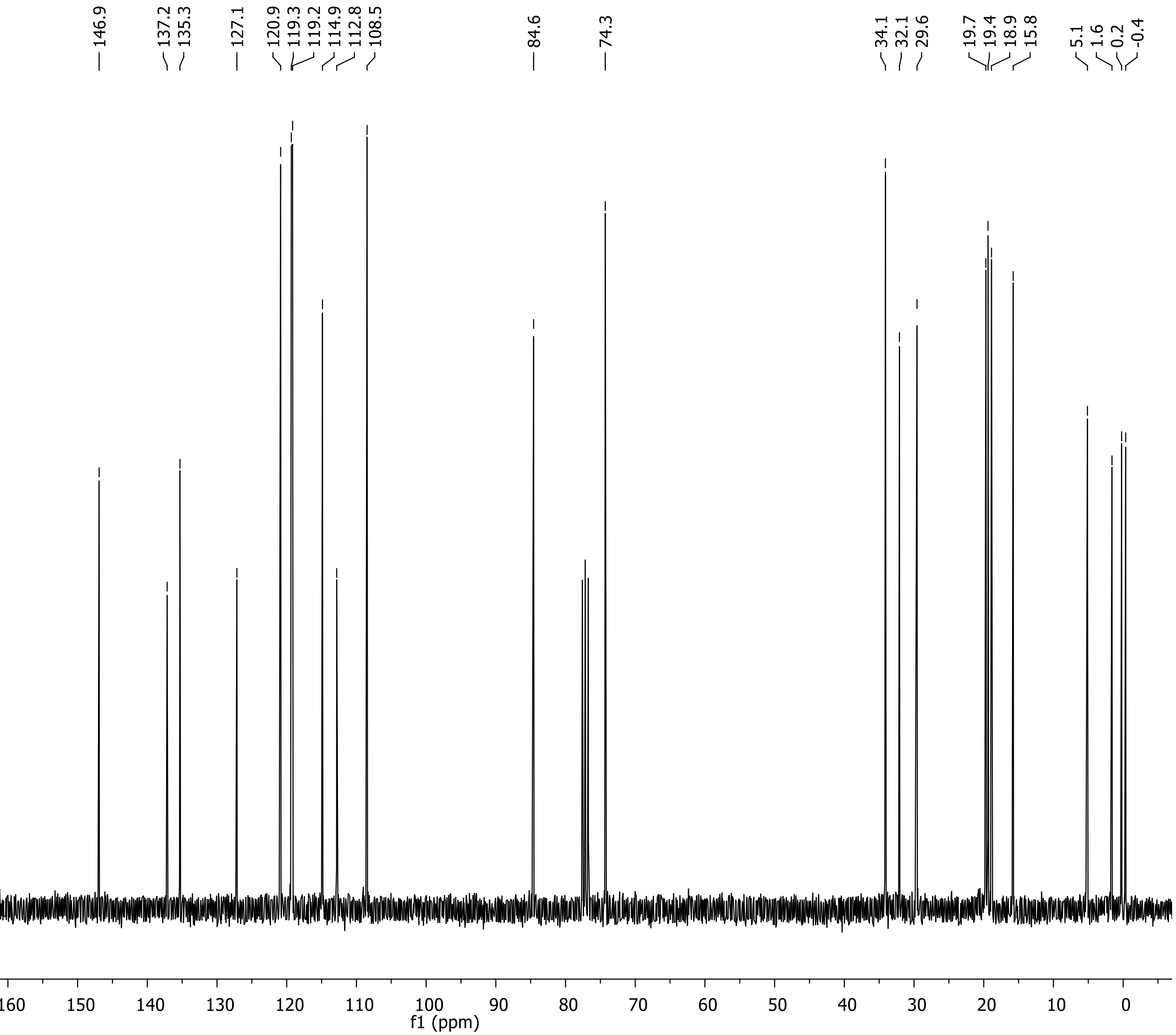

DEPT ($CDCl_3$, 75.4 MHz)

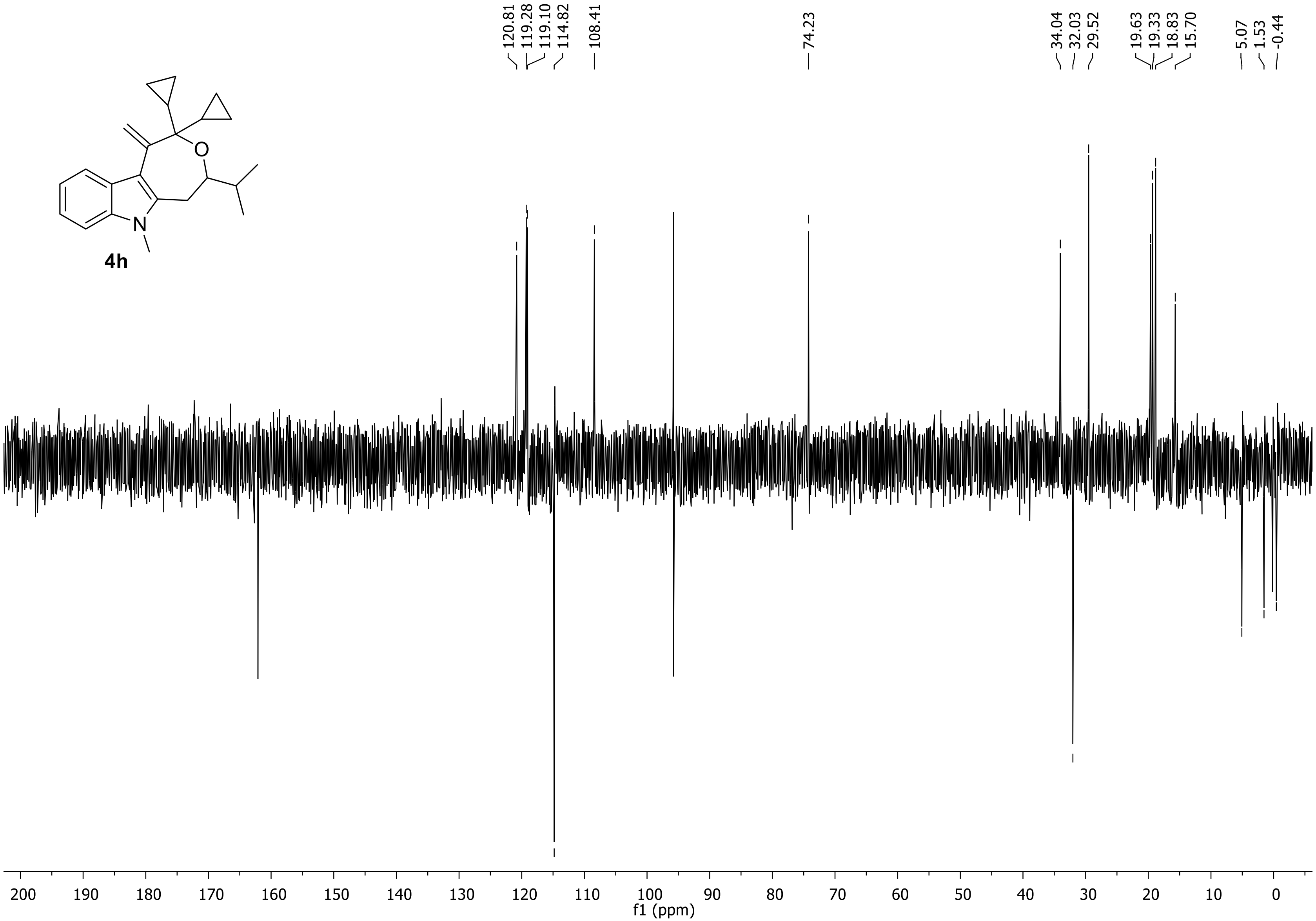

^{1}H NMR ($CDCl_3$, 300 MHz)

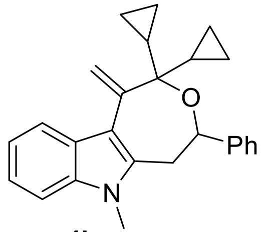

4i

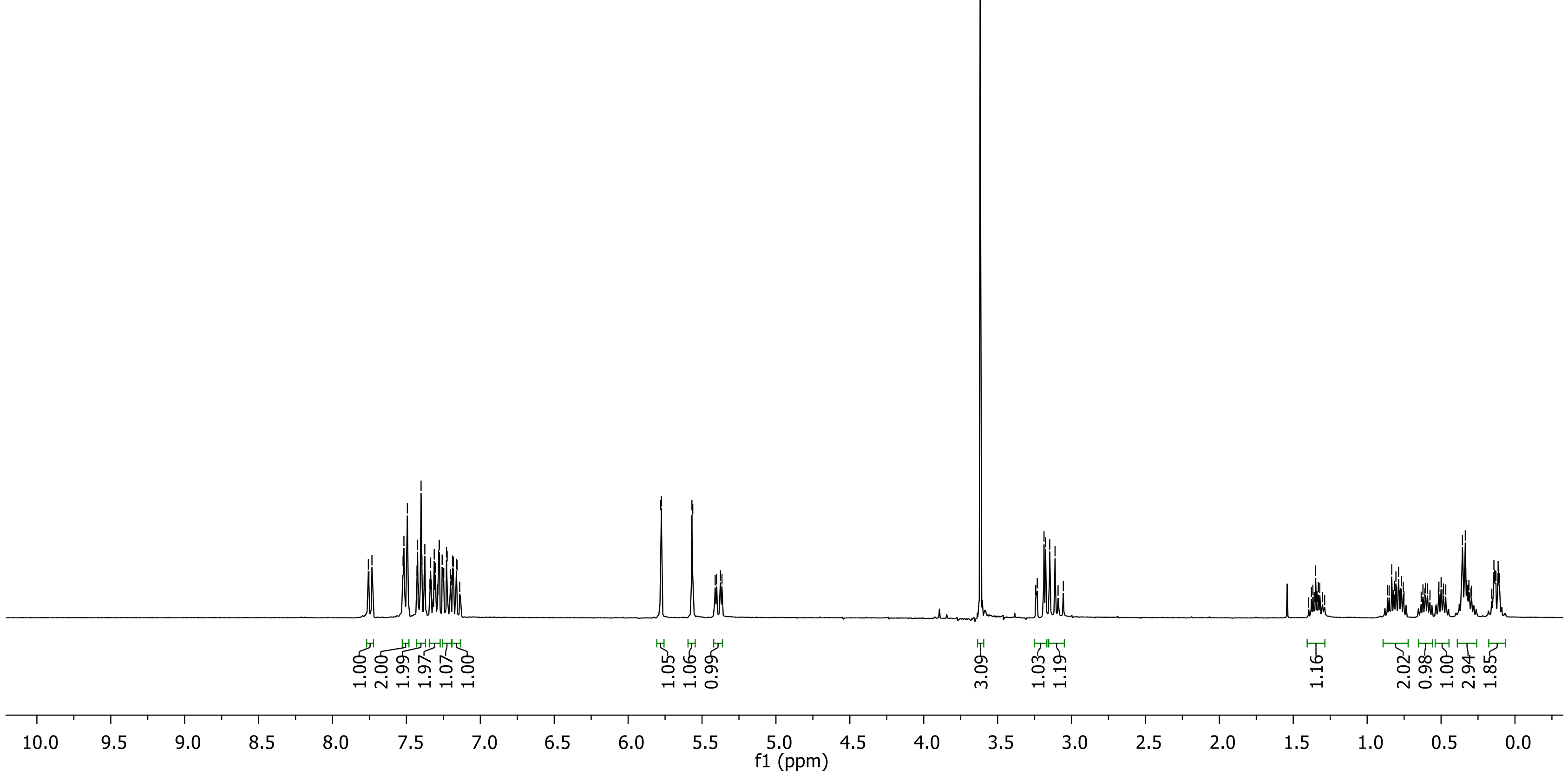

^{13}C NMR ($CDCl_3$, 75.4 MHz)

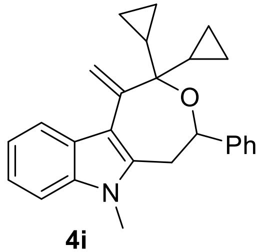

4i

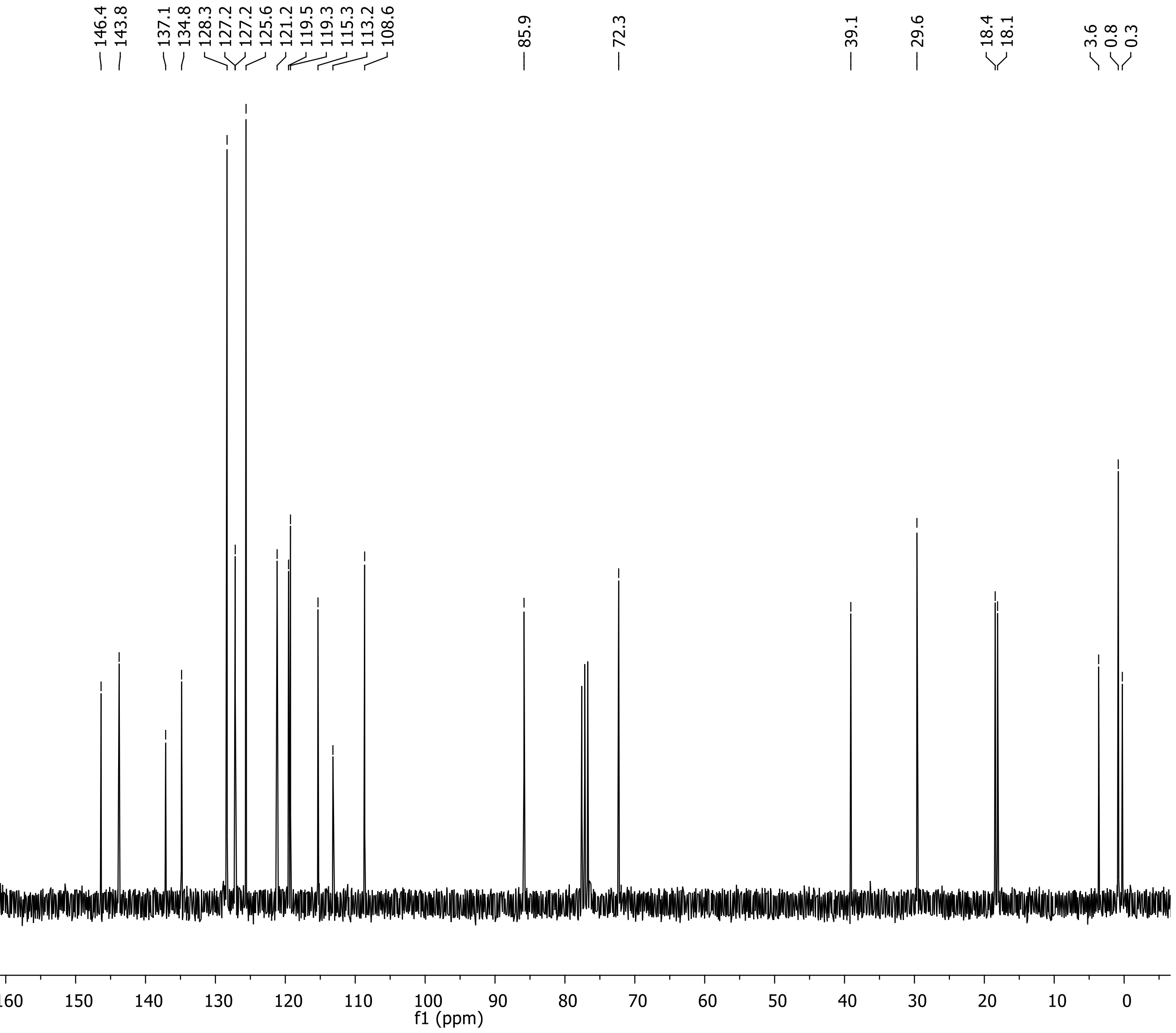

DEPT ($CDCl_3$, 75.4 MHz)

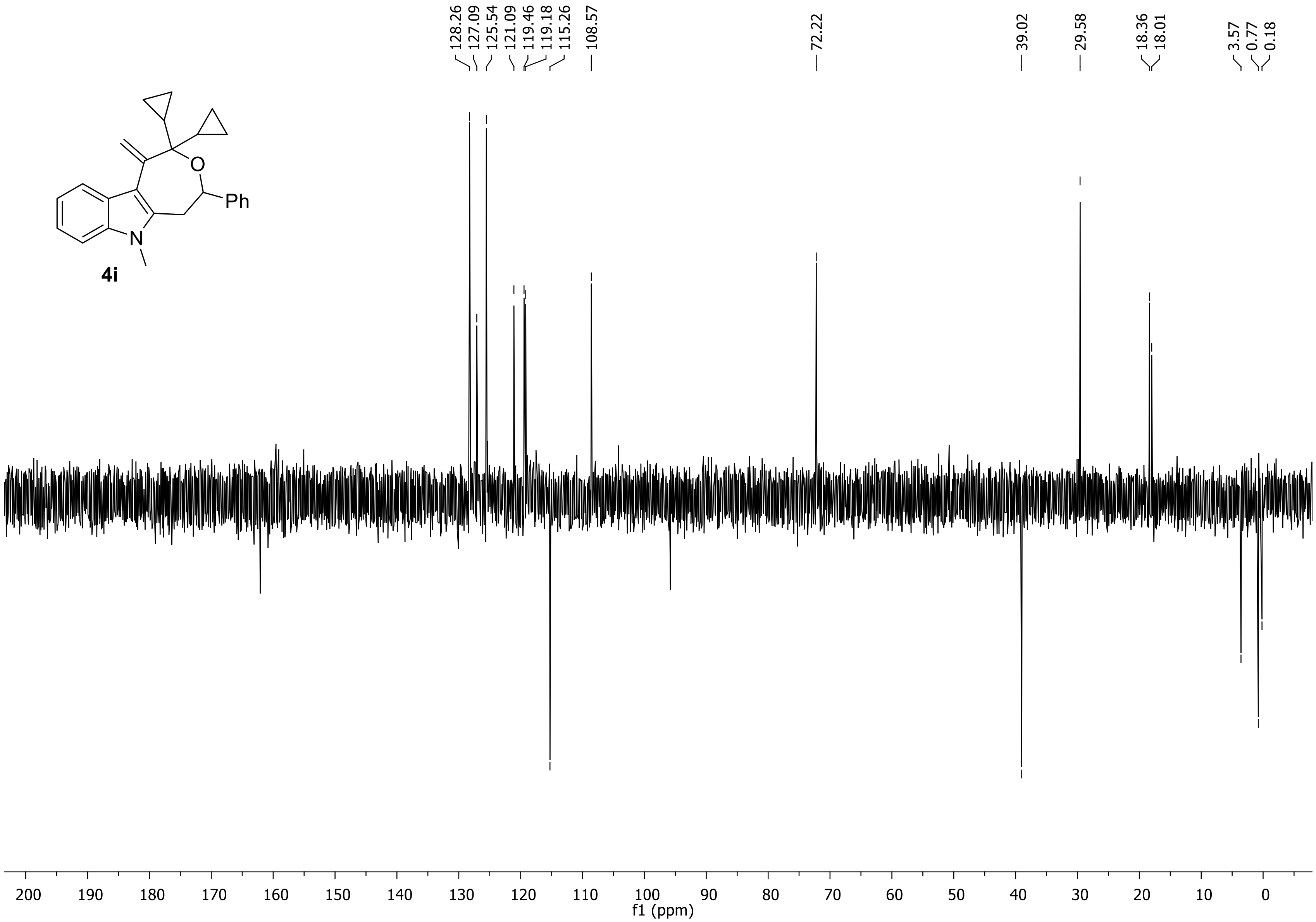

^{1}H NMR ($CDCl_3$, 300 MHz)

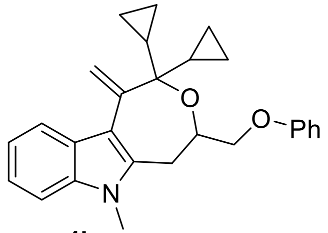

4j

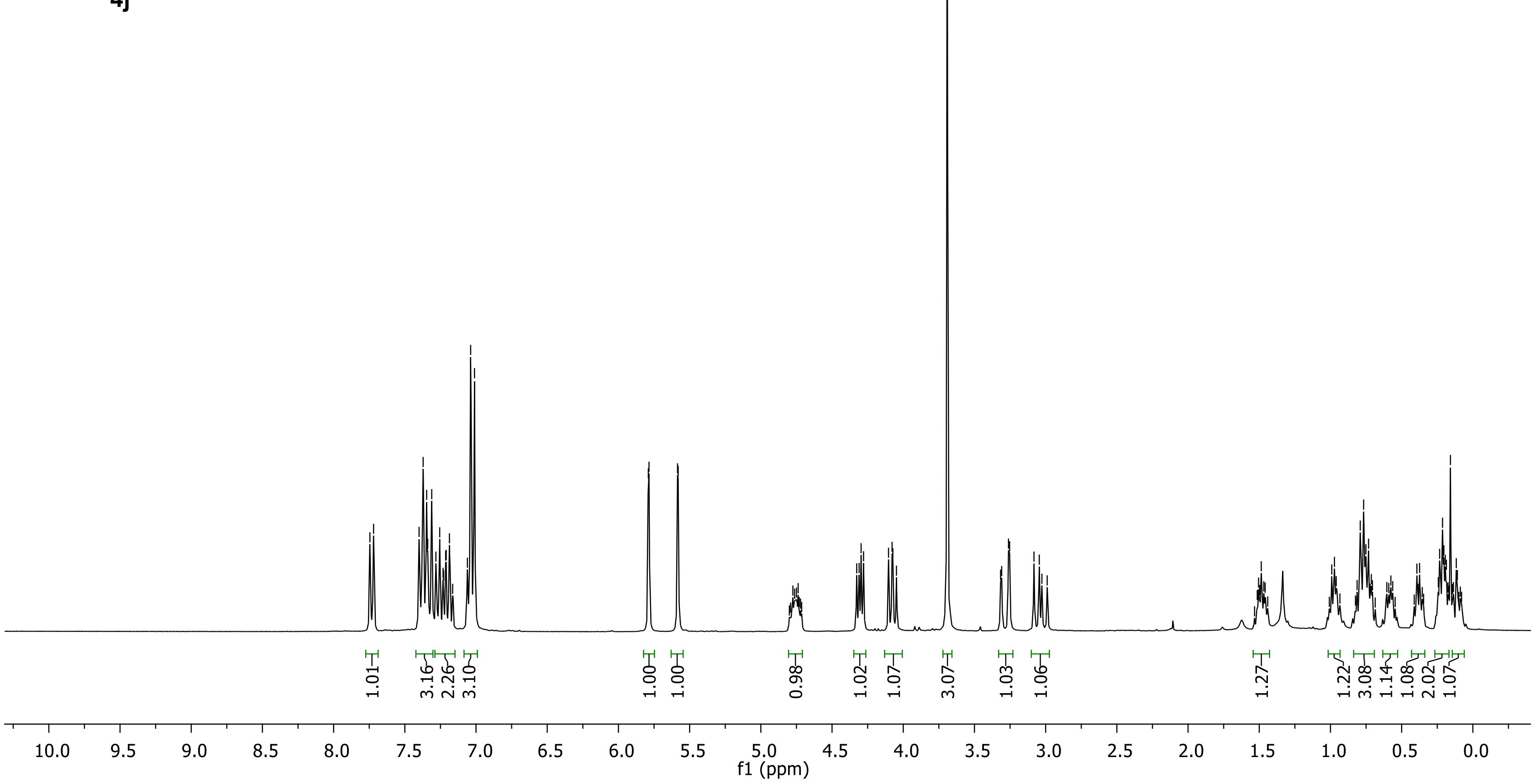

^{13}C NMR ($CDCl_3$, 75.4 MHz)

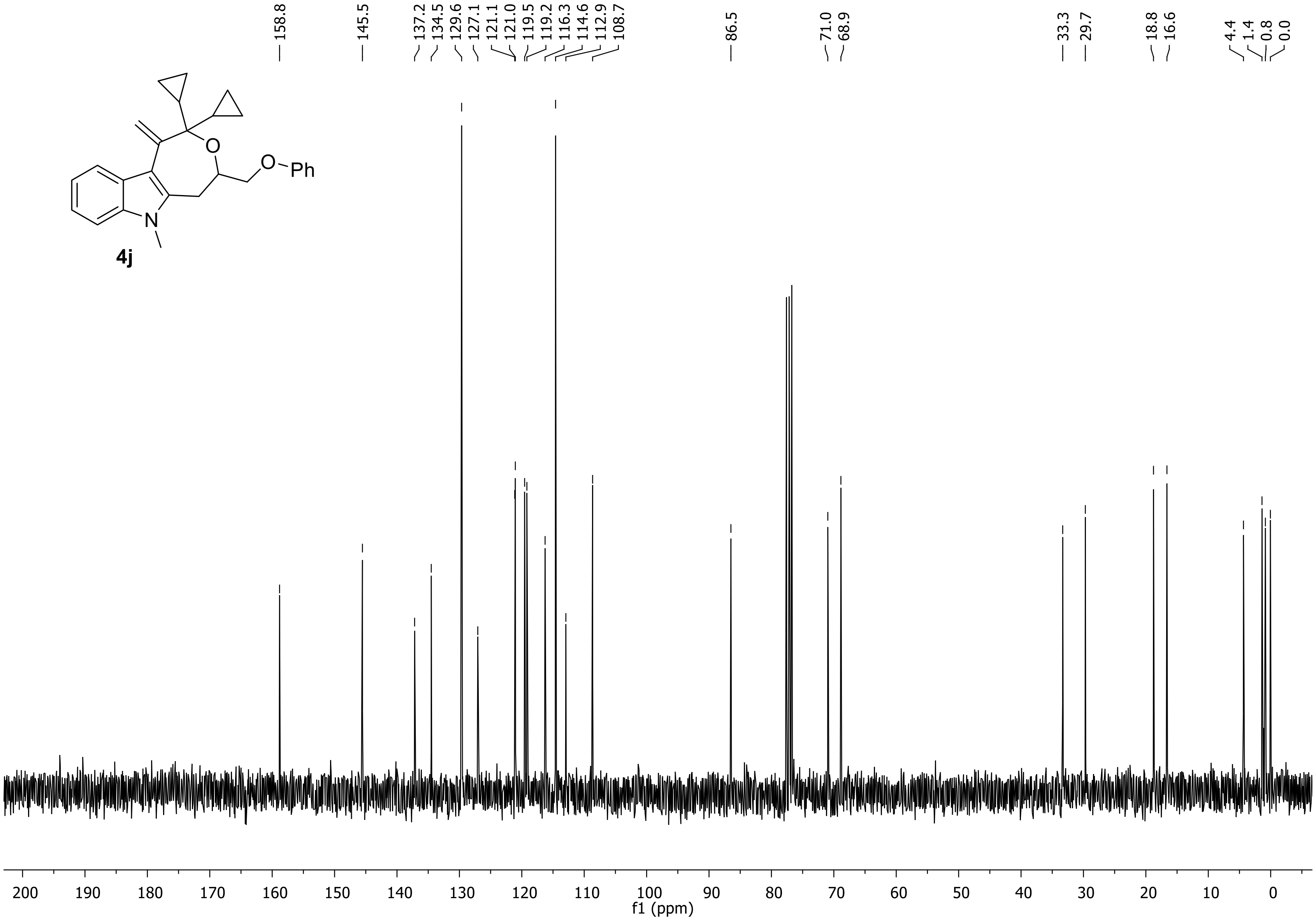

DEPT ($CDCl_3$, 75.4 MHz)

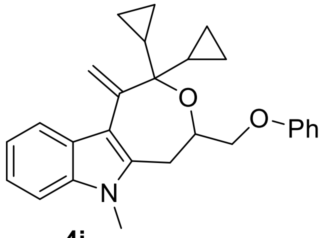

4j

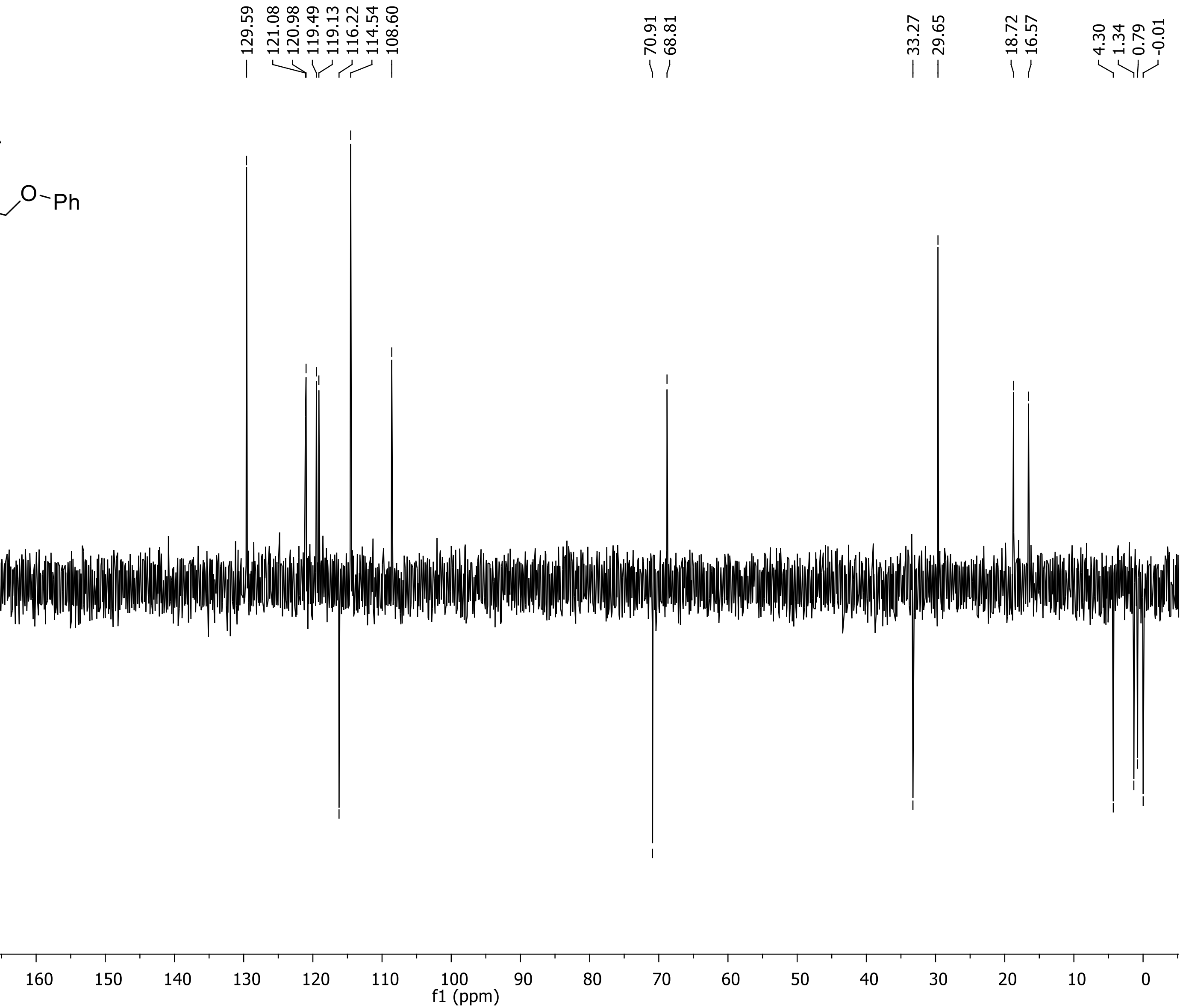

^{1}H NMR ($CDCl_3$, 300 MHz)

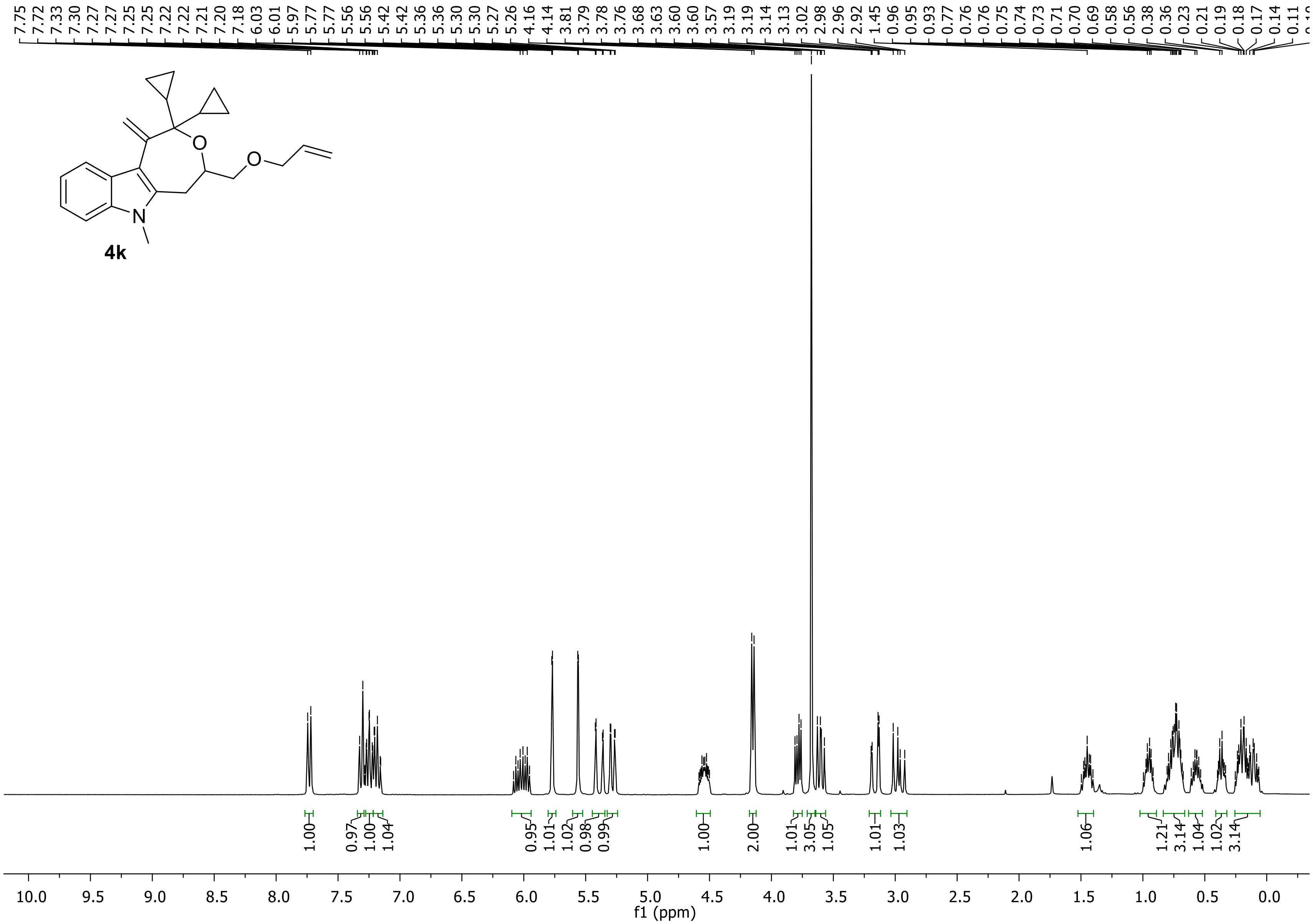

^{13}C NMR ($CDCl_3$, 75.4 MHz)

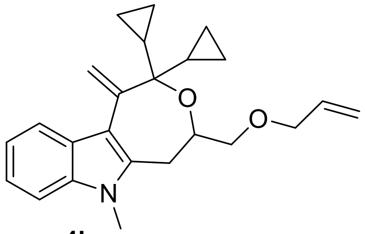

4k

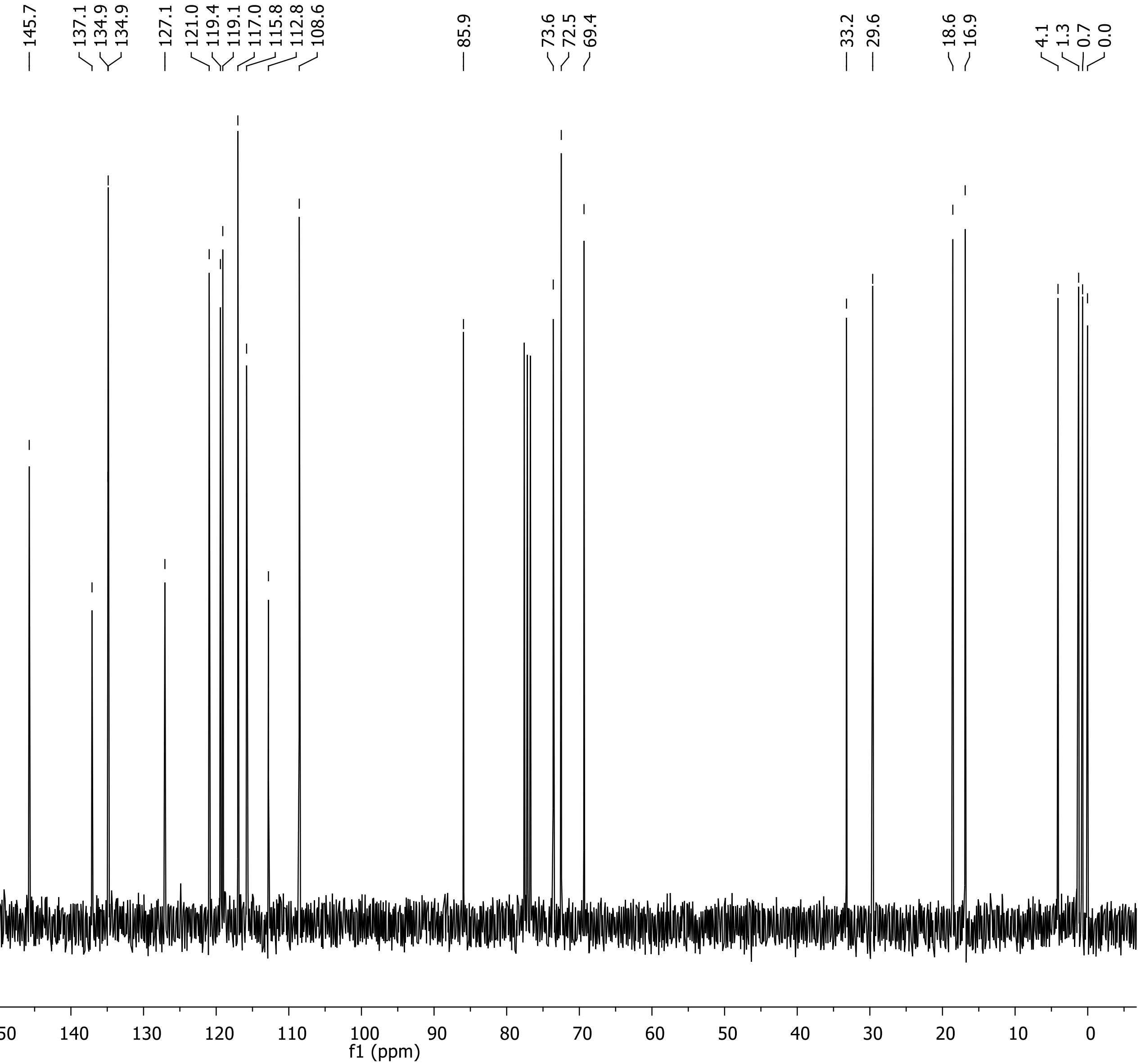

DEPT ($CDCl_3$, 75.4 MHz)

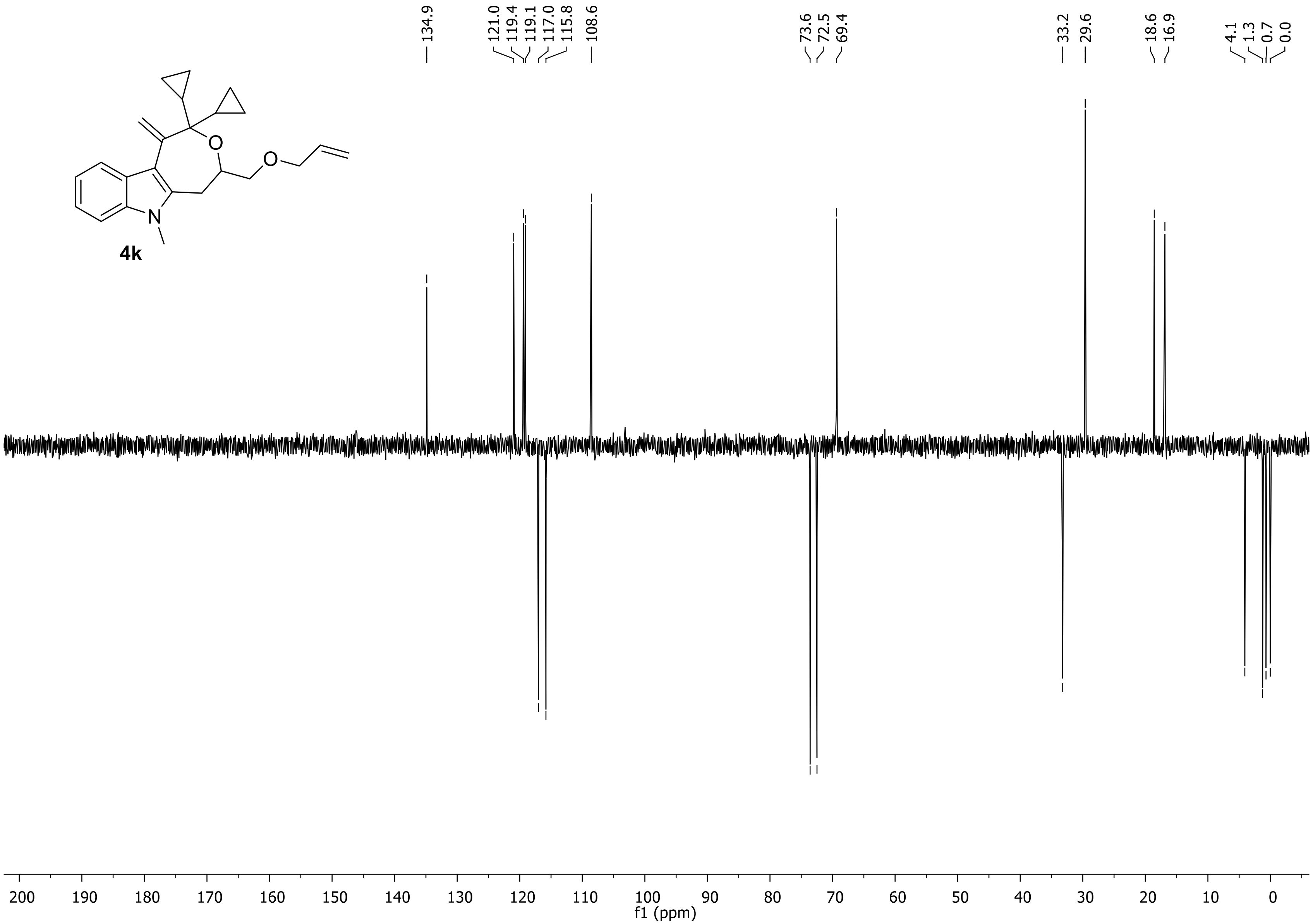

^{1}H NMR ($CDCl_3$, 500 MHz)

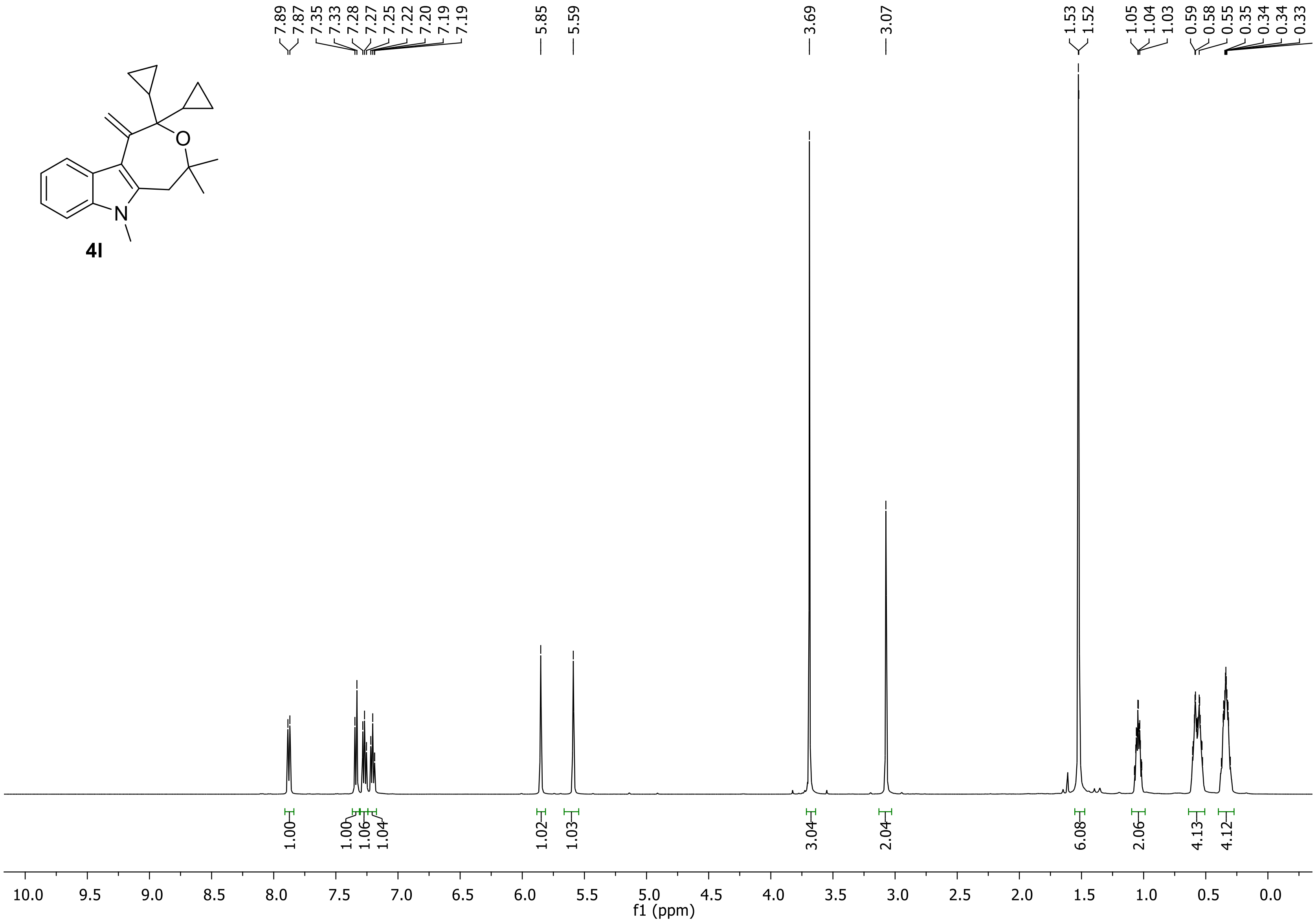

^{13}C NMR ($CDCl_3$, 125.7 MHz)

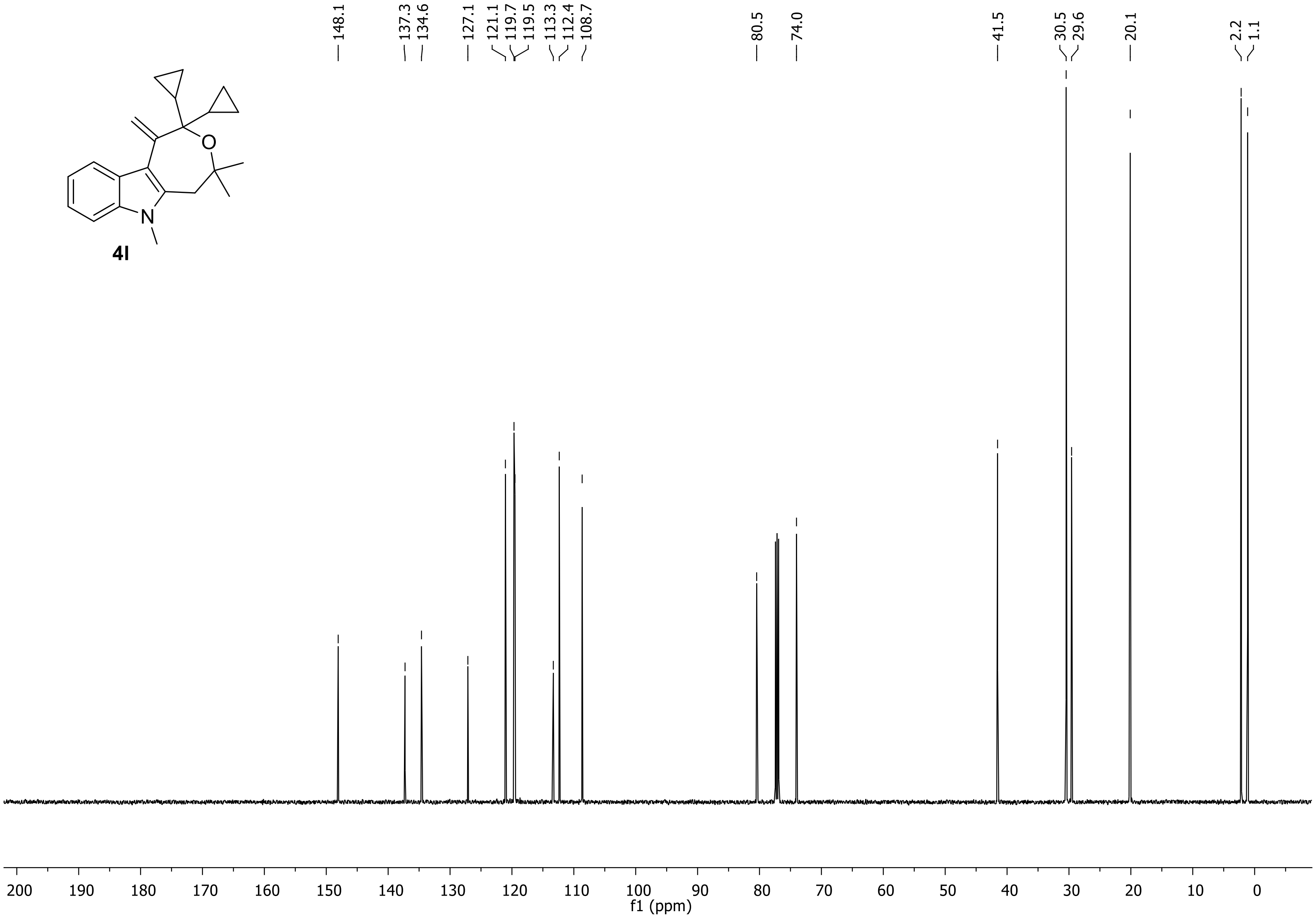

4l

DEPT ($CDCl_3$, 125.7 MHz)

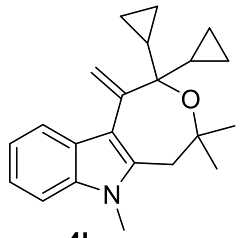

4l

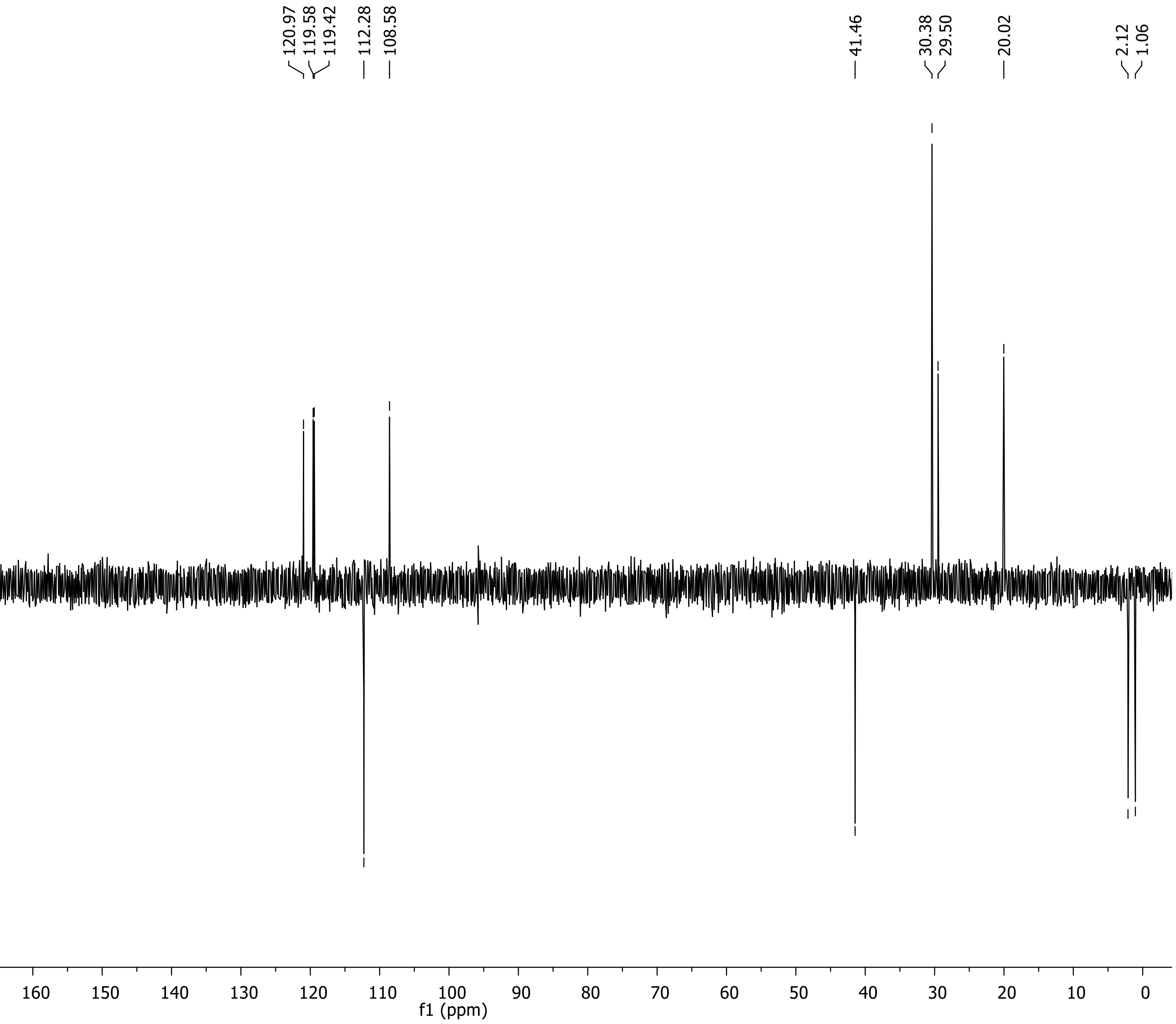

^{1}H NMR ($CDCl_3$, 500 MHz)

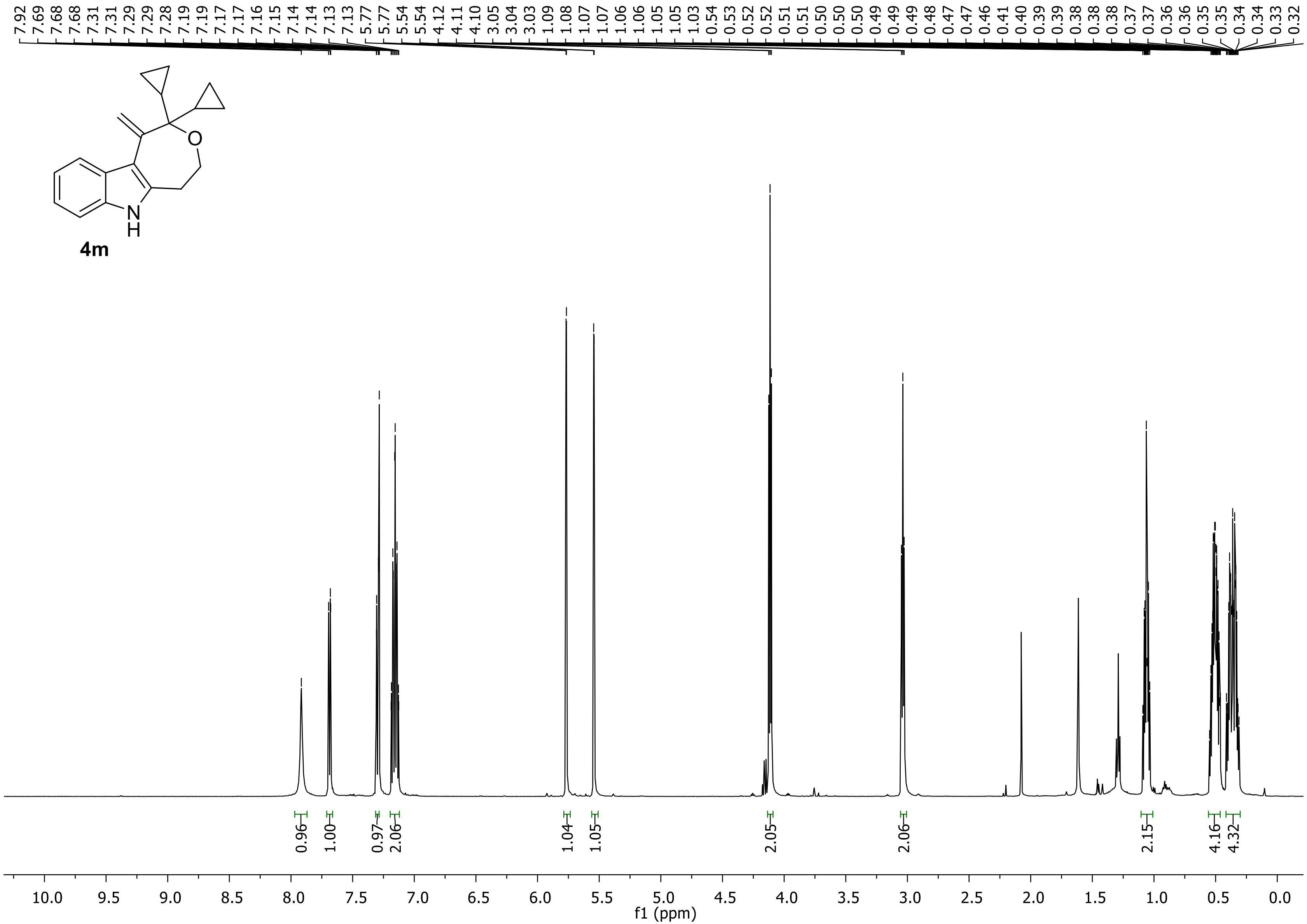

^{13}C NMR ($CDCl_3$, 125.7 MHz)

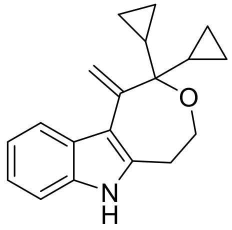

4m

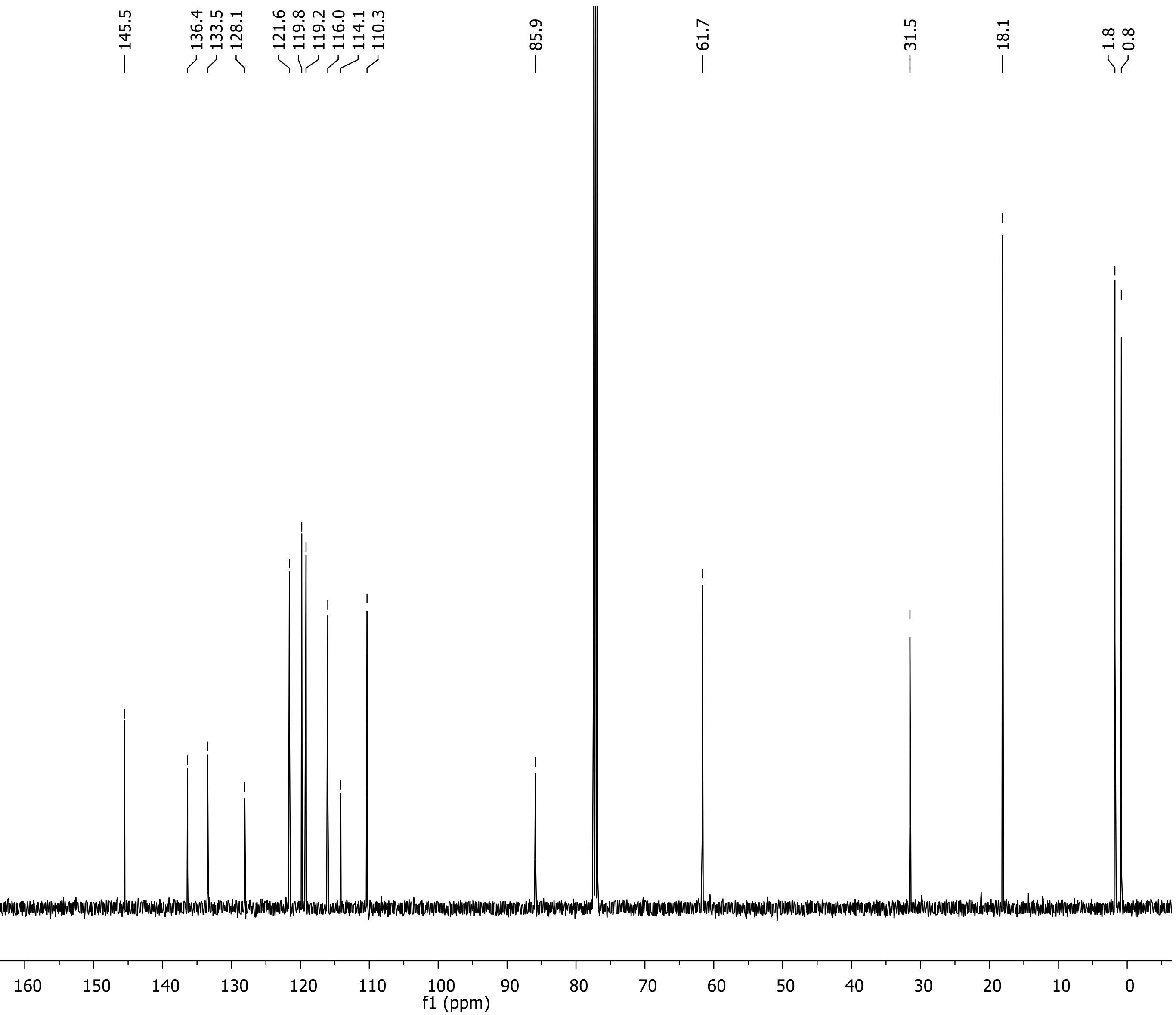

DEPT ($CDCl_3$, 125.7 MHz)

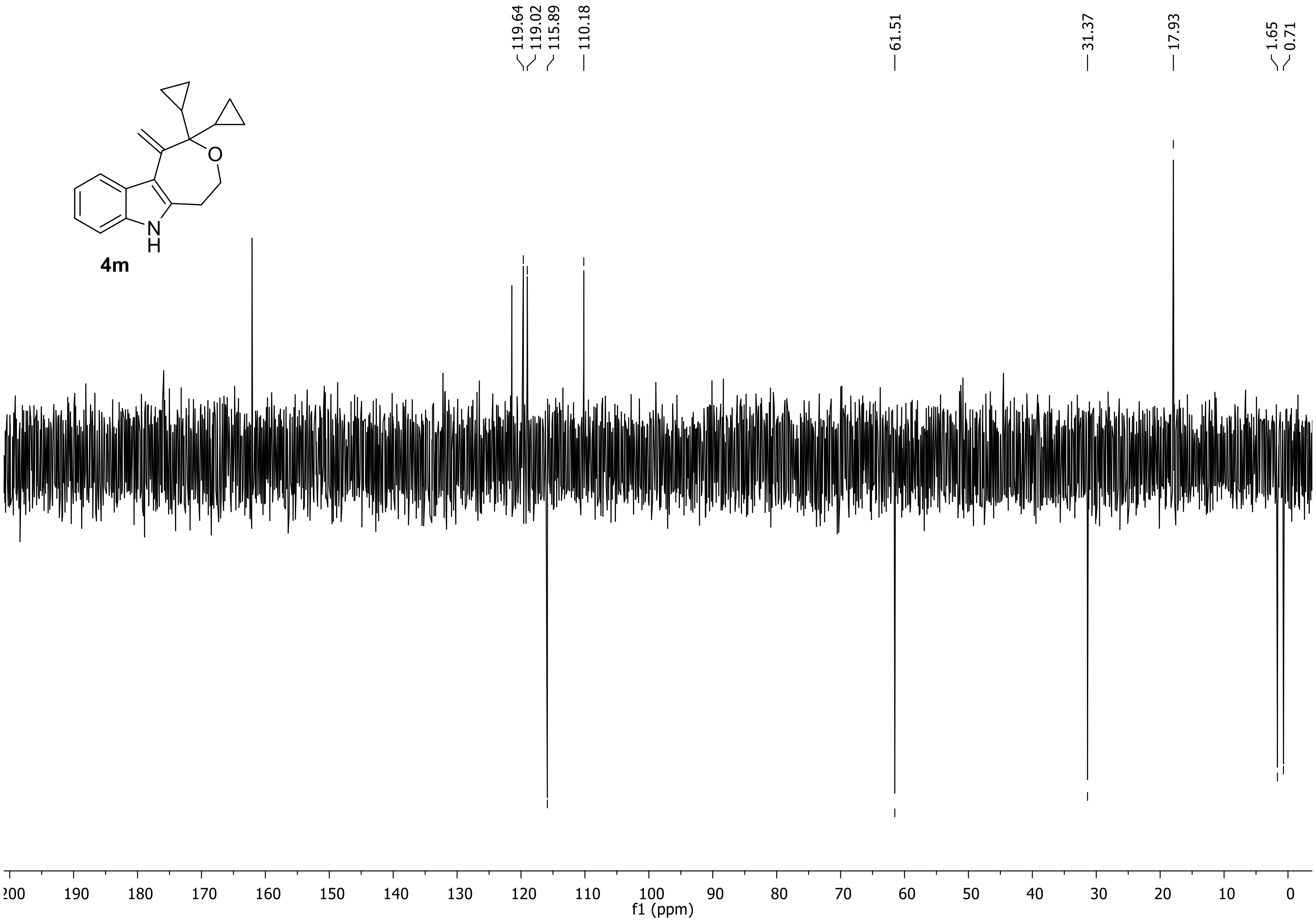

^{1}H NMR ($CDCl_3$, 500 MHz)

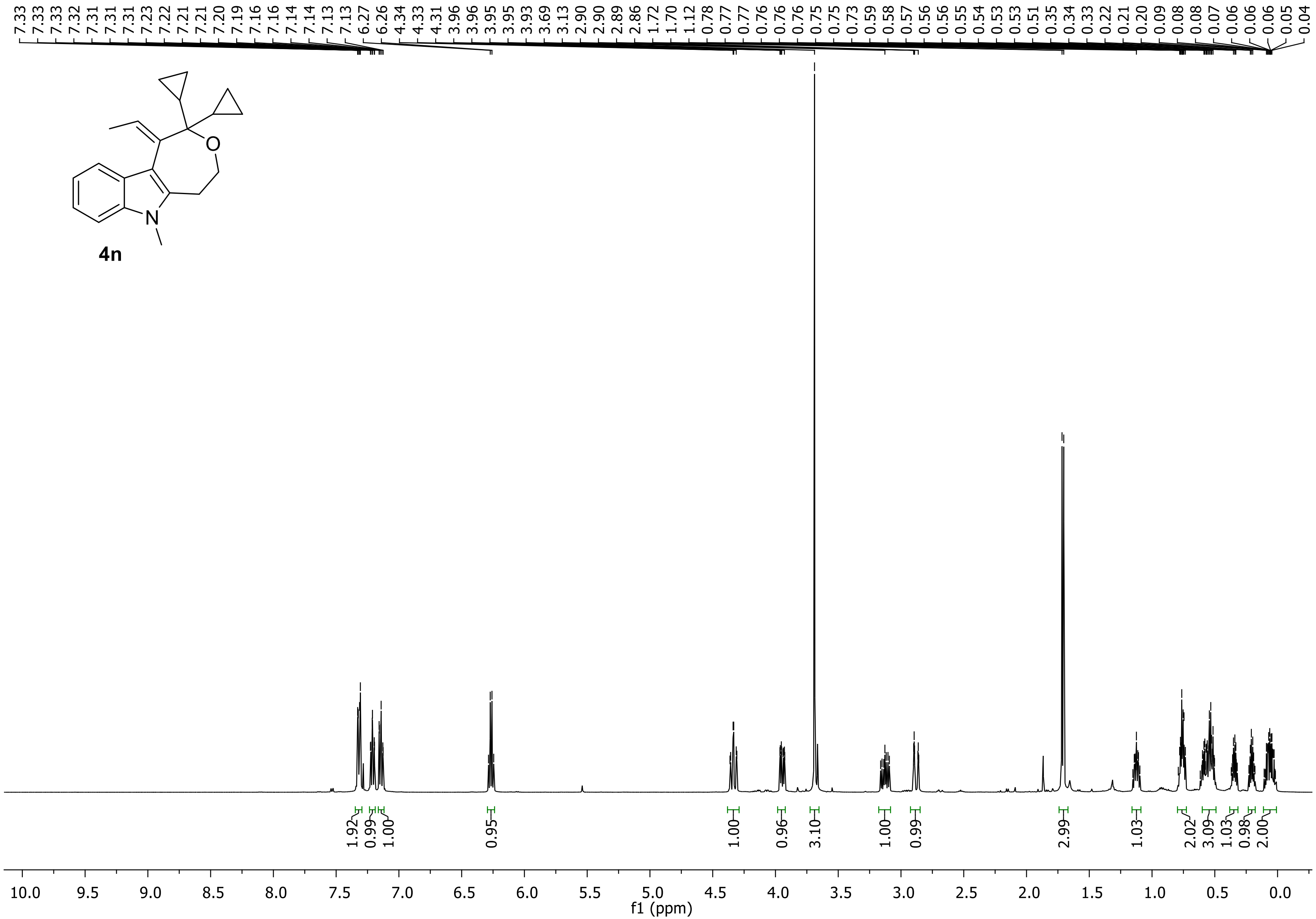

4n

^{13}C NMR ($CDCl_3$, 125.7 MHz)

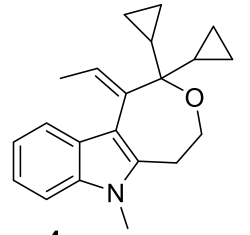

4n

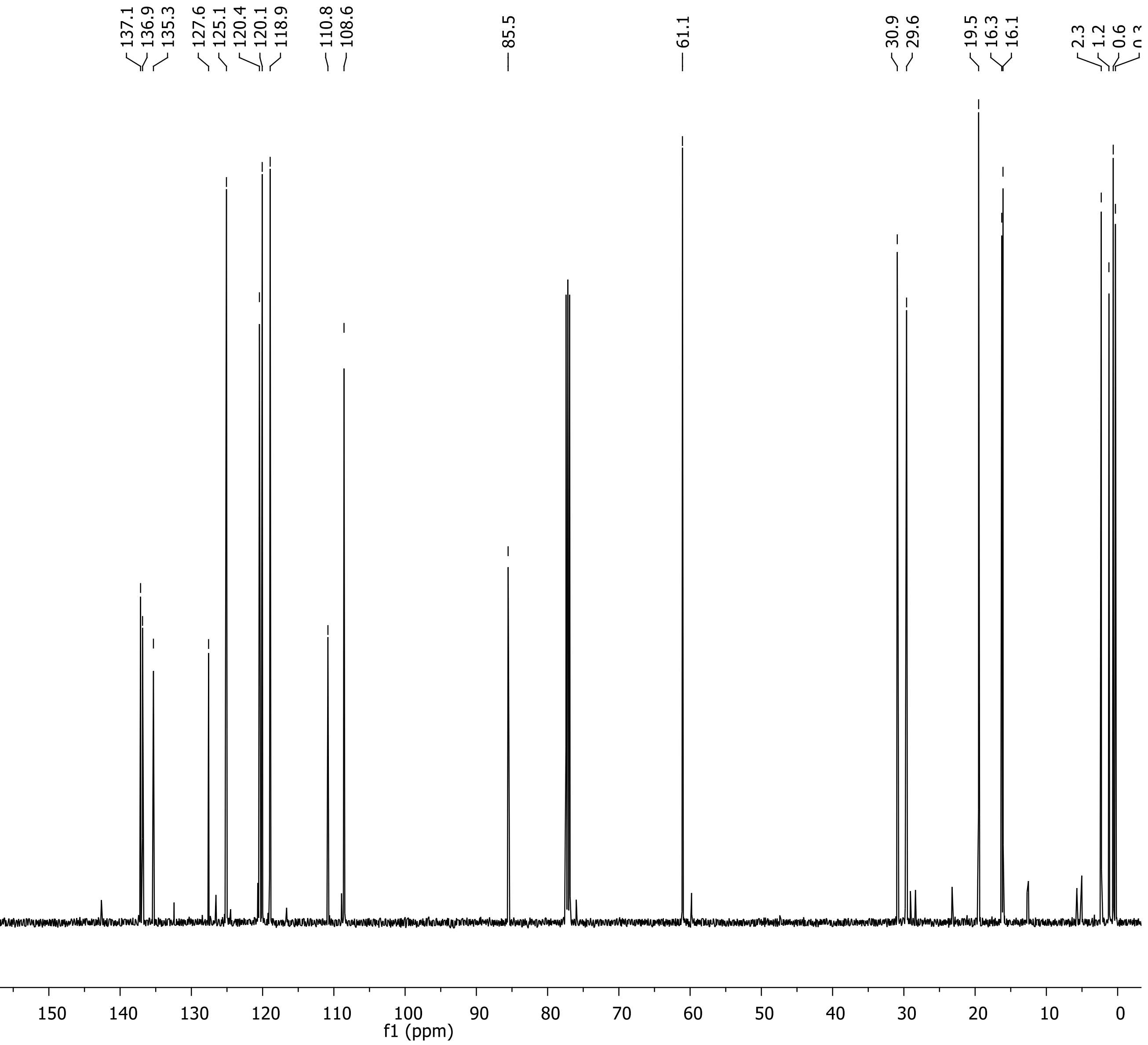

DEPT ($CDCl_3$, 125.7 MHz)

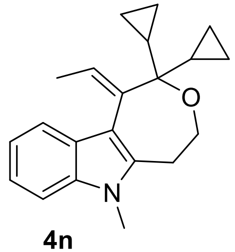

4n

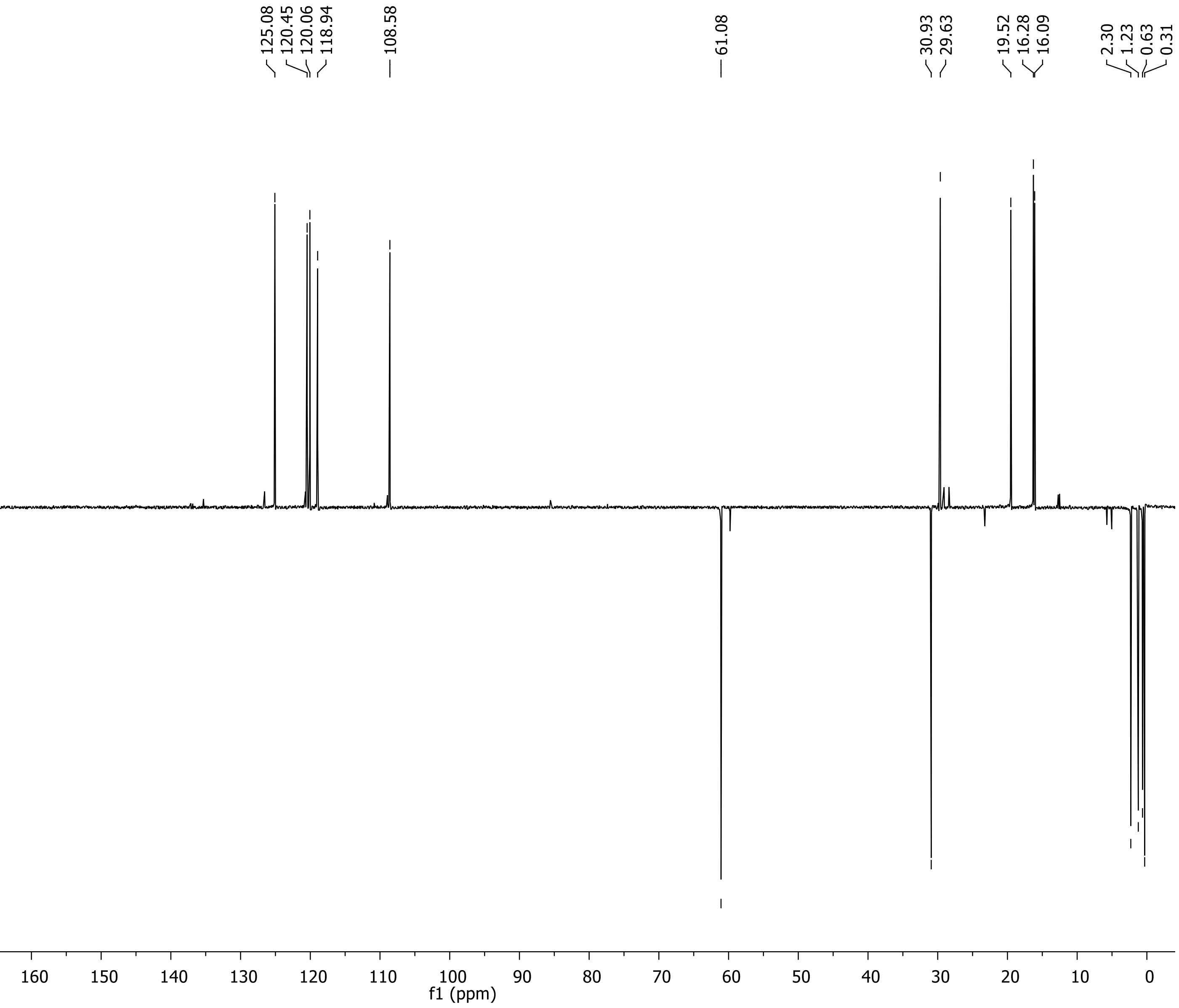

NOESY ($CDCl_3$, 500 MHz)

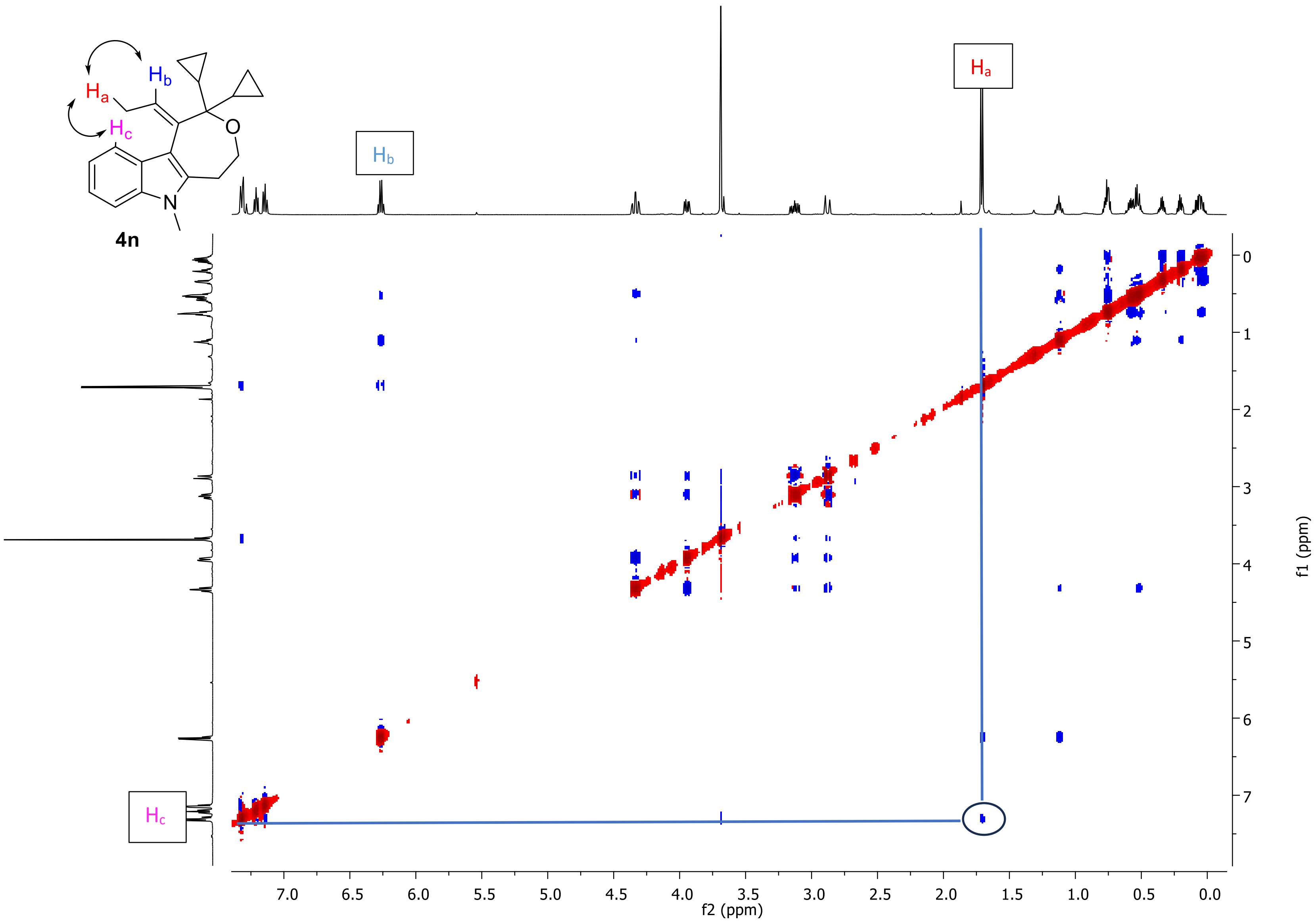

^{1}H NMR ($CDCl_3$, 300 MHz)

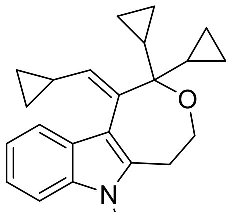

4o

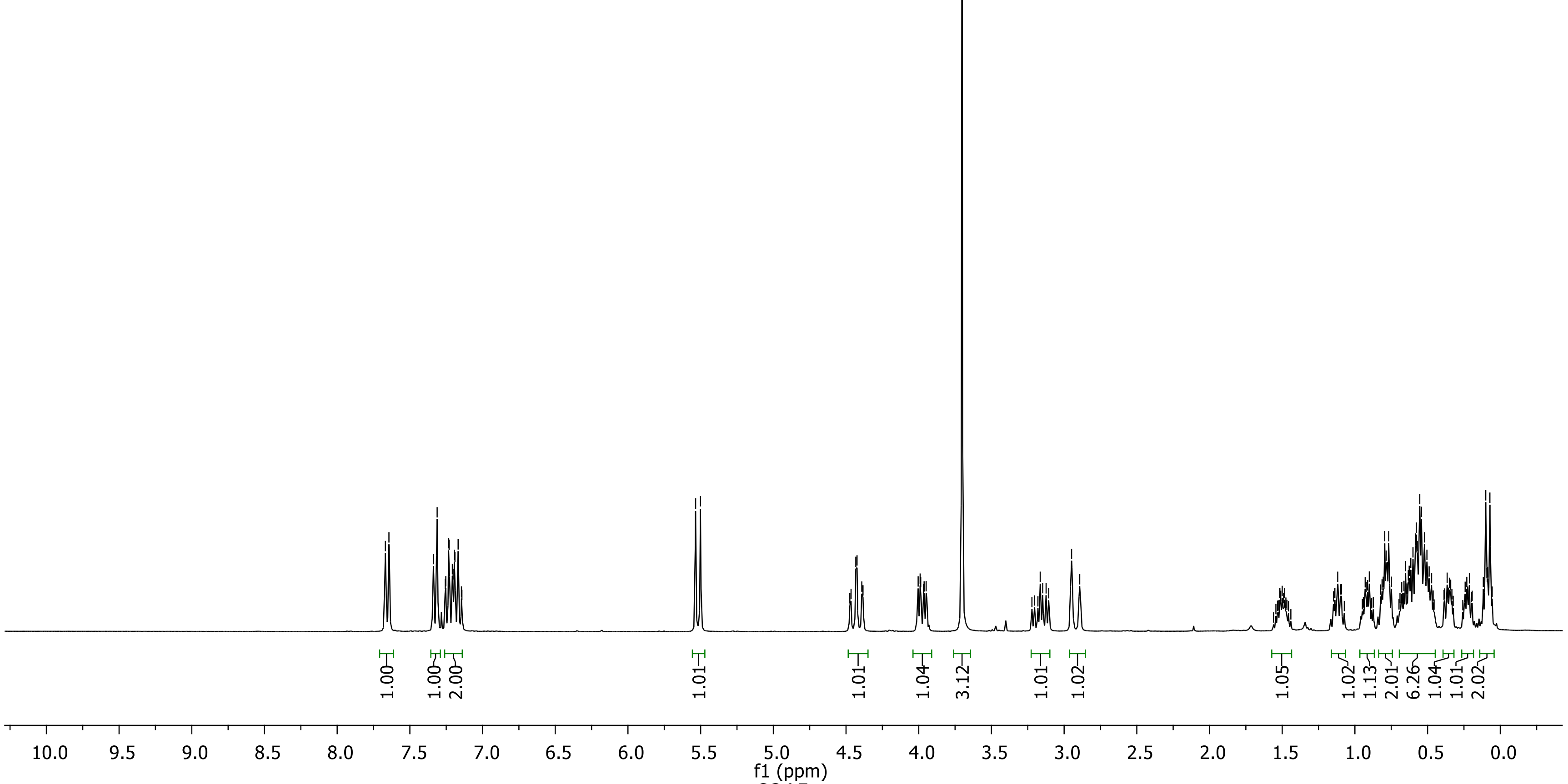

^{13}C NMR ($CDCl_3$, 75.4 MHz)

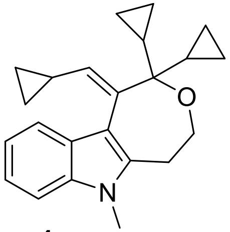

4o

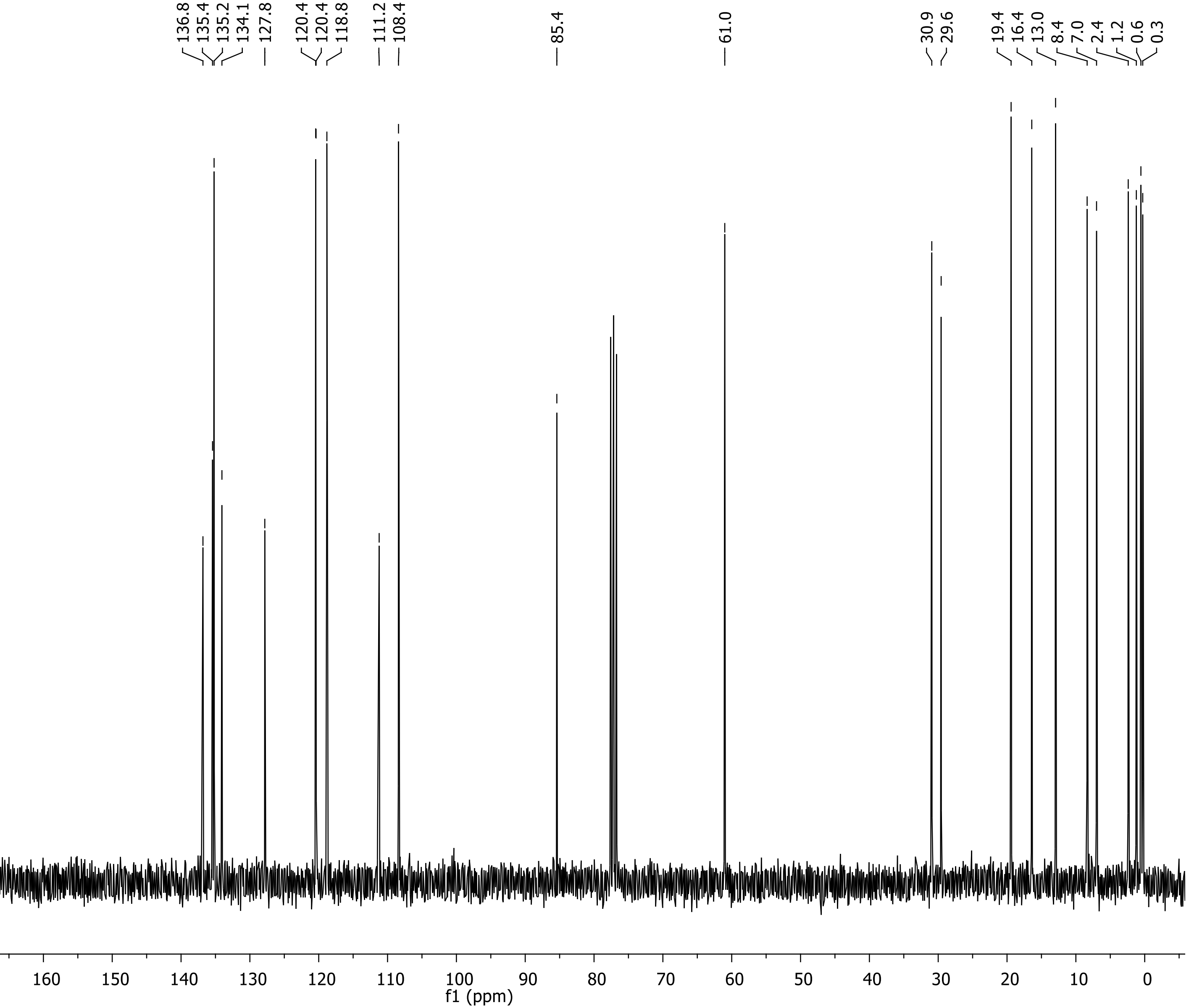

DEPT ($CDCl_3$, 75.4 MHz)

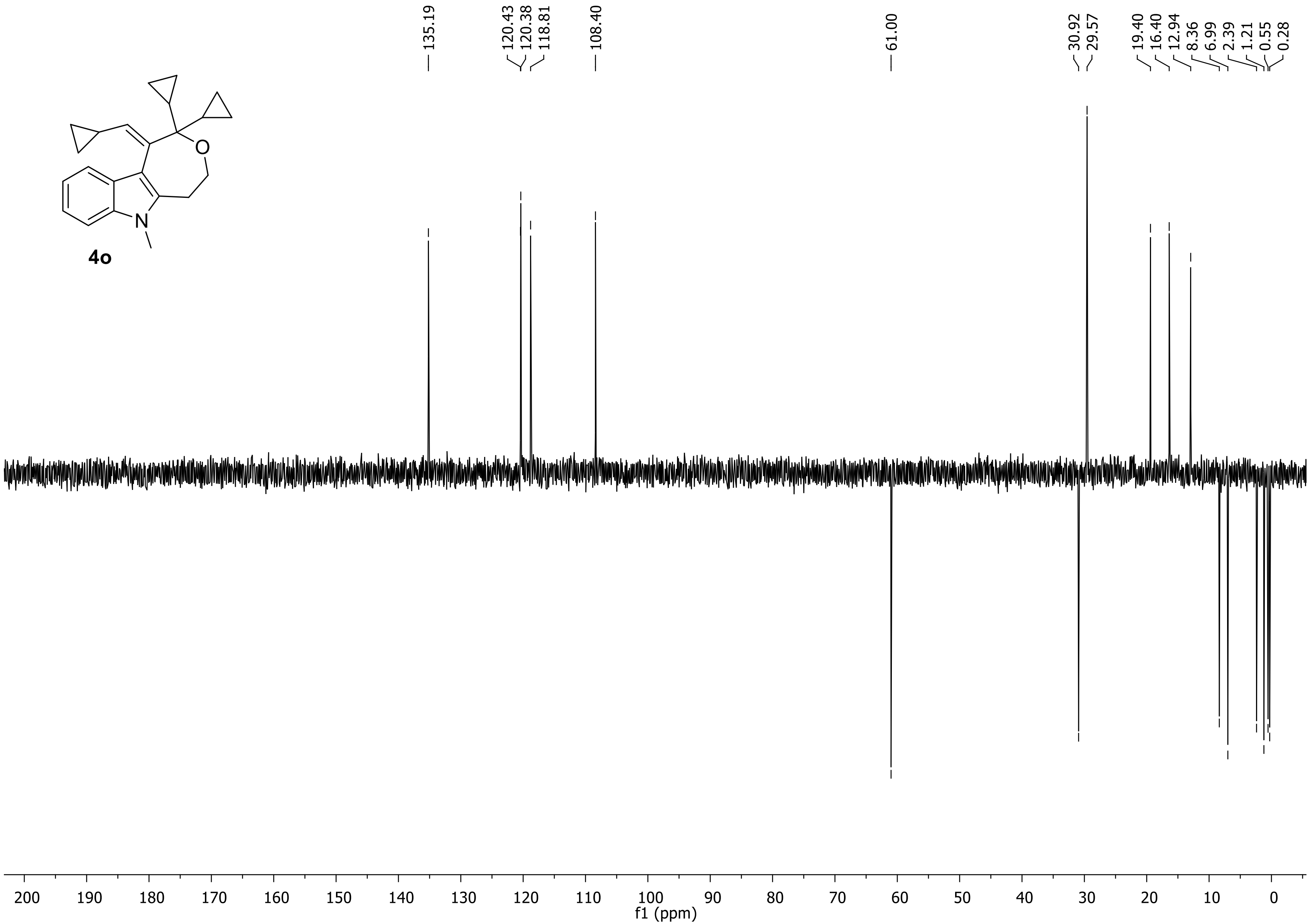

^{1}H NMR ($CDCl_3$, 300 MHz)

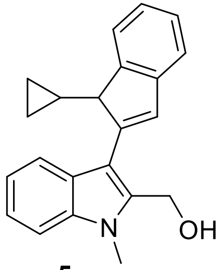

5

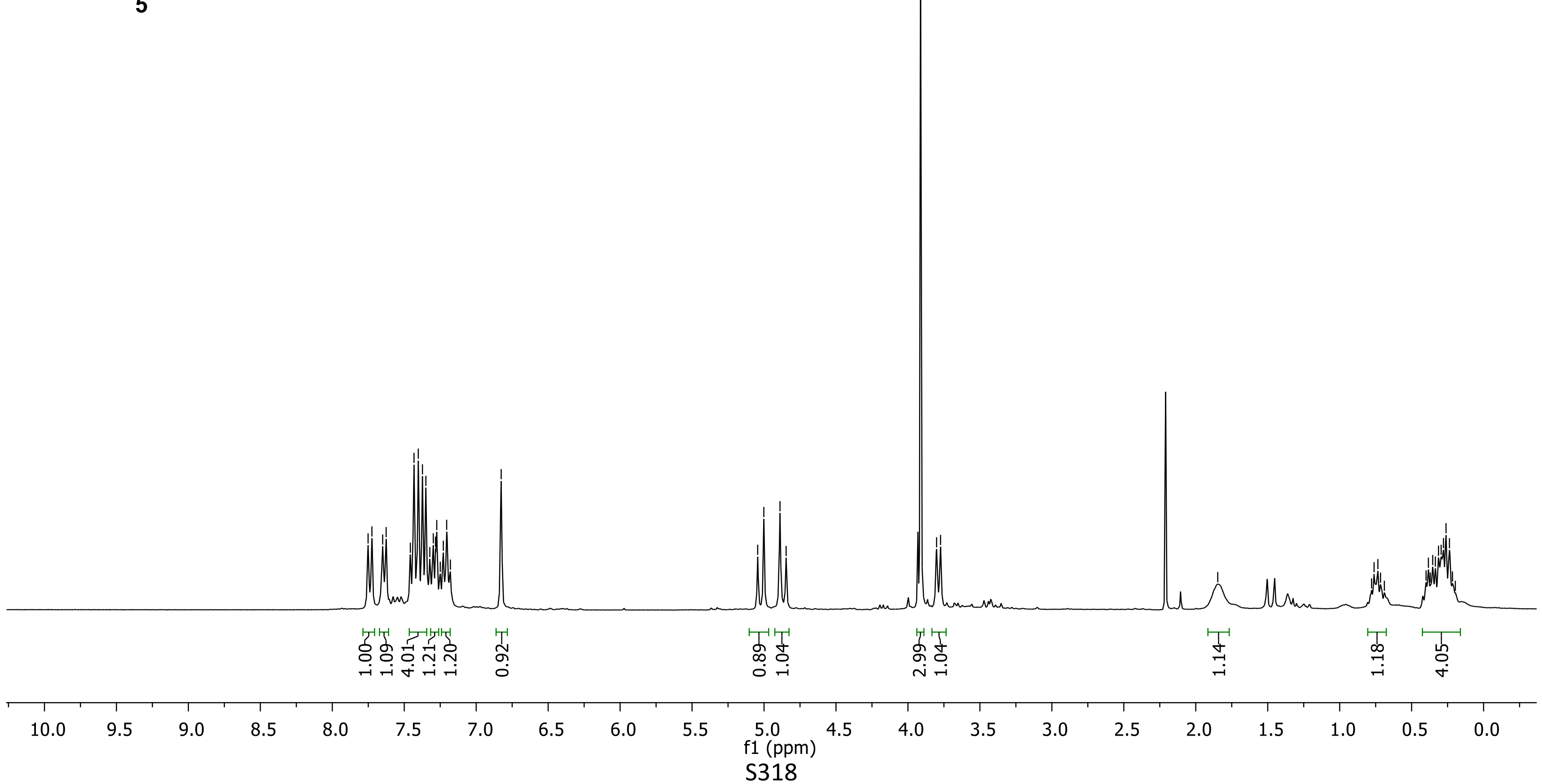

^{13}C NMR ($CDCl_3$, 75.4 MHz)

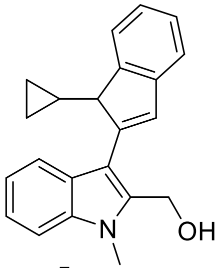

5

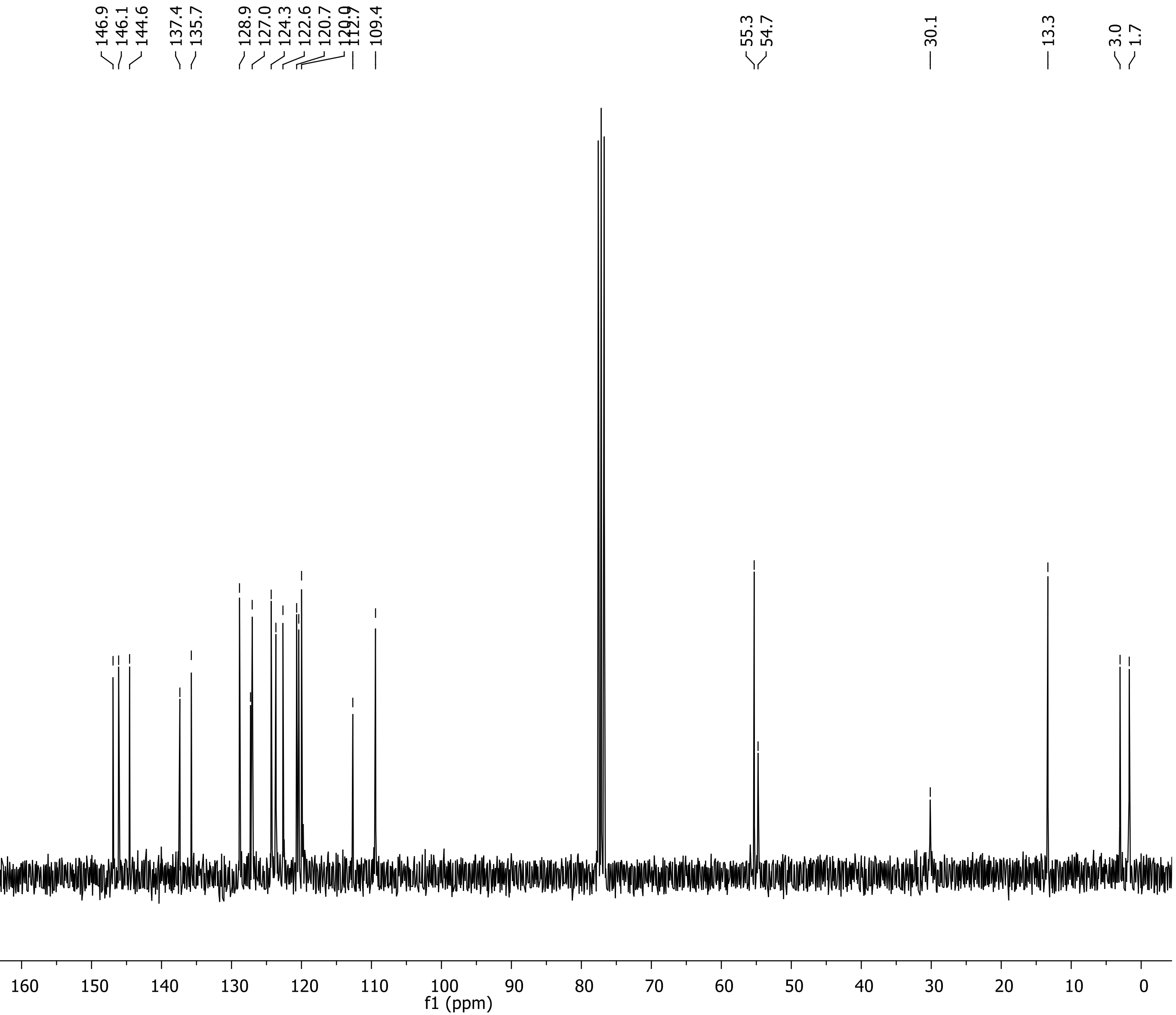

DEPT ($CDCl_3$, 75.4 MHz)

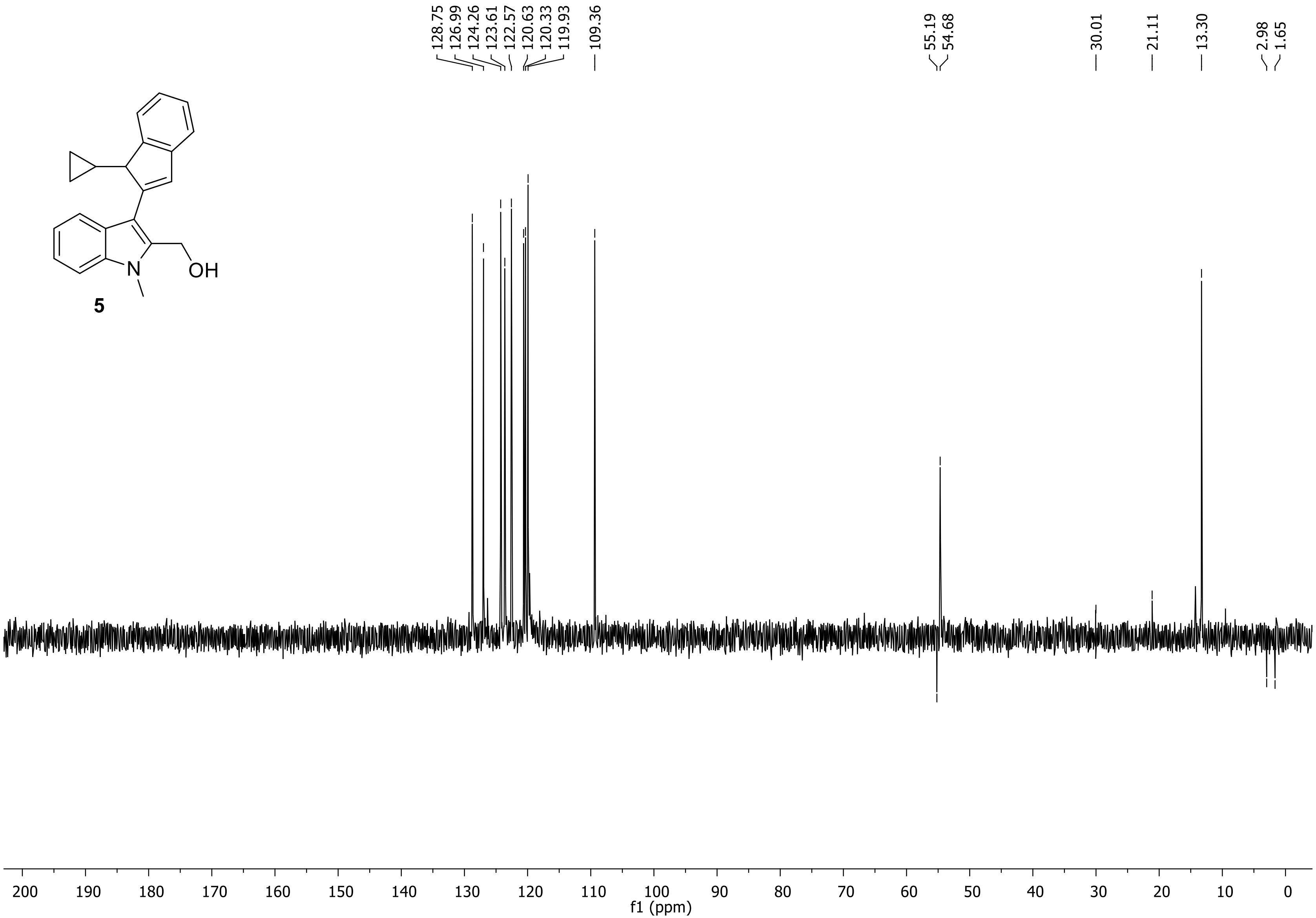

^{1}H NMR ($CDCl_3$, 300 MHz)

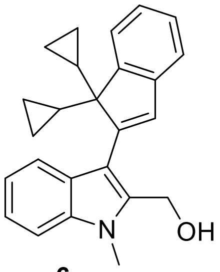

6

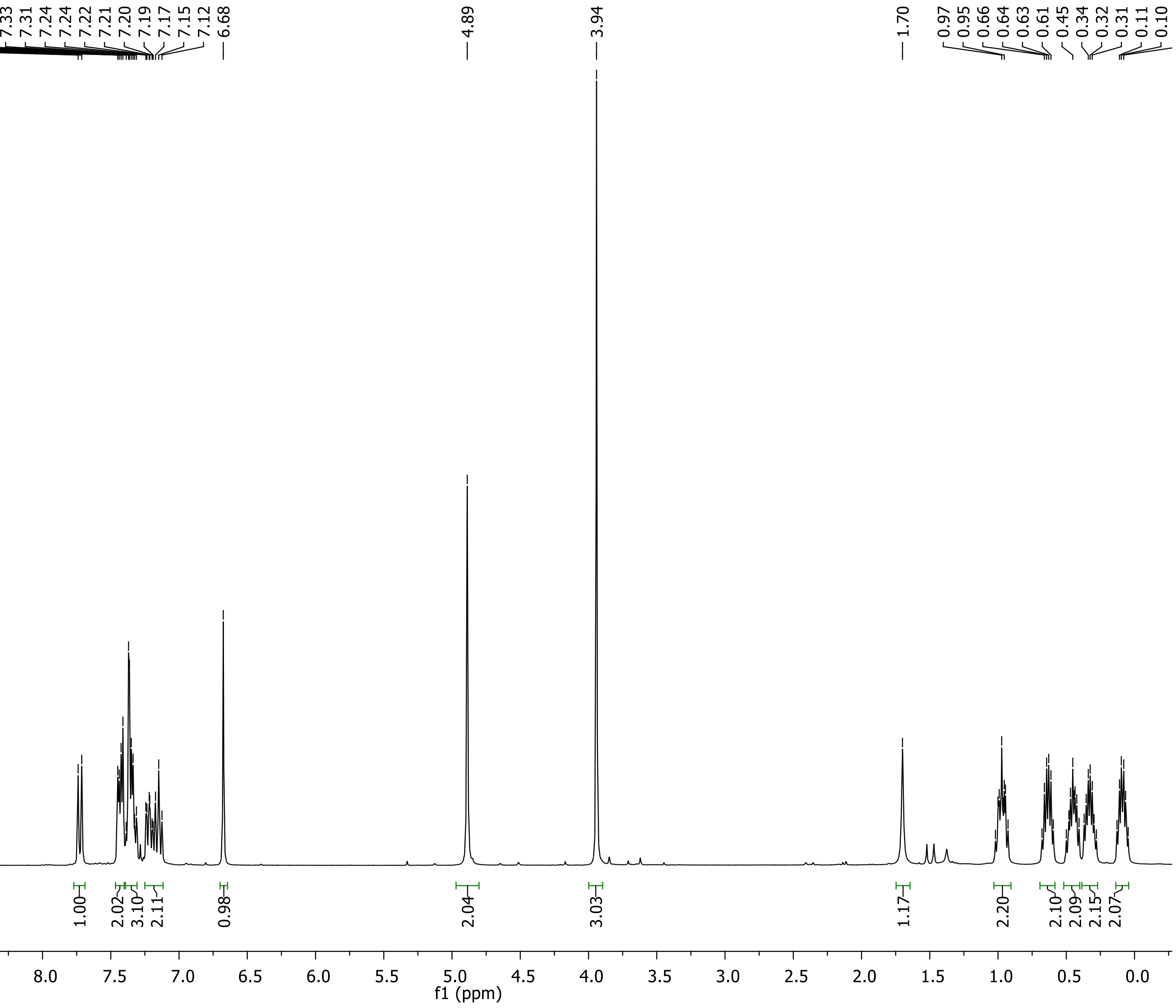

^{13}C NMR ($CDCl_3$, 75.4 MHz)

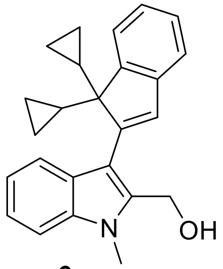

6

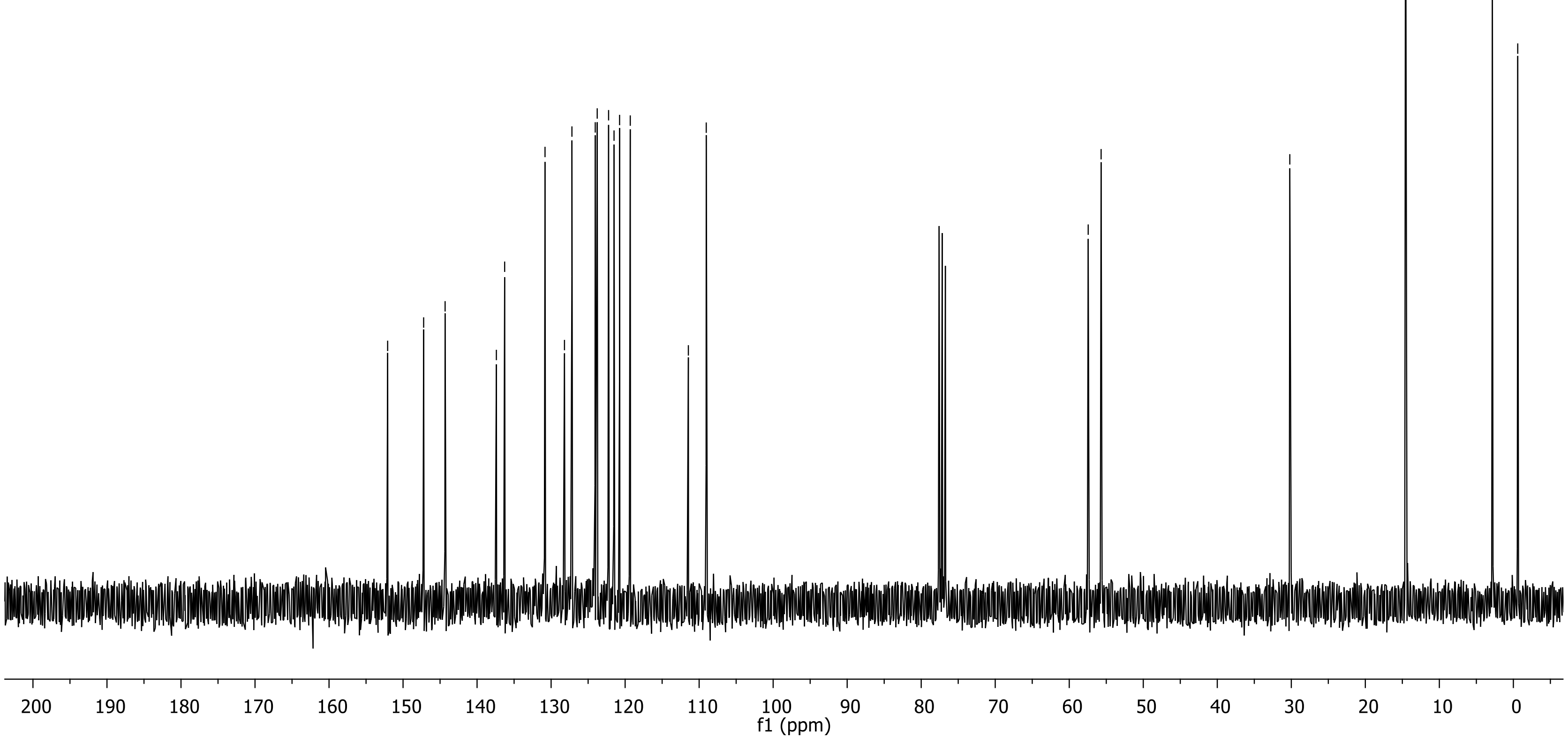

DEPT ($CDCl_3$, 75.4 MHz)

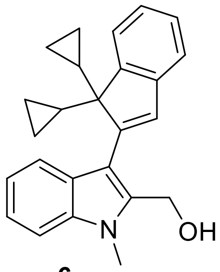

6

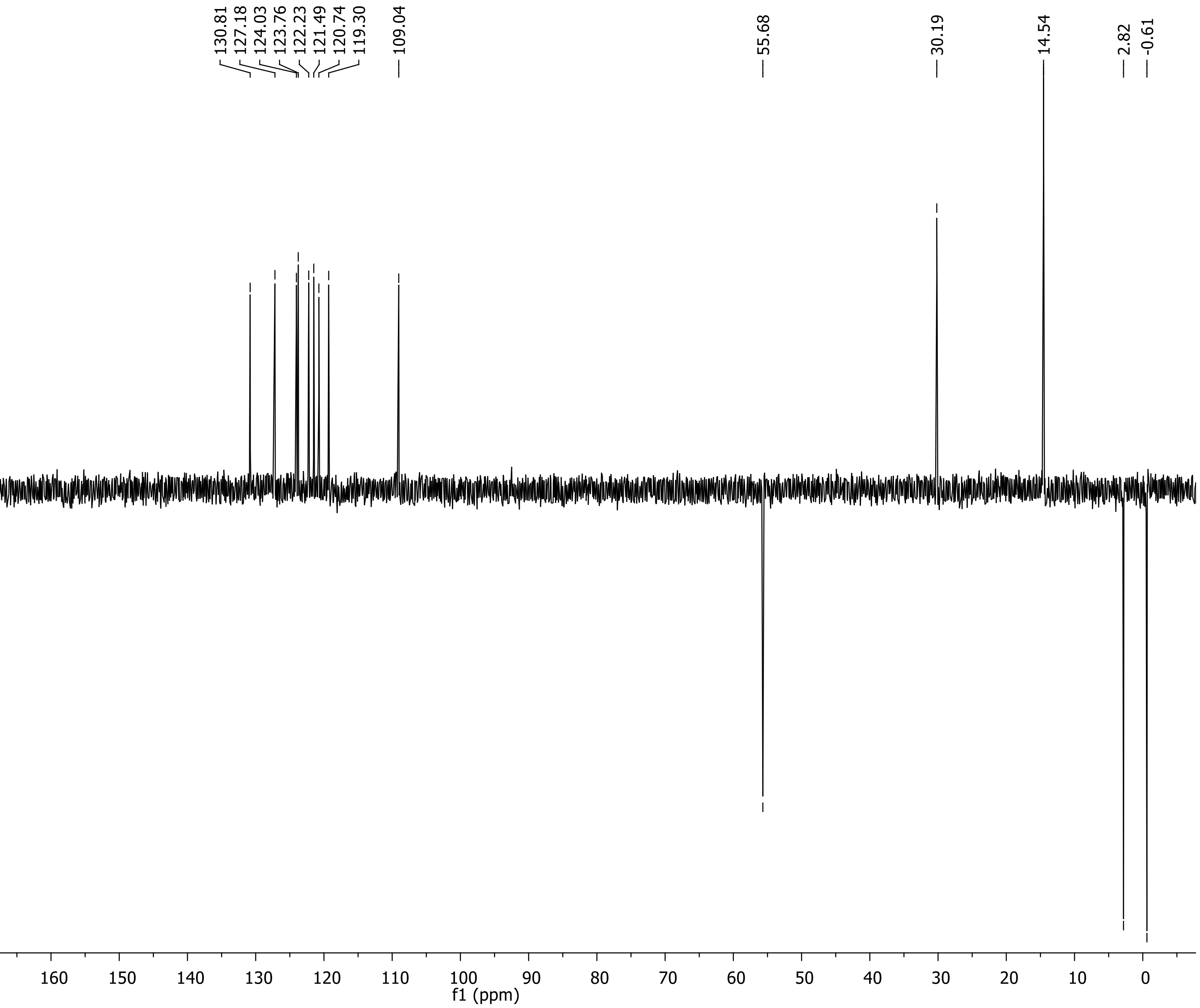

^1H NMR ((CD_3)$_2$O, 500 MHz)

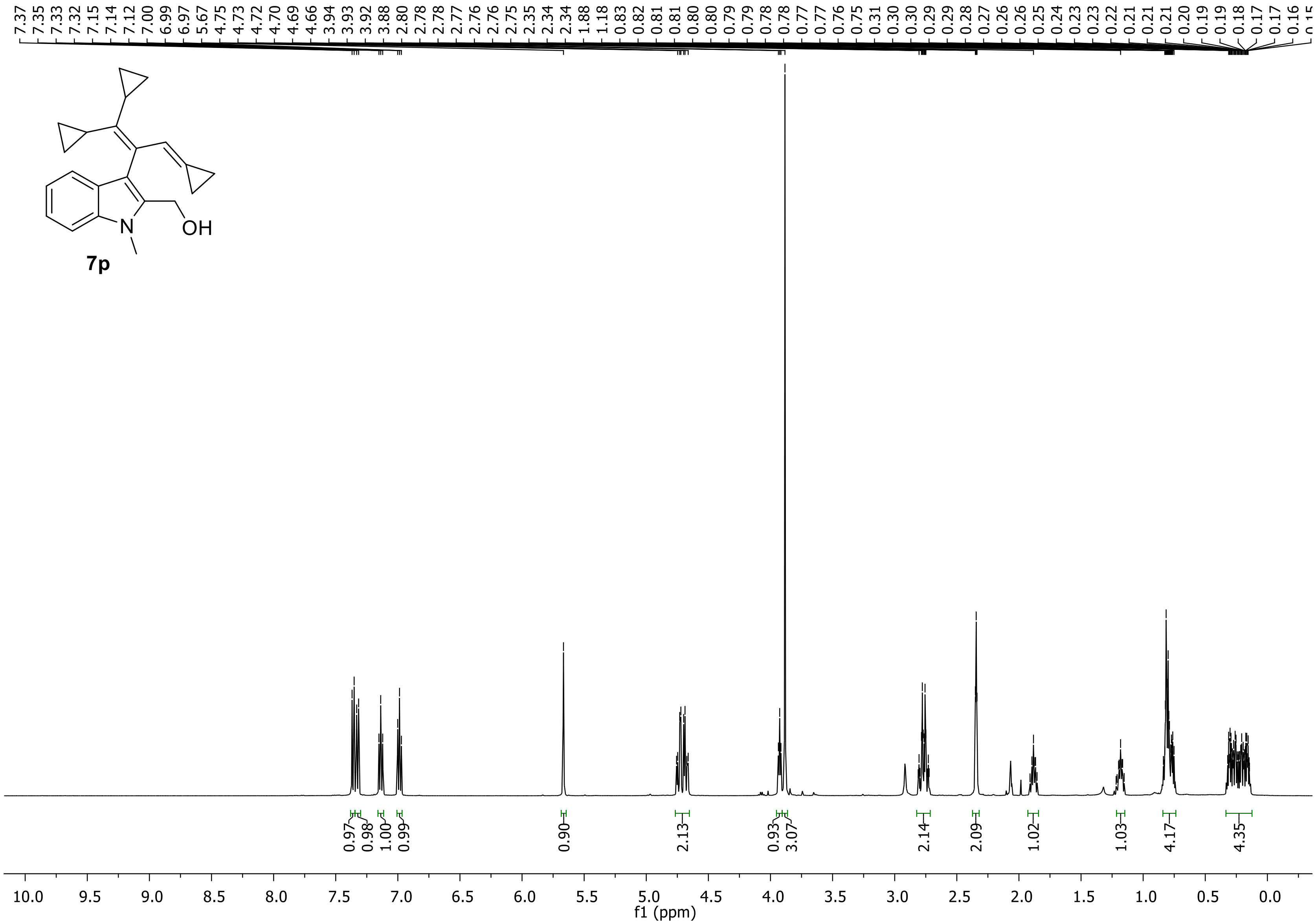

^{13}C NMR ($(CD_3)_2O$, 125.7 MHz)

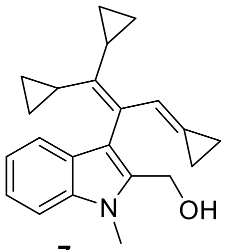

7p

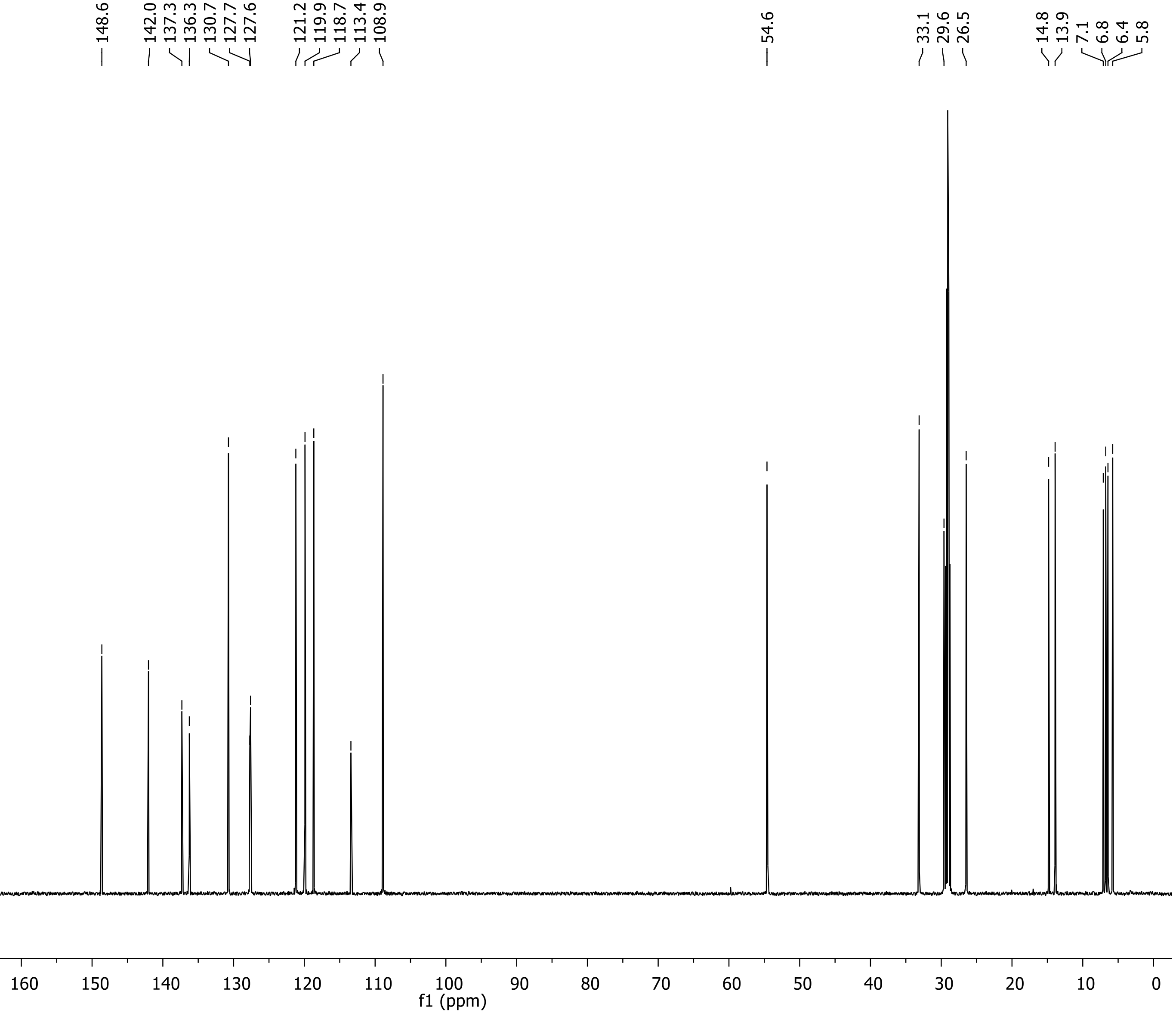

DEPT ((CD$_3$)$_2$O, 125.7 MHz)

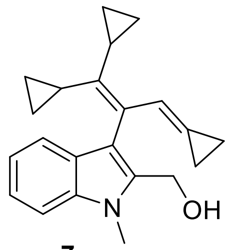

7p

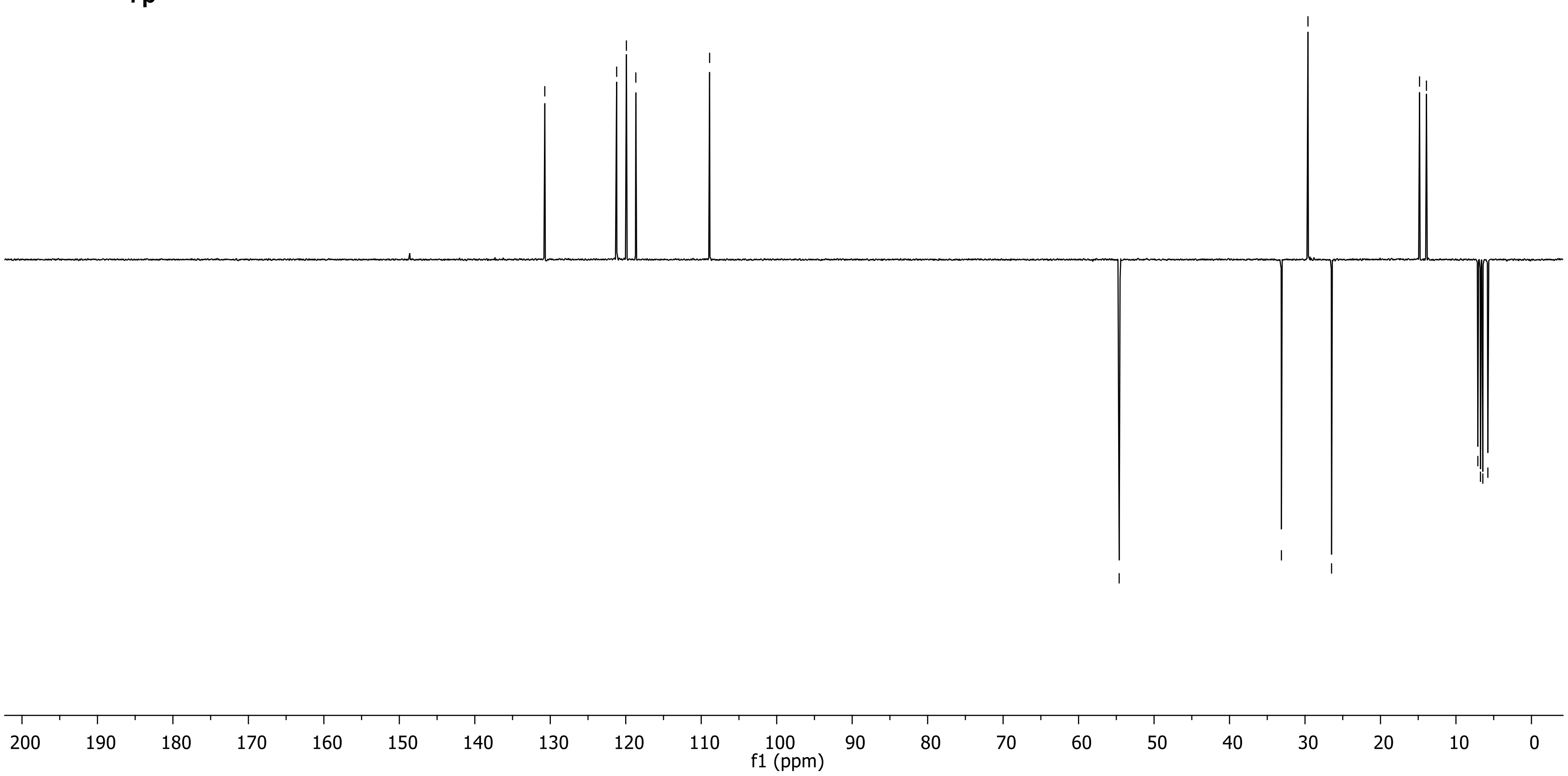

^{1}H NMR ($CDCl_3$, 300 MHz)

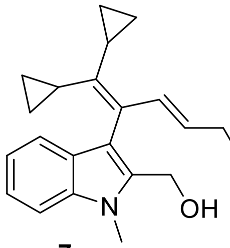

7q

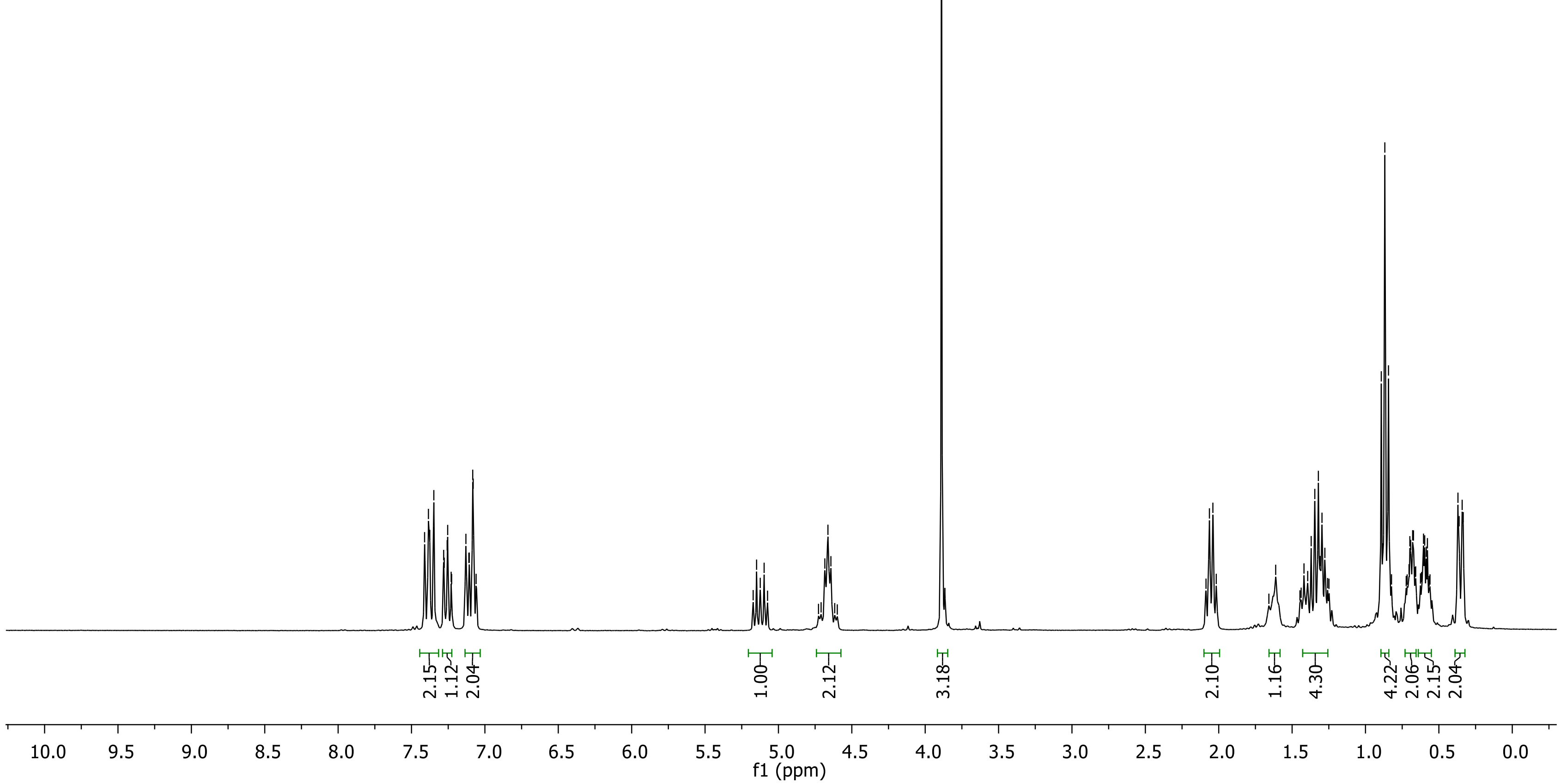

^{13}C NMR ($CDCl_3$, 75.4 MHz)

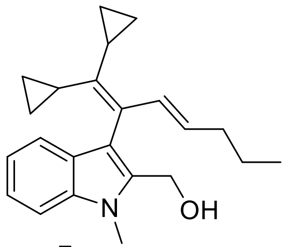

7q

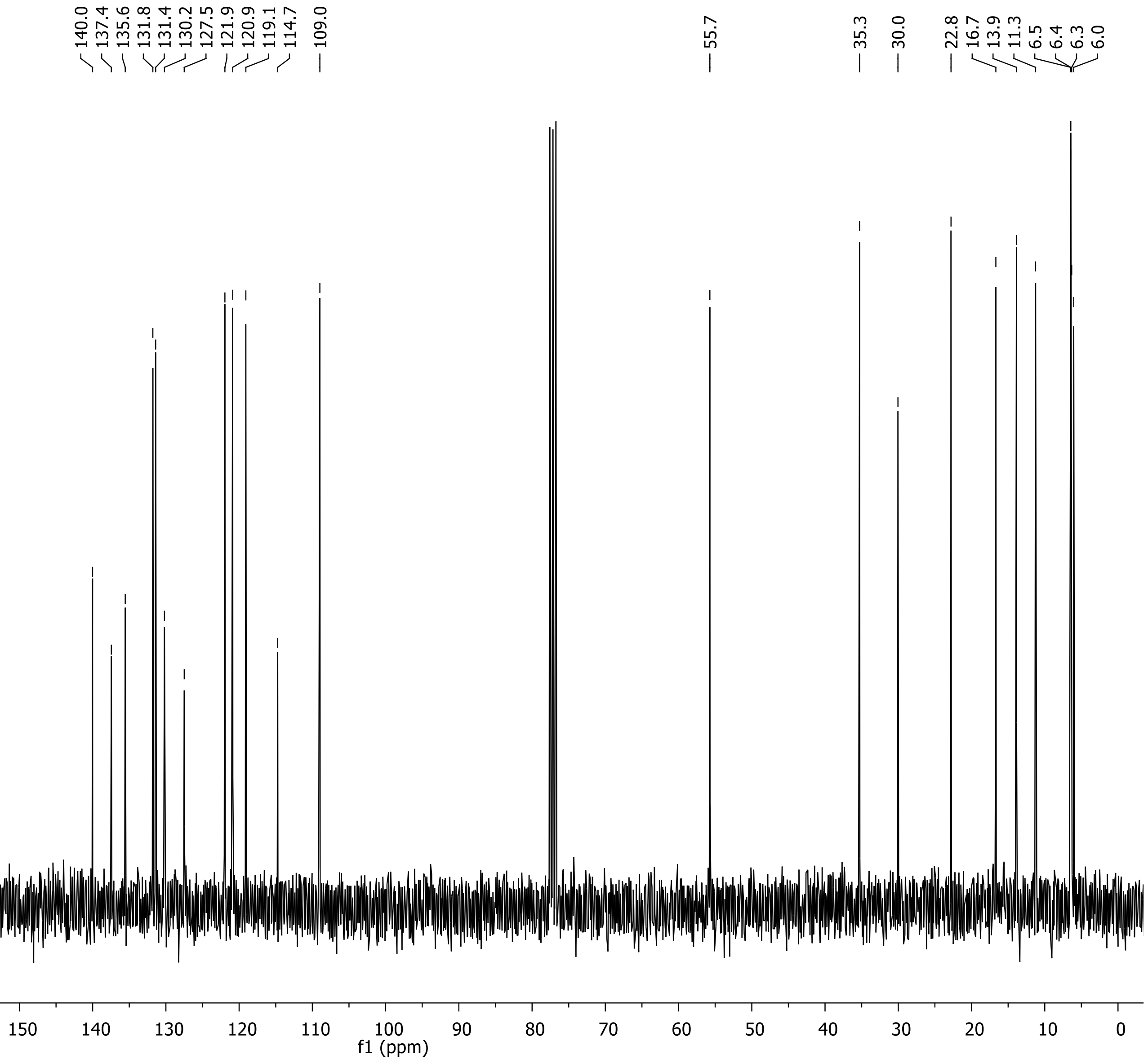

DEPT ($CDCl_3$, 75.4 MHz)

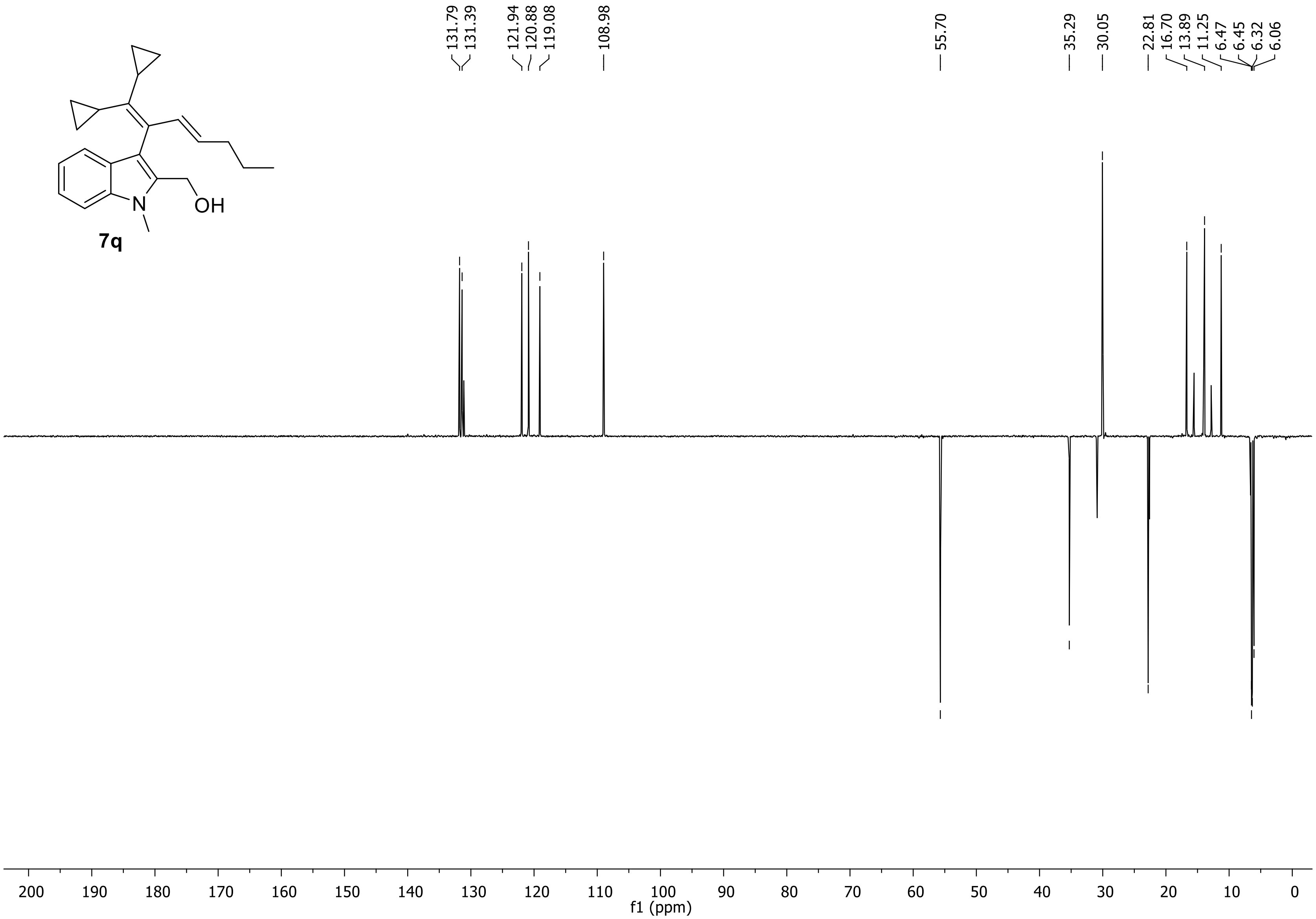

^1H NMR ($CDCl_3$, 300 MHz)

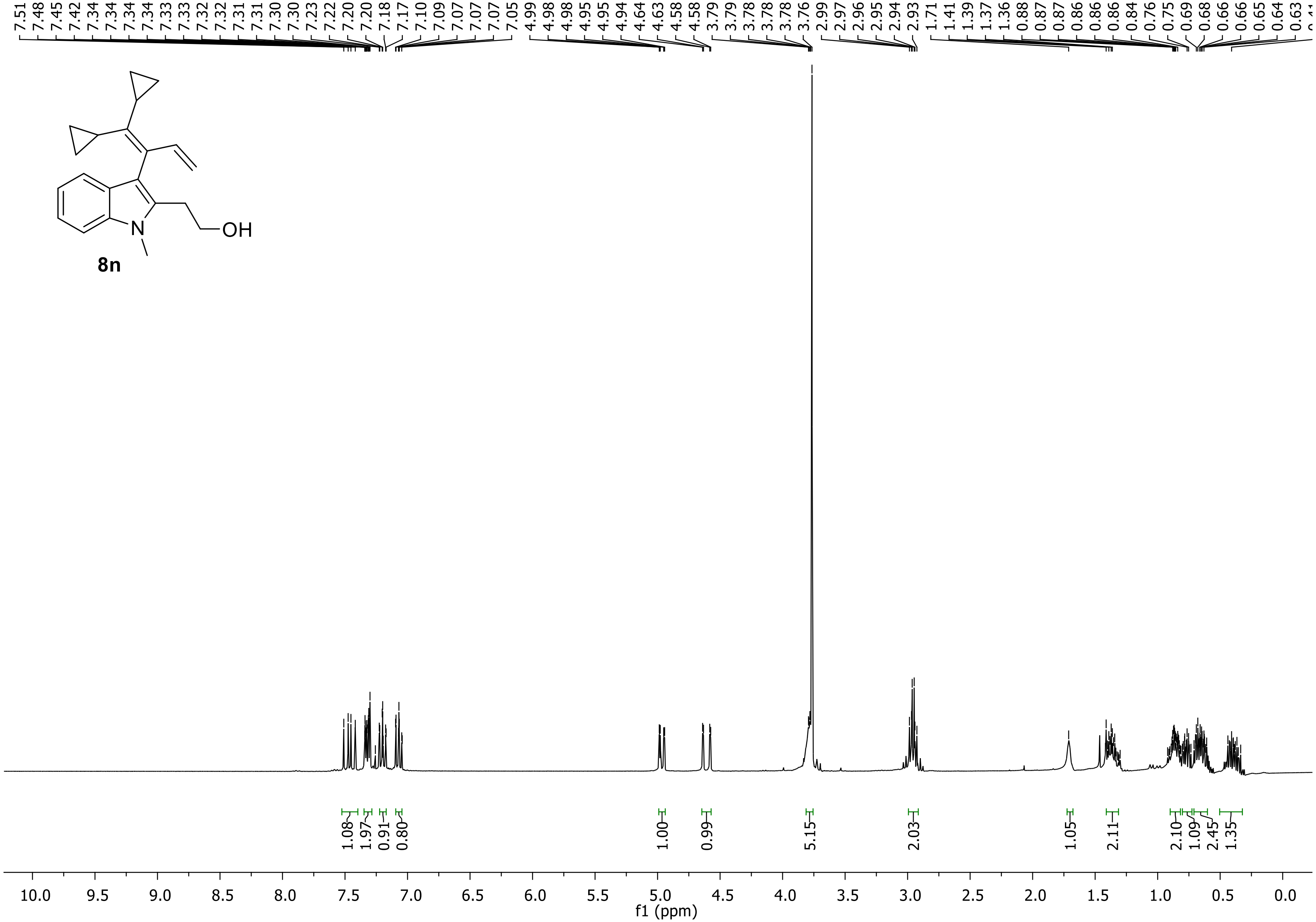

8n

^{13}C NMR ($CDCl_3$, 75.4 MHz)

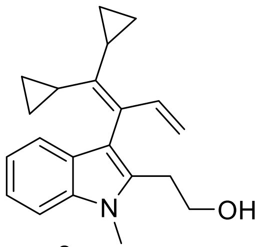

8n

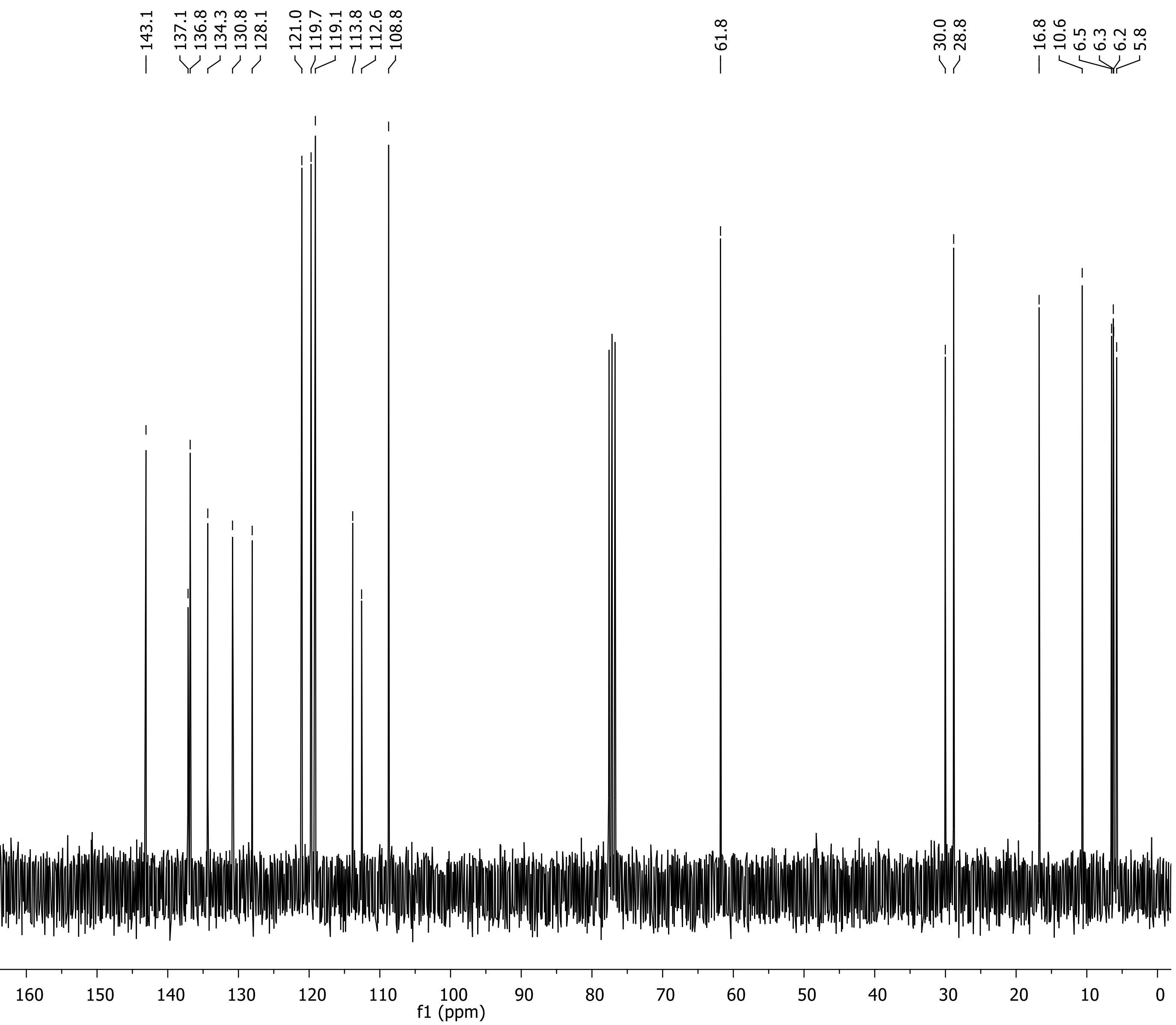

DEPT ($CDCl_3$, 75.4 MHz)

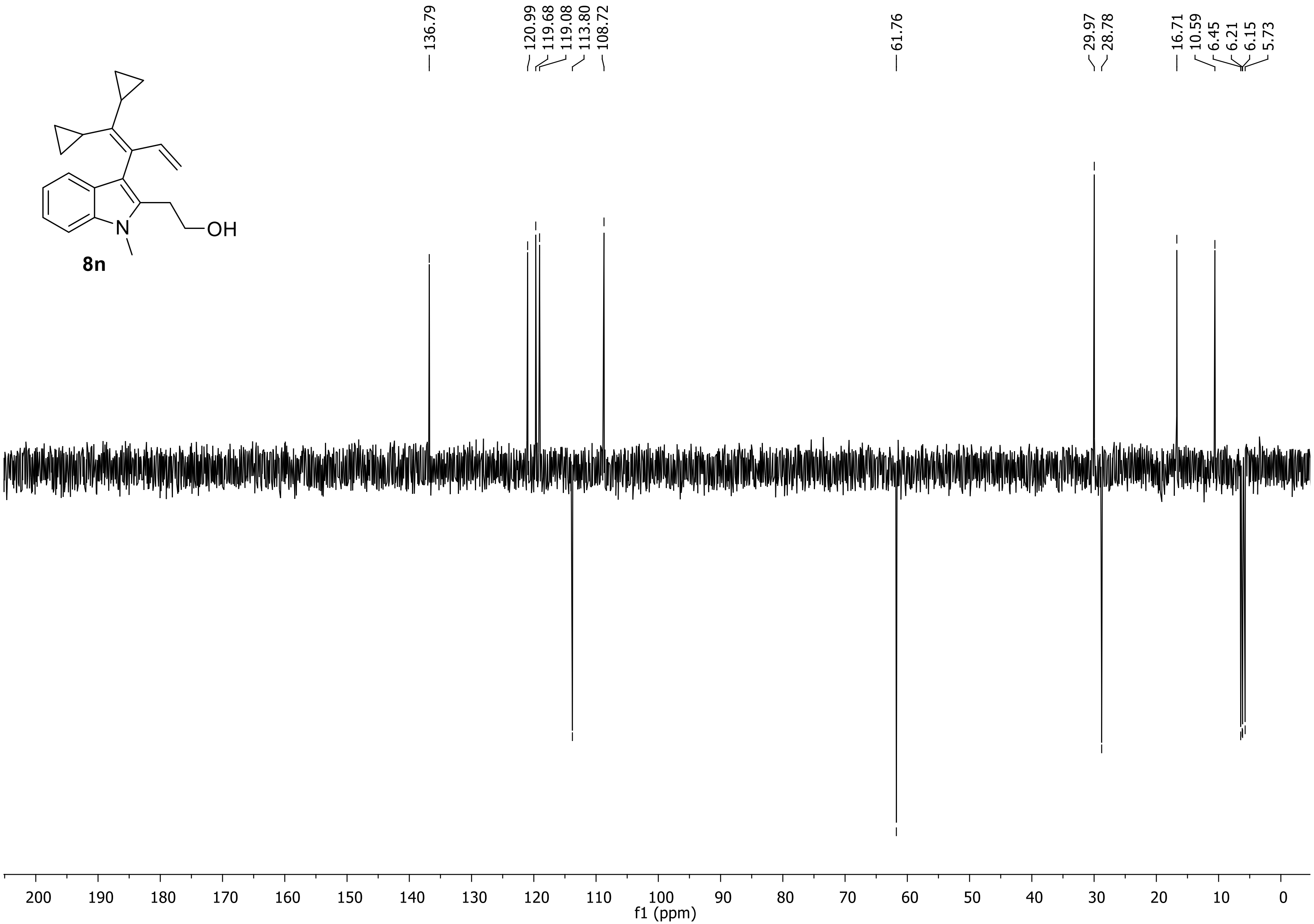

^{1}H NMR ($CDCl_3$, 300 MHz)

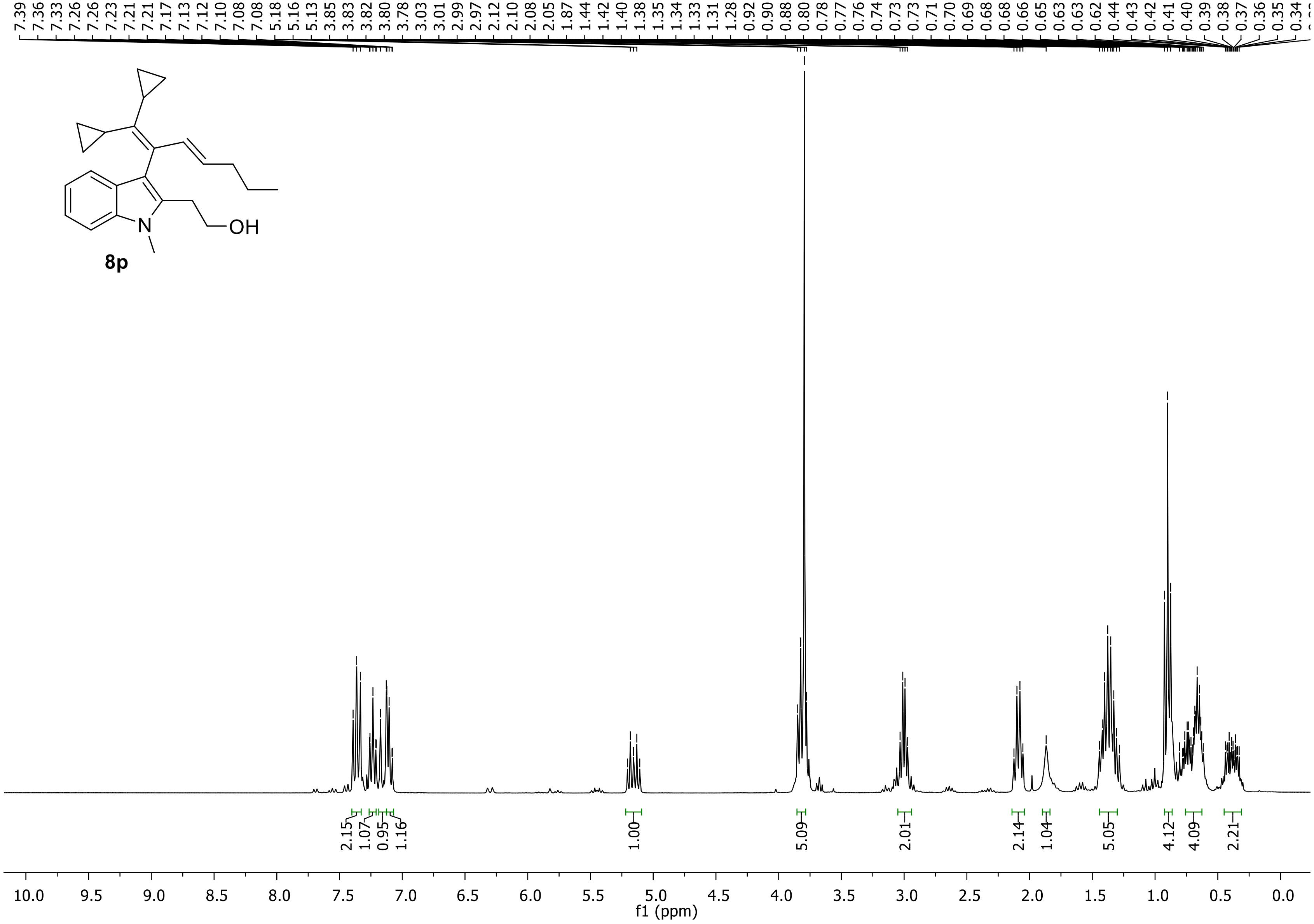

^{13}C NMR ($CDCl_3$, 75.4 MHz)

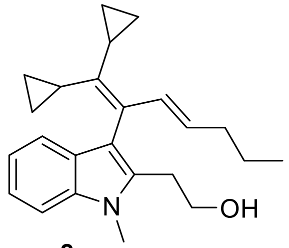

8p

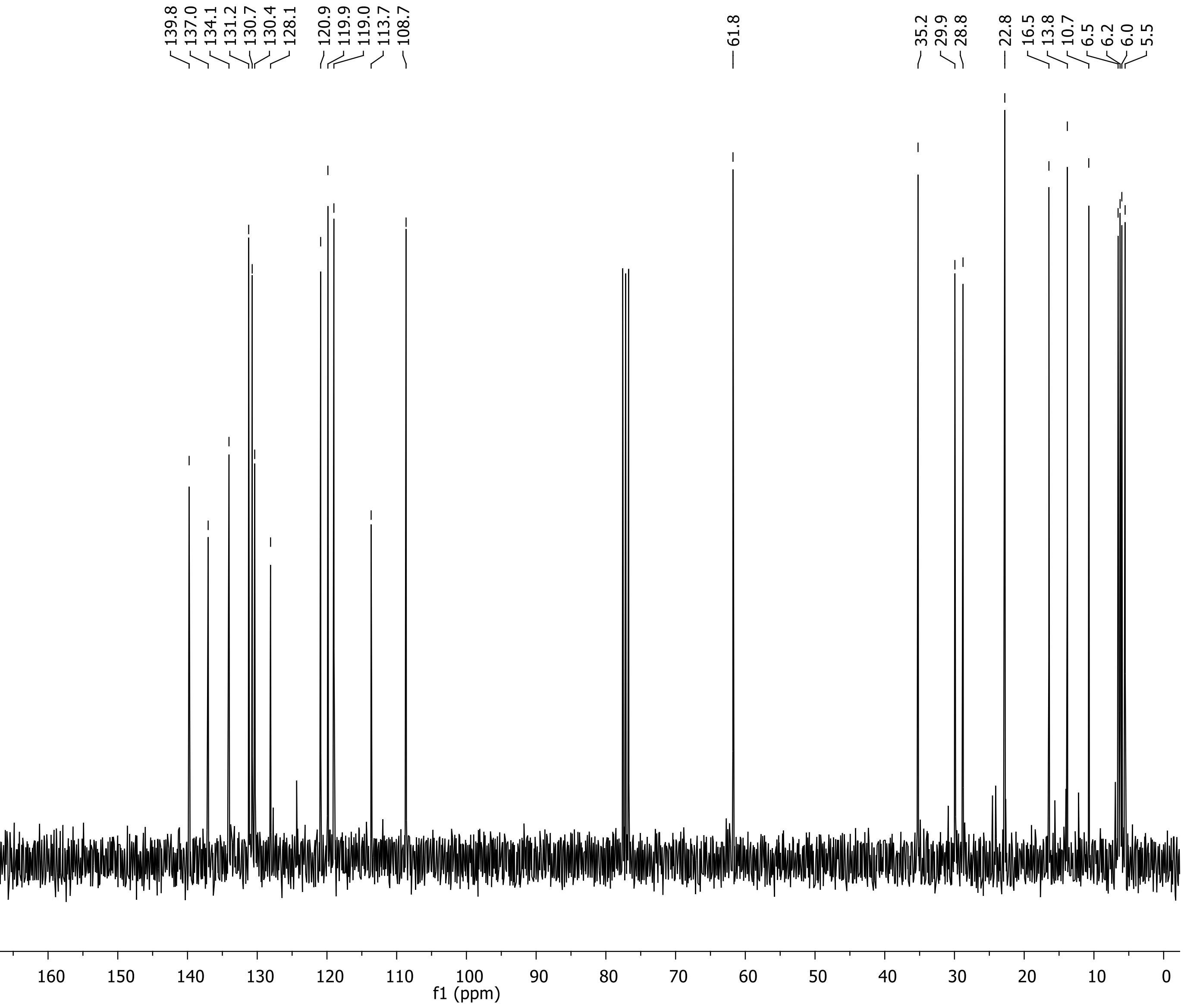

DEPT ($CDCl_3$, 75.4 MHz)

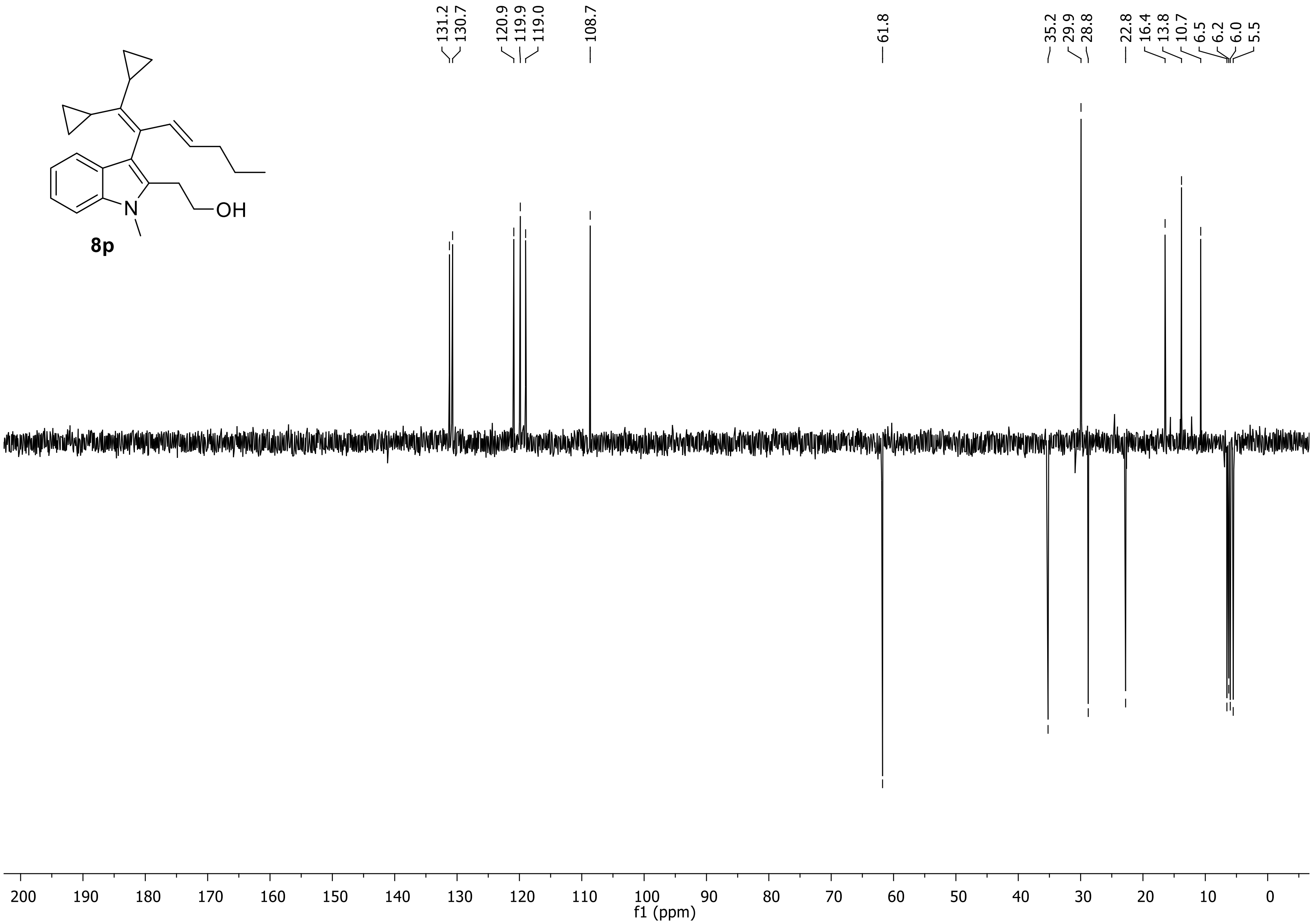

^1H NMR ($CDCl_3$, 300 MHz)

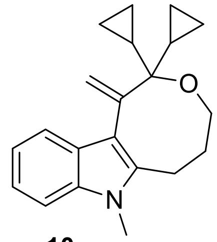

10a

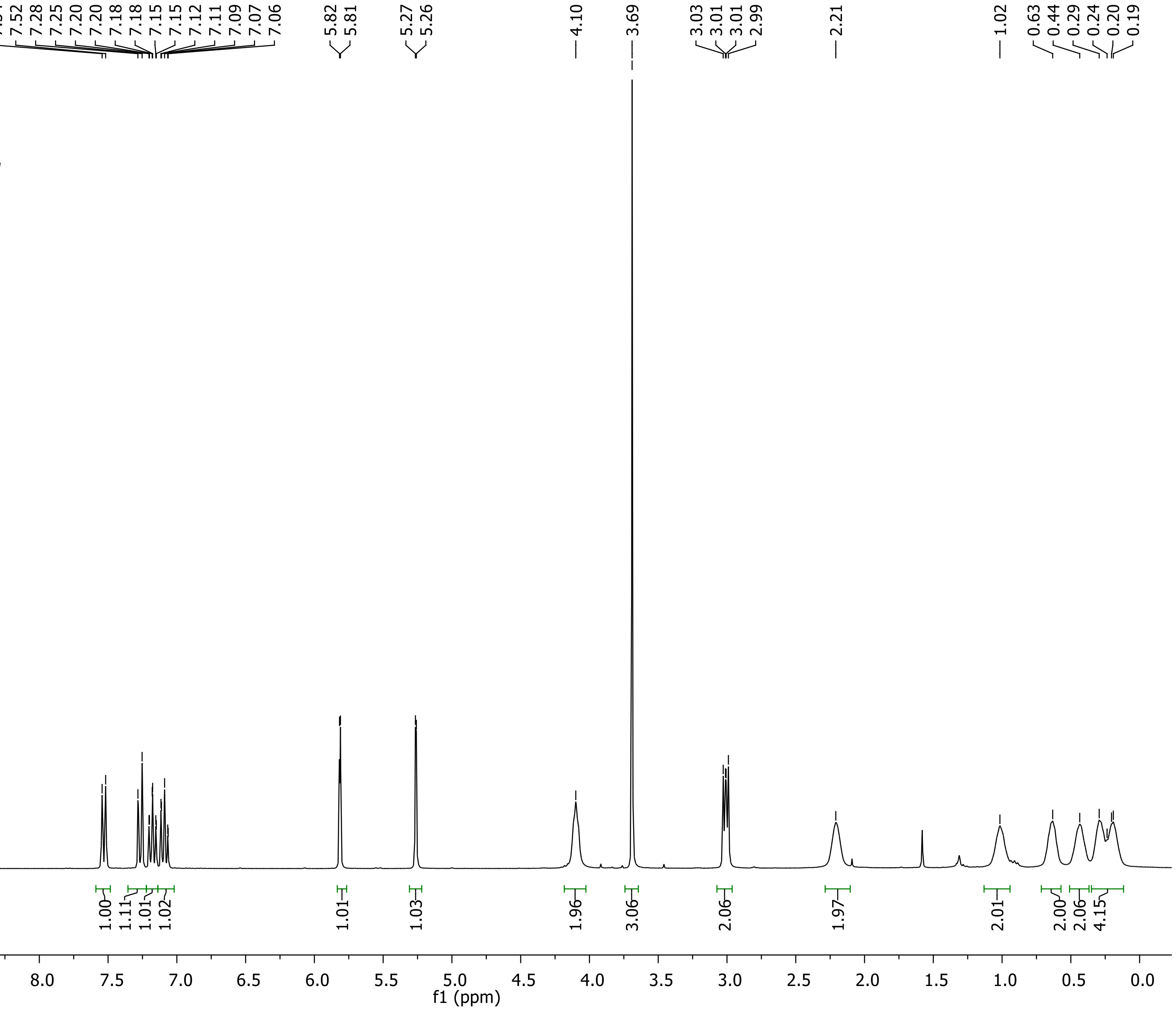

^{13}C NMR ($CDCl_3$, 75.4 MHz)

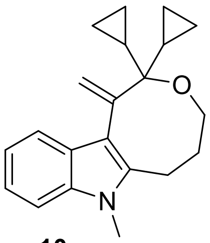

10a

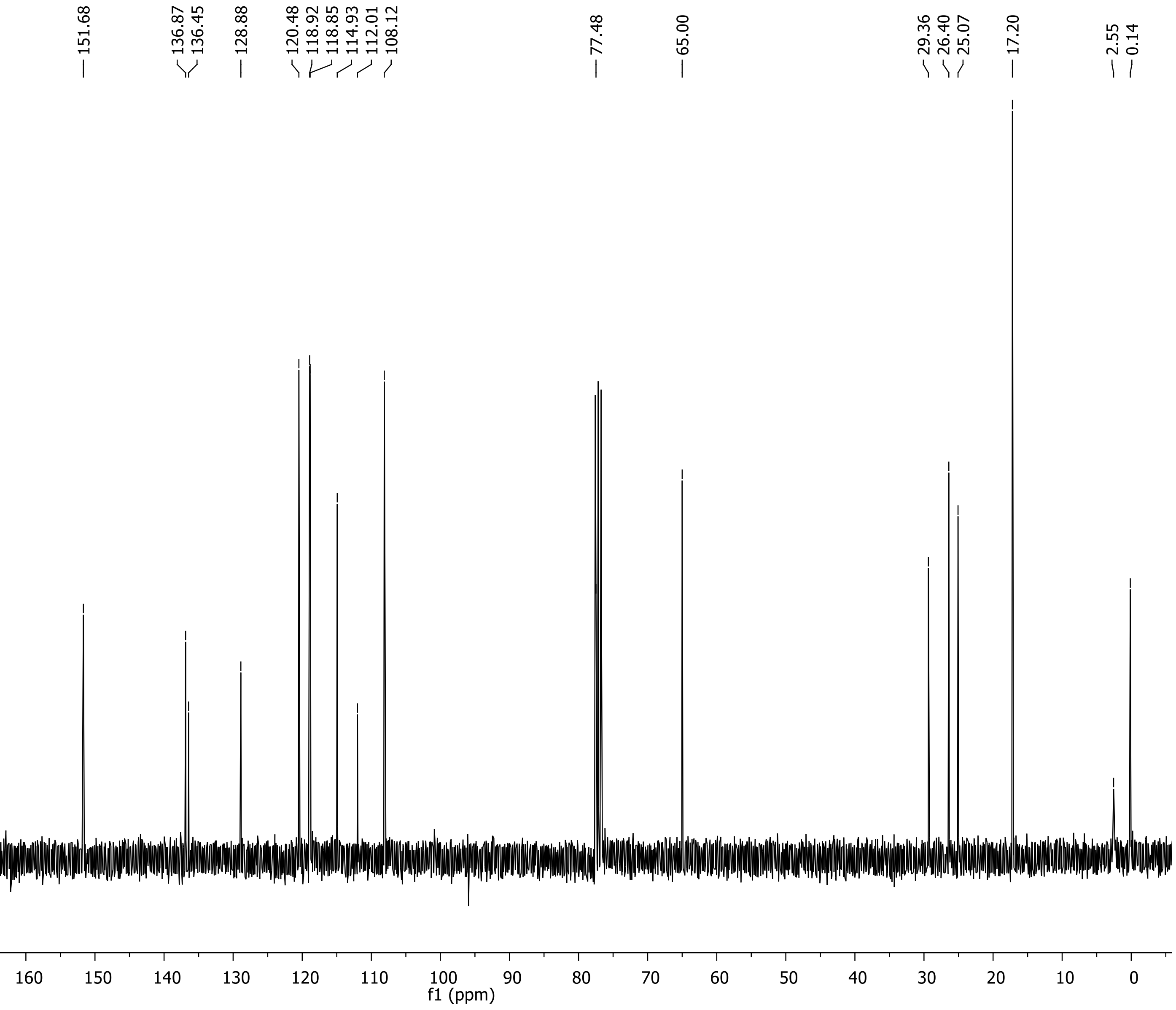

DEPT ($CDCl_3$, 75.4 MHz)

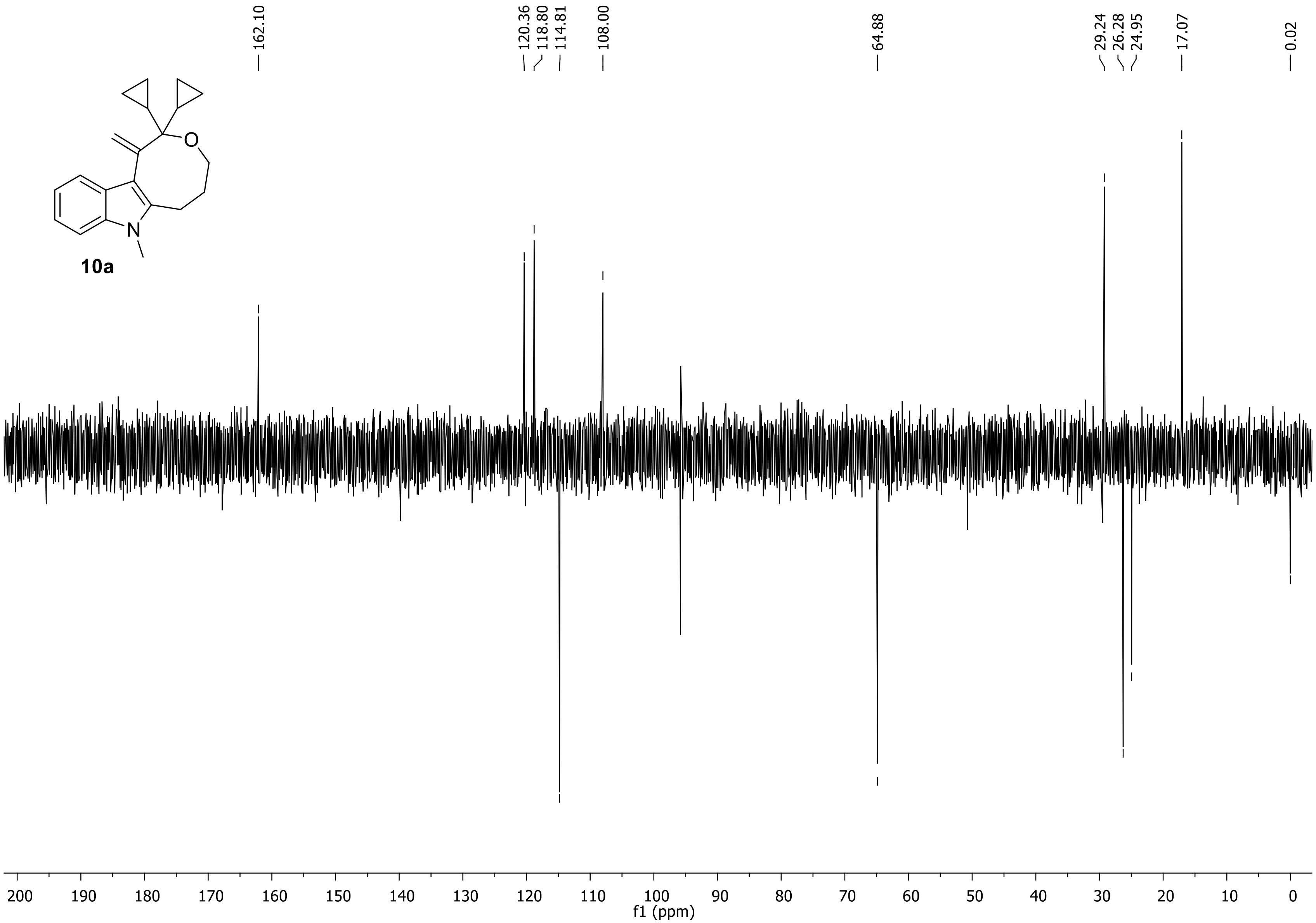

^{1}H NMR (DMSO-d6, 500 MHz, 100 °C)

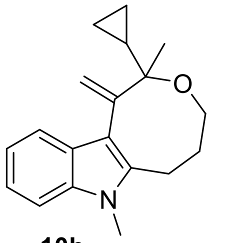

10b

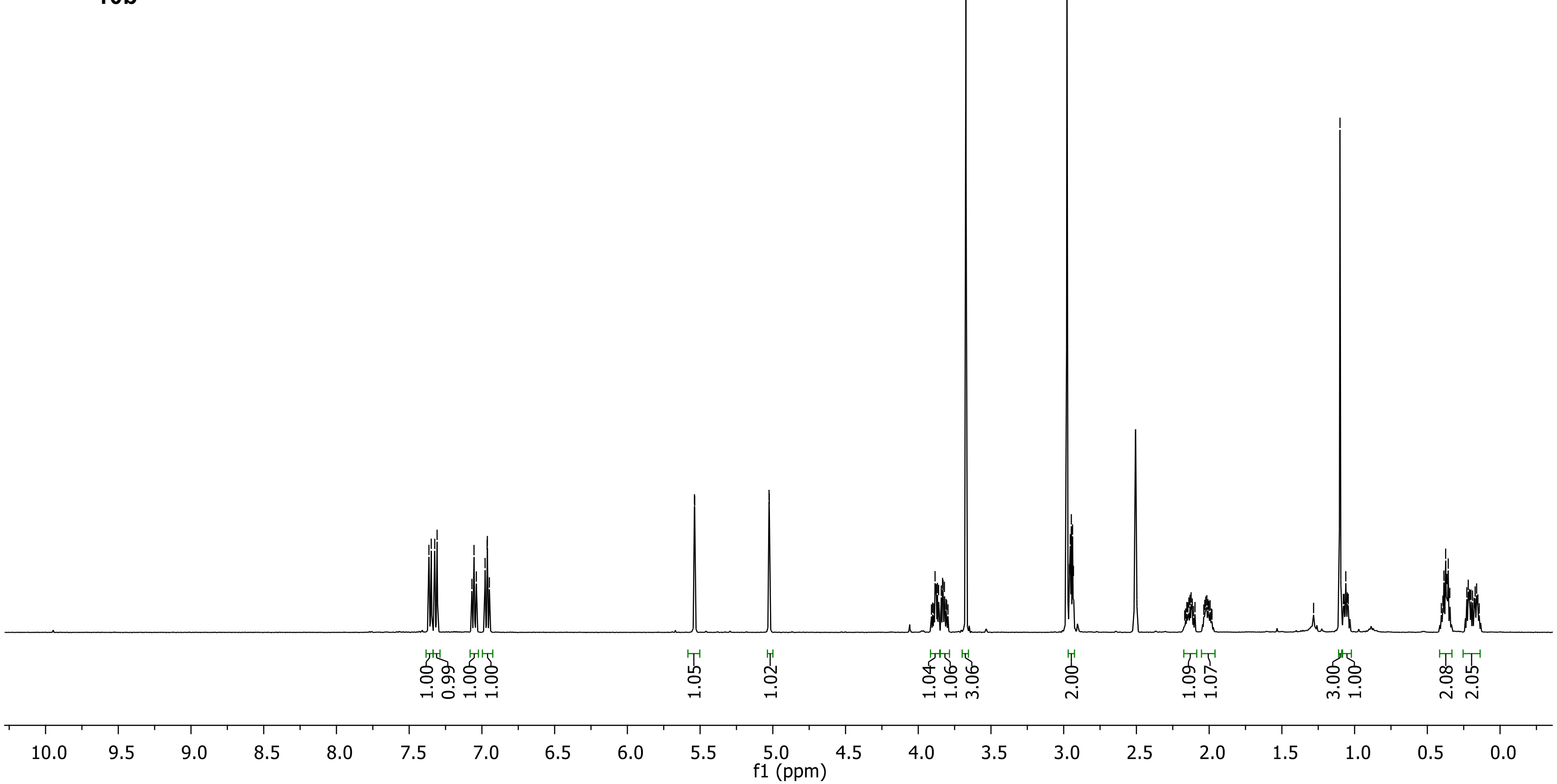

^{13}C NMR (DMSO-d6, 125.7 MHz, 100 °C)

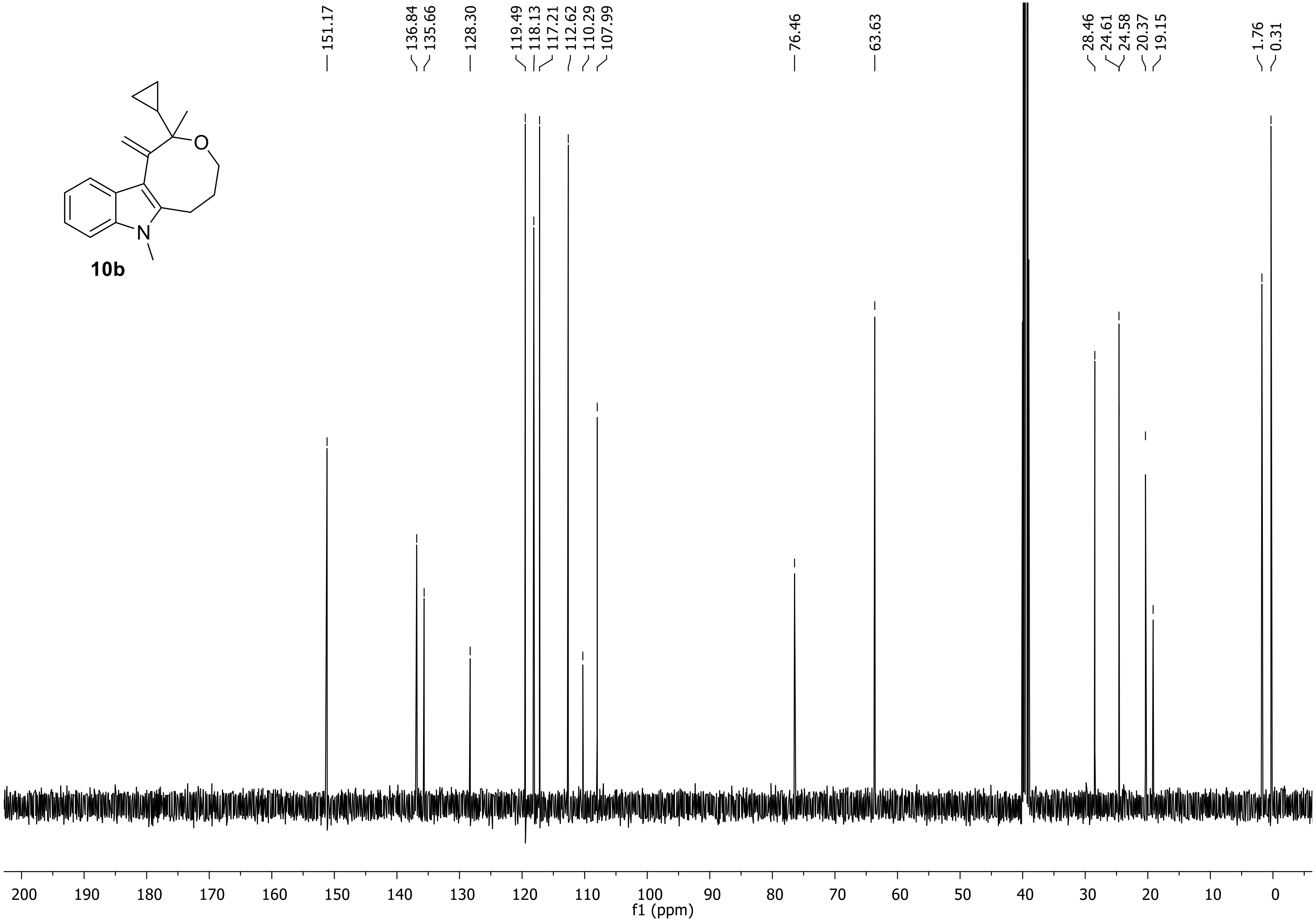

10b

DEPT (DMSO-d6, 125.7 MHz, 100 °C)

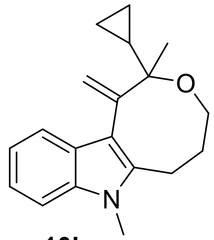

10b

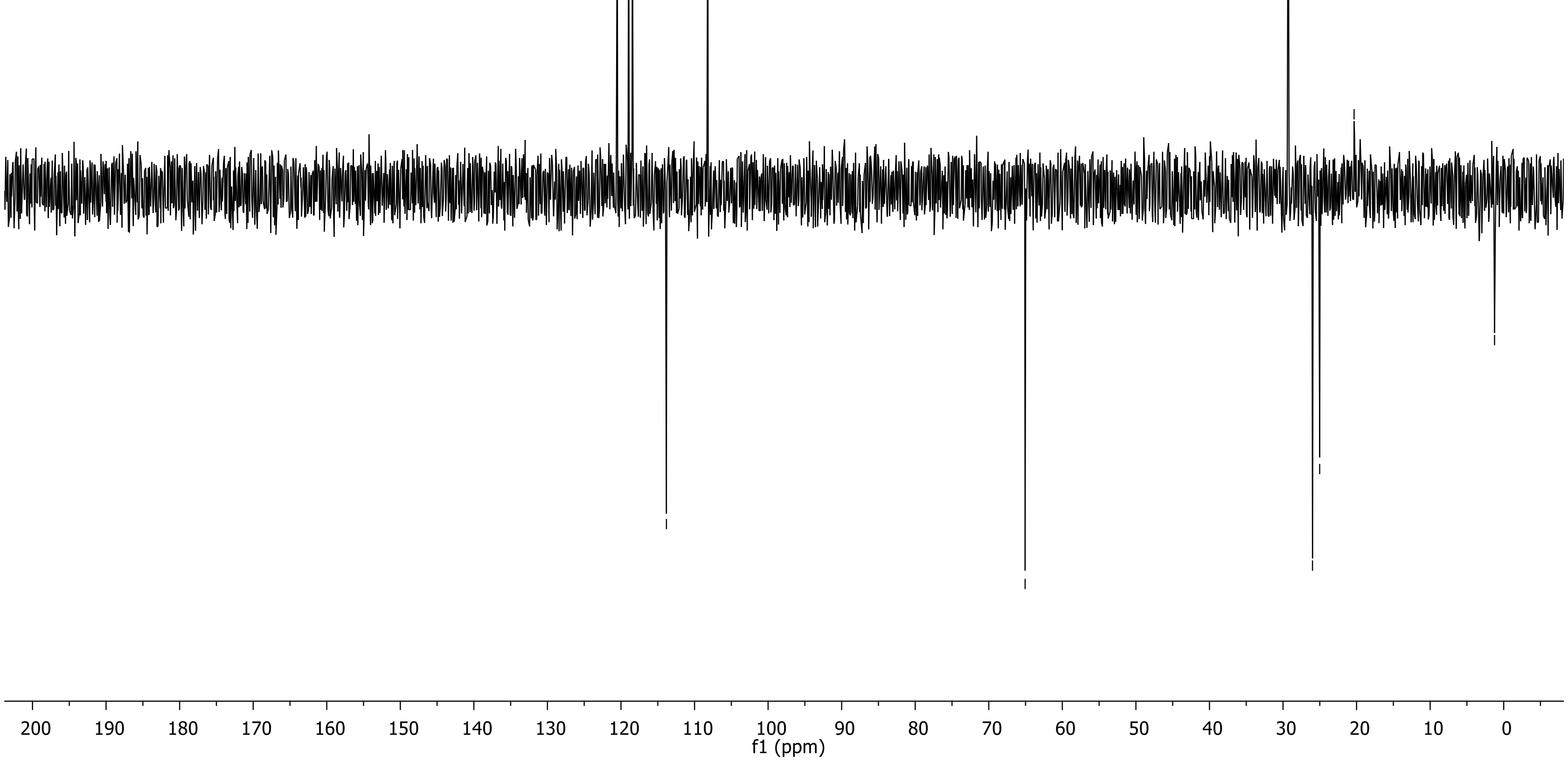

^1H NMR ($CDCl_3$, 300 MHz)

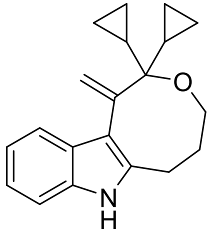

10c

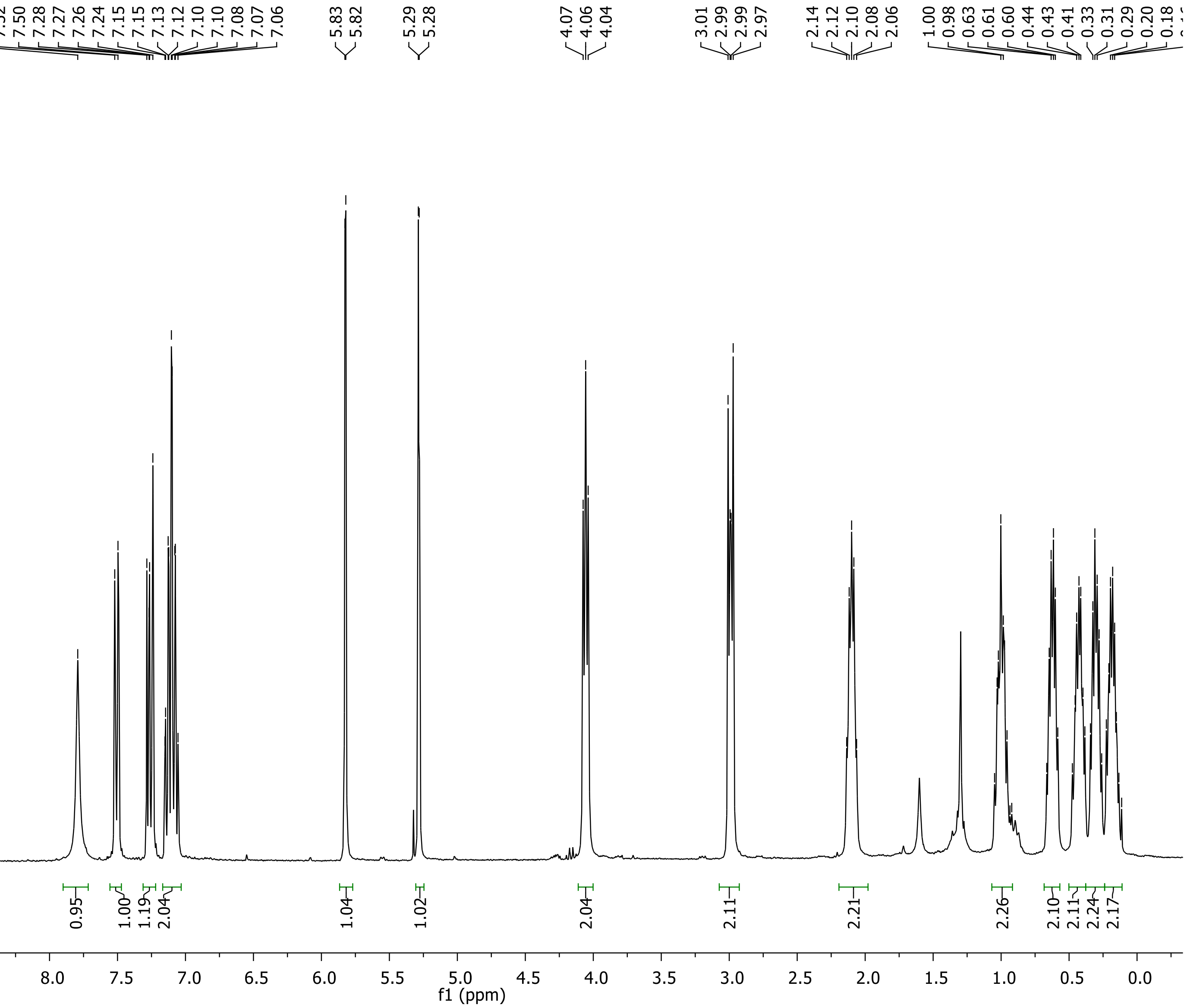

^{13}C NMR ($CDCl_3$, 75.4 MHz)

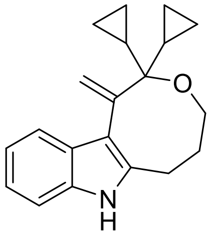

10c

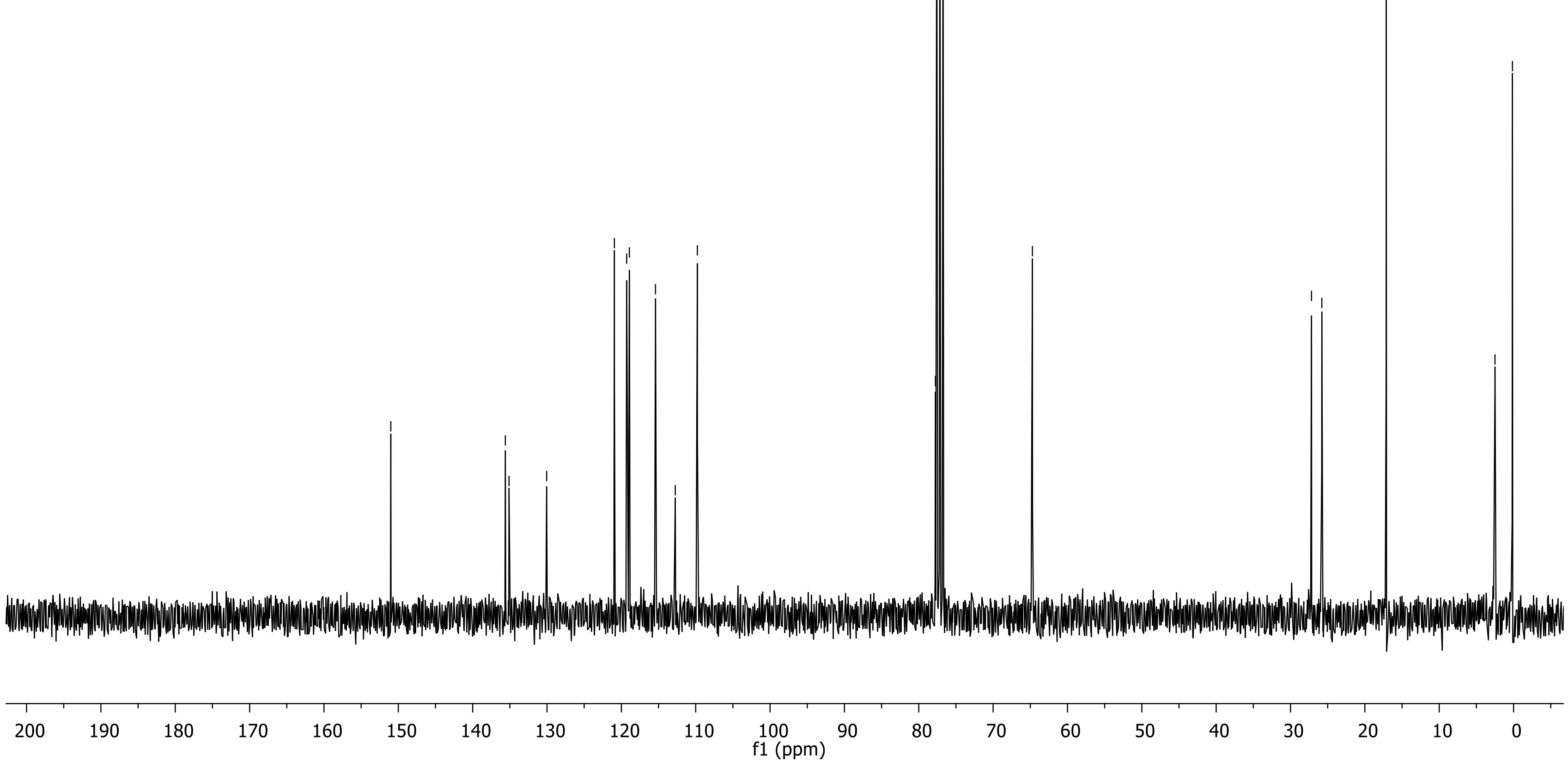

DEPT ($CDCl_3$, 75.4 MHz)

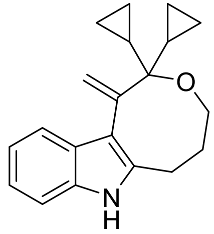

10c

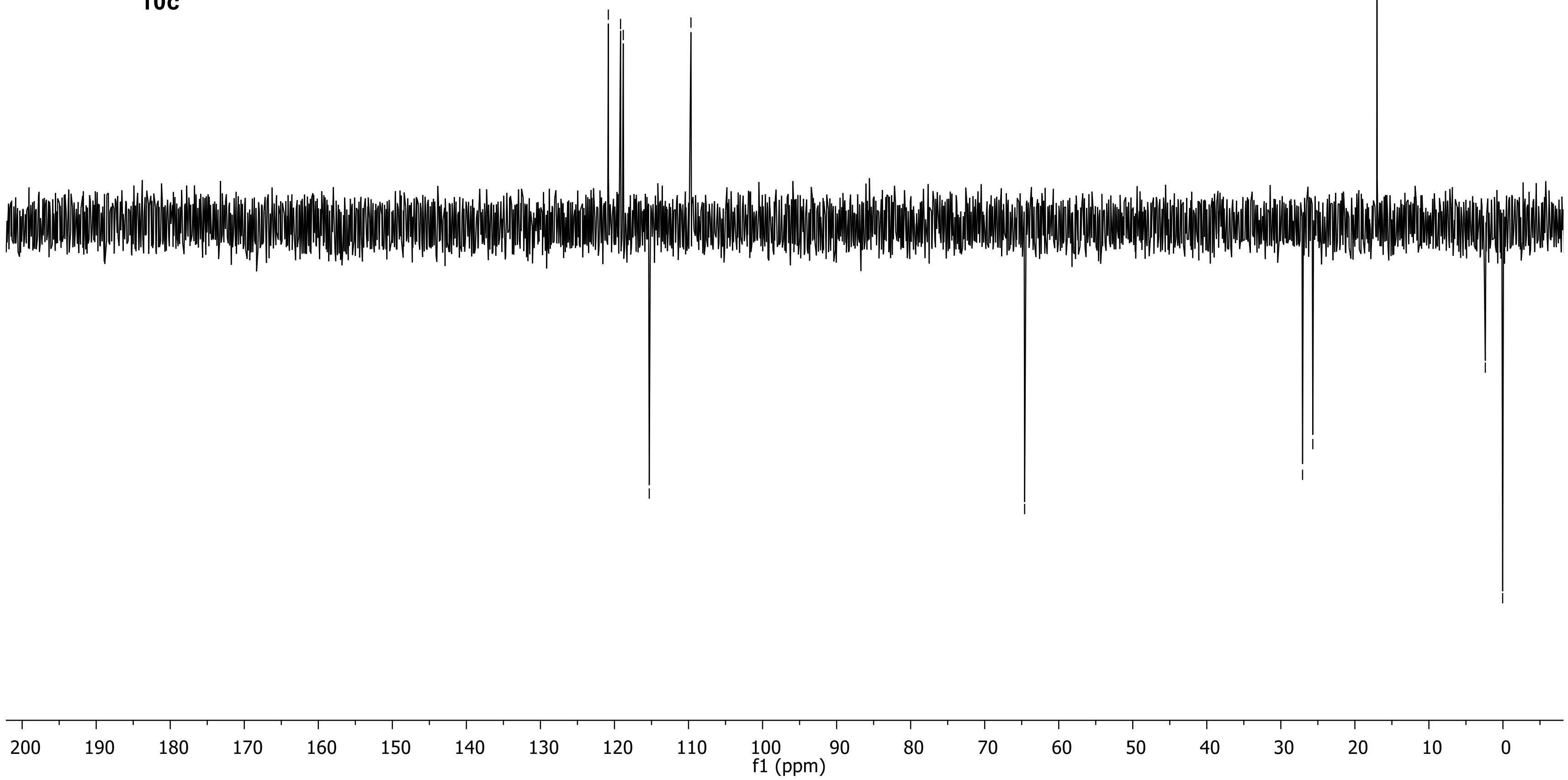

^{1}H NMR ($CDCl_3$, 300 MHz)

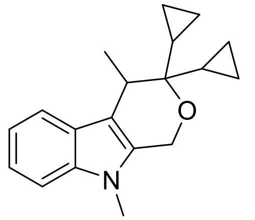

11a

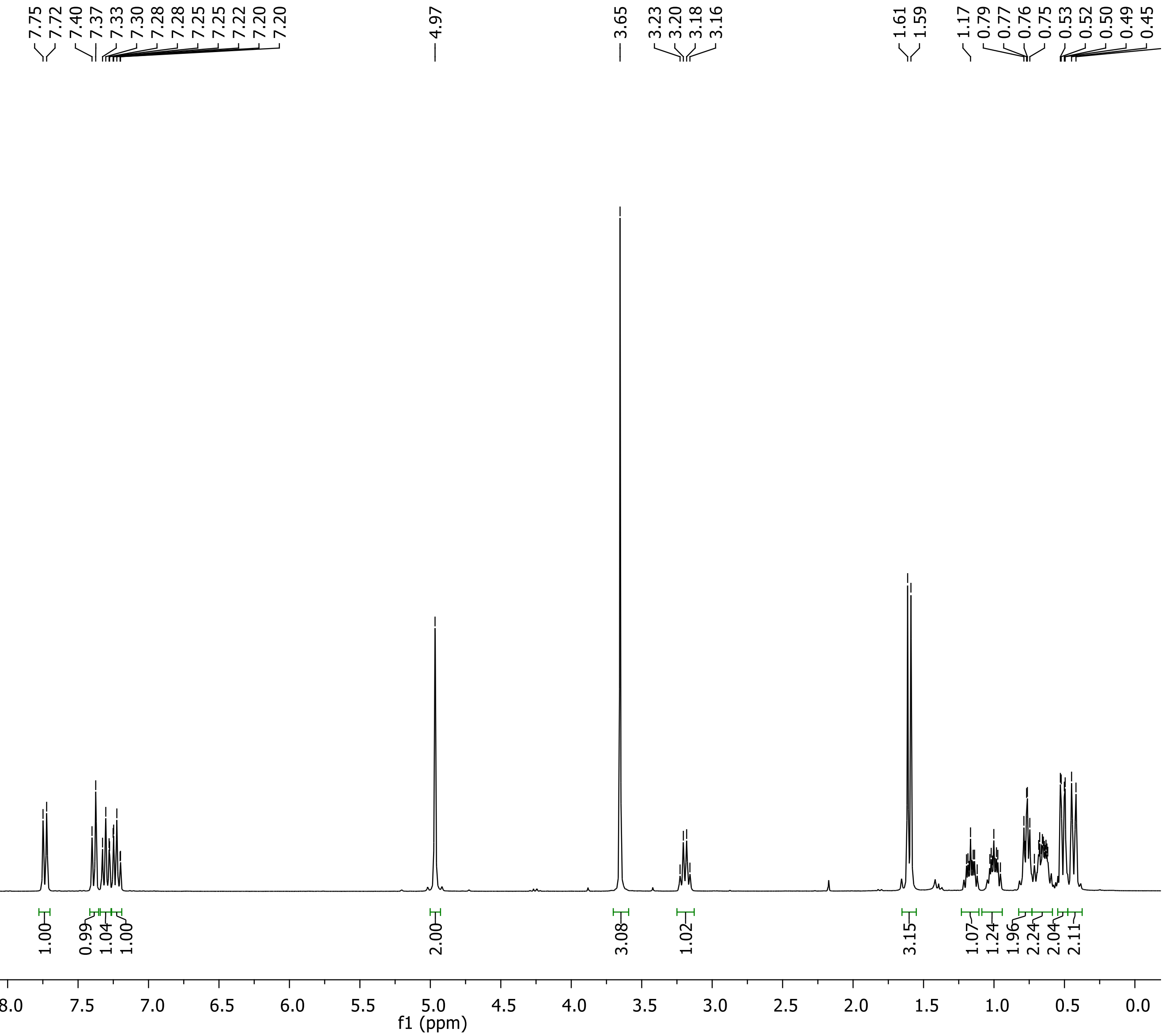

^{13}C NMR ($CDCl_3$, 75.4 MHz)

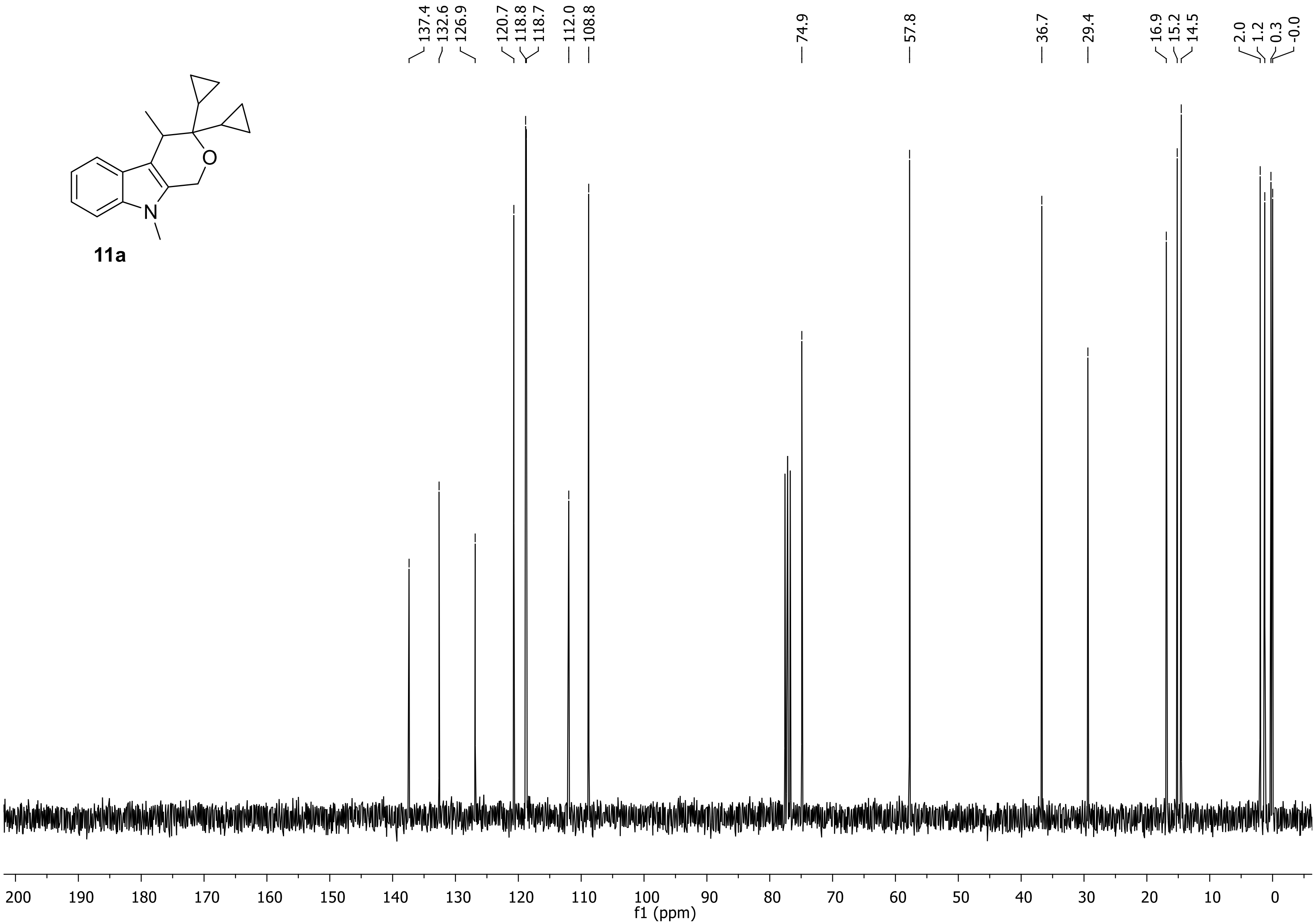

DEPT ($CDCl_3$, 75.4 MHz)

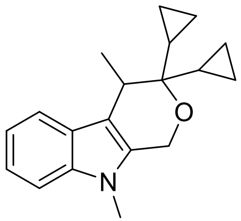

11a

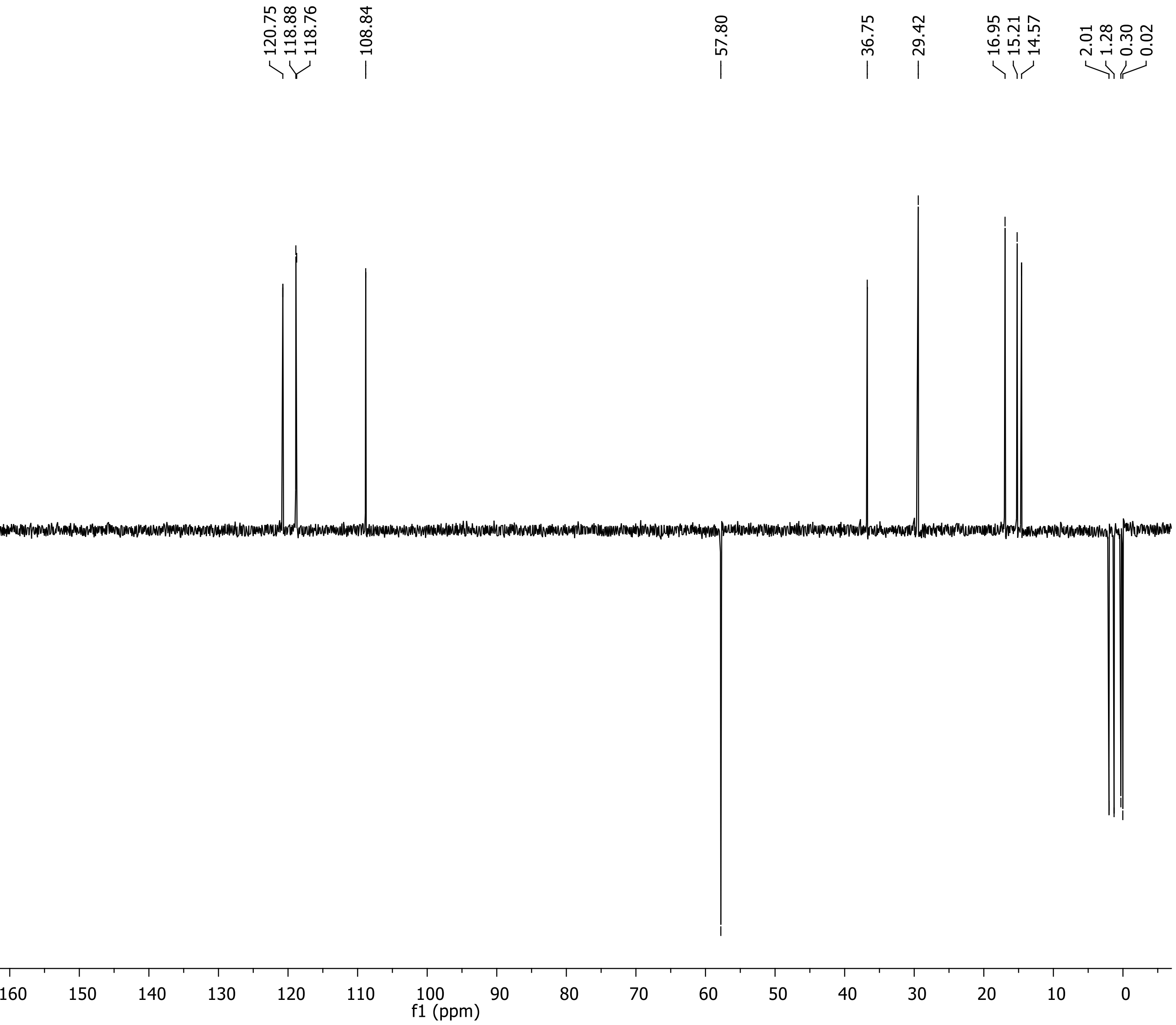

^{1}H NMR ($CDCl_3$, 300 MHz)

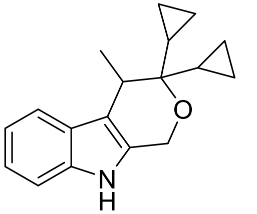

11b

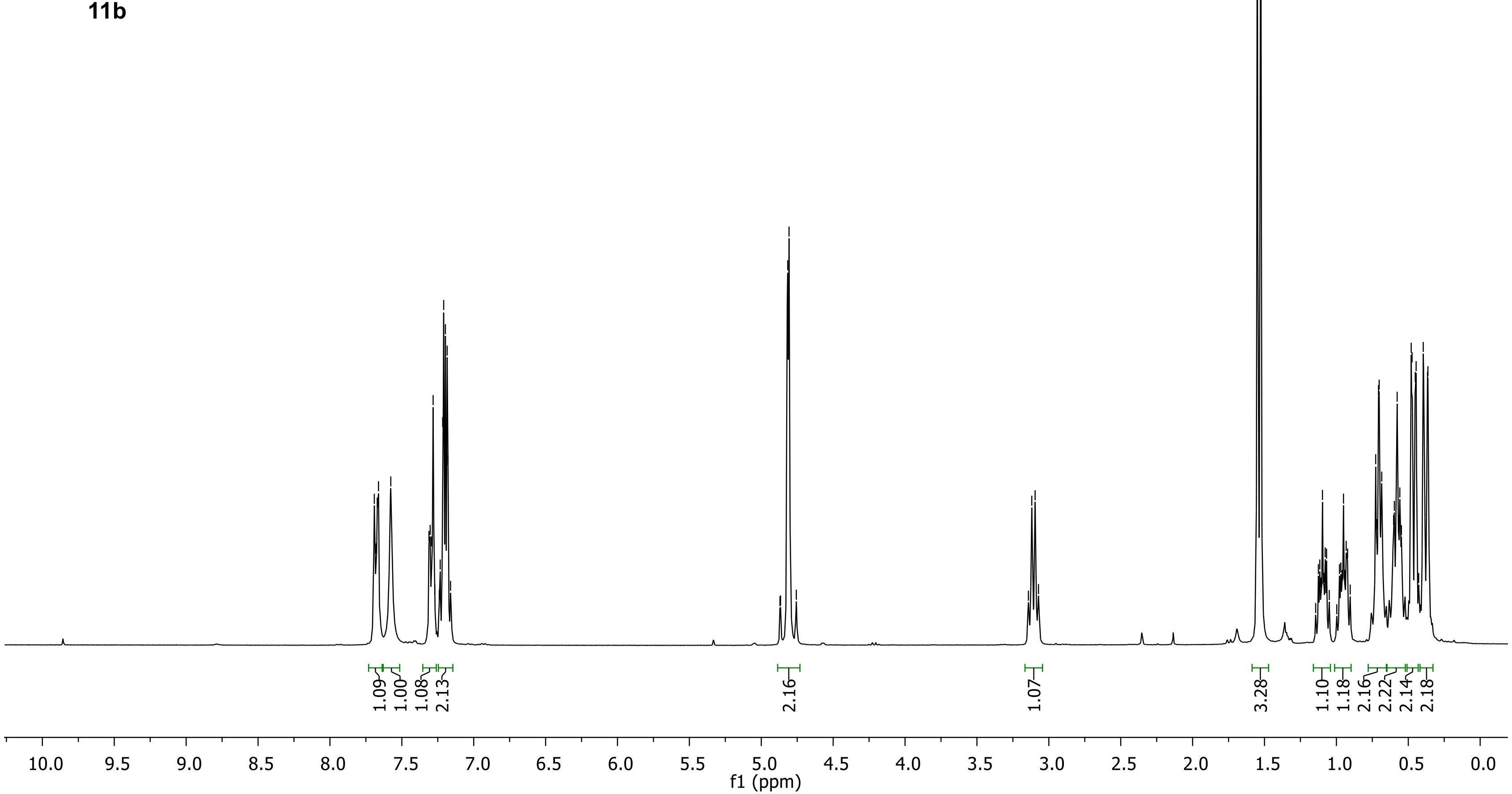

^{13}C NMR ($CDCl_3$, 75.4 MHz)

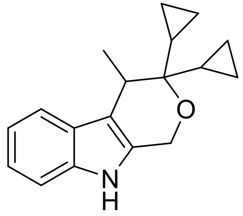

11b

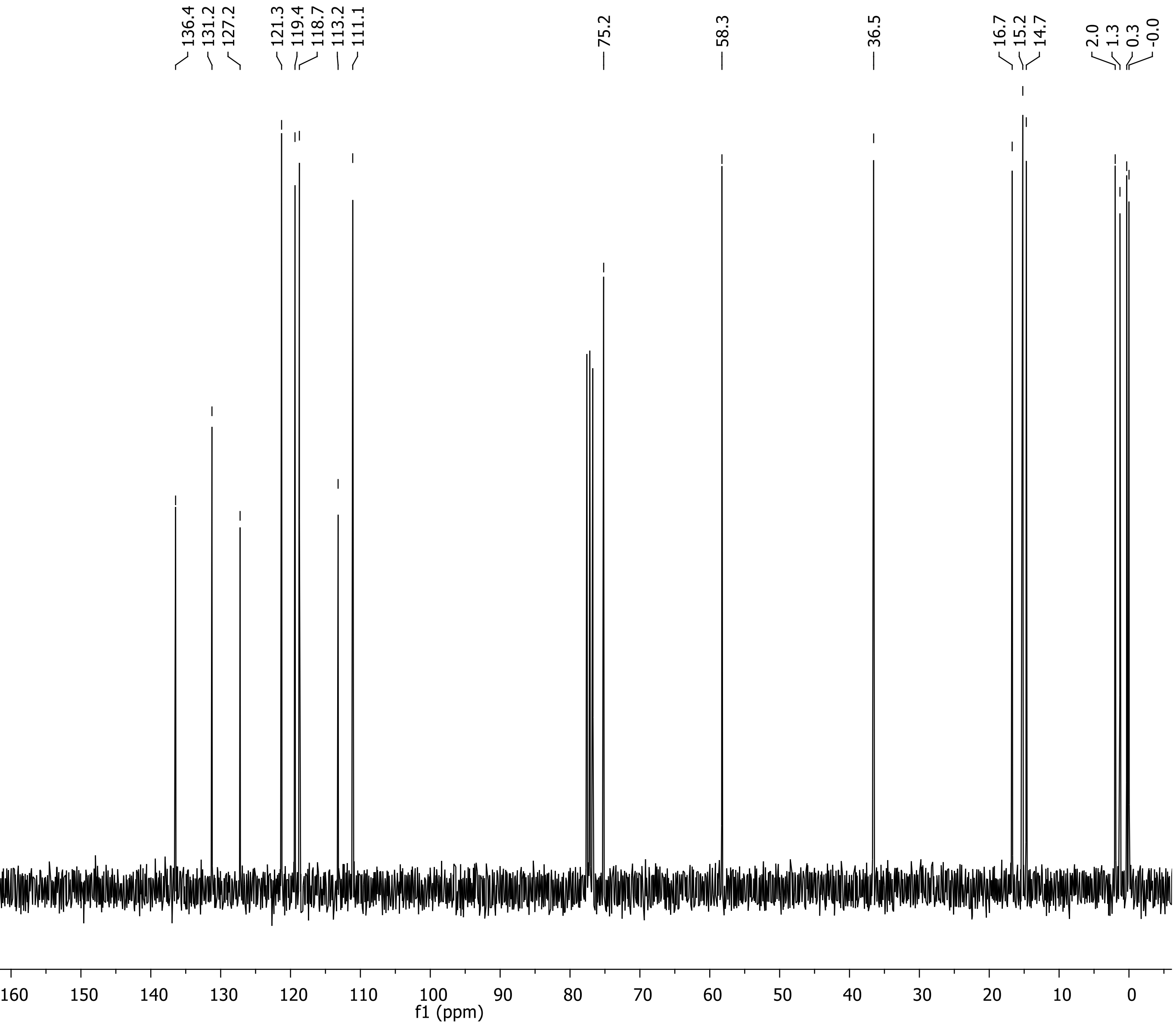

DEPT ($CDCl_3$, 75.4 MHz)

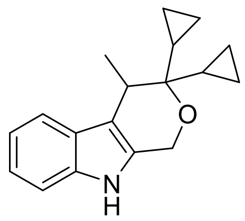

11b

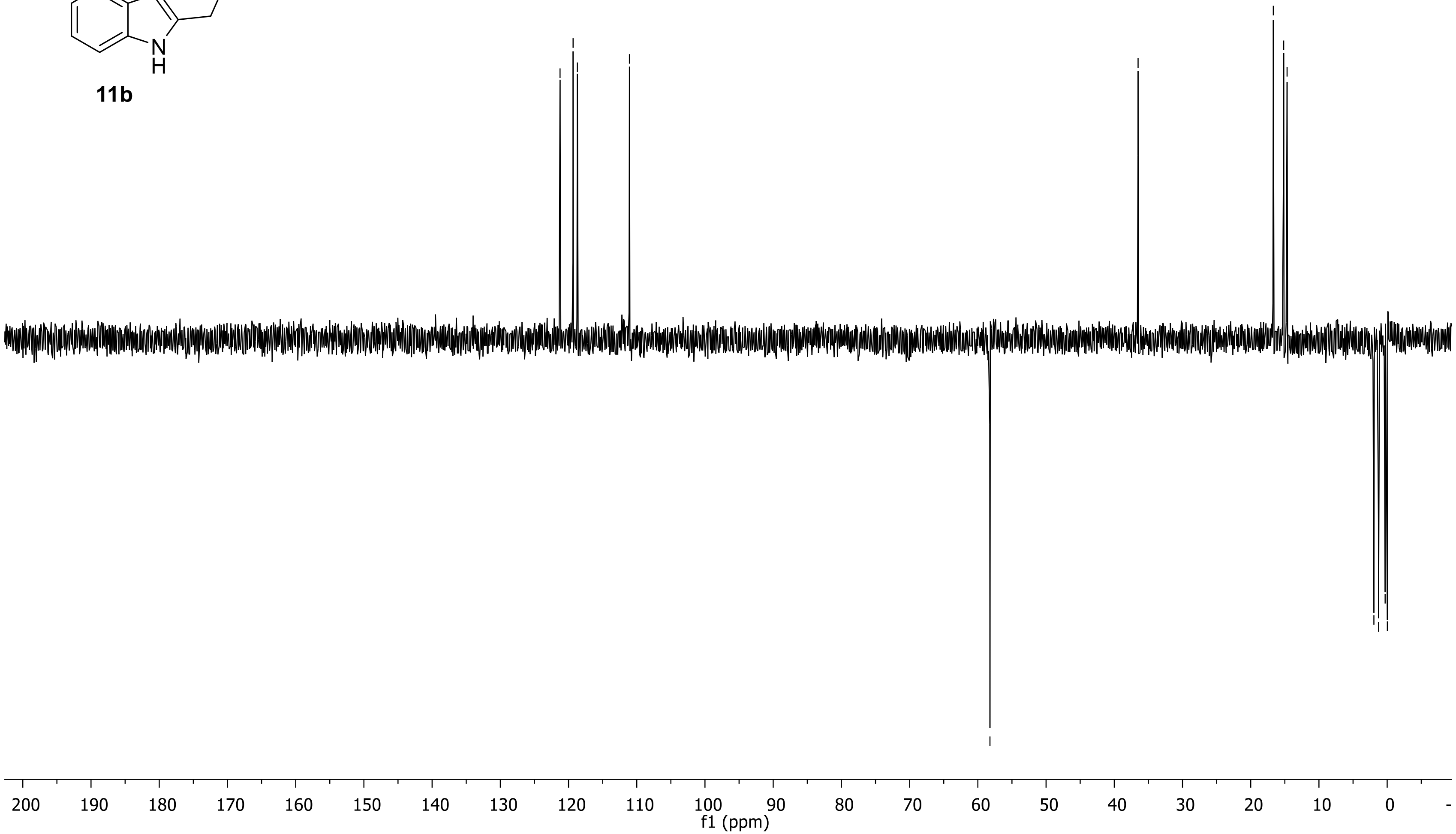

^{1}H NMR ($CDCl_3$, 500 MHz)

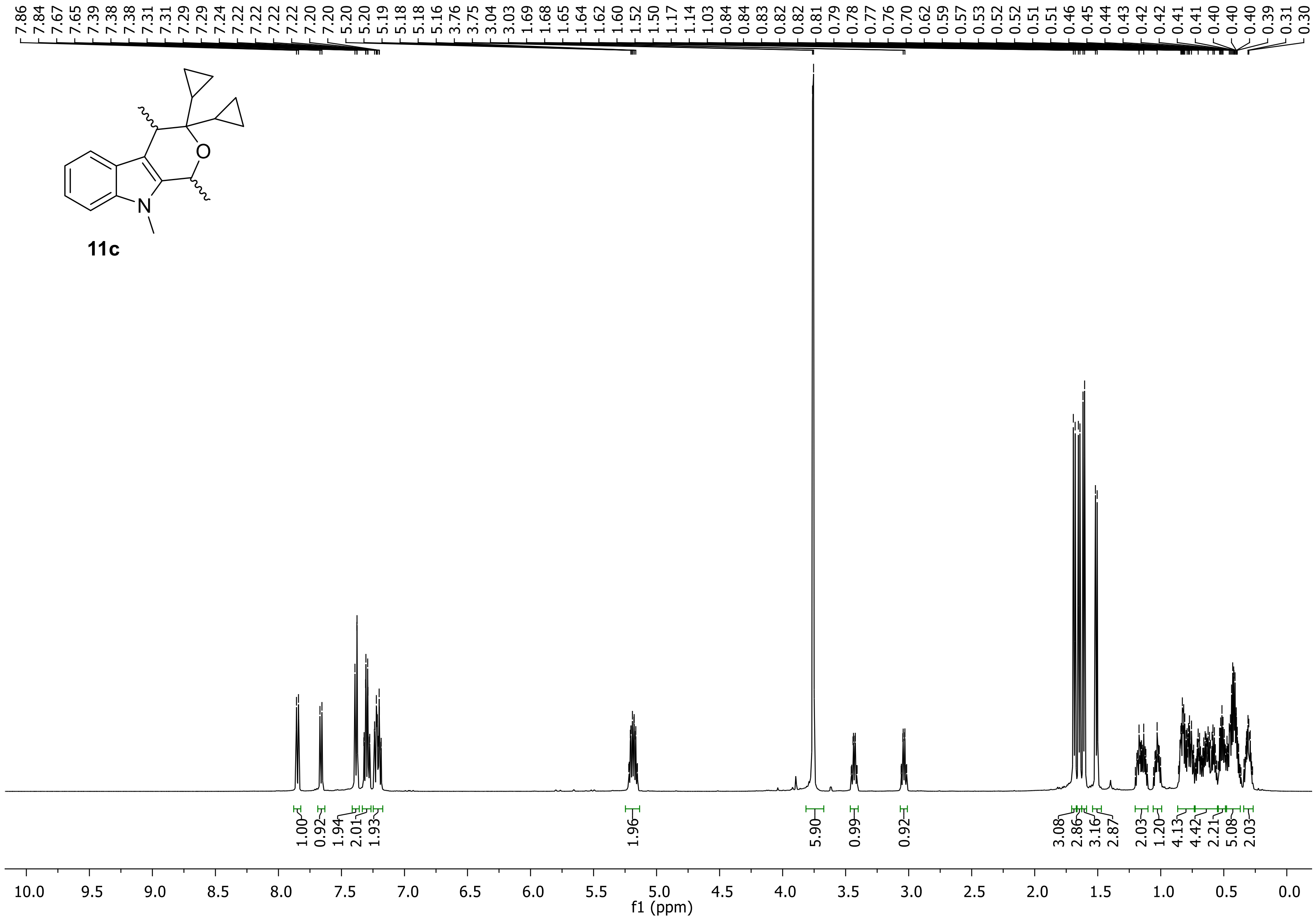

^{13}C NMR ($CDCl_3$, 125.7 MHz)

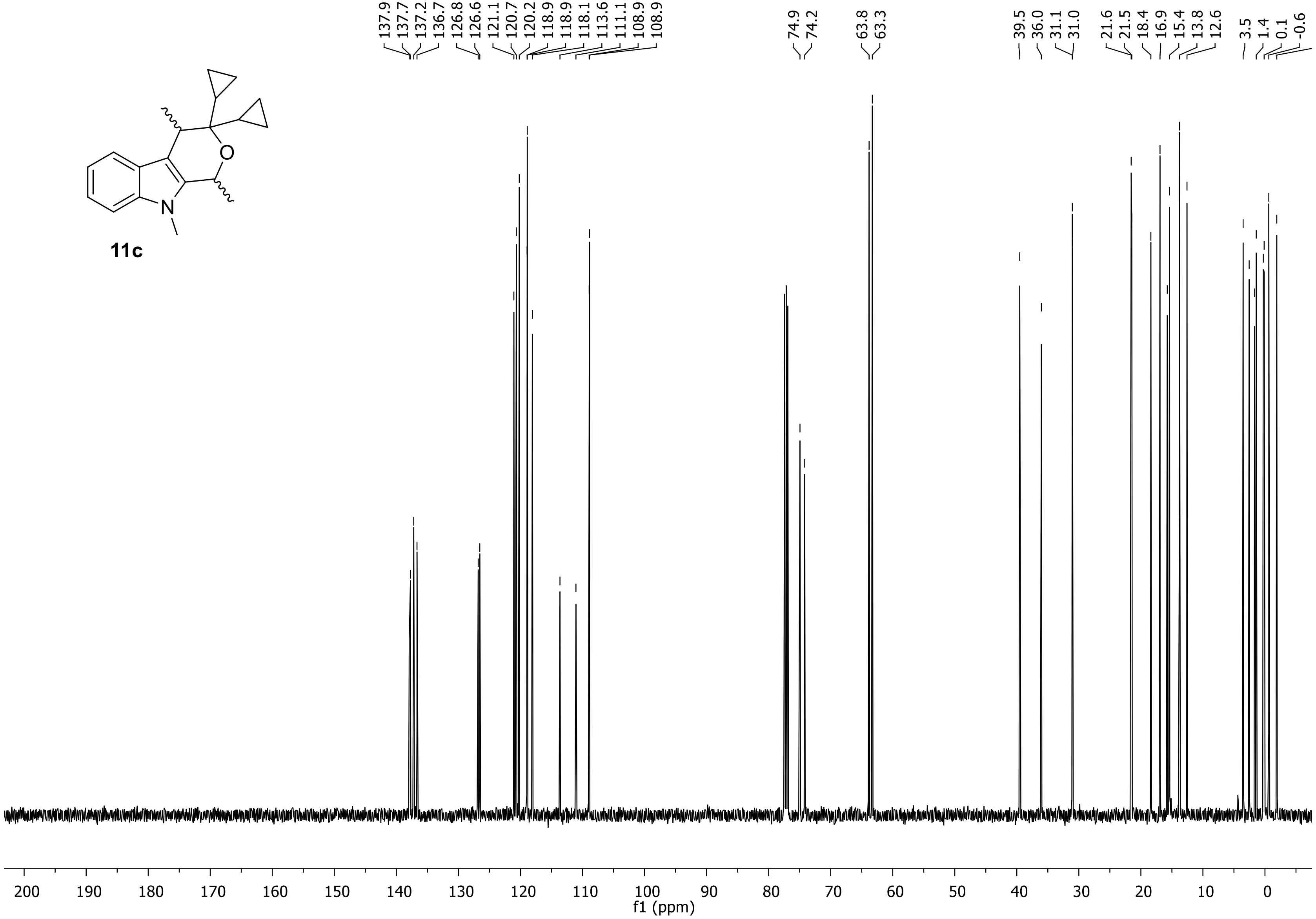

11c

DEPT ($CDCl_3$, 125.7 MHz)

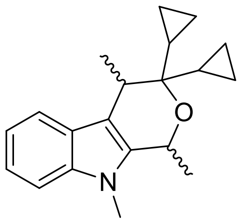

11c

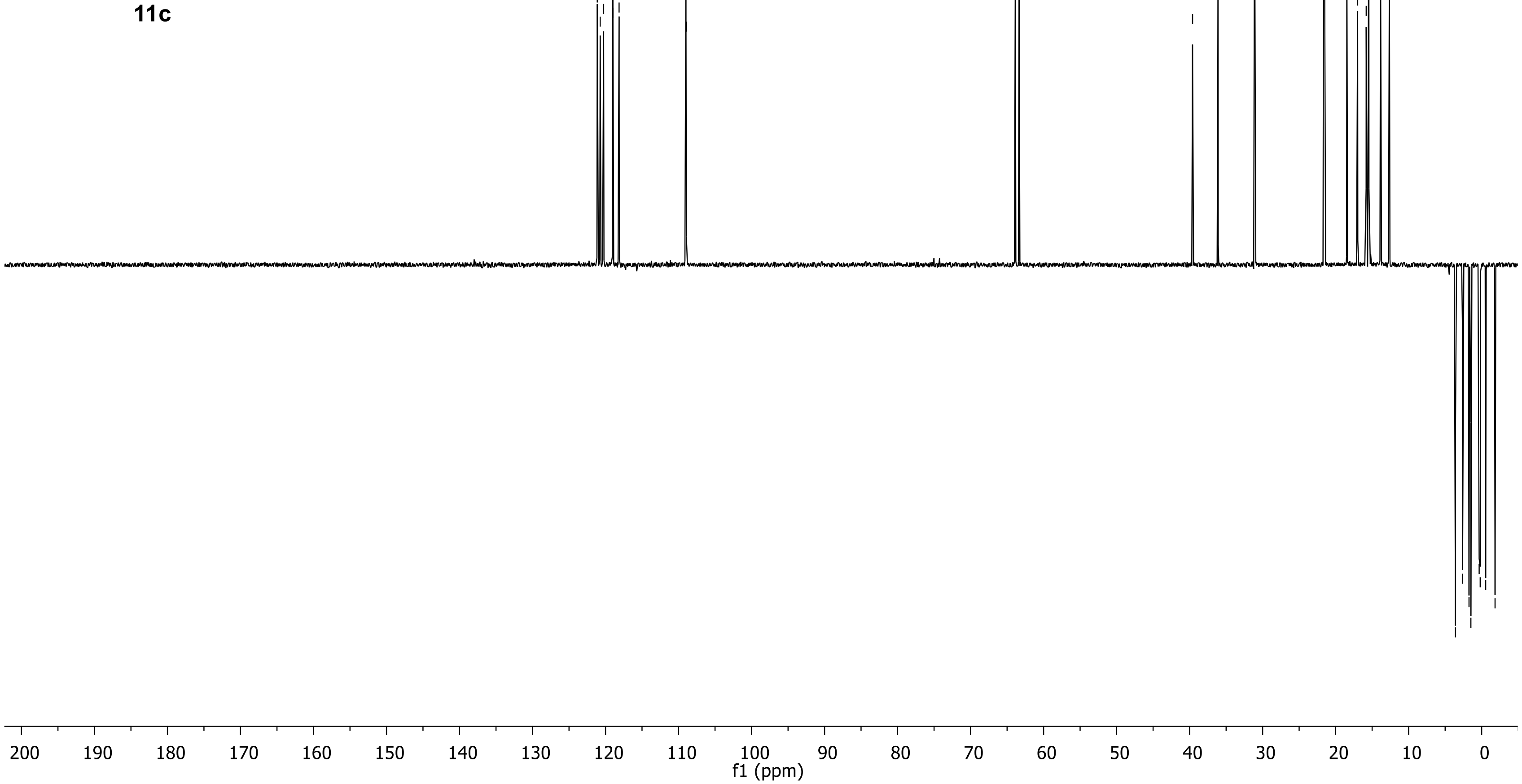

^{1}H NMR ($CDCl_3$, 300 MHz)

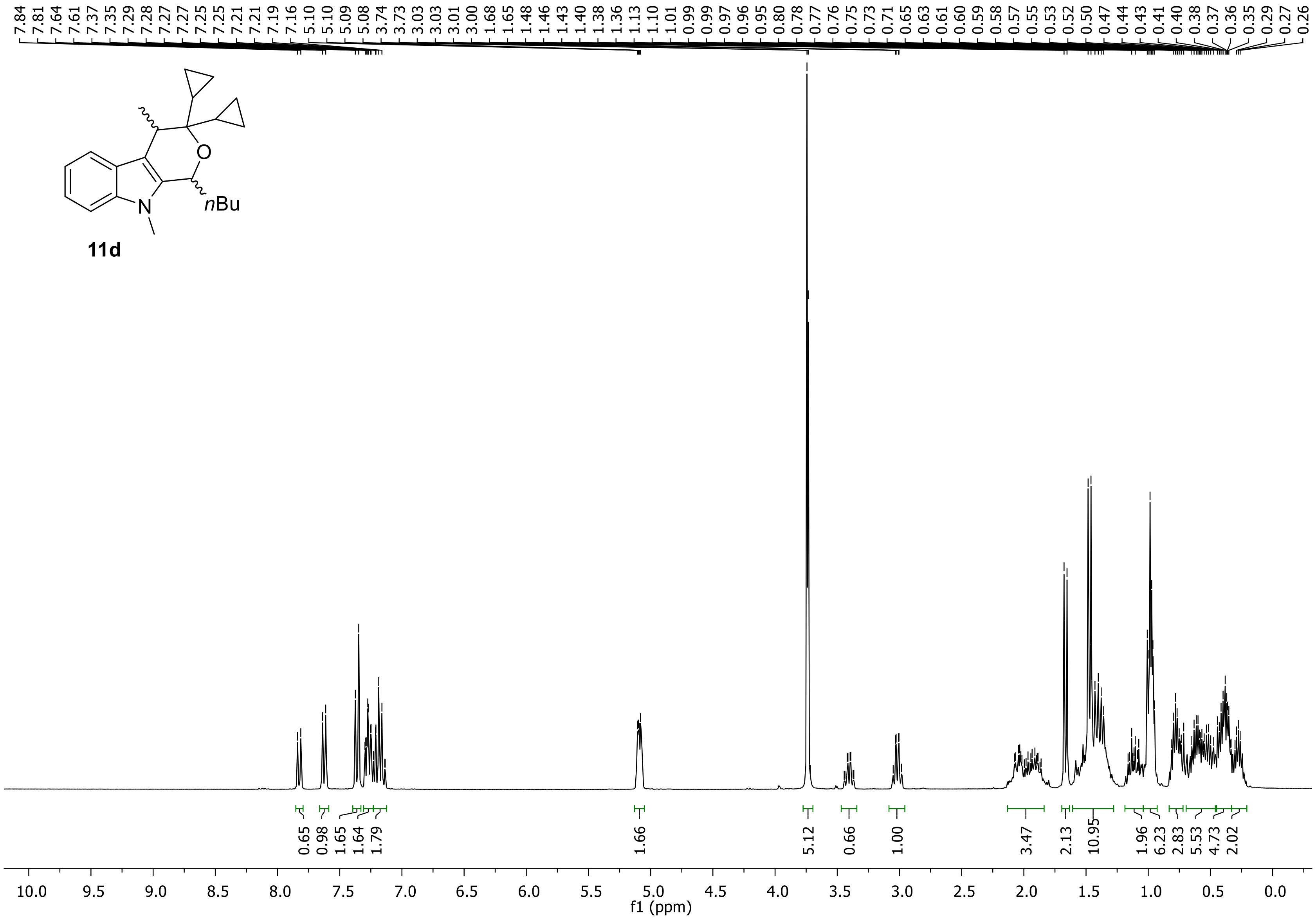

^{13}C NMR ($CDCl_3$, 75.4 MHz)

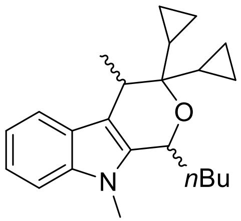

11d

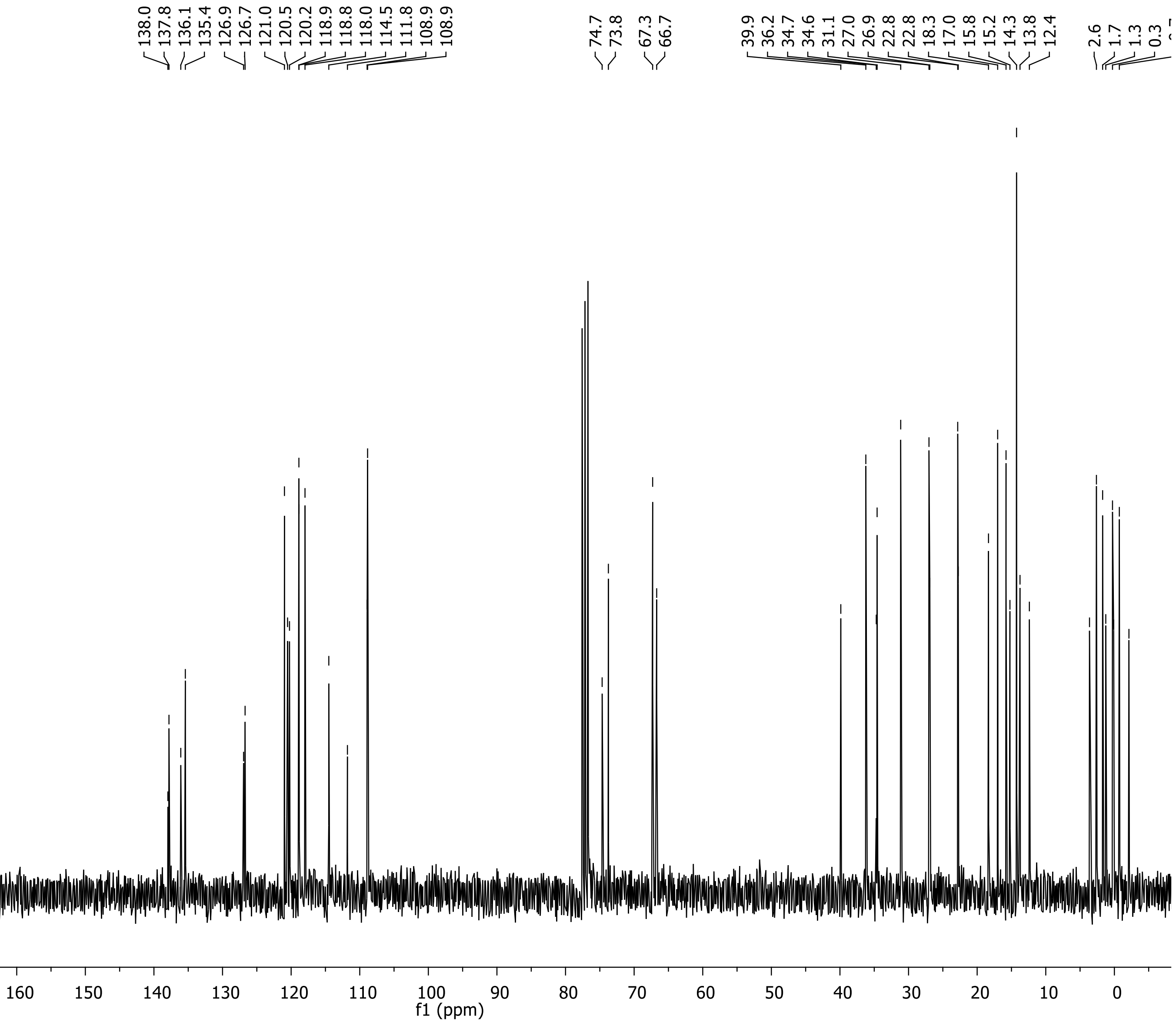

DEPT ($CDCl_3$, 75.4 MHz)

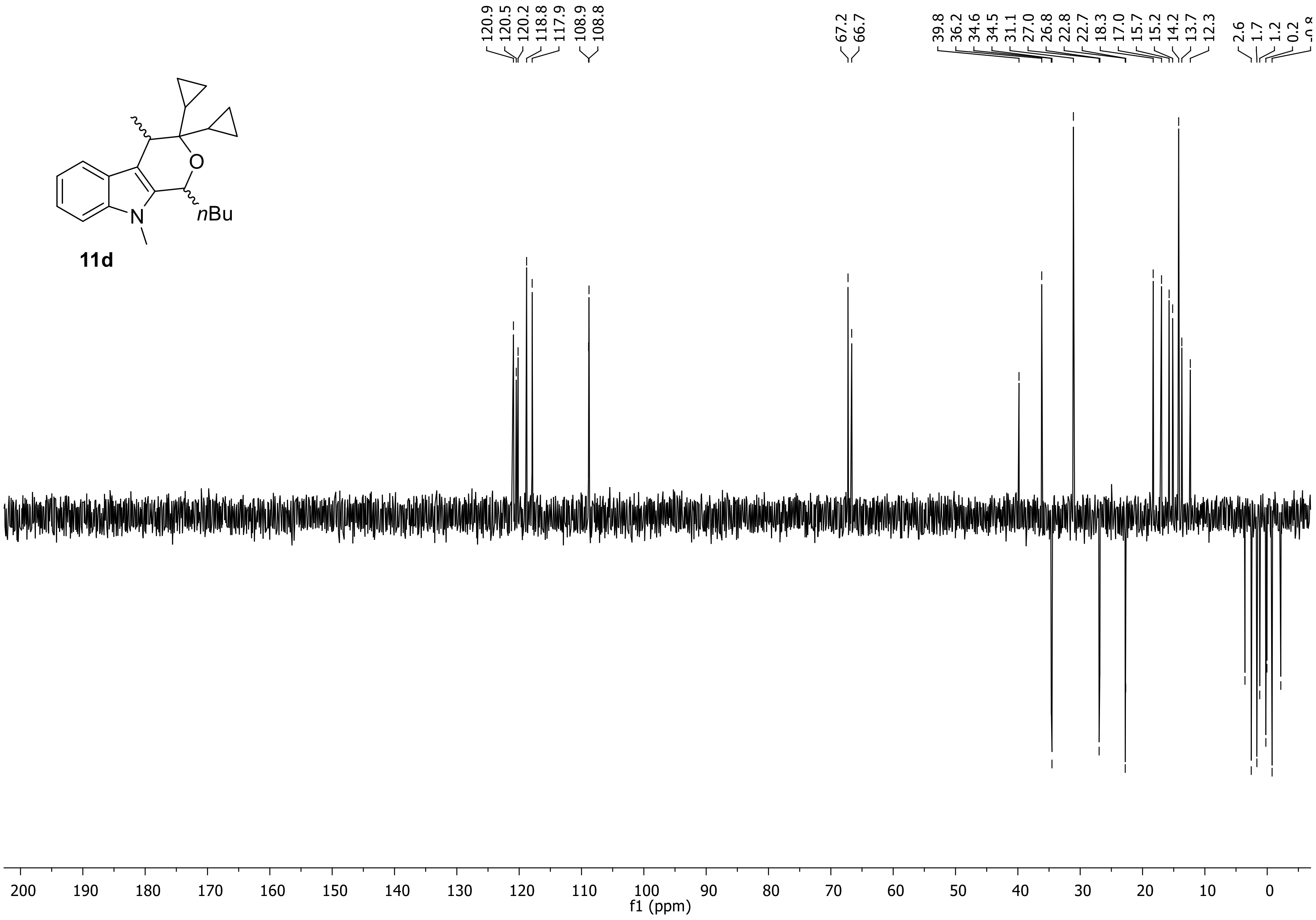

^{1}H NMR ($CDCl_3$, 500 MHz)

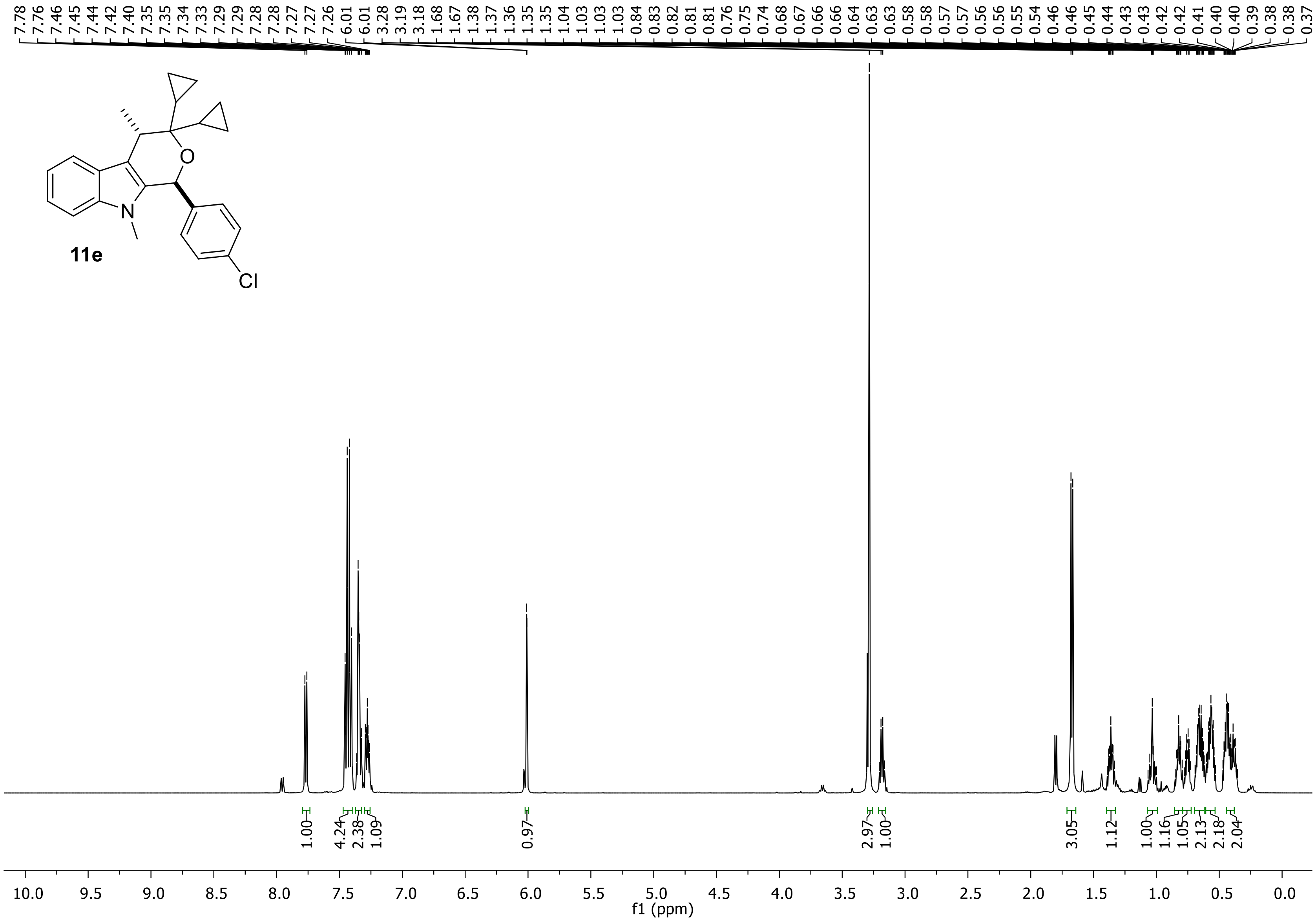

11e

^{13}C NMR ($CDCl_3$, 125.7 MHz)

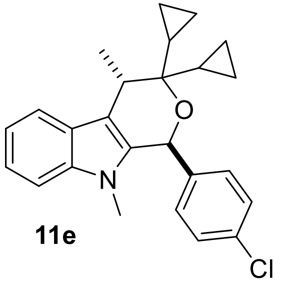

11e

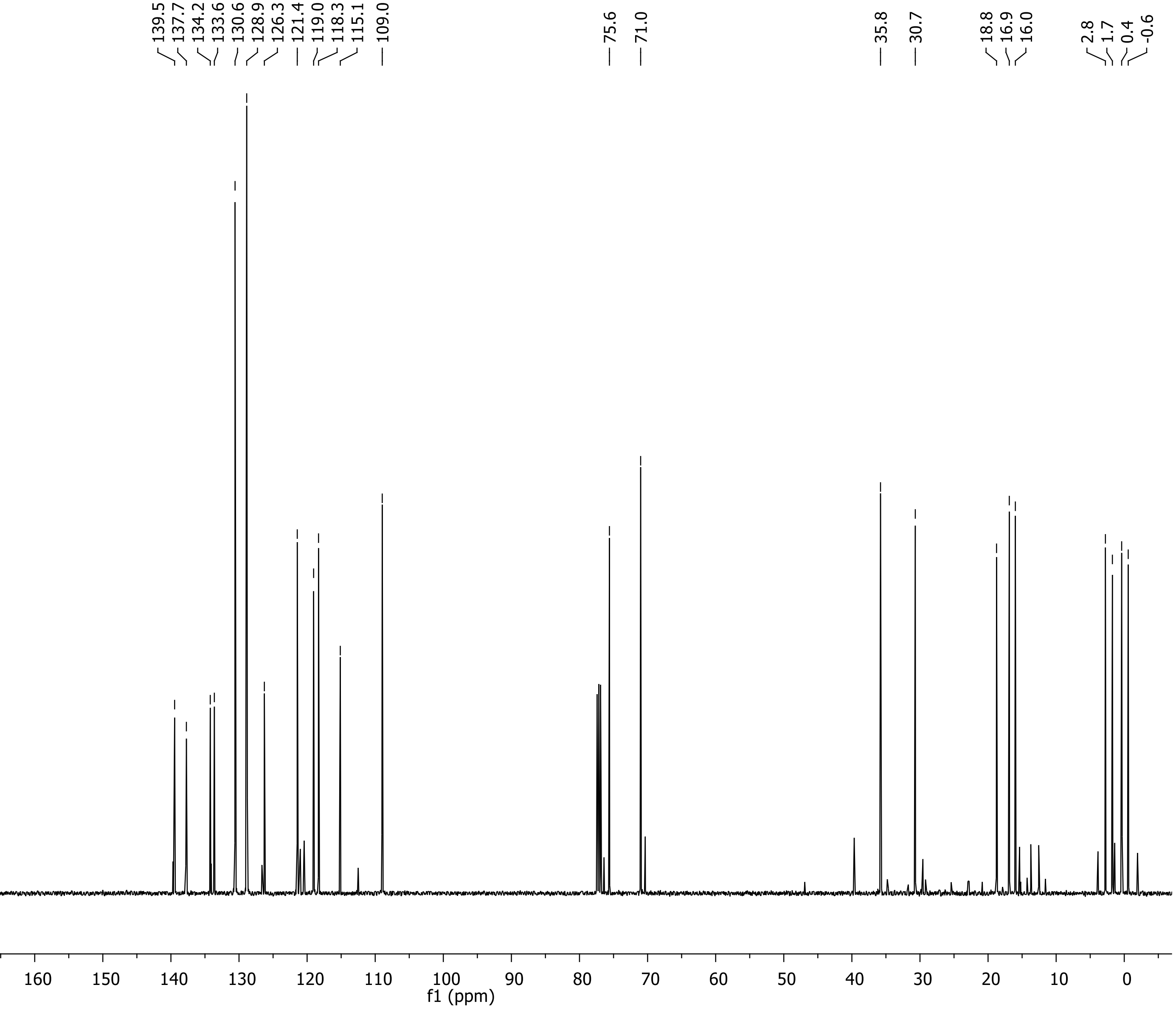

DEPT ($CDCl_3$, 125.7 MHz)

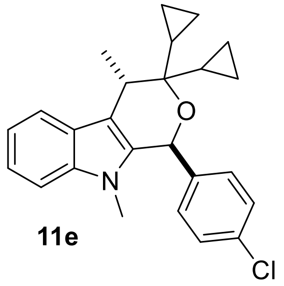

11e

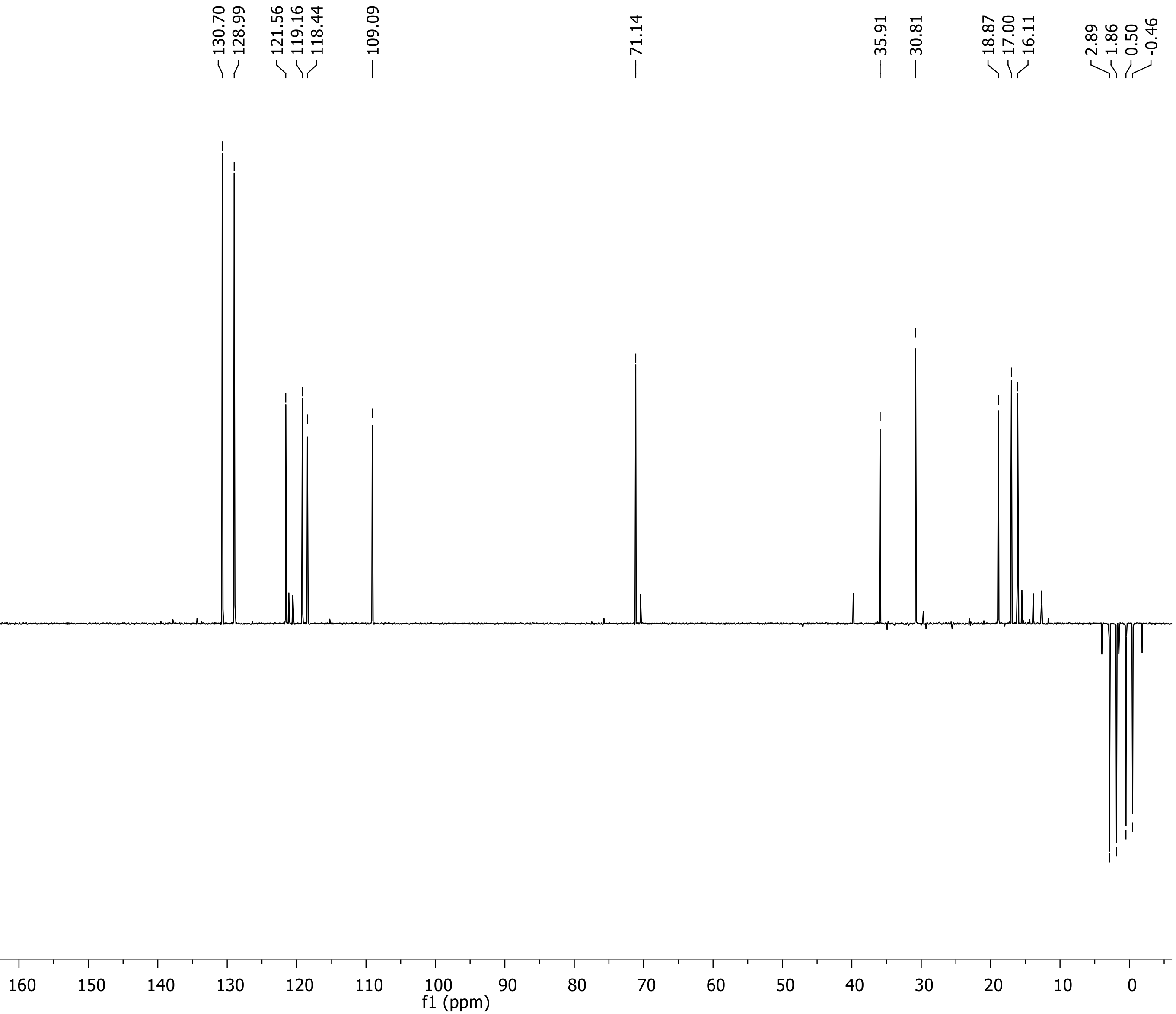

COSY ($CDCl_3$, 300 MHz)

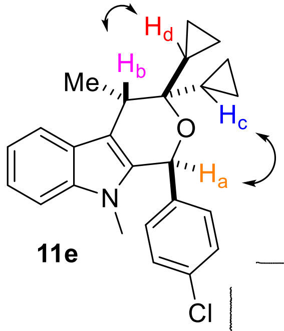

11e

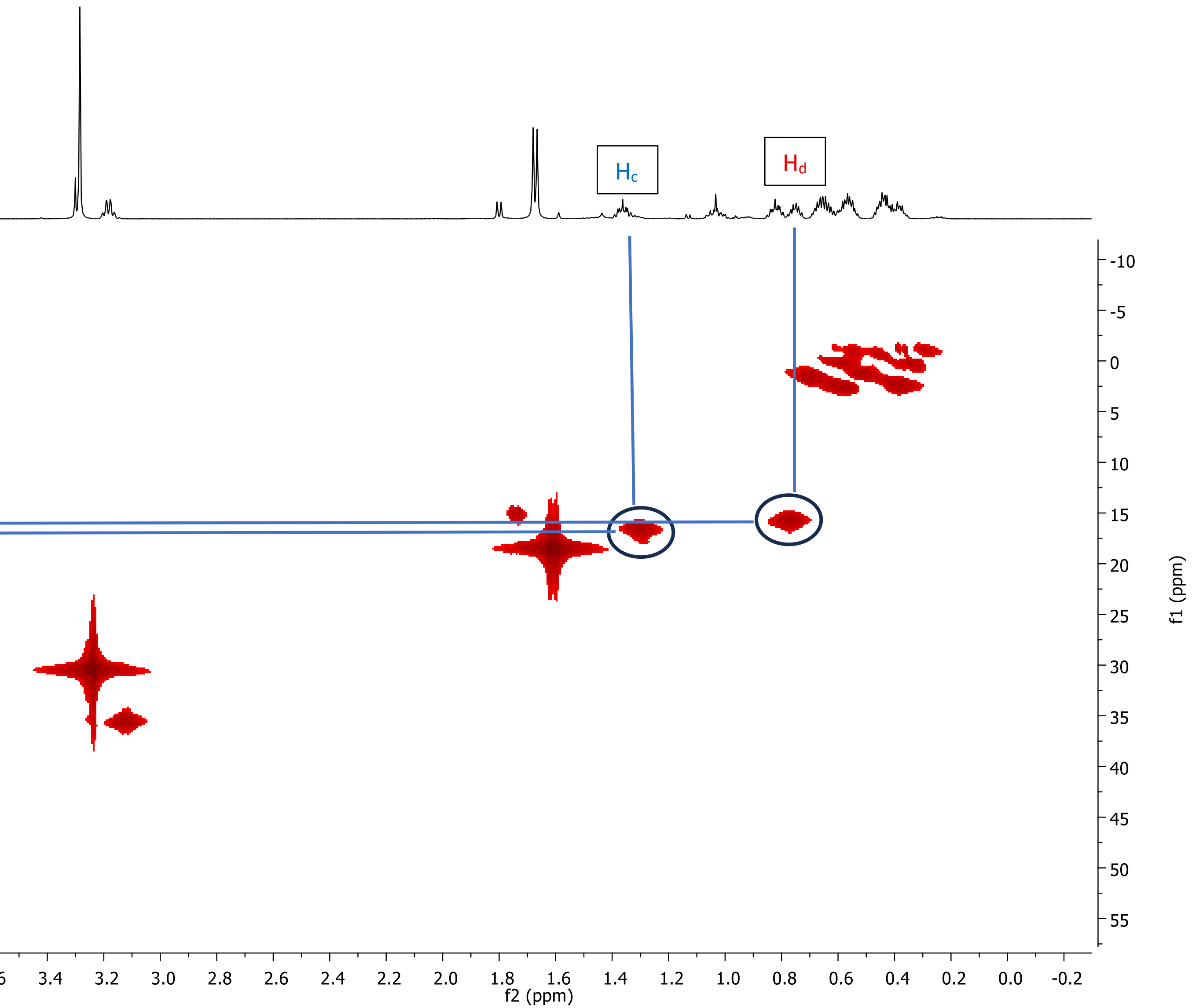

NOESY ($CDCl_3$, 300 MHz)

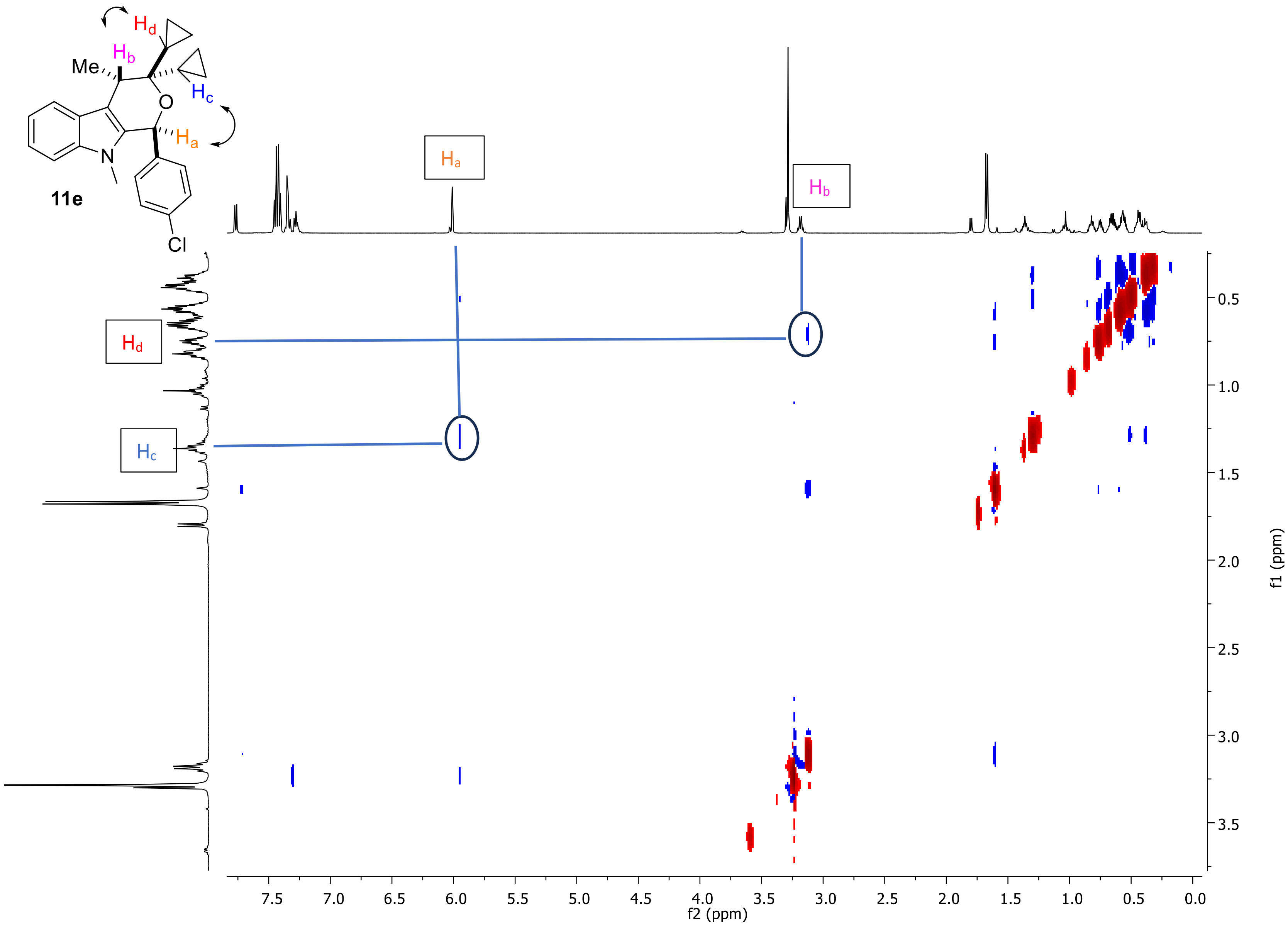

^{1}H NMR ($CDCl_3$, 300 MHz)

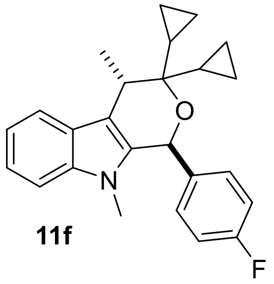

11f

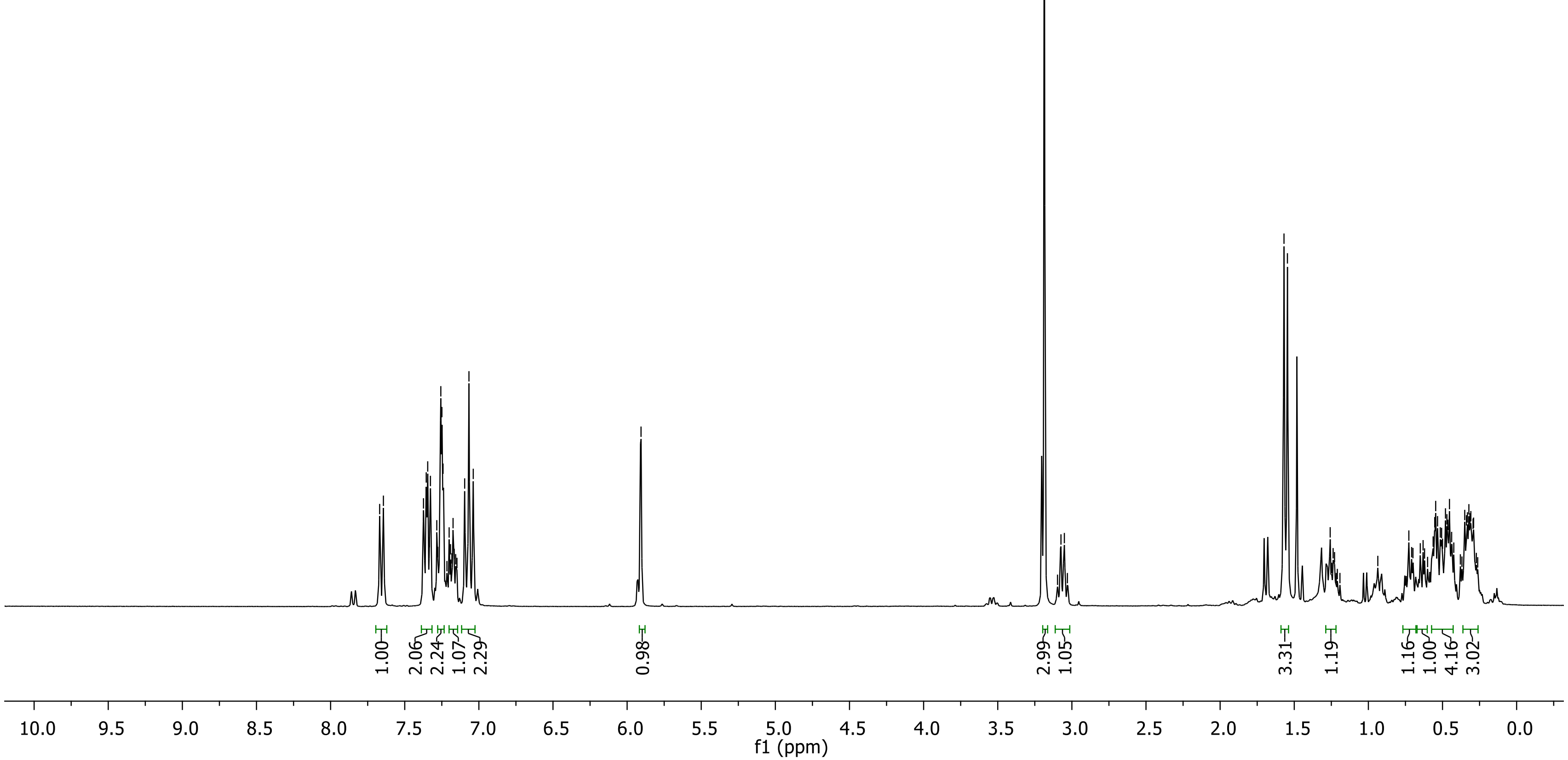

^{13}C NMR ($CDCl_3$, 75.4 MHz)

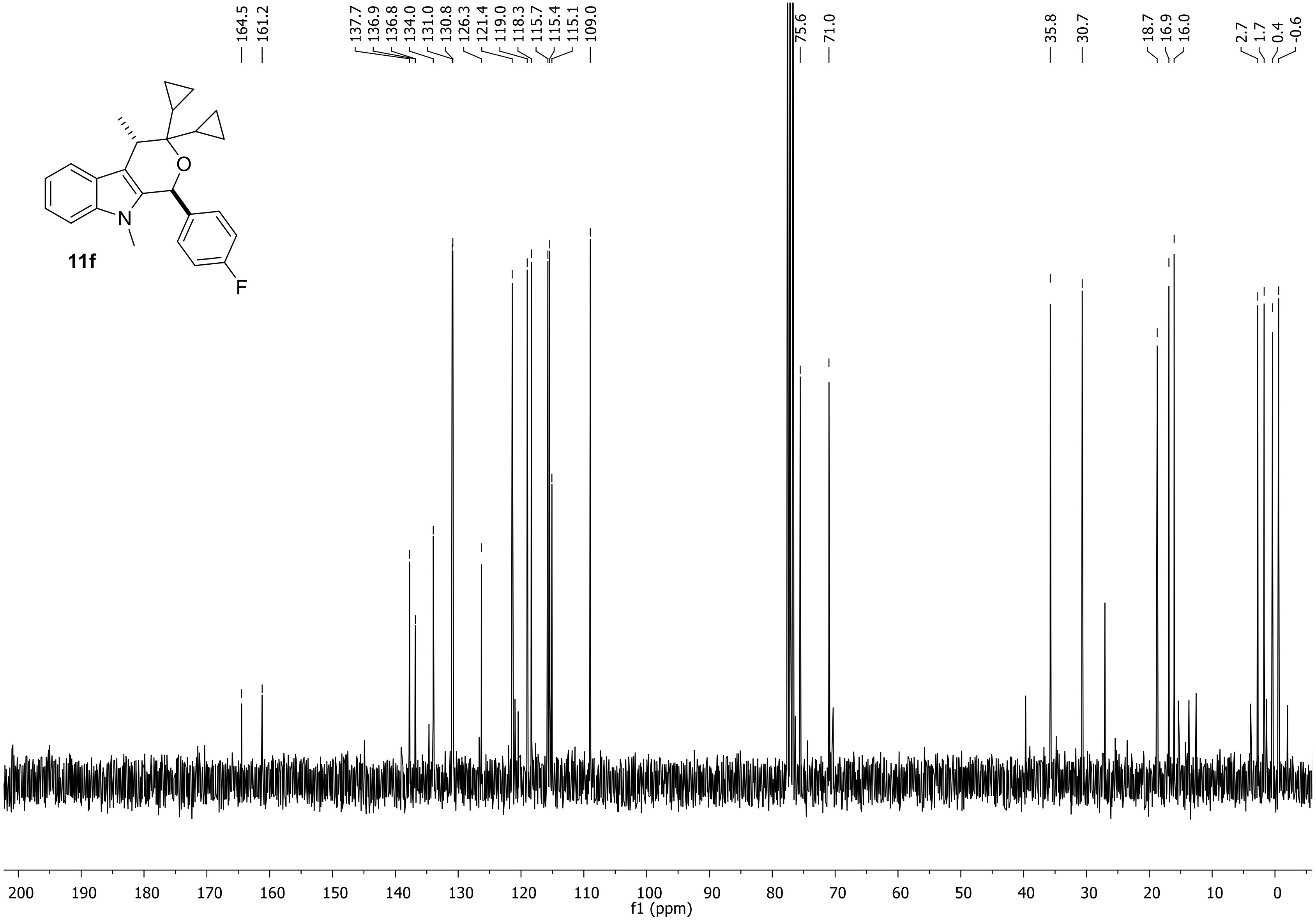

DEPT (CDCl$_3$, 75.4 MHz)

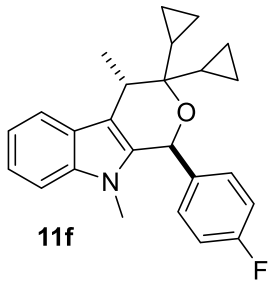

11f

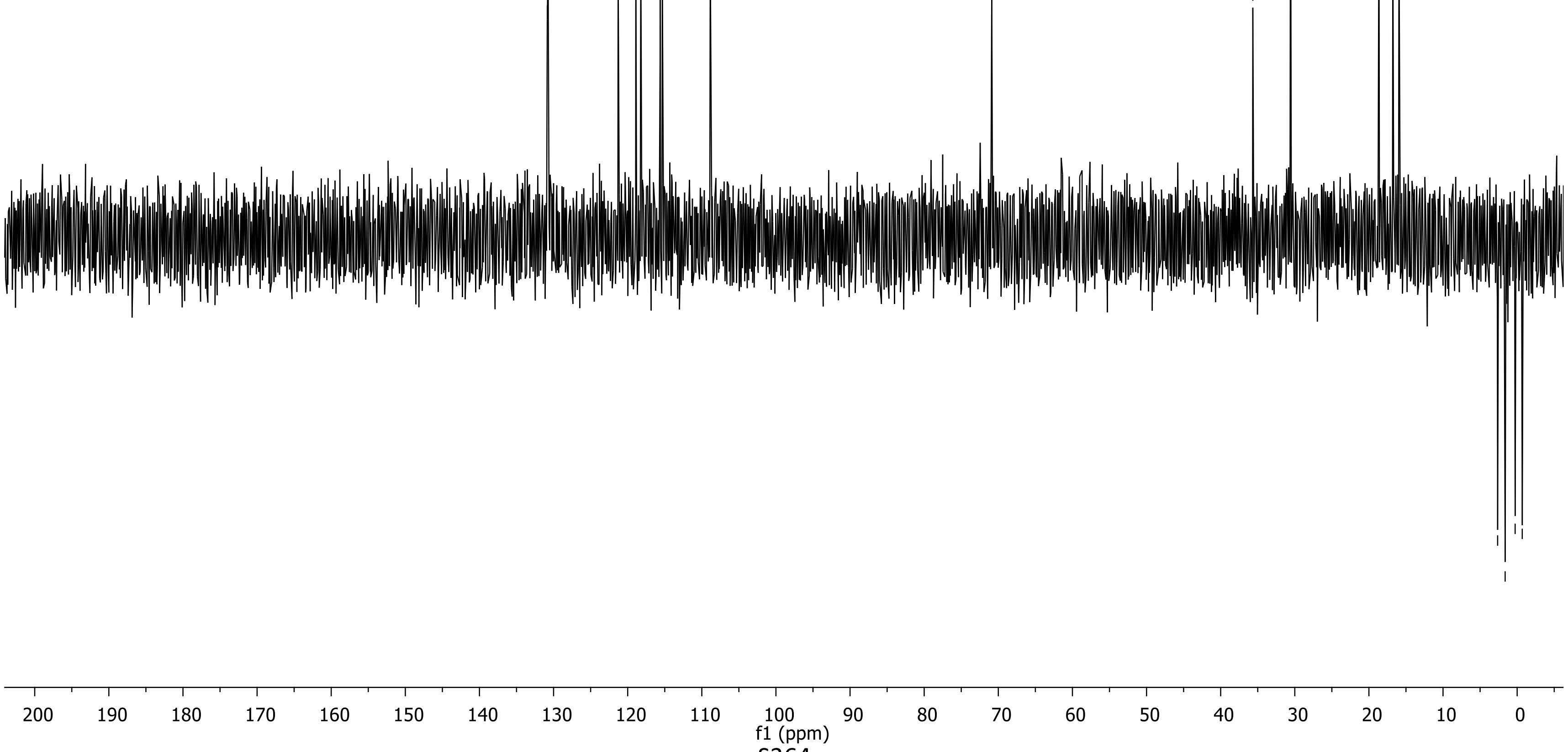

COSY (CDCl3, 300 MHz)

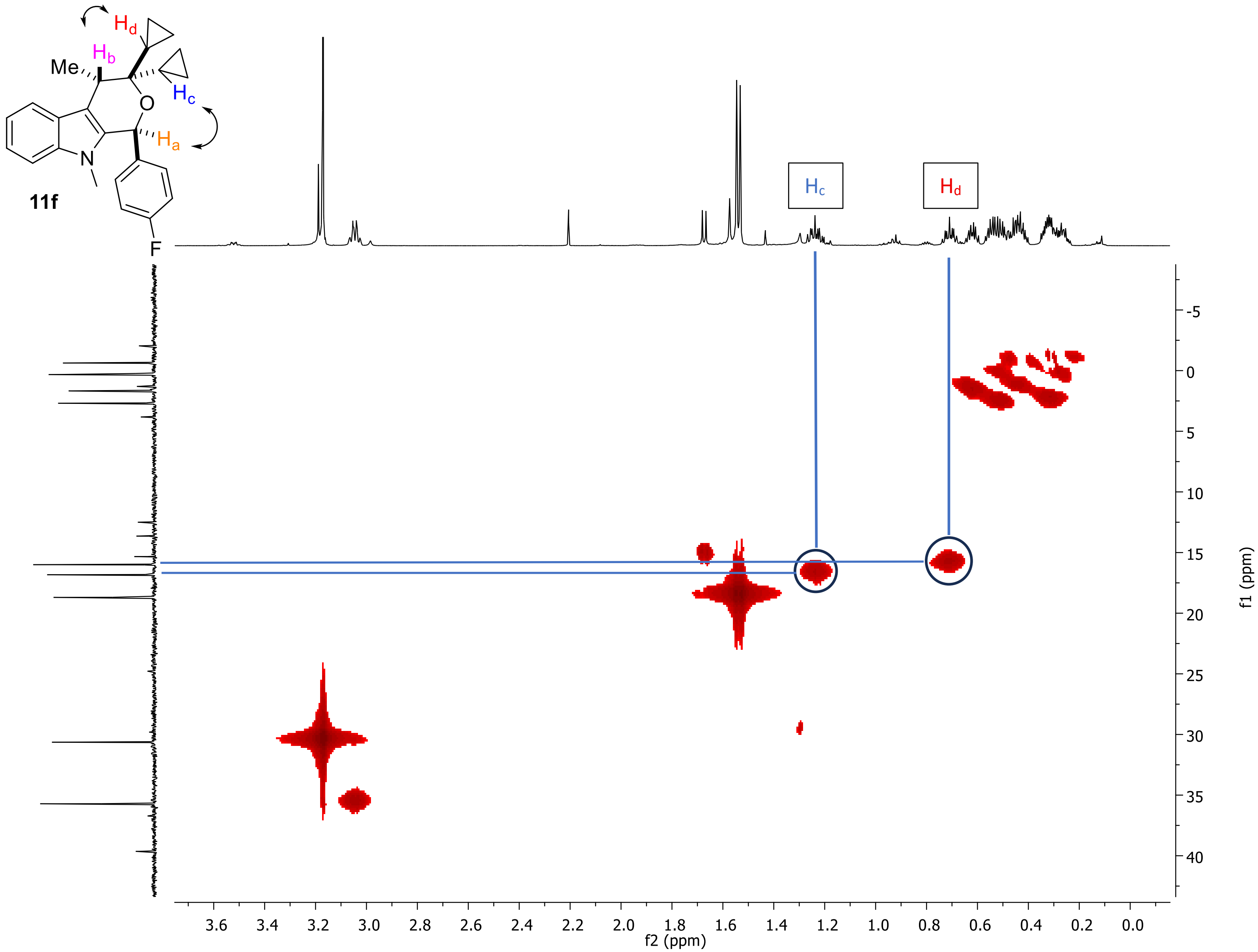

NOESY ($CDCl_3$, 300 MHz)

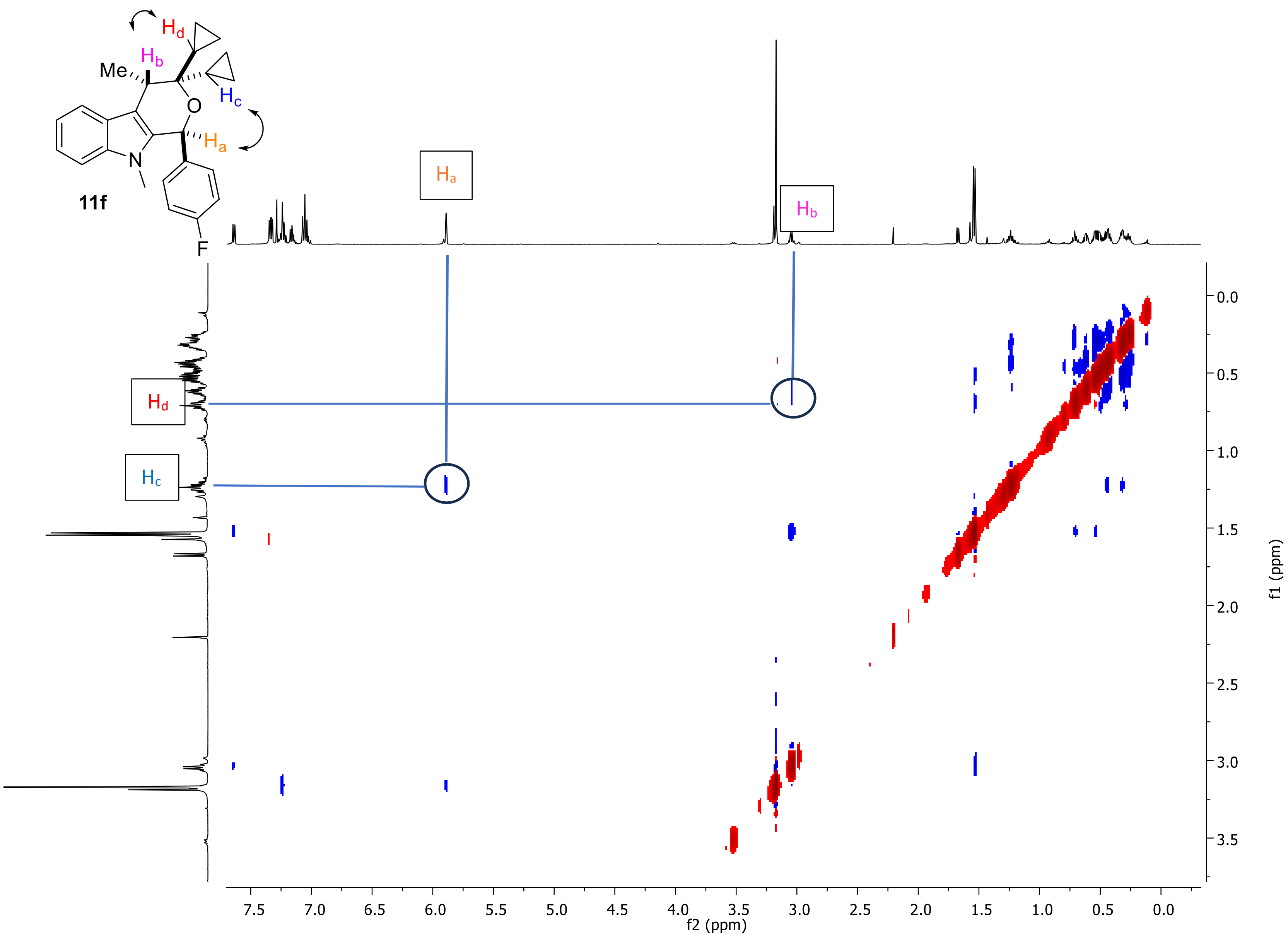

^1H NMR ($CDCl_3$, 300 MHz)

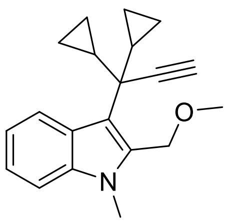

S23

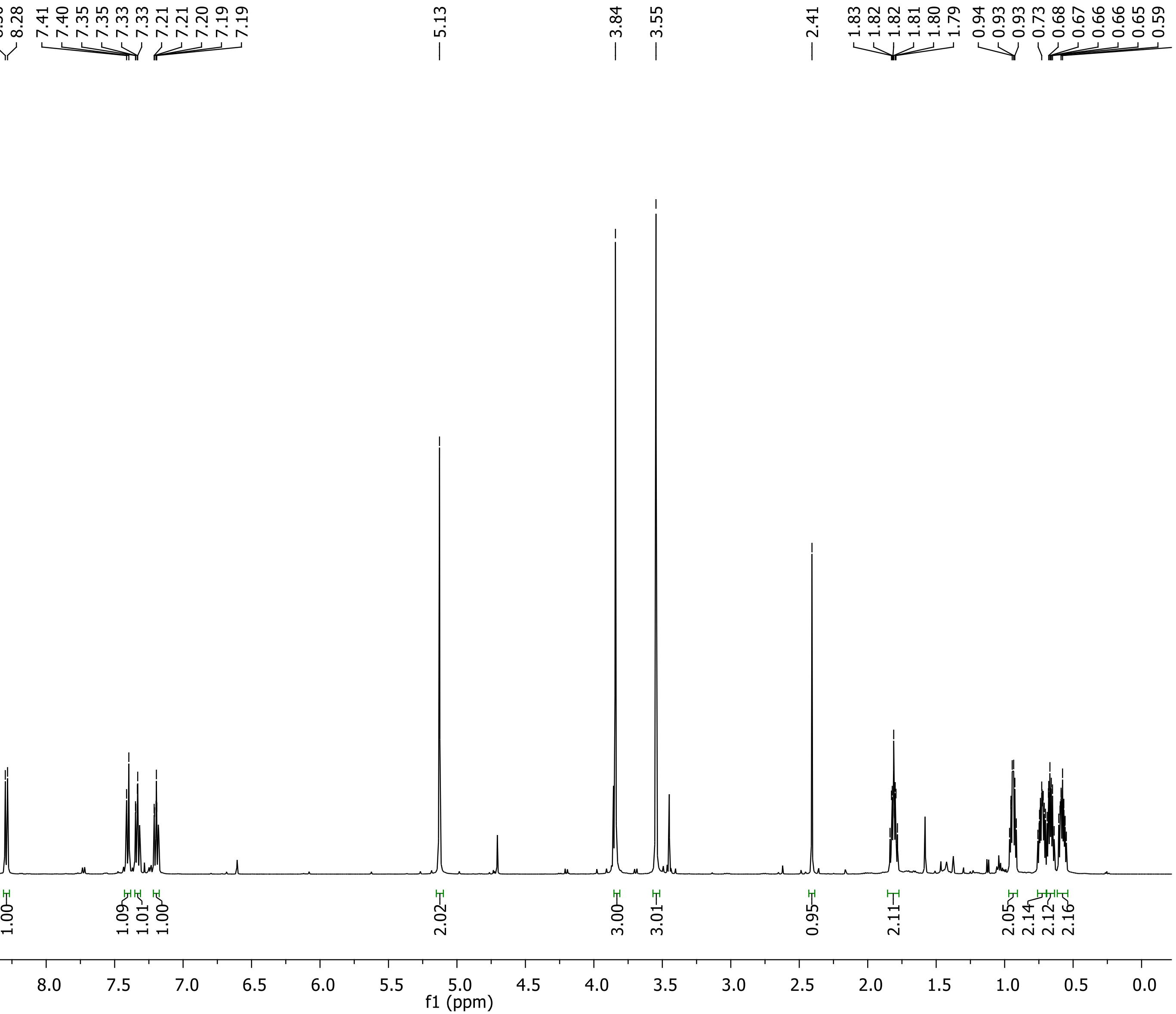

^{13}C NMR ($CDCl_3$, 75.4 MHz)

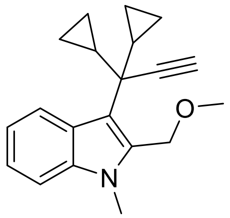

S23

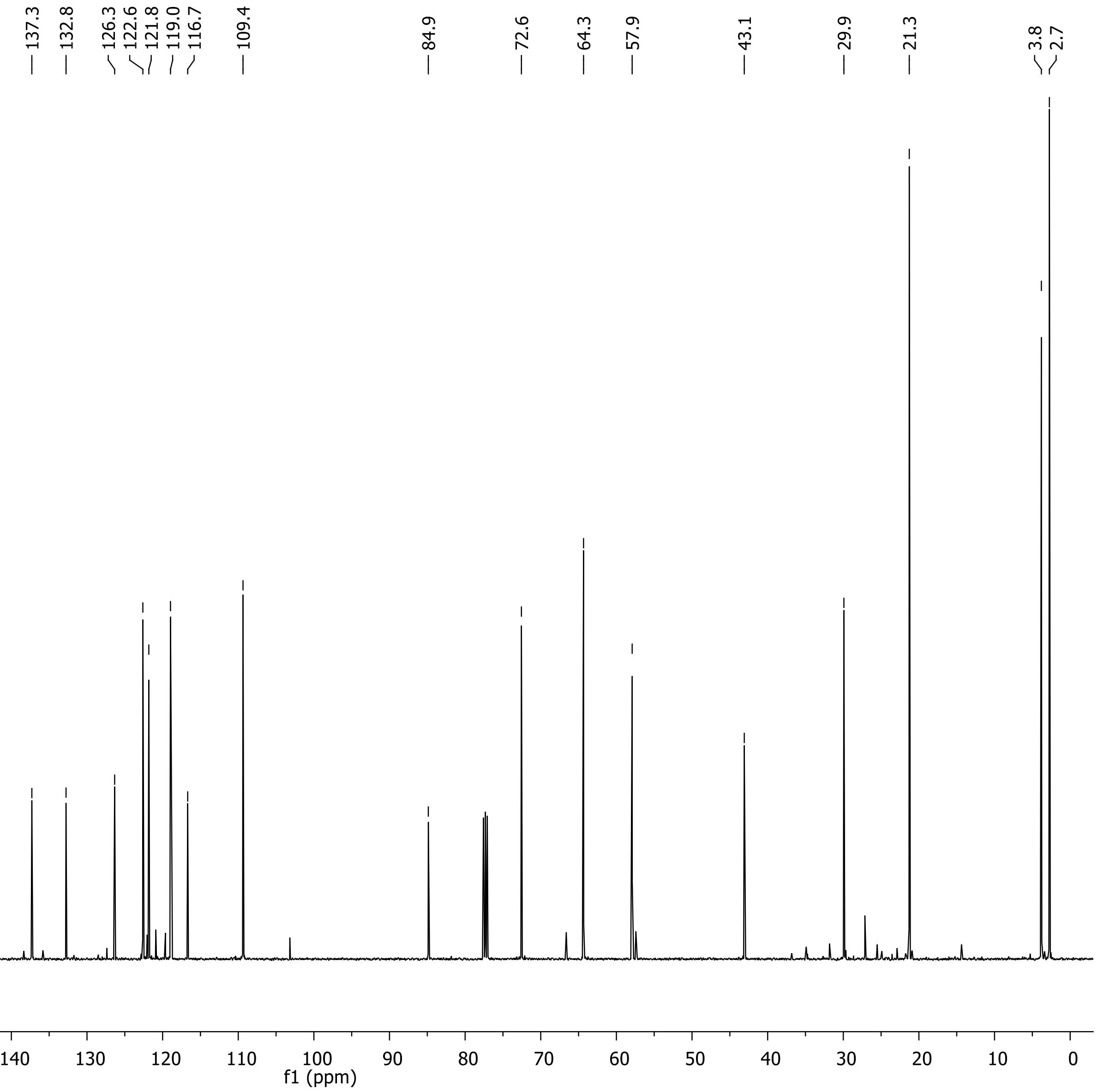

^1H NMR ($CDCl_3$, 300 MHz)

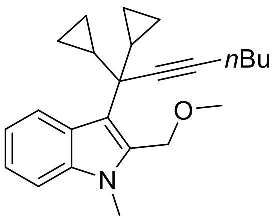

S24

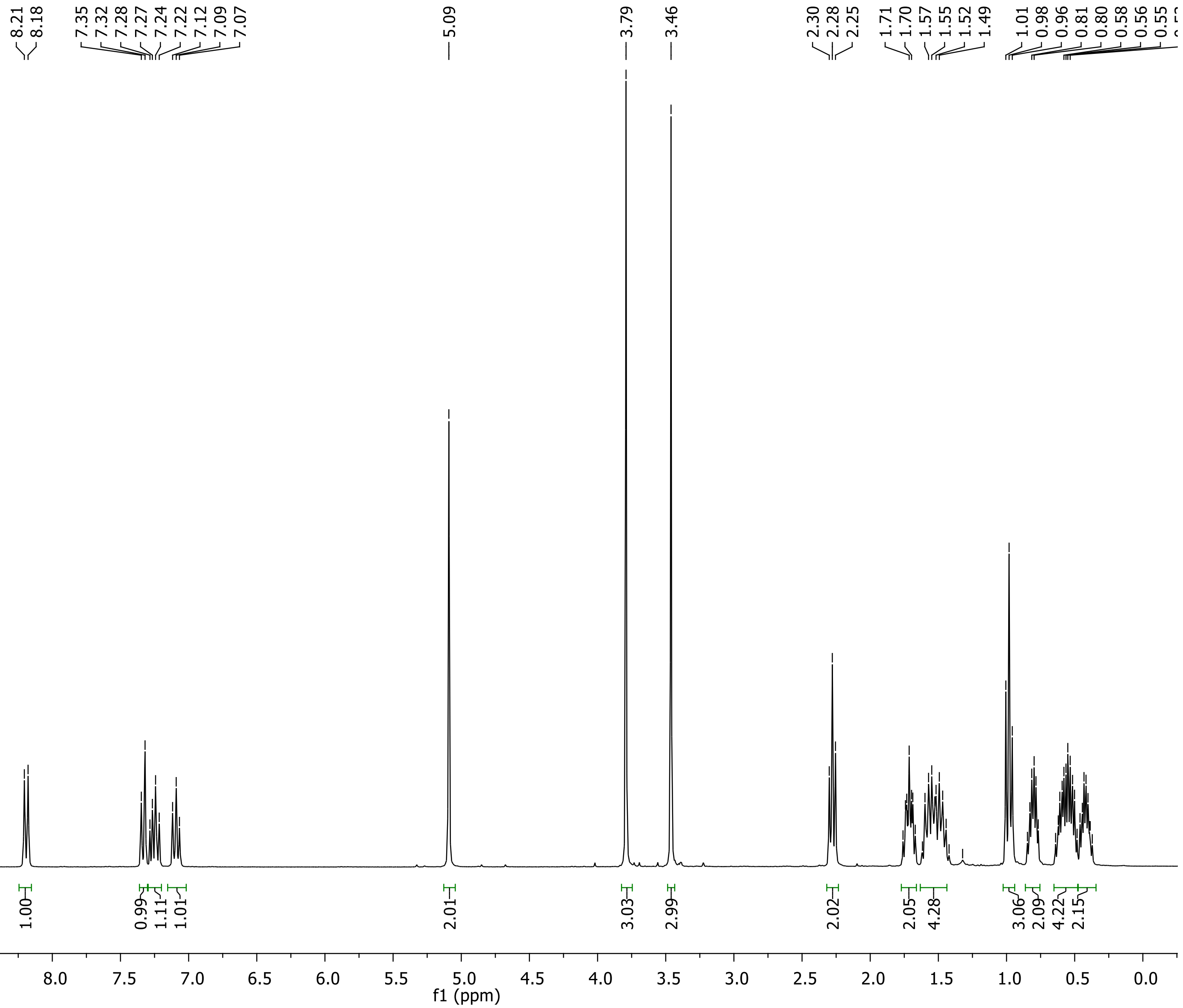

^{13}C NMR ($CDCl_3$, 75.4 MHz)

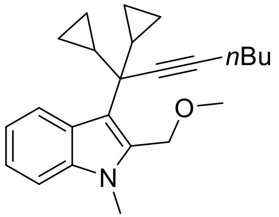

S24

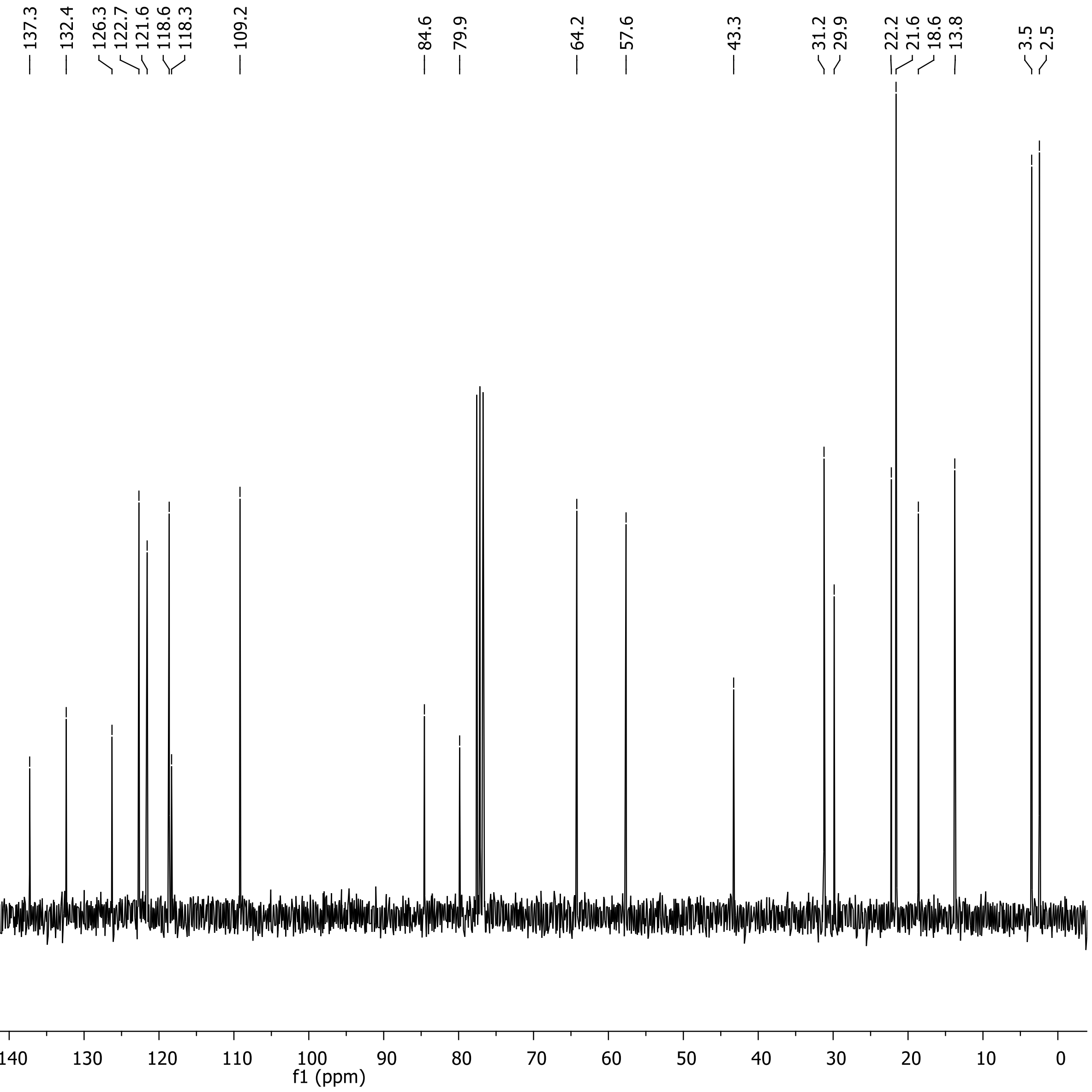

DEPT ($CDCl_3$, 75.4 MHz)

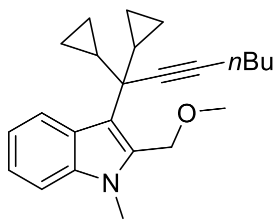

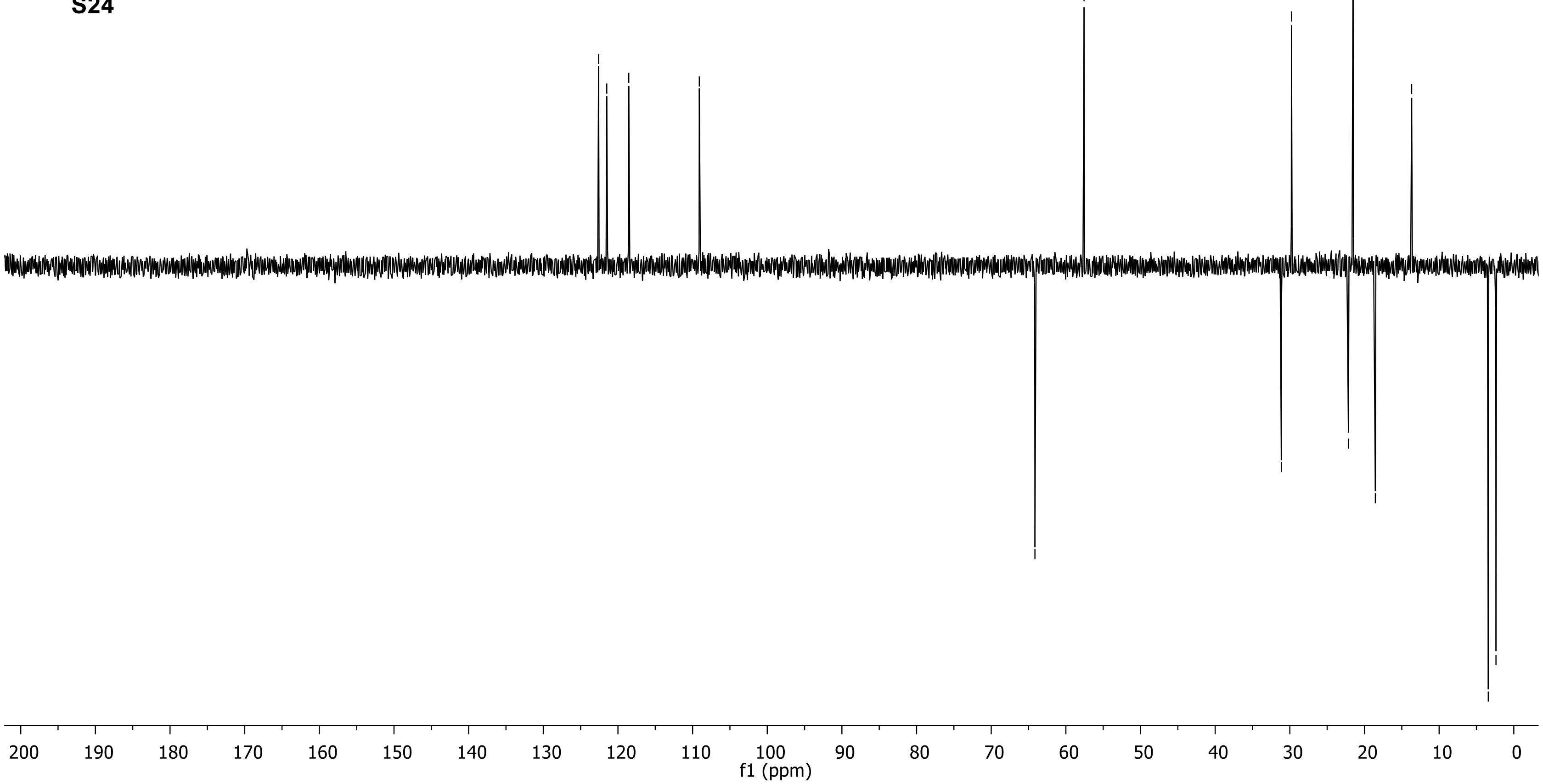

^{1}H NMR (CDCl$_3$, 300 MHz)

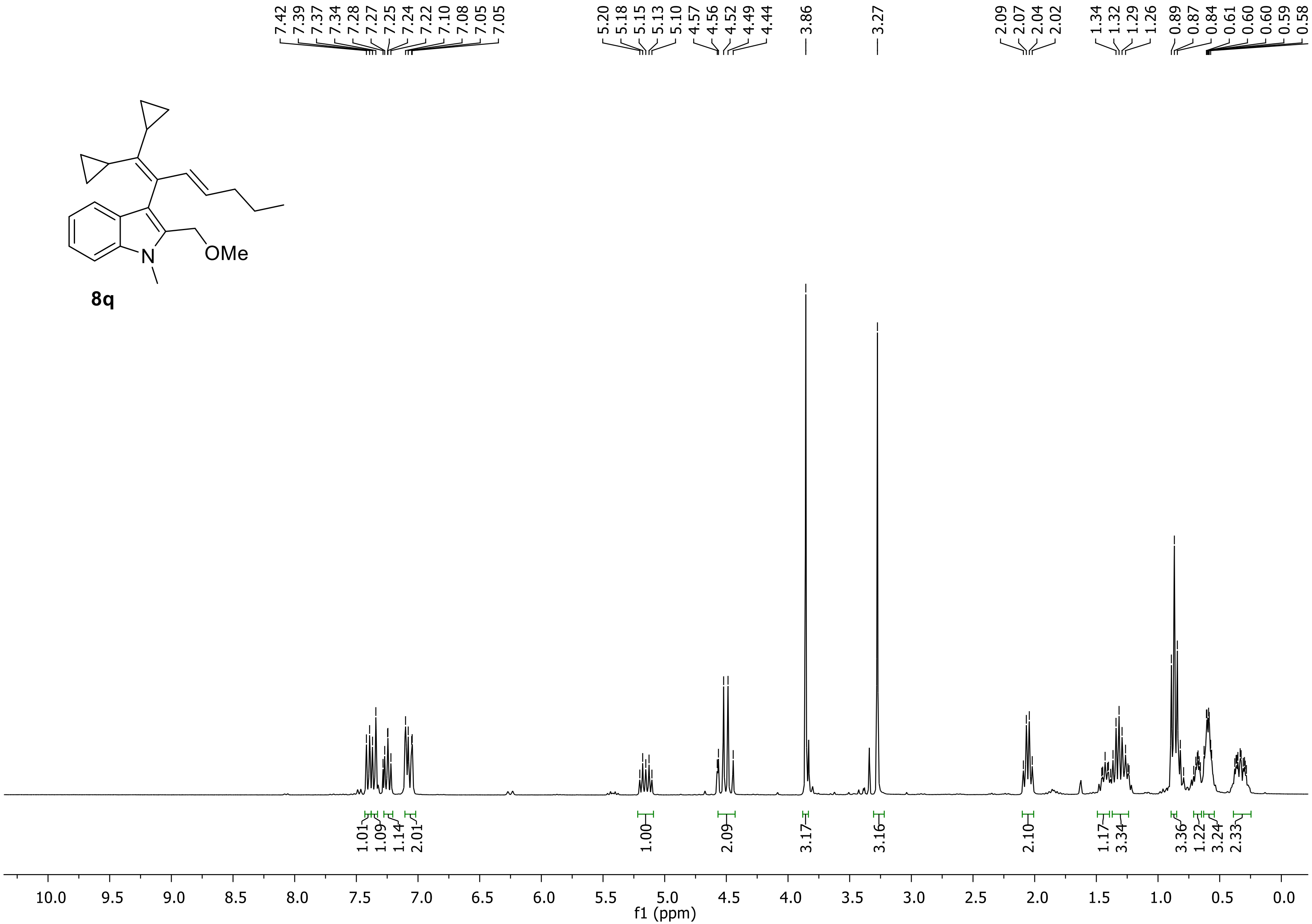

^{13}C NMR (CDCl$_3$, 75.4 MHz)

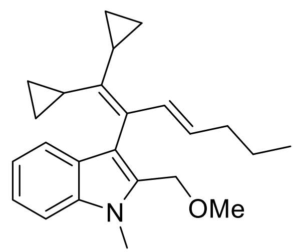

8q

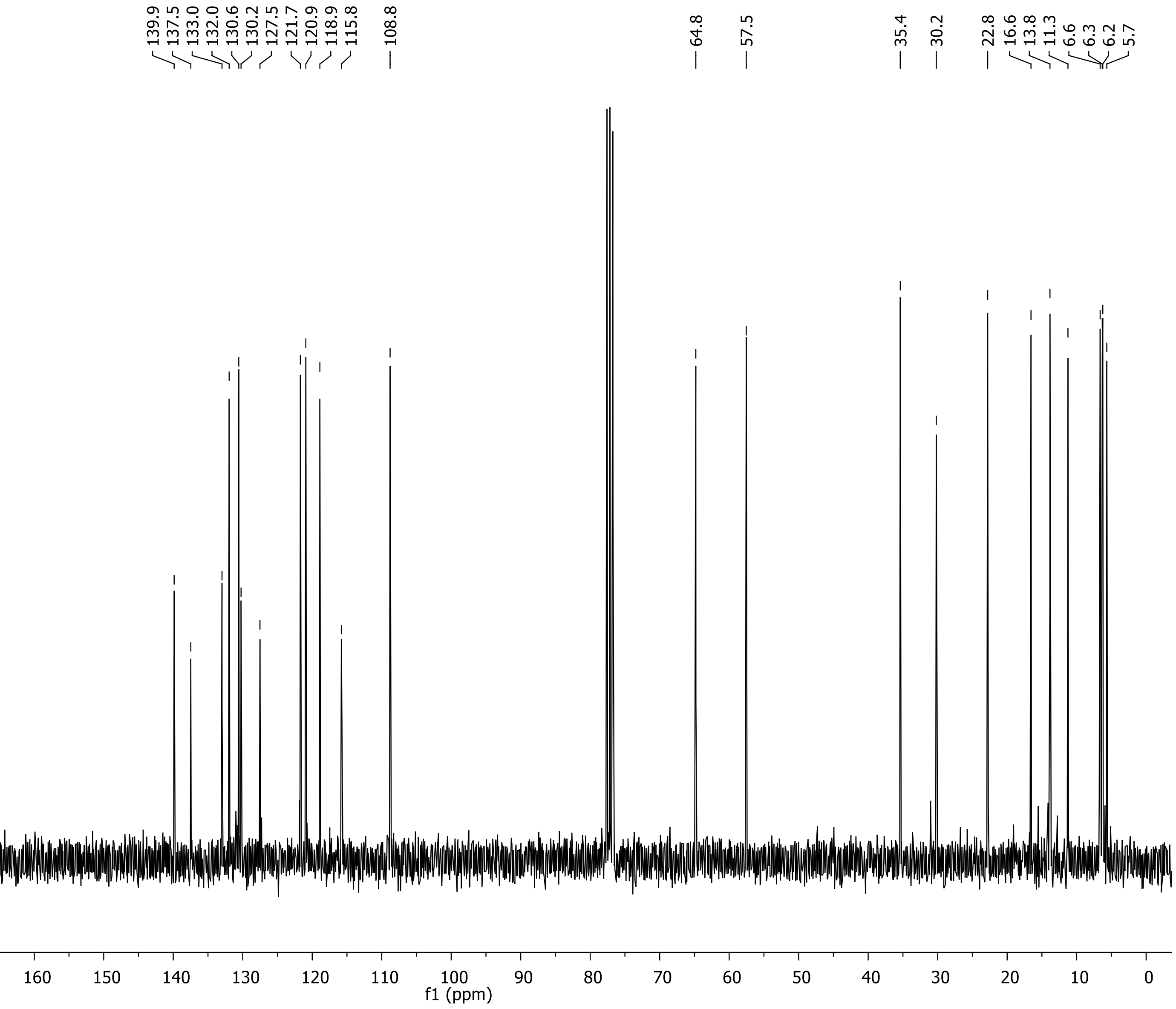

DEPT ($CDCl_3$, 75.4 MHz)

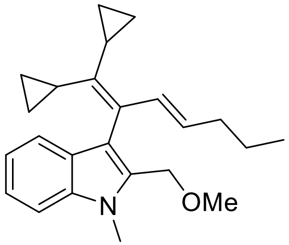

8q

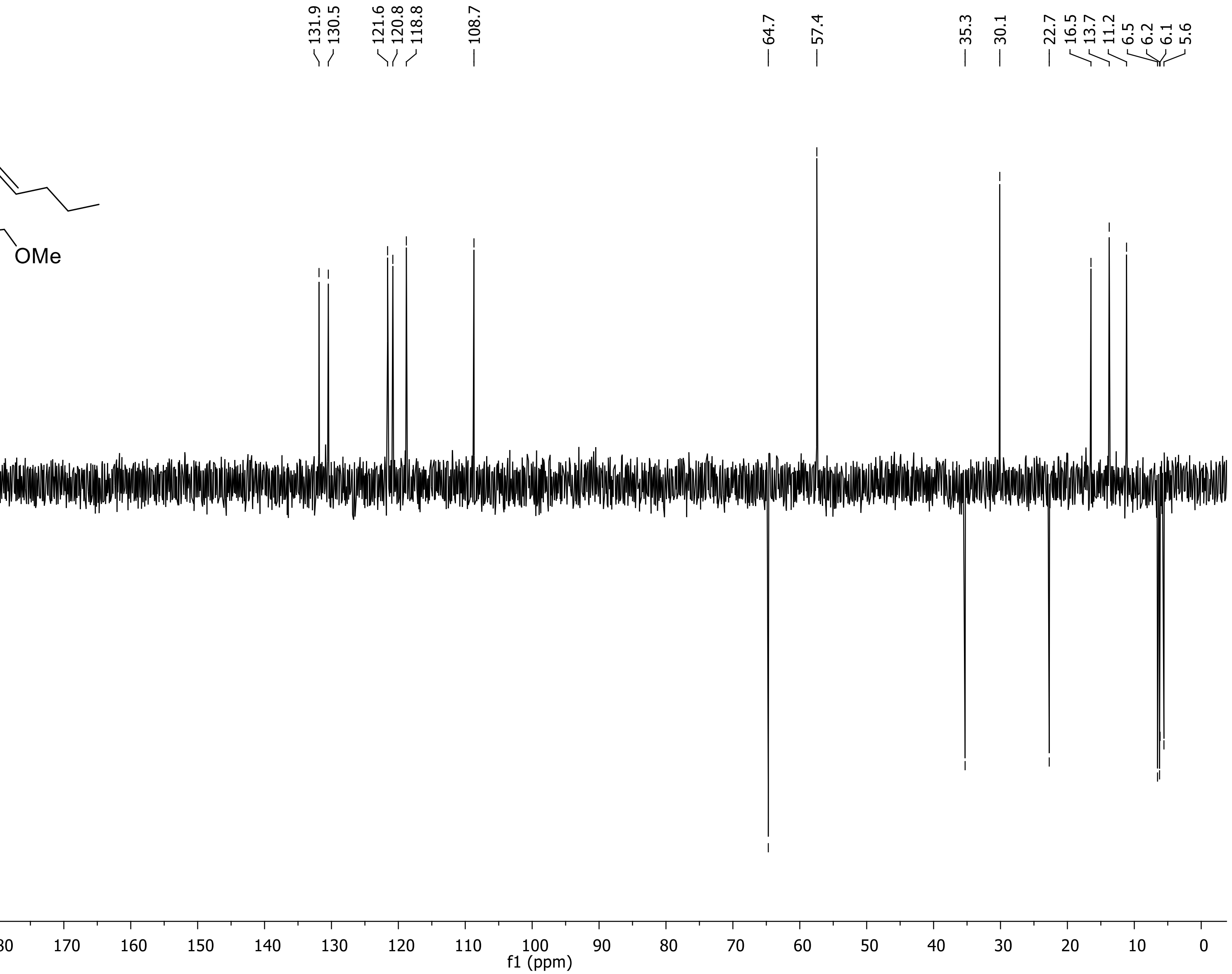

^{1}H NMR ($CDCl_3$, 300 MHz)

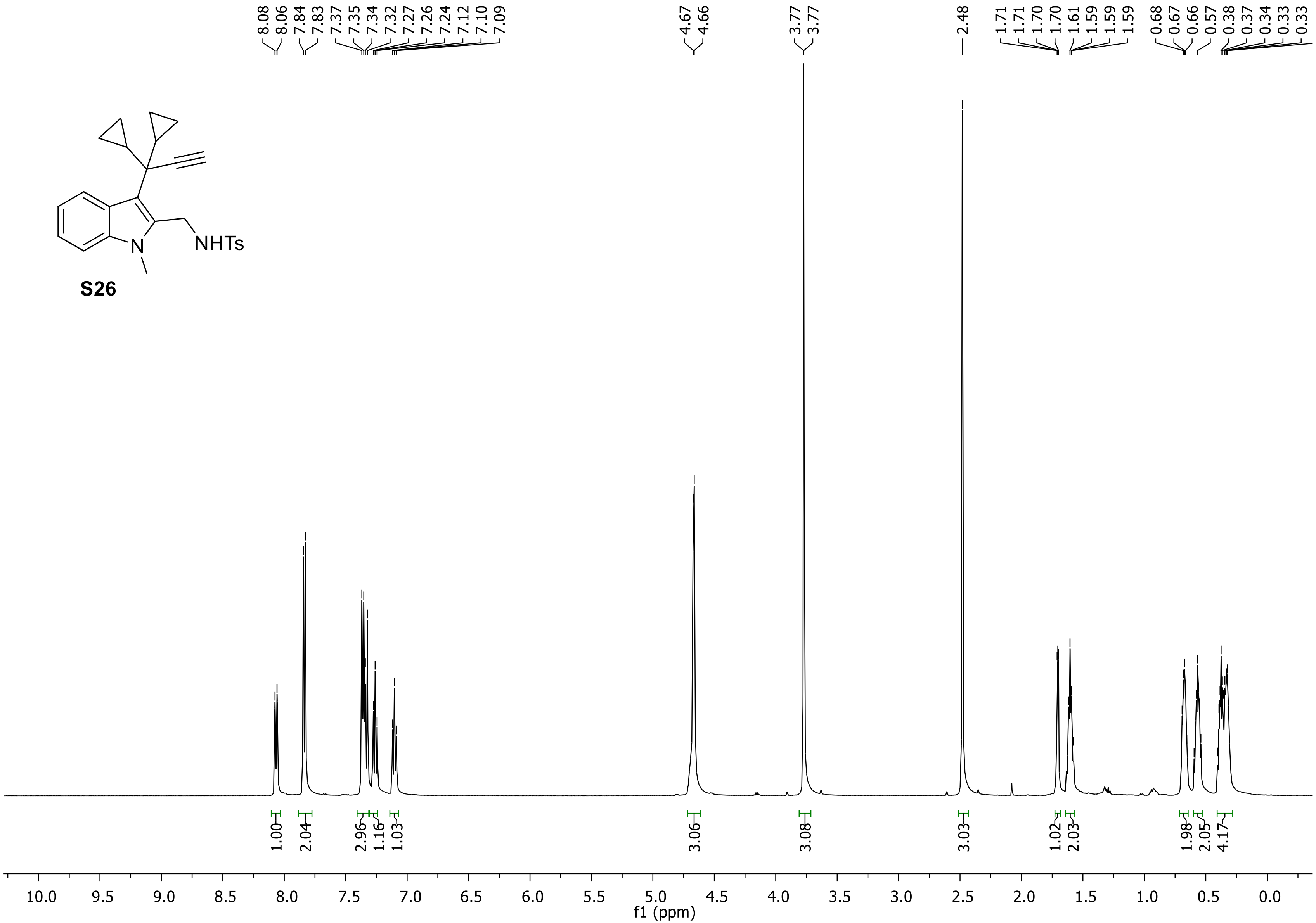

^{13}C NMR ($CDCl_3$, 75.4 MHz)

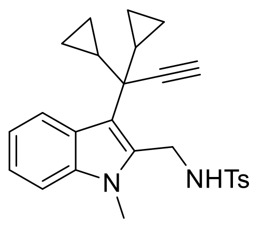

S26

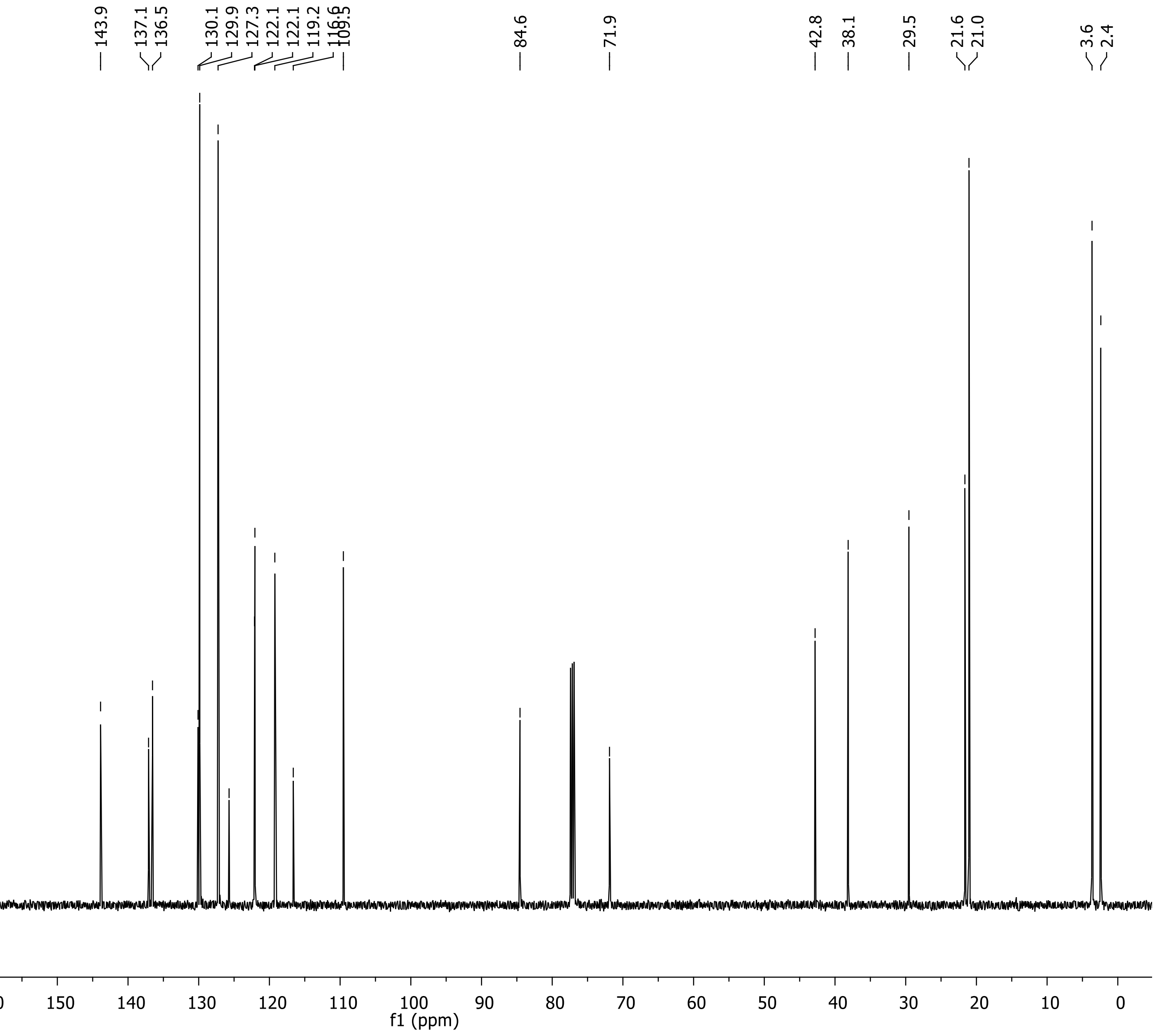

Supplement: Supplementary file 1 [file ol6c00991_si_001.pdf]
